# Direct Access to Iron Carbenes from Aldehyde, Ketone, and Formamide Feedstocks

**DOI:** 10.1021/jacs.5c21614

**Published:** 2026-04-28

**Authors:** P. Scott Pedersen, Katherine I. Burton, Sven H. M. Kaster, Eva Lin, Andria L. Pace, Marian C. Bryan, Taylor M. Sodano, Nicholas E. Intermaggio, Christopher B. Kelly, David W. C. MacMillan

**Affiliations:** † 6740 Merck Center for Catalysis at Princeton University, Princeton, New Jersey 08544, United States; ‡ Global Discovery Chemistry, Johnson & Johnson Innovative Medicine, Spring House, Pennsylvania 19477, United States; § Discovery Process Research, Johnson & Johnson Innovative Medicine, Spring House, Pennsylvania 19477, United States

## Abstract

Metallocarbenes are powerful intermediates for constructing complex molecular architectures, yet their generation typically relies on hazardous diazo or ylide precursors. We report photocatalytic and electrochemical strategies for direct carbene formation from unmodified carbonyl compounds using a low-valent iron system. The reaction proceeds through net oxidative addition of Fe­(I) into the carbonyl bond, followed by α-protonation and α-elimination to generate a reactive Fe–carbene intermediate. This platform enables cyclopropanation of diverse alkenes—including complex, drug-like, and amino-acid-derived substrates—under mild conditions, delivering highly functionalized, sp^3^-rich products. Mechanistic studies reveal direct carbonyl activation rather than free radical intermediacy, with a concerted–asynchronous olefin addition pathway. These findings establish a general strategy for carbonyl-to-carbene conversion, expanding the scope of base-metal catalysis and providing a blueprint for redefining carbonyl activation in synthetic chemistry.

## Graphical abstract

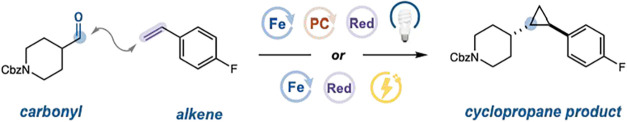

## Introduction

Carbenes—high-energy intermediates that underpin many of the field’s most powerful bond-forming reactions—have long been central to the construction of three-dimensional (3D) molecular architectures in pharmaceutical, agrochemical, and materials chemistry.^1–4^ Traditionally, access to free carbenes and metallocarbenes has relied on prefunctionalized carbene precursors, such as high-energy diazo compounds,^4–6^ geminal dihalides,^7–10^ sulfonium ylides,^11^ and related activated species.^12,13^ While these methods have enabled remarkable advances, the need for specialized, hazardous, or synthetically demanding precursors restricts the range of accessible carbene structures. Seminal work by Uyeda,^7,8^ Nagib,^9,10^ and others has established these precursors as mechanistically rich platforms capable of delivering a wide spectrum of carbene-based transformations. In contrast, a general method to generate carbenes directly from simple carbonyl compounds—particularly aldehydes—would be transformational, opening streamlined routes to reactive intermediates from abundant, stable, and inexpensive feedstocks.

Recent advances have established aldehydes as viable precursors to ketyl radicals, which can be intercepted by base-metal catalysts and, following α-elimination, furnish metallocarbene intermediates (Figure 1a).^14–20^ These strategies typically rely on ketyl-radical generation via boryl ligation or metal-based reduction, enabling simple carbonyl compounds to serve as carbene precursors. However, such approaches are limited by the requirement for both readily reducible substrates and sterically accessible ketyl-radical intermediates. Efficient interception of the ketyl radical by the metal porphyrin constrains steric accessibility.^15^ Because ketyl radicals are typically generated via reductive pathways, the precursor must be the most readily reduced species in solution. Under such conditions, competitive reduction of other components—including the metal catalyst—can occur, leading to catalyst deactivation and restricting the scope to substrates with accessible reduction potentials.^15,19,20^ To address this limitation, the Guo group developed an alternative strategy employing oxidizable boryl radical precursors that, upon oxidation, generate boryl radicals capable of reducing aldehydes to ketyl radicals.^17,18^ Despite this advance, sterically hindered substrates and more challenging-to-reduce carbonyls remain largely inaccessible, motivating the development of more general strategies for ketyl-radical generation from native carbonyls.

**Figure 1:**
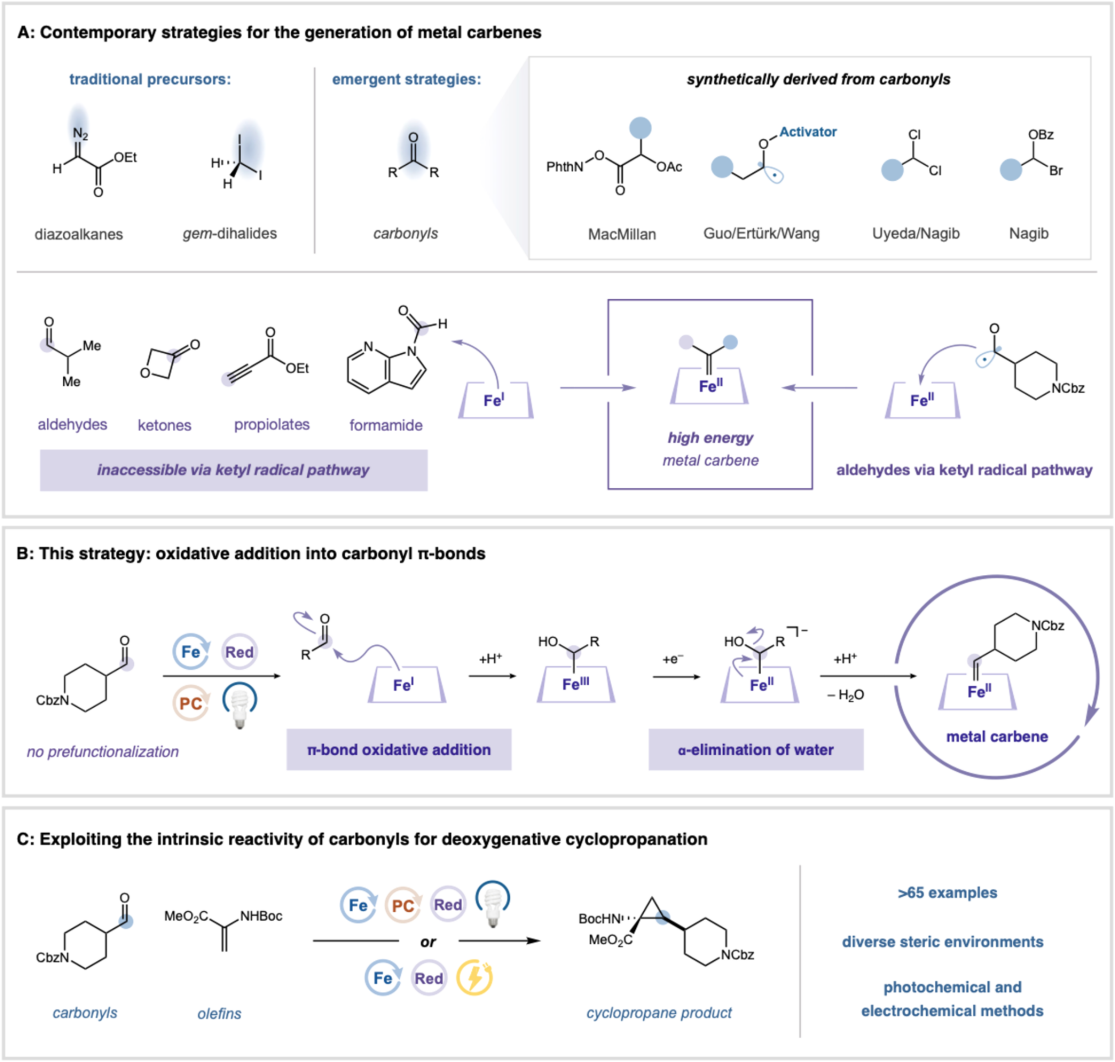
Photoredox-enabled carbonyl-to-carbene reactivity. Cyclopropanation of carbonyl feedstocks. PC = Photocatalyst, Red = reductant.

Other complementary approaches have emerged to circumvent these challenges. For example, the Zhuo group reported an elegant deoxygenative activation of preorganized 1,2-dicarbonyl scaffolds to generate molybdenum–carbene intermediates, enabling access to sterically congested systems. However, this approach is constrained by the need for preassembled substrates.^21,22^

We questioned whether an alternative paradigm could be realized in which low-valent metal species directly engage carbonyl functionalities, obviating the need for ketyl-radical intermediates. Specifically, we envisioned that photo- or electrochemically generated low-valent metals could undergo nucleophilic addition to carbonyl π-systems to form α-hydroxy metal intermediates, which upon protonation and subsequent α-elimination would furnish metallocarbenes. This design leverages the intrinsic polarity of the carbonyl group in conjunction with controlled metal redox states to enable a net oxidative addition across an electrophilic π-bond. Guided by our prior observation that nonclassical leaving groups are competent in iron-catalyzed carbene formation, we hypothesized that unactivated carbonyls could serve directly as carbene precursors under mild conditions.^23^ This approach would circumvent limitations associated with carbonyl reduction by instead gating reactivity through the generation of a nucleophilic, low-valent metal species, thereby enabling broader substrate generality. We were not alone in recognizing this opportunity; contemporaneously, the Xu group reported an electrochemical strategy for direct reduction of iron catalysts to generate metallocarbene intermediates.^24^ However, the scope of this transformation was limited to simple aldehydes, and superstoichiometric quantities were required to achieve reactivity. Cyclopropanation was further restricted to activated styrene acceptors, underscoring the challenges associated with promoting carbene transfer under fragment-coupling conditions.

Herein, we present complementary photocatalytic and electrochemical platforms that enable the direct, prefunctionalization-free conversion of carbonyls and propiolates to metallocarbenes via a mechanistically conserved π-bond nucleophilic addition (Figure 1a). This strategy allows structurally diverse carbonyl compounds to couple with olefins, affording cyclopropanes with high chemoselectivity. Key to the system’s success is a sterically encumbered Hantzsch ester, which suppresses undesired addition to styrenes, combined with an imidazole additive that promotes carbene transfer, likely through coordination as an apical ligand at the metal center. These features enable the formation of sterically diverse metallocarbenes from carbonyl substrates, while the enhanced carbene-transfer reactivity accommodates electronically deactivated olefin coupling partners. Mechanistic studies support direct activation of the carbonyl by a low-valent metal species, distinguishing this approach from ketyl-radical-based manifolds (Figure 1b). These insights and optimizations facilitate the incorporation of sterically hindered aldehydes, ketones, formamides, and propiolates, highlighting nucleophilic addition to π-bonds as a mechanistically enabling step. Together, these findings establish a general set of conditions that leverage π-bond addition by low-valent metal species as a broadly applicable strategy to access metallocarbene intermediates (Figure 1c).

## Reaction Design

The net oxidative addition of a metal across a carbonyl bond, though underutilized, has recently been leveraged with a variety of metals and has been applied to various cyclopropanation reactions.^21,22,24–26^ Our group and others have shown that photochemical or electrochemical reduction of Fe­(III) complexes generates nucleophilic Fe­(I) species capable of S_N_2-type oxidative addition.^24,25,27,28^ We hypothesized that intrinsically nucleophilic Fe­(I) could similarly engage carbonyls through a net oxidative addition. Importantly, stoichiometric precedents establish that Fe­(I) is competent in this transformation, suggesting that catalysis should be attainable.^25^

According to our reaction design, photocatalytic or electrochemical reduction of Fe­(III)-Cl could generate a low-valent, nucleophilic Fe­(I) complex capable of undergoing oxidative addition into the electrophilic carbonyl to form an Fe­(III)-alkyl intermediate. Protonation, reduction, and α-elimination furnish the metallocarbene, which reacts with an alkene to yield the cyclopropane. Finally, the resulting Fe­(II) species is photocatalytically or electrochemically reduced to Fe­(I) to close the catalytic cycle. A terminal reductant, such as a Hantzsch ester, enables this net reductive photoredox process.^29^

A general strategy for the direct activation of native carbonyls to carbenes would enable the broad and rapid diversification of carbonyls and olefins into complex cyclopropanated products. Cyclopropanes constitute an important class of small-molecule motifs due to their unique topology and wide utility in pharmaceuticals, materials science, and chemical biology.^30–33^ With this strategy, we envisioned enabling the streamlined synthesis and diversification of a wide variety of complex carbonyl and olefin starting materials, providing efficient access to structurally rich cyclopropane-containing scaffolds.

## Results and Discussion

### Reaction Development

We first examined cyclopropanation using limiting equivalents of aldehyde as the carbene precursor. Under blue-light irradiation, *tert*-butyl 4-(2-oxoethyl)­piperidine-1-carboxylate, Fe­(III) Octaethylporphine chloride, (Fe­(OEP)–Cl) (*E*_1/2_(Fe^II^/Fe^I^) = −1.12 V vs SCE),^27^ [Ir­(dF­(Me)­ppy)_2_(dtbbpy)]­PF_6_ (*E*_1/2_(Ir^III^/Ir^II^) = −1.41 V vs SCE),^34^ 1-fluoro-4-(prop-1-en-2-yl)­benzene, and a Hantzsch ester reductant, 2,6-DiMe-HEH (diethyl 2,6-dimethyl-1,4-dihydropyridine-3,5-dicarboxylate), were combined in methanol to give the desired cyclopropane in only trace yield (Table 1, entry 1). Replacing methanol with tert-amyl alcohol to suppress acetal formation, adding benzonitrile to improve Fe­(OEP)–Cl solubility, and introducing water as a proton source led to an increase in yield to 32% (entry 2). We hypothesized that the Hantzsch ester reductant was reversibly sequestering the aldehyde, thereby diminishing the productive carbene formation. To disrupt this deleterious pathway, we introduced steric bulk adjacent to the reductant nitrogen. Indeed, employing 2,6-iPr-HEH (diethyl 2,6-diisopropyl-1,4-dihydropyridine-3,5-dicarboxylate) as the sacrificial reductant led to full aldehyde consumption and a modest increase in yield (entry 3).^35^ However, these conditions lacked generality across diverse aldehyde–olefin combinations. Reasoning that accelerating carbene transfer might enhance the overall efficiency, we drew inspiration from the proximal imidazole coordination used in heme enzymes. Incorporation of an imidazole additive significantly promoted carbene delivery and improved reactivity, providing cyclopropanes in up to 98% assay yield (entry 4).^36–41^ Control experiments omitting Fe­(OEP)–Cl, photocatalyst, or light resulted in no detectable product (entries 5–7), consistent with a metallaphotoredox mechanism. Other Fe–porphyrin catalysts were competent but afforded reduced yields (entry 8).

**Table 1:**
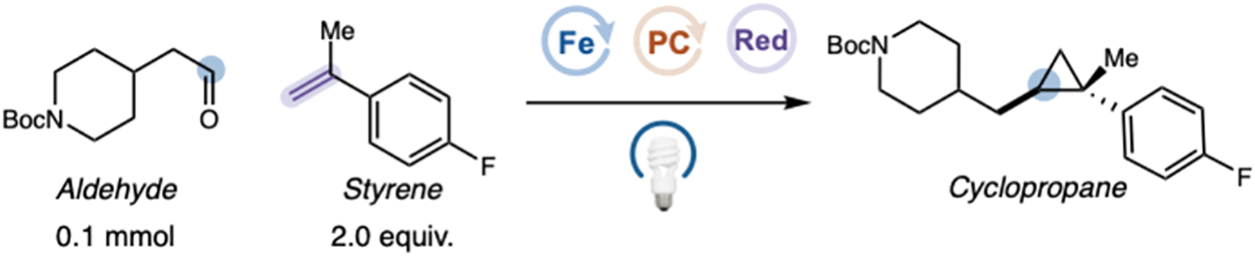
Reaction Optimization

entry^b^	deviation	yield[Table-fn t1fn3]
1	no imidazole, 2,6-DiMe-HEH, methanol	<5%
2	no imidazole, 2,6-DiMe-HEH	32%
3	no imidazole	42%
4	none	98%
5	no Fe(OEP)-Cl	0%
6	no lr(dFMeppy)_2_(dtbbpy)PF_6_(1 mol %)	0%
7	no light	0%
8	Fe(TPP)-Cl	68%

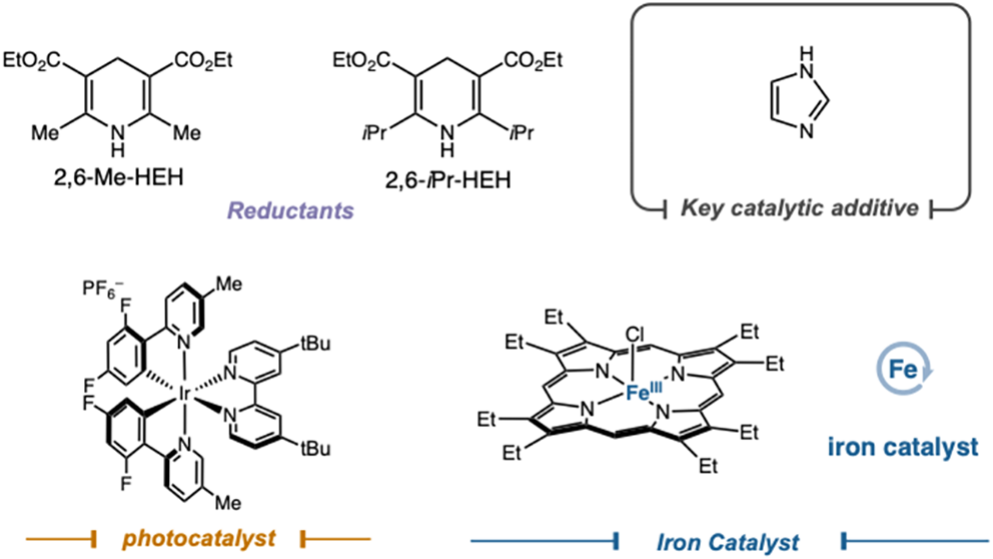

a Performed on 0.1 mmol scale.

b Assay yield determined versus 1,4-difluorobenzene internal standard. Standard conditions: Fe­(OEP)Cl (7.5 mol %), imidazole (7.5 mol %), *tert*-butyl 4-(2-oxoethyl)­piperidine-1-carboxylate (1.0 equiv), Ir­(dFMeppy)_2_(dtbbpy)­PF_6_ (1 mol %), 1-fluoro-4-(prop-1-en-2-yl)­benzene (2.0 equiv), and Hantzsch ester (2.5 equiv) in tAmylOH/PhCN/H_2_O (18:2:1 volumetric ratio, (0.3M)) for 18 h at 450 nm (20% light intensity, M2 generation plates, 2000 rpm stir rate, fans 1500 rpm). Fe­(OEP)-Cl = Fe­(III) Octaethylporphyrin chloride. Fe­(TPP)-Cl = Fe­(III) Tetraphenylporphyrinchloride.

### Reaction Scope

With the optimal conditions in hand, we evaluated the scope of the reaction (Figure 2). Commercially available aqueous formaldehyde, an attractive one-carbon addition reagent, was effective as a carbene precursor in reactions with 1,1-disubstituted styrenes (**1**). Additionally, other valuable classes of olefins, including enamines (**2**), internal olefin containing 1,2 dihydropyridines (**3**) and 1,1-alkyl-substituted olefins (**4**), were efficiently engaged as coupling partners under the cyclopropanation conditions. As amines are key diversification handles in pharmaceutical settings, we were pleased to demonstrate the compatibility of acid-labile Boc-protected amines (**5**). Aldehydes bearing amine functionality provided high yields, and even Fmoc-protected substrates—typically sensitive to photochemical conditions—were well tolerated (**6**,**7**). We next assessed the steric limits of the reaction. Ketyl-radical approaches often show steric sensitivity due to competing side reactions of the high-energy intermediate.^15^ In contrast, our mechanism, which avoids a free ketyl radical, proved to be highly tolerant of steric demand. Numerous substrates bearing α-tertiary carbons were tolerated (**8–15**), as were aldehydes bearing α-quaternary centers (**16**). We also demonstrated fragment coupling of aldehydes derived from complex molecules, highlighting the potential for late-stage diversification (**17**, **18**).

**Figure 2:**
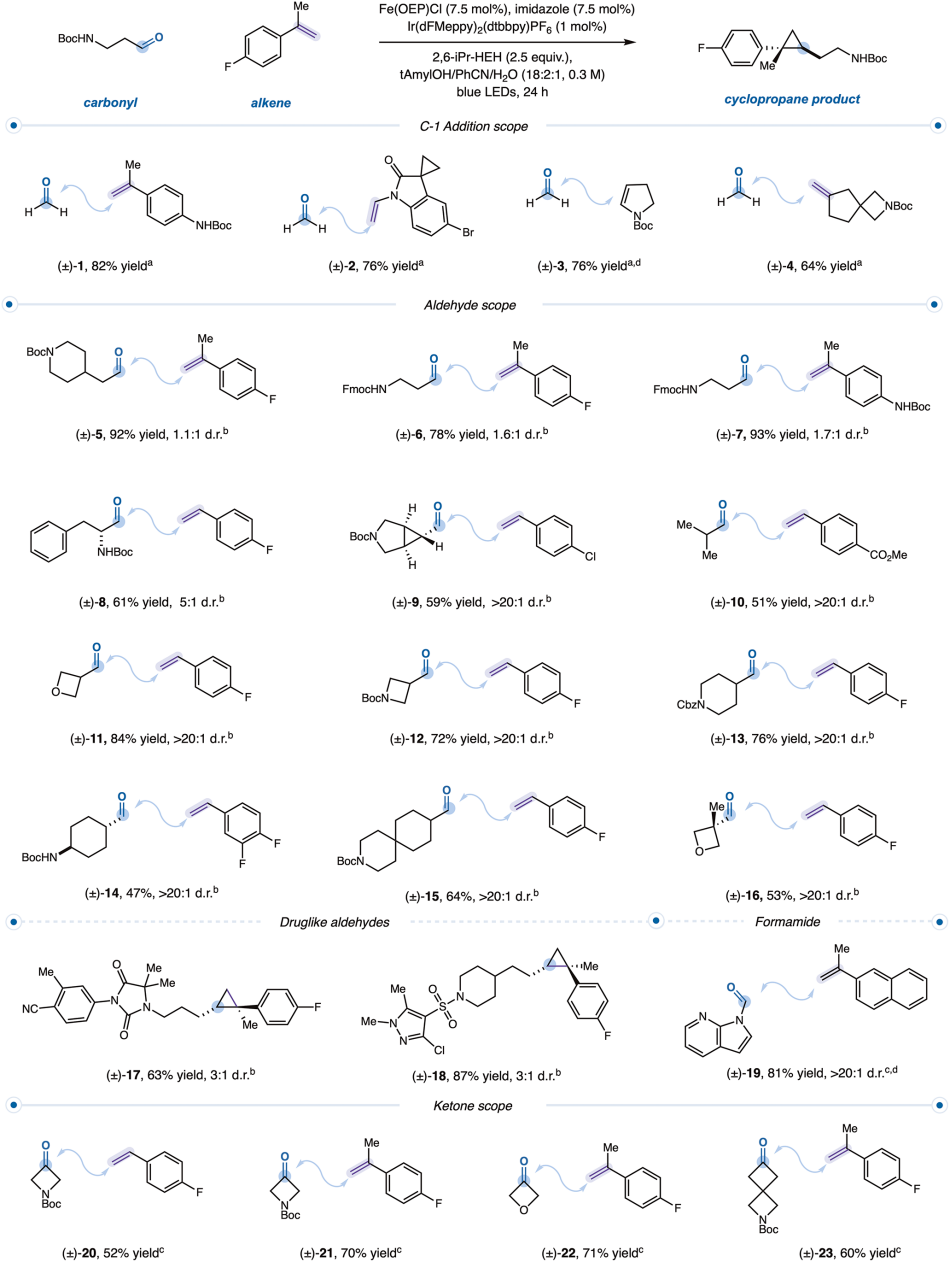
Scope of carbene precursors for cyclopropane formation All isolated yields are reported. See Supporting Information for experimental details. ^a^Performed according to general procedure **D**. ^b^Performed according to general procedure **A**. ^c^Performed according to general procedure **B**. ^d^Assay yield versus mesitylene.

We then probed the amenability of the α-heteroatom functionality. Excitingly, a formyl-aza-indole derivative served as a competent carbene precursor, undergoing smooth activation and cyclopropanation under optimized conditions (**19**). To the best of our knowledge, this constitutes the first direct catalytic activation of a formamide to generate a reactive carbene intermediate, furnishing densely functionalized cyclopropanes bearing heteroatom-adjacent pharmacophores.

Given the broad steric tolerance observed for aldehydes, we evaluated ketone substrates. To date, Fe–carbene intermediates have not been generated directly from native carbonyl substrates of this class, and their engagement herein represents a noteworthy expansion of Fe–carbene reactivity. Under slightly modified conditions, 4-membered cyclic ketones coupled with styrenes—including 1,1-disubstituted derivatives—to furnish three-dimensional cyclopropanes with synthetically valuable exit vectors (**20**-**23**).

We next examined the impact of electronic variation within the styrene coupling partner on carbene transfer (Figure 3). Using an α-tertiary aldehyde, we observed efficient incorporation across both electron-rich and electron-poor styrenes, with electronic effects exerting only a modest influence on the yield (**24**–**33**). Functional groups offering opportunities for downstream diversification—including Boc-protected amines (**28**), bromides (**30**), esters (**31**), and nitriles (**33**)—were well tolerated, and nitrogen-containing heterocycles, such as indazoles (**34**), coupled smoothly under the reaction conditions. The method also accommodated bicyclic frameworks (**35**). Highlighting the mild nature of the transformation, both *trans* (**36**) and *cis* (**37**) isomers of a cyclobutene derivative reacted with a benzofuran-derived styrene, while preserving their relative configuration. In addition, cyclopropanation of 1,3-diene proceeded selectively at the terminal position (**38**).

**Figure 3:**
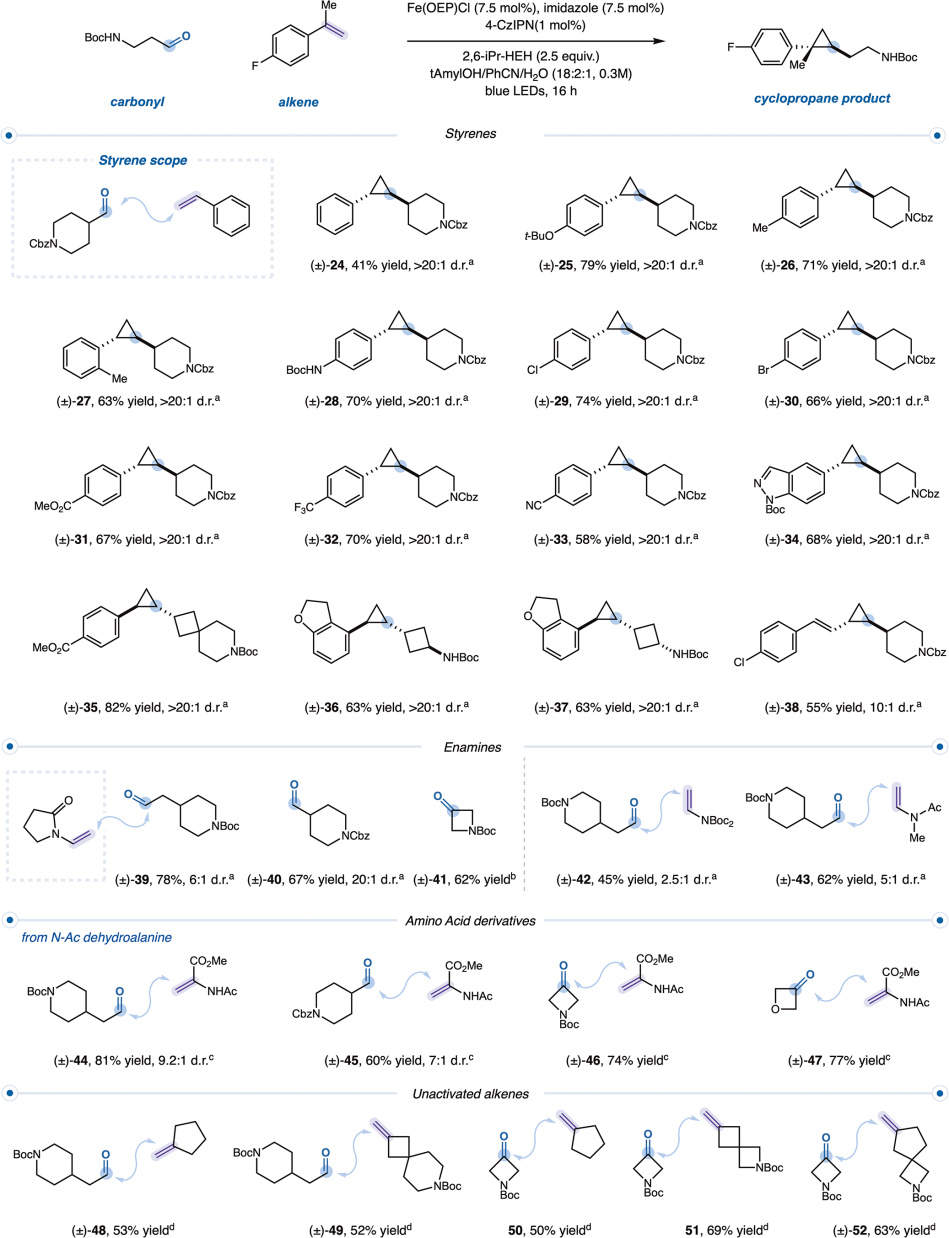
Scope of olefin coupling partners. All isolated yields are reported. See Supporting Information for experimental details. ^a^Performed according to general procedure **A**. ^b^Performed according to general procedure **B**. ^c^Performed according to general procedure **E**. ^d^Performed according to general procedure **C**.

Enamine-type olefins—valuable handles for introducing nitrogen functionality adjacent to the cyclopropane—were well tolerated. A vinyl pyrrolidinone olefin coupled with β-tertiary, α-tertiary, and ketone-derived carbonyls in excellent yields (**39**–**41**). Vinyl di-Boc- and acetate-protected amines were likewise compatible, and their mild deprotection offers convenient access to cyclopropylamines for downstream diversification (**42**–**43**). We were particularly interested in accessing amino-acid derivatives, which offer unique three-dimensionality and new exit vectors for peptide diversification. Reoptimization revealed that pairing a less reducing photocatalyst, 4-ClCzIPN (2,4,5,6-tetrakis­(3,6-dichlorocarbazol-9-yl)­benzene-1,3-dicarbonitrile) (*E*_1/2_(PC/PC^•–^) = −0.97 V vs SCE in MeCN),^42^ with Fe­(TMP)Cl (5,10,15,20-Tetrakis­(4-methoxyphenyl)-21*H*,23*H*-porphine iron­(III) chloride) enabled efficient cyclopropanation of dehydroalanine derivatives (**44**–**47**). Notably, the carbonyl-derived products provide a distinct entry point to highly three-dimensional amino-acid scaffolds. The resulting ketone-derived cyclopropanes provide rigidified noncanonical amino-acid motifs that may modulate binding properties or confer enhanced conformational control when embedded within peptide frameworks.^43,44^

To probe the limits of carbene transfer, we investigated unactivated alkenes, which lack the electronic bias that typically facilitates cyclopropanation. Despite the attenuated reactivity, 1,1-disubstituted alkenes underwent smooth cyclopropanation, enabling incorporation of diverse carbocyclic and saturated heterocyclic scaffolds. Both ketone- and aldehyde-derived carbenes coupled efficiently under optimized conditions, providing a robust cross-coupling platform to highly sp^3^-rich products with broad structural diversity and potential for downstream functionalization (**48**–**52**).

We next explored cyclopropanation in complex settings relevant to medicinal chemistry and peptide building blocks (Figure 4). Azetidinone ketones coupled with complex styrenes—including a fenofibrate derivative (**53**), a tyrosine derivative (**54**), an indazole (**55**), and spirocyclic benzofuran (**56**)—highlight the modular pairing of ketone and olefin partners. Tyrosine-derived styrenes engaged aldehydes efficiently, generating products with unique exit vectors and enabling amino-alcohol cross-couplings (**57**, **58**). Vinyl metaxalone (**59**, **60**) and vinyl tedizolid derivatives (**61**), the latter bearing a free primary alcohol, underwent high-yielding cyclopropanation. Notably, an ezetimibe derivative reacted efficiently under a 1:1 stoichiometry of aldehyde and styrene, underscoring the practicality of the protocol for late-stage cross-coupling of densely functionalized substrates (**62**). More demanding combinations—such as vinyl tedizolid with a thiazole-derived aldehyde (**63**)—also coupled efficiently. Substrates containing tertiary alcohols readily reacted with alaninal derivatives (**64**). A 3-azaspiro[5.5]­undecane-derived aldehyde underwent cross-coupling with a spiro-azetidine–isobenzofuran bearing a terminal alkene, enabling rapid access to highly three-dimensional, structurally complex scaffolds (**65**). Together, these results demonstrate the amenability of the optimized cyclopropanation conditions to diverse electronic and steric environments, complex functional groups, and sensitive motifs.

**Figure 4:**
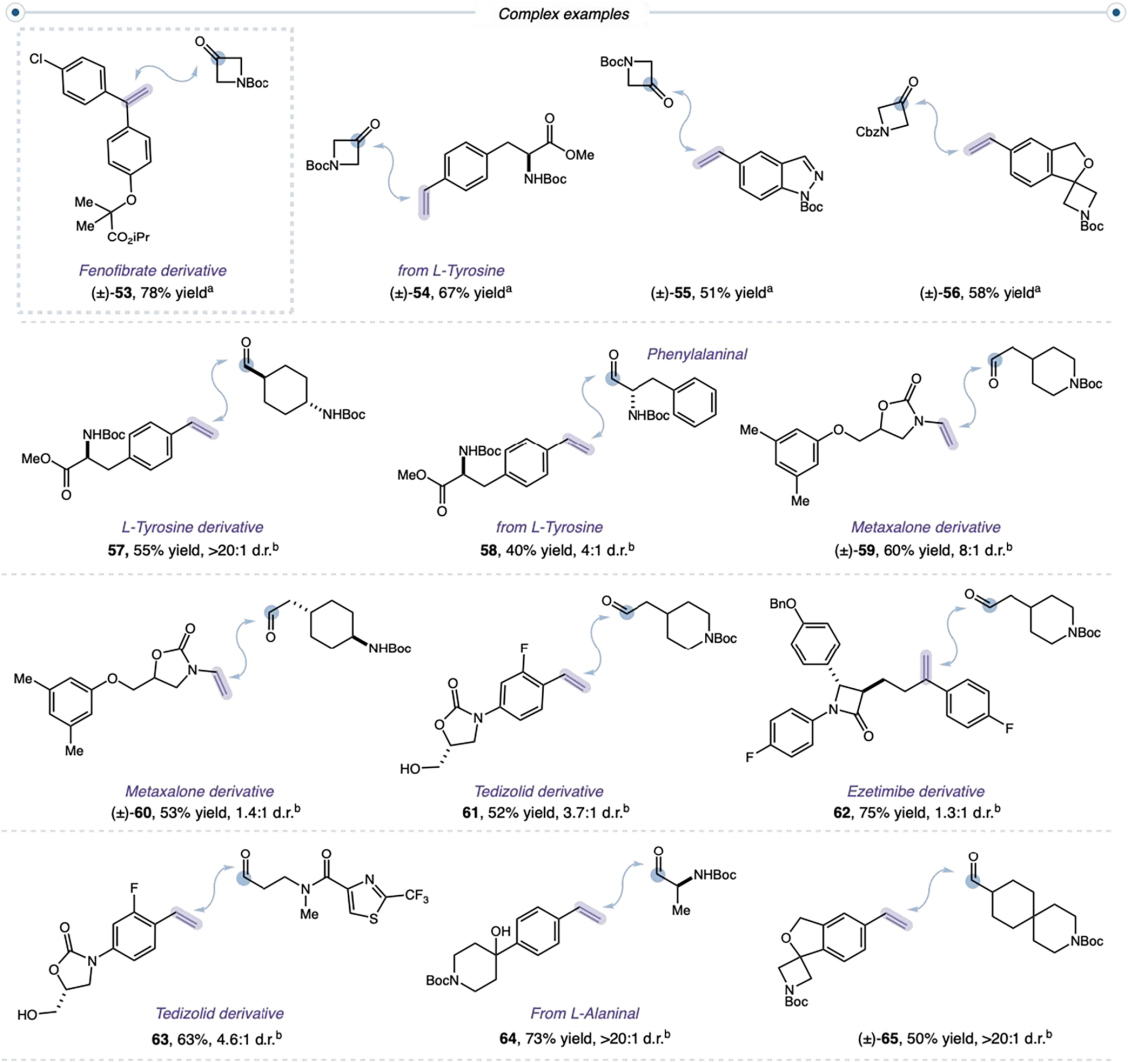
Drug-like olefins and carbene precursors. All isolated yields are reported. See Supporting Information for the experimental details. ^a^Performed according to general procedure **B**. ^b^Performed according to general procedure **A**.

### Mechanistic Studies

The reaction is proposed to proceed through a metal-mediated carbene-formation pathway. First, Fe­(OEP) **I** is reduced to low-valent Fe­(I) (*E*_1/2_ (Fe^II^/Fe^I^) = −1.12 V vs SCE), capable of activating the carbonyl precursor to form an Fe­(III) α-hydroxy–metalated intermediate **II** (Figure 5a). A second reduction event regenerates an Fe­(II) complex **III**, which, upon protonation and subsequent α-elimination, delivers the Fe­(II) carbene species **IV**.^23^ This metallocarbene undergoes olefin addition to furnish the cyclopropane while regenerating Fe­(II) catalyst **I**. Throughout the cycle, the Hantzsch ester is expected to serve as both the sacrificial reductant and a potential proton donor in the α-protonation step.^29^

**Figure 5:**
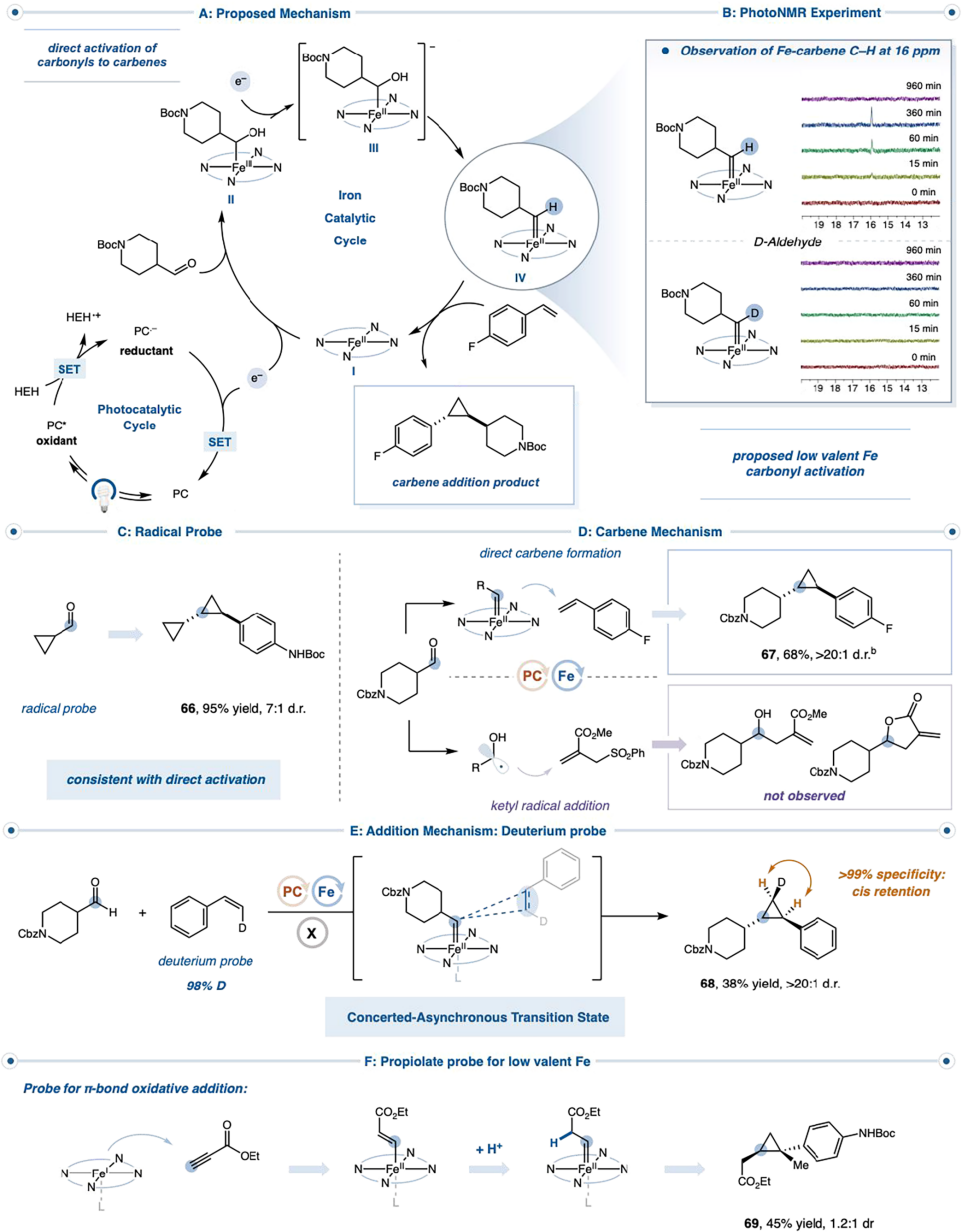
Mechanistic studies on the direct formation of carbenes from carbonyls.^**a** a^Isolated yields are reported. See Supporting Information for experimental details. ^b^Assay yield reported versus 1,4-difluorobenzene

To interrogate this proposal, we evaluated the reaction under in situ PhotoNMR conditions.^28,45,46^ Rapid formation of the cyclopropane product was observed, accompanied by the appearance of a persistent signal at ∼16 ppm, which dissipated at the end of the reaction (Figure 5b). Given that Fe-carbene complexes typically resonate at ∼19 ppm, we hypothesized that this peak could correspond to either the metallocarbene or a reduced iron species.^5,28,46^ To probe this, we subjected the corresponding deuterated aldehyde to identical conditions. Product formation occurred at a comparable rate, but the ∼16 ppm signal was absent, supporting the assignment of this resonance to the carbene and ruling out a reduced Fe species.

Having established the presence of a metallocarbene, we next evaluated the mechanism of carbene formation. Cyclopropycarbaldehyde underwent smooth conversion to **66** without any detectable ring-opening products, consistent with the suppression of free ketyl intermediates (Figure 5c). We reasoned that a free ketyl-radical pathway was unlikely and that iron must be directly involved in carbonyl activation. We further probed ketyl involvement using an allyl-sulfone trap, which is known to outcompete Fe-porphyrins for ketyl-radical capture (Figure 5d).^19^ Under our conditions, cyclopropane **67** was formed in a good yield with no ketyl-derived products. In the absence of the iron catalyst, neither ketyl-type products nor cyclopropane was detected, confirming that iron is essential for generating the reactive intermediate.

We next examined the mechanism of carbene addition to determine whether the transformation proceeds through a concerted or a stepwise pathway. Under reaction conditions, both *E*- and *Z*-deuterostyrenes delivered products with complete stereospecificity, strongly supporting a concerted–asynchronous carbene transfer mechanism (Figure 5e, **68**).^36–41,47^ Such behavior is consistent with reports of stereospecific olefin cyclopropanation from electrophilic metal carbenes. In line with these precedents, the role of imidazole as a trans-ligand—proposed to enhance positive charge accumulation on the carbene carbon—proved critical: addition of imidazole increased the yield, presumably by accelerating the carbene–olefin coupling step.

To further assess the capacity of low-valent Fe to mediate carbene formation, we turned our attention to propiolates as alternative precursors. Propiolates are known to undergo vinylmetal formation through low-valent metal oxidative addition into the π-system, and we reasoned that our Fe system might engage them analogously, with subsequent α-protonation furnishing the corresponding carbene (Figure 5f).^48^ Indeed, with minor adjustments to the reaction conditions, propiolates were smoothly converted to reactive carbenes en route to cyclopropyl adducts **69**. Taken together, these studies support a unified mechanistic picture in which low-valent iron directly engages carbonyl and other electrophilic π-systems to furnish α-metalated intermediates that undergo protonation and α-elimination to generate metallocarbenes, which in turn react with olefins through a concerted asynchronous carbene transfer pathway.

### Electrochemical Platform

Given the broad scope and utility of the photochemical variant, we reasoned that an electrochemical platform capable of accessing the same redox manifold could enhance the scalability while providing complementary mechanistic insight (Figure 6). Under constant-current electrolysis in an undivided cell, desired product **70** was obtained in 55% yield. Under these conditions, the catalytic system efficiently delivers the low-valent Fe species, which mediates carbene formation and transfer. The use of B_2_cat_2_ as a redox mediator enabled efficient generation of this species, and imidazole remained essential for promoting carbene transfer from the metallocarbene intermediate to the olefin.^49^ To probe the possibility of direct carbonyl reduction, competition experiments were performed. Notably, no ketyl-radical addition products were observed (Supporting Information). Moreover, in the case of sterically congested aldehydes, no ketylstyrene coupling products were detected, suggesting that direct carbonyl reduction is disfavored and supporting a mechanism in which both the electrochemical and photochemical variants activate carbonyl precursors through low-valent Fe to access a common metallocarbene intermediate **71**. Finally, the electrochemical protocol demonstrated practical utility, enabling the gram-scale preparation of **72**.

**Figure 6:**
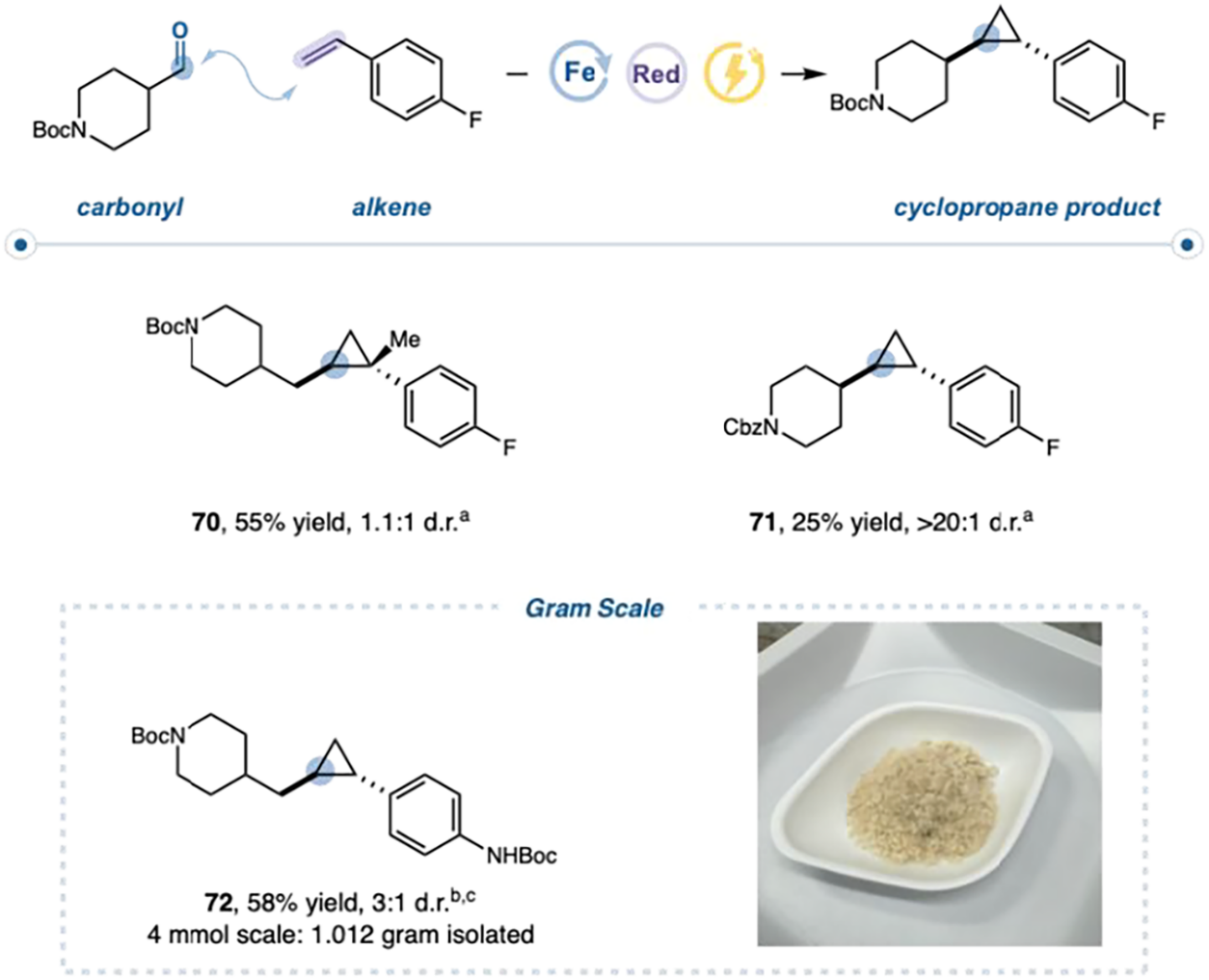
Electrochemical setup. RVC­(+)/RVC(−) undivided cell, I = 5 mA, C = 3.0 F/mol, styrene (1.0 equiv, 0.5 mmol), iPr-HEH (1.0 equiv, 0.5 mmol), aldehyde (2.0 equiv, 1.0 mmol), imidazole (0.1 equiv, 0.05 mmol), Fe­(OEP)Cl (0.1 equiv, 0.05 mmol), B_2_cat_2_ (0.2, 0.1 mmol), KPF_6_ (0.2 M), water (10 μL, 1 equiv, 5.0 mmol), boric acid (0.20 equiv, 0.10 mmol), and DMA (1.6 mL, 0.3 M). ^a^Assay yield determined by ^19^F NMR versus 1,4-difluorobenzene internal standard. ^b^I = 35 mA, C = 3.0 F/mol. ^c^Isolated yield.

## Conclusions

Collectively, these findings establish a direct, low-valent Fe strategy for generating metallocarbenes from native carbonyls and set the stage for redefining carbonyl activation as a platform for constructing complex three-dimensional molecular architectures. We anticipate that underexplored modes of metal-mediated activation will offer general, mild pathways to metallocarbene intermediates. By enabling modular carbene generation from simple functional groups, this platform opens new regions of the chemical space and provides a versatile foundation for diverse synthetic applications.

## Supplementary Material



## Notes

Additional experimental details; optimization studies; reaction scope; characterization; and spectra (PDF)
