# Supplementary material for: Direct Access to Iron Carbenes from Aldehyde, Ketone, and Formamide Feedstocks
Source: J Am Chem Soc. 2026 Apr 28;148(18):18703–14. doi: 10.1021/jacs.5c21614 (PMC13184977; doi:10.1021/jacs.5c21614)
Supplement: Supplementary file 1 [file ja5c21614_si_001.pdf]

Supplementary Information

Direct Access to Iron Carbenes from Aldehyde, Ketone, and Formamide Feedstocks

P. Scott Pedersen,[1,#] Katherine I. Burton,[1,#] Sven H. M. Kaster,[1,#] Eva Lin,[1,†] Andria L. Pace,[1,†] Marian C. Bryan,[2] Taylor M. Sodano,[2] Nicholas E. Intermaggio,[2] Christopher B. Kelly,[3] and David W. C. MacMillan[1,*]

[1]Merck Center for Catalysis at Princeton University, Princeton, New Jersey 08544, USA. [2]Global Discovery Chemistry, Johnson & Johnson Innovative Medicine, Spring House, PA, 19477, USA. [3]Discovery Process Research, Johnson & Johnson Innovative Medicine, Spring House, PA, 19477, USA. [#]These authors contributed equally. [†]These authors contributed equally. *Corresponding author.

**Corresponding author. Email: dmacmill@princeton.edu*

Table of Contents

1. General Information S3
2. Reaction Optimization S5
3. General Procedure for Substrate Synthesis S10
4. General Reaction Procedures S15
5. Experimental and Characterization Data S19
6. Mechanism Data S88
7. Electrochemistry Optimization and Characterization S113
8. Effect of π-nucleophilicity on yield of reaction S121
9. References S123
10. Spectral Data S124

1. General Information

Commercial reagents were used without further purification unless otherwise indicated. All photocatalysts investigated are commercially available but are also available from literature reported procedures. Organic solutions were concentrated under reduced pressure using a Büchi rotary evaporator with a water bath; this is interchangeably referred to as "evaporated in vacuo" in the text and supplementary information. Chromatographic purification of compounds was executed using an automated Combi-flash NextGen 300^+ system or an automated Biotage Isolera Four system using RediSep Rf Gold silica gel columns (20 to 40 microns). Where specified, reverse phase chromatography was performed on a Teledyne ISCO ACCQPrep HP150 system using RediSep® Prep HPLC Columns (30 mm x 250 mm C18, 100 Å) with 0.1% formic acid buffered water and acetonitrile solutions. Reverse phase chromatography was performed on the same PrepHPLC system using a Waters XBridge BEH C18 OBD Prep Column (30 mm x 150 mm, 130 Å, 5 µm) with 0.1% ammonium hydroxide buffered water and acetonitrile solutions. Thin-layer chromatography (TLC) was performed using Analtech Uniplate 0.25 mm or Supelco 0.20 mm silica gel F-254 plates. Visualization of the developed chromatography was performed using a UV lamp, or staining with $KMnO_4$. Preparative thin-layer chromatography was performed on Analtech 1 mm or Supelco 0.50 mm silica gel F-254 plates. ^{1}H NMR spectra were recorded at 400 MHz or 500 MHz, using a BRUKER NanoBay Avance III HD 400 or BRUKER Avance III NMR spectrometer, respectively. ^{13}C NMR spectra were recorded at 101 MHz or 126 MHz on a BRUKER NanoBay Avance III HD 400 or BRUKER Avance III NMR spectrometer, respectively. Chemical shifts of ^{1}H NMR and ^{13}C NMR spectra (measured at 298 K) are given in ppm by using residual solvent signals as references ($CDCl_3$: 7.26 ppm and 77.16 ppm, respectively; DMSO-*d6*: 2.50 ppm and 39.52 ppm, respectively; MeOD: 3.31 ppm and 49.00 ppm, respectively; MeCN-*d3*: 1.94 ppm and 118.26 ppm, respectively; D_2O: 4.79 ppm).[4] ^{19}F NMR spectra were recorded on a BRUKER NanoBay Avance III HD 300 or BRUKER NanoBay Avance III HD 300, and are reported unreferenced. Coupling constants (J) are reported in Hertz (Hz). Standard abbreviations indicating multiplicity were used as follows: s (singlet), d (doublet), t (triplet), q (quartet), p (pentet), s (sextet), h (septet), m (multiplet), b (broad). Apparent multiplets arising from overlapping signals are marked as virtual multiplets (*virt*). Data for ^{13}C NMR are reported in terms of chemical shifts; multiplicity and coupling constants are included when coupling with ^{19}F nuclei.

Liquid chromatography (LC) analysis was performed on an Agilent 1200 Infinity or 1290 Infinity II LC system. IR spectra were recorded on a Perkin Elmer Spectrum 100 FTIR spectrometer and are reported in wavenumbers (cm^{-1}). High Resolution Mass Spectra (HRMS) were obtained from the Princeton University Mass Spectral Facility.

2. Reaction Optimization

Table S1.

Evaluation of solvent effect for unactivated alkenes.

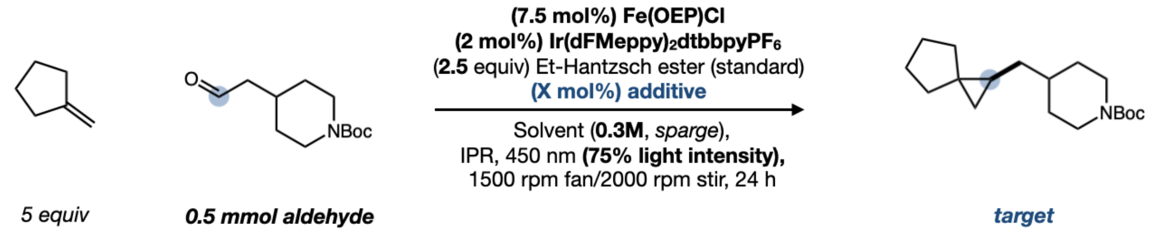

	Solvent	*Additive:*	Outcome:
(No additive Controls)	DMA	*NONE replicate 1*	***6% target yield***
	DMA	*NONE replicate 2*	***4% target yield***
	DMA	***7.5 mol% imidazole***	***15% target yield***
	(18:2:1) TAA:PhCN:H_2O	***7.5 mol% imidazole***	***53% target yield***
	DMA	***7.5 mol% NBocHisOH***	***21% target yield***

Table S2. Evaluation of catalyst conditions for ketone optimization

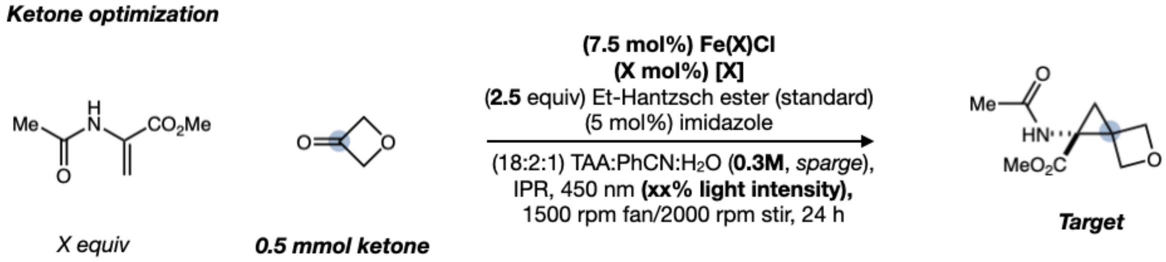

7.5 mol% [Fe]	X mol% [Photocat]	X equiv alkene		Outcome:
Fe(OEP)Cl	2 mol% 4CzIPN	2.00	75% light	29% yield
Fe(OEP)Cl	2 mol% 4ClCzIPN	2.00	75% light	40% yield
Fe(PMP)Cl	2 mol% 4ClCzIPN	2.00	75% light	44% yield
Fe(OEP)Cl	2 mol% 4CzIPN	3.50	75% light	41% yield
Fe(PMP)Cl	2 mol% 4ClCzIPN	3.50	75% light	77% yield*
Fe(PMP)Cl	5 mol% 4ClCzIPN	3.50	75% light	67% yield**

Table S3. Evaluation of alcohol solvents. Detection of undesired acetal byproduct.

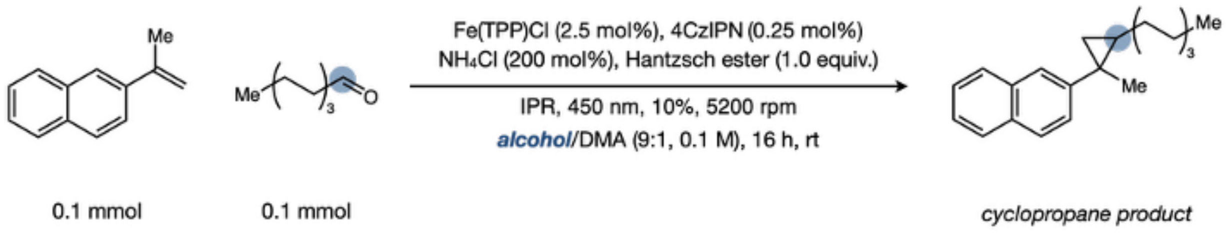

Alcohol solvent	yield	styrene	aldehyde	acetal
MeOH	30%	20%	13%	30%
EtOH	n.d.	98%	14%	86%
iPrOH	1%	n.d.	14%	76%
tAmylOH	14%	22%	59%	n.d.

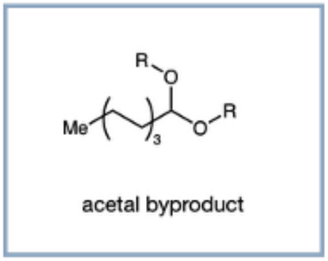

Table S4. Observation of Hantzsch ester undesired formaldehyde adduct formation

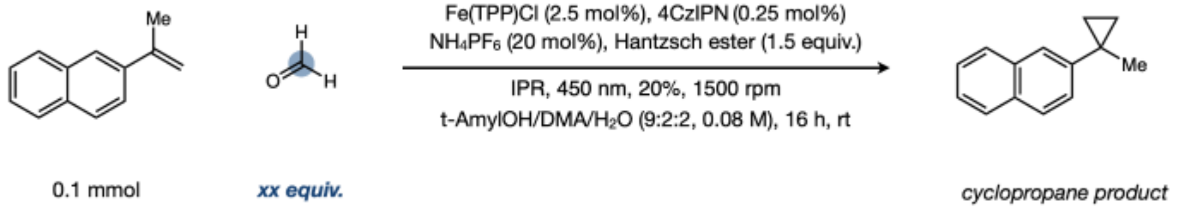

Formaldehyde loading	yield	styrene	formaldehyde
3.0 equiv.	28%	43%	33%
5.0 equiv.	21%	51%	81%

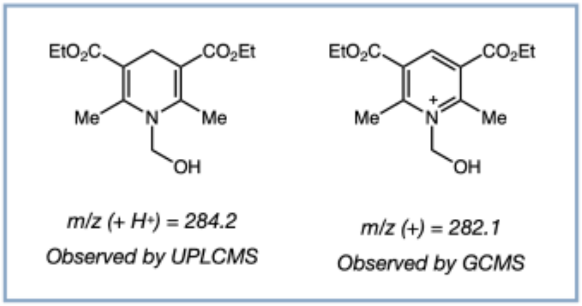

Table S5. Evaluation of imidazole type apical ligands

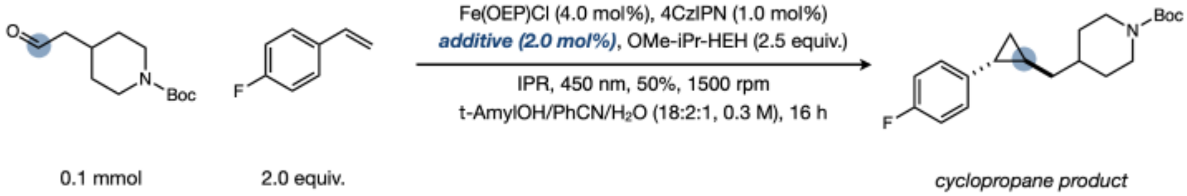

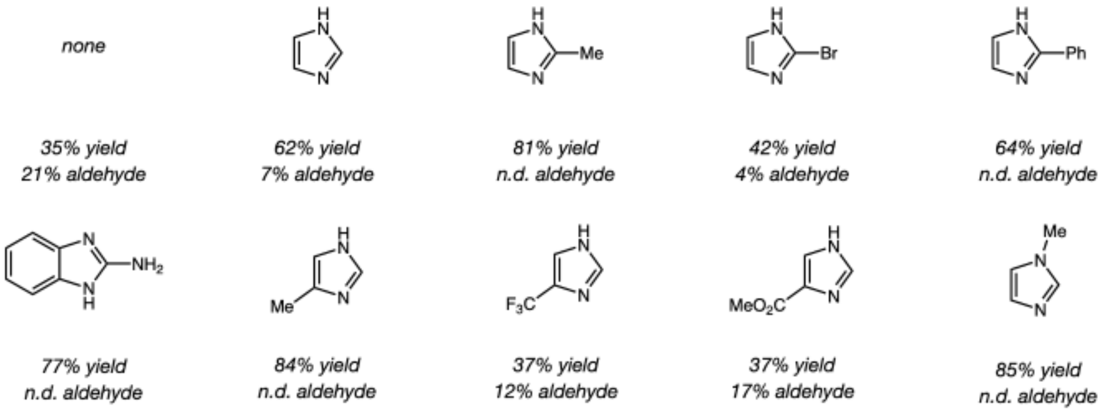

Table S6. Evaluation of additional non-imidazole type apical ligands

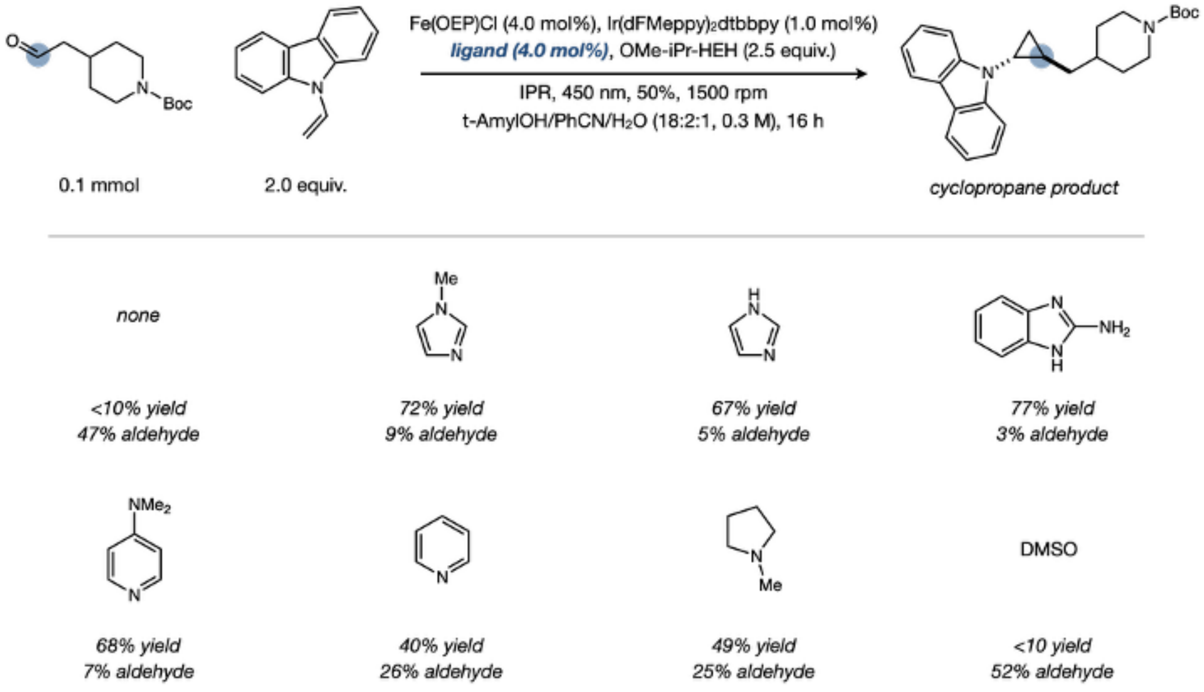

Table S7. Evaluation of other solvents

Solvent + imidazole effect

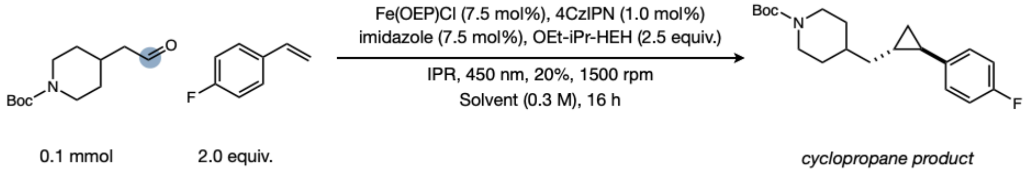

	TAA:PhCN:H2O	*THF*	*DMF*	*DCM*	*ACN*
Imidazole	*98% AY, 3:1 dr*<br>*trace RSM AI.*	*0% AY*<br>*>95% RSM AI.*	*95% AY, 4:1 dr*<br>*0% RSM AI.*	*6% AY, 4:1 dr*<br>*81% RSM AI.*	*22% AY, 4:1 dr*<br>*28% RSM AI.*

Yield determined by NMR assay vs 1,4 difluorobenzene

Table S8. Evaluation of other solvents with Branched aldehyde

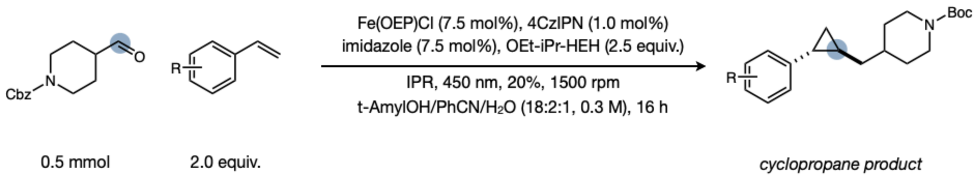

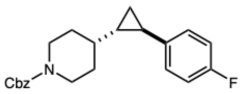

Standard Cond.
85% AY
76% IY

THF	Acetonitrile	DMF
0% AY	45% AY	75% AY

Yield determined by 19F NMR vs 1,4 Difluorobenzene

3. General Procedure for Substrate Synthesis

i. Synthesis of Hantzsch Ester HEH-1

Preparation of Standard Hantzsch Ester (S1) diethyl 2,6-diisopropyl-1,4-dihydropyridine-3,5-dicarboxylate (HEH-1)

The title compound was prepared according to a modified literature procedure.[1]

To an oven-dried 500 mL round-bottom flask equipped with magnetic stir bar was added paraformaldehyde (5 g, 167 mmol), ammonium acetate (19.3 g, 250 mmol, 1.5 equiv), and ethyl 4-methyl-3-oxo-pentanoate (53.0 g, 333 mmol, 2.0 equiv). The vessel was then evacuated and backfilled with nitrogen three times. The mixture was stirred at 120°C for 12 hours. The reaction solution was then filtered over a pad of celite and subsequently subjected to silica gel column chromatography (70:30 Hexanes/EtOAc), followed by recrystallization in cold pentanes (200 mL).

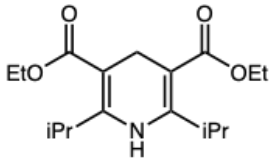

^{1}H NMR (500 MHz, $CDCl_3$) δ 5.74 (s, 1H), 4.16 (q, J = 7.1 Hz, 4H), 4.10 (p, J = 7.0 Hz, 2H), 3.26 (s, 2H), 1.28 (t, J = 7.1 Hz, 6H), 1.13 (d, J = 7.0 Hz, 12H).

ii. Synthesis of Styrenes

General protocol (S2)

General procedure for styrene synthesis was adapted from literature precedent.[2]

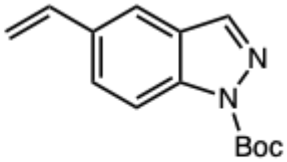

***tert*-butyl 6-vinyl-1*H*-indazole-1-carboxylate**

Palladium chloride (23.87 mg, 134.6 µmol, 0.02 equiv.), triphenylphosphine (105.9 mg, 403.8 mg, 0.06 equiv.), potassium vinyltrifluoroborate (1.08 g, 8.08 mmol, 1.2 equiv.), tert-butyl 5-bromo-1H-indazole-1-carboxylate (2.0 g, 6.73 mmol, 1.0 equiv.), and cesium carbonate (6.6 g, 20.2 mmol, 3.0 equiv.) were weighed out into a 40 ml vial. This vial was capped and then 20 ml of THF and 2.0 mL DI water were added. This reaction was then heated for 24 hours at 80ºC. The reaction was cooled and extracted with ethyl acetate (25 ml) washing with deionized water (100 ml). This crude was subject to column chromatography (0-20% EA/Hx) giving the product as a white solid in 33.2% yield (545 mg, 2.23 mmol).

^{1}H NMR (500 MHz, Chloroform-*d*) δ 8.15 (d, *J* = 0.8 Hz, 1H), 8.13 (d, *J* = 8.8 Hz, 1H), 7.72 – 7.68 (m, 1H), 7.65 (dd, *J* = 8.7, 1.6 Hz, 1H), 6.83 (dd, *J* = 17.6, 10.9 Hz, 1H), 5.79 (dd, *J* = 17.5, 0.8 Hz, 1H), 5.30 (dd, *J* = 10.9, 0.7 Hz, 1H), 1.73 (s, 9H).

^{13}C NMR (126 MHz, Chloroform-*d*) δ 149.29, 139.85, 136.38, 133.75, 127.20, 126.42, 118.92, 114.76, 114.17, 85.11, 28.34.

HRMS *m*/*z* calcd. For $C_{14}H_{16}N_2O_2Na^+$ ($[M+Na]_+$) 267.1104, found 267.1108.

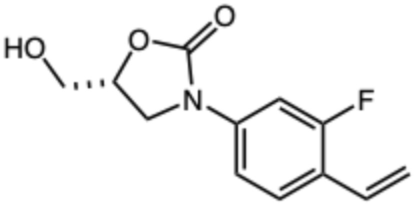

(*R*)-3-(3-fluoro-4-vinylphenyl)-5-(hydroxymethyl)oxazolidin-2-one

Palladium chloride (24.5 mg, 137.9 µmol, 0.02 equiv.), triphenylphosphine (108.5 mg, 413.7 mg, 0.06 equiv.), potassium vinyltrifluoroborate (1.11 g, 8.27 mmol, 1.2 equiv.),(R)-3-(4-bromo-3-fluorophenyl)-5-(hydroxymethyl)oxazolidin-2-one (2.0 g, 6.89 mmol, 1.0 equiv.), and cesium carbonate (6.74 g, 20.7 mmol, 3.0 equiv.) were weighed out into a 40 ml vial. This vial was capped and then 20 ml of THF and 2.0 mL DI water were added. This reaction was then heated for 24 hours at 80ºC. The reaction was cooled and extracted with ethyl acetate (25 ml) washing with deionized water (100 ml). This crude was subject to column chromatography (0-20% EA/Hx) giving the product as a white solid in 55.0% yield (900 mg, 3.79 mmol).

Some starting material impurity.

^{1}H NMR (500 MHz, Acetone-d_6) δ 7.57 (t, *J* = 8.7 Hz, 1H), 7.55 (dd, *J* = 13.6, 2.1 Hz, 1H), 7.31 (dd, *J* = 8.6, 2.4 Hz, 1H), 6.78 (dd, *J* = 17.8, 11.3 Hz, 1H), 5.79 (dd, *J* = 17.8, 1.2 Hz, 1H), 5.29 (dd, *J* = 11.3, 1.2 Hz, 1H), 4.76 (ddt, *J* = 9.8, 6.3, 3.7 Hz, 1H), 4.35 (t, *J* = 5.9 Hz, 1H), 4.15 (t, *J* = 9.0 Hz, 1H), 3.96 (dd, *J* = 8.8, 6.3 Hz, 1H), 3.85 (ddd, *J* = 12.3, 5.6, 3.4 Hz, 1H), 3.73 (ddd, *J* = 12.4, 6.2, 4.0 Hz, 1H).
^{13}C NMR (126 MHz, Acetone) δ 161.05 (d, *J* = 246.2 Hz), 155.24, 141.02 (d, *J* = 11.4 Hz), 129.49 (d, *J* = 3.4 Hz), 128.39 (d, *J* = 5.3 Hz), 120.63 (d, *J* = 12.6 Hz), 115.86 (d, *J* = 4.7 Hz), 114.13 (d, *J* = 3.1 Hz), 105.74 (d, *J* = 28.4 Hz), 74.30, 63.16, 46.95.
^{19}F NMR (471 MHz, Acetone) δ -118.63 (dd, *J* = 13.7, 8.8 Hz, 1F).

HRMS *m/z* calcd. For $C_{12}H_{12}FNO_3Na^+$ ($[M+Na]^+$) 238.0874, found 238.0871.

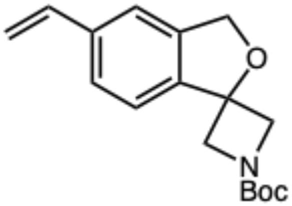

***tert*-butyl 5'-vinyl-3'*H*-spiro[azetidine-3,1'-isobenzofuran]-1-carboxylate**
Palladium chloride (26.06 mg, 146.97 µmol, 0.02 equiv.), triphenylphosphine (115.64 mg, 115.64 mg, 0.06 equiv.), potassium vinyltrifluoroborate (1.18 g, 8.82 mmol, 1.2 equiv.), tert-Butyl 5'-bromo-3'H-spiro[azetidine-3,1'-isobenzofuran]-1-carboxylate (2.5 g, 7.35 mmol, 1.0 equiv.), and Cesium carbonate (7.18 g, 22.05 mmol, 3.0 equiv.) were weighed out into a 40 ml vial. This vial was capped and then 25 ml of THF and 2.5 ml DI water were added. This reaction was then heated for 24 hours at 80ºC. The reaction was cooled and extracted with ethyl acetate (25 ml) washing with deionized water (100 ml). This crude was subject to column chromatography (0-20% EA/Hx) giving the product as a white solid in 85% yield (1.8 g, 6.26 mmol).

^{1}H NMR (500 MHz, Chloroform-*d*) δ 7.41 (s, 1H), 7.41 (s, 1H), 7.25 (s, 1H), 6.74 (dd, *J* = 17.6, 10.9 Hz, 1H), 5.76 (dd, *J* = 17.6, 0.8 Hz, 1H), 5.28 (dd, *J* = 10.8, 0.8 Hz, 1H), 5.10 (d, *J* = 1.0 Hz, 2H), 4.31 (dd, *J* = 9.3, 1.2 Hz, 2H), 4.13 (dd, *J* = 9.3, 1.2 Hz, 2H), 1.48 (s, 9H).

^{13}C NMR (126 MHz, Chloroform-*d*) δ 156.63, 141.34, 139.32, 138.37, 136.39, 126.82, 120.93, 118.33, 114.73, 82.67, 79.97, 72.79, 28.55.
HRMS *m/z* calcd. For $C_{17}H_{21}NO_3Na^+$ ($[M+Na]^+$) 310.1413, found 310.1415.

iii. Synthesis of Formamide

Protocol adapted from literature precedent.[3]
To a flame-dried 3-neck 250 mL round bottom flask was added 1H-pyrrolo[2,3-b]pyridine (2.00 g, 16.9 mmol, 1.00 equiv) and freshly distilled, anhydrous THF (15 mL). Under ice bath cooling, EtMgBr (5.6 mL, 16.9 mmol, 1.00 equiv, as 3.0M stock solution in ether) was added dropwise over 20 minutes. The resulting mixture was permitted to stir at room temperature for 0.5 h, to generate a white solution. To this solution was added ice-cold ethyl formate (4.1 mL, 51.00 mmol, 3 equiv). *Nota bene:* the protocol performs best when using fresh, chilled (between -5°C to 0 °C) ethyl formate. The resulting yellow solution was permitted to slowly warm to room temperature over 12 hours. The resulting white crude was passed over a short silica gel plug, with hexanes/ethyl acetate (250 mL, 1:1, v/v) eluent. The resulting crude was purified via 25 g Biotage Sf.r standard phase column and purified with eluents (0-10%) ethyl acetate in hexanes to furnish a white solid.

Nota bene: heating the reaction protocol induces formation of undesired regioisomeric formylation byproducts.

iv. Synthesis of 4ClCzIPN

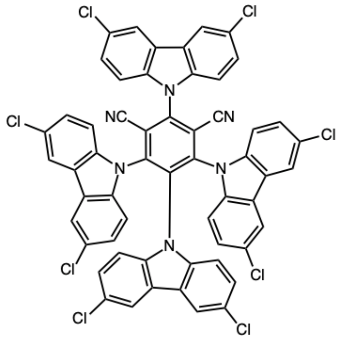

2,4,5,6-tetrakis(3,6-dichlorocarbazol-9-yl)benzene-1,3-dicarbonitrile

Protocol adapted from literature precedent.[4]

A flame-dried 250 mL round bottom flask was charged with 3,6-dichloro-9H-carbazole (2.36 g, 10.0 mmol, 5.00 equiv), 2,4,5,6-tetrafluoroisophthalonitrile (0.4 g, 2.00 mmol), and anhydrous THF (25 mL). Under ice bath conditions, NaH (0.4 g, 10 mmol, 5.00 equiv, 60% in mineral oil) was added. The resulting mixture was permitted to stir at room temperature for 12 h, then quenched under ice bath conditions. The resulting crude was purified via silica gel chromatography (0-10% hexanes/ethyl acetate) to furnish a yellow solid (1.96 g, 1.84 mmol, 92% yield).

4) General Procedures for Carbene Reactivity

Note: *In all cases, unless otherwise specified, Hantzsch ester refers to compound* ***HEH-1*** *(See synthesis section for further details).*

Note: *In all cases, if the carbonyl component is a liquid, it is added immediately prior to sparging.*

Note: *For monosubstituted styrenes: 4CzIPN is the optimal photocatalyst to avoid competitive triplet sensitization pathways.*

General Procedure A – Aldehyde to Carbene Conversion: Cyclopropanation of Styrenes and activated alkenes

<u>0.5 mmol scale:</u> To a 40-mL vial equipped with magnetic stir bar was added Ir(dFMeppy)$_2$(dtbbpy)PF$_6$ (1 mol%) if 1,1 disubstituted alkene, 4-CzIPN (1 mol%) if a monosubstituted styrene, Fe(OEP)Cl (7.5 mol%), Hantzsch ester (2.5 equiv), styrene (2.0 equiv), imidazole (7.5 mol%), aldehyde (0.5 mmol). To this the standard solvent solution tAmylOH/PhCN/H_2O (18:2:1 volumetric ratio) (0.3M) is added. The capped vial is sealed with electrical tape, and the reaction solution was then sparged for 5 minutes under nitrogen atmosphere. Immediately following sparging, the vial cap was sealed with parafilm and placed in an M1 Penn integrated photoreactor and irradiated for 18 hours at 450 nm (20% light intensity, M2 generation plates, 2000 rpm stir rate, fans 1500 rpm). Immediately following irradiation, the reaction crude was evaporated in vacuo and the resulting residue was directly subjected to separation protocol using flash column chromatography (SiO_2) to furnish the cyclopropanated product.

General Procedure B – Ketone and Formamide to carbene reactions

<u>0.5 mmol scale:</u> To a 40-mL vial equipped with magnetic stir bar was added Ir(dFMeppy)$_2$(dtbbpy)PF$_6$ (2 mol%), Fe(OEP)Cl (7.5 mol%), Hantzsch ester (2.5 equiv), alkene/styrene (2.0 equiv), imidazole (7.5 mol%), ketone/formamide (0.5 mmol). To this the standard solvent solution tAmylOH/PhCN/H_2O (18:2:1 volumetric ratio) (0.3M) is added. The capped vial is sealed with electrical tape, and the reaction solution was then sparged for 5 minutes under nitrogen atmosphere. Immediately following sparging, the vial cap was sealed with parafilm and placed in an M1 Penn integrated photoreactor and irradiated for 24 hours at 450 nm (75% light intensity, M1 generation plates, 2000 rpm stir rate, fans 1500 rpm). Immediately following irradiation, the reaction crude was evaporated in vacuo, and the resulting residue was directly subjected to separation protocol using flash column chromatography (SiO_2) or reverse phase chromatography to furnish the cyclopropanated product.

<u>Nota bene: 0.1 mmol scale:</u> Similar yields with ketone precursor can be afforded on small scale by using Fe(TPP)Cl (15 mol%), and 4ClCzIPN (2 mol%), and commercial diludine (2.5 equiv); 24 hours at 450 nm (100% light intensity, M1 generation plates, 2000 rpm stir rate, fans 1500 rpm).

General Procedure C – Cyclopropanation of unactivated alkene coupling partners

0.5 mmol scale: To a 40-mL vial equipped with magnetic stir bar was added Ir(dFMeppy)$_2$(dtbbpy)PF$_6$ (2 mol%), Fe(OEP)Cl (7.5 mol%), Hantzsch ester (2.5 equiv), alkene (1 mmol, 2 equiv), N-Boc-His-OH (5 mol%), and aldehyde (0.5 mmol). To this the standard solvent solution tAmylOH/PhCN/H_2O (18:2:1 volumetric ratio) (0.3M) is added. The capped vial is sealed with electrical tape, and the reaction solution was then sparged for 5 minutes under nitrogen atmosphere. Immediately following sparging, the vial cap was sealed with parafilm and placed in an M1 Penn integrated photoreactor and irradiated for 24 hours at 450 nm (50% light intensity, M1 generation plates, 2000 rpm stir rate, fans 1500 rpm). Immediately following irradiation, the reaction crude was evaporated in vacuo, and the resulting residue was directly subjected to separation protocol using flash column chromatography (SiO_2) to furnish the cyclopropanated product. Note, higher alkene loadings are tolerated.

General Procedure D – Formaldehyde as direct carbene precursors with unactivated alkenes

0.5 mmol scale for styrene substrates: To a 40-mL vial equipped with magnetic stir bar was added Ir(dFMeppy)$_2$(dtbbpy)PF$_6$ (2 mol%), Fe(OEP)Cl (4 mol%), Hantzsch ester (2.5 equiv), alkene (0.5 mmol, limiting), imidazole (5 mol%), and formaldehyde (10 equiv, as commercial 37% by weight stock solution in water). To this the standard solvent solution tAmylOH/PhCN/H_2O (18:2:1 volumetric ratio) (0.3M) is added. The capped vial is sealed with electrical tape, and the reaction solution was then sparged for 5 minutes under nitrogen atmosphere. Immediately following sparging, the vial cap was sealed with parafilm and placed in an M1 Penn integrated photoreactor and irradiated for 24 hours at 450 nm (50% light intensity, M1 generation plates, 2000 rpm stir rate, fans 1500 rpm). Immediately following irradiation, the reaction crude was evaporated in vacuo, and the resulting residue was directly subjected to separation protocol using flash column chromatography (SiO_2) to furnish the cyclopropanated product.

0.1 mmol scale for non-styrene substrates: To an 8-mL vial equipped with magnetic stir bar was added by (8:2 DCM:MeCN v/v) stock solution: 4ClCzIPN (2 mol%), Fe(TPP)Cl (15 mol%), and imidazole (5 mol%). Next, the solvent was evaporated in vacuo to afford dry material. To this residue was added: Hantzsch ester (2.5 equiv), alkene (0.1 mmol, limiting), the standard solvent solution tAmylOH/PhCN/H_2O (18:2:1 volumetric ratio) (0.3M) and formaldehyde (10 equiv, as commercial 37% by weight stock solution in water). The capped vial was sealed with electrical tape, and the reaction solution was then sparged for 3 minutes under nitrogen atmosphere. Immediately following sparging, the vial cap was sealed with parafilm and placed in an M1 Penn integrated photoreactor and irradiated for 24 hours at 450 nm (50% light intensity, M1 generation plates, 2000 rpm stir rate, fans 1500 rpm). Immediately following irradiation, the reaction crude was evaporated in vacuo, and the resulting residue was directly subjected to separation protocol using flash column chromatography (SiO_2) to furnish the cyclopropanated product.

General Procedure E – Noncanonical amino acids as alkene coupling partners

0.5 mmol scale: To a 40-mL vial equipped with magnetic stir bar was added 4ClCzIPN (2 mol%), Fe(PMP)Cl (7.5 mol%), Hantzsch ester (2.5 equiv), noncanonical amino acid alkene (2.0 equiv), imidazole (7.5 mol%), aldehyde/ketone (0.5 mmol). To this the standard solvent solution tAmylOH/PhCN/H_2O (18:2:1 volumetric ratio) (0.3M) is added. The capped vial is sealed with electrical tape, and the reaction solution was then sparged for 5 minutes under nitrogen atmosphere. Immediately following sparging, the vial cap was sealed with parafilm and placed in an M1 Penn integrated photoreactor and irradiated for 24 hours at 450 nm (75% light intensity, M1 generation plates, 2000 rpm stir rate, fans 1500 rpm). Immediately following irradiation, the reaction crude was evaporated in vacuo and the resulting residue was directly subjected to separation protocol using flash column chromatography (SiO_2) to furnish the cyclopropanated product.

General Procedure F – Electrochemical setup
An oven dried ElectraSyn vial (5.0 mL) with a magnetic stir bar was charged with styrene (1.0 equiv, 0.5 mmol), iPr-HEH (1.0 equiv, 0.5 mmol), aldehyde (2.0 equiv, 1.0 mmol), imidazole (0.1 equiv, 0.05 mmol), Fe(OEP)Cl (0.1 equiv, 0.05 mmol), B_2cat_2 (0.2, 0.1 mmol), KPF_6 (0.2 M), water (10 μL, 1 equiv, 0.5 mmol), boric acid (0.20 equiv, 0.10 mmol), and DMA (1.6 mL, 0.3 M). If the aldehyde or styrene are liquid, it is added after the solvent. The electrasyn vial cap, equipped with RVC foam connected to the vial cap by Cu wire (see below for images of set up), was inserted into the vial containing the reaction mixture. (*Note: ElectraSyn RVC holders sold commercially can also be used for comparable yields*). The vial was allowed to stir for 2 minutes under nitrogen atmosphere. The reaction was carried out under nitrogen atmosphere at room temperature. The vial was connected to the ElectraSyn, and the ElectraSyn was set up as follows: New exp. > Constant current > 5 mA > No ref. electrode > Total charge > 0.5 mmol, 3.0 F/mol > No alternating polarity > Start.

After electrolysis, the electrodes were rinsed with ethyl acetate into the vial, and the solvent was removed by rotary evaporation. The crude material was redissolved in acetonitrile, then filtered through a 10 Micron Polyethylene Frit or a plug of celite. The filtrate was concentrated by rotary evaporation and then purified by automated flash chromatography followed by preparative HPLC.

Graphical guide for DIY RVC electrode set up:
Note: Commercial ElectraSyn RVC holders (not pictured) can also be used for comparable yields.

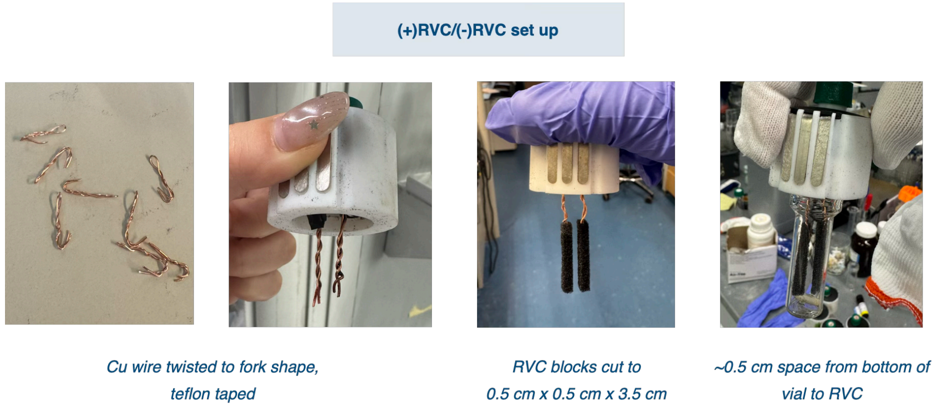

Figure S1. Electrochemical set up

Left to right: Copper wire twisted into fork shape, and then taped onto the ElectraSyn vial cap connections with Teflon tape. Tweezers are helpful for this task. Then, RVC blocks cut from foam panels purchased from ERG aerospace (Duocel RVC foam panel, 100 ppi) were inserted onto the holders. A clean, oven dried 5 mL ElectraSyn vial was then equipped with the electrode, and the electrode can be cut if needed to insure the same amount of spacing from the bottom of the vial to the electrode (~0.5 cm of space).

Reticulated vitreous carbon (RVC) foam electrodes were cut from a 6″ × 6″ sheet of RVC foam (100 ppi, 0.5″ thickness) purchased from ERG Aerospace. Decreased reaction yields were observed upon reuse of RVC electrodes, even after thorough washing with acetone and water followed by air-drying. The use of freshly cut RVC foam for each reaction was necessary to ensure reproducibility.

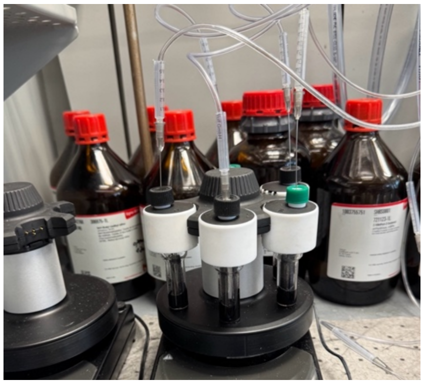

Figure S2. Graphical representation for a 6-vial reaction set up on Electrasyn Carousel

The reaction vials were placed until positive N_2 pressure through a thin nitrogen line connected to a 22G black needle which is pierced through the septa of each vial holder.

5) **Experimental and Characterization Data for Non-Literature Known Starting Materials**

Prepared according to General Procedure B *with tert-butyl 3-oxoazetidine-1-carboxylate ketone precursor*

tert-butyl (4-(1-methylcyclopropyl)phenyl)carbamate):

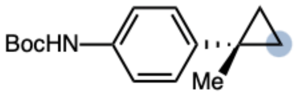

Prepared according to the general procedure **A** with tert-butyl N-(4-isopropenylphenyl)carbamate (116.7 mg, 0.5 mmol, 1.00 equiv), **Ir[dF(Me**)ppy]2(dtbbpy)PF6 (5.07 mg, 5.00 µmol, 0.01 equiv), Fe(OEP)Cl (23.4 mg, 37.5 µmol, 0.075 equiv), imidazole (2.55 mg, 37.5 µmol, 0.075 equiv), **HEH-1** (387 mg, 1.25 mmol, 2.5 equiv), and standard solvent mixture TAA/PhCN/H_2O (1.66 mL, 0.3 M). To this was added formaldehyde (247 µL, 2.50 mmol, 5 equiv) as a 38% commercial stock solution in water. The reaction was sparged for 5 minutes under positive N_2 pressure, then irradiated at 20% light intensity for 16 hours (1500 rpm, 2000 stir rate). After irradiation, the solvent was removed under reduced pressure. The crude residue was loaded onto 25 g Biotage Sf.r normal phase column and purified with eluents 0%-100% EtOAc in hexanes. The fractions containing product were concentrated and further purified by preparative HPLC (XBridge BEH C18 OBD column, 40%-100% MeCN in H_2O with 0.1% NH_4OH) to furnish product as a colorless oil (101.4 mg, 410 µmol, 82% yield).

^{1}H NMR (500 MHz, $CDCl_3$) δ 7.25 (d, *J* = 8.8 Hz, 2H), 7.18 (s, 2H), 6.39 (s, 1H), 1.51 (s, 9H), 1.37 (s, 3H), 0.83 – 0.77 (m, 2H), 0.71 – 0.65 (m, 2H).

^{13}C NMR (126 MHz, $CDCl_3$) δ 152.9, 141.9, 135.8, 127.4, 118.6, 28.4, 28.3, 25.9, 19.4, 15.3.

HRMS *m/z* calcd. For $C_{11}H_{14}NO_2^+$ ([M-iBu+H]$_+$) 192.1018, found 192.1018.

tert-butyl (1*S*,5*R*)-2-azabicyclo[3.1.0]hexane-2-carboxylate

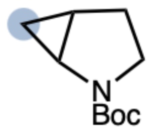

Prepared according to the general procedure **D** with N-(tert-butoxycarbonyl)-2,3-dihydropyrrole (17.3 µL, 0.1 mmol, scaled out 5 times, 1.00 equiv), 4ClCzIPN (2 mol%) (2.13 mg, 2.00 µmol, 0.02 equiv), Fe(TPP)Cl (5.3 mg, 0.008 mmol, 0.075 equiv), imidazole (0.3 mg, 5.00 µmol, 0.05 equiv), **HEH-1** (77 mg, 0.25 mmol, 2.5 equiv), and standard solvent mixture TAA/PhCN/H_2O (1.66 mL). To this was added formaldehyde (74 µL, 1.0 mmol, 10 equiv) as an aqueous 37% w/w with 10% methanol commercial stock solution. Reaction was sparged for 3 minutes under positive N_2 pressure, then irradiated at 50% light intensity for 24 hours. After irradiation, the five identical reactions were pooled, and the solvent was removed under reduced pressure. The crude residue was loaded onto 25 g Biotage Sf.r normal phase column and purified with eluents 0%–30% EtOAc in hexanes. The fractions containing product were concentrated and further purified by preparative HPLC (XBridge BEH C18 OBD column, 20%-100% MeCN in H_2O with 0.1% NH_4OH) to furnish the product as a pale yellow oil. Isolated and crude target characterization match reported spectra.[5] Target is reported as a 76% assay yield.

^{1}H NMR (500 MHz, $CDCl_3$) δ 3.62 (s, 1H), 3.34 (d, *J* = 13.4 Hz, 1H), 2.92 (q, *J* = 9.6 Hz, 1H), 2.05 (q, *J* = 9.7 Hz, 1H), 1.95 – 1.87 (m, 1H), 0.65 (s, 1H), 0.51 (td, *J* = 5.4, 2.5 Hz, 1H).

^{13}C NMR (126 MHz, $CDCl_3$) δ 155.44, 79.38, 43.79, 35.66, 28.63, 26.13 (apparent d, *J* = 89.1 Hz), 15.53 (apparent d, *J* = 97.4 Hz), 11.00.

HRMS (ESI-TOF) *m*/*z* calcd. For $C_6H_{10}NO_2^+$ ([M+H]$^+$) 128.0706, found 128.0705.

5'-bromo-1'-cyclopropylspiro[cyclopropane-1,3'-indolin]-2'-one

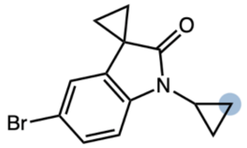

Prepared according to the general procedure **D** with 5'-bromo-1'-vinylspiro[cyclopropane-1,3'-indolin]-2'-one (132 mg, 0.5 mmol, 1.00 equiv), Ir(dFMeppy)$_2$(dtbbpy)PF$_6$ (1 mol%) (5.00 mg, 5.00 μmol, 0.01 equiv), Fe(OEP)Cl (12.5 mg, 0.02 mmol, 0.04 equiv), imidazole (1.4 mg, 0.02 mmol, 0.04 equiv), **HEH-1** (77 mg, 0.25 mmol, 2.5 equiv), and standard solvent mixture TAA/PhCN/H_2O (1.66 mL). To this was added formaldehyde (82 μL, 1.0 mmol, 2 equiv) as an aqueous commercial stock solution. Reaction was sparged for 5 minutes under positive N_2 pressure, then irradiated at 20% light intensity for 16 hours. After irradiation, the solvent was removed under reduced pressure. The crude residue was loaded onto 25 g Biotage Sf.r normal phase column and purified with eluents 0%-100% EtOAc in hexanes. The fractions containing product were concentrated and further purified by preparative HPLC (XBridge BEH C18 OBD column, 20%-100% MeCN in H_2O with 0.1% NH_4OH) to furnish product as a pale yellow oil (105.2 mg, 0.38 mmol, 76% yield).

^{1}H NMR (500 MHz, CDCl$_3$) δ 7.34 (dd, *J* = 8.3, 2.0 Hz, 1H), 7.01 (d, *J* = 8.3 Hz, 1H), 6.90 (d, *J* = 2.0 Hz, 1H), 2.67 (tt, *J* = 7.1, 3.8 Hz, 1H), 1.90 – 1.60 (m, 2H), 1.63 – 1.39 (m, 2H), 1.05 (td, *J* = 7.3, 5.3 Hz, 2H), 0.98 – 0.81 (m, 2H).

^{13}C NMR (126 MHz, CDCl$_3$) δ 176.99, 176.03, 143.03, 141.73, 133.40, 132.74, 129.43, 121.79, 121.45, 114.80, 114.49, 110.70, 109.46, 35.18, 27.12, 22.41, 19.85, 19.69, 12.93, 6.08.

HRMS (ESI-TOF) *m/z* calcd. For $C_{13}H_{12}BrNO^+$ ([M+H]$^+$) 278.0175, found 278.0176.

tert-butyl 7-azadispiro[2.1.3.2]decane-7-carboxylate

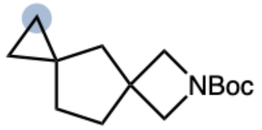

Prepared according to the general procedure **D** with minor modification in five identical replicates with *tert*-butyl 6-methylene-2-azaspiro[3.4]octane-2-carboxylate (16.9 mg, 0.1 mmol, 1.00 equiv), 4ClCzIPN (2.13 mg, 2.00 µmol, 0.02 equiv), Fe(TPP)Cl (10.6 mg, 0.015 mmol, 0.15 equiv), imidazole (0.34 mg, 5 µmol, 0.05 equiv), **HEH-1** (77 mg, 0.25 mmol, 2.5 equiv), and standard solvent mixture TAA/PhCN/H_2O (0.33 mL). To this was added formaldehyde (74 µL, 10 mmol, 10 equiv) as a aqueous 37% w/w with 10% methanol stock solution. Reaction was sparged for 3 minutes under positive N_2 pressure, then irradiated at 50% light intensity for 24 hours.

After irradiation, the solvent was removed under reduced pressure. The crude residue was loaded onto 25 g Biotage Sf.r normal phase column and purified with eluents 0% to 10% EtOAc in hexanes. The fractions containing product were concentrated and further purified by preparative HPLC (XBridge BEH C18 OBD column, 20%-100% MeCN in H_2O with 0.1% NH_4OH) to furnish product as a pale yellow oil (76 mg, 0.32 mmol, 64% yield).

^{1}H NMR (500 MHz, $CDCl_3$) δ 3.79 (d, *J* = 2.0 Hz, 4H), 1.92 (dd, *J* = 8.2, 6.5 Hz, 2H), 1.74 (s, 2H), 1.58 (dd, *J* = 8.2, 6.6 Hz, 2H), 1.43 (s, 9H), 0.58 – 0.19 (m, 4H).

^{13}C NMR (126 MHz, $CDCl_3$) δ 156.60, 79.24, 60.75, 47.49, 41.96, 38.43, 34.26, 28.55, 21.02, 12.83.

HRMS (ESI-TOF) *m*/*z* calcd. For $C_{10}H_{16}NO_2H^+$ ([M+H]$^+$) 182.11758, found 182.1180

tert-butyl 4-(((2R)-2-(4-fluorophenyl)-2-methylcyclopropyl)methyl)piperidine-1-carboxylate

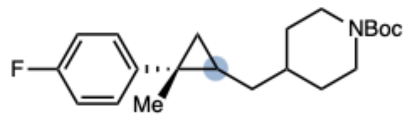

Prepared according to the general procedure **A** with tert-butyl 4-(2-oxoethyl)piperidine-1-carboxylate (114 mg, 0.5 mmol, 1.00 equiv), 2-(4-fluorophenyl)-1-propene (0.135 mL, 1 mmol, 2.00 equiv), Ir(dF(Me)ppy)$_2$(dtbppy)PF$_6$ (5.10 mg, 50 μmol, 0.01 equiv), Fe(OEP)Cl (12.5 mg, 0.02 mmol, 0.04 equiv), imidazole (1.4 mg, 0.02 mmol, 0.04 equiv), **HEH-1** (387 mg, 1.25 mmol, 2.5 equiv), and standard solvent mixture TAA/PhCN/H_2O (1.66 mL). Reaction was sparged for 5 minutes under positive N_2 pressure, then irradiated at 20% light intensity for 16 hours. After irradiation, the solvent was removed under reduced pressure. The crude residue was loaded onto 50 g Biotage Sf.r normal phase column and purified with eluents 0%-10% EtOAc in hexanes. The fractions containing product were concentrated and further purified by preparative HPLC (XBridge BEH C18 OBD column, 20%-100% MeCN in H_2O with 0.1% NH_4OH) to furnish product as a colorless oil (160 mg, 0.46 mmol, 92% yield (1.1:1 d.r.)).

^{1}H NMR (500 MHz, CDCl$_3$) δ 7.22 – 7.10 (m, 2H), 7.04 – 6.87 (m, 2H), 4.24 – 3.79 (m, 1H), 2.66 (dt, *J* = 34.7, 12.5 Hz, 2H), 1.75 (ddt, *J* = 39.1, 13.0, 2.8 Hz, 1H), 1.67 – 1.50 (m, 1H), 1.44 (d, *J* = 11.8 Hz, 9H), 1.38 – 1.22 (m, 4H), 1.21 – 1.09 (m, 1H), 1.07 – 0.83 (m, 2H), 0.80 – 0.60 (m, 1H), 0.44 – 0.25 (m, 1H).

^{13}C NMR (126 MHz, CDCl$_3$) δ 169.00, 160.96 (d, *J* = 243.4 Hz), 160.73 (d, *J* = 243.4 Hz), 154.97 (d, *J* = 2.4 Hz), 144.40 (d, *J* = 3.1 Hz), 139.68 (d, *J* = 3.2 Hz), 130.73 (d, *J* = 7.8 Hz), 128.39 (d, *J* = 7.8 Hz), 114.94 (dd, *J* = 21.1, 6.3 Hz), 98.89, 79.26, 79.19, 59.67, 37.80, 37.08, 36.65, 36.38, 32.46, 32.28, 28.71, 28.58, 28.56, 24.91, 24.10, 23.55, 23.36, 20.88 (d, *J* = 13.1 Hz), 18.95.

^{19}F NMR (471 MHz, CDCl$_3$) δ -117.29 – -117.40 (m), -117.93 – -118.08 (m).

HRMS *m*/*z* calcd. For $C_{17}H_{23}FNO_2^+$ ([M+H]) 292.1746, found 292.1753.

(9H-fluoren-9-yl)methyl (2-((2R)-2-(4-fluorophenyl)-2-methylcyclopropyl)ethyl)carbamate

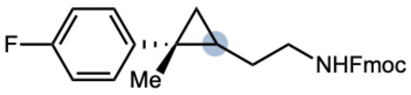

Prepared according to the general procedure **A** with 9H-fluoren-9-ylmethyl N-(3-oxopropyl)carbamate (147.7 mg, 0.5 mmol, 1.00 equiv), 1-fluoro-4-isopropenyl-benzene (134.82 µL, 1 mmol, 2.00 equiv), Ir(dF(Me)ppy)$_2$(dtbppy)PF$_6$ (5.10 mg, 50 µmol, 0.01 equiv), Fe(OEP)Cl (23.4 mg, 0.04 mmol, 0.075 equiv), imidazole (2.55 mg, 0.075 mmol, 0.075 equiv), **HEH-1** (387 mg, 1.25 mmol, 2.5 equiv), and standard solvent mixture TAA/PhCN/H_2O (1.66 mL). Reaction was sparged for 5 minutes under positive N_2 pressure, then irradiated at 20% light intensity for 16 hours. After irradiation, the solvent was removed under reduced pressure. The crude residue was loaded onto 50 g Biotage Sf.r normal phase column and purified with eluents 0%-10% EtOAc in hexanes. The fractions containing product were concentrated and further purified by preparative HPLC (XBridge BEH C18 OBD column, 20%-100% MeCN in H_2O with 0.1% NH4OH) to furnish product as a colorless oil (161.2 mg, 387.97 µmol, 77.6% yield, 1.6:1 dr).

Summary of diastereomers

^{1}H NMR (500 MHz, Chloroform-*d*) δ 7.79 (d, *J* = 7.6 Hz, 2H), 7.61 (t, *J* = 8.2 Hz, 2H), 7.42 (t, *J* = 7.5 Hz, 2H), 7.36 – 7.28 (m, 3H), 7.26 – 7.11 (m, 2H), 7.03 – 6.92 (m, 2H), 4.92 – 4.52 (m, 1H), 4.52 – 4.15 (m, 3H), 3.47 – 3.13 (m, 2H), 1.83 – 1.44 (m, 2H), 1.38 (s, 2H), 1.36 (s, 1H), 1.16 – 0.41 (m, 3H).

^{13}C NMR (126 MHz, Chloroform-*d*) δ 162.38 (d, *J* = 243.8 Hz), 162.08 (d, *J* = 243.8 Hz), 156.53, 156.45, 144.14, 144.12, 144.02, 141.47 (d, *J* = 1.9 Hz), 139.34, 130.76 (d, *J* = 7.3 Hz), 128.71 (d, *J* = 7.9 Hz), 127.80 (d, *J* = 2.2 Hz), 127.16, 127.15, 125.13, 120.12, 120.11, 115.20 (d, *J* = 21.9 Hz), 115.03 (d, *J* = 21.9 Hz), 66.64, 66.57, 47.46, 47.44, 41.55, 41.11, 31.51, 30.02, 28.56, 25.30, 23.53, 23.30, 20.95, 20.20, 18.19.

^{19}F NMR (470 MHz, $CDCl_3$) δ -116.83 (q, *J* = 7.1 Hz, 1F), -117.55 (q, *J* = 7.3 Hz, 1.6F).

HRMS (ESI-TOF) *m*/*z* calcd. For $C_{27}H_{26}FNO_2$ ($[M+H]^+$) 416.2021, found 416.2020

***tert*-butyl (4-((1R)-2-(2-((((9H-fluoren-9-yl)methoxy)carbonyl)amino)ethyl)-1 methylcyclopropyl)phenyl)carbamate**

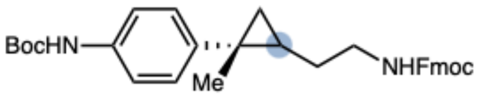

Prepared according to the general procedure **A** with (9H-Fluoren-9-yl)methyl (3-oxopropyl)carbamate (147.7 mg, 0.5 mmol, 1.00 equiv), tert-butyl N-(4-isopropenylphenyl)carbamate (233.31 mg, 1 mmol, 2.00 equiv), 4-CzIPN (3.9 mg, 50 µmol, 0.01 equiv), Fe(OEP)Cl (23.4 mg, 0.04 mmol, 0.075 equiv), imidazole (2.6 mg, 0.075 mmol, 0.05 equiv), **HEH-1** (387 mg, 1.25 mmol, 2.5 equiv), and standard solvent mixture TAA/PhCN/H_2O (1.66 mL). Reaction was sparged for 5 minutes under positive N_2 pressure, then irradiated at 20% light intensity for 16 hours. After irradiation, the solvent was removed under reduced pressure. The crude residue was loaded onto 50 g Biotage Sf.r normal phase column and purified with eluents 0%-25% EtOAc in hexanes. The fractions containing product were concentrated and further purified by preparative HPLC (XBridge BEH C18 OBD column, 20%-100% MeCN in H_2O with 0.1% NH4OH) to furnish product as white solid (238.8 mg, 465.8 µmol, 93.2% yield 1.7:1 dr).

Summary of diastereomers
^{1}H NMR (500 MHz, Chloroform-*d*) δ 7.76 (d, J = 7.6 Hz, 2H), 7.59 (dd, J = 7.5, 7.5 Hz, 2H), 7.40 (dd, J = 7.5, 7.5 Hz, 2H), 7.30 (ddd, J = 7.5, 7.5, 1.1 Hz, 2H), 7.28 – 7.21 (m, 2H), 7.15 (dd, J = 18.8, 8.2 Hz, 2H), 6.45 – 6.39 (m, 1H), 4.99 – 4.15 (m, 4H), 3.43 – 3.00 (m, 2H), 1.81 – 1.42 (m, 2H), 1.51 (d, J = 1.5 Hz, 9H), 1.34 (d, 3H), 1.13 – 0.26 (m, 3H).

^{13}C NMR (126 MHz, $CDCl_3$) δ 156.52, 156.44, 152.99, 144.18, 143.11, 141.45, 138.31, 136.44, 136.04, 129.80, 127.79, 127.77, 127.60, 127.16, 125.18, 120.10, 120.08, 118.83, 118.68, 80.55, 66.65, 66.58, 47.44, 41.58, 41.15, 31.41, 30.04, 28.49, 28.41, 25.31, 23.60, 23.52, 23.37, 20.71, 20.22, 17.95.

HRMS (ESI-TOF) *m*/*z* calcd. For $C_{32}H_{37}N_2O_4$ ($[M+H]^+$) 513.2748, found 513.2755

***tert*-butyl ((1R)-1-(2-(4-fluorophenyl)cyclopropyl)-2-phenylethyl)carbamate**

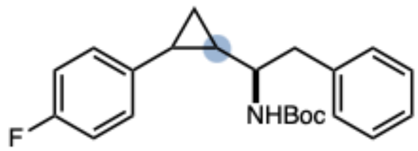

Prepared according to the general procedure **A** with tert-butyl (1-oxo-3-phenylpropan-2-yl)carbamate (124.65 mg, 0.5 mmol, 1.00 equiv), 1-fluoro-4-vinyl-benzene (120.0 µL, 1 mmol, 2.00 equiv), 4-CzIPN (3.9 mg, 50 µmol, 0.01 equiv), Fe(OEP)Cl (23.4 mg, 0.04 mmol, 0.075 equiv), imidazole (2.6 mg, 0.075 mmol, 0.05 equiv), **HEH-1** (387 mg, 1.25 mmol, 2.5 equiv), and standard solvent mixture TAA/PhCN/H_2O (1.66 mL). Reaction was sparged for 5 minutes under positive N_2 pressure, then irradiated at 50% light intensity for 16 hours. After irradiation, the solvent was removed under reduced pressure. The crude residue was loaded onto 50 g Biotage Sf.r normal phase column and purified with eluents 0%-50% EtOAc in hexanes. The fractions containing product were concentrated and further purified by preparative HPLC (XBridge BEH C18 OBD column, 20%-100% MeCN in H_2O with 0.1% NH4OH) to furnish product as a white solid (109.2 mg, 307.2 µmol, 61.4% yield, 5:1 dr).

Summary of Diastereomers
^{1}H NMR (500 MHz, $CDCl_3$) δ 7.29 (t, *J* = 7.8 Hz, 0.2H), 7.20 (td, *J* = 5.5, 2.6 Hz, 3H), 7.14 – 7.06 (m, 2H), 6.90 (t, *J* = 8.7 Hz, 2H), 6.82 – 6.76 (m, 2H), 4.54 (br, 1H), 3.40 (br, 1H), 2.98 (dd, *J* = 13.5, 5.5 Hz, 1H), 2.92 (dd, *J* = 6.2, 2.7 Hz, 0.2H), 2.86 (dd, *J* = 13.4, 7.1 Hz, 1H), 1.69 – 1.54 (m, 1H), 1.44 (s, 8H), 1.42 (s, 1H), 1.06 – 0.96 (m, 2H), 0.92 – 0.75 (m, 1H)**.**

^{13}C NMR (126 MHz, $CDCl_3$) δ 161.25 (d, *J* = 243.1 Hz), 155.54, 137.90, 129.82, 129.76, 128.47, 127.41 (d, *J* = 7.6 Hz), 127.20 (d, *J* = 7.7 Hz), 126.55, 126.45, 115.04 (d, *J* = 21.3 Hz), 79.48, 55.98, 41.85, 28.54, 27.65, 22.19, 20.90, 14.27, 13.76.

^{19}F NMR (471 MHz, $CDCl_3$) δ -118.00 (s, 1F).

HRMS (ESI-TOF) *m*/*z* calcd. For $NaC_{22}H_{26}FNO_2$ ($[M+Na]^+$) 378.1840, found 378.1840

***tert*-butyl 6-(2-(4-chlorophenyl)cyclopropyl)-3-azabicyclo[3.1.0]hexane-3-carboxylate**

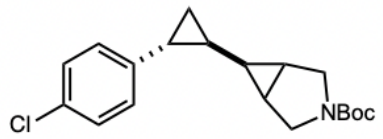

Prepared according to the general procedure **A** with tert-butyl 6-formyl-3-azabicyclo[3.1.0]hexane-3-carboxylate (105.63 mg, 0.5 mmol, 1.00 equiv), 1-chloro-4-vinyl-benzene (138.6 mg, 1 mmol, 2.00 equiv), 4-CzIPN (3.9 mg, 50 μmol, 0.01 equiv), Fe(OEP)Cl (23.4 mg, 0.04 mmol, 0.075 equiv), imidazole (2.6 mg, 0.075 mmol, 0.05 equiv), **HEH-1** (387 mg, 1.25 mmol, 2.5 equiv), and standard solvent mixture TAA/PhCN/H_2O (1.66 mL). Reaction was sparged for 5 minutes under positive N_2 pressure, then irradiated at 20% light intensity for 16 hours. After irradiation, the solvent was removed under reduced pressure. The crude residue was loaded onto 50 g Biotage Sf.r normal phase column and purified with eluents 0%-10% EtOAc in hexanes. The fractions containing product were concentrated and further purified by preparative HPLC (XBridge BEH C18 OBD column, 20%-100% MeCN in H_2O with 0.1% NH_4OH) to furnish product as a white solid (98.7 mg, 295.6 μmol, 59.1% yield, >20:1 d.r.).

^{1}H NMR (500 MHz, $CDCl_3$) δ 7.22 – 7.16 (m, 2H), 6.97 – 6.90 (m, 2H), 3.56 – 3.52 (m, 2H), 3.33 (dd, *J* = 10.8, 4.3 Hz, 2H), 1.66 (dt, *J* = 8.5, 5.1 Hz, 1H), 1.43 (s, 9H), 1.37 (dt, *J* = 7.7, 3.8 Hz, 1H), 1.33 (dt, *J* = 7.5, 3.8 Hz, 1H), 1.03 (ddt, *J* = 9.1, 6.0, 4.8 Hz, 1H), 0.86 – 0.76 (m, 2H), 0.71 (q, *J* = 5.1 Hz, 1H).

^{13}C NMR (126 MHz, $CDCl_3$) δ 155.06, 141.80, 131.12, 128.46, 127.10, 79.40, 48.41, 28.63, 24.40, 23.55, 21.69 (HSQC shows a second carbon underneath this peak), 14.62.

HRMS *m*/*z* calcd. For $C_{15}H_{16}ClNO_2^+$ ([M+H]+) 278.09428, found 278.09369.

methyl 4-(2-isopropylcyclopropyl)benzoate

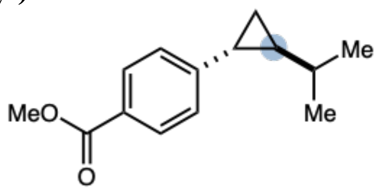

Prepared according to the general procedure **A** with methyl 4-vinylbenzoate (162.2 mg, 1.0 mmol, 2.00 equiv), 2-methylpropanal (45.4 µL, 0.5 mmol, 1.0 equiv), **4CzIPN** (0.8 mg, 5.00 µmol, 0.01 equiv), Fe(OEP)Cl (23.4 mg, 37.5 µmol, 0.075 equiv), imidazole (2.55 mg, 37.5 µmol, 0.075 equiv), **HEH-1** (387 mg, 1.25 mmol, 2.5 equiv), and standard solvent mixture TAA/PhCN/H_2O (1.66 mL, 0.3 M). The reaction was sparged for 5 minutes under positive N_2 pressure, then irradiated at 20% light intensity for 16 hours (1500 rpm, 2000 stir rate).

After irradiation, the solvent was removed under reduced pressure. The crude residue was loaded onto 25 g Biotage Sf.r normal phase column and purified with eluents 0%-100% EtOAc in hexanes. The fractions containing product were concentrated and further purified by preparative HPLC (XBridge BEH C18 OBD column, 40%-100% MeCN in H_2O with 0.1% NH_4OH) to furnish product as a colorless oil (109.2 mg, 255 µmol, 51% yield, >20:1 d.r.).

^{1}H NMR (500 MHz, $CDCl_3$) δ 7.93 – 7.86 (m, 2H), 7.11 – 7.05 (m, 2H), 3.89 (s, 3H), 1.70 (dt, *J* = 7.8, 4.9, 4.9 Hz, 1H), 1.17 – 1.07 (m, 1H), 1.02 (d, *J*= 6.4 Hz, 6H), 0.95 – 0.86 (m, 3H).

^{13}C NMR (126 MHz, $CDCl_3$) δ 167.29, 150.26, 129.73, 127.10, 125.50, 52.02, 33.62, 33.12, 22.85, 22.20, 21.88, 16.35.

HRMS *m/z* calcd. For $C_{14}H_{19}O_2^+$ ($[M+H]^+$) 219.1380, found 219.1389.

3-((1R,2R)-2-(4-fluorophenyl)cyclopropyl)oxetane:

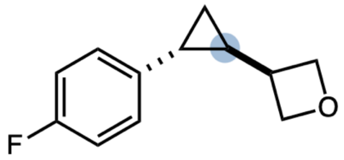

Prepared according to the general procedure **A** with 1-fluoro-4-vinyl-benzene (122 mg, 1.0 mmol, 2.00 equiv), oxetane-3-carbaldehyde (43.0 mg, 0.5 mmol, 1.0 equiv), **4CzIPN** (0.8 mg, 5.00 µmol, 0.01 equiv), Fe(OEP)Cl (23.4 mg, 37.5 µmol, 0.075 equiv), imidazole (2.55 mg, 37.5 µmol, 0.075 equiv), **HEH-1** (387 mg, 1.25 mmol, 2.5 equiv), and standard solvent mixture TAA/PhCN/H_2O (1.66 mL, 0.3 M). The reaction was sparged for 5 minutes under positive N_2 pressure, then irradiated at 20% light intensity for 16 hours (1500 rpm, 2000 stir rate).

After irradiation, the solvent was removed under reduced pressure. The crude residue was loaded onto 25 g Biotage Sf.r normal phase column and purified with eluents 0%-100% EtOAc in hexanes. The fractions containing product were concentrated and further purified by preparative HPLC (XBridge BEH C18 OBD column, 40%-100% MeCN in H_2O with 0.1% NH_4OH) to furnish product as a yellow oil (81.0 mg, 421 µmol, 84% yield, >20:1 d.r.).

^{1}H NMR (500 MHz, $CDCl_3$) δ 7.02 (dd, *J* = 8.5, 5.4 Hz, 2H), 6.95 (t, *J* = 8.7 Hz, 2H), 4.81 (dt, *J* = 6.4, 2.2 Hz, 2H), 4.51 (td, *J* = 6.3, 3.1 Hz, 2H), 2.83 (hept, *J* = 6.7 Hz, 1H), 1.73 (dt, *J* = 9.3, 4.9 Hz, 1H), 1.41 (ddd, *J* = 13.5, 8.4, 5.1 Hz, 1H), 0.91 (dt, *J* = 8.7, 5.2 Hz, 1H), 0.85 (dt, *J* = 8.7, 5.5 Hz, 1H).

^{13}C NMR (126 MHz, $CDCl_3$) δ 161.24 (d, *J* = 243.4 Hz), 138.27 (d, *J* = 3.1 Hz), 127.37 (d, *J* = 7.8 Hz), 115.20 (d, *J* = 21.3 Hz), 76.7, 76.6, 38.8, 25.2, 20.6, 13.8.

^{19}F NMR (376 MHz, $CDCl_3$) δ -117.75 – -117.81 (m).

HRMS *m/z* calcd. For $C_{12}H_{14}FO^+$ ([M+H]+) 193.1073, found 193.1077.

tert-butyl 3-(2-(4-fluorophenyl)cyclopropyl)azetidine-1-carboxylate

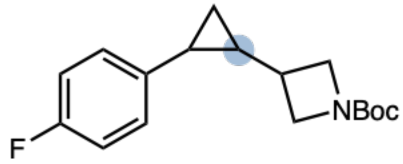

Prepared according to the general procedure **A** with tert-butyl 3-formylazetidine-1-carboxylate (92.6 mg, 0.5 mmol, 1.00 equiv), 1-fluoro-4-vinylbenzene (120.0 µl, 1 mmol, 2.00 equiv), 4-CzIPN (3.9 mg, 50 µmol, 0.01 equiv), Fe(OEP)Cl (23.4 mg, 0.04 mmol, 0.075 equiv), imidazole (2.6 mg, 0.075 mmol, 0.05 equiv), **HEH-1** (387 mg, 1.25 mmol, 2.5 equiv), and standard solvent mixture TAA/PhCN/H_2O (1.66 mL). Reaction was sparged for 5 minutes under positive N_2 pressure, then irradiated at 20% light intensity for 16 hours. After irradiation, the solvent was removed under reduced pressure. The crude residue was loaded onto 50 g Biotage Sf.r normal phase column and purified with eluents 0%-100% EtOAc in hexanes. The fractions containing product were concentrated and further purified by preparative HPLC (XBridge BEH C18 OBD column, 20%-100% MeCN in H_2O with 0.1% NH4OH) to furnish product as a colorless oil (104.1 mg, 0.389 µmol, 71.5% yield, >20:1 d.r.).

^{1}H NMR (500 MHz, $CDCl_3$) δ 7.00 (dd, *J* = 8.7, 5.4 Hz, 2H), 6.92 (t, *J* = 8.7 Hz, 2H), 4.08 (t, *J* = 11.6 Hz, 2H), 2.68 (tt, *J* = 12.6, 3.0 Hz, 2H), 1.82 – 1.71 (m, 2H), 1.68 (dt, *J* = 7.9, 5.0 Hz, 1H), 1.46 (s, 10H), 1.30 (dddd, *J* = 22.6, 12.9, 11.3, 4.3 Hz, 2H), 0.96 – 0.86 (m, 1H), 0.86 – 0.77 (m, 3H).

^{13}C NMR (126 MHz, $CDCl_3$) δ 161.16 (d, *J* = 243.1 Hz), 155.05, 139.14 (d, *J* = 3.0 Hz), 127.45 (d, *J* = 7.7 Hz), 115.04 (d, *J* = 20.9 Hz), 79.44, 44.08, 41.30, 32.02, 31.62, 28.91, 28.63, 21.33, 14.30.

^{19}F NMR (471 MHz, $CDCl_3$) δ -118.22 (t, *J* = 5.3 Hz, 1F).

HRMS (ESI-TOF) *m*/*z* calcd. For $C_{17}H_{22}FNO_2^+$ ([M+H]$^+$) 236.1082, found 236.1089.

benzyl 4-(2-(4-fluorophenyl)cyclopropyl)piperidine-1-carboxylate

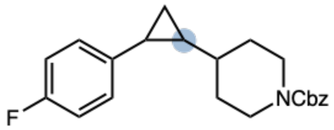

Prepared according to the general procedure **A** with benzyl 4-formylpiperidine-1-carboxylate (123.6 mg, 0.5 mmol, 1.00 equiv), 1-fluoro-4-vinylbenzene (120.0 µl, 1.00 mmol, 2.0 equiv.), 4-CzIPN (3.9 mg, 50 µmol, 0.01 equiv), Fe(OEP)Cl (23.4 mg, 0.04 mmol, 0.075 equiv), imidazole (2.6 mg, 0.075 mmol, 0.05 equiv), **HEH-1** (387 mg, 1.25 mmol, 2.5 equiv), and standard solvent mixture TAA/PhCN/H_2O (1.66 mL). Reaction was sparged for 5 minutes under positive N_2 pressure, then irradiated at 20% light intensity for 16 hours. After irradiation, the solvent was removed under reduced pressure. The crude residue was loaded onto 50 g Biotage Sf.r normal phase column and purified with eluents 0%-100% EtOAc in hexanes. The fractions containing product were concentrated and further purified by preparative HPLC (XBridge BEH C18 OBD column, 20%-100% MeCN in H_2O with 0.1% NH_4OH) to furnish product as a colorless oil (135.0 mg, 382.0 µmol, 76.4% yield, >20:1 d.r.).

^{1}H NMR (500 MHz, Chloroform-*d*) δ 7.40 – 7.30 (m, 5H), 7.00 (dd, *J* = 8.7, 5.4 Hz, 2H), 6.97 – 6.87 (m, 2H), 5.14 (s, 2H), 4.21 – 4.17 (m, 2H), 2.85 – 2.73 (m, 2H), 1.80 (t, *J* = 14.6 Hz, 2H), 1.69 (dt, *J* = 7.9, 5.0 Hz, 1H), 1.43 – 1.29 (m, 1H), 0.99 – 0.88 (m, 1H), 0.87 – 0.75 (m, 3H).

^{13}C NMR (126 MHz, Chloroform-*d*) δ 161.13 (d, *J* = 243.2 Hz), 155.42, 139.00 (d, *J* = 3.0 Hz), 137.08, 128.59, 128.00 (d, *J* = 11.4 Hz), 127.37 (d, *J* = 7.7 Hz), 115.11 (d, *J* = 21.3 Hz), 67.09, 44.26, 44.24, 41.15, 32.68 – 30.59 (m) (broad due to N-Cbz rotamers), 28.80, 21.32, 14.28.

^{19}F NMR (471 MHz, Chloroform-*d*) δ -118.09.

HRMS (ESI-TOF) *m*/*z* calcd. For $C_{22}H_{24}FNO_2^+$ ([M+H]$^+$) 354.1864, found 354.1866.

***tert*-butyl ((1R,4r)-4-((1R)-2-(3,4-difluorophenyl)cyclopropyl)cyclohexyl)carbamate**

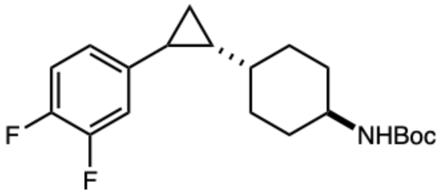

Prepared according to the general procedure **A** with tert-butyl N-(4-formylcyclohexyl)carbamate (113.7 mg, 0.5 mmol, 1.00 equiv), 1,2-difluoro-4-vinyl-benzene (123.7 µl, 1.00 mmol, 2.0 equiv.), 4-CzIPN (3.9 mg, 50 µmol, 0.01 equiv), Fe(OEP)Cl (23.4 mg, 0.04 mmol, 0.075 equiv), imidazole (2.6 mg, 0.075 mmol, 0.05 equiv), **HEH-1** (387 mg, 1.25 mmol, 2.5 equiv), and standard solvent mixture TAA/PhCN/H_2O (1.66 mL). Reaction was sparged for 5 minutes under positive N_2 pressure, then irradiated at 20% light intensity for 16 hours. After irradiation, the solvent was removed under reduced pressure. The crude residue was loaded onto 50 g Biotage Sf.r normal phase column and purified with eluents 0%-100% EtOAc in hexanes. The fractions containing product were concentrated and further purified by preparative HPLC (XBridge BEH C18 OBD column, 20%-100% MeCN in H_2O with 0.1% NH4OH) to furnish product as a white solid (82.0 mg, 233.3 µmol, 46.7% yield, >20:1 d.r.).

^{1}H NMR (500 MHz, $CDCl_3$) δ 7.00 (dt, *J* = 10.3, 8.4 Hz, 1H), 6.79 (ddd, *J* = 11.7, 7.5, 2.2 Hz, 1H), 6.74 (ddd, *J* = 8.6, 4.1, 1.8 Hz, 1H), 4.52 – 4.12 (m, 1H), 3.44 – 3.22 (m, 1H), 2.07 – 1.97 (m, 3H), 1.92 – 1.81 (m, 2H), 1.61 (dt, *J* = 8.0, 5.1 Hz, 1H), 1.44 (s, 9H), 1.30 – 1.14 (m, 3H), 1.04 (tdt, *J* = 12.9, 11.6, 3.8 Hz, 2H), 0.84 – 0.72 (m, 4H), 0.72 – 0.60 (m, 1H).

^{13}C NMR (126 MHz, $CDCl_3$) δ 155.38, 150.39 (dd, *J* = 234.4, 12.7 Hz), 148.11 (dd, *J* = 232.6, 12.7 Hz), 141.01 (dd, *J* = 5.8, 3.5 Hz), 121.75 (dd, *J* = 6.0, 3.3 Hz), 116.94 (d, *J* = 17.1 Hz), 114.59 (d, *J* = 17.3 Hz), 79.24, 49.91, 42.24, 33.52, 33.48, 31.66, 31.21, 29.85, 28.58, 21.52, 21.50, 14.88.

^{19}F NMR (471 MHz, $CDCl_3$) δ -138.69 (ddd, *J* = 20.7, 11.8, 8.3 Hz, 1F), -142.59 – -143.38 (m, 1F).

HRMS (ESI-TOF) *m/z* calcd. For $C_{22}H_{24}FNO_2^+$ ([M+H]$^+$) 354.1864, found 354.1866.

tert-butyl 9-(2-(4-fluorophenyl)cyclopropyl)-3-azaspiro[5.5]undecane-3-carboxylate

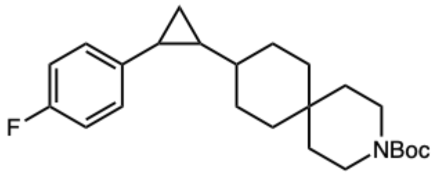

Prepared according to the general procedure **A** with tert-butyl 2-formyl-7-azaspiro[3.5]nonane-7-carboxylate (140.7 mg, 0.5 mmol, 1.00 equiv), 4-Fluorostyrene (120 µL, 1 mmol, 2.00 equiv), 4-CzIPN (3.9 mg, 50 µmol, 0.01 equiv), Fe(OEP)Cl (23.4 mg, 0.04 mmol, 0.075 equiv), imidazole (2.6 mg, 0.075 mmol, 0.05 equiv), **HEH-1** (387 mg, 1.25 mmol, 2.5 equiv), and standard solvent mixture TAA/PhCN/H_2O (1.66 mL). Reaction was sparged for 5 minutes under positive N_2 pressure, then irradiated at 20% light intensity for 16 hours. After irradiation, the solvent was removed under reduced pressure. The crude residue was loaded onto 50 g Biotage Sf.r normal phase column and purified with eluents 0%-100% EtOAc in hexanes. The fractions containing product were concentrated and further purified by preparative HPLC (XBridge BEH C18 OBD column, 20%-100% MeCN in H_2O with 0.1% NH_4OH) to furnish product as a colorless oil (123.3 mg, 318.2 µmol, 63.6% yield, >20:1 d.r.).

^{1}H NMR (500 MHz, $CDCl_3$) δ 7.00 (dd, *J* = 8.6, 5.5 Hz, 2H), 6.91 (dd, *J* = 8.7, 8.7 Hz, 2H), 3.35 (ddd, *J* = 12.8, 7.1, 4.6 Hz, 4H), 1.77 – 1.61 (m, 5H), 1.53 – 1.39 (m, 2H), 1.45 (s, (9H), 1.33 – 1.21 (m, 4H), 1.14 – 1.01 (m, 2H), 0.88 – 0.64 (m, 4H).

^{13}C NMR (126 MHz, $CDCl_3$) δ 161.06 (d, *J* = 242.7 Hz), 155.21, 139.60 (d, *J* = 2.9 Hz), 127.38 (d, *J* = 7.9 Hz), 115.04 (d, *J* = 21.3 Hz), 79.28, 43.23, 39.99, 35.70, 35.64, 31.90, 31.16, 29.65, 28.63, 27.77, 27.32, 21.38.

^{19}F NMR (471 MHz, $CDCl_3$) δ -118.50 (tt, *J* = 9.2, 5.3 Hz, 1F).

HRMS (ESI-TOF) *m/z* calcd. For $C_{24}H_{34}FNO_2^+$ ([M+H]$^+$) 332.2021, found 332.2024.

3-((1R)-2-(4-fluorophenyl)cyclopropyl)-3-methyloxetane

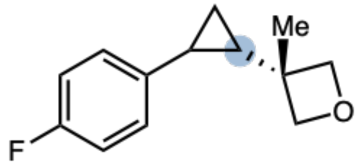

Prepared according to the general procedure **A** with 1-fluoro-4-vinyl-benzene (122 mg, 1.0 mmol, 2.00 equiv), 3-methyloxetane-3-carbaldehyde (50.1 mg, 0.5 mmol, 1.0 equiv), **4CzIPN** (0.8 mg, 5.00 µmol, 0.01 equiv), Fe(OEP)Cl (23.4 mg, 37.5 µmol, 0.075 equiv), imidazole (2.55 mg, 37.5 µmol, 0.075 equiv), **HEH-1** (387 mg, 1.25 mmol, 2.5 equiv), and standard solvent mixture TAA/PhCN/H_2O (1.66 mL, 0.3 M). The reaction was sparged for 5 minutes under positive N_2 pressure, then irradiated at 20% light intensity for 16 hours (1500 rpm, 2000 stir rate).

After irradiation, the solvent was removed under reduced pressure. The crude residue was loaded onto 25 g Biotage Sf.r normal phase column and purified with eluents 0%-100% EtOAc in hexanes. The fractions containing product were concentrated and further purified by preparative HPLC (XBridge BEH C18 OBD column, 40%-100% MeCN in H_2O with 0.1% NH_4OH) to furnish product as a yellow oil (103.1 mg, 266 µmol, 53% yield, >20:1 d.r.).

^{1}H NMR (500 MHz, $CDCl_3$) δ 7.09 – 7.02 (m, 2H), 6.99 – 6.91 (m, 2H), 4.40 (t, *J* = 5.5, 5.5 Hz, 2H), 4.35 (dd, *J* = 5.8, 1.7 Hz, 2H), 1.89 (dt, *J* = 8.8, 5.1, 5.1 Hz, 1H), 1.38 (s, 3H), 1.21 (ddd, *J* = 8.8, 6.0, 4.9 Hz, 1H), 1.03 (dt, *J* = 8.8, 5.7, 5.7 Hz, 1H), 0.93 (dt, *J* = 8.9, 5.4, 5.4 Hz, 1H).

^{13}C NMR (126 MHz, $CDCl_3$) δ 161.26 (d, *J* = 243.3 Hz), 138.44 (d, *J* = 3.1 Hz), 127.52 (d, *J* = 7.8 Hz), 115.20 (d, *J* = 21.2 Hz), 81.02, 80.88, 39.54, 29.51, 24.40, 18.95, 11.89.

^{19}F NMR (376 MHz, $CDCl_3$) δ -117.82 (ddt, *J* = 11.9, 5.9, 3.3, 3.3 Hz).

HRMS *m/z* calcd. For $C_{13}H_{16}FO^+$ ([M+H]$^+$) 207.1173, found 207.1176.

4-(3-(3-((1R,2R)-2-(4-fluorophenyl)-2-methylcyclopropyl)propyl)-4,4-dimethyl-2,5-dioxoimidazolidin-1-yl)-2-methylbenzonitrile

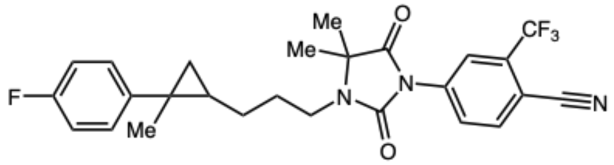

Prepared according to the general procedure **A** with 1-fluoro-4-isopropenyl-benzene (136 mg, 1.0 mmol, 2.00 equiv), 4-[4,4-dimethyl-2,5-dioxo-3-(4-oxobutyl)imidazolidin-1-yl]-2-(trifluoromethyl)benzonitrile (183.7 mg, 0.5 mmol, 1.0 equiv), Ir[dF(Me)ppy]2(dtbbpy)PF6 (5.1 mg, 5.00 µmol, 0.01 equiv), Fe(OEP)Cl (23.4 mg, 37.5 µmol, 0.075 equiv), imidazole (2.55 mg, 37.5 µmol, 0.075 equiv), **HEH-1** (387 mg, 1.25 mmol, 2.5 equiv), and standard solvent mixture TAA/PhCN/H_2O (1.66 mL, 0.3 M). The reaction was sparged for 5 minutes under positive N_2 pressure, then irradiated at 20% light intensity for 16 hours (1500 rpm, 2000 stir rate).

After irradiation, the solvent was removed under reduced pressure. The crude residue was loaded onto 25 g Biotage Sf.r normal phase column and purified with eluents 0%-100% EtOAc in hexanes. The fractions containing product were concentrated and further purified by preparative HPLC (XBridge BEH C18 OBD column, 40%-100% MeCN in H_2O with 0.1% NH_4OH) to furnish product as a yellow oil (153.0 mg, 266 µmol, 62.8% yield, 3:1 d.r.).

^{1}H NMR (500 MHz, $CDCl_3$) δ 8.16 (dd, J = 16.5, 2.1 Hz, 1H), 8.00 (ddd, J = 21.3, 8.5, 2.1 Hz, 1H), 7.90 (dd, J = 8.5, 4.4 Hz, 1H), 7.21 (ddd, J = 17.6, 8.4, 5.5 Hz, 2H), 6.95 (q, J = 9.1, 9.1, 8.7 Hz, 2H), 3.50 – 3.35 (m, 1H), 3.32 – 3.09 (m, 1H), 1.98 – 1.20 (m, 13H), 1.11 – 0.53 (m, 2H), 0.40 (t, J = 5.2, 5.2 Hz, 1H). [Summary of diastereomers]

^{13}C NMR (126 MHz, $CDCl_3$) δ 174.7, 174.6, 161.24 (d, J = 243.9 Hz), 160.97 (d, J = 243.7 Hz),152.8, 152.6, 144.04 (d, J = 3.1 Hz), 139.33 (d, J = 3.1 Hz), 133.58 (q, J = 33.2 Hz), 133.55 (q, J = 32.7 Hz), 130.66 (d, J = 7.8 Hz), 128.61 (d, J = 7.8 Hz), 127.9, 122.53 (q, J = 274.3 Hz), 122.98 (q, J = 4.7 Hz), 115.06, 114.94 (d, J = 21.1 Hz), 114.90 (d, J = 21.1 Hz), 108.20 (d, J = 2.3 Hz),108.1, 61.9, 61.8, 40.3, 40.3, 30.0, 29.3, 28.5, 28.4, 27.0, 25.6, 25.4, 25.2, 23.8, 23.6, 23.5, 23.4, 23.4, 22.7, 20.7, 20.4, 18.3. [Summary of diastereomers]

^{19}F NMR (376 MHz, $CDCl_3$) δ -62.00, -117.07 (ddd, *J* = 14.3, 8.9, 5.5 Hz), -117.74 (td, *J* = 8.9, 8.9, 4.5 Hz). [summary of diastereomers]

HRMS *m*/*z* calcd. For $C_{26}H_{25}F_4N_3NaO_2^+$ ([M+Na]$_+$) 510.1775, found 510.1791.

1-((3-chloro-1,5-dimethyl-1H-pyrazol-4-yl)sulfonyl)-4-(2-((1R,2R)-2-(4-fluorophenyl)-2-methylcyclopropyl)ethyl)piperidine

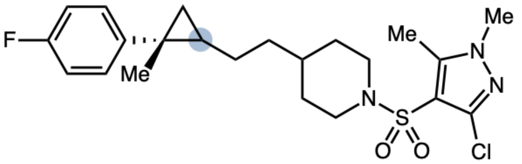

Prepared according to the general procedure **A** with c3-(1-((1,3,5-trimethyl-1H-pyrazol-4-yl)sulfonyl)piperidin-4-yl)propanal (167 mg, 0.5 mmol, 1.00 equiv), 1-fluoro-4-(1-methylethenyl)benzene (135 μL, 1 mmol, 2.00 equiv), Ir(dF(Me)ppy)$_2$(dtbppy)PF$_6$ (5 mg, 5 μmol, 0.01 equiv), Fe(OEP)Cl (12.5 mg, 0.02 mmol, 0.004 equiv), imidazole (1.7 mg, 0.025 mmol, 0.05 equiv), **HEH-1** (387 mg, 1.25 mmol, 2.5 equiv), and standard solvent mixture TAA/PhCN/H_2O (1.66 mL). Reaction was sparged for 5 minutes under positive N_2 pressure, then irradiated at 20% light intensity for 16 hours. After irradiation, the solvent was removed under reduced pressure. The crude residue was loaded onto 25 g Biotage Sf.r normal phase column and purified with eluents 0%-100% EtOAc in hexanes. The fractions containing product were concentrated and further purified by preparative HPLC (XBridge BEH C18 OBD column, 20%-100% MeCN in H_2O with 0.1% NH_4OH) to furnish product as a clear solid (197.5 mg, 0.44 mmol, 87% yield, (3:1) d.r.).

^{1}H NMR (500 MHz, CDCl$_3$) δ 7.24 – 7.11 (m, 2H), 6.98 – 6.81 (m, 2H), 3.79 (d, *J* = 8.8 Hz, 4H), 2.43 (td, *J* = 11.4, 2.6 Hz, 1H), 2.39 (s, 2H), 2.36 (s, 1H), 2.31 (dt, *J* = 11.8, 1.9 Hz, 1H), 1.83 – 1.65 (m, 1H), 1.64 – 1.55 (m, 1H), 1.43 (m, *J* = 15.5, 13.2, 10.0, 6.9, 3.9 Hz, 2H), 1.30 (d, *J* = 5.2 Hz, 4H), 1.19 – 1.04 (m, 1H), 1.04 – 0.94 (m, 1H), 0.91 – 0.74 (m, 1H), 0.69 – 0.49 (m, 1H), 0.31 (dd, *J* = 5.9, 4.5 Hz, 1H).

^{13}C NMR (126 MHz, CDCl$_3$) δ 169.27, 166.58, 161.15 (d, *J* = 243.7 Hz), 160.88 (d, *J* = 243.5 Hz), 149.20, 149.16, 144.43 (d, *J* = 3.1 Hz), 139.58 (d, *J* = 3.2 Hz), 130.63 (d, *J* = 7.7 Hz), 129.74, 129.70, 128.59 (d, *J* = 7.8 Hz), 114.89 (d), 114.64 (d, *J* = 21.1 Hz), 112.77, 112.68, 46.15, 46.10 (d, *J* = 1.5 Hz), 36.71, 36.69, 36.55, 35.88, 35.16, 34.98, 31.68, 31.53, 31.48, 31.41, 28.55, 27.96, 26.31 (d, *J* = 69.8 Hz), 25.72 (d, *J* = 51.1 Hz), 23.67, 22.21, 20.62, 20.40, 18.23, 14.20, 14.17.

^{19}F NMR (471 MHz, CDCl3) δ -117.33 (tt, J = 8.7, 5.5 Hz), -117.99 (tt, J = 8.5, 5.3 Hz).

HRMS (ESI-TOF) *m/z* calcd. For $C_{22}H_{30}ClFN_3O_2S^+$ ([M+H]$^+$) 454.1726, found 454.1731.

1-((2S)-2-methyl-2-(naphthalen-2-yl)cyclopropyl)-1H-pyrrolo[2,3-b]pyridine

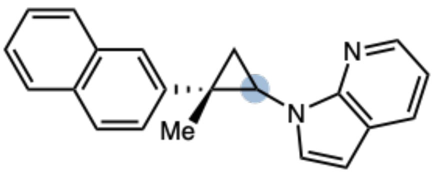

Prepared according to the general procedure **B** using formamide 1*H*-pyrrolo[2,3-*b*]pyridine-1-carbaldehyde (73 mg, 0.5 mmol, 1.00 equiv), 2-(prop-1-en-2-yl)naphthalene (136 mg, 1 mmol, 2.00 equiv), $Ir(dF(Me)ppy)_2(dtbppy)PF_6$ (10.1 mg, 10 μmol, 0.02 equiv), Fe(OEP)Cl (23.4 mg, 0.04 mmol, 0.075 equiv), imidazole (1.7 mg, 0.025 mmol, 0.05 equiv), **HEH-1** (387 mg, 1.25 mmol, 2.5 equiv), and standard solvent mixture TAA/PhCN/H_2O (1.66 mL). Reaction was sparged for 5 minutes under positive N_2 pressure, then irradiated at 75% light intensity for 24 hours. After irradiation, the solvent was removed under reduced pressure. The crude residue was loaded onto 25 g Biotage Sf.r reverse phase column and purified with eluents 0%-70% MeCN in H_2O with 0.1% NH4OH. The fractions containing product were concentrated and further purified by preparative HPLC (XBridge BEH C18 OBD column, 20%-80% MeCN in H_2O with 0.1% NH_4OH) to furnish the product as a white solid. Identified as 81% assay yield versus 1 equiv (0.5 mmol mesitylene) internal standard by ^{1}H NMR; see spectral characterization for assay yield.

Diastereomer 1

^{1}H NMR (500 MHz, CDCl3) δ 8.43 (dd, J = 4.7, 1.6 Hz, 1H), 7.72 (dd, *J* = 7.8, 1.6 Hz, 1H), 7.67 – 7.54 (m, 3H), 7.45 (d, *J* = 8.5 Hz, 1H), 7.37 – 7.30 (m, 2H), 7.31 – 7.19 (m, 2H), 7.04 (dd, *J* = 7.7, 4.7 Hz, 1H), 6.66 (d, *J* = 3.6 Hz, 1H), 6.00 (d, *J* = 3.6 Hz, 1H), 4.03 (dd, *J* = 8.1, 4.6 Hz, 1H), 2.10 (dd, *J* = 6.4, 4.6 Hz, 1H), 1.72 (s, 3H), 1.56 (dd, *J* = 8.1, 6.3 Hz, 1H).

^{13}C NMR (126 MHz, $CDCl_3$) δ 149.09, 142.79, 138.45, 133.29, 132.24, 128.71, 127.67 (d, *J* = 5.4 Hz), 127.51 (d, *J* = 7.2 Hz), 127.34, 126.92, 125.76, 125.51, 121.20, 116.03, 98.96, 39.53, 30.06 (d, *J* = 52.4 Hz), 28.78, 26.68, 17.68.

Diastereomer 2

^{1}H NMR (500 MHz, $CDCl_3$) δ 8.46 (dd, *J* = 4.7, 1.6 Hz, 1H), 8.04 (d, *J* = 1.9 Hz, 1H), 8.00 – 7.80 (m, 5H), 7.48 (dqd, *J* = 8.2, 6.9, 1.5 Hz, 2H), 7.28 (d, *J* = 3.5 Hz, 1H), 7.12 (dd, *J* = 7.8, 4.7 Hz, 1H), 6.48 (d, *J* = 3.5 Hz, 1H), 3.82 (dd, *J* = 8.1, 4.6 Hz, 1H), 1.84 (dd, *J* = 8.1, 5.9 Hz, 1H), 1.60 (dd, *J* = 5.9, 4.6 Hz, 2H), 1.20 (s, 3H).

^{13}C NMR (126 MHz, $CDCl_3$) δ 149.57, 143.50, 143.02, 133.66, 132.50, 128.74, 128.60, 128.46, 127.90, 127.78, 127.14, 126.60, 126.13, 125.67, 121.10, 116.25, 99.62, 39.26, 28.42, 20.95, 18.97.

HRMS (ESI-TOF) *m*/*z* calcd. For $C_{21}H_{19}N_2^+$ ($[M+H]^+$) 299.15428, found 299.155591.

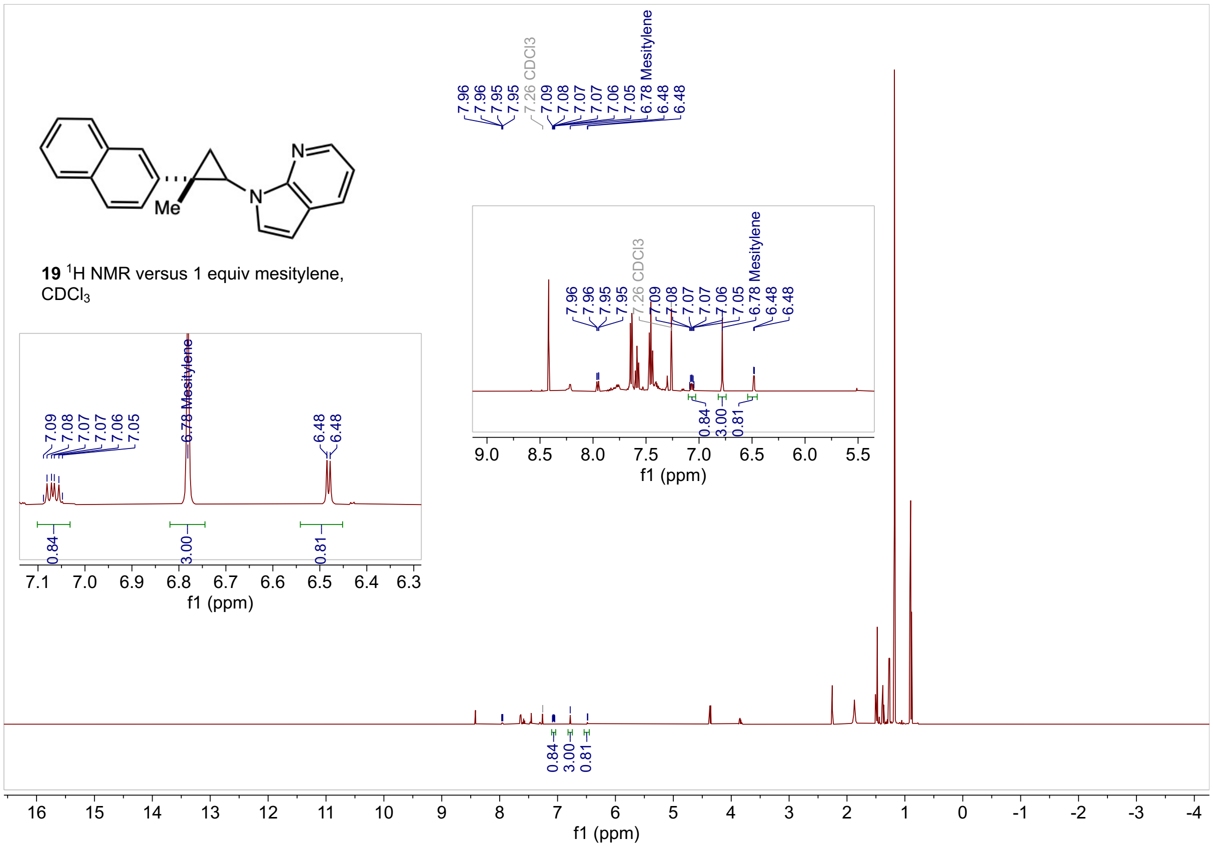

Assignment of d.r. as >20:1 by ^{1}H-NMR of the crude.

***tert*-butyl 1-(4-fluorophenyl)-5-azaspiro[2.3]hexane-5-carboxylate**

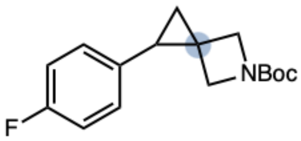

Prepared according to the general procedure **B** with *tert-butyl 3-oxoazetidine-1-carboxylate ketone* (85.6 mg, 0.5 mmol, 1.00 equiv), 4-fluorostyrene (119 μL, 1 mmol, 2.00 equiv), 4CzIPN (7.89 mg, 10 μmol, 0.02 equiv), Fe(OEP)Cl (23.4 mg, 0.04 mmol, 0.075 equiv), imidazole (1.7 mg, 0.025 mmol, 0.05 equiv), **HEH-1** (387 mg, 1.25 mmol, 2.5 equiv), and standard solvent mixture TAA/PhCN/H_2O (1.66 mL). Reaction was sparged for 5 minutes under positive N_2 pressure, then irradiated at 50% light intensity for 24 hours. After irradiation, the solvent was removed under reduced pressure. The crude residue was loaded onto 25 g Biotage Sf.r normal phase column and purified with eluents 0%-50% EtOAc in hexanes. The fractions containing product were concentrated and further purified by preparative HPLC (XBridge BEH C18 OBD column, 20%-100% MeCN in H_2O with 0.1% NH_4OH) to furnish product as a yellow oil (72.3 mg, 0.26 mmol, 52% yield).

^{1}H NMR (500 MHz, $CDCl_3$) δ 6.92 (ddd, *J* = 8.8, 5.4, 2.5 Hz, 4H), 4.09 – 4.00 (m, 2H), 3.80 (dd, *J* = 108.8, 8.5 Hz, 2H), 2.07 (dd, *J* = 9.3, 6.1 Hz, 1H), 1.44 (s, 9H), 1.24 (dd, *J* = 9.3, 6.1 Hz, 1H), 1.03 (t, *J* = 6.2 Hz, 1H).

^{13}C NMR (126 MHz, $CDCl_3$) δ 162.40, 160.46, 156.09, 134.66 (d, *J* = 3.1 Hz), 128.05 (d, *J* = 7.8 Hz), 115.34 (d, *J* = 21.4 Hz), 79.56, 28.46, 25.08, 22.94 (d, *J* = 0.8 Hz), 16.85.

^{19}F NMR (471 MHz, $CDCl_3$) δ -117.13 (td, *J* = 8.7, 4.4 Hz).

HRMS (ESI-TOF) *m*/*z* calcd. For $C_{12}H_{12}FNO_2^+$ ([M+H]$^+$) 222.0925, found 222.0929.

***tert*-butyl (S)-1-(4-fluorophenyl)-1-methyl-5-azaspiro[2.3]hexane-5-carboxylate**

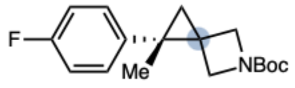

Prepared according to the general procedure **B** with *tert*-butyl 3-oxoazetidine-1-carboxylate ketone precursor (85.6 mg, 0.5 mmol, 1.00 equiv), 1-fluoro-4-(1-methylethenyl)benzene (135 μL, 1.00 mmol, 2.00 equiv), Ir(dF(Me)ppy)$_2$(dtbppy)PF$_6$ (10.1 mg, 10 μmol, 0.02 equiv), Fe(OEP)Cl (23.4 mg, 0.04 mmol, 0.075 equiv), imidazole (1.7 mg, 0.025 mmol, 0.05 equiv), **HEH-1** (387 mg, 1.25 mmol, 2.5 equiv), and standard solvent mixture TAA/PhCN/H_2O (1.66 mL). Reaction was sparged for 5 minutes under positive N_2 pressure, then irradiated at 50% light intensity for 24 hours. After irradiation, the solvent was removed under reduced pressure. The crude residue was loaded onto 25 g Biotage Sf.r normal phase column and purified with eluents 0%-30% EtOAc in hexanes. The fractions containing product were concentrated and further purified by preparative HPLC (XBridge BEH C18 OBD column, 20%-100% MeCN in H_2O with 0.1% NH4OH) to furnish product as a yellow oil (102 mg, 0.35 mmol, 70% yield).

^{1}H NMR (500 MHz, CDCl$_3$) δ 7.13 – 7.09 (m, 2H), 6.98 (t, *J* = 8.6 Hz, 2H), 4.02 (dd, *J* = 111.7, 8.4 Hz, 2H), 3.65 (dd, *J* = 50.3, 8.5 Hz, 2H), 1.43 (d, *J* = 1.9 Hz, 9H), 1.32 (s, 3H), 1.19 (d, *J* = 5.8 Hz, 1H), 0.83 (d, *J* = 5.8 Hz, 1H).

^{13}C NMR (126 MHz, CDCl$_3$) δ 162.34, 160.39, 156.21, 138.14 (d, *J* = 3.1 Hz), 129.05 (d, *J* = 7.9 Hz), 115.30 (d, *J* = 21.3 Hz), 79.52, 54.37 (d, *J* = 77.2 Hz), 28.49, 26.17, 25.72, 22.83, 22.66.

^{19}F NMR (376 MHz, CDCl$_3$) δ -116.87 (ddd, *J* = 14.0, 8.7, 5.3 Hz, 1F).

HRMS (ESI-TOF) *m*/*z* calcd. For $C_{13}H_{15}FNO_2^+$ ([M+H]$^+$) 236.10818, found 236.107873.

(S)-1-(4-fluorophenyl)-1-methyl-5-oxaspiro[2.3]hexane

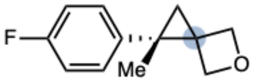

Prepared according to the general procedure **B** with 3-oxetanone precursor (32.06 µL, 0.5 mmol, 1.00 equiv), 1-fluoro-4-(1-methylethenyl)benzene (337 µL, 2.50 mmol, 5.00 equiv), Ir(dF(Me)ppy)$_2$(dtbppy)PF$_6$ (10.1 mg, 10 µmol, 0.02 equiv), Fe(OEP)Cl (23.4 mg, 0.04 mmol, 0.075 equiv), imidazole (1.7 mg, 0.025 mmol, 0.05 equiv), **HEH-1** (387 mg, 1.25 mmol, 2.5 equiv), and standard solvent mixture TAA/PhCN/H_2O (1.66 mL). Reaction was sparged for 5 minutes under positive N_2 pressure, then irradiated at 75% light intensity for 24 hours. After irradiation, the solvent was removed under reduced pressure. The crude residue was loaded onto 25 g Biotage Sf.r normal phase column and purified with eluents 0%-15% EtOAc in hexanes. The fractions containing product were carefully concentrated and further purified by preparative HPLC (XBridge BEH C18 OBD column, (20%-40%) MeCN in H_2O with 0.1% NH_4OH), then extracted into diethyl ether (3 x 100 mL) and carefully evaporated to furnish product as a yellow oil (68 mg, 36 mmol, 71% yield).

^{1}H NMR (400 MHz, CDCl$_3$) δ 7.20 – 6.93 (m, 4H), 4.95 – 4.32 (m, 4H), 1.31 (d, *J* = 1.4 Hz, 3H), 1.02 (dd, *J* = 138.2, 6.1 Hz, 2H).

^{13}C NMR (101 MHz, CDCl$_3$) δ 162.60, 160.17, 138.11 (d, *J* = 3.2 Hz), 129.01 (d, *J* = 7.9 Hz), 115.33 (d, *J* = 21.2 Hz), 76.37, 30.44, 25.78, 22.37, 22.25.

^{19}F NMR (376 MHz, CDCl$_3$) δ -116.95 (q, *J* = 8.5 Hz, 1F).

***tert*-butyl (R)-1-(4-fluorophenyl)-1-methyl-7-azadispiro[2.1.35.13]nonane-7-carboxylate**

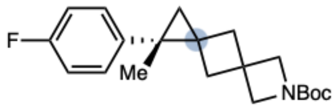

Prepared according to the general procedure **B** with *tert-butyl 6-oxo-2-azaspiro[3.3]heptane-2-carboxylate* precursor (105.6 mg, 0.5 mmol, 1.00 equiv), 1-fluoro-4-(1-methylethenyl)benzene (337 µL, 2.50 mmol, 5.00 equiv), Ir(dF(Me)ppy)$_2$(dtbppy)PF$_6$ (2.13 mg, 10 µmol, 0.02 equiv), Fe(OEP)Cl (23.4 mg, 0.04 mmol, 0.075 equiv), imidazole (1.7 mg, 0.025 mmol, 0.05 equiv), **HEH-1** (387 mg, 1.25 mmol, 2.5 equiv), and standard solvent mixture TAA/PhCN/H_2O (1.66 mL). Reaction was sparged for 5 minutes under positive N_2 pressure, then irradiated at 75% light intensity for 24 hours. After irradiation, the solvent was removed under reduced pressure. The crude residue was loaded onto 25 g Biotage Sf.r normal phase column and purified with eluents 0%-20% EtOAc in hexanes. The fractions containing product were concentrated and further purified by preparative HPLC (XBridge BEH C18 OBD column, 20%-90% MeCN in H_2O with 0.1% NH4OH), then extracted into diethyl ether (250 mL) and carefully evaporated *in vacuo* to furnish product as an orange-yellow oil (99.2 mg, 0.3 mmol, 60% yield).

^{1}H NMR (400 MHz, CDCl$_3$) δ 7.39 – 7.33 (m, 2H), 7.31 – 7.21 (m, 2H), 4.31 – 4.23 (m, 2H), 4.23 – 4.11 (m, 2H), 2.90 – 2.40 (m, 2H), 2.24 (d, *J* = 2.0 Hz, 2H), 1.73 (s, 9H), 1.57 (s, 3H), 1.34 (d, *J* = 5.2 Hz, 1H), 0.96 (d, *J* = 5.2 Hz, 1H).

^{13}C NMR (101 MHz, CDCl$_3$) δ 162.31, 159.89, 156.39, 139.55 (d, *J* = 3.1 Hz), 129.08 (d, *J* = 7.8 Hz), 115.03 (d, *J* = 21.1 Hz), 79.37, 39.23, 38.87, 32.67, 28.52, 26.27, 26.01, 24.89, 22.69.

^{19}F NMR (376 MHz, CDCl$_3$) δ -117.8 (m).

HRMS (ESI-TOF) *m*/*z* calcd. For $C_{15}H_{19}FN^+$ ([M+H]$^+$) 232.14958, found 232.150705.

benzyl 4-((1S,2R)-2-phenylcyclopropyl)piperidine-1-carboxylate

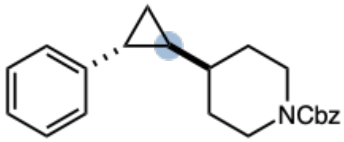

Prepared according to the general procedure **A** with benzyl 4-formylpiperidine-1-carboxylate (123.6 mg, 0.5 mmol, 1.00 equiv), styrene (115 µl, 1 mmol, 2.00 equiv), 4-CzIPN (3.9 mg, 50 µmol, 0.01 equiv), Fe(OEP)Cl (23.4 mg, 0.04 mmol, 0.075 equiv), imidazole (2.6 mg, 0.075 mmol, 0.05 equiv), **HEH-1** (387 mg, 1.25 mmol, 2.5 equiv), and standard solvent mixture TAA/PhCN/H_2O (1.66 mL). Reaction was sparged for 5 minutes under positive N_2 pressure, then irradiated at 20% light intensity for 16 hours. After irradiation, the solvent was removed under reduced pressure. The crude residue was loaded onto 50 g Biotage Sf.r normal phase column and purified with eluents 0%-100% EtOAc in hexanes. The fractions containing product were concentrated and further purified by preparative HPLC (XBridge BEH C18 OBD column, 20%-100% MeCN in H_2O with 0.1% NH_4OH) to furnish product as a colorless oil (68.2 mg, 203. µmol, 40.7% yield, >20:1 d.r.).

^{1}H NMR (500 MHz, $CDCl_3$) δ 7.41 – 7.29 (m, 5H), 7.27 – 7.21 (m, 2H), 7.18 – 7.10 (m, 1H), 7.07 – 7.03 (m, 2H), 5.13 (s, 2H), 4.32 – 3.89 (m, 2H), 2.86 – 2.48 (m, 2H), 1.89 – 1.76 (m, 2H), 1.71 (dt, *J* = 9.0, 4.7 Hz, 1H), 1.40 – 1.30 (m, 2H), 1.14 – 0.87 (m, 3H), 0.82 (d, *J* = 8.5 Hz, 1H).

^{13}C NMR (126 MHz, $CDCl_3$) δ 155.45, 143.55, 137.11, 128.61, 128.41, 128.05, 127.97, 125.92, 125.53, 67.10, 44.31, 44.29, 41.29, 32.81 – 30.87 (broad), 29.00, 22.07, 14.58.

HRMS (ESI-TOF) *m*/*z* calcd. For $C_{22}H_{25}NO_2^+$ ([M+H]$^+$) 336.1958, found 336.1958.

benzyl 4-((1*S*,2*R*)-2-(4-(*tert*-butoxy)phenyl)cyclopropyl)piperidine-1-carboxylate

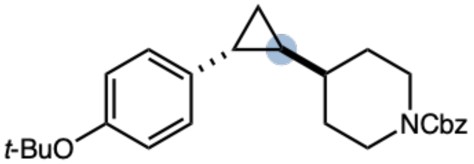

Prepared according to the general procedure **A** with benzyl 4-formylpiperidine-1-carboxylate (123.6 mg, 0.5 mmol, 1.00 equiv), 1-*tert*-butoxy 4-vinyl styrene (190.0 µl, 1.00 mmol, 2.0 equiv.), 4-CzIPN (3.9 mg, 50 µmol, 0.01 equiv), Fe(OEP)Cl (23.4 mg, 0.04 mmol, 0.075 equiv), imidazole (2.6 mg, 0.075 mmol, 0.05 equiv), **HEH-1** (387 mg, 1.25 mmol, 2.5 equiv), and standard solvent mixture TAA/PhCN/H_2O (1.66 mL). Reaction was sparged for 5 minutes under positive N_2 pressure, then irradiated at 20% light intensity for 16 hours. After irradiation, the solvent was removed under reduced pressure. The crude residue was loaded onto 50 g Biotage Sf.r normal phase column and purified with eluents 0%-100% EtOAc in hexanes. The fractions containing product were concentrated and further purified by preparative HPLC (XBridge BEH C18 OBD column, 20%-100% MeCN in H_2O with 0.1% NH4OH) to furnish product as a colorless oil (161.0 mg, 0.395 mmol, 79.0%, >20:1 d.r.).

^{1}H NMR (500 MHz, $CDCl_3$) δ 7.39 – 7.28 (m, 5H), 6.96 – 6.89 (m, 2H), 6.89 – 6.82 (m, 2H), 5.13 (s, 2H), 4.26 – 4.03 (m, 2H), 2.86 – 2.60 (m, 2H), 1.89 – 1.74 (m, 2H), 1.66 (dt, *J* = 9.2, 4.9 Hz, 1H), 1.43 – 1.22 (m, 2H), 1.31 (s, 9H), 0.99 – 0.89 (m, 1H), 0.88 – 0.79 (m, 2H), 0.77 (dtd, *J* = 8.4, 4.3, 1.4 Hz, 1H).

^{13}C NMR (126 MHz, $CDCl_3$) δ 155.47, 153.18, 138.29, 137.14, 128.61, 128.05, 127.97, 126.31, 124.25, 78.29, 67.10, 44.32 (rotamers), 41.25, 31.56, 28.96, 28.64, 21.50, 14.31.

HRMS (ESI-TOF) *m*/*z* calcd. For $C_{26}H_{34}NO_3^+$ ($[M+H]^+$) 408.2533, found 408.2535.

benzyl 4-((1*S*,2*R*)-2-(*p*-tolyl)cyclopropyl)piperidine-1-carboxylate

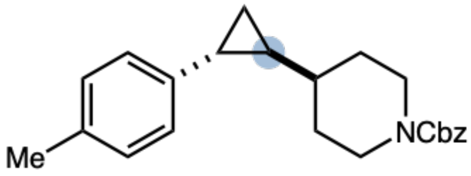

Prepared according to the general procedure **A** with benzyl 4-formylpiperidine-1-carboxylate (123.6 mg, 0.5 mmol, 1.00 equiv), 1-methyl 4-vinyl styrene (132.0 µl, 1.00 mmol, 2.0 equiv.), 4-CzIPN (3.9 mg, 50 µmol, 0.01 equiv), Fe(OEP)Cl (23.4 mg, 0.04 mmol, 0.075 equiv), imidazole (2.6 mg, 0.075 mmol, 0.05 equiv), **HEH-1** (387 mg, 1.25 mmol, 2.5 equiv), and standard solvent mixture TAA/PhCN/H_2O (1.66 mL). Reaction was sparged for 5 minutes under positive N_2 pressure, then irradiated at 20% light intensity for 16 hours. After irradiation, the solvent was removed under reduced pressure. The crude residue was loaded onto 50 g Biotage Sf.r normal phase column and purified with eluents 0%-100% EtOAc in hexanes. The fractions containing product were concentrated and further purified by preparative HPLC (XBridge BEH C18 OBD column, 20%-100% MeCN in H_2O with 0.1% NH4OH) to furnish product as a colorless oil (137.0 mg, 0.356 mmol, 71.3%, >20:1 d.r.).

^{1}H NMR (500 MHz, $CDCl_3$) δ 7.39 (d, *J* = 4.4 Hz, 4H), 7.09 (d, *J* = 7.8 Hz, 2H), 6.98 (d, *J* = 7.7 Hz, 2H), 5.16 (s, 2H), 4.20 (s, 2H), 2.80 (t, *J* = 12.6 Hz, 2H), 2.33 (s, 3H), 1.83 (t, *J* = 13.8 Hz, 2H), 1.70 (dt, *J* = 9.1, 4.8 Hz, 1H), 1.36 (qt, *J* = 11.9, 4.7 Hz, 2H), 1.02 – 0.85 (m, 3H), 0.81 (dd, *J* = 8.5, 2.6 Hz, 1H).

^{13}C NMR (126 MHz, $CDCl_3$) δ 155.47, 140.47, 137.13, 135.06, 129.11, 128.61, 128.06, 127.98, 125.88, 67.10, 44.33, 44.31, 41.32, 31.94, 31.59, 28.78, 21.72, 21.07, 14.35.

HRMS (ESI-TOF) *m*/*z* calcd. For $C_{23}H_{28}NO_2^+$ ([M+H]$^+$) 350.2115, found 350.2118.

benzyl 4-((1*S*,2*R*)-2-(*o*-tolyl)cyclopropyl)piperidine-1-carboxylate

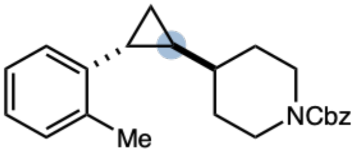

Prepared according to the general procedure **A** with benzyl 4-formylpiperidine-1-carboxylate (123.6 mg, 0.5 mmol, 1.00 equiv), 1-methyl 2-vinyl styrene (129.0 µl, 1.00 mmol, 2.0 equiv.), 4-CzIPN (3.9 mg, 50 µmol, 0.01 equiv), Fe(OEP)Cl (23.4 mg, 0.04 mmol, 0.075 equiv), imidazole (2.6 mg, 0.075 mmol, 0.05 equiv), **HEH-1** (387 mg, 1.25 mmol, 2.5 equiv), and standard solvent mixture TAA/PhCN/H_2O (1.66 mL). Reaction was sparged for 5 minutes under positive N_2 pressure, then irradiated at 20% light intensity for 16 hours. After irradiation, the solvent was removed under reduced pressure. The crude residue was loaded onto 50 g Biotage Sf.r normal phase column and purified with eluents 0%-100% EtOAc in hexanes. The fractions containing product were concentrated and further purified by preparative HPLC (XBridge BEH C18 OBD column, 20%-100% MeCN in H_2O with 0.1% NH4OH) to furnish product as an off-white solid (110.2 mg, 0.315 mmol, 63.1%, >20:1 d.r.).

^{1}H NMR (500 MHz, $CDCl_3$) δ 7.42 – 7.30 (m, 5H), 7.18 – 7.06 (m, 3H), 6.92 (dd, *J* = 7.1, 2.0 Hz, 1H), 5.16 (s, 2H), 4.33 – 4.00 (m, 2H), 3.06 – 2.72 (m, 2H), 2.43 (s, 3H), 1.85 (dd, *J* = 29.5, 13.1 Hz, 2H), 1.75 (dt, *J* = 8.8, 5.1 Hz, 1H), 1.49 – 1.29 (m, 2H), 1.11 – 0.91 (m, 2H), 0.88 – 0.77 (m, 2H).

^{13}C NMR (126 MHz, $CDCl_3$) δ 155.47, 140.94, 137.32, 137.13, 129.80, 128.62, 128.07, 127.99, 126.00, 125.77, 125.37, 67.13, 44.37, 44.34, 41.17, 32.23, 31.63, 27.11, 20.05, 19.78, 13.08.

HRMS (ESI-TOF) *m*/*z* calcd. For $C_{23}H_{28}NO_2^+$ ($[M+H]^+$) 350.2115, found 350.2120.

benzyl 4-((1*S*,2*R*)-2-(4-((*tert*-butoxycarbonyl)amino)phenyl)cyclopropyl)piperidine-1-carboxylate

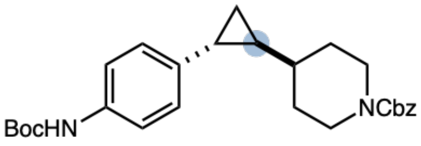

Prepared according to the general procedure **A** with benzyl 4-formylpiperidine-1-carboxylate (123.6 mg, 0.5 mmol, 1.00 equiv), tert-butyl N-(4-vinylphenyl)carbamate (219.3 mg, 1.00 mmol, 2.0 equiv.), 4-CzIPN (3.9 mg, 50 μmol, 0.01 equiv), Fe(OEP)Cl (23.4 mg, 0.04 mmol, 0.075 equiv), imidazole (2.6 mg, 0.075 mmol, 0.05 equiv), **HEH-1** (387 mg, 1.25 mmol, 2.5 equiv), and standard solvent mixture TAA/PhCN/H_2O (1.66 mL). Reaction was sparged for 5 minutes under positive N_2 pressure, then irradiated at 20% light intensity for 16 hours. After irradiation, the solvent was removed under reduced pressure. The crude residue was loaded onto 50 g Biotage Sf.r normal phase column and purified with eluents 0%-100% EtOAc in hexanes. The fractions containing product were concentrated and further purified by preparative HPLC (XBridge BEH C18 OBD column, 20%-100% MeCN in H_2O with 0.1% NH_4OH) to furnish product as an off-white solid (156.8 mg, 0.348 mmol, 69.6%, >20:1 d.r.).

^{1}H NMR (500 MHz, $CDCl_3$) δ 7.40 – 7.27 (m, 5H), 7.22 (d, *J* = 8.1 Hz, 2H), 6.96 (d, *J* = 8.6 Hz, 2H), 6.39 (s, 1H), 5.13 (s, 2H), 4.25 – 3.95 (m, 2H), 2.76 (t, *J* = 12.7 Hz, 2H), 1.78 (t, *J* = 14.7 Hz, 2H), 1.65 (dt, *J* = 9.1, 4.8 Hz, 1H), 1.38 – 1.24 (m, 2H), 0.91 (ddt, *J* = 11.3, 7.7, 3.8 Hz, 1H), 0.86 – 0.79 (m, 2H), 0.77 (dd, *J* = 8.6, 1.0 Hz, 1H).

^{13}C NMR (126 MHz, $CDCl_3$) δ 155.46, 153.01, 138.28, 137.11, 135.94, 128.05, 127.97, 118.94, 80.51, 67.10, 44.31, 44.29, 41.24, 31.55, 28.73, 28.49, 21.51, 14.19.

HRMS (ESI-TOF) *m*/*z* calcd. For $C_{27}H_{35}N_2O_4^+$ ([M+H]$^+$) 451.2592, found 451.2952.

benzyl 4-((1*S*,2*R*)-2-(4-chlorophenyl)cyclopropyl)piperidine-1-carboxylate

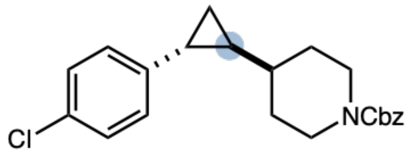

Prepared according to the general procedure **A** with benzyl 4-formylpiperidine-1-carboxylate (123.6 mg, 0.5 mmol, 1.00 equiv), 1-chloro 4-vinyl styrene (120.0 µl, 1.00 mmol, 2.0 equiv.), 4-CzIPN (3.9 mg, 50 µmol, 0.01 equiv), Fe(OEP)Cl (23.4 mg, 0.04 mmol, 0.075 equiv), imidazole (2.6 mg, 0.075 mmol, 0.05 equiv), **HEH-1** (387 mg, 1.25 mmol, 2.5 equiv), and standard solvent mixture TAA/PhCN/H_2O (1.66 mL). Reaction was sparged for 5 minutes under positive N_2 pressure, then irradiated at 20% light intensity for 16 hours. After irradiation, the solvent was removed under reduced pressure. The crude residue was loaded onto 50 g Biotage Sf.r normal phase column and purified with eluents 0%-100% EtOAc in hexanes. The fractions containing product were concentrated and further purified by preparative HPLC (XBridge BEH C18 OBD column, 20%-100% MeCN in H_2O with 0.1% NH_4OH) to furnish product as an off-white solid (137.0 mg, 0.370 mmol, 74.1%, >20:1 d.r.).

^{1}H NMR (500 MHz, $CDCl_3$) δ 7.40 – 7.29 (m, 5H), 7.24 – 7.17 (m, 2H), 6.99 – 6.92 (m, 2H), 5.13 (s, 2H), 4.26 – 4.05 (m, 2H), 2.88 – 2.61 (m, 2H), 1.84 – 1.75 (m, 2H), 1.67 (dt, *J* = 7.1, 5.0 Hz, 1H), 1.39 – 1.26 (m, 2H), 0.99 – 0.88 (m, 1H), 0.89 – 0.74 (m, 3H).

^{13}C NMR (126 MHz, $CDCl_3$) δ 155.45, 142.06, 137.09, 131.12, 128.61, 128.47, 128.07, 127.98, 127.27, 67.13, 44.28 (rotamer), 44.25 (rotamer), 41.18, 31.81- 31.52 (rotamers), 29.23, 21.53, 14.65.

HRMS (ESI-TOF) *m*/*z* calcd. For $C_{22}H_{24}ClNO_2^+$ ([M+H]$^+$) 370.1569, found 370.1572.

benzyl 4-((1*S*,2*R*)-2-(4-bromophenyl)cyclopropyl)piperidine-1-carboxylate

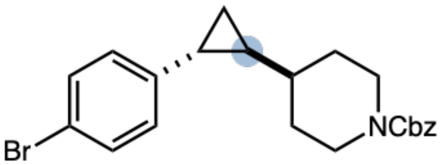

Prepared according to the general procedure **A** with benzyl 4-formylpiperidine-1-carboxylate (123.6 mg, 0.5 mmol, 1.00 equiv), 1-bromo 4-vinyl styrene (131.0 µl, 1.00 mmol, 2.0 equiv.), 4-CzIPN (3.9 mg, 50 µmol, 0.01 equiv), Fe(OEP)Cl (23.4 mg, 0.04 mmol, 0.075 equiv), imidazole (2.6 mg, 0.075 mmol, 0.05 equiv), **HEH-1** (387 mg, 1.25 mmol, 2.5 equiv), and standard solvent mixture TAA/PhCN/H_2O (1.66 mL). Reaction was sparged for 5 minutes under positive N_2 pressure, then irradiated at 20% light intensity for 16 hours. After irradiation, the solvent was removed under reduced pressure. The crude residue was loaded onto 50 g Biotage Sf.r normal phase column and purified with eluents 0%-100% EtOAc in hexanes. The fractions containing product were concentrated and further purified by preparative HPLC (XBridge BEH C18 OBD column, 20%-100% MeCN in H_2O with 0.1% NH_4OH) to furnish product as an off-white solid (137.2 mg, 0.331 mmol, 66.2%, >20:1 d.r.).

^{1}H NMR (500 MHz, $CDCl_3$) δ 7.44 – 7.28 (m, 7H), 6.94 – 6.87 (m, 2H), 5.13 (s, 2H), 4.25 – 4.05 (m, 2H), 3.00 – 2.57 (m, 2H), 1.82 – 1.73 (m, 2H), 1.72 – 1.62 (m, 1H), 1.36 – 1.30 (m, 2H), 0.99 – 0.88 (m, 1H), 0.88 – 0.79 (m, 3H).

^{13}C NMR (126 MHz, $CDCl_3$) δ 155.44, 142.62, 137.08, 131.40, 128.61, 128.08, 127.98, 127.66, 119.02, 67.13, 44.27, 44.24, 41.18, 32.23 – 30.83 (broad), 29.28, 21.60, 14.69.

HRMS (ESI-TOF) *m*/*z* calcd. For $C_{22}H_{25}BrNO_2^+$ ($[M+H]^+$) 414.1063, found 414.1061.

benzyl 4-((1*S*,2*R*)-2-(4-(methoxycarbonyl)phenyl)cyclopropyl)piperidine-1-carboxylate

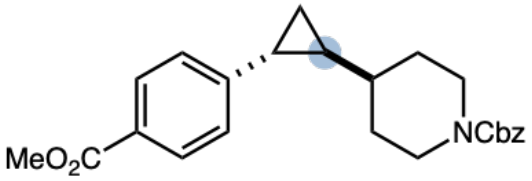

Prepared according to the general procedure **A** with benzyl 4-formylpiperidine-1-carboxylate (123.6 mg, 0.5 mmol, 1.00 equiv), methyl 4-vinylbenzoate (162.2 mg, 1.00 mmol, 2.0 equiv.), 4-CzIPN (3.9 mg, 50 μmol, 0.01 equiv), Fe(OEP)Cl (23.4 mg, 0.04 mmol, 0.075 equiv), imidazole (2.6 mg, 0.075 mmol, 0.05 equiv), **HEH-1** (387 mg, 1.25 mmol, 2.5 equiv), and standard solvent mixture TAA/PhCN/H_2O (1.66 mL). Reaction was sparged for 5 minutes under positive N_2 pressure, then irradiated at 20% light intensity for 16 hours. After irradiation, the solvent was removed under reduced pressure. The crude residue was loaded onto 50 g Biotage Sf.r normal phase column and purified with eluents 0%-100% EtOAc in hexanes. The fractions containing product were concentrated and further purified by preparative HPLC (XBridge BEH C18 OBD column, 20%-100% MeCN in H_2O with 0.1% NH4OH) to furnish product as a yellow oil (131.1 mg, 0.333 mmol, 66.6%, >20:1 d.r.).

^{1}H NMR (500 MHz, CDCl$_3$) δ 7.90 (d, *J* = 8.4 Hz, 2H), 7.06 (d, *J* = 8.5 Hz, 2H), 3.89 (s, 3H), 3.34 (td, *J* = 5.0, 2.2 Hz, 2H), 3.26 (td, *J* = 5.3, 3.4 Hz, 2H), 2.20 – 2.10 (m, 1H), 2.00 – 1.87 (m, 2H), 1.68 (dt, *J* = 9.0, 4.8 Hz, 1H), 1.55 (t, *J* = 5.6 Hz, 2H), 1.49 (ddd, *J* = 12.4, 8.6, 1.6 Hz, 2H), 1.46 – 1.42 (m, 2H) ,1.44 (s, 9H), 1.24 – 1.17 (m, 1H), 0.94 (dt, *J* = 8.6, 5.1 Hz, 1H), 0.88 (dt, *J* = 8.6, 6.0 Hz, 1H).

^{13}C NMR (126 MHz, CDCl$_3$) δ 167.13, 154.99, 149.64, 129.63, 127.10, 125.37, 79.23, 51.94, 40.84, 40.61, 39.26, 36.60, 36.25, 36.18, 33.68, 31.89, 29.97, 28.48, 21.77, 15.33.

HRMS (ESI-TOF) *m*/*z* calcd. For $C_{24}H_{27}NO_4^+$ ([M+H]$^+$) 394.2013, found 394.2018.

benzyl 4-((1*S*,2*R*)-2-(4-(trifluoromethyl)phenyl)cyclopropyl)piperidine-1-carboxylate

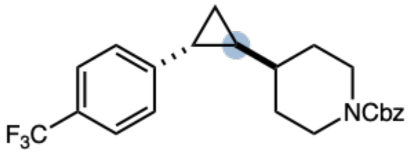

Prepared according to the general procedure **A** with benzyl 4-formylpiperidine-1-carboxylate (123.6 mg, 0.5 mmol, 1.00 equiv), 1-trifluoromethyl-4-vinylbenzene (150.0 µl, 1.00 mmol, 2.0 equiv.), 4-CzIPN (3.9 mg, 50 µmol, 0.01 equiv), Fe(OEP)Cl (23.4 mg, 0.04 mmol, 0.075 equiv), imidazole (2.6 mg, 0.075 mmol, 0.05 equiv), **HEH-1** (387 mg, 1.25 mmol, 2.5 equiv), and standard solvent mixture TAA/PhCN/H_2O (1.66 mL). Reaction was sparged for 5 minutes under positive N_2 pressure, then irradiated at 20% light intensity for 16 hours. After irradiation, the solvent was removed under reduced pressure. The crude residue was loaded onto 50 g Biotage Sf.r normal phase column and purified with eluents 0%-100% EtOAc in hexanes. The fractions containing product were concentrated and further purified by preparative HPLC (XBridge BEH C18 OBD column, 20%-100% MeCN in H_2O with 0.1% NH4OH) to furnish product as a yellow oil (141.2 mg, 0.350 mmol, 70.0% yield, >20:1 d.r.).

^{1}H NMR (500 MHz, Chloroform-*d*) δ 7.51 (d, *J* = 8.1 Hz, 2H), 7.42 – 7.29 (m, 4H), 7.12 (d, *J* = 8.1 Hz, 2H), 5.14 (s, 2H), 4.25 – 4.11 (m, 2H), 2.90 – 2.64 (m, 2H), 1.83 – 1.70 (m, 3H), 1.43 – 1.25 (m, 2H), 1.12 – 0.83 (m, 3H).

^{13}C NMR (126 MHz, Chloroform-*d*) δ 155.42, 147.90 (d, *J* = 1.4 Hz), 137.06, 128.60, 128.02 (d, *J* = 12.2 Hz), 125.94, 125.33 (q, *J* = 3.8 Hz), 123.41 (q, *J* = 271.6 Hz), 67.13, 44.24, 44.20, 41.18, 32.35 – 30.81 (m) (broad due to N-Cbz rotamers), 29.94, 21.98, 15.32.

^{19}F NMR (471 MHz, $CDCl_3$) δ -62.21 (s).

HRMS (ESI-TOF) *m*/*z* calcd. For $C_{23}H_{25}F_3NO_2^+$ ([M+H]$^+$) 404.1832, found 404.1836.

benzyl 4-((1*S*,2*R*)-2-(4-cyanophenyl)cyclopropyl)piperidine-1-carboxylate

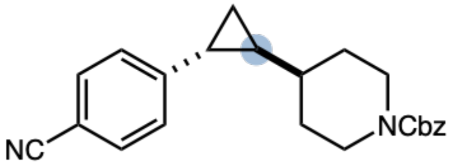

Prepared according to the general procedure **A** with benzyl 4-formylpiperidine-1-carboxylate (123.6 mg, 0.5 mmol, 1.00 equiv), 4-vinylbenzonitrile (130.0 µl, 1.00 mmol, 2.0 equiv.)., 4-CzIPN (3.9 mg, 50 µmol, 0.01 equiv), Fe(OEP)Cl (23.4 mg, 0.04 mmol, 0.075 equiv), imidazole (2.6 mg, 0.075 mmol, 0.05 equiv), **HEH-1** (387 mg, 1.25 mmol, 2.5 equiv), and standard solvent mixture TAA/PhCN/H_2O (1.66 mL). Reaction was sparged for 5 minutes under positive N_2 pressure, then irradiated at 20% light intensity for 16 hours. After irradiation, the solvent was removed under reduced pressure. The crude residue was loaded onto 50 g Biotage Sf.r normal phase column and purified with eluents 0%-100% EtOAc in hexanes. The fractions containing product were concentrated and further purified by preparative HPLC (XBridge BEH C18 OBD column, 20%-100% MeCN in H_2O with 0.1% NH4OH) to furnish product as a yellow oil (103.9 mg, 0.288 mmol, 57.7% yield, >20:1 d.r.).

^{1}H NMR (500 MHz, CDCl$_3$) δ 7.52 (d, *J* = 8.3 Hz, 2H), 7.39 – 7.28 (m, 5H), 7.11 – 7.07 (m, 2H), 5.12 (s, 2H), 4.48 – 3.99 (m, 2H), 2.99 – 2.58 (m, 2H), 1.95 – 1.67 (m, 3H), 1.40 – 1.26 (m, 2H), 1.10 – 0.85 (m, 4H).

^{13}C NMR (126 MHz, CDCl$_3$) δ 155.42, 149.65, 137.03, 132.28, 128.63, 128.11, 128.02, 126.32, 119.30, 109.08, 67.18, 44.22, 44.17, 41.15, 32.28 – 30.97 (m), 30.64, 22.39, 15.92.

HRMS (ESI-TOF) *m*/*z* calcd. For $C_{23}H_{25}N_2O_2^+$ ([M+H]$^+$) 361.1911, found 361.1910.

***tert*-butyl 5-((1*R*,2*S*)-2-(1-((benzyloxy)carbonyl)piperidin-4-yl)cyclopropyl)-1*H*-indazole-1-carboxylate**

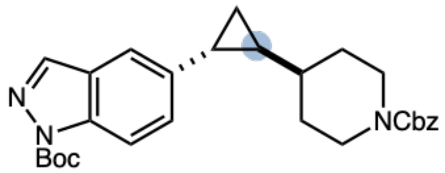

Prepared according to the general procedure **A** with benzyl 4-formylpiperidine-1-carboxylate (123.6 mg, 0.5 mmol, 1.00 equiv), tert-butyl 5-vinylindazole-1-carboxylate (244.3 mg, 1.00 mmol, 2.0 equiv.), 4-CzIPN (3.9 mg, 50 μmol, 0.01 equiv), Fe(OEP)Cl (23.4 mg, 0.04 mmol, 0.075 equiv), imidazole (2.6 mg, 0.075 mmol, 0.05 equiv), **HEH-1** (387 mg, 1.25 mmol, 2.5 equiv), and standard solvent mixture TAA/PhCN/H_2O (1.66 mL). Reaction was sparged for 5 minutes under positive N_2 pressure, then irradiated at 20% light intensity for 16 hours. After irradiation, the solvent was removed under reduced pressure. The crude residue was loaded onto 50 g Biotage Sf.r normal phase column and purified with eluents 0%-100% EtOAc in hexanes. The fractions containing product were concentrated and further purified by preparative HPLC (XBridge BEH C18 OBD column, 20%-100% MeCN in H_2O with 0.1% NH4OH) to furnish product as a white solid (162.4 mg, 0.341 mmol, 68.3% yield, >20:1 d.r.).

^{1}H NMR (500 MHz, $CDCl_3$) δ 8.08 (d, *J* = 0.8 Hz, 1H), 8.04 (d, *J* = 8.6 Hz, 1H), 7.42 – 7.29 (m, 6H), 7.24 (dd, *J* = 8.6, 1.7 Hz, 1H), 5.13 (s, 2H), 4.19 (t, *J* = 11.5 Hz, 2H), 2.78 (td, *J* = 11.0, 2.9 Hz, 2H), 1.90 – 1.77 (m, 3H), 1.72 (s, 8H), 1.42 – 1.28 (m, 2H), 1.07 – 0.80 (m, 3H).

^{13}C NMR (126 MHz, $CDCl_3$) δ 155.45, 149.35, 139.40, 139.16, 138.38, 137.08, 128.61, 128.11, 128.07, 127.98, 126.30, 117.47, 114.45, 84.86, 67.13, 44.29, 44.26, 41.21, 31.95, 31.56, 29.15, 28.33, 21.86, 14.43.

HRMS (ESI-TOF) *m*/*z* calcd. For $C_{28}H_{33}N_3O_4Na^+$ ([M+Na]$^+$) 498.2363, found 498.2369.

***tert*-butyl 2-((1*R*,2*S*)-2-(4-(methoxycarbonyl)phenyl)cyclopropyl)-7-azaspiro[3.5]nonane-7-carboxylate**

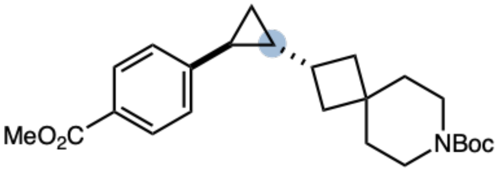

Prepared according to the general procedure **A** with tert-butyl 2-formyl-7-azaspiro[3.5]nonane-7-carboxylate (126.7 mg, 0.500 mmol, 1.0 equiv.), methyl 4-vinylbenzoate (162.2 mg, 1.00 mmol, 2.0 equiv.), 4-CzIPN (3.9 mg, 50 μmol, 0.01 equiv), Fe(OEP)Cl (23.4 mg, 0.04 mmol, 0.075 equiv), imidazole (2.6 mg, 0.075 mmol, 0.05 equiv), **HEH-1** (387 mg, 1.25 mmol, 2.5 equiv), and standard solvent mixture TAA/PhCN/H_2O (1.66 mL). Reaction was sparged for 5 minutes under positive N_2 pressure, then irradiated at 20% light intensity for 16 hours. After irradiation, the solvent was removed under reduced pressure. The crude residue was loaded onto 50 g Biotage Sf.r normal phase column and purified with eluents 0%-100% EtOAc in hexanes. The fractions containing product were concentrated and further purified by preparative HPLC (XBridge BEH C18 OBD column, 20%-100% MeCN in H_2O with 0.1% NH4OH) to furnish product as a yellow oil (163.8 mg, 0.410 mmol, 82.0%, >20:1 d.r.).

^{1}H NMR (500 MHz, CDCl$_3$) δ 7.90 (d, *J* = 8.4 Hz, 2H), 7.06 (d, *J* = 8.5 Hz, 2H), 3.89 (s, 3H), 3.34 (td, *J* = 5.0, 2.2 Hz, 2H), 3.26 (td, *J* = 5.3, 3.4 Hz, 2H), 2.20 – 2.10 (m, 1H), 2.00 – 1.87 (m, 2H), 1.68 (dt, *J* = 9.0, 4.8 Hz, 1H), 1.55 (t, *J* = 5.6 Hz, 2H), 1.49 (ddd, *J* = 12.4, 8.6, 1.6 Hz, 2H), 1.46 – 1.42 (m, 2H) ,1.44 (s, 9H), 1.24 – 1.17 (m, 1H), 0.94 (dt, *J* = 8.6, 5.1 Hz, 1H), 0.88 (dt, *J* = 8.6, 6.0 Hz, 1H).

^{13}C NMR (126 MHz, CDCl$_3$) δ 167.13, 154.99, 149.64, 129.63, 127.10, 125.37, 79.23, 51.94, 40.84, 40.61, 39.26, 36.60, 36.25, 36.18, 33.68, 31.89, 29.97, 28.48, 21.77, 15.33.

HRMS (ESI-TOF) *m*/*z* calcd. For $C_{24}H_{33}NO_4Na^+$ ([M+Na]$^+$) 422.2302, found 422.2306.

tert-butyl((1_R_,3_r_)-3-((1_R_,2_S_)-2-(2,3-dihydrobenzofuran-4 yl)cyclopropyl)cyclobutyl)carbamate

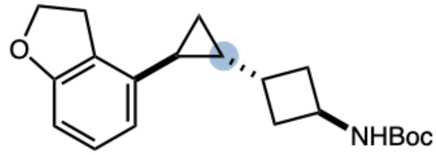

Prepared according to the general procedure **A** with _tert_-butyl ((1_r_,3_r_)-3-formylcyclobutyl)carbamate (99.6 mg, 0.500 mmol, 1.0 equiv.), 4-vinyl-2,3-dihydrobenzofuran (135.4 µl, 1.00 mmol, 2.0 equiv.), 4-CzIPN (3.9 mg, 50 µmol, 0.01 equiv), Fe(OEP)Cl (23.4 mg, 0.04 mmol, 0.075 equiv), imidazole (2.6 mg, 0.075 mmol, 0.05 equiv), **HEH-1** (387 mg, 1.25 mmol, 2.5 equiv), and standard solvent mixture TAA/PhCN/H_2O (1.66 mL). Reaction was sparged for 5 minutes under positive N_2 pressure, then irradiated at 20% light intensity for 16 hours. After irradiation, the solvent was removed under reduced pressure. The crude residue was loaded onto 50 g Biotage Sf.r normal phase column and purified with eluents 0%-100% EtOAc in hexanes. The fractions containing product were concentrated and further purified by preparative HPLC (XBridge BEH C18 OBD column, 20%-100% MeCN in H_2O with 0.1% NH4OH) to furnish product as a clear oil (104.0 mg, 0.316 mmol, 63.1% yield, >20:1 d.r.).

^{1}H NMR (500 MHz, Chloroform-_d_) δ 7.02 (dd, _J_ = 7.9, 7.9 Hz, 1H), 6.60 (d, _J_ = 7.8 Hz, 1H), 6.38 (d, _J_ = 7.7 Hz, 1H), 4.81 – 4.65 (m, 1H), 4.58 (ddd, _J_ = 9.9, 8.4, 1.8 Hz, 2H), 4.28 – 4.14 (m, 1H), 3.22 (t, _J_ = 8.8 Hz, 2H), 2.23 – 2.14 (m, 2H), 2.10 – 1.95 (m, 3H), 1.55 (dt, _J_ = 8.7, 4.9 Hz, 1H), 1.44 (s, 9H), 1.30 – 1.21 (m, 1H), 0.94 (dt, _J_ = 8.5, 5.1 Hz, 1H), 0.78 (dt, _J_ = 8.7, 5.2 Hz, 1H).

^{13}C NMR (126 MHz, Chloroform-_d_) δ 159.57, 155.02, 139.81, 128.10, 125.80, 115.89, 106.48, 79.29, 70.99, 44.24, 35.26, 34.83, 32.44, 28.73, 28.43, 26.69, 19.48, 13.31. (HSQC shows that the cyclobutane carbons are not equivalent).

HRMS (ESI-TOF) _m/z_ calcd. For $C_{20}H_{27}NO_3Na^+$ ([M+Na]$^+$) 352.1883, found 352.1881.

tert-butyl((1S,3s)-3-((1R,2S)-2-(2,3-dihydrobenzofuran-4-yl)cyclopropyl)cyclobutyl)carbamate

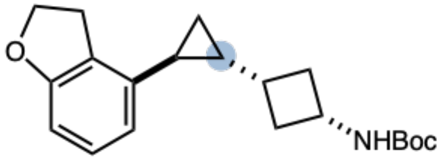

Prepared according to the general procedure **A** with *tert*-butyl ((1*r*,3*s*)-3-formylcyclobutyl)carbamate (99.6 mg, 0.500 mmol, 1.0 equiv.), 4-vinyl-2,3-dihydrobenzofuran (135.4 μl, 1.00 mmol, 2.0 equiv.), 4-CzIPN (3.9 mg, 50 μmol, 0.01 equiv), Fe(OEP)Cl (23.4 mg, 0.04 mmol, 0.075 equiv), imidazole (2.6 mg, 0.075 mmol, 0.05 equiv), **HEH-1** (387 mg, 1.25 mmol, 2.5 equiv), and standard solvent mixture TAA/PhCN/H_2O (1.66 mL). Reaction was sparged for 5 minutes under positive N_2 pressure, then irradiated at 20% light intensity for 16 hours. After irradiation, the solvent was removed under reduced pressure. The crude residue was loaded onto 50 g Biotage Sf.r normal phase column and purified with eluents 0%-100% EtOAc in hexanes. The fractions containing product were concentrated and further purified by preparative HPLC (XBridge BEH C18 OBD column, 20%-100% MeCN with 0.1% NH4OH) to furnish product as a clear oil (104.4 mg, 0.317 mmol, 63.4% yield, >20:1 d.r.).

^{1}H NMR (500 MHz, Chloroform-*d*) δ 7.01 (dd, *J* = 7.8, 7.8 Hz, 1H), 6.60 (d, *J* = 7.9 Hz, 1H), 6.35 (d, *J* = 7.8 Hz, 1H), 4.68 – 4.61 (m, 1H), 4.58 (t, *J* = 9.1 Hz, 2H), 4.02 – 3.80 (m, 1H), 3.21 (td, *J* = 8.6, 3.0 Hz, 2H), 2.52 – 2.40 (m, 2H), 1.84 – 1.72 (m, 1H), 1.58 – 1.47 (m, 3H), 1.44 (s, 8H), 1.10 – 1.01 (m, 1H), 0.90 (dt, *J* = 8.5, 5.1 Hz, 1H), 0.76 (dt, *J* = 8.7, 5.3 Hz, 1H).

^{13}C NMR (126 MHz, Chloroform-*d*) δ 159.69, 155.03, 139.90, 128.20, 126.01, 116.00, 106.62, 79.39, 71.12, 42.01, 36.75, 36.31, 31.68, 28.81, 28.55, 26.84, 19.23, 12.91. (HSQC shows that the cyclobutane carbons are not equivalent).

HRMS (ESI-TOF) *m*/*z* calcd. For $C_{20}H_{27}NO_3Na^+$ ([M+Na]$^+$) 352.1883, found 352.1881.

benzyl 4-((1*S*,2*S*)-2-((*E*)-4-chlorostyryl)cyclopropyl)piperidine-1-carboxylate

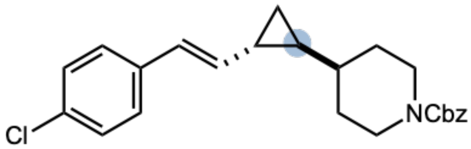

Prepared according to the general procedure **A** with benzyl 4-formylpiperidine-1-carboxylate (123.6 mg, 0.5 mmol, 1.00 equiv), 1-[(1E)-buta-1,3-dienyl]-4-chloro-benzene (164.3 mg, 1.00 mmol, 2.0 equiv.), 4-CzIPN (3.9 mg, 50 μmol, 0.01 equiv), Fe(OEP)Cl (23.4 mg, 0.04 mmol, 0.075 equiv), imidazole (2.6 mg, 0.075 mmol, 0.05 equiv), **HEH-1** (387 mg, 1.25 mmol, 2.5 equiv), and standard solvent mixture TAA/PhCN/H_2O (1.66 mL). Reaction was sparged for 5 minutes under positive N_2 pressure, then irradiated at 20% light intensity for 16 hours. After irradiation, the solvent was removed under reduced pressure. The crude residue was loaded onto 50 g Biotage Sf.r normal phase column and purified with eluents 0%-100% EtOAc in hexanes. The fractions containing product were concentrated and further purified by preparative HPLC (XBridge BEH C18 OBD column, 20%-100% MeCN with 0.1% NH_4OH) to furnish product as a clear oil (108.2 mg, 0.273 mmol, 54.7% yield, 10:1 dr).

^{1}H NMR (500 MHz, $CDCl_3$) δ 7.39 – 7.29 (m, 5H), 7.23 (d, *J* = 8.8 Hz, 2H), 7.20 (d, *J* = 8.7 Hz, 2H), *minor* 6.46 (d, *J* = 15.7 Hz, 0.11H), *major* 6.35 (d, *J* = 15.7 Hz, 0.88H), *minor* 5.93 (dd, *J* = 15.7, 9.1 Hz, 0.11H), 5.74 (dd, *J* = 15.7, 8.8 Hz, 0.89H), 5.13 (s, 2H), 4.23 – 3.98 (m, 2H), 3.15 – 2.67 (m, 2H), 1.86 – 1.68 (m, 2H), 1.40 – 1.23 (m, 3H), 0.96 – 0.82 (m, 1H), 0.82 – 0.72 (m, 1H), 0.72 – 0.66 (m, 2H).
Unassigned diastereomers

^{13}C NMR (126 MHz, $CDCl_3$) δ 155.46, 137.11, 136.29, 134.93, 132.20, 128.80, 128.75, 128.62, 128.08, 127.99, 126.97, 126.87, 126.14, 67.13, 44.29, 44.25, 40.79, 32.01-31.35 (rotamers), 21.09, 13.25.

HRMS (ESI-TOF) *m*/*z* calcd. For $C_{14}H_{26}ClNO_2^+$ ([M+H]$^+$) 396.1725, found 396.1731.

***tert*-butyl 4-((2-(2-oxopyrrolidin-1-yl)cyclopropyl)methyl)piperidine-1-carboxylate**

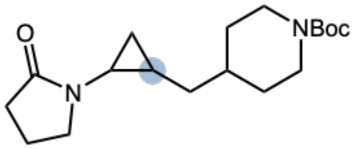

Prepared according to the general procedure **A** with *tert*-butyl tert-butyl 4-(2-oxoethyl)piperidine-1-carboxylate (113.7 mg, 0.500 mmol, 1.0 equiv.), 1-vinylpyrrolidin-2-one (106.8 ml, 1.00 mmol, 2.0 equiv.), [Ir[dF(CF_3)ppy]$_2$(dtbbpy)]PF_6 (5.1 mg, 0.05 mmol, 0.01 equiv.), Fe(OEP)Cl (23.4 mg, 0.04 mmol, 0.075 equiv), imidazole (2.6 mg, 0.075 mmol, 0.05 equiv), **HEH-1** (387 mg, 1.25 mmol, 2.5 equiv), and standard solvent mixture TAA/PhCN/H_2O (1.66 mL). Reaction was sparged for 5 minutes under positive N_2 pressure, then irradiated at 20% light intensity for 16 hours. After irradiation, the solvent was removed under reduced pressure. The crude residue was loaded onto 50 g Biotage Sf.r normal phase column and purified with eluents 0%-100% EtOAc in hexanes. The fractions containing product were concentrated and further purified by preparative HPLC (XBridge BEH C18 OBD column, 20%-100% MeCN with 0.1% NH_4OH) to furnish product as a clear oil (6:1 ratio of diastereomers, 125.5 mg, 0.389 mmol, 77.8%, 6:1 d.r.).

Mixture of Diastereomers

^{1}H NMR (500 MHz, $CDCl_3$) δ 4.11 – 4.03 (m, 2H), 3.41 – 3.25 (m, 2H), 2.79 – 2.54 (m, 2H), 2.47 – 2.26 (m, 3H), 2.08 – 1.89 (m, 2H), 1.79 – 1.69 (m, 2H), 1.64 – 1.54 (m, 1H), 1.45 (s, 9H), 1.31 – 1.23 (m, 1H), 1.20 – 1.01 (m, 4H), 0.88 – 0.72 (m, 1H), 0.57 – 0.38 (m, 1H).

^{13}C NMR (126 MHz, $CDCl_3$) δ (177.17) 176.17, 155.04, (79.33) 79.30, (49.32) 47.65, 44.18, 39.86, (36.59), 36.27, (35.00), (32.49) 32.39, (32.34) 32.21, 32.00, 31.94 (31.91), (29.89) 28.62, (18.57) 18.21, (16.48) 15.85, 12.28 (10.78).

HRMS (ESI-TOF) *m*/*z* calcd. For $C_{18}H_{30}N_2O_3Na^+$ ([M+Na]$^+$) 345.2148, found 345.2151.

benzyl 4-(2-(2-oxopyrrolidin-1-yl)cyclopropyl)piperidine-1-carboxylate

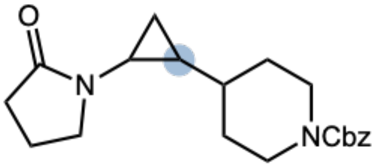

Prepared according to the general procedure **A** with benzyl 4-formylpiperidine-1-carboxylate (123.6 mg, 0.500 mmol, 1.0 equiv.), 1-vinylpyrrolidin-2-one (106.8 ml, 1.00 mmol, 2.0 equiv.), [Ir[dF(CF_3)ppy]$_2$(dtbbpy)]PF_6 (5.1 mg, 0.05 mmol, 0.01 equiv.), Fe(OEP)Cl (23.4 mg, 0.04 mmol, 0.075 equiv), imidazole (2.6 mg, 0.075 mmol, 0.05 equiv), **HEH-1** (387 mg, 1.25 mmol, 2.5 equiv), and standard solvent mixture TAA/PhCN/H_2O (1.66 mL). Reaction was sparged for 5 minutes under positive N_2 pressure, then irradiated at 50% light intensity for 16 hours. After irradiation, the solvent was removed under reduced pressure. The crude residue was loaded onto 50 g Biotage Sf.r normal phase column and purified with eluents 0%-100% EtOAc in hexanes. The fractions containing product were concentrated and further purified by preparative HPLC (XBridge BEH C18 OBD column, 20%-100% MeCN with 0.1% NH4OH) to furnish product as a clear oil (115.4 mg, 0.337 mmol, 67.4%, 20:1 d.r.).

^{1}H NMR (500 MHz, $CDCl_3$) δ 7.38 – 7.27 (m, 5H), 5.12 (s, 2H), 4.15 (d, *J* = 13.3 Hz, 2H), 3.27 (t, *J* = 7.1 Hz, 2H), 2.92 – 2.69 (m, 2H), 2.44 – 2.31 (m, 3H), 2.00 – 1.93 (m, 2H), 1.92 – 1.87 (m, 1H), 1.48 – 1.25 (m, 3H), 0.92 – 0.79 (m, 3H), 0.65 (d, *J* = 7.6 Hz, 1H).

^{13}C NMR (126 MHz, $CDCl_3$) δ 176.41, 155.43, 137.08, 128.61, 128.06, 127.97, 67.12, 47.82, 44.13, 39.37, 31.90, 31.61, 31.11, 30.81, 24.24, 18.14, 10.95.

HRMS (ESI-TOF) *m*/*z* calcd. For $C_{20}H_{27}N_2O_3^+$ ([M+H]$^+$) 343.2016, found 343.2019.

tert-butyl 1-(2-oxopyrrolidin-1-yl)-5-azaspiro[2.3]hexane-5-carboxylate

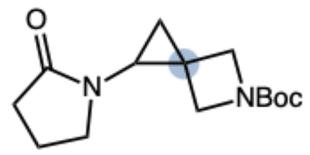

Prepared according to the general procedure **B** with *tert*-butyl 3-oxoazetidine-1-carboxylate ketone precursor (86 mg, 0.5 mmol, 1.00 equiv), 1-vinyl-2-pyrrolidinone (107 μL, 1.00 mmol, 2.00 equiv), Ir(dF(Me)ppy)$_2$(dtbppy)PF$_6$ (2.13 mg, 10 μmol, 0.02 equiv), Fe(OEP)Cl (23.4 mg, 0.04 mmol, 0.075 equiv), imidazole (1.7 mg, 0.025 mmol, 0.05 equiv), **HEH-1** (387 mg, 1.25 mmol, 2.5 equiv), and standard solvent mixture TAA/PhCN/H_2O (1.66 mL). Reaction was sparged for 5 minutes under positive N_2 pressure, then irradiated at 75% light intensity for 24 hours. After irradiation, the solvent was removed under reduced pressure. The crude residue was loaded onto 25 g Biotage Sf.r reverse phase column and purified with eluents 0%-50% MeCN in H_2O with 0.1% NH4OH to furnish product as a yellow oil (83 mg, 0.31 mmol, 62% yield).

^{1}H NMR (400 MHz, $CDCl_3$) δ 4.04 (d, *J* = 8.0 Hz, 1H), 3.91 – 3.70 (m, 3H), 3.26 (qd, *J* = 8.1, 1.4 Hz, 1H), 3.10 (ddd, *J* = 9.5, 7.9, 5.5 Hz, 1H), 2.67 (dd, *J* = 8.5, 5.2 Hz, 1H), 2.48 – 2.25 (m, 2H), 2.13 – 1.72 (m, 2H), 1.36 (d, *J* = 1.7 Hz, 9H), 1.12 – 0.89 (m, 2H).

^{13}C NMR (101 MHz, $CDCl_3$) δ 176.69, 155.91, 79.40, 47.37, 33.37, 31.49, 28.35, 20.38, 18.28, 14.01.

HRMS (ESI-TOF) *m*/*z* calcd. For $C_{14}H_{22}N_2O_3^+$ ([M+H]$^+$) 267.1703, found 267.1708

tert-butyl4-((2-(bis(_tert_-butoxycarbonyl)amino)cyclopropyl)methyl)piperidine-1-carboxylate

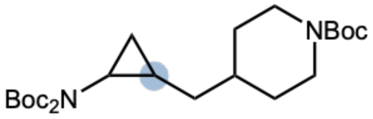

Prepared according to the general procedure **A** with tert-butyl 4-(2-oxoethyl)piperidine-1-carboxylate (113.7 mg, 0.500 mmol, 1.0 equiv.), tert-butyl (tert-butoxycarbonyl)(vinyl)carbamate (243.3 mg, 1.00 mmol, 2.0 equiv.), [Ir[dF(Me)ppy]$_2$(dtbbpy)]PF$_6$ (5.1 mg, 0.05 mmol, 0.01 equiv.), Fe(OEP)Cl (23.4 mg, 0.04 mmol, 0.075 equiv), imidazole (2.6 mg, 0.075 mmol, 0.05 equiv), **HEH-1** (387 mg, 1.25 mmol, 2.5 equiv), and standard solvent mixture TAA/PhCN/H_2O (1.66 mL). Reaction was sparged for 5 minutes under positive N_2 pressure, then irradiated at 50% light intensity for 16 hours. After irradiation, the solvent was removed under reduced pressure. The crude residue was loaded onto 50 g Biotage Sf.r normal phase column and purified with eluents 0%-100% EtOAc in hexanes. The fractions containing product were concentrated and further purified by preparative HPLC (XBridge BEH C18 OBD column, 20%-100% MeCN with 0.1% NH4OH) to furnish product as a clear oil (103.0 mg, 0.227 mmol, 45.3%, 2.5:1 d.r.).

Isolated as mixture of diastereomers.
^{1}H NMR (500 MHz, CDCl$_3$) δ 4.20 – 3.85 (m, 2H), 2.77 – 2.23 (m, 2H), 1.81 – 1.55 (m, 3H), 1.50 (s, 18H), 1.45 (s, 9H), 1.18 – 0.93 (m, 3H), 0.91 – 0.75 (m, 1H), *minor* 0.69 (q, J = 6.5 Hz, 0.28H), *major* 0.33 (q, J = 5.4 Hz, 0.71H).

Isolated as mixture of diastereomers.
^{13}C NMR (126 MHz, CDCl$_3$) δ 155.04, 153.78, 153.33, 82.32, 82.22, 79.33, 44.96 – 43.64 (m), 39.36, 36.43, 36.04, 34.48, 34.33, 32.66, 32.53, 32.33, 32.19, 28.62, 28.25, 28.16, 20.82, 17.58, 16.78, 14.59.

HRMS (ESI-TOF) *m*/*z* calcd. For $C_{24}H_{42}N_2O_6Na^+$ ([M+Na]$^+$) 561.2935, found 561.2952

***tert*-butyl 4-((2-(*N*-methylacetamido)cyclopropyl)methyl)piperidine-1-carboxylate**

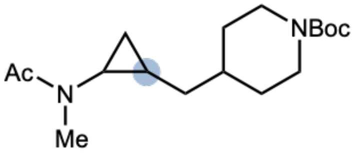

Prepared according to the general procedure **A** with tert-butyl 4-(2-oxoethyl)piperidine-1-carboxylate (113.7 mg, 0.5 mmol, 1.00 equiv), *N*-methyl-*N*-vinylacetamide (99 mg, 1.00 mmol, 2.00 equiv), [Ir[dF(Me)ppy]$_2$(dtbbpy)]PF$_6$ (5.1 mg, 0.05 mmol, 0.01 equiv.), Fe(OEP)Cl (23.4 mg, 0.04 mmol, 0.075 equiv), imidazole (2.6 mg, 0.075 mmol, 0.05 equiv), **HEH-1** (387 mg, 1.25 mmol, 2.5 equiv), and standard solvent mixture TAA/PhCN/H_2O (1.67 mL). Reaction was sparged for 5 minutes under positive N_2 pressure, then irradiated at 50% light intensity for 16 hours. After irradiation, the solvent was removed under reduced pressure. The crude residue was loaded onto 50 g Biotage Sf.r normal phase column and purified with eluents 0%-20% EtOAc in hexanes. The fractions containing product were concentrated and further purified by preparative HPLC (XBridge BEH C18 OBD column, 20%-90% MeCN in H_2O with 0.1% NH4OH), then extracted into diethyl ether (250 mL) and carefully evaporated *in vacuo* to furnish product as an orange-yellow oil (97.7 mg, 314.7 μmol, 62% yield, 5:1 dr).

^{1}H NMR (500 MHz, CDCl3) Major: δ 4.08 (d, J = 13.3 Hz, 2H), 2.87 (s, 3H), 2.72 – 2.62 (m, 2H), 2.37 (dt, J = 7.1, 3.5, 3.5 Hz, 1H), 2.15 (d, J = 6.1 Hz, 3H), 2.05 – 1.98 (m, 1H), 1.76 – 1.67 (m, 2H), 1.64 – 1.50 (m, 2H), 1.44 (s, 9H), 1.11 (dq, J = 30.8, 11.3, 9.6, 9.6 Hz, 3H), 0.94 – 0.81 (m, 1H), 0.67 (q, J = 6.3, 6.2, 6.2 Hz, 1H).
Minor: δ 4.08 (s, 2H), 2.88 (s, 3H), 2.78 (td, J = 7.4, 7.4, 4.5 Hz, 1H), 2.66 (s, 2H), 2.15 (s, 3H), 1.44 (s, 13H), 1.10 (ddd, J = 29.4, 13.7, 9.0 Hz, 4H), 0.73 (ddd, J = 13.4, 10.2, 5.6 Hz, 1H), 0.48 (p, J = 6.0, 6.0, 5.3, 5.3 Hz, 1H).
^{13}C NMR (126 MHz, CDCl3) Major: δ 173.4, 154.9, 79.3, 43.9, 39.2, 38.3, 36.1, 33.8, 32.2, 30.9, 28.5, 22.5, 20.2, 16.4.
Minor: δ 174.0, 173.0, 154.9, 79.3, 79.2, 37.5, 36.5, 36.4, 35.3, 34.8, 34.7, 34.7, 32.5, 32.2, 31.9, 30.9, 29.3, 28.5, 22.7, 22.1, 17.8, 17.3, 13.6, 11.7.
HRMS (ESI-TOF) m/z calcd. For $C_{17}H_{30}N_2O_3Na+$ ([M+Na]+) 333.2148, found 333.2154

***tert*-butyl 4-(((1S,2S)-2-acetamido-2-(methoxycarbonyl)cyclopropyl)methyl)piperidine-1-carboxylate**

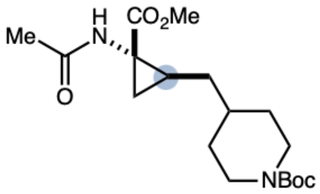

Prepared according to the general procedure **E** with *tert*-butyl 4-(2-oxoethyl)piperidine-1-carboxylate aldehyde precursor (114 mg, 0.5 mmol, 1.00 equiv), methyl 2-acetamidoacrylate (250 mg, 1.75 mmol, 3.50 equiv), 4ClCzIPN (10.6 mg, 10 μmol, 0.02 equiv), Fe(PMP)Cl (31.3 mg, 0.04 mmol, 0.075 equiv), imidazole (1.7 mg, 0.025 mmol, 0.05 equiv), **HEH-1** (387 mg, 1.25 mmol, 2.5 equiv), and standard solvent mixture TAA/PhCN/H_2O (1.66 mL). Reaction was sparged for 5 minutes under positive N_2 pressure, then irradiated at 75% light intensity for 24 hours. After irradiation, the solvent was removed under reduced pressure. The crude residue was loaded onto 25 g Biotage Sf.r reverse phase column and purified with eluents 0%-40% MeCN in H_2O with 0.1% NH4OH, then the fractions containing product were concentrated and further purified by preparative HPLC (XBridge BEH C18 OBD column, 0%-40% MeCN in H_2O with 0.1% NH4OH) to furnish product as a yellow oil (144 mg, 0.41 mmol, 81% yield (9.2:1 d.r.)).

^{1}H NMR (500 MHz, $CDCl_3$) δ 5.98 (s, 1H), 4.07 (d, *J* = 13.2 Hz, 2H), 3.66 (s, 3H), 2.71 – 2.62 (m, 2H), 2.01 (s, 3H), 1.85 – 1.63 (m, 5H), 1.53 (ddtd, *J* = 14.7, 11.1, 7.0, 3.4 Hz, 1H), 1.43 (s, 10H), 1.18 – 0.96 (m, 3H), 0.84 (dd, *J* = 7.3, 4.5 Hz, 1H).

^{13}C NMR (126 MHz, $CDCl_3$) δ 175.82, 173.19, 173.01, 171.79, 154.96, 79.53, 79.44, 52.62, 44.04, 37.36, 36.26, 35.01, 32.25, 28.56, 26.13, 23.28, 23.23.

HRMS (ESI-TOF) *m*/*z* calcd. For $C_{18}H_{30}N_2O_5^+$ ([M+Na]$^+$) 377.2047, found 377.2056

benzyl 4-((2S)-2-acetamido-2-(methoxycarbonyl)cyclopropyl)piperidine-1-carboxylate

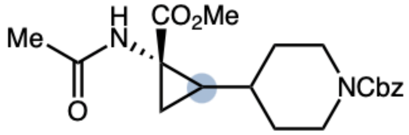

Prepared according to the general procedure **E** with N-Benzylpiperidine-4-carboxaldehyde (102 mg, 0.5 mmol, 1.00 equiv), methyl 2-acetamidoacrylate (250 mg, 1.75 mmol, 3.50 equiv), 4ClCzIPN (10.6 mg, 10 μmol, 0.02 equiv), Fe(PMP)Cl (31.3 mg, 0.04 mmol, 0.075 equiv), imidazole (1.7 mg, 0.025 mmol, 0.05 equiv), **HEH-1** (387 mg, 1.25 mmol, 2.5 equiv), and standard solvent mixture TAA/PhCN/H_2O (1.66 mL). Reaction was sparged for 5 minutes under positive N_2 pressure, then irradiated at 75% light intensity for 24 hours. After irradiation, the solvent was removed under reduced pressure. The crude residue was loaded onto 25 g Biotage Sf.r reverse phase column and purified with eluents 0%-50% MeCN in H_2O with 0.1% NH4OH, then the fractions containing product were concentrated and further purified by preparative HPLC (XBridge BEH C18 OBD column, 0%-40% MeCN in H_2O with 0.1% NH4OH) to furnish product as a white solid (112 mg, 0.3 mmol, 60% yield, (7:1 d.r.)).

^{1}H NMR (500 MHz, $CDCl_3$) δ 7.38 – 7.27 (m, 5H), 6.18 (s, 0H), 5.11 (s, 2H), 4.20 – 4.05 (m, 2H), 3.67 (d, *J* = 16.0 Hz, 3H), 2.76 (q, *J* = 13.3 Hz, 2H), 2.52 – 2.38 (m, 0H), 2.01 (d, *J* = 4.7 Hz, 2H), 1.94 (s, 0H), 1.87 – 1.78 (m, 1H), 1.75 – 1.29 (m, 6H), 1.24 (s, 1H), 1.15 – 1.03 (m, 1H), 0.92 (dd, *J* = 7.7, 5.0 Hz, 1H).

^{13}C NMR (126 MHz, $CDCl_3$) δ 202.81, 172.67, 171.96, 155.33, 155.25, 136.92, 136.76, 128.56, 128.17 – 127.97, 127.90, 67.07, 52.63, 44.08, 43.84, 38.08, 35.44, 33.00, 32.34, 31.56, 23.33.

HRMS (ESI-TOF) *m*/*z* calcd. For $C_{20}H_{26}N_2O_5^+$ ([M+H]$^+$) 375.1915, found 375.1925

5-(tert-butyl) 1-methyl (R)-1-acetamido-5-azaspiro[2.3]hexane-1,5-dicarboxylate

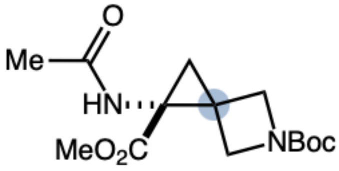

Prepared according to the general procedure **E** with *tert-butyl 3-oxoazetidine-1-carboxylate ketone precursor* (85.6 mg, 0.5 mmol, 1.00 equiv), methyl 2-acetamidoacrylate (143 mg, 1.00 mmol, 2.00 equiv), 4ClCzIPN (10.6 mg, 10 μmol, 0.02 equiv), Fe(PMP)Cl (31.3 mg, 0.04 mmol, 0.075 equiv), imidazole (1.7 mg, 0.025 mmol, 0.05 equiv), **HEH-1** (387 mg, 1.25 mmol, 2.5 equiv), and standard solvent mixture TAA/PhCN/H_2O (1.66 mL). Reaction was sparged for 5 minutes under positive N_2 pressure, then irradiated at 50% light intensity for 24 hours. After irradiation, the solvent was removed under reduced pressure. The crude residue was loaded onto 25 g Biotage Sf.r reverse phase column and purified with eluents 0%-50% MeCN in H_2O with 0.1% NH_4OH, then the fractions containing product were concentrated and further purified by preparative HPLC (XBridge BEH C18 OBD column, 0%-40% MeCN in H_2O with 0.1% NH4OH) to furnish product as a yellow oil (110 mg, 0.37 mmol, 74% yield).

^{1}H NMR (500 MHz, $CDCl_3$) δ 7.13 (s, 1H), 3.96 (t, *J* = 11.0 Hz, 3H), 3.78 (d, *J* = 8.7 Hz, 1H), 3.60 (s, 3H), 2.09 (s, 2H), 1.93 (s, 3H), 1.74 (d, *J* = 6.2 Hz, 1H), 1.35 (s, 9H), 1.27 (d, *J* = 6.2 Hz, 1H), 1.17 (s, 1H).

^{13}C NMR (126 MHz, $CDCl_3$) δ ^{13}C NMR (126 MHz, $CDCl_3$) δ 210.90, 171.92, 170.56, 155.96, 79.75, 52.42, 38.72, 29.83, 28.30, 25.16, 22.79.

HRMS (ESI-TOF) *m*/*z* calcd. For $C_{14}H_{22}N_2O_3^+$ ([M+Na]$^+$) 321.1421, found 321.1435

methyl 1-acetamido-5-oxaspiro[2.3]hexane-1-carboxylate

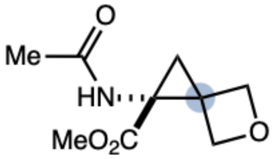

Prepared according to the general procedure **E** with 3-oxetanone (32 µL, 0.5 mmol, 1.00 equiv), methyl 2-acetamidoacrylate (143 mg, 1.00 mmol, 2.00 equiv), 4ClCzIPN (10.6 mg, 10 µmol, 0.02 equiv), Fe(PMP)Cl (31.3 mg, 0.04 mmol, 0.075 equiv), imidazole (1.7 mg, 0.025 mmol, 0.05 equiv), **HEH-1** (387 mg, 1.25 mmol, 2.5 equiv), and standard solvent mixture TAA/PhCN/H_2O (1.66 mL). Reaction was sparged for 5 minutes under positive N_2 pressure, then irradiated at 75% light intensity for 24 hours. After irradiation, the solvent was removed under reduced pressure. The crude residue was loaded onto 25 g Biotage Sf.r reverse phase column and purified with eluents 0%-10% MeCN in H_2O with 0.1% NH_4OH, then the fractions containing product were extracted (100 mL x 4) then additionally the aqueous fractions were concentrated and collected to furnish product as a clear yellow oil (77 mg, 0.385 mmol, 77% yield). *Nota bene: product is extremely water soluble. Retain and evaporate all aqueous fractions to ensure full recovery of the title compound.*

^{1}H NMR (500 MHz, $CDCl_3$) δ 6.15 (s, 1H), 4.77 (s, 2H), 4.73 – 4.62 (m, 2H), 3.69 (s, 3H), 2.02 (s, 3H), 1.86 (d, J = 6.3 Hz, 1H), 1.53 – 1.32 (m, 1H), 1.26 (d, J = 17.1 Hz, 1H).

^{13}C NMR (126 MHz, $CDCl_3$) δ 171.65, 170.43, 76.27, 74.75, 52.69, 38.90, 34.26, 24.61, 23.20.

HRMS (ESI-TOF) *m/z* calcd. For $C_9H_{14}NO_4^+$ ([M+Na]$^+$) 222.0737, found 222.0743

tert-butyl (S)-4-(spiro[2.4]heptan-1-ylmethyl)piperidine-1-carboxylate

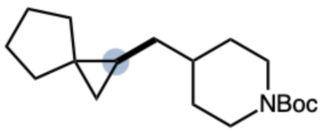

Prepared according to the general procedure **C** with N-Boc-4-piperidineacetaldehyde precursor (114 mg, 0.5 mmol, 1.00 equiv), methylenecyclopentane (263 μL, 2.50 mmol, 5.00 equiv), Ir(dF(Me)ppy)$_2$(dtbppy)PF$_6$ (10.1 mg, 10 μmol, 0.02 equiv), Fe(OEP)Cl (23.4 mg, 0.04 mmol, 0.075 equiv), NBocHisOH (6.4 mg, 0.025 mmol, 0.05 equiv), **HEH-1** (387 mg, 1.25 mmol, 2.5 equiv), and standard solvent mixture TAA/PhCN/H_2O (1.66 mL). Reaction was sparged for 5 minutes under positive N_2 pressure, then irradiated at 75% light intensity for 24 hours. After irradiation, the solvent was removed under reduced pressure. The crude residue was loaded onto 25 g Biotage Sf.r phase column and purified with eluents 0%-20% EtOAc in hexanes, then the fractions containing product were further purified by preparative HPLC (XBridge BEH C18 OBD column, 0%-100% MeCN in H_2O with 0.1% NH_4OH) to furnish product as a yellow oil (78.2 mg, 0.265 μmol, 53% yield).

^{1}H NMR (500 MHz, CDCl$_3$) δ 4.06 (d, *J* = 13.0 Hz, 2H), 2.67 (t, *J* = 12.5 Hz, 2H), 1.81 – 1.54 (m, 6H), 1.44 (s, 12H), 1.36 – 1.25 (m, 2H), 1.20 – 0.95 (m, 3H), 0.73 – 0.50 (m, 2H), 0.07 – -0.04 (m, 1H).

^{13}C NMR (126 MHz, CDCl$_3$) δ 155.01, 79.18, 44.25, 37.75, 37.61, 37.02, 32.36 (apparent d, *J* = 5.0 Hz), 30.50, 28.58, 26.59 (apparent d, *J* = 4.0 Hz, *apparent doublet due to rotamers*), 26.20, 21.00, 20.48.

HRMS (ESI-TOF) *m*/*z* calcd. For $C_{14}H_{23}NO_2^+$ ([M+H]$^+$) 238.1802, found 238.1812.

tert-butyl 1-((1-(tert-butoxycarbonyl)piperidin-4-yl)methyl)-8-azadispiro[2.1.55.13]undecane-8-carboxylate

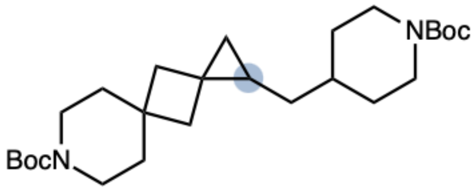

Prepared according to the general procedure **C** with N-Boc-4-piperidineacetaldehyde precursor (114 mg, 0.5 mmol, 1.00 equiv), *tert*-butyl 2-methylidene-7-azaspiro[3.5]nonane-7-carboxylate (593 mg, 2.50 mmol, 5.00 equiv), Ir(dF(Me)ppy)$_2$(dtbppy)PF$_6$ (10.1 mg, 10 μmol, 0.02 equiv), Fe(OEP)Cl (23.4 mg, 0.04 mmol, 0.075 equiv), NBocHisOH (6.4 mg, 0.025 mmol, 0.05 equiv), **HEH-1** (387 mg, 1.25 mmol, 2.5 equiv), and standard solvent mixture TAA/PhCN/H_2O (1.66 mL). Reaction was sparged for 5 minutes under positive N_2 pressure, then irradiated at 75% light intensity for 24 hours. After irradiation, the solvent was removed under reduced pressure. The crude residue was loaded onto 25 g Biotage Sf.r phase column and purified with eluents 0%-50% EtOAc in hexanes, then the fractions containing product were further purified by preparative HPLC (XBridge BEH C18 OBD column, 0%-100% MeCN in H_2O with 0.1% NH_4OH) to furnish product as a yellow oil (116 mg, 0.258 mmol, 52% yield).

^{1}H NMR (500 MHz, CDCl$_3$) δ 4.07 (s, 2H), 3.30 (dq, J = 13.5, 6.7 Hz, 4H), 2.67 (d, J = 13.2 Hz, 2H), 2.16 (s, trace acetone), 1.84 (d, J = 11.3 Hz, 1H), 1.80 (s, 2H), 1.74 – 1.67 (m, 3H), 1.65 (s, 0H), 1.61 (t, J = 5.6 Hz, 2H), 1.57 – 1.51 (m, 2H), 1.44 (d, J = 2.0 Hz, 17H), 1.37 – 1.25 (m, 1H), 1.10 (dtdd, J = 14.9, 10.9, 6.9, 4.0 Hz, 2H), 0.80 (dt, J = 14.0, 7.2 Hz, 1H), 0.60 – 0.49 (m, 2H), 0.01 (d, J = 3.4 Hz, 1H).

^{13}C NMR (126 MHz, CDCl$_3$) δ 207.04, 155.13, 155.05, 79.30, 79.28, 41.53, 37.78, 37.14, 36.68, 34.58, 32.12, 28.60 (d, J = 1.3 Hz), 19.27, 18.98, 18.46.

HRMS (ESI-TOF) *m/z* calcd. For $C_{17}H_{29}N_2O_2^+$ ([M+H]$^+$) 293.22238, found 293.22194.

***tert*-butyl 2-azadispiro[3.0.45.14]decane-2-carboxylate**

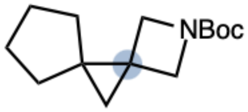

Prepared according to the general procedure **E** with *tert*-butyl 3-oxoazetidine-1-carboxylate ketone precursor (85.6 mg, 0.5 mmol, 1.00 equiv), methylenecyclopentane (263 μL, 2.50 mmol, 5.00 equiv), 4ClCzIPN (10.6 mg, 10 μmol, 0.02 equiv), Fe(PMP)Cl (31.3 mg, 0.04 mmol, 0.075 equiv), imidazole (2.6 mg, 0.04 mmol, 0.075 equiv), **HEH-1** (387 mg, 1.25 mmol, 2.5 equiv), and standard solvent mixture TAA/PhCN/H_2O (1.66 mL). Reaction was sparged for 5 minutes under positive N_2 pressure, then irradiated at 75% light intensity for 24 hours. After irradiation, the solvent was removed under reduced pressure. The crude residue was loaded onto 25 g Biotage Sf.r phase column and purified with eluents 0%-20% EtOAc in hexanes, then the fractions containing product were further purified by preparative HPLC (XBridge BEH C18 OBD column, 0%-100% MeCN in H_2O with 0.1% NH_4OH) to furnish product as a yellow oil (59.1 mg, 0.25 mmol, 50% yield).

^{1}H NMR (500 MHz, $CDCl_3$) δ 3.84 (dd, *J* = 51.1, 8.2 Hz, 4H), 1.71 (m, 4H), 1.44 (s, 11H), 1.36 (dt, *J* = 12.5, 6.5 Hz, 2H), 0.56 (s, 2H).

^{13}C NMR (126 MHz, $CDCl_3$) δ 156.41, 79.36, 54.40, 31.88, 28.56, 28.39, 26.68, 23.45, 21.79.

HRMS (ESI-TOF) *m*/*z* calcd. For $C_{10}H_{15}NO_2^+$ ([M+H]$^+$) 182.1176, found 182.1178 need HRMS.

di-tert-butyl 2,9-diazatrispiro[3.0.1.37.15.14]dodecane-2,9-dicarboxylate

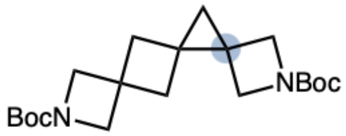

Prepared according to the general procedure **B** with *tert*-butyl 3-oxoazetidine-1-carboxylate ketone precursor (85.6 mg, 0.5 mmol, 1.00 equiv), tert-butyl 6-methylene-2-azaspiro[3.3]heptane-2-carboxylate (523 mg, 2.50 mmol, 5.00 equiv), Ir(dF(Me)ppy)$_2$(dtbppy)PF$_6$ (10.1 mg, 10 µmol, 0.02 equiv), Fe(OEP)Cl (23.4 mg, 0.04 mmol, 0.075 equiv), imidazole (1.7 mg, 25 µmol, 0.05 equiv), **HEH-1** (387 mg, 1.25 mmol, 2.5 equiv), and standard solvent mixture TAA/PhCN/H_2O (1.66 mL). Reaction was sparged for 5 minutes under positive N_2 pressure, then irradiated at 75% light intensity for 24 hours. After irradiation, the solvent was removed under reduced pressure. The crude residue was loaded onto 25 g Biotage Sf.r phase column and purified with eluents 0%-20% EtOAc in hexanes, then the fractions containing product were further purified by preparative HPLC (XBridge BEH C18 OBD column, 0%-100% MeCN in H_2O with 0.1% NH_4OH) to furnish product as a yellow oil (126 mg, 0.35 mmol, 69% yield).

^{1}H NMR (500 MHz, CDCl$_3$) δ 3.95 (d, *J* = 16.8 Hz, 4H), 3.86 – 3.71 (m, 4H), 2.17 (dd, *J* = 73.4, 12.2 Hz, 4H), 1.43 (d, *J* = 9.7 Hz, 18H), 0.58 (s, 2H).

^{13}C NMR (126 MHz, CDCl$_3$) δ 156.37, 156.33, 128.82, 127.23, 127.15, 79.62, 79.49, 37.83, 33.02, 28.51 (d, *J* = 1.8 Hz), 21.62, 21.05, 19.86.

HRMS (ESI-TOF) *m*/*z* calcd. For $C_{20}H_{32}N_2O_4^+$ ([M+Na]$^+$) 387.2254, found 387.2271

di-tert-butyl 2,9-diazatrispiro[3.0.1.37.25.14]tridecane-2,9-dicarboxylate

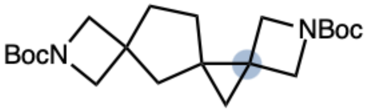

Prepared according to the general procedure **B** with *tert*-butyl 3-oxoazetidine-1-carboxylate ketone precursor (85.6 mg, 0.5 mmol, 1.00 equiv), *tert*-butyl 6-methylene-2-azaspiro[3.4]octane-2-carboxylate (558 mg, 2.50 mmol, 5.00 equiv), $Ir(dF(Me)ppy)_2(dtbppy)PF_6$ (10.1 mg, 10 μmol, 0.02 equiv), Fe(OEP)Cl (23.4 mg, 0.04 mmol, 0.075 equiv), imidazole (1.7 mg, 25 μmol, 0.05 equiv), **HEH-1** (387 mg, 1.25 mmol, 2.5 equiv), and standard solvent mixture TAA/PhCN/H_2O (1.66 mL). Reaction was sparged for 5 minutes under positive N_2 pressure, then irradiated at 75% light intensity for 24 hours. After irradiation, the solvent was removed under reduced pressure. The crude residue was loaded onto 25 g Biotage Sf.r phase column and purified with eluents 0%-20% EtOAc in hexanes, then the fractions containing product were further purified by preparative HPLC (XBridge BEH C18 OBD column, 0%-100% MeCN in H_2O with 0.1% NH_4OH) to furnish product as a yellow oil (119 mg, 0.32 mmol, 63% yield).

^{1}H NMR (500 MHz, $CDCl_3$) δ 3.99 – 3.73 (m, 8H), 2.07 – 1.85 (m, 2H), 1.81 – 1.74 (m, 1H), 1.66 – 1.53 (m, 2H), 1.40 (d, *J* = 5.9 Hz, 19H), 0.70 – 0.42 (m, 2H).

^{13}C NMR (126 MHz, $CDCl_3$) δ 156.55, 156.35, 79.57, 79.44, 60.62, 59.80, 54.61, 54.05, 43.30, 41.94, 38.40, 30.24, 28.53, 26.95, 23.04, 22.42.

HRMS (ESI-TOF) *m*/*z* calcd. For $C_{21}H_{34}N_2O_4^+$ ([M+Na]$^+$) 401.2411, found 401.2429

tert-butyl-1-(4-chlorophenyl)-1-(4-((1-isopropoxy-2-methyl-1-oxopropan-2-yl)oxy)phenyl)-5-azaspiro[2.3]hexane-5-carboxylate

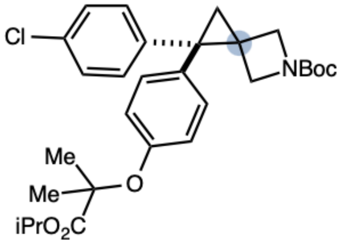

Prepared according to the general procedure **B** with *tert*-butyl 3-oxoazetidine-1-carboxylate ketone precursor (85.6 mg, 0.5 mmol, 1.00 equiv), isopropyl 2-(4-(1-(4-chlorophenyl)vinyl)phenoxy)-2-methylpropanoate (359 mg, 1.00 mmol, 2.00 equiv), Ir(dF(Me)ppy)$_2$(dtbppy)PF$_6$ (10.1 mg, 10 μmol, 0.02 equiv), Fe(OEP)Cl (23.4 mg, 0.04 mmol, 0.075 equiv), imidazole (1.7 mg, 25 μmol, 0.05 equiv), **HEH-1** (387 mg, 1.25 mmol, 2.5 equiv), and standard solvent mixture TAA/PhCN/H_2O (1.66 mL). Reaction was sparged for 5 minutes under positive N_2 pressure, then irradiated at 75% light intensity for 24 hours. After irradiation, the solvent was removed under reduced pressure. The crude residue was loaded onto 25 g Biotage Sf.r phase column and purified with eluents 0%-40% EtOAc in hexanes, then the fractions containing product were further purified by preparative HPLC (XBridge BEH C18 OBD column, 0%-70% MeCN in H_2O with 0.1% NH_4OH) to furnish product as a yellow oil (200 mg, 0.39 mmol, 78% yield).

^{1}H NMR (500 MHz, $CDCl_3$) δ 7.25 – 7.19 (m, 2H), 7.02 (dd, *J* = 17.6, 8.2 Hz, 4H), 6.86 – 6.74 (m, 2H), 5.05 (p, *J* = 6.3 Hz, 1H), 4.04 – 3.89 (m, 2H), 3.81 (dd, *J* = 20.2, 8.8 Hz, 2H), 1.67 – 1.48 (m, 9H), 1.42 (s, 9H), 1.18 (d, *J* = 6.4 Hz, 6H).

^{13}C NMR (126 MHz, $CDCl_3$) δ 173.62, 156.06, 154.40, 140.28, 133.99, 132.27, 130.02, 129.64, 128.62, 118.91, 79.61, 79.15, 68.95, 35.15, 28.45, 28.00, 25.46, 24.99, 21.60, 21.59.

HRMS (ESI-TOF) *m/z* calcd. For $C_{29}H_{36}ClNO_5^+$ ([M+Na]$^+$) 536.2174, found 536.2192

***tert*-butyl 1-(4-((*S*)-2-((*tert*-butoxycarbonyl)amino)-3-methoxy-3-oxopropyl)phenyl)-5-azaspiro[2.3]hexane-5-carboxylate**

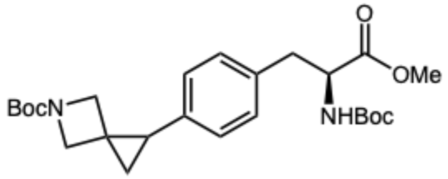

Prepared according to the general procedure **A** with *tert*-butyl 3-oxoazetidine-1-carboxylate ketone precursor (85.6 mg, 0.5 mmol, 1.00 equiv), methyl 2-(tert-butoxycarbonylamino)-3-(4-vinylphenyl)propanoate (305.4 mg, 1.00 mmol, 2.00 equiv), 4CzIPN (7.9 mg, 10 μmol, 0.02 equiv), Fe(OEP)Cl (23.4 mg, 0.04 mmol, 0.075 equiv), imidazole (1.7 mg, 0.025 mmol, 0.05 equiv), **HEH-1** (387 mg, 1.25 mmol, 2.5 equiv), and standard solvent mixture TAA/PhCN/H_2O (1.66 mL). Reaction was sparged for 5 minutes under positive N_2 pressure, then irradiated at 50% light intensity for 24 hours. After irradiation, the solvent was removed under reduced pressure. The crude residue was loaded onto 25 g Biotage Sf.r normal phase column and purified with eluents 0%-30% EtOAc in hexanes. The fractions containing product were concentrated and further purified by preparative HPLC (XBridge BEH C18 OBD column, 20%-100% MeCN in H_2O with 0.1% NH4OH) to furnish product as a white solid (153.2 mg, 0.333 mmol, 67% yield).

^{1}H NMR (500 MHz, $CDCl_3$) δ 7.04 (d, J = 7.8 Hz, 2H), 6.85 (d, J = 7.8 Hz, 2H), 4.95 (d, J = 8.3 Hz, 1H), 4.56 (q, J = 6.7 Hz, 1H), 4.05 (d, J = 8.2 Hz, 1H), 4.00 (d, J = 8.1 Hz, 1H), 3.92 (dd, J = 8.4, 1.8 Hz, 1H), 3.75 – 3.67 (m, 4H), 3.07 (dd, J = 13.7, 5.4 Hz, 1H), 3.01 (dd, J = 13.9, 6.2 Hz, 1H), 2.04 (dd, J = 9.2, 6.1 Hz, 1H), 1.42 (s, 18H), 1.22 (dd, J = 9.2, 6.1 Hz, 1H), 1.03 (td, J = 6.1, 2.3 Hz, 1H).

^{13}C NMR (126 MHz, $CDCl_3$) δ 172.47, 156.13, 155.21, 137.99, 133.77, 129.48, 126.76, 80.07, 79.56, 57.24, 54.54, 54.03, 52.36, 52.35, 38.07, 28.53, 25.59, 23.21, 17.24.

HRMS (ESI-TOF) *m*/*z* calcd. For $C_{25}H_{36}N_2O_6Na^+$ ([M+Na]$^+$) 483.2456, found 483.2474.

tert-butyl 5-(5-(tert-butoxycarbonyl)-5-azaspiro[2.3]hexan-1-yl)-1H-indazole-1-carboxylate

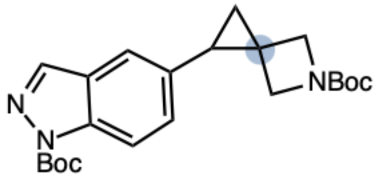

Prepared according to the general procedure **B** with *tert*-butyl 3-oxoazetidine-1-carboxylate ketone precursor (85.6 mg, 0.5 mmol, 1.00 equiv), *tert*-butyl 5-vinyl-1H-indazole-1-carboxylate (244 mg, 1.00 mmol, 2.00 equiv), 4CzIPN (7.9 mg, 10 μmol, 0.02 equiv), Fe(OEP)Cl (23.4 mg, 0.04 mmol, 0.075 equiv), imidazole (2.6 mg, 0.04 mmol, 0.075 equiv), **HEH-1** (387 mg, 1.25 mmol, 2.5 equiv), and standard solvent mixture TAA/PhCN/H_2O (1.66 mL). Reaction was sparged for 5 minutes under positive N_2 pressure, then irradiated at 75% light intensity for 24 hours. After irradiation, the solvent was removed under reduced pressure. The crude residue was loaded onto 25 g Biotage Sf.r phase column and purified with eluents 0%-100% EtOAc in hexanes, then the fractions containing product were further purified by preparative HPLC (XBridge BEH C18 OBD column, 0%-60% MeCN in H_2O with 0.1% NH_4OH) to furnish product as a yellow oil (101 mg, 0.252 mmol, 51% yield).

^{1}H NMR (500 MHz, $CDCl_3$) δ 8.08 (d, *J* = 9.1 Hz, 2H), 7.28 (d, *J* = 1.6 Hz, 1H), 7.15 (dd, *J* = 8.7, 1.7 Hz, 1H), 4.18 – 3.99 (m, 2H), 3.91 (d, *J* = 8.5 Hz, 1H), 3.64 (d, *J* = 8.4 Hz, 1H), 2.25 – 2.10 (m, 1H), 1.71 (s, 9H), 1.40 (s, 9H), 1.34 – 1.16 (m, 2H), 1.13 (t, *J* = 6.2 Hz, 1H).

^{13}C NMR (126 MHz, $CDCl_3$) δ 156.07, 149.25, 139.30, 138.56, 134.72, 128.46, 126.31, 118.32, 114.59, 84.95, 79.58, 28.46, 28.27, 25.57, 23.22, 16.86.

HRMS (ESI-TOF) *m/z* calcd. For $C_{22}H_{29}N_3O_4^+$ ([M+Na]$^+$) 422.205, found 422.206361

***tert*-butyl 5'-(5-((benzyloxy)carbonyl)-5-azaspiro[2.3]hexan-1-yl)-3'*H*-spiro[azetidine-3,1'-isobenzofuran]-1-carboxylate**

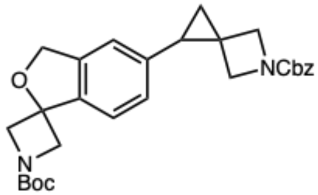

Prepared according to the general procedure **B** with benzyl 3-oxoazetidine-1-carboxylate (102.6 mg, 0.5 mmol, 1.00 equiv), *tert*-butyl 5'-vinyl-3'*H*-spiro[azetidine-3,1'-isobenzofuran]-1-carboxylate (305.4 mg, 1.00 mmol, 2.00 equiv), 4CzIPN (7.9 mg, 10 μmol, 0.02 equiv), Fe(OEP)Cl (23.4 mg, 0.04 mmol, 0.075 equiv), imidazole (1.7 mg, 0.025 mmol, 0.05 equiv), **HEH-1** (387 mg, 1.25 mmol, 2.5 equiv), and standard solvent mixture TAA/PhCN/H_2O (1.66 mL). Reaction was sparged for 5 minutes under positive N_2 pressure, then irradiated at 50% light intensity for 24 hours. After irradiation, the solvent was removed under reduced pressure. The crude residue was loaded onto 50 g Biotage Sf.r normal phase column and purified with eluents 0%-30% EtOAc in hexanes. The fractions containing product were concentrated and further purified by preparative HPLC (XBridge BEH C18 OBD column, 20%-100% MeCN in H_2O with 0.1% NH_4OH) to furnish product as a white solid (153.2 mg, 0.333 mmol, 57.9% yield).

^{1}H NMR (500 MHz, $CDCl_3$) δ 7.44 – 7.27 (m, 7H), 6.94 (d, J = 7.9 Hz, 1H), 6.76 (s, 1H), 5.08 (s, 1H), 5.06 (s, 2H), 4.29 (dd, J = 9.4, 1.1 Hz, 2H), 4.19 – 4.08 (m, 4H), 4.01 (d, J = 8.5 Hz, 1H), 3.77 (d, J = 8.5 Hz, 1H), 2.13 (dd, J = 9.2, 6.3 Hz, 1H), 1.48 (s, 9H), 1.28 (dd, J = 9.3, 6.3 Hz, 1H), 1.10 (t, J = 6.2 Hz, 1H).

^{13}C NMR (126 MHz, $CDCl_3$) δ 156.61, 156.24, 139.73, 139.28, 136.71, 128.59, 128.19, 128.16, 126.90, 120.83, 118.69, 82.64, 79.93, 72.76, 66.86, 31.05, 28.52, 25.81, 23.66, 17.11.

HRMS (ESI-TOF) *m*/*z* calcd. For $C_{28}H_{32}N_2O_5Na^+$ ([M+Na]$^+$) 499.2203, found 499.2214.

Methyl 2-((*tert*-butoxycarbonyl)amino)-3-(4-((2*r*)-2-((1*r*,4*r*)-4-((*tert*-butoxycarbonyl)amino)cyclohexyl)cyclopropyl)phenyl)propanoate

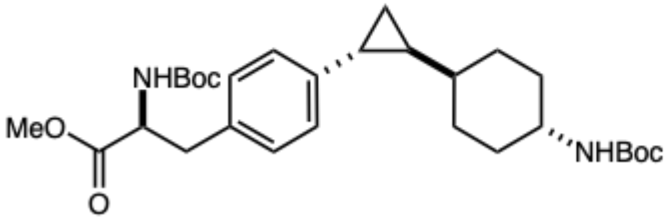

Prepared according to the general procedure **A** with tert-butyl N-(4-formylcyclohexyl)carbamate (113.7 mg, 0.5 mmol, 1.00 equiv), methyl 2-(tert-butoxycarbonylamino)-3-(4-vinylphenyl)propanoate (305.4 mg, 1 mmol, 2.00 equiv), 4-CzIPN (3.9 mg, 50 µmol, 0.01 equiv), Fe(OEP)Cl (23.4 mg, 0.04 mmol, 0.075 equiv), imidazole (2.6 mg, 0.075 mmol, 0.05 equiv), **HEH-1** (387 mg, 1.25 mmol, 2.5 equiv), and standard solvent mixture TAA/PhCN/H_2O (1.66 mL). Reaction was sparged for 5 minutes under positive N_2 pressure, then irradiated at 20% light intensity for 16 hours. After irradiation, the solvent was removed under reduced pressure. The crude residue was loaded onto 50 g Biotage Sf.r normal phase column and purified with eluents 0%-100% EtOAc in hexanes. The fractions containing product were concentrated and further purified by preparative HPLC (XBridge BEH C18 OBD column, 20%-100% MeCN in H_2O with 0.1% NH4OH) to furnish product white solid (141.6 mg, 274.0 µmol, 54.8% yield, >20:1 dr).

^{1}H NMR (500 MHz, $CDCl_3$) δ 6.98 (d, *J* = 8.0 Hz, 2H), 6.94 (d, *J* = 8.2 Hz, 2H), 4.93 (d, *J* = 8.4 Hz, 1H), 4.55 (q, *J* = 6.6 Hz, 1H), 4.37 – 4.33 (m, 1H), 3.72 (s, 3H), 3.41 – 3.37 (m, 1H), 3.06 (dd, *J* = 13.9, 5.7 Hz, 1H), 2.99 (dd, *J* = 14.0, 6.2 Hz, 1H), 2.07 – 1.96 (m, 2H), 1.95 – 1.79 (m, 2H), 1.61 (dt, *J* = 8.2, 4.9 Hz, 1H), 1.44 (s, 9H), 1.41 (s, 9H), 1.28 – 1.15 (m, 2H), 1.10 – 0.98 (m, 2H), 0.86 – 0.72 (m, 3H), 0.72 – 0.62 (m, 1H).

^{13}C NMR (126 MHz, $CDCl_3$) δ 172.56, 155.39,155.24, 142.73, 132.86, 129.32, 126.05, 80.02,79.20, 54.53, 52.34, 49.97, 42.44, 37.97, 33.59, 33.56, 31.70, 31.29, 29.68, 28.59, 28.45, 21.90, 14.87.

HRMS (ESI-TOF) *m*/*z* calcd. For $C_{29}H_{44}N_2O_6Na^+$ ([M+Na]$^+$) 539.3091, found 539.3103.

methyl (2*S*)-2-((*tert*-butoxycarbonyl)amino)-3-(4-((1*S*)-2-((*S*)-1-((*tert*-butoxycarbonyl)amino)-2-phenylethyl)cyclopropyl)phenyl)propanoate

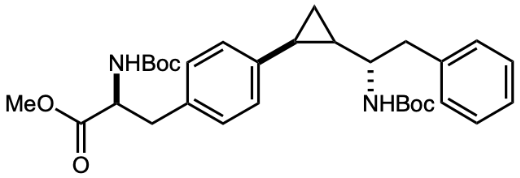

Prepared according to the general procedure **A** with tert-butyl (R)-(1-oxo-3-phenylpropan-2-yl)carbamate (124.7 mg, 0.5 mmol, 1.00 equiv), methyl (S)-2-((tert-butoxycarbonyl)amino)-3-(4-vinylphenyl)propanoate (305.4 mg, 1 mmol, 2.00 equiv), 4-CzIPN (3.9 mg, 50 µmol, 0.01 equiv), Fe(OEP)Cl (23.4 mg, 0.04 mmol, 0.075 equiv), imidazole (2.6 mg, 0.075 mmol, 0.05 equiv), **HEH-1** (387 mg, 1.25 mmol, 2.5 equiv), and standard solvent mixture TAA/PhCN/H_2O (1.66 mL). Reaction was sparged for 5 minutes under positive N_2 pressure, then irradiated at 20% light intensity for 16 hours. After irradiation, the solvent was removed under reduced pressure. The crude residue was loaded onto 50 g Biotage Sf.r normal phase column and purified with eluents 0%-100% EtOAc in hexanes. The fractions containing product were concentrated and further purified by preparative HPLC (XBridge BEH C18 OBD column, 20%-100% MeCN in H_2O with 0.1% NH4OH) to furnish product as a white solid (108.0 mg, 200.5 µmol, 40.1% yield, 4:1 dr).

Summary of diastereomers and rotamers
^{1}H NMR (500 MHz, $CDCl_3$) δ 7.28 (s, 0.6H), 7.23 – 6.92 (m, 7H), 6.78 (d, *J* = 7.8 Hz, 2H), 5.04 – 4.27 (m, 3H), 3.72 (s, 2.4H), 3.71 (s, 0.6H), , 3.42 – 3.38 (m, 1H), 3.17 – 2.80 (m, 4H), 1.60 (dt, *J* = 9.2, 5.0 Hz, 1H), 1.46 – 1.37 (m, 19H), 1.09 – 0.88 (m, 2H), 0.88 – 0.75 (m, 1H).

Summary of diastereomers
^{13}C NMR (126 MHz, $CDCl_3$) δ 172.52, 155.55, 155.23, 141.16, 137.91, 137.85, 133.20, 129.83, 129.76, 129.31, 129.23, 128.42, 126.49, 126.38, 126.11, 125.99, 80.04, 79.40, 55.95, 54.56, 52.31, 41.77, 37.97, 28.52, 28.45, 27.83, 27.72, 22.47, 21.21, 14.58, 13.97.

HRMS (ESI-TOF) *m*/*z* calcd. For $C_{31}H_{42}N_2O_6Na^+$ ([M+Na]$_+$) 561.2935, found 561.2952.

tert-butyl-4-(((1R,2R)-2-(5-((3,5-dimethylphenoxy)methyl)-2-oxooxazolidin-3-yl)cyclopropyl)methyl)piperidine-1-carboxylate

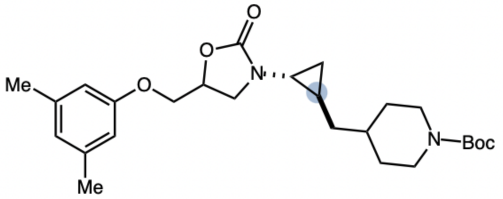

Prepared according to the general procedure **A** with 5-[(3,5-dimethylphenoxy)methyl]-3-vinyl-oxazolidin-2-one (247.3 mg, 1.0 mmol, 2.00 equiv), tert-butyl 4-(2-oxoethyl)piperidine-1-carboxylate (133.7 mg, 0.5 mmol, 1.0 equiv), Ir[dF(Me)ppy]$_2$(dtbbpy)PF$_6$ (5.1 mg, 5.00 μmol, 0.01 equiv), Fe(OEP)Cl (23.4 mg, 37.5 μmol, 0.075 equiv), imidazole (2.55 mg, 37.5 μmol, 0.075 equiv), HEH-1 (387 mg, 1.25 mmol, 2.5 equiv), and standard solvent mixture TAA/PhCN/H_2O (1.66 mL, 0.3 M). The reaction was sparged for 5 minutes under positive N_2 pressure, then irradiated at 20% light intensity for 16 hours (1500 rpm, 2000 stir rate).

After irradiation, the solvent was removed under reduced pressure. The crude residue was loaded onto 25 g Biotage Sf.r normal phase column and purified with eluents 0%-100% EtOAc in hexanes. The fractions containing product were concentrated and further purified by preparative HPLC (XBridge BEH C18 OBD column, 40%-100% MeCN in H_2O with 0.1% NH_4OH) to furnish product as a yellow oil (137.6 mg, 300.0 μmol, 60% yield, 8:1 d.r.).

^{1}H NMR (500 MHz, CDCl$_3$) δ 6.63 (s, 1H), 6.50 (d, *J* = 3.4 Hz, 2H), 4.73 (dp, *J* = 13.1, 4.6, 4.6, 4.1, 4.1 Hz, 1H), 4.04 (dtd, *J* = 23.5, 10.0, 9.8, 5.6 Hz, 4H), 3.70 – 3.60 (m, 1H), 3.46 (ddd, *J* = 8.7, 5.9, 2.0 Hz, 1H), 2.74 – 2.65 (m, 2H), 2.27 (s, 7H), 1.83 – 1.70 (m, 2H), 1.66 – 1.54 (m, 1H), 1.44 (s, 9H), 1.30 (ddt, *J* = 13.9, 10.0, 5.2, 5.2 Hz, 1H), 1.21 – 1.04 (m, 4H), 0.89 (ddd, *J* = 9.1, 5.4, 3.5 Hz, 1H), 0.57 (q, *J* = 6.1, 6.0, 6.0 Hz, 1H). [Summary of diastereomers]

^{13}C NMR (126 MHz, CDCl$_3$) δ 158.4, 158.4, 158.2, 158.1, 154.9, 139.5, 139.4, 123.4, 112.3, 79.2, 70.9, 70.9, 68.0, 67.8, 48.9, 48.6, 44.0, 36.5, 34.6, 32.4, 32.2, 32.2, 30.2, 30.0, 28.5, 21.4, 16.3, 16.0, 11.2, 11.1. [Summary of diastereomers]

HRMS (ESI-TOF) *m*/*z* calcd. For $C_{26}H_{38}N_2O_5Na^+$ ([M+Na]$_+$) 481.2673, found 481.2687.

***tert*-butyl ((1*r*,4*r*)-4-((2-(5-((3,5-dimethylphenoxy)methyl)-2-oxooxazolidin-3-yl)cyclopropyl)methyl)cyclohexyl)carbamate**

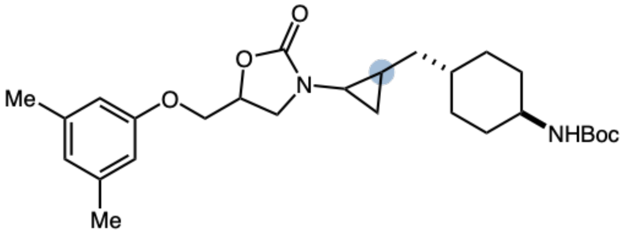

Prepared according to the general procedure **A** with 5-[(3,5-dimethylphenoxy)methyl]-3-vinyl-oxazolidin-2-one (247.3 mg, 1.0 mmol, 2.00 equiv), tert-butyl N-[4-(2-oxoethyl)cyclohexyl]carbamate (241.3 mg, 0.5 mmol, 1.0 equiv), Ir[dF(Me)ppy]$_2$(dtbbpy)PF$_6$ (5.1 mg, 5.00 μmol, 0.01 equiv), Fe(OEP)Cl (23.4 mg, 37.5 μmol, 0.075 equiv), imidazole (2.55 mg, 37.5 μmol, 0.075 equiv), HEH-1 (387 mg, 1.25 mmol, 2.5 equiv), and standard solvent mixture TAA/PhCN/H_2O (1.66 mL, 0.3 M). The reaction was sparged for 5 minutes under positive N_2 pressure, then irradiated at 20% light intensity for 16 hours (1500 rpm, 2000 stir rate). After irradiation, the solvent was removed under reduced pressure. The crude residue was loaded onto 25 g Biotage Sf.r normal phase column and purified with eluents 0%-100% EtOAc in hexanes. The fractions containing product were concentrated and further purified by preparative HPLC (XBridge BEH C18 OBD column, 40%-100% MeCN in H_2O with 0.1% NH_4OH) to furnish product as a yellow oil (125.0 mg, 264.4 μmol, 53% yield, 1.4:1 dr).

^{1}H NMR (500 MHz, CDCl$_3$) δ 6.63 (s, 1H), 6.51 (s, 2H), 4.77 – 4.68 (m, 1H), 4.35 (s, 1H), 4.11 – 3.98 (m, 2H), 3.64 (td, *J* = 8.8, 8.8, 2.7 Hz, 1H), 3.46 (dd, *J* = 8.8, 5.9 Hz, 1H), 3.35 (s, 1H), 2.28 (s, 7H), 2.03 – 1.96 (m, 2H), 1.92 – 1.80 (m, 2H), 1.44 (s, 10H), 1.32 – 1.22 (m, 1H), 1.19 – 0.96 (m, 6H), 0.90 (dt, *J* = 9.2, 4.8, 4.8 Hz, 1H), 0.57 (q, *J* = 6.2, 6.0, 6.0 Hz, 1H).

^{13}C NMR (126 MHz, CDCl$_3$) δ 158.2, 157.4, 157.4, 155.3, 139.4, 123.4, 112.3, 70.7, 70.7, 68.0, 68.0, 49.9, 47.9, 47.9, 39.7, 37.0, 33.4, 32.2, 31.9, 31.9, 31.8, 31.8, 28.5, 21.4, 17.7, 13.0, 13.0.

HRMS (ESI-TOF) *m*/*z* calcd. For $C_{27}H_{41}N_2O_5Na^+$ ([M+Na]$_+$) 495.2829, found 495.2829.

tert-butyl 4-(((1_S_,2_S_)-2-(2-fluoro-4-((_R_)-5-(hydroxymethyl)-2-oxooxazolidin-3-yl)phenyl)cyclopropyl)methyl)piperidine-1-carboxylate

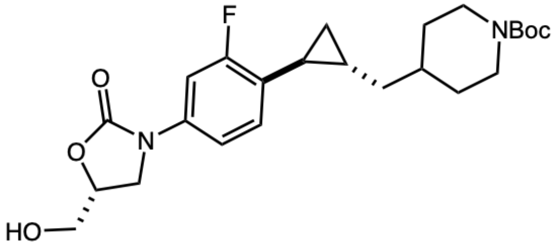

Prepared according to the general procedure **A** with tert-butyl 4-(2-oxoethyl)piperidine-1-carboxylate (113.7 mg, 0.5 mmol, 1.00 equiv), (*R*)-3-(3-fluoro-4-vinylphenyl)-5-(hydroxymethyl)oxazolidin-2-one (237.2 mg, 1.00 mmol, 2.00 equiv), 4CzIPN (3.9 mg, 10 μmol, 0.01 equiv), Fe(OEP)Cl (23.4 mg, 0.04 mmol, 0.075 equiv), imidazole (2.6 mg, 0.04 mmol, 0.075 equiv), **HEH-1** (387 mg, 1.25 mmol, 2.5 equiv), and standard solvent mixture TAA/PhCN/H_2O (1.66 mL). Reaction was sparged for 5 minutes under positive N_2 pressure, then irradiated at 20% light intensity for 16 hours. After irradiation, the solvent was removed under reduced pressure. The crude residue was loaded onto 50 g Biotage Sf.r phase column and purified with eluents 0%-100% EtOAc in hexanes, then the fractions containing product were further purified by preparative HPLC (XBridge BEH C18 OBD column, 0%-60% MeCN in H_2O with 0.1% NH_4OH) to furnish product as a white solid(116.5 mg, 259.6 μmol, 52% yield, 3.7:1 dr).

^{1}H NMR (500 MHz, $CDCl_3$) δ 7.53 – 7.34 (m, 1H), 7.20 – 7.12 (m, 1H), 7.10 – 6.80 (m, 1H), 4.81 – 4.72 (m, 1H), 4.14 – 3.91 (m, 5H), 3.89 – 3.75 (m, 1H), 2.81 – 2.46 (m, 2H), 2.15 – 2.09 (m, 0.24H), 1.84 – 1.64 (m, 3H), 1.60 (ddq, *J* = 15.0, 7.0, 3.6 Hz, 1H), 1.46 (d, *J* = 8.5 Hz, 8H), 1.44 – 1.30 (m, 2H), 1.26 – 0.88 (m, 4H), 0.77 (dt, *J* = 8.6, 5.0 Hz, 0.74H), 0.66 – 0.62 (m, 0.27H).

^{13}C NMR (126 MHz, $CDCl_3$) δ 162.87 (d, *J* = 224.7 Hz), 161.64 (d, *J* = 244.3 Hz), 155.07, 154.61, 154.57, 137.41 (d, *J* = 10.5 Hz), 136.67 (d, *J* = 10.7 Hz), 130.29 (d, *J* = 6.0 Hz), 126.41 (d, *J* = 5.8 Hz), 126.24 (d, *J* = 14.9 Hz), 113.63 (d, *J* = 2.8 Hz), 113.58 (d, *J* = 2.7 Hz), 112.81 (d, *J* = 3.3 Hz), 112.76 (d, *J* = 3.2 Hz), 105.85 (dd, *J* = 28.1, 1.6 Hz), 105.54 (dd, *J* = 28.4, 5.0 Hz), 79.36, 79.32, 72.89, 62.92, 46.47, 46.43, 44.18, 41.39, 36.67, 36.47, 35.52, 32.30, 32.27, 32.21, 32.18, 28.61, 20.34, 16.33, 16.19, 16.16, 14.71, 14.51,14.49, 9.60.

HRMS *m/z* calcd. For $C_{24}H_{34}FN_2O_5^+$ ([M+H]+) 449.2447, found 449.2444.

N-(2-((2S)-2-(2-fluoro-4-((R)-5-(hydroxymethyl)-2-oxooxazolidin-3-yl)phenyl)cyclopropyl)ethyl)-N-methyl-2-(trifluoromethyl)thiazole-4-carboxamide

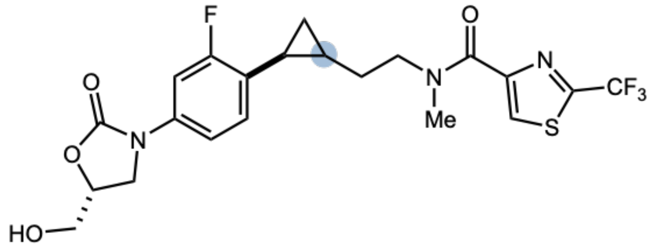

Prepared according to the general procedure **A** with 3-(3-fluoro-4-vinyl-phenyl)-5-(hydroxymethyl)oxazolidin-2-one (237 mg, 1.0 mmol, 2.00 equiv), N-methyl-N-(3-oxopropyl)-2-(trifluoromethyl)thiazole-4-carboxamide (133.1 mg, 0.5 mmol, 1.0 equiv), 4CzIPN (3.94 mg, 5.00 μmol, 0.01 equiv), Fe(OEP)Cl (23.4 mg, 37.5 μmol, 0.075 equiv), imidazole (2.55 mg, 37.5 μmol, 0.075 equiv), **HEH-1** (387 mg, 1.25 mmol, 2.5 equiv), and standard solvent mixture TAA/PhCN/H_2O (1.66 mL, 0.3 M). The reaction was sparged for 5 minutes under positive N_2 pressure, then irradiated at 20% light intensity for 16 hours (1500 rpm, 2000 stir rate).

After irradiation, the solvent was removed under reduced pressure. The crude residue was loaded onto 25 g Biotage Sf.r normal phase column and purified with eluents 0%-100% EtOAc in hexanes. The fractions containing product were concentrated and further purified by preparative HPLC (XBridge BEH C18 OBD column, 40%-100% MeCN in H_2O with 0.1% NH_4OH) to furnish product as a yellow oil (153.5 mg, 287.2 μmol, 63% assay yield). Assay and d.r. reported based on uHPLC trace.

^{1}H NMR (500 MHz, $CDCl_3$) Major: δ 8.12 (d, J = 13.5 Hz, 1H), 7.35 – 7.27 (m, 1H), 7.06 (dd, J = 8.6, 2.3 Hz, 1H), 6.79 (dtd, J = 35.5, 8.5, 8.5, 2.0 Hz, 1H), 4.70 (ddt, J = 10.0, 7.3, 3.5, 3.5 Hz, 1H), 4.03 – 3.55 (m, 6H), 3.26 (s, 2H), 3.10 (s, 2H), 1.89 – 1.67 (m, 3H), 0.96 – 0.72 (m, 3H).

^{13}C NMR (126 MHz, $CDCl_3$) Major: δ 162.4, 162.3, 162.3, 160.5, 154.9, 154.7, 154.6, 154.2, 153.9, 152.3, 152.0, 136.9, 136.8, 136.8, 136.7, 128.2, 127.7, 126.6, 126.5, 126.5, 126.4, 125.6, 125.5, 125.4, 125.3, 122.7, 120.5, 118.3, 116.2, 113.4, 113.4, 105.8, 105.7, 105.5, 73.0, 62.6, 50.9, 48.9, 46.4, 37.5, 34.7, 33.1, 31.6, 19.7, 19.2, 19.2, 15.9, 15.9, 15.6, 15.6, 14.0, 14.0, 13.7, 13.7.

^{19}**F NMR (376 MHz, CDCl$_3$) Major:** δ -61.06 (d, *J* = 6.4 Hz), -117.43 – -117.67 (m).

HRMS *m/z* calcd. For $C_{21}H_{21}F_4N_3O_4SNa^+$ ([M+Na]$_+$) 510.1081, found 510.1096.

Yield and d.r. determined by uHPLC trace

Standard (Mesitylene) retention time= 4.1 mins (Integration area = 2176)

Product retention time= 3.2 and 3.3 mins (Integration area = 1465, 314)

63% assay yield, 4.6:1 d.r.

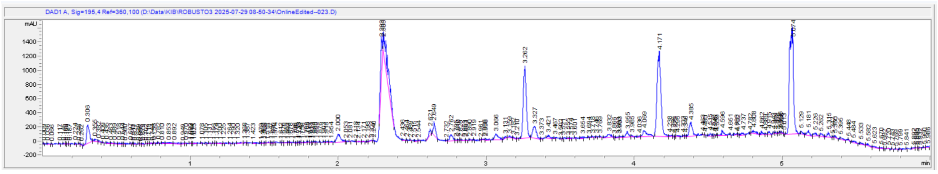

Figure S3. crude assay yield for title compound

***tert*-butyl 4-(((1*S*)-2-(2-((2*S*,3*R*)-2-(4-(benzyloxy)phenyl)-1-(4-fluorophenyl)-4-oxoazetidin-3-yl)ethyl)-2-(4-fluorophenyl)cyclopropyl)methyl)piperidine-1-carboxylate**

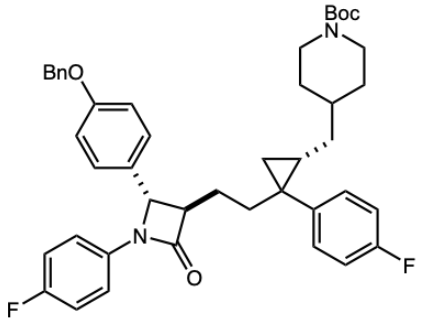

Prepared according to the general procedure **A** with tert-butyl 4-(2-oxoethyl)piperidine-1-carboxylate (113.6 mg, 0.5 mmol, 1.00 equiv), (3R,4S)-4-(4-(benzyloxy)phenyl)-1-(4-fluorophenyl)-3-(3-(4-fluorophenyl)but-3-en-1-yl)azetidin-2-one (247.8 mg, 1.00 mmol, 2.00 equiv), Ir(dFMe)ppy)$_2$dtbbpyPF$_6$ (5 mg, 5 μmol, 0.01 equiv), Fe(OEP)Cl (12.5 mg, 0.02 mmol, 0.04 equiv), imidazole (1.4 mg, 0.2 mmol, 0.04 equiv), **HEH-1** (387 mg, 1.25 mmol, 2.5 equiv), and standard solvent mixture TAA/PhCN/H_2O (1.66 mL). Reaction was sparged for 5 minutes under positive N_2 pressure, then irradiated at 20% light intensity for 16 hours. After irradiation, the solvent was removed under reduced pressure. The crude residue was loaded onto 25 g Biotage Sf.r phase column and purified with eluents 0%-100% EtOAc in hexanes, then the fractions containing product were further purified by preparative HPLC (XBridge BEH C18 OBD column, 0%-100% MeCN in H_2O with 0.1% NH_4OH) to furnish product as a pink crystalline solid (264 mg, 0.375 mmol, 75% yield (1.3:1)).

^{1}H NMR (500 MHz, CDCl$_3$) δ 7.57 – 7.26 (m, 4H), 7.26 – 7.12 (m, 5H), 7.07 – 6.79 (m, 4H), 5.07 (d, *J* = 3.2 Hz, 2H), 4.72 – 4.37 (m, 1H), 4.32 – 3.82 (m, 2H), 3.14 – 2.91 (m, 1H), 2.89 – 2.47 (m, 2 H), 2.20 – 1.55 (m, 3H), 1.49 (d, *J* = 13.1 Hz, 9H), 1.39 – 1.14 (m, 1H), 1.13 – 0.92 (m, 1H), 0.92 – 0.57 (m, 1H), 0.50 – 0.24 (m, 1H).

^{13}C NMR (126 MHz, CDCl$_3$) δ 167.48 (dd, *J* = 3.9, 2.8 Hz), 161.38 (d, *J* = 244.8 Hz), 161.36 (d, *J* = 244.7 Hz), 161.15 (d, *J* = 244.4 Hz), 161.14 (d, *J* = 244.5 Hz), 158.87 (dd, *J* = 243.2, 1.7 Hz), 154.89, 154.86, 141.73 (dd, *J* = 3.2, 1.5 Hz), 137.08 (d, *J* = 3.1 Hz), 136.96 (d, *J* = 3.0 Hz), 136.68 (t, *J* = 1.7 Hz), 134.00 (d, *J* = 2.5 Hz), 131.50 (d, *J* = 7.9 Hz), 130.34 (dd, *J* = 7.9, 1.9 Hz), 129.79,

129.77, 118.31 (d, *J* = 7.7 Hz), 115.75 (dd, *J* = 22.6, 1.7 Hz), 115.51 (t, *J* = 2.1 Hz), 79.21 (d, *J* = 1.6 Hz), 79.13, 70.08, 61.27 – 60.28 (m), 39.51, 39.38, 37.63, 37.02, 36.98, 36.52 (d, *J* = 1.9 H z), 35.91, 32.43, 32.31, 32.19, 29.56, 29.48, 28.68, 28.51, 28.49, 26.89, 26.84, 26.44 (d, *J* = 1.7 Hz), 23.23, 23.17, 22.75, 22.71, 18.86, 18.55, 18.05, 17.80.

^{19}F NMR (470 MHz, $CDCl_3$) δ -116.64 (ddt, *J* = 89.7, 15.7, 8.2 Hz, 1F), -118.26 (tdd, *J* = 13.5, 8.7, 4.7 Hz, 1F).

HRMS (ESI-TOF) *m*/*z* calcd. For $C_{39}H_{41}F_2N_2O_2^+$ ([M+H$^+$]$^+$) 607.3131, found 607.3135

***tert*-butyl 4-(4-(2-((*S*)-1-((*tert*-butoxycarbonyl)amino)ethyl)cyclopropyl)phenyl)-4-hydroxypiperidine-1-carboxylate**

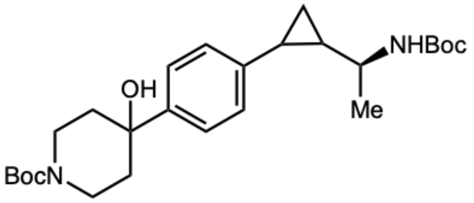

Prepared according to the general procedure **A** with tert-butyl N-(1-methyl-2-oxo-ethyl)carbamate (86.6 mg, 0.500 mmol, 1.0 equiv.), benzyl 4-hydroxy-4-(4-vinylphenyl)piperidine-1-carboxylate (337.4 mg, 1.00 mmol, 2.0 equiv.), 4CzIPN (3.9 mg, 50 μmol, 0.01 equiv), Fe(OEP)Cl (23.4 mg, 0.04 mmol, 0.075 equiv), imidazole (2.6 mg, 0.04 mmol, 0.075 equiv), **HEH-1** (387 mg, 1.25 mmol, 2.5 equiv), and standard solvent mixture DMF (1.66 mL). Reaction was sparged for 5 minutes under positive N_2 pressure, then irradiated at 20% light intensity for 16 hours. After irradiation, the solvent was removed under reduced pressure. The crude residue was loaded onto 50 g Biotage Sf.r phase column and purified with eluents 0%-100% EtOAc in hexanes, then the fractions containing product were further purified by preparative HPLC (XBridge BEH C18 OBD column, 0%-60% MeCN in H_2O with 0.1% NH_4OH) to furnish product as a white solid (167.0 mg, 363 μmol, 72.5% yield, >20:1 d.r.).

^{1}H NMR (500 MHz, $CDCl_3$) δ 7.35 (d, J = 8.5 Hz, 2H), 7.04 (d, J = 8.2 Hz, 2H), 4.61 – 4.30 (m, 1H), 4.20 – 3.78 (m, 2H), 3.38 – 3.30 (m, 1H), 3.24 (t, J = 12.9 Hz, 2H), 2.07 – 1.92 (m, 2H), 1.79 (dt, J = 8.7, 5.0 Hz, 1H), 1.75 – 1.67 (m, 2H), 1.48 (s, 9H), 1.45 (s, 9H), 1.47 – 1.40 (m, 2H), 1.23 (d, J = 6.7 Hz, 3H), 1.16 – 1.02 (m, 2H), 0.89 (dt, J = 8.3, 5.1 Hz, 1H).

^{13}C NMR (126 MHz, $CDCl_3$) δ 155.55, 155.05, 145.51, 141.97, 126.12, 124.61, 79.64, 71.51, 50.20, 38.28, 29.64, 28.64, 28.58, 21.22, 21.00, 14.17.

HRMS *m*/*z* calcd. For $C_{26}H_{40}N_2O_5Na^+$ ([M+Na]$^+$) 483.2829, found 483.2834.

tert-butyl 5'-(2-(3-(tert-butoxycarbonyl)-3-azaspiro[5.5]undecan-9-yl)cyclopropyl)-3'H-spiro[azetidine-3,1'-isobenzofuran]-1-carboxylate

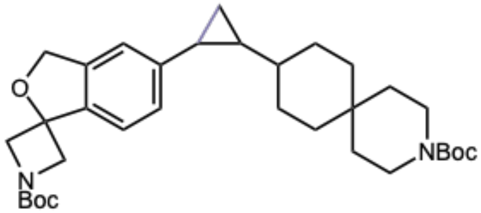

Prepared according to the general procedure **A** with tert-butyl 9-formyl-3-azaspiro[5.5]undecane-3-carboxylate (140.69 mg, 0.500 mmol, 1.0 equiv.), *tert*-butyl 5'-vinyl-3'*H*-spiro[azetidine-3,1'-isobenzofuran]-1-carboxylate (287.35 mg , 1.00 mmol, 2.0 equiv.), 4CzIPN (3.9 mg, 50 μmol, 0.01 equiv), Fe(OEP)Cl (23.4 mg, 0.04 mmol, 0.075 equiv), imidazole (2.6 mg, 0.04 mmol, 0.075 equiv), **HEH-1** (387 mg, 1.25 mmol, 2.5 equiv), and standard solvent mixture TAA/PhCN/H_2O (1.66 mL, 0.3 M). Reaction was sparged for 5 minutes under positive N_2 pressure, then irradiated at 20% light intensity for 16 hours. After irradiation, the solvent was removed under reduced pressure. The crude residue was loaded onto 50 g Biotage Sf.r phase column and purified with eluents 0%-100% EtOAc in hexanes, then the fractions containing product were further purified by preparative HPLC (XBridge BEH C18 OBD column, 0%-60% MeCN in H_2O with 0.1% NH_4OH) to furnish product as a white solid (138.7 mg, 250.9 μmol, 50.2% yield, >20:1 d.r.).

^{1}H NMR (500 MHz, $CDCl_3$) δ 7.31 (d, J = 7.8 Hz, 1H), 7.06 (dd, J = 7.9, 1.6 Hz, 1H), 6.87 (d, J = 1.6 Hz, 1H), 5.05 (s, 2H), 4.27 (dd, J = 9.3, 1.1 Hz, 2H), 4.11 (dd, J = 9.3, 1.2 Hz, 2H), 3.39 – 3.29 (m, 4H), 1.67 (tdd, J = 10.5, 6.7, 2.7 Hz, 5H), 1.47 (s, 9H), 1.45 (s, 9H), 1.37 – 1.22 (m, 4H), 1.08 (td, J = 13.1, 3.5 Hz, 2H), 0.91 – 0.84 (m, 2H), 0.84 – 0.79 (m, 1H), 0.79 – 0.68 (m, 1H).
^{13}C NMR (126 MHz, $CDCl_3$) δ 156.63, 155.20, 145.00, 139.04, 138.78, 126.22, 120.59, 117.85, 82.68, 79.88, 79.30, 72.82, 64.27, 43.29, 41.71 – 38.08 (m), 35.67, 35.63, 31.87, 31.15, 30.25, 28.62, 28.54, 27.75, 27.33, 22.07, 15.12.

HRMS *m*/*z* calcd. For $C_{33}H_{49}N_2O_5^+$ ([M+H]+) 576.3461, found 576.3476.

6) Mechanism Data

PhotoNMR setup was conducted according to literature precedent.[6]

tert-butyl 4-formylpiperidine-1-carboxylate (106.6 mg, 0.5 mmol, 1.00 equiv), 1-fluoro-4-vinylbenzene (120.0 µl, 1.00 mmol, 2.0 equiv.), 4-CzIPN (3.9 mg, 50 µmol, 0.01 equiv), Fe(OEP)Cl (23.4 mg, 0.04 mmol, 0.075 equiv), imidazole (2.6 mg, 0.075 mmol, 0.05 equiv), **HEH-1** (387 mg, 1.25 mmol, 2.5 equiv), and D_3-ACN(1.66 mL) Reaction was sparged for 5 minutes under positive N_2 pressure. To this sealed vial under nitrogen was added 1,4 difluorobenzene (13µl, 0.125 mmol, 0.25 equiv.) then taken into the glovebox. In the glovebox, 500 µl were taken via syringe and added to a PhotoNMR tube. This tube was sealed and removed from the glovebox. This was taken to the instrument and subjected to irradiation by a 445 nm light. ^{1}H-NMR and ^{19}F NMR were collected at timepoints during the 16 hour course of reaction.

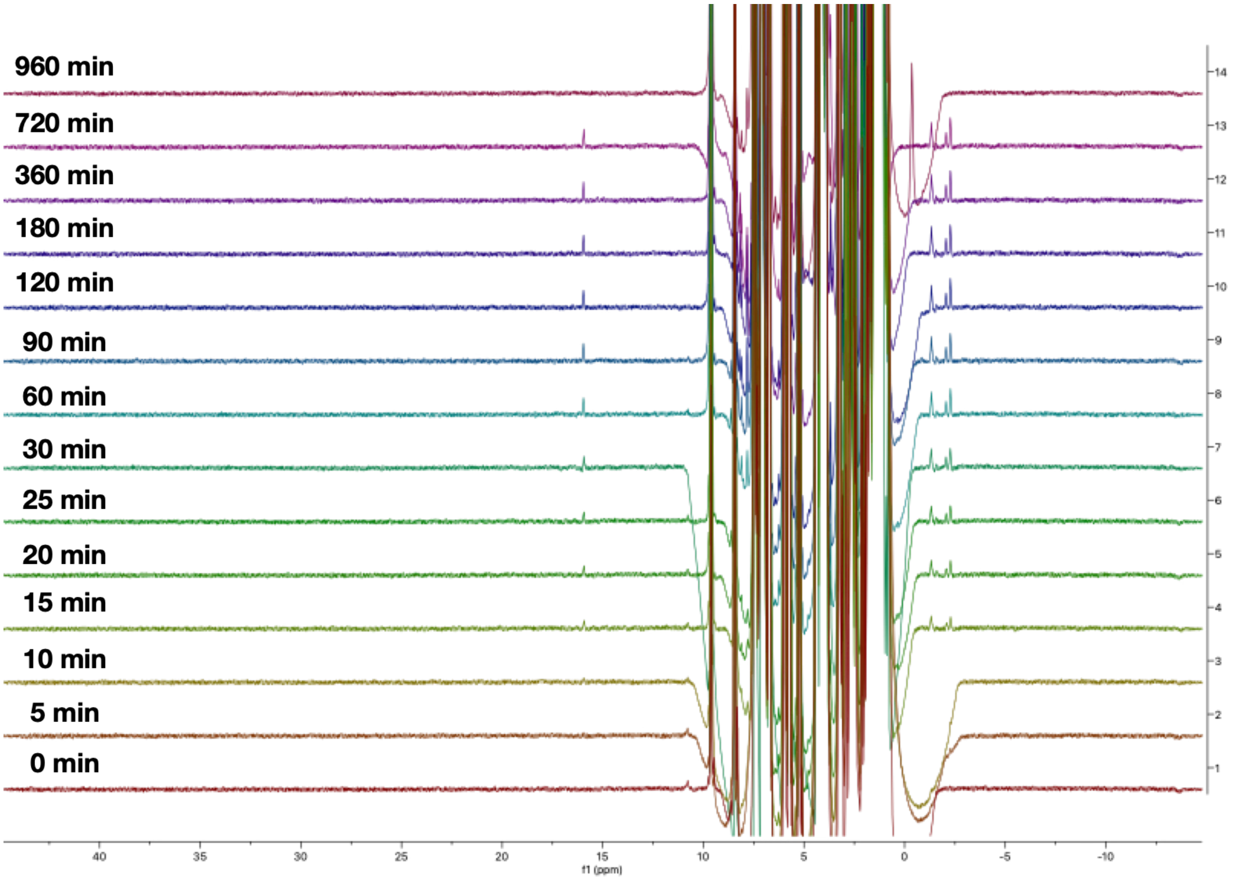

Figure S4. PhotoNMR experiment

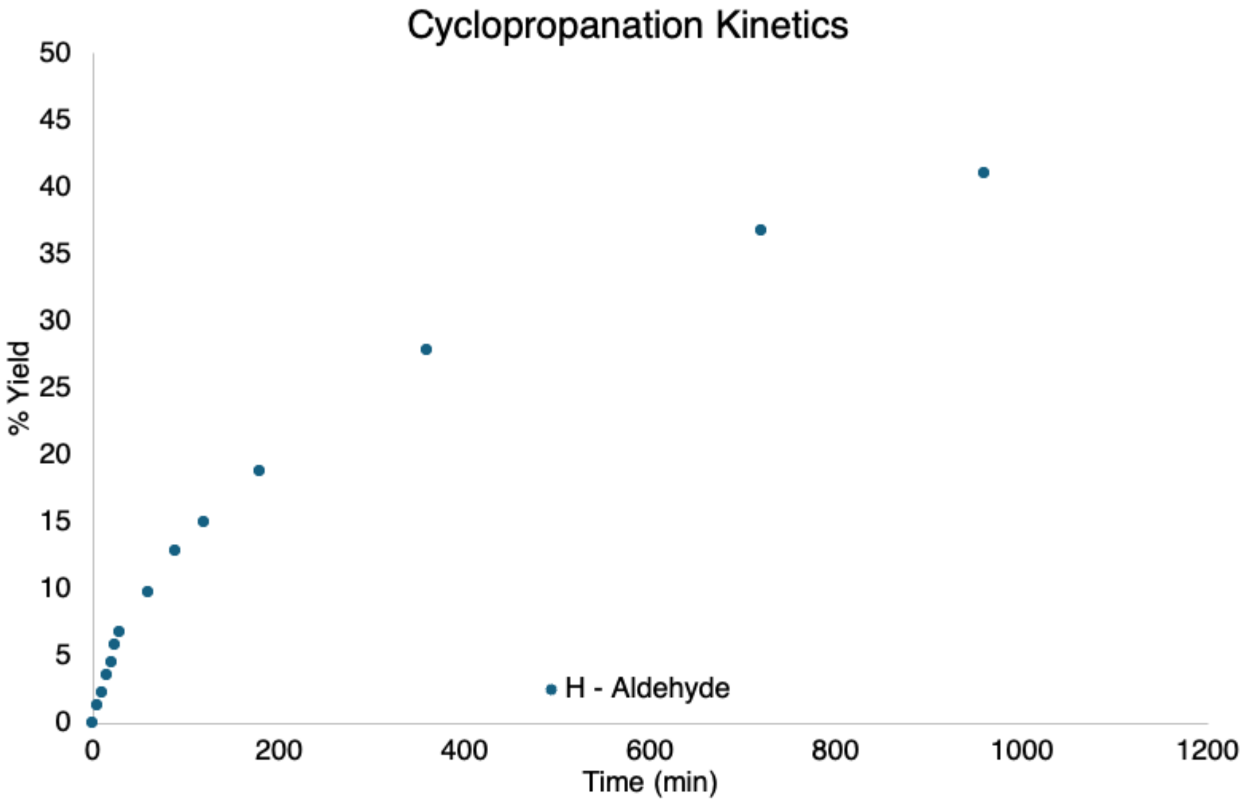

Figure S5. Cyclopropanation kinetics experiment: Assigned by ^{19}F NMR vs. 1,4-difluorobenzene internal standard.

Deuterated substrate preparation:

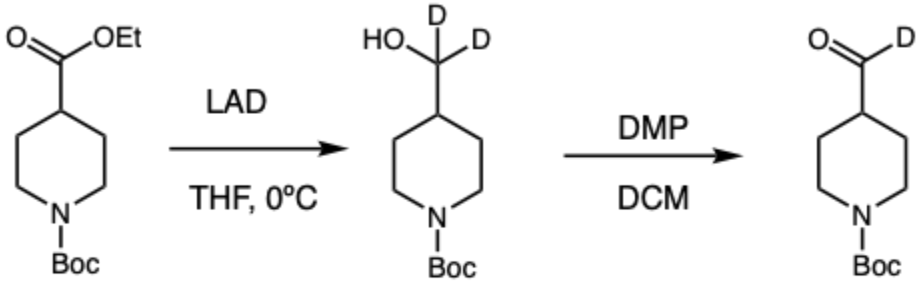

Scheme S1. Preparation of deuterated substrate

To a oven dried round bottom flask with Ethyl N-Boc-piperidine-4-carboxylate (3.68 g, 14.29 mmol, 1.5 equiv.) in 15 ml THF at 0 ºC was added portionwise $LiAlD_4$ (0.400 g, 9.53 mmol, 1.0 equiv.) and let stir for 2 hours at 0 ºC before being quenched by addition of 0.4 ml DI water, 0.4 ml 15% NaOH, and 1.2 mL DI water. $MgSO_4$ was added to the reaction and then it was diluted with ether and filtered. The collected liquid was subjected to column chromatography (0-50% ethyl acetate in hexanes) to give the product as a white solid. This product was then dissolved in DCM (0.1 M) and DMP (1.5 equiv.) was added at 0 ºC. After 16 hours, water was added and the mixture was extracted into DCM. After concentration, the mixture was subjected to column chromatography to give the product as a clear oil. Product matched reported spectra.

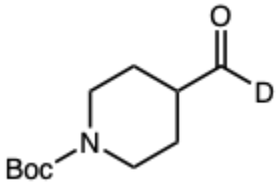

***tert*-butyl 4-(formyl-*d*)piperidine-1-carboxylate**

^{1}H NMR (500 MHz, $CDCl_3$) δ 9.66 (s, 0H), 4.04 – 3.91 (m, 2H), 2.92 (ddd, J = 13.8, 10.8, 3.1 Hz, 2H), 2.40 (tt, J = 10.6, 4.0 Hz, 1H), 1.95 – 1.82 (m, 2H), 1.56 (tdd, J = 10.8, 7.3, 5.4 Hz, 2H), 1.45 (s, 9H).
^{13}C NMR (126 MHz, $CDCl_3$) δ 205.15 – 198.97 (m), 154.69, 79.75, 47.90 – 47.76 (m), 42.91, 28.43, 25.16.

Protocol for deuterated substrate experiment:

tert-butyl 4-(formyl-*d*)piperidine-1-carboxylate (107.1 mg, 0.5 mmol, 1.00 equiv), 1-fluoro-4-vinylbenzene (120.0 µl, 1.00 mmol, 2.0 equiv.), 4-CzIPN (3.9 mg, 50 µmol, 0.01 equiv), Fe(OEP)Cl (23.4 mg, 0.04 mmol, 0.075 equiv), imidazole (2.6 mg, 0.075 mmol, 0.05 equiv), **HEH-1** (387 mg, 1.25 mmol, 2.5 equiv), and D_3-ACN(1.66 mL) Reaction was sparged for 5 minutes under positive N_2 pressure. To this sealed vial under nitrogen was added 1,4 difluorobenzene (13µl, 0.125 mmol, 0.25 equiv.) then taken into the glovebox. In the glovebox, 500 µl were taken via syringe and added to a PhotoNMR tube. This tube was sealed and removed from the glovebox. This was taken to the instrument and subjected to irradiation by a 445 nm light. ^{1}H-NMR and ^{19}F NMR were collected at timepoints during the 16 hour course of reaction.

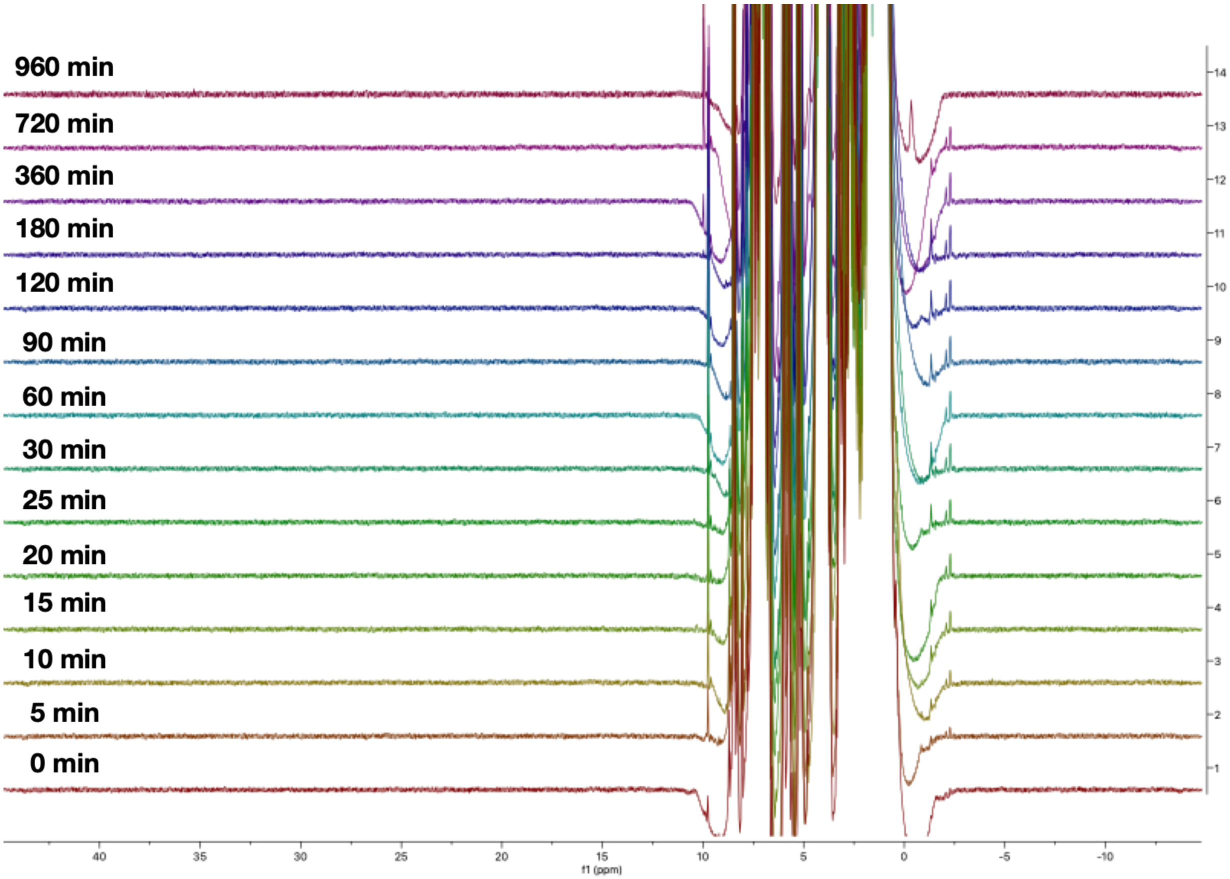

Figure S6. Deuterated probe photoNMR experimental ^{1}H NMR spectra

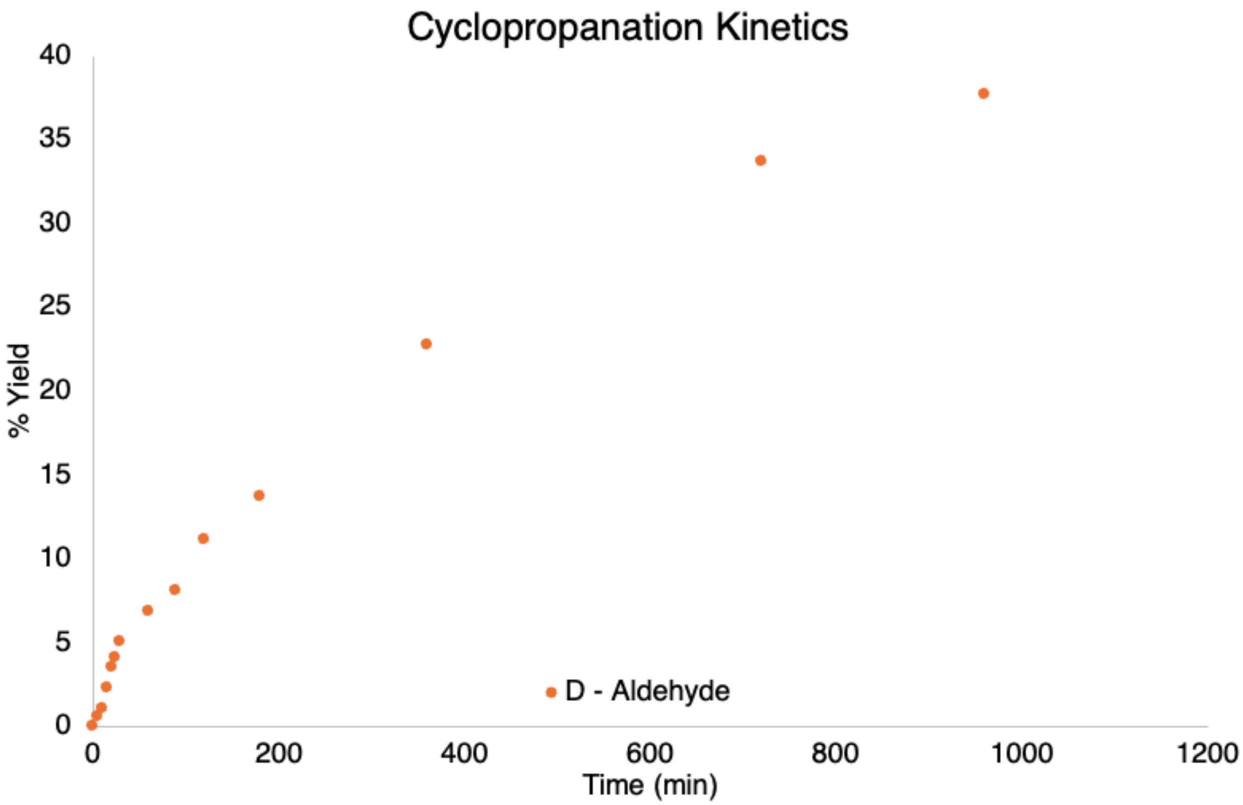

Figure S7. Deuterated probe cyclopropanation kinetics data

Competition Experiment

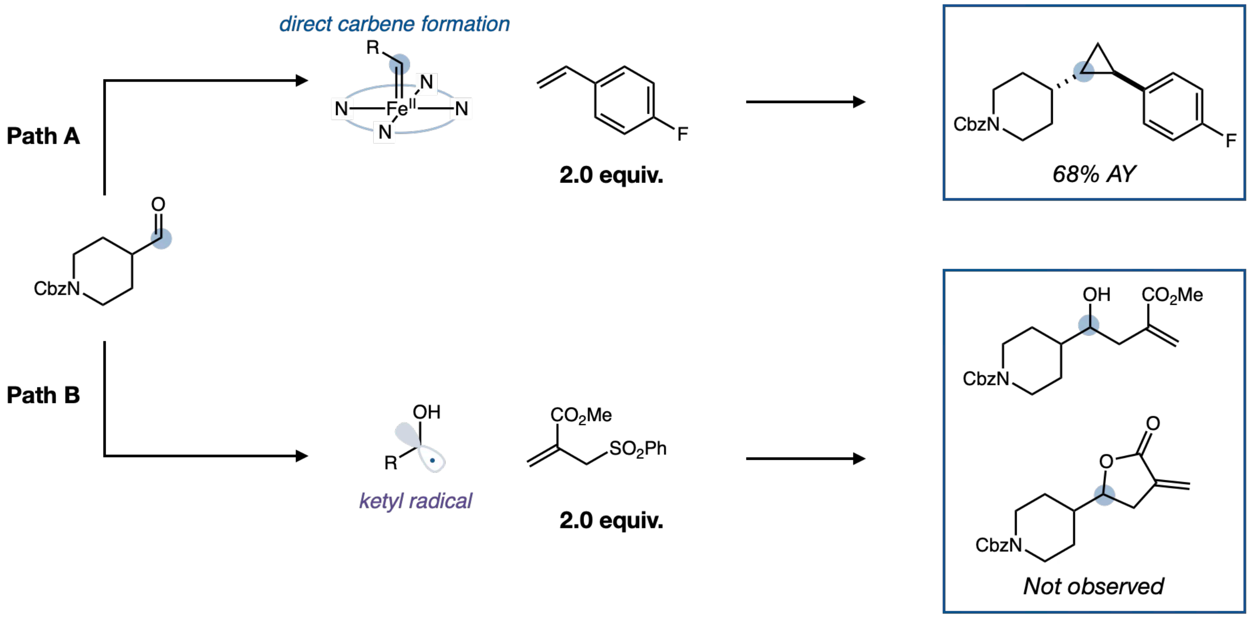

Figure S8. Competition experiment: hypothesized ketyl radical products not observed

Prepared according to the general procedure **B** with benzyl 4-formylpiperidine-1-carboxylate (123.6 mg, 0.5 mmol, 1.00 equiv), 1-fluoro-4-vinylbenzene (120.0 µl, 1.00 mmol, 2.0 equiv.), methyl 2-((phenylsulfonyl)methyl)acrylate (240.0 mg, 1.00 mmol, 2.0 equiv.) and 4-CzIPN (3.9 mg, 50 µmol, 0.01 equiv), Fe(OEP)Cl (23.4 mg, 0.04 mmol, 0.075 equiv), imidazole (2.6 mg, 0.075 mmol, 0.05 equiv), **HEH-1** (387 mg, 1.25 mmol, 2.5 equiv), and standard solvent mixture TAA/PhCN/H_2O (1.66 mL). At the end of the reaction, NMR assay was performed with 1,4 difluorobenzene (51.4 µl, 0.500 mmol, 1.0 equiv.) for ^{19}F NMR, and mesitylene (69.4 µl, 0.500 mmol, 1.0 equiv.) for ^{1}H NMR and UPLC-MS.

Assay yield for the Cyclopropane product: 69% by ^{19}F NMR
Isolated yield: 65%, 114.3 mg, 0.324 mmol
No ketyl radical addition products observed by UPLC-MS or ^{1}H NMR.

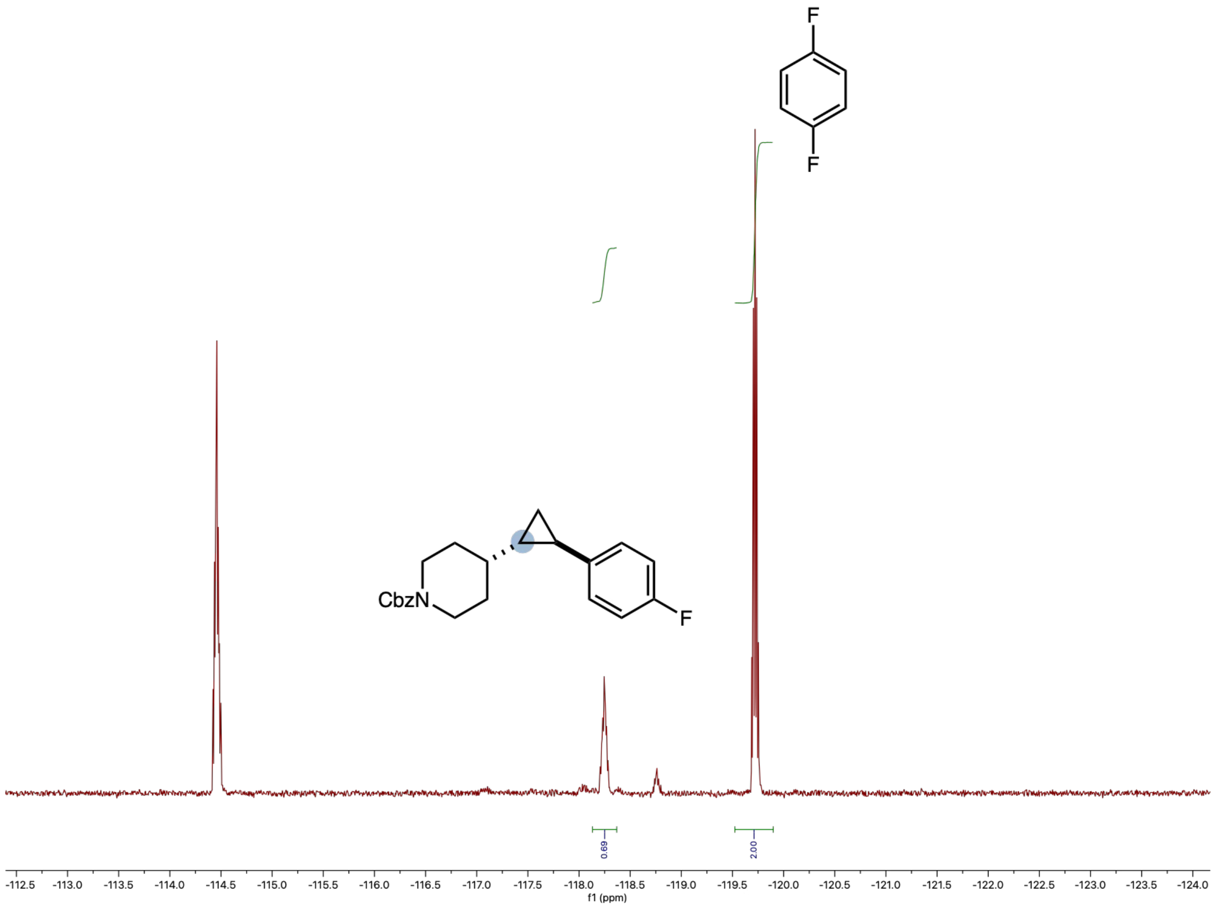

Figure S9. Experimental data for competition experiment: ^{19}F NMR versus 1,4-difluorobenzene

Modified conditions for no [Fe] experiment.

benzyl 4-formylpiperidine-1-carboxylate (123.6 mg, 0.5 mmol, 1.00 equiv), 1-fluoro-4-vinylbenzene (120.0 µl, 1.00 mmol, 2.0 equiv.), methyl 2-((phenylsulfonyl)methyl)acrylate (240.0 mg, 1.00 mmol, 2.0 equiv.) and 4-CzIPN (3.9 mg, 50 ⌠mol, 0.01 equiv), imidazole (2.6 mg, 0.075 mmol, 0.05 equiv), **HEH-1** (387 mg, 1.25 mmol, 2.5 equiv), and standard solvent mixture TAA/PhCN/H_2O (1.66 mL).The reaction was sparged for 5 minutes under positive N_2 pressure, then irradiated at 20% light intensity for 16 hours. After irradiation, the solvent was removed under reduced pressure. At the end of the reaction, NMR assay was performed with 1,4 difluorobenzene (51.4 µl, 0.500 mmol, 1.0 equiv.) for ^{19}F NMR, and mesitylene (69.4 µl, 0.500 mmol, 1.0 equiv.) for ^{1}H NMR and UPLC-MS.

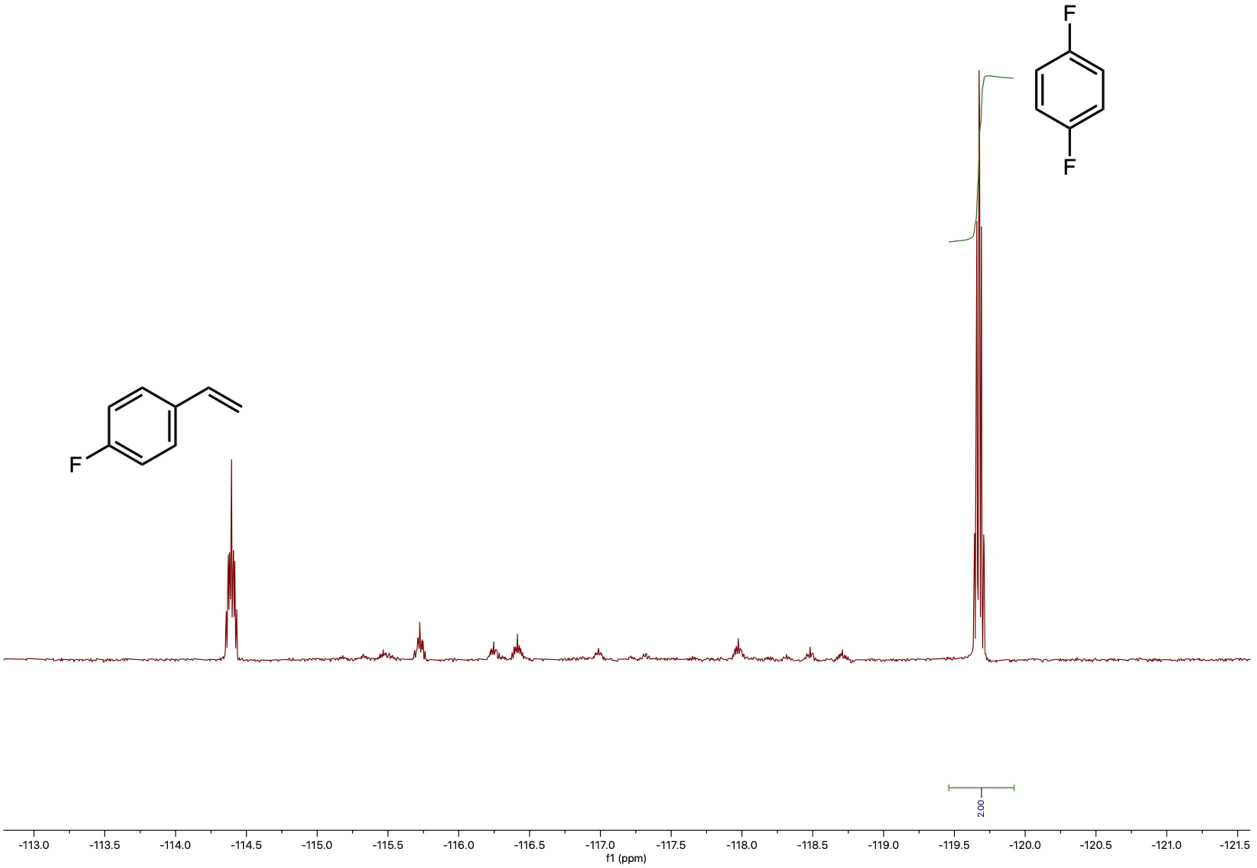

Figure S10. No Fe(OEP)-Cl experiment: ^{19}F NMR versus 1,4-difluorobenzene

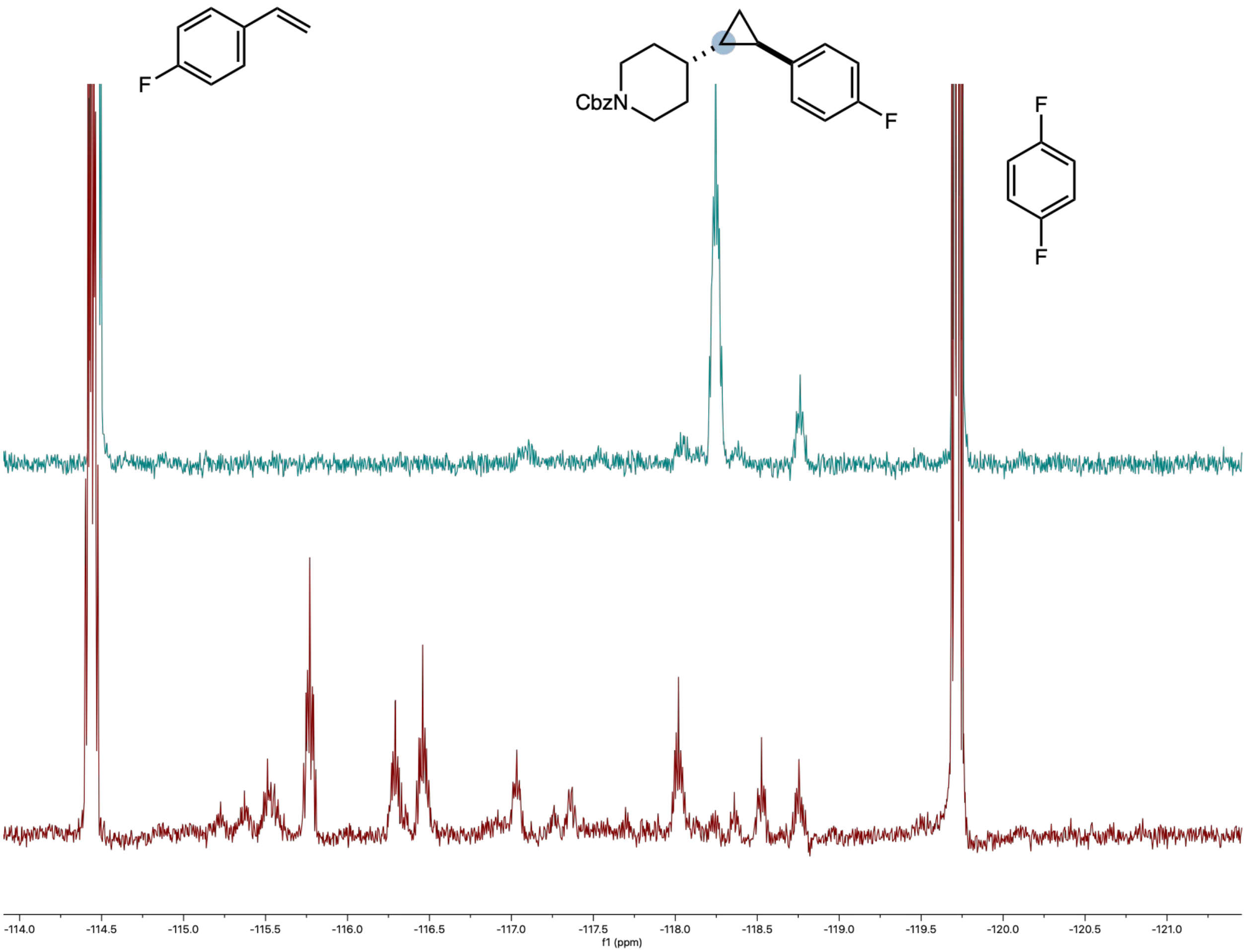

Figure S11. Baseline impurity. No peak observed by UPLC MS. ^{19}F NMR versus 1,4-difluorobenzene

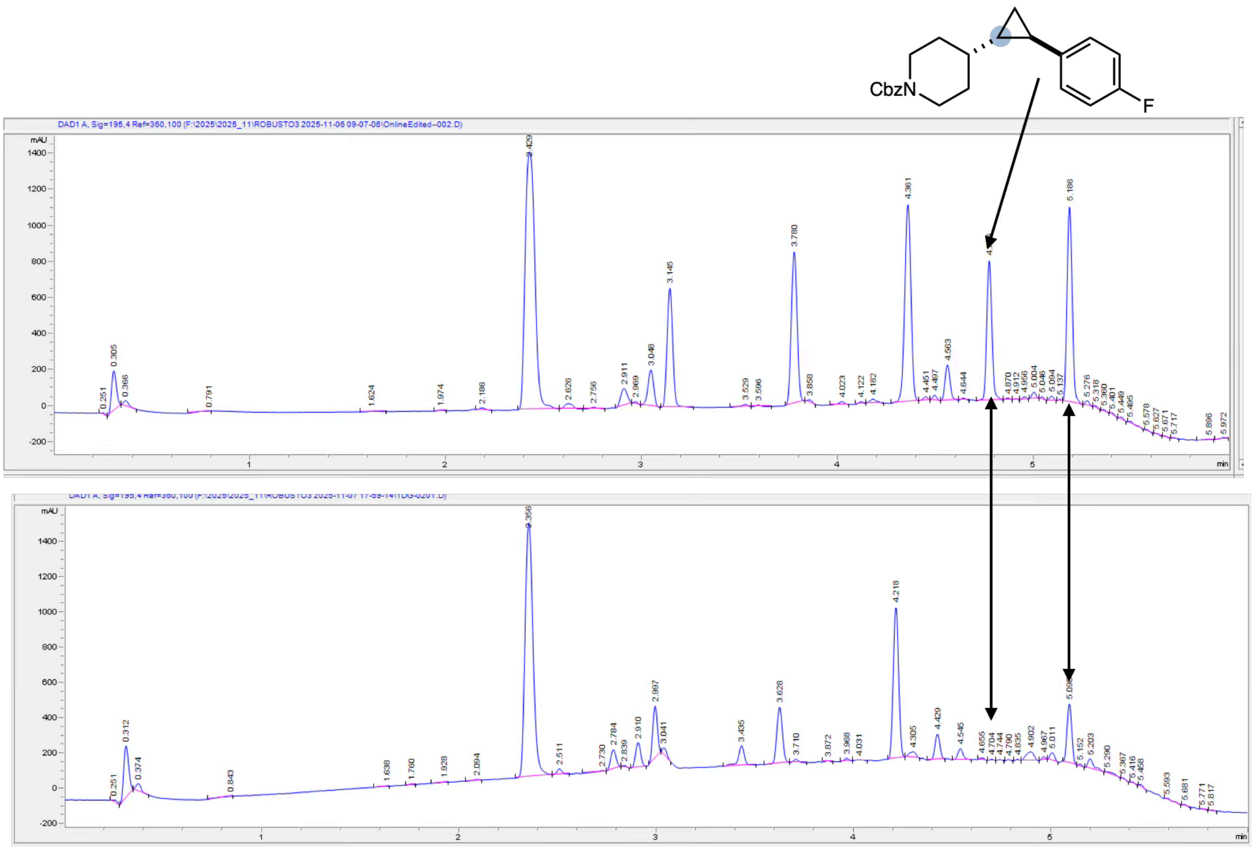

Figure S12. Baseline impurity. No peak observed by UPLC MS.

Confirmation of *trans*- relative confirmation

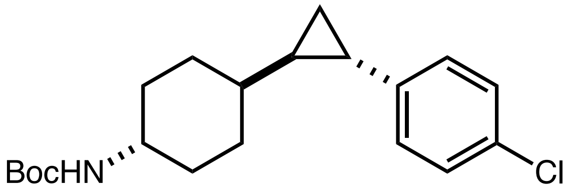

tert-butyl (4-(2-(4-chlorophenyl)cyclopropyl)cyclohexyl)carbamate

Prepared according to the general procedure **A** with tert-butyl N-(4-formylcyclohexyl)carbamate (113.7 mg, 0.5 mmol, 1.00 equiv), 1-chloro-4-vinyl-benzene (120.0 µl, 1.00 mmol, 2.0 equiv.), 4-CzIPN (3.9 mg, 50 µmol, 0.01 equiv), Fe(OEP)Cl (23.4 mg, 0.04 mmol, 0.075 equiv), imidazole (2.6 mg, 0.075 mmol, 0.05 equiv), **HEH-1** (387 mg, 1.25 mmol, 2.5 equiv), and standard solvent mixture TAA/PhCN/H_2O (1.66 mL). Reaction was sparged for 5 minutes under positive N_2 pressure, then irradiated at 20% light intensity for 16 hours. After irradiation, the solvent was removed under reduced pressure. The crude residue was loaded onto 50 g Biotage Sf.r normal phase column and purified with eluents 0%-100% EtOAc in hexanes. The fractions containing product were concentrated and dissolved in Acetonitrile (10 ml). Upon sonication, the product preicpitated out as a white solid, which was collected via filtration (81.0 mg, 231.5 µmol, 46.3% yield).

^{1}H NMR (500 MHz, $CDCl_3$) δ 7.19 (d, J = 8.4 Hz, 2H), 6.95 (d, J = 8.5 Hz, 2H), 4.65 – 4.04 (m, 1H), 3.58 – 3.29 (m, 1H), 2.07 – 1.96 (m, 2H), 1.93 – 1.83 (m, 2H), 1.65 – 1.58 (m, 1H), 1.44 (s, 9H), 1.30 – 1.15 (m, 2H), 1.10 – 0.98 (m, 2H), 0.91 – 0.72 (m, 3H), 0.72 – 0.60 (m, 1H).

^{13}C NMR (126 MHz, $CDCl_3$) δ 155.37, 142.48, 130.93, 128.41, 127.21, 79.23, 49.93, 42.35, 33.55, 33.51, 31.67, 31.25, 29.91, 28.58, 21.70, 14.95.

HRMS (ESI-TOF) *m*/*z* calcd. For $C_{20}H_{28}ClNNaO_2^+$ ($[M+Na]^+$) 372.1706, found 372.1707.

The product was dissolved in 5 ml of D2 DCM and the solvent was subjected to crystalization via slow diffuion of ether into D2 DCM. The crystal structure was solved and is shown below. Deposition number **2530892**.

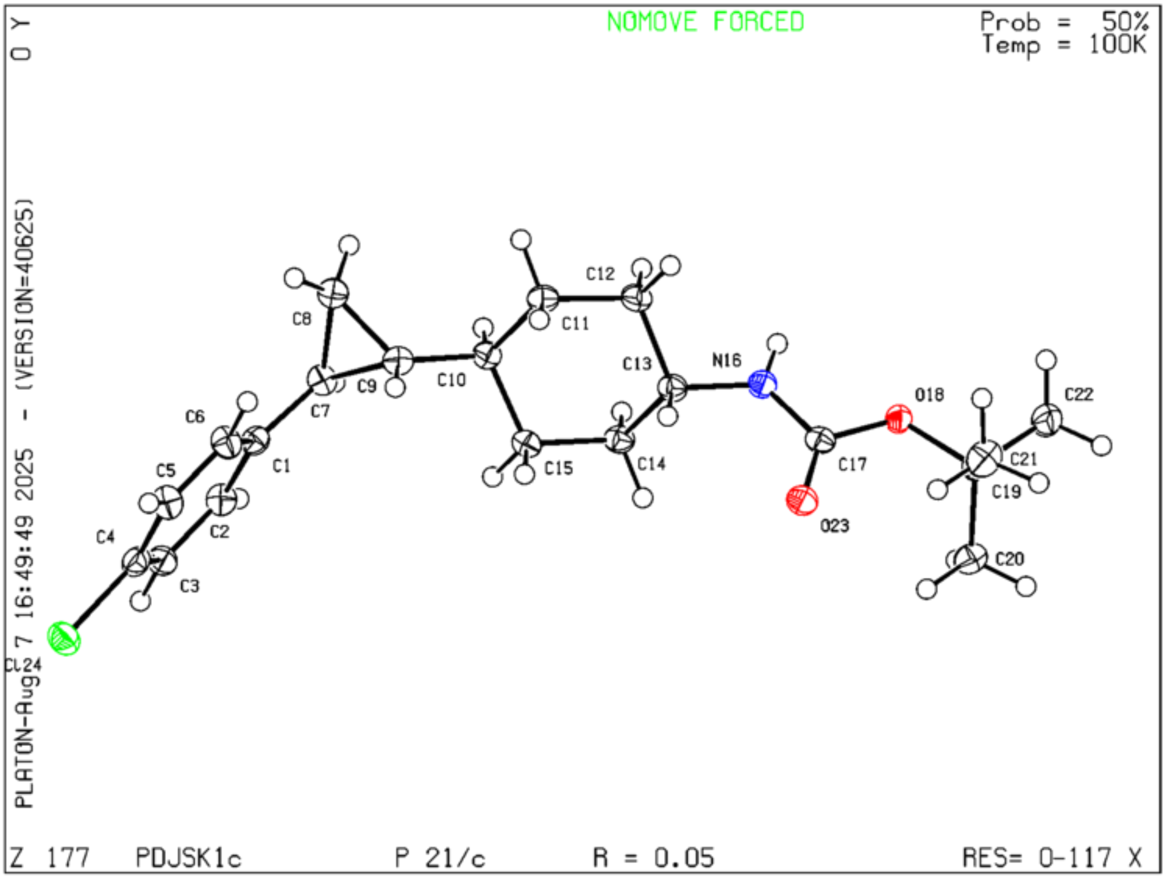

Figure S13. Crystal structure

Bond precision:	C-C = 0.0022 A		Wavelength=1.54184
Cell:	a=18.0676(4)	b=9.7214(2)	c=10.4585(2)
	alpha=90	beta=90.496(2)	gamma=90
Temperature:	100 K		

	Calculated	Reported
Volume	1836.89(7)	1836.89(7)
Space group	P 21/c	P 21/c
Hall group	-P 2ybc	-P 2ybc
Moiety formula	C20 H28 Cl N O2	?
Sum formula	C20 H28 Cl N O2	C20 H28 Cl N O2
Mr	349.88	349.88
Dx,g cm-3	1.265	1.265
Z	4	4
Mu (mm-1)	1.924	1.924
F000	752.0	752.0
F000'	755.34	
h,k,lmax	22,11,12	22,11,12
Nref	3485	3485

Tmin,Tmax	0.794,0.857	0.665,1.000
Tmin'	0.794	

Correction method= # Reported T Limits: Tmin=0.665 Tmax=1.000 AbsCorr = GAUSSIAN

Data completeness= 1.000	Theta(max)= 70.075	
R(reflections)= 0.0475(3084)		wR2(reflections)= 0.1430(3485)
S = 1.098	Npar= 224	

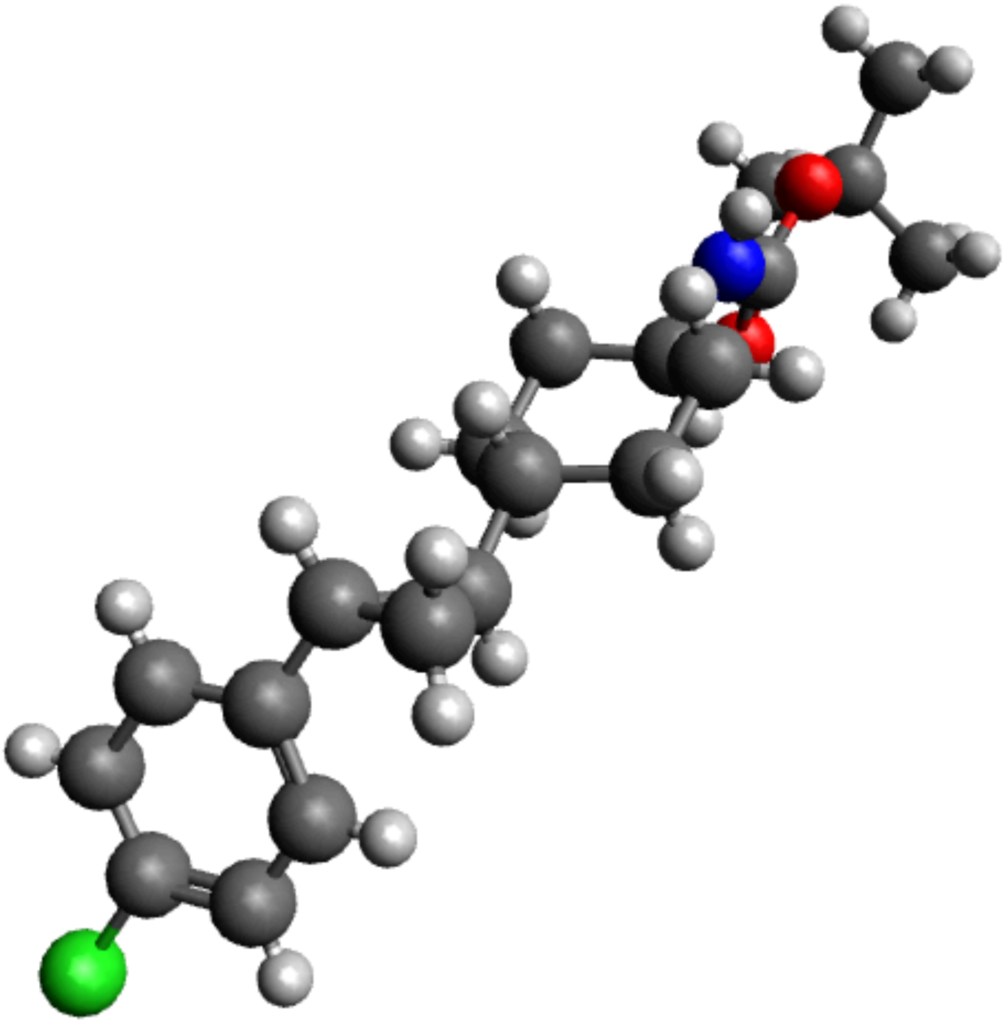

Figure S14. Crystal structure showing trans relative configuration.

Deuterium labelling experiment for mechanism of insertion

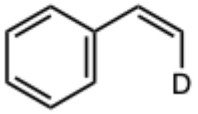

(Z)-β-deuteriostyrene

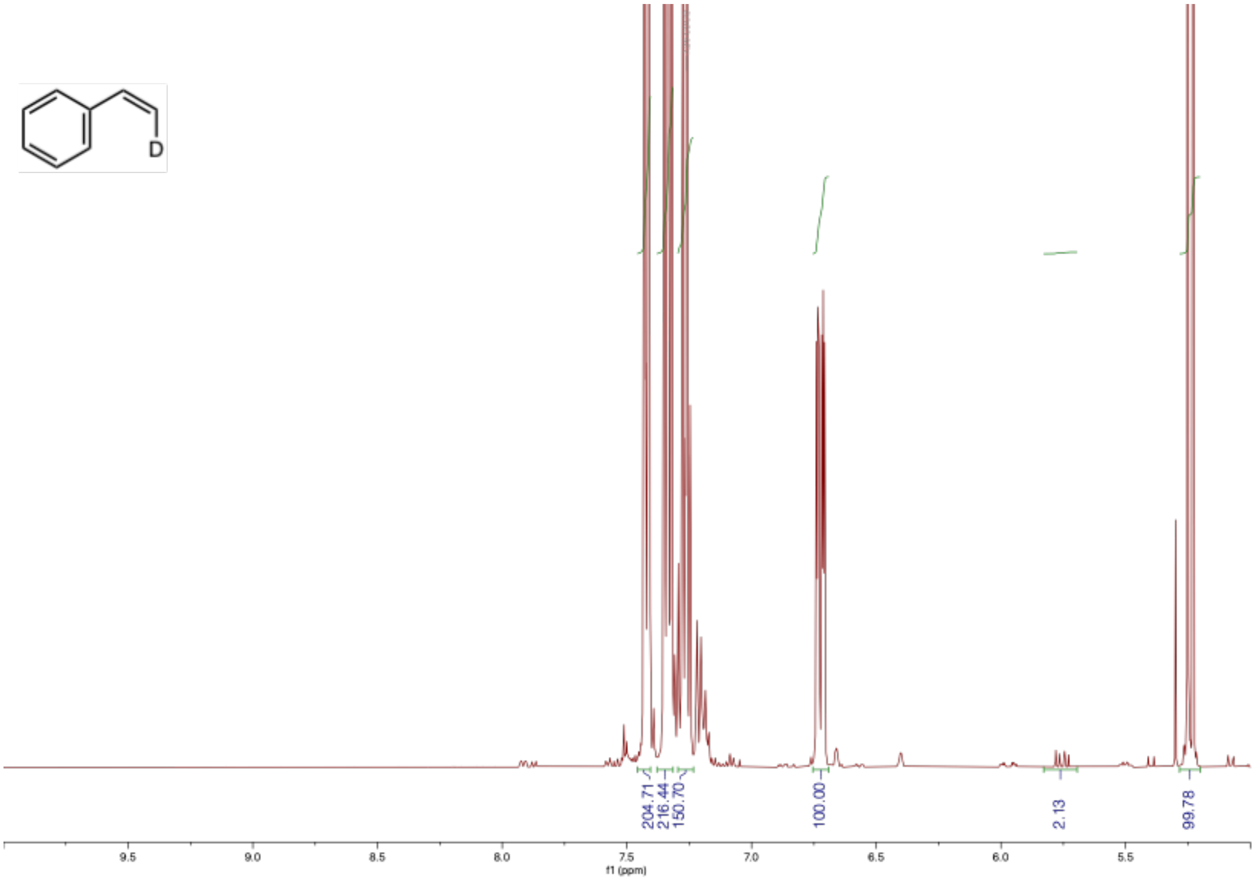

Figure S15. Deuterium labelling experiment: insertion mechanism. Data matched reported spectra with 97.87% D-incorporation.

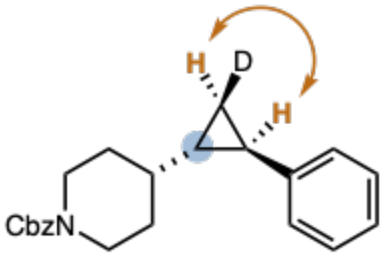

benzyl 4-((1*s*,2*r*,3*r*)-2-phenylcyclopropyl-3-*d*)piperidine-1-carboxylate

Prepared according to the general procedure **A** with benzyl 4-formylpiperidine-1-carboxylate (123.6 mg, 0.5 mmol, 1.00 equiv), (Z)-β-deuteriostyrene (116.0 µl, 1.00 mmol, 2.0 equiv.), 4-CzIPN (3.9 mg, 50 µmol, 0.01 equiv), Fe(OEP)Cl (23.4 mg, 0.04 mmol, 0.075 equiv), imidazole (2.6 mg, 0.075 mmol, 0.05 equiv), **HEH-1** (387 mg, 1.25 mmol, 2.5 equiv), and standard solvent mixture TAA/PhCN/H_2O (1.66 mL). Reaction was sparged for 5 minutes under positive N_2 pressure, then irradiated at 20% light intensity for 16 hours. After irradiation, the solvent was removed under reduced pressure. The crude residue was loaded onto 50 g Biotage Sf.r normal phase column and purified with eluents 0%-100% EtOAc in hexanes. The fractions containing product were concentrated and further purified by preparative HPLC (XBridge BEH C18 OBD column, 20%-100% MeCN in H_2O with 0.1% NH_4OH) to furnish product as a colorless oil (64.5 mg, 191.7 µmol, 38.3% yield, >20: dr).

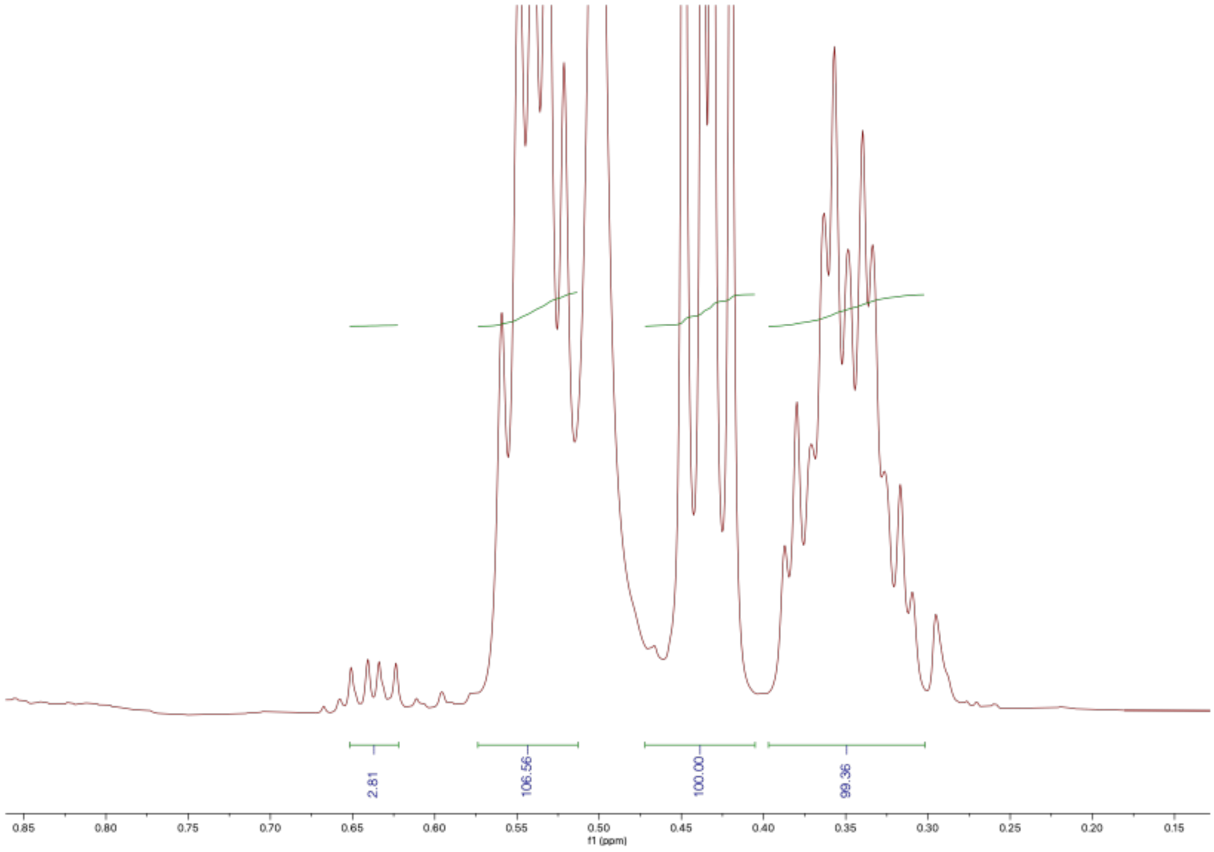

From: 0.610 ppm

To: 0.646 ppm

Residual Error:1.85e+08

#	ppm	Height	Width	L/G	Area
1	0.6424	136414.9	1.63	0.09	2167734
2	0.6324	151121.7	1.63	0.43	2290307
3	0.6257	127346.5	1.49	-0.2	1921018
4	0.6231	43939.89	2.4	0.2	1016055
5	0.6153	142849.4	1.59	0	2243873
				Sum	9638987.7
				Sum - impurity	8622932.61

Percent integration	purity*	0.89458903
Integral		2.50484927

Line fitting was applied due to inseparable impurity at 0.68-0.63.
% Specificity: 97.50/97.87 = 0.996% specific.

^{1}H NMR (500 MHz, C_6D_6) δ 7.33 – 7.28 (m, 2H), 7.15 – 7.10 (m, 4H), 7.08 – 7.04 (m, 2H), 6.95 – 6.90 (m, 2H), 5.20 (s, 2H), 4.59 – 3.90 (m, 2H), 2.46 – 2.36 (m, 2H), 1.44 – 1.20 (m, 3H), 1.10 – 0.95 (m, 3H), 0.64 (dd, J = 8.6, 5.1 Hz, 0.0281H), 0.54 (dt, J = 8.9, 5.2 Hz, 1H), 0.43 (dd, J = 8.7, 5.7 Hz, 1H), 0.40 – 0.30 (m, 1H).

^{1}H NMR (500 MHz, $CDCl_3$) δ 7.41 – 7.29 (m, 5H), 7.26 (t, J = 7.6 Hz, 2H), 7.19 – 7.13 (m, 1H), 7.09 – 7.03 (m, 2H), 5.15 (s, 2H), 4.25 – 4.12 (m, 2H), 2.79 (t, J = 12.7 Hz, 2H), 1.87 – 1.81 (m, 2H), 1.72 (dd, J = 8.7, 4.3 Hz, 1H), 1.43 – 1.29 (m, 2H), 1.01 – 0.87 (m, 2H), 0.83 (dd, J = 8.7, 5.4 Hz, 1H).

^{13}C NMR (126 MHz, $CDCl_3$) δ 155.47, 143.55, 137.12, 128.62, 128.42, 128.06, 127.98, 125.93, 125.53, 67.11, 44.32, 44.30, 41.28, 31.94, 31.58, 28.92, 21.99, 16.26 – 12.51 (m).

HRMS (ESI-TOF) *m*/*z* calcd. For $C_{22}H_{25}DNO_2^+$ ($[M+H^+]^+$) 337.2021, found 337.2025

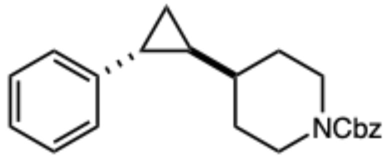

^{1}H NMR (500 MHz, C_6D_6) δ 7.33 – 7.26 (m, 2H), 7.15 – 7.10 (m, 4H), 7.09 – 7.01 (m, 2H), 6.95 – 6.88 (m, 2H), 5.20 (d, *J* = 2.6 Hz, 2H), 4.65 – 3.84 (m, 2H), 2.41 (tt, *J* = 13.1, 3.0 Hz, 2H), 1.38 (dt, *J* = 9.2, 4.9 Hz, 1H), 1.35 – 1.21 (m, 2H), 0.65 (dt, *J* = 8.4, 4.8 Hz, 1H), 0.61 – 0.50 (m, 1H), 0.45 (ddd, *J* = 8.6, 5.6, 4.5 Hz, 1H), 0.35 (tdt, *J* = 11.9, 8.0, 3.7 Hz, 1H).

^{13}C NMR (126 MHz, C_6D_6) δ 155.21, 143.72, 137.89, 128.70, 128.60, 128.45, 128.06, 126.26, 125.74, 67.12, 44.33, 40.94, 33.67 – 30.41 (m), 28.90, 22.04, 14.31.

NOE characterization

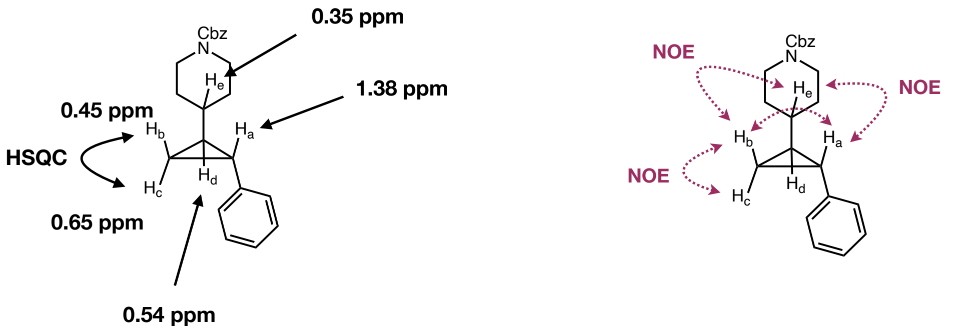

The integration of the cis to phenyl is identified as the deuterated peak, confirming the selectivity of this reaction.

Figure S16. Assignment of proton peaks on substrate **24**

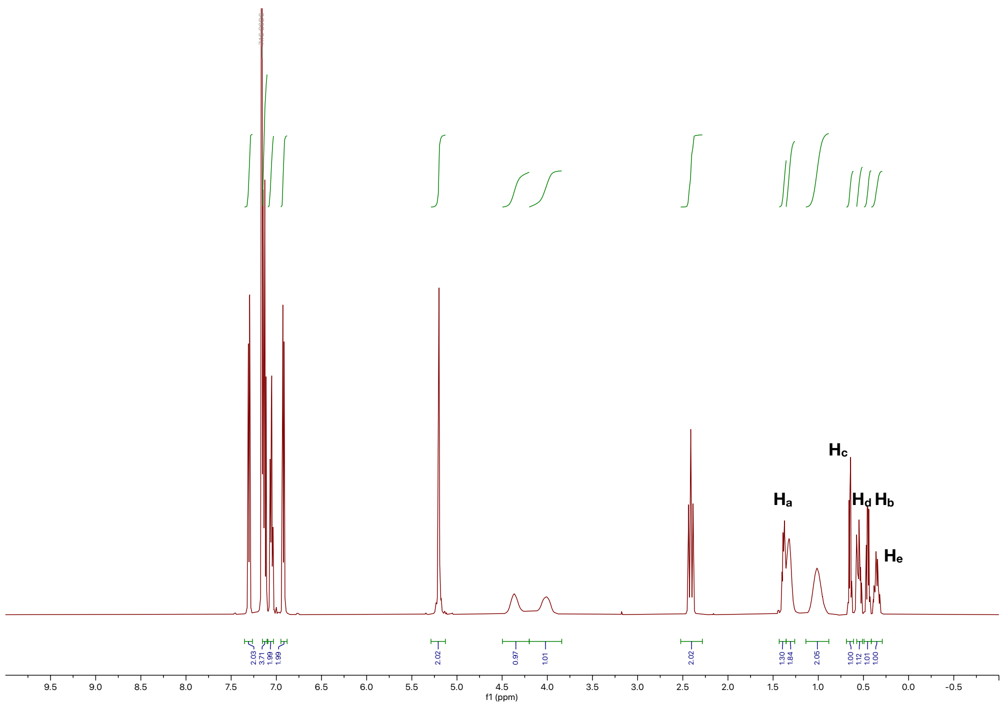

Figure S17. Proton spectra of substrate **24**

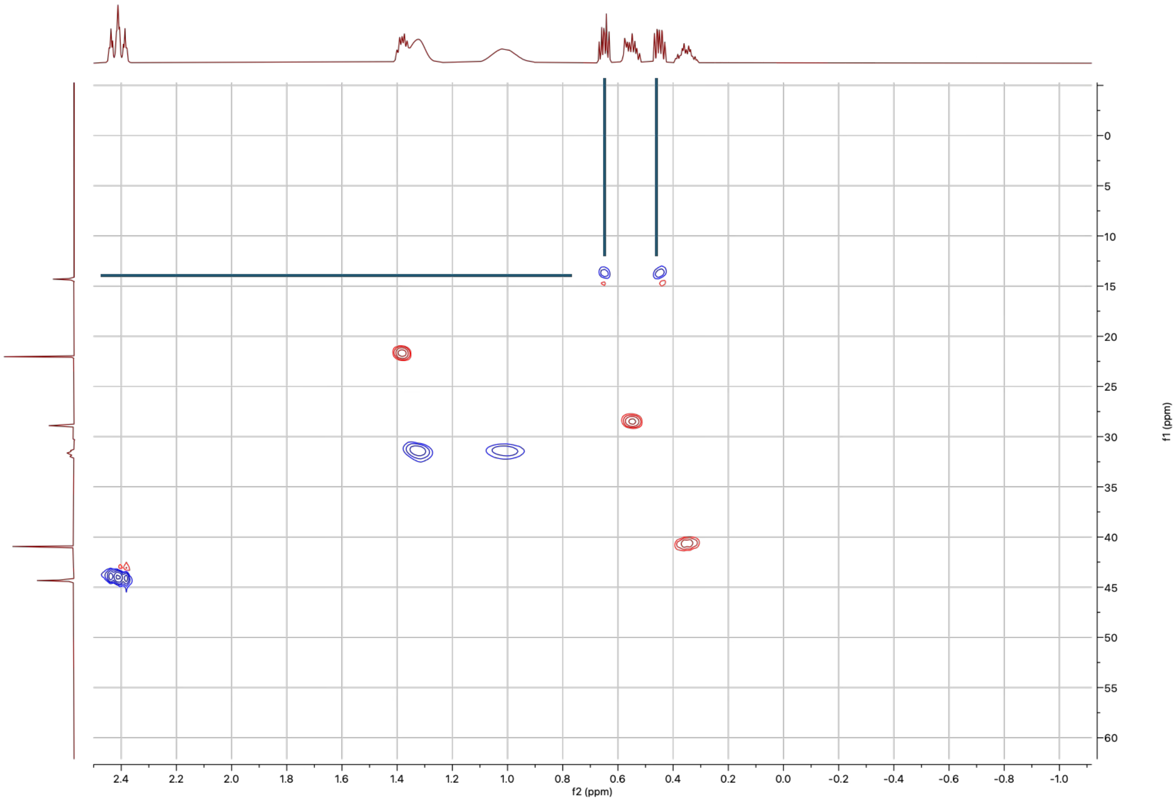

Figure S18. Identification of carbon bearing protons H_b and H_c.

COSY

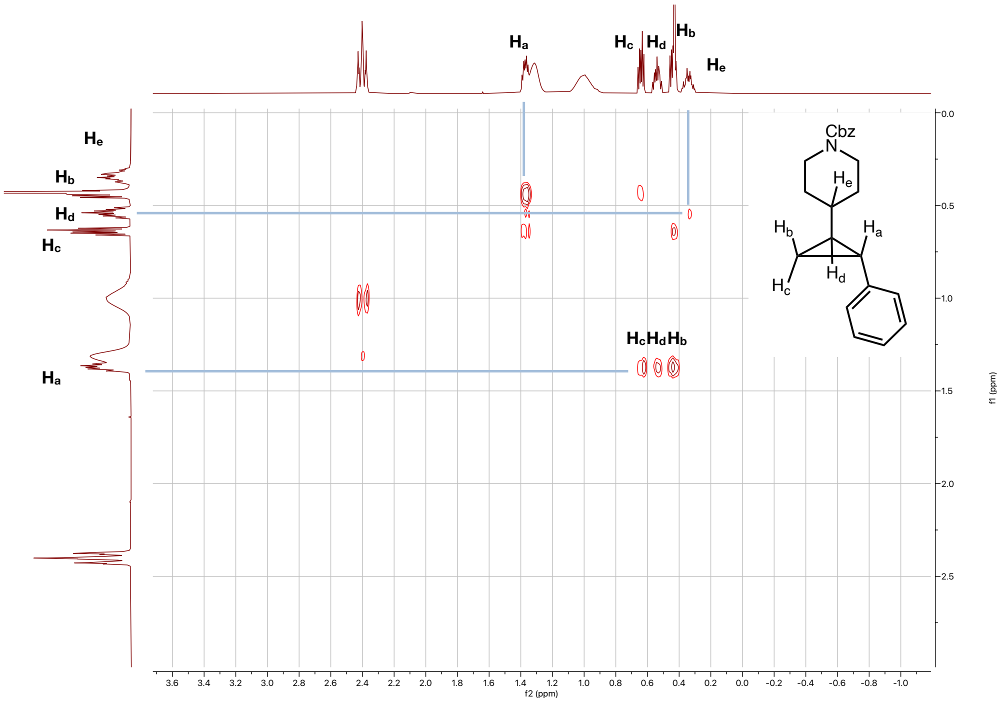

Figure S19. COSY structural analysis data

2D-NOESY

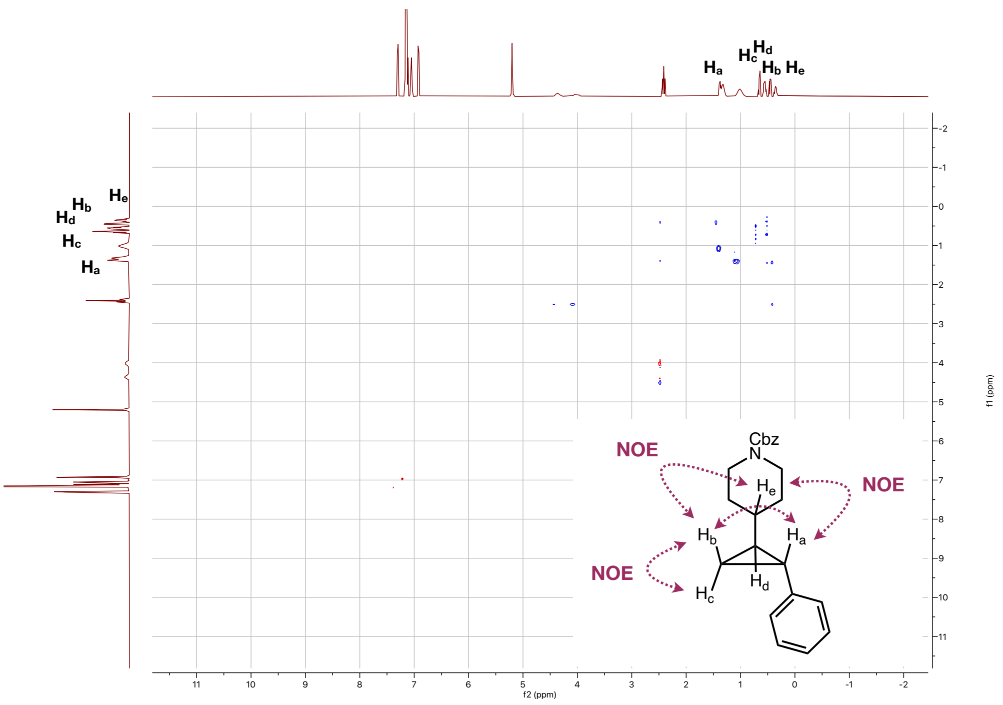

Figure S20. NOESY structural analysis data (zoomed out).

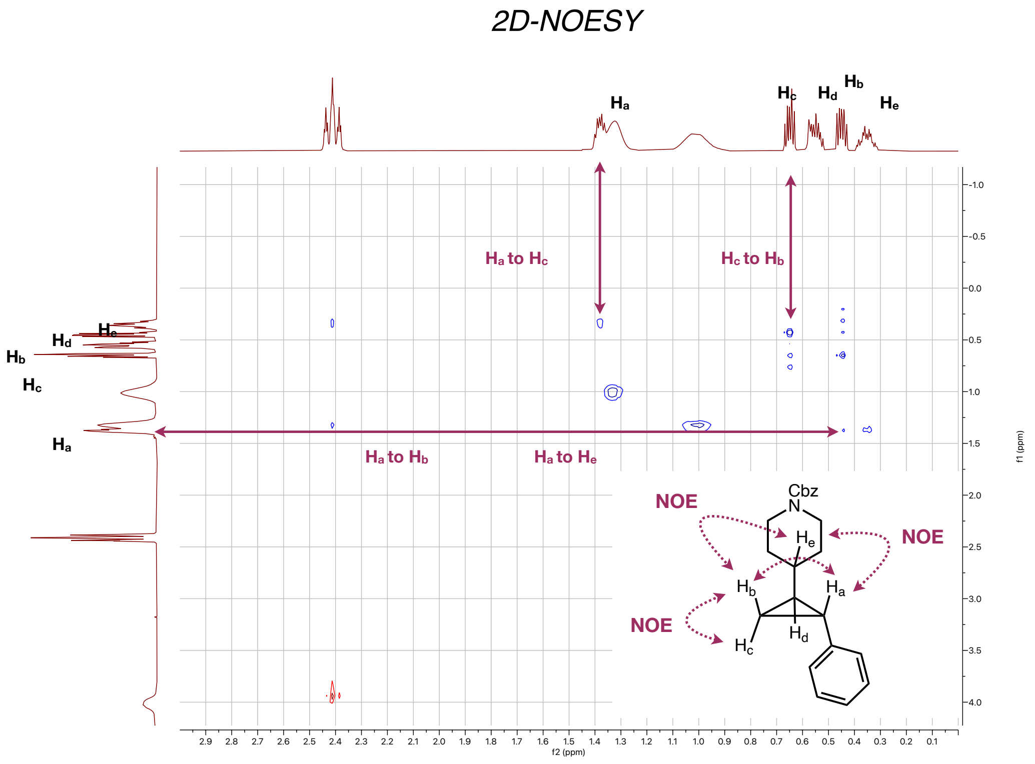

Figure S21. NOESY structural analysis data (zoomed in).

Ring opening

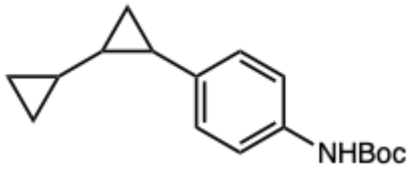

tert-butyl N-[4-(2-cyclopropylcyclopropyl)phenyl]carbamate

Prepared according to the general procedure **A** with cyclopropanecarbaldehyde (37.4 µl, 0.5 mmol, 1.00 equiv), tert-butyl N-(4-vinylphenyl)carbamate (219.3 mg, 1.00 mmol, 2.0 equiv.), 4-CzIPN (3.9 mg, 50 µmol, 0.01 equiv), Fe(OEP)Cl (23.4 mg, 0.04 mmol, 0.075 equiv), imidazole (2.6 mg, 0.075 mmol, 0.05 equiv), **HEH-1** (387 mg, 1.25 mmol, 2.5 equiv), and standard solvent mixture TAA/PhCN/H_2O (1.66 mL). Reaction was sparged for 5 minutes under positive N_2 pressure, then irradiated at 20% light intensity for 16 hours. After irradiation, the solvent was removed under reduced pressure. The crude residue was loaded onto 50 g Biotage Sf.r normal phase column and purified with eluents 0%-100% EtOAc in hexanes. The fractions containing product were concentrated and further purified by preparative HPLC (XBridge BEH C18 OBD column, 20%-100% MeCN in H_2O with 0.1% NH4OH) to furnish product as a white solid (130.1 mg, 475.9 µmol, 95.2% yield, 7:1 dr).

Summary of diastereomers

^{1}H NMR (500 MHz, $CDCl_3$) δ 7.26 – 7.15 (m, 3H), 7.00 – 6.92 (m, 2H), 6.39 (d, *J* = 17.4 Hz, 1H), 2.04 (td, *J* = 8.7, 5.9 Hz, 0.14H), 1.61 (dt, *J* = 8.7, 5.0 Hz, 1H), 1.53 – 1.50 (m, 9H), 1.11 – 1.02 (m, 1H), 0.98 – 0.88 (m, 1H), 0.84 – 0.67 (m, 2H), 0.47 – 0.34 (m, 2H), 0.21 – 0.02 (m, 2H). **^{13}C NMR (126 MHz, $CDCl_3$)** δ 153.00, 138.60, 136.02, 135.77, 134.98, 129.87, 126.31, 118.88, 118.31, 80.45, 28.50, 25.15, 22.48, 21.26, 20.94, 13.53, 12.49, 9.89, 9.71, 4.82, 4.66, 3.46, 2.73.

HRMS (ESI-TOF) *m*/*z* calcd. For $C_{17}H_{23}NO_2Na^+$ ([M+Na^+]$^+$) 296.1621, found 296.1622.

Propiolate Probe:

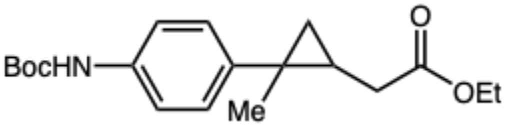

ethyl 2-(2-(4-((*tert*-butoxycarbonyl)amino)phenyl)-2-methylcyclopropyl)acetate

Prepared with ethyl propiolate (76.0 µl, 0.75 mmol, 1.00 equiv), tert-butyl N-(4-isopropenylphenyl)carbamate (116.7 mg, 0.5 mmol, 2.0 equiv.), 4-CzIPN (2.0 mg, 50 µmol, 0.005 equiv), Fe(TPP)Cl (3.53 mg, 0.05 mmol, 0.01 equiv), diethyl 2,6-dimethyl-1,4-dihydropyridine-3,5-dicarboxylate (253.3 mg, 1.00 mmol, 2.0 equiv), and methanol (5.0 ml, 0.1M). Reaction was sparged for 5 minutes under positive N_2 pressure, then irradiated at 10% light intensity for 16 hours. After irradiation, the solvent was removed under reduced pressure. The crude residue was loaded onto 50 g Biotage Sf.r normal phase column and purified with eluents 0%-100% EtOAc in hexanes. The fractions containing product were concentrated and further purified by preparative HPLC (XBridge BEH C18 OBD column, 20%-100% MeCN in H_2O with 0.1% NH4OH) to furnish product as a clear oil (80.1 mg, 240.2 µmol, 48.1% yield, 1.21:1 dr).

Major:
^{1}H NMR (500 MHz, $CDCl_3$) δ 7.28 – 7.24 (m, 4H), 6.39 (d, *J* = 8.7 Hz, 2H), 4.19 (td, *J* = 7.3, 6.6 Hz, 2H), 2.59 (dd, *J* = 16.5, 6.4 Hz, 1H), 2.39 (dd, *J* = 16.5, 8.5 Hz, 1H), 1.51 (s, 9H), 1.33 (s, 3H), 1.29 (t, *J* = 7.1 Hz, 3H), 1.27 – 1.23 (m, 1H), 1.08 (dd, *J* = 8.9, 4.8 Hz, 1H), 0.42 (dd, *J* = 5.8, 4.9 Hz, 1H).
Minor:
^{1}H NMR (500 MHz, $CDCl_3$) δ 7.28 – 7.24 (m, 2H), 7.17 (d, *J* = 8.5 Hz, 2H), 6.39 (d, *J* = 8.7 Hz, 1H), 4.08 (q, *J* = 7.2 Hz, 2H), 2.03 (dd, *J* = 16.7, 6.8 Hz, 1H), 1.79 (dd, *J* = 16.7, 7.6 Hz, 1H), 1.51 (s, 9H), 1.36 (s, 3H), 1.36 – 1.33 (m, 1H), 1.25 – 1.18 (m, 1H), 0.82 (dd, *J* = 8.3, 4.9 Hz, 1H), 0.77 (t, *J* = 5.2 Hz, 1H).
Mixture of Diastereomers:
^{13}C NMR (126 MHz, $CDCl_3$) δ 173.43, 152.87, 137.77, 136.48, 136.06, 129.79, 128.24, 118.66, 118.57, 80.44, 60.44, 60.18, 36.02, 34.79, 30.93, 28.36, 28.14, 25.39, 23.92, 21.19, 21.01, 20.59, 19.45, 17.57, 14.31, 14.22.

HRMS (ESI-TOF) *m*/*z* calcd. For $C_{19}H_{28}NO_4^+$ ($[M+H^+]^+$) 334.2013, found 334.2012

7) Electrochemistry Optimization and Characterization

Optimization:

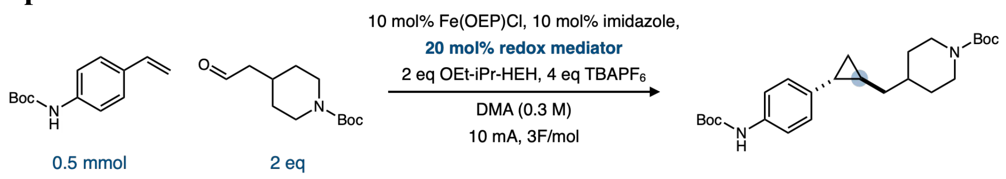

Mediator	Electrodes	Result
No mediator	(+)Cu foam/(-)graphite	28% pdt, 33% styrene
No mediator	(+)graphite/(-)graphite	6% pdt, 43% styrene
No mediator	(+)RVC/(-)RVC	36% pdt, 32% styrene
w/ TPPO	(+)Cu foam/(-)graphite	24% pdt, 28% styrene
w/ TPPO	(+)graphite/(-)graphite	8% pdt, 39% styrene
w/ B_2Cat_2	(+)RVC/(-)RVC	**51% pdt,** 22% styrene
w/ B_2Cat_2	(+)graphite/(-)graphite	1% pdt, 67% styrene
w/ AQ	(+)Ni foam/(-)Pt	24% pdt, 43% styrene
w/ AQ	(+)RVC/(-)RVC	2% pdt, 76% styrene

Table S9. Screening of electrode material and redox mediators

Discussion: We evaluated a variety of electrodes and mediators to optimize reaction performance. Among the electrodes tested, carbon-based materials consistently gave the best yield and reproducibility. Reticulated vitreous carbon (RVC) electrodes led to a more substantial increase in yield compared to graphite. Electrodes were also screened in combination with mediators including B_2cat_2, TPPO, and anthraquinone (AQ). In this context, B_2cat_2 emerged as the most effective mediator, giving the highest yield under otherwise comparable conditions.

■ Electrolyte screening

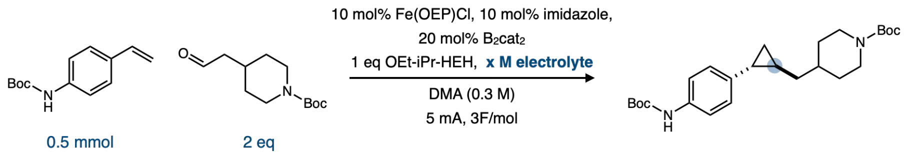

0.2 M KPF_6	63% pdt, 17% styrene	0.5 M TBAOTf	18% pdt, 57% styrene
0.2 M $TBANTf_2$	46% pdt, 44% styrene	0.5 M $NaPF_6$	58% pdt, 16% styrene
0.5 M $TBANTf_2$	56% pdt, 22% styrene	0.5 M $LiClO_4$	43% pdt, 25% styrene
0.5 M $TBAPF_6$	59% pdt, 24% styrene		
0.2 M $TBABPh_4$	60% pdt, 45% styrene		
0.5 M $TBABPh_4$	49% pdt, 24% styrene		

(+)RVC/(-)RVC
Electrasyn IKA Undivided cell

Table S10. Screening of electrolytes and concentration.

Discussion: A range of supporting electrolytes and concentrations (0.2 or 0.5 M) were evaluated to optimize current passage and reaction efficiency. Tetrabutylammonium salts including $TBAPF_6$, $TBANTf_2$, and $TBABPh_4$ afforded good yields, although it was observed that $TBAPF_6$ was not very reproducible and $TBABPh_4$ produced a more complex and less clean reaction profile. In contrast, KPF_6 was found to be the most reproducible and was therefore chosen as the optimal electrolyte.

■ Fe x electrolyte

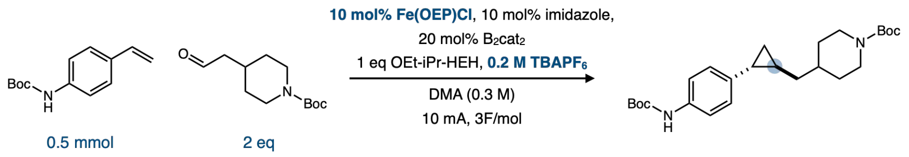

	$TBANTf_2$	$TBABPh_4$
Fe(OEP)Cl	42% pdt, 26% styrene	31% pdt, 49% styrene
Fe(TPP)Cl	1% pdt, 71% styrene	2% pdt, 59% styrene
Fe(TMPP)Cl	10% pdt, 58% styrene	10% pdt, 35% styrene

(+)RVC/(-)RVC
Electrasyn IKA Undivided cell

Table S11. Screening of Fe porphyrin catalyst and electrolyte

Discussion: A variety of iron catalysts were evaluated for the electrochemical system, including Fe(TPP) and Fe(TMPP), but none provided higher yields or improved reproducibility compared with Fe(OEP).

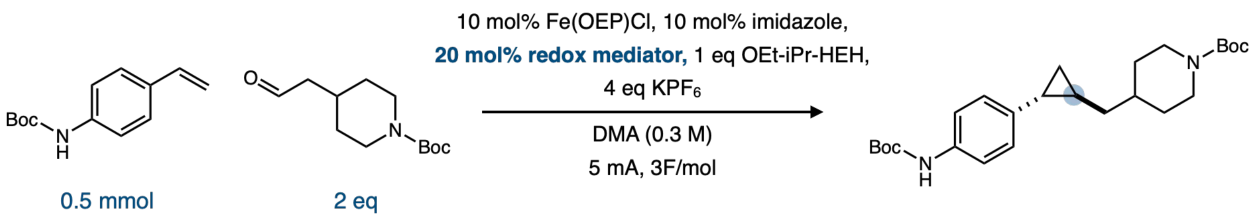

**No mediator**	36% pdt, 32% styrene
**w/ B_2Cat_2**	53% pdt, 26% styrene
**w/ B_2Cat_2, 20 mol% boric acid, 1 eq water**	**61% pdt,** 22% styrene

Table S12. Water and boric acid additive screen

Discussion: The inclusion of boric acid and water in the reaction mixture improved yields of the desired product, indicating that additional protons facilitate a critical step in carbene generation. This effect is consistent with proton-assisted α-elimination, in which protonation of the hydroxyl group on the aldehyde-derived intermediate promotes water loss and formation of the carbene equivalent.

■ Other metal catalysts tried

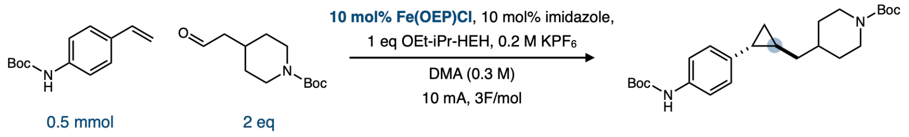

Fe(acac)$_2$	Co(TMPP)	Fe(acac)$_2$	
Fe(acac)$_3$	Co(TPP)	Fe(acac)$_3$	**No product in all cases**<br>Significant styrene RSM<br>HEH mostly all oxidized
$Cp_2Fe(CO)_4$	Co(OEP)	$Cp_2Fe(CO)_4$	
[Fe(III)(Salpen(tBu))]Cl	Vit B12	[Fe(III)(Salpen(tBu))]Cl	
[Fe(III)(Salen(tBu))]Cl	Co(Pc)	[Fe(III)(Salen(tBu))]Cl	
	Mn(TPP)Cl		

(+)RVC/(-)RVC
Electrasyn IKA Undivided cell

Table S13. Other metal catalysts screened

Discussion: In addition to iron porphyrin catalysts, other transition metal complexes, including cobalt and manganese porphyrins, were evaluated but failed to promote carbene formation under the reaction conditions.

Competition experiment

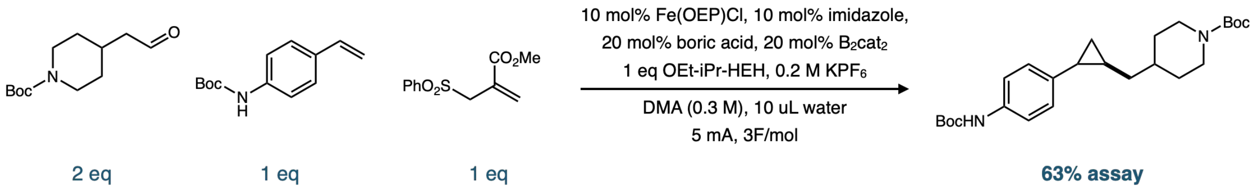

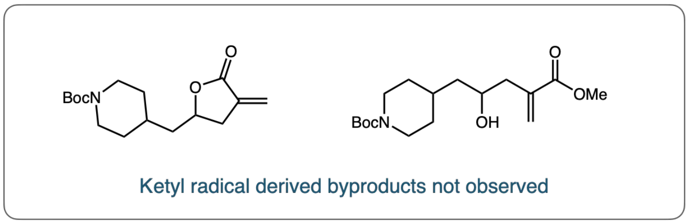

Figure S22. Competition experiment: hypothesized ketyl radical products not observed

Prepared according to General Procedure F. An oven dried ElectraSyn vial (5.0 mL) with a magnetic stir bar was charged with *tert*-butyl (4-vinylphenyl)carbamate (1.0 equiv, 0.5 mmol), iPr-HEH (1.0 equiv, 0.5 mmol), *tert*-butyl 4-(2-oxoethyl)piperidine-1-carboxylate (2.0 equiv, 1.0 mmol), methyl 2-((phenylsulfonyl)methyl)acrylate (0.5 mmol, 1.0 equiv.), imidazole (0.1 equiv, 0.05 mmol), Fe(OEP)Cl (0.1 equiv, 0.05 mmol), B_2cat_2 (0.2, 1.0 mmol), KPF_6 (0.2 M), water

(10 µL, 1 equiv, 0.5 mmol), boric acid (0.20 equiv, 0.10 mmol), and DMA (1.6 mL, 0.3 M). The electrasyn vial cap equipped with RVC foam was inserted into the vial containing the reaction mixture. The vial was allowed to stir for 2 minutes and sparged under constant nitrogen flow through a thin black needle in the septa. The reaction was carried out under room temperature. The vial was connected to the ElectraSyn, and the ElectraSyn was set up as follows: New exp. > Constant current > 5 mA > No ref. electrode > Total charge > 0.5 mmol, 3.0 F/mol > No alternating polarity > Start.

After electrolysis, the crude reaction mixture was analayzed using uHPLC, with mesitylene (0.5 eq) as the standard.

Assay yield for the Cyclopropane product: 63% by uHPLC.

Standard (Mesitylene) retention time= 4.321 mins (Integration area = 372)
Product retention time= 5.019 mins (Integration area = 474)
63% assay yield

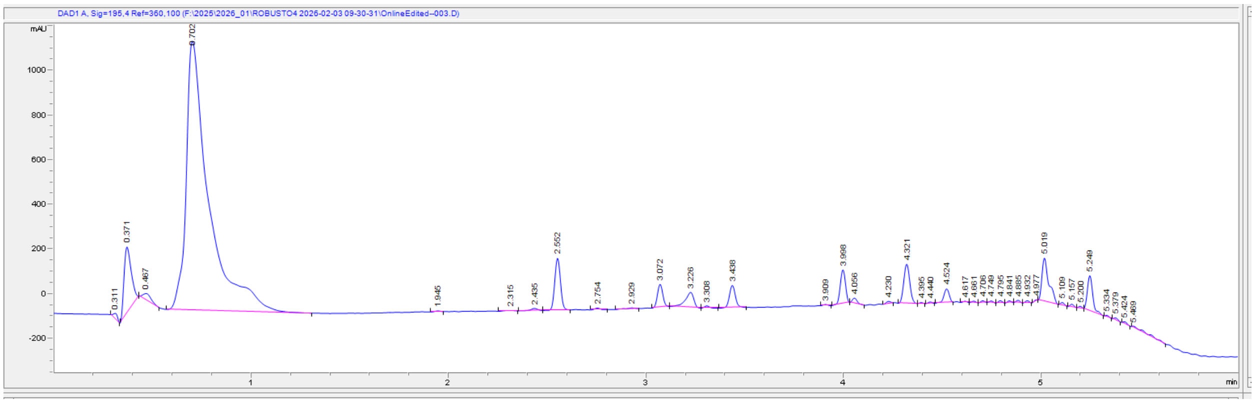

***tert*-butyl 4-((2-(4-((*tert*-butoxycarbonyl)amino)phenyl)cyclopropyl)methyl)piperidine-1-carboxylate**

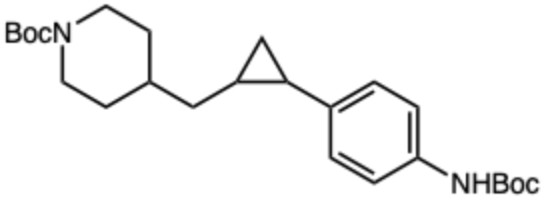

Gram scale synthesis

Prepared according to the general procedure **F** with tert-butyl N-(4-vinylphenyl)carbamate (877 mg, 4.0 mmol, 1.0 equiv), N-Boc-4-piperidineacetaldehyde (1.82 g, 8.00 mmol, 2.00 equiv), Fe(OEP)Cl (249.6 mg, 0.40 mmol, 0.10 equiv), imidazole (27.2 mg, 0.4 mmol, 0.10 equiv), **HEH-1** (1.24 g, 4.00 mmol, 1.00 equiv), B_2cat_2 (190.3 mg, 0.80 mmol, 0.20 equiv), KPF_6 (236 mg, 1.28 mmol, 0.32 equiv, 0.2 M), boric acid (49.5 mg, 0.8 mmol, 0.20 equiv), water (80 µL, 4.40 mmol, 1.10 equiv) and DMA (6.4 mL). The reaction was carried out under room temperature using an

Electrasyn 2.0 and the vials were under positive N_2 pressure. The Electrasyn settings were 35 mA, 3F/mol, and 1000 stirring. After electrolysis, the solvent was removed under reduced pressure. The crude material was redissolved in acetonitrile, then filtered through a 10 Micron Polyethylene Frit or a plug of celite. The filtrate was concentrated by rotary evaporation and then loaded onto 50 g Biotage Sf.r normal phase column and purified with eluents 0%-100% EtOAc in hexanes followed by preparative HPLC (XBridge BEH C18 OBD column, 40%-100% MeCN in H_2O with 0.1% NH_4OH) to furnish product as a off white amorphous solid (1.012 g, 2.35 mmol, 58% yield (3:1 d.r.)).

^{1}H NMR (500 MHz, Chloroform-*d*) δ 7.25 – 7.19 (m, 2H), 7.06 (d, *J* = 8.4 Hz, 0.6H), 6.96 – 6.90 (m, 1.7H), 6.66 – 6.48 (m, 1H), 4.28 – 3.88 (m, 2H), 2.69 – 2.58 (m, 2H), 2.02 (td, *J* = 8.6, 5.9 Hz, 0.25H), 1.74 – 1.60 (m, 2H), 1.57 – 1.47 (m, 2H), 1.50 (s, 3H), 1.49 (s, 6H), 1.44 (s, 6H), 1.43 (s, 3H), 1.41 – 0.88 (m, 6H), 0.84 (dt, *J* = 8.4, 4.9 Hz, 1H), 0.69 (dt, *J* = 8.7, 5.4 Hz, 1H), 0.57 (q, *J* = 5.5 Hz, 0.27H).

^{13}C NMR (126 MHz, Chloroform-*d*) δ 154.97, 153.03, 152.98, 138.37, 136.20, 135.88, 134.05, 129.50, 126.02, 118.94, 118.25, 80.30, 79.23, 79.17, 45.55 – 42.71 (m), 41.51, 36.77, 36.53, 35.30, 32.34, 32.20, 28.56, 28.44, 22.99, 21.35, 20.04, 16.54, 15.87, 10.08.

HRMS (ESI-TOF) *m*/*z* calcd. For $C_{21}H_{30}N_2O_4Na^+$ ([M-iBu+H]$_+$) 375.2278, found 375.2280.

benzyl 4-(2-(4-fluorophenyl)cyclopropyl)piperidine-1-carboxylate

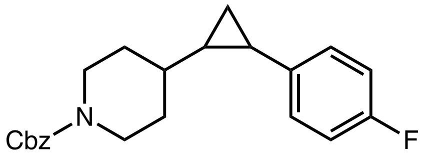

Prepared according to the general procedure **F** with 1-fluoro-4-vinyl-benzene (60 µL, 0.50 mmol, 1.09 equiv), benzyl 4-formylpiperidine-1-carboxylate (247 mg, 1.00 mmol, 2.00 equiv), Fe(OEP)Cl (31.2 mg, 0.05 mmol, 0.10 equiv), imidazole (3.4 mg, 0.05 mmol, 0.10 equiv), **HEH-1** (155 mg, 0.50 mmol, 1.00 equiv), B_2Cat_2 (23.8 mg, 0.10 mmol, 0.20 equiv), KPF_6 (59.0 mg, 0.321 mmol, 0.64 equiv, 0.2 M), boric acid (6.2 mg, 0.10 mmol, 0.20 equiv), water (10 µL, 0.5 mmol, 1.00 equiv) and DMA (1.66 mL). The reaction was carried out under room temperature using an Electrasyn 2.0 and the vials were under positive N_2 pressure. The Electrasyn settings were 5 mA, 3F/mol, and 500 stirring. After electrolysis, 30 µL of 1,4-difluorobenzene was added into the vial via syringe. The assay yield was determined using ^{19} F NMR based on authentic product.

25% assay yield

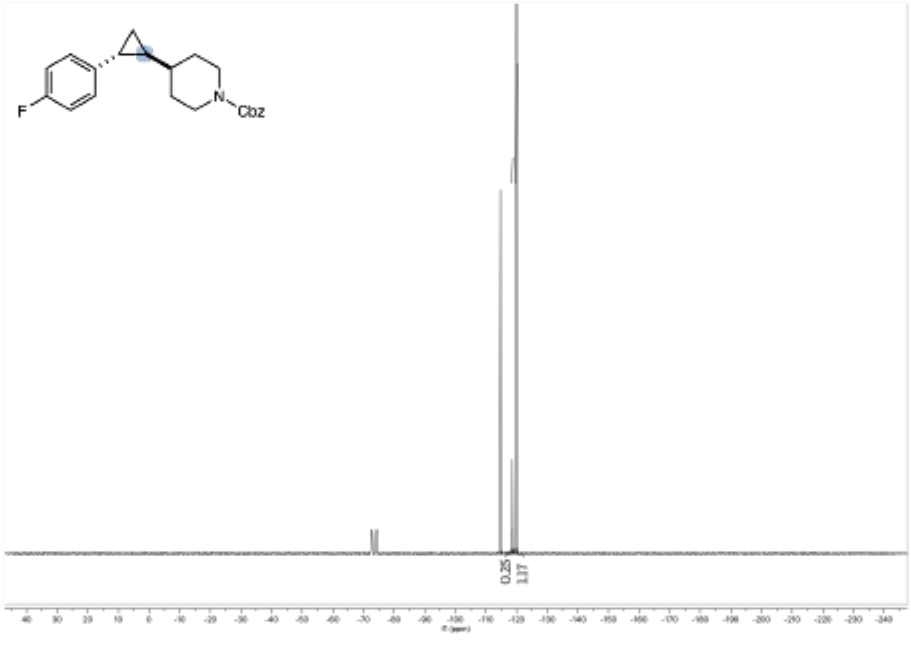

***tert*-butyl 4-(((1*S*,2*S*)-2-(4-fluorophenyl)-2-methylcyclopropyl)methyl)piperidine-1-carboxylate**

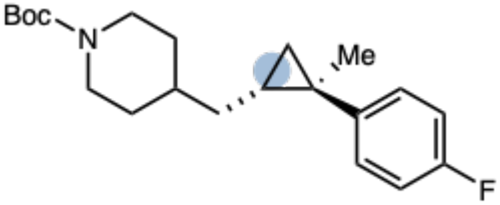

Prepared according to the general procedure **F** with 1-fluoro-4-isopropenyl-benzene (67 µL, 0.50 mmol, 1.09 equiv), N-Boc-4-piperidineacetaldehyde (227 mg, 1.00 mmol, 2.00 equiv), Fe(OEP)Cl (31.2 mg, 0.05 mmol, 0.10 equiv), imidazole (3.4 mg, 0.05 mmol, 0.10 equiv), **HEH-1** (155 mg, 0.50 mmol, 1.00 equiv), B_2cat_2 (23.8 mg, 0.10 mmol, 0.20 equiv), KPF6 (59.0 mg, 0.321 mmol, 0.64 equiv, 0.2 M), boric acid (6.2 mg, 0.10 mmol, 0.20 equiv), water (10 µL, 0.5 mmol, 1.00 equiv) and DMA (1.66 mL). The reaction was carried out under room temperature using an Electrasyn 2.0 and the vials were under positive N_2 pressure. The Electrasyn settings were 5 mA, 3F/mol, and 500 stirring. After electrolysis, 30 µL of 1,4-difluorobenzene was added into the vial via syringe. The assay yield was determined using 19 F NMR based on authentic product.

55% assay, 1.1:1 d.r.

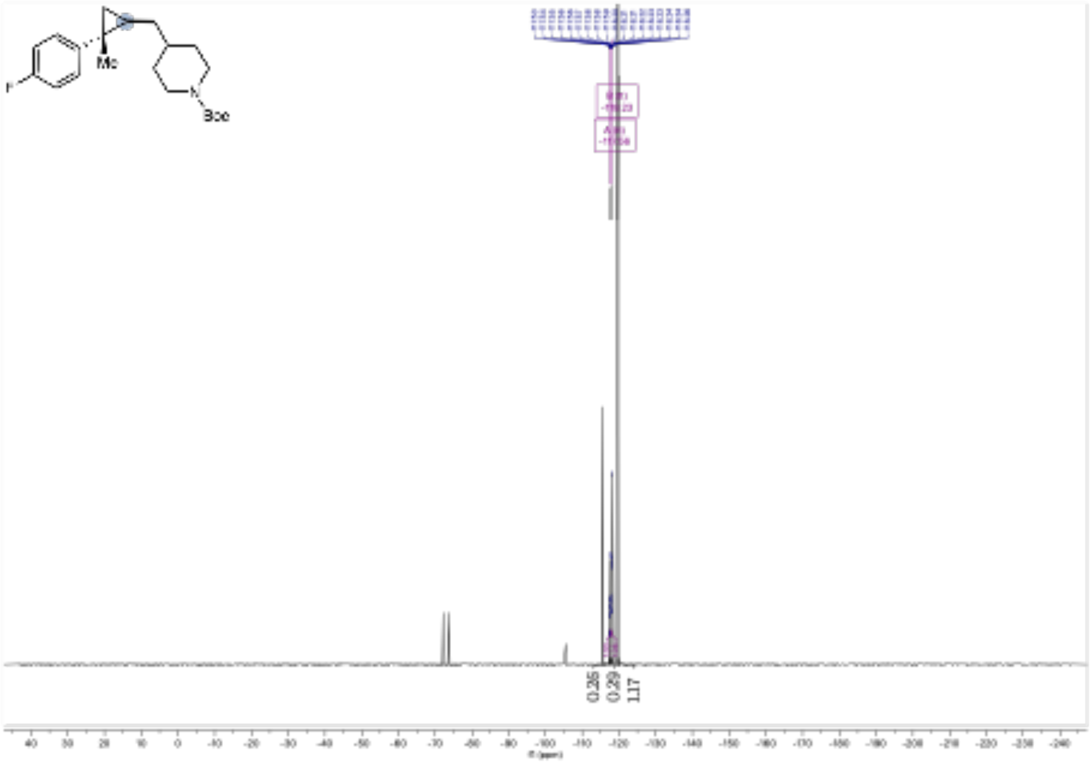

8) Effect of π-nucleophilicity on yield of reaction

Prepared according to the general procedure **C** with N-Boc-4-piperidineacetaldehyde precursor (22.7 mg, 0.10 mmol, 1.00 equiv), olefin (0.50 mmol, 5.00 equiv), Ir(dF(Me)ppy)$_2$(dtbppy)PF$_6$ (2.0 mg, 0.02 μmol, 0.02 equiv), Fe(OEP)Cl (4.7 mg, 0.0075 mmol, 0.075 equiv), Imidazole (0.5 mg, 0.0075 mmol, 0.075 equiv), **HEH-1** (77.4 mg, 0.25 mmol, 2.5 equiv), and standard solvent mixture TAA/PhCN/H_2O (0.33 mL). Reaction was sparged for 5 minutes under positive N_2 pressure, then irradiated at 50% light intensity for 24 hours. After irradiation, mesitylene was added (10 μl, 0.71 mmol) and a crude ^{1}H-NMR was taken and UPLC-MS. Product yield was based on UPLC-MS a calibration curve in tandem with ^{1}H-NMR vs. Mesitylene.

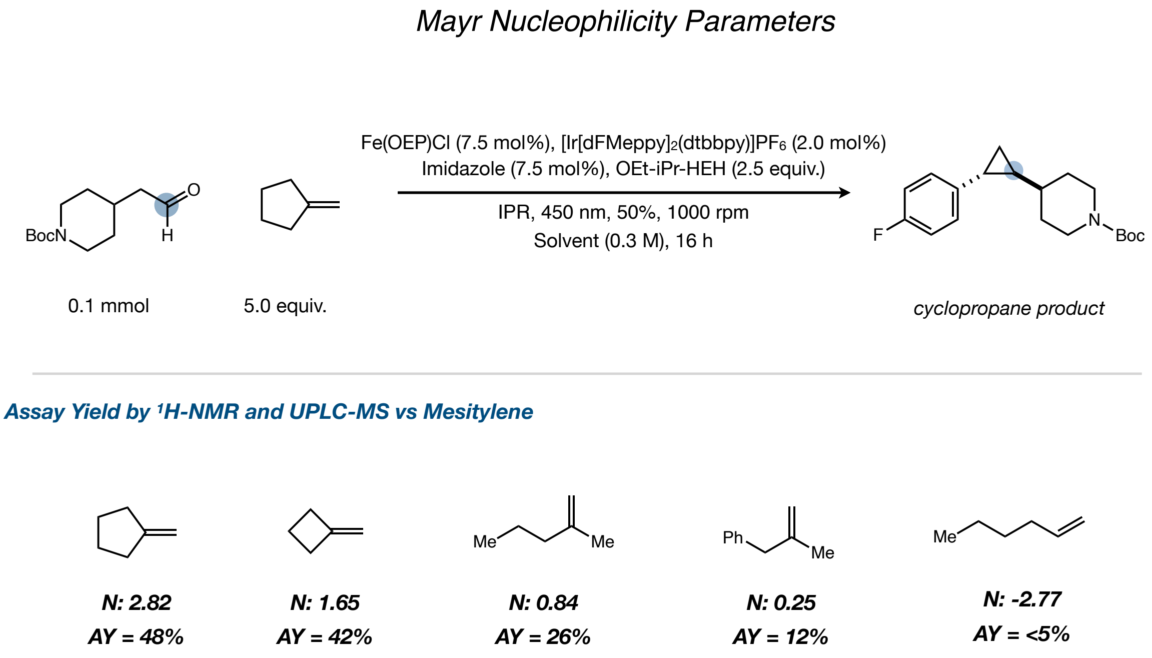

Figure S23. Effect of π-nucleophilicity on yield of reaction.[7,8]

9) References

1. Isomura, M., Petrone, D. A., Carreira, E. M. Coordination-Induced Stereocontrol over Carbocations: Asymmetric Reductive Deoxygenation of Racemic Tertiary Alcohols. *J. Am. Chem. Soc.* **2024**, *146* (48), 32925–32932.
2. Ryoo, H., Keum, H., Kim, D., Chang, S. Harnessing Bifunctional N-Benzoyloxyamides or Photoredox Amidative Dual Functionalizations of Alkenes. *Angew. Chem. Int. Ed.* **2025**, *64* (32), 1–7.
3. Ryabchuk, P., Stier, K., Junge, K., Checinski, M. P., Beller, M., Molecularly Defined Manganese Catalyst for Low-Temperature Hydrogenation of Carbon Monoxide. *J. Am. Chem. Soc.* **2019**, *141* (42), 16923–16929
4. Garreau, M., Le Vaillant, F., Waser, J. C-Terminal Bioconjugation of Peptides through Photoredox Catalyzed Decarboxylative Alkynylation. *Angew. Chem. Int. Ed.* **58** (24), 8182–8186.
5. Crawford, J. J., Daohong, L., Kolesnikov, A., Lee, W., Landry, M. L. Synthesis, **2020**, *52* (22), 3420–3426.
6. Pace, A. L.; Xu, F.; Liu, W.; Lavagnino, M. N.; MacMillan, D. W. C. Iron-Catalyzed Cross-Electrophile Coupling for the Formation of All-Carbon Quaternary Centers. *J. Am. Chem. Soc.* **2024**, *146* (48), 32925–32932.
7. Mayr, H.; Bug, T.; Gotta, M. F.; Hering, N.; Irrgang, B.; Janker, B.; Kempf, B.; Loos, R.; Ofial, A.R.; Remennikov, G.; Schimmel, H. Reference scales for the characterization of cationic electrophiles and neutral nucleophiles. *J. Am. Chem. Soc.* **2001**, *123* (39), 9500-9512.
8. Ammer, J.; Nolte, C.; & Mayr, H. Free Energy Relationships for Reactions of Substituted Benzhydrylium Ions: From Enthalpy over Entropy to Diffusion Control. *J. Am. Chem. Soc.* **2012**, *134* (33), 9500-9512.

10) Spectral Data

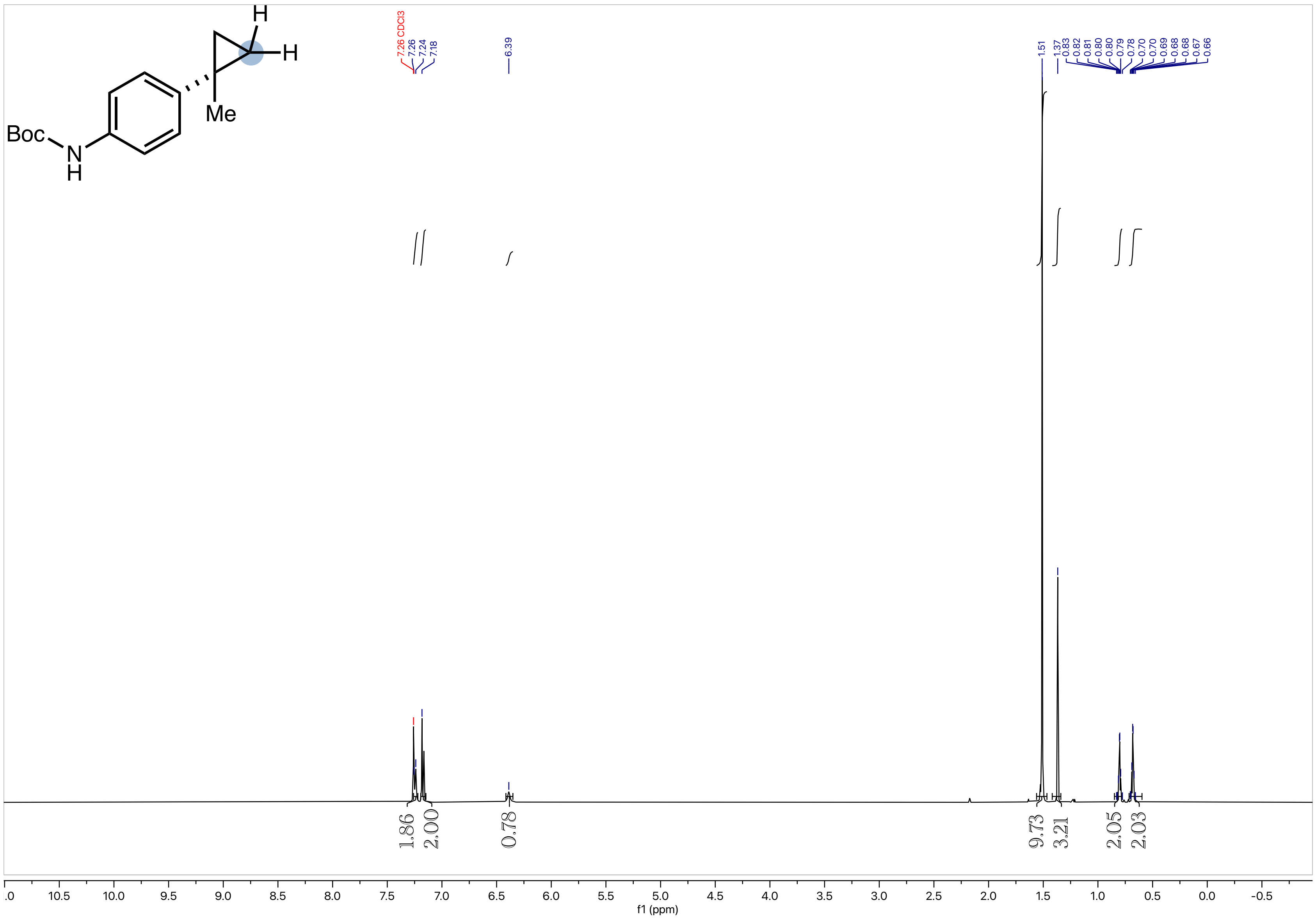

Boc
N
H
Me
H
H
7.26 CDCl3
7.26
7.24
7.18
6.39
1.51
1.37
0.83
0.82
0.81
0.80
0.80
0.79
0.78
0.70
0.70
0.69
0.68
0.68
0.67
0.66
1.86
2.00
0.78
9.73
3.21
2.05
2.03
f1 (ppm)

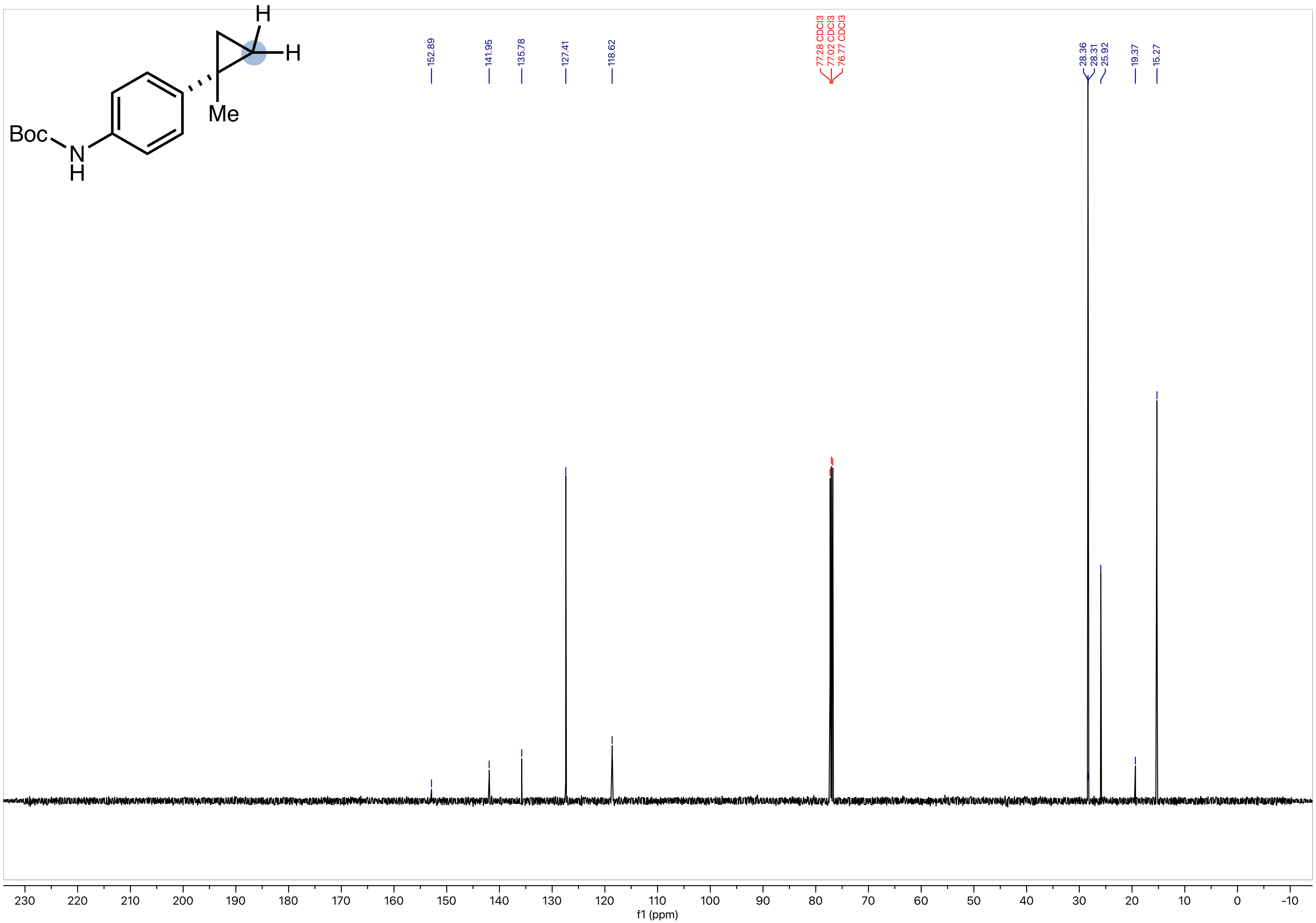

Boc
N
H
Me
H
H
152.89
141.95
135.78
127.41
118.62
77.28 CDCl3
77.02 CDCl3
76.77 CDCl3
28.36
28.31
25.92
19.37
15.27
230 220 210 200 190 180 170 160 150 140 130 120 110 100 90 80 70 60 50 40 30 20 10 0 -10
f1 (ppm)

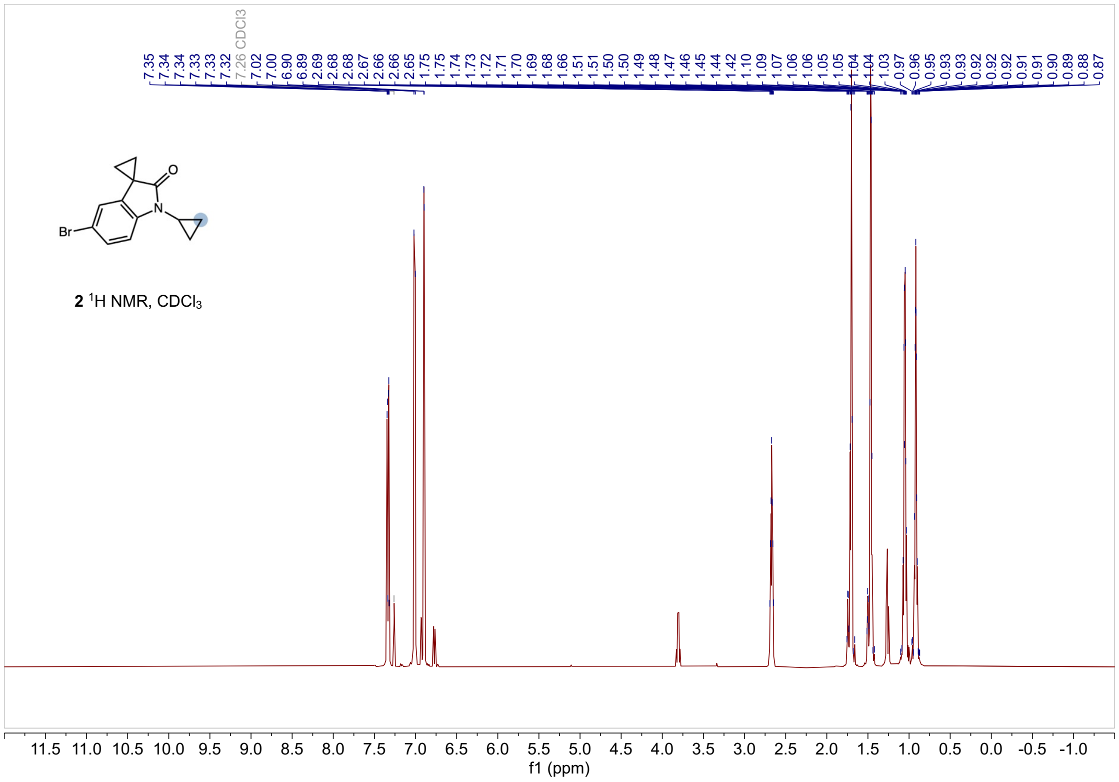

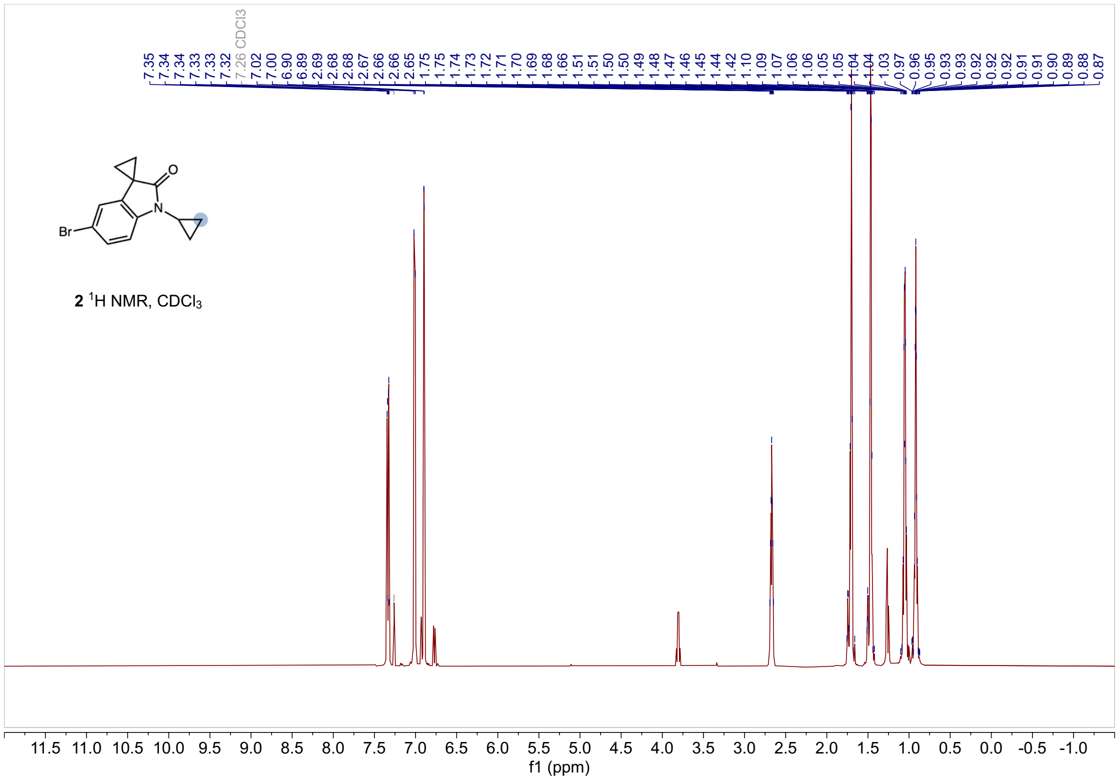
7.35 7.34 7.34 7.33 7.33 7.32 7.26 CDCl3 7.02 7.00 6.90 6.89 2.69 2.68 2.68 2.67 2.66 2.66 2.65 1.75 1.75 1.74 1.73 1.72 1.71 1.70 1.69 1.68 1.66 1.51 1.51 1.50 1.50 1.49 1.48 1.47 1.46 1.45 1.44 1.42 1.10 1.09 1.07 1.06 1.06 1.05 1.05 1.04 1.04 1.03 0.97 0.96 0.95 0.93 0.93 0.92 0.92 0.92 0.91 0.91 0.90 0.89 0.88 0.87
O
N
Br
2 ¹H NMR, CDCl3
11.5 11.0 10.5 10.0 9.5 9.0 8.5 8.0 7.5 7.0 6.5 6.0 5.5 5.0 4.5 4.0 3.5 3.0 2.5 2.0 1.5 1.0 0.5 0.0 -0.5 -1.0
f1 (ppm)

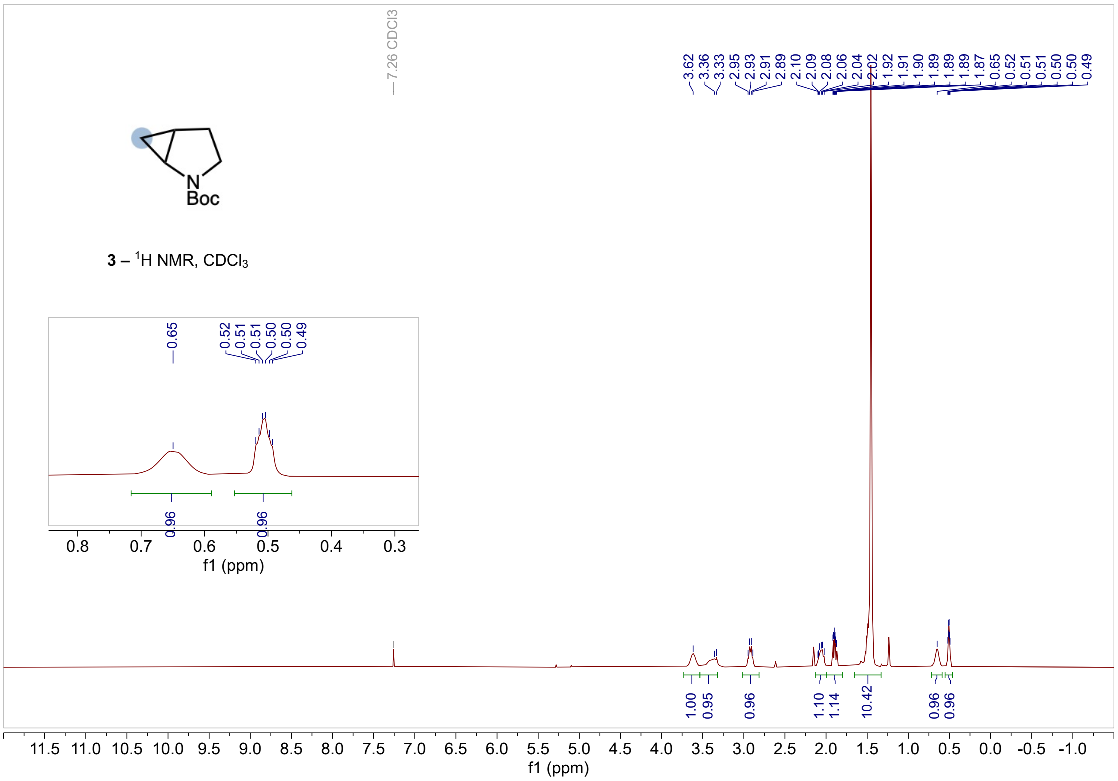
Boc
N
3 – ^{1}H NMR, $CDCl_3$
7.26 CDCl3
3.62
3.36
3.33
2.95
2.93
2.91
2.89
2.10
2.09
2.08
2.06
2.04
2.02
1.92
1.91
1.90
1.89
1.89
1.87
0.65
0.52
0.51
0.51
0.50
0.50
0.49
1.00
0.95
0.96
1.10
1.14
10.42
0.96
0.96
0.8 0.7 0.6 0.5 0.4 0.3
f1 (ppm)
11.5 11.0 10.5 10.0 9.5 9.0 8.5 8.0 7.5 7.0 6.5 6.0 5.5 5.0 4.5 4.0 3.5 3.0 2.5 2.0 1.5 1.0 0.5 0.0 -0.5 -1.0
f1 (ppm)

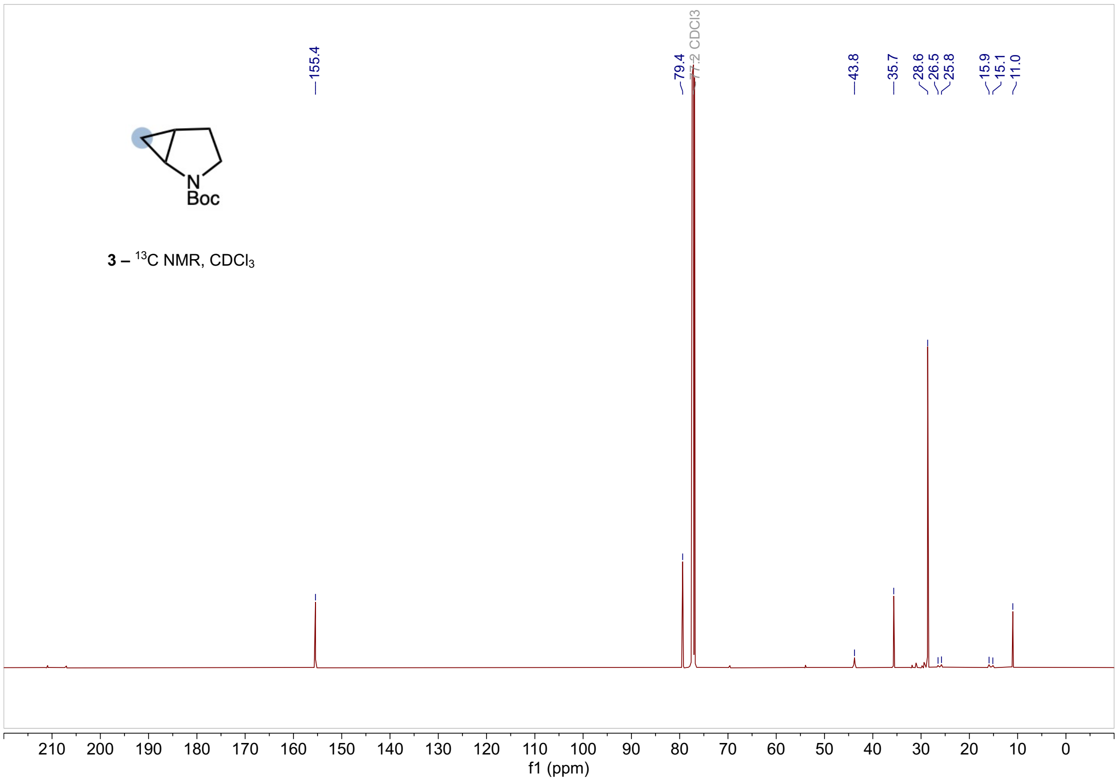
Boc
N
3 – ^{13}C NMR, $CDCl_3$
155.4
79.4
77.2 CDCl3
43.8
35.7
28.6
26.5
25.8
15.9
15.1
11.0
210 200 190 180 170 160 150 140 130 120 110 100 90 80 70 60 50 40 30 20 10 0
f1 (ppm)

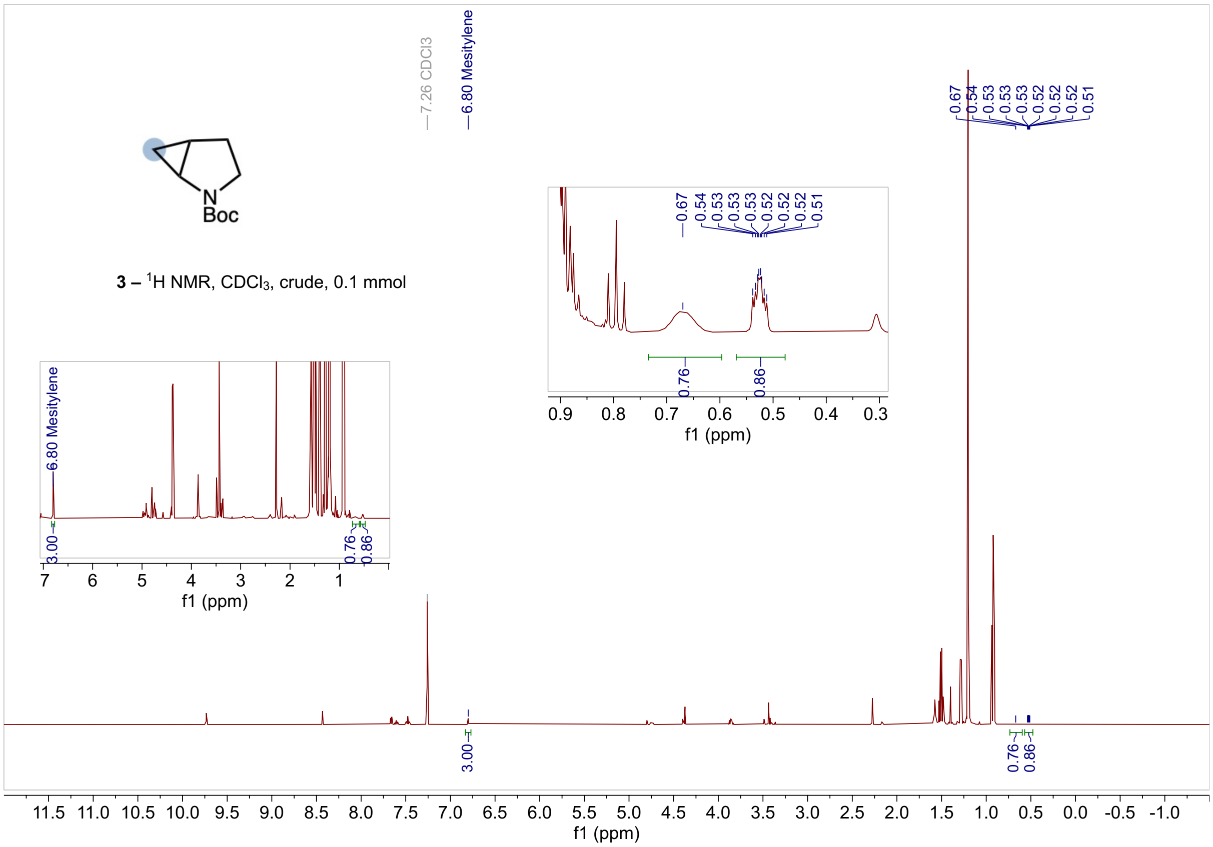

N
Boc
3 – 1H NMR, CDCl3, crude, 0.1 mmol
7.26 CDCl3
6.80 Mesitylene
0.67
0.54
0.53
0.53
0.53
0.52
0.52
0.52
0.51
3.00
0.76
0.86
f1 (ppm)

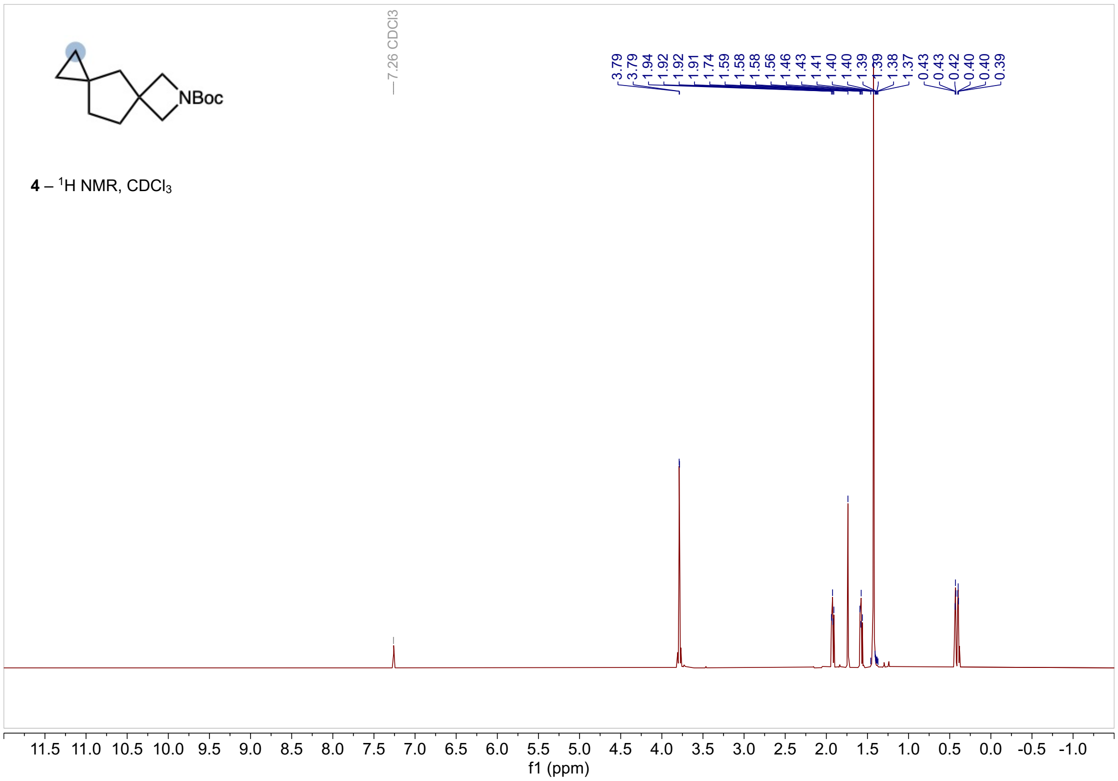

4 – ^{1}H NMR, $CDCl_3$

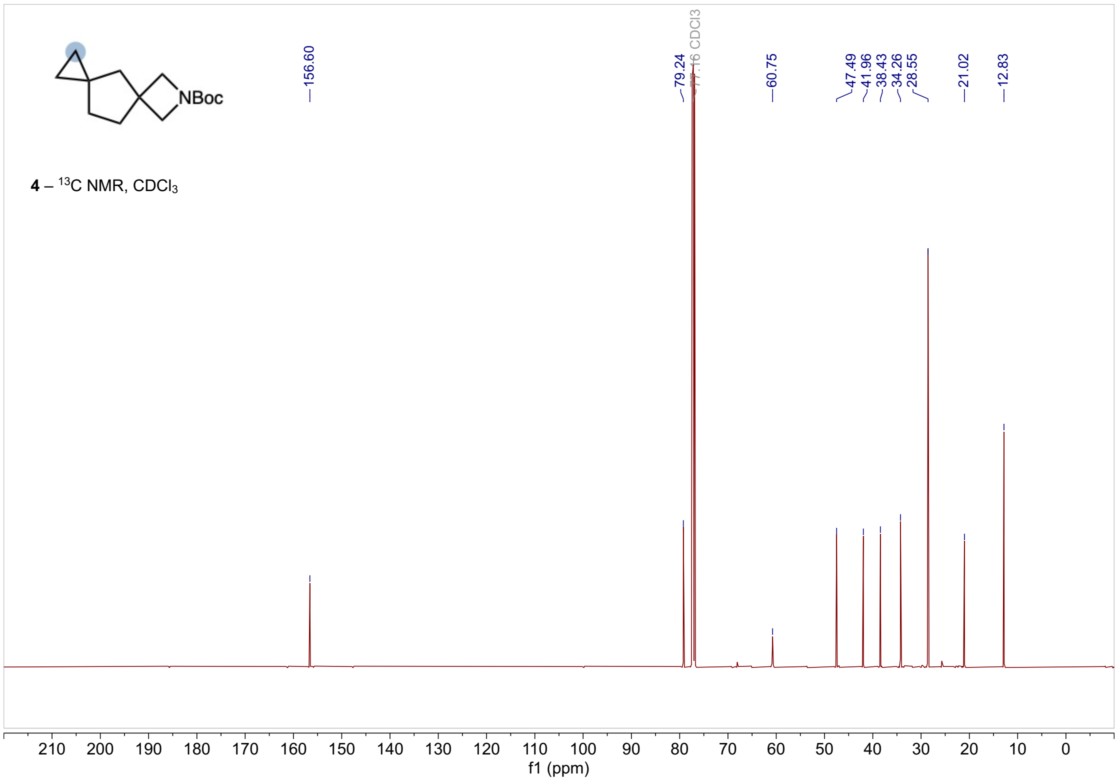

4 – ^{13}C NMR, $CDCl_3$

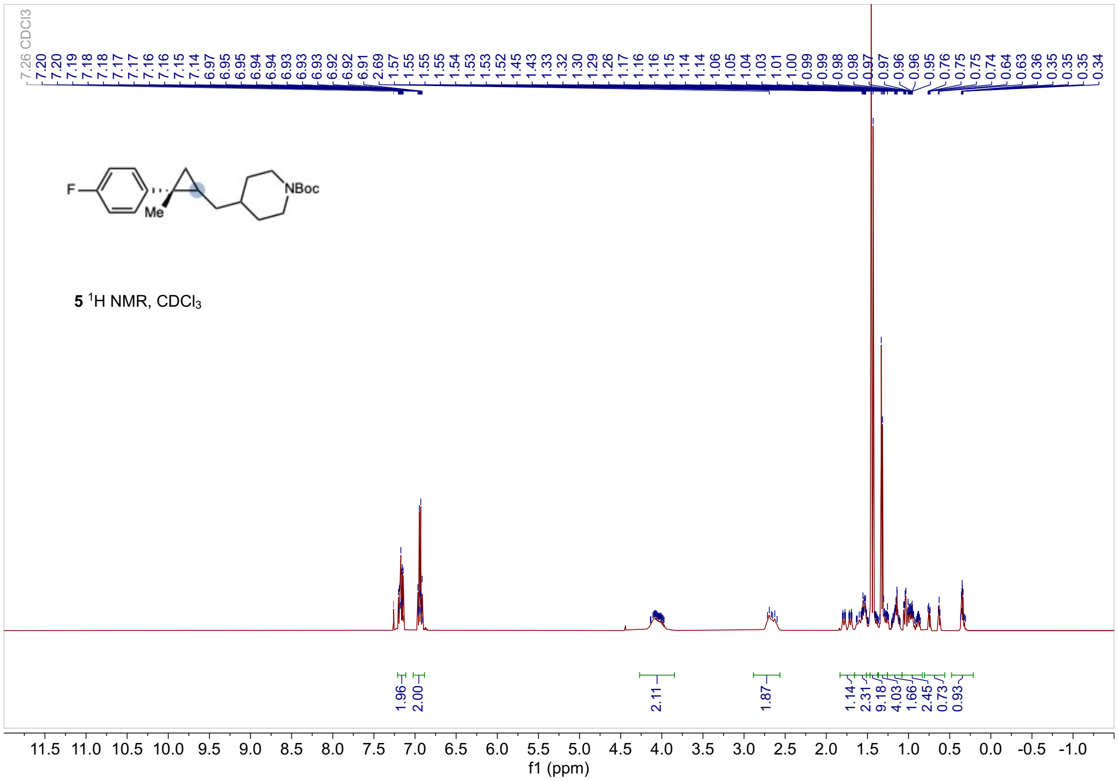

7.26 CDCl3
7.20 7.20 7.19 7.18 7.18 7.17 7.16 7.16 7.15 7.14 6.97 6.95 6.95 6.94 6.93 6.93 6.93 6.92 6.92 6.91 2.69 1.57 1.55 1.55 1.54 1.53 1.53 1.52 1.45 1.43 1.33 1.32 1.30 1.29 1.26 1.17 1.16 1.16 1.15 1.14 1.14 1.06 1.05 1.04 1.03 1.01 1.00 0.99 0.99 0.98 0.98 0.97 0.97 0.96 0.96 0.95 0.76 0.75 0.75 0.74 0.64 0.63 0.36 0.35 0.35 0.34
F
Me
NBoc
5 ¹H NMR, CDCl3
1.96 2.00 2.11 1.87 1.14 2.31 9.18 4.03 1.66 2.45 0.73 0.93
11.5 11.0 10.5 10.0 9.5 9.0 8.5 8.0 7.5 7.0 6.5 6.0 5.5 5.0 4.5 4.0 3.5 3.0 2.5 2.0 1.5 1.0 0.5 0.0 -0.5 -1.0
f1 (ppm)

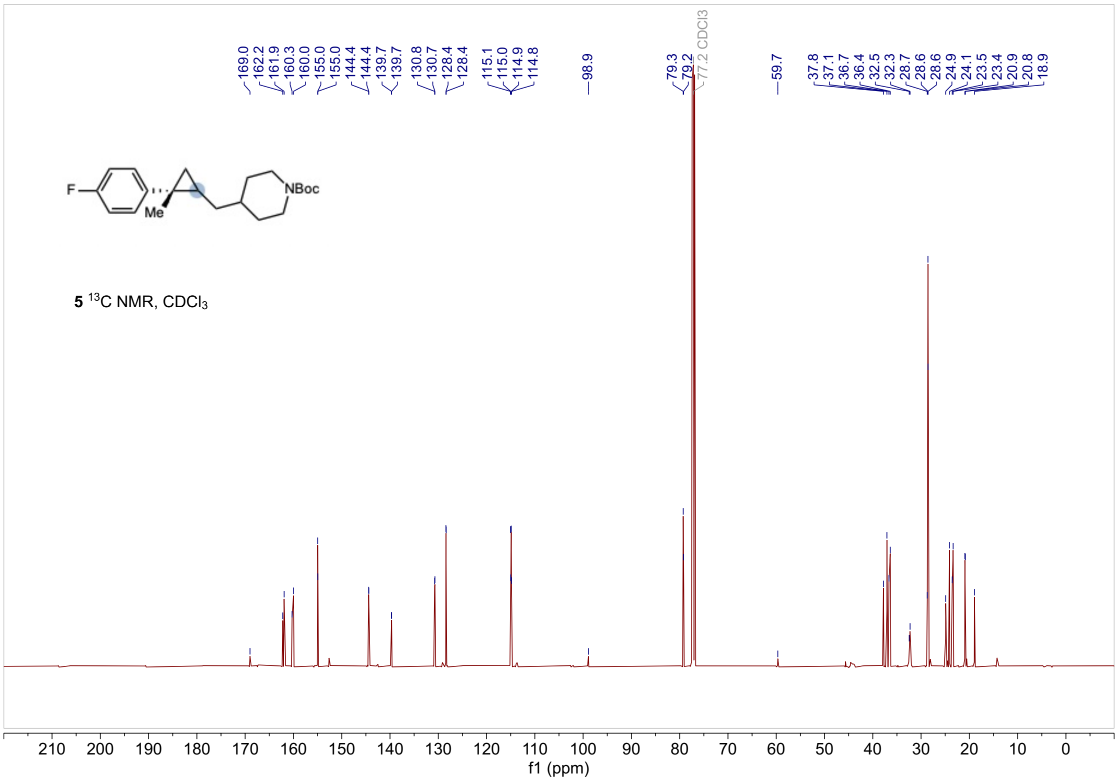

169.0 162.2 161.9 160.3 160.0 155.0 144.4 144.4 139.7 139.7 130.8 130.7 128.4 128.4 115.1 115.0 114.9 114.8
98.9
79.3 79.2 77.2 CDCl3
59.7
37.8 37.1 36.7 36.4 32.5 32.3 28.7 28.6 28.6 24.9 24.1 23.5 23.4 20.9 20.8 18.9
F
Me
NBoc
5 ¹³C NMR, CDCl3
210 200 190 180 170 160 150 140 130 120 110 100 90 80 70 60 50 40 30 20 10 0
f1 (ppm)

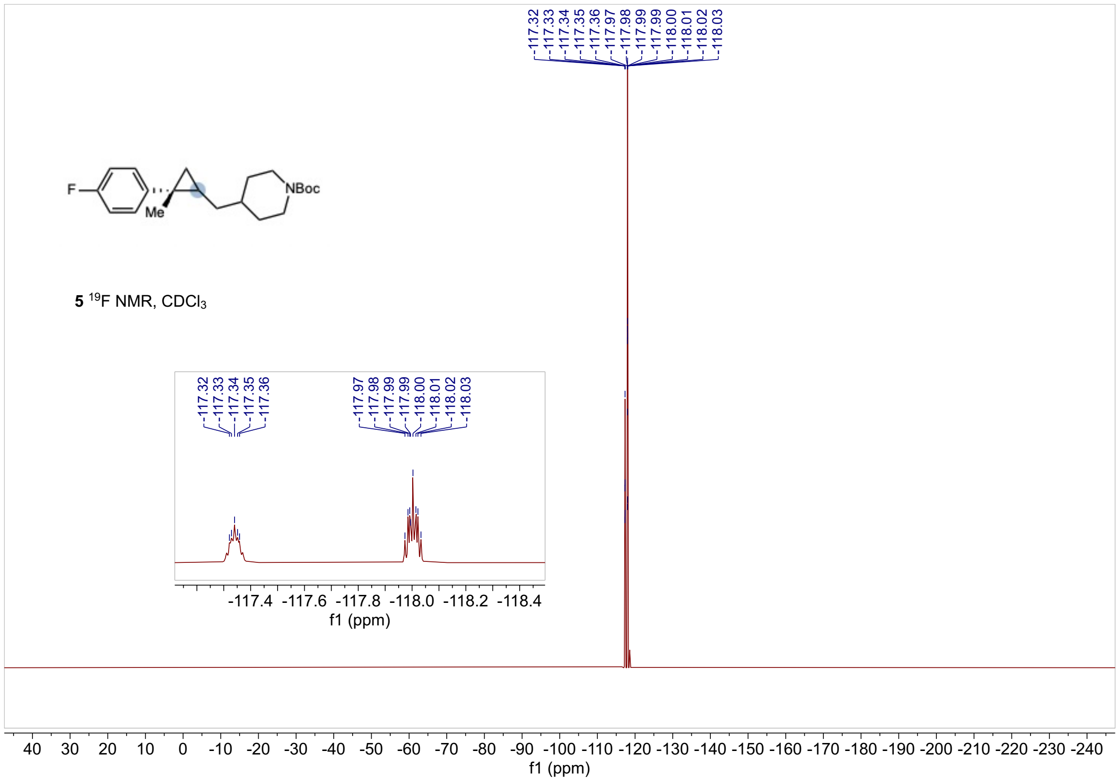

-117.32
-117.33
-117.34
-117.35
-117.36
-117.97
-117.98
-117.99
-117.99
-118.00
-118.01
-118.02
-118.03
F
Me
NBoc
5 ^{19}F NMR, CDCl$_3$
-117.4 -117.6 -117.8 -118.0 -118.2 -118.4
f1 (ppm)
40 30 20 10 0 -10 -20 -30 -40 -50 -60 -70 -80 -90 -100 -110 -120 -130 -140 -150 -160 -170 -180 -190 -200 -210 -220 -230 -240
f1 (ppm)

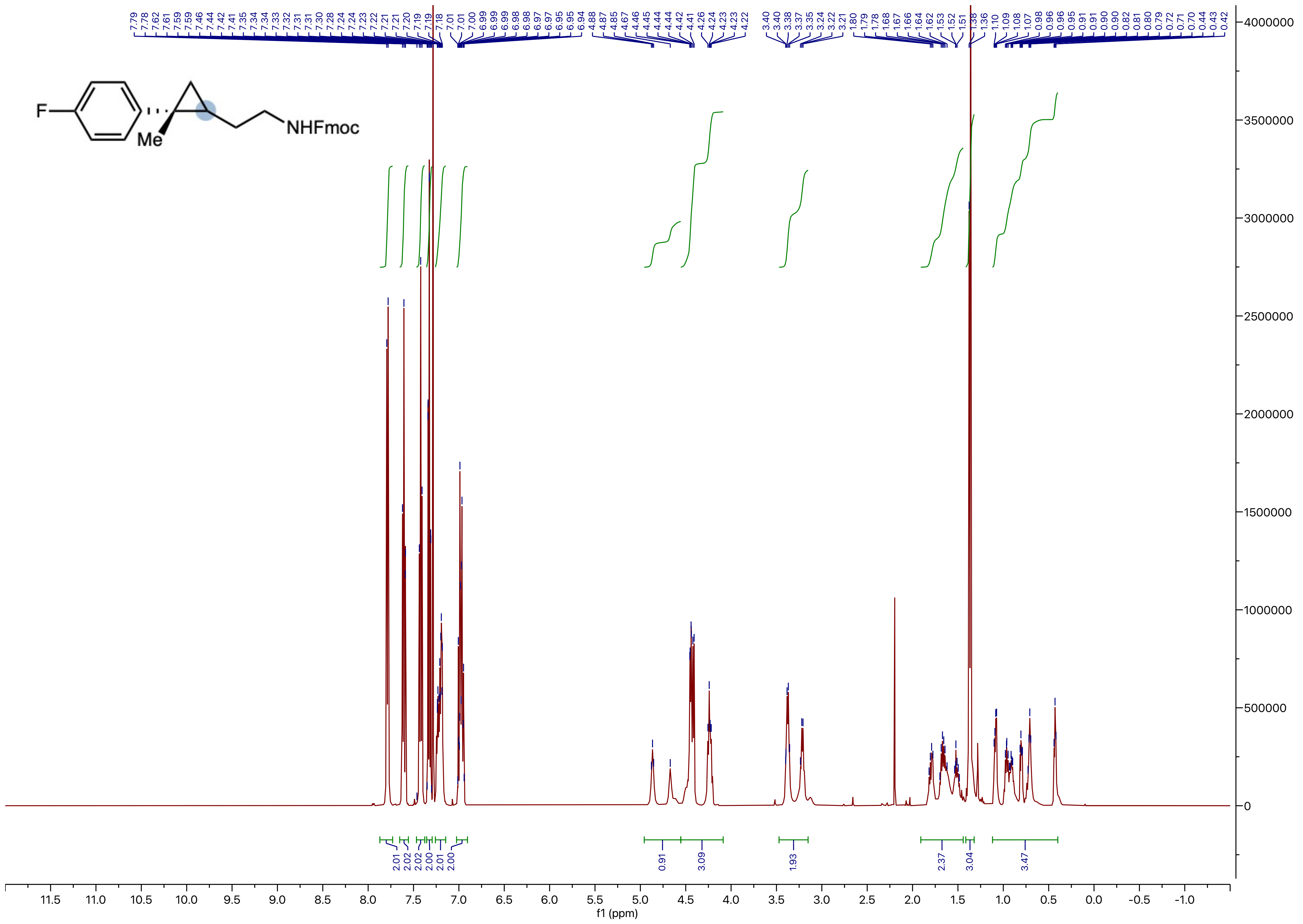

F
Me
NHFmoc
f1 (ppm)

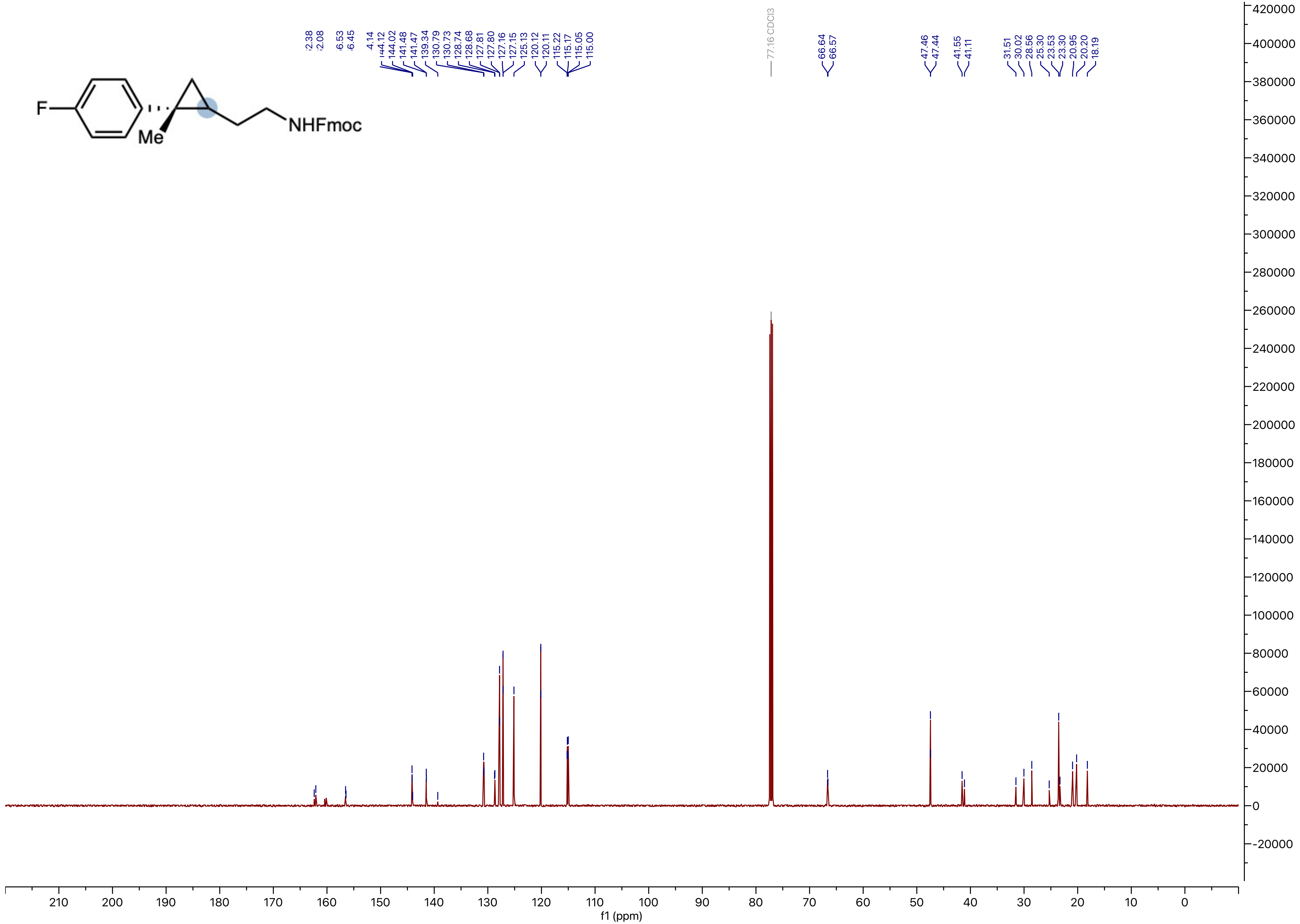
F
Me
NHFmoc
144.02
141.48
141.47
139.34
130.79
130.73
128.74
128.68
127.81
127.80
127.16
127.15
125.13
120.12
120.11
115.22
115.17
115.05
115.00
77.16 CDCl3
66.64
66.57
47.46
47.44
41.55
41.11
31.51
30.02
28.56
25.30
23.53
23.30
20.95
20.20
18.19
f1 (ppm)

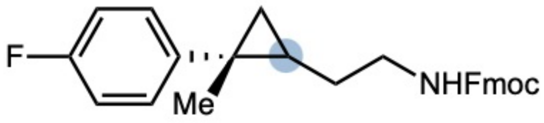
F
Me
NHFmoc

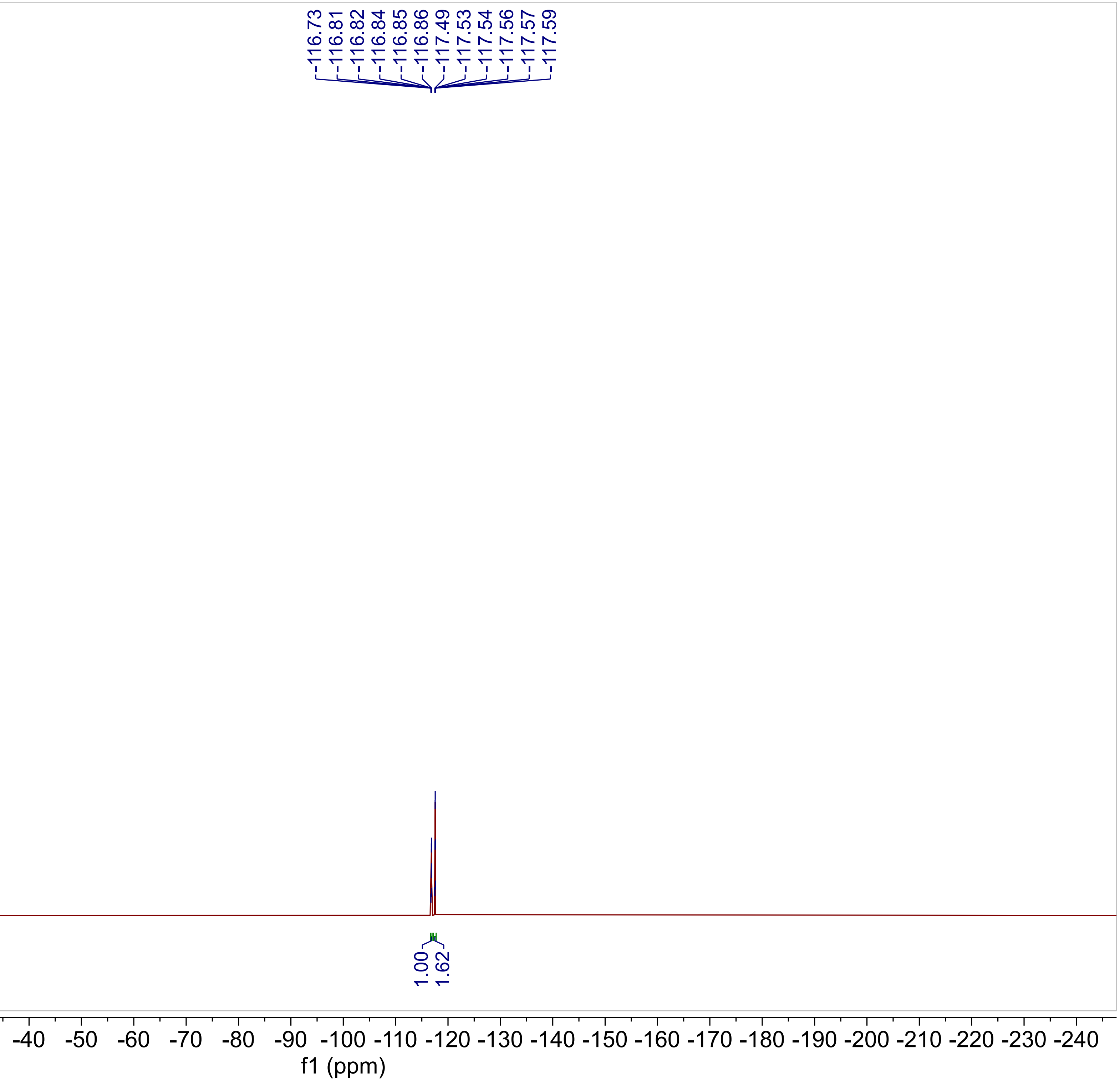
-116.73
-116.81
-116.82
-116.84
-116.85
-116.86
-117.49
-117.53
-117.54
-117.56
-117.57
-117.59
1.00
1.62
40 30 20 10 0 -10 -20 -30 -40 -50 -60 -70 -80 -90 -100 -110 -120 -130 -140 -150 -160 -170 -180 -190 -200 -210 -220 -230 -240
f1 (ppm)

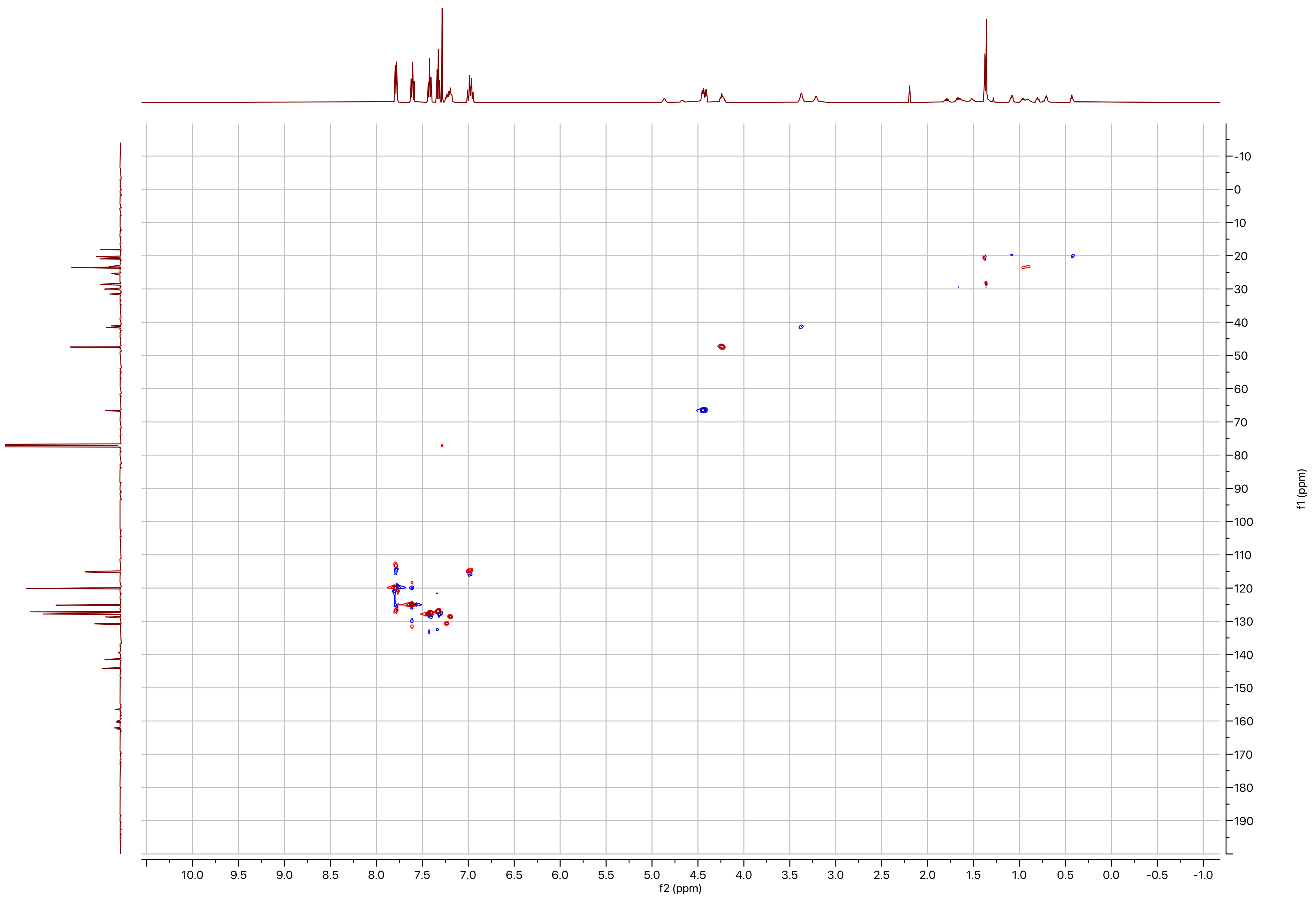

f2 (ppm)
f1 (ppm)

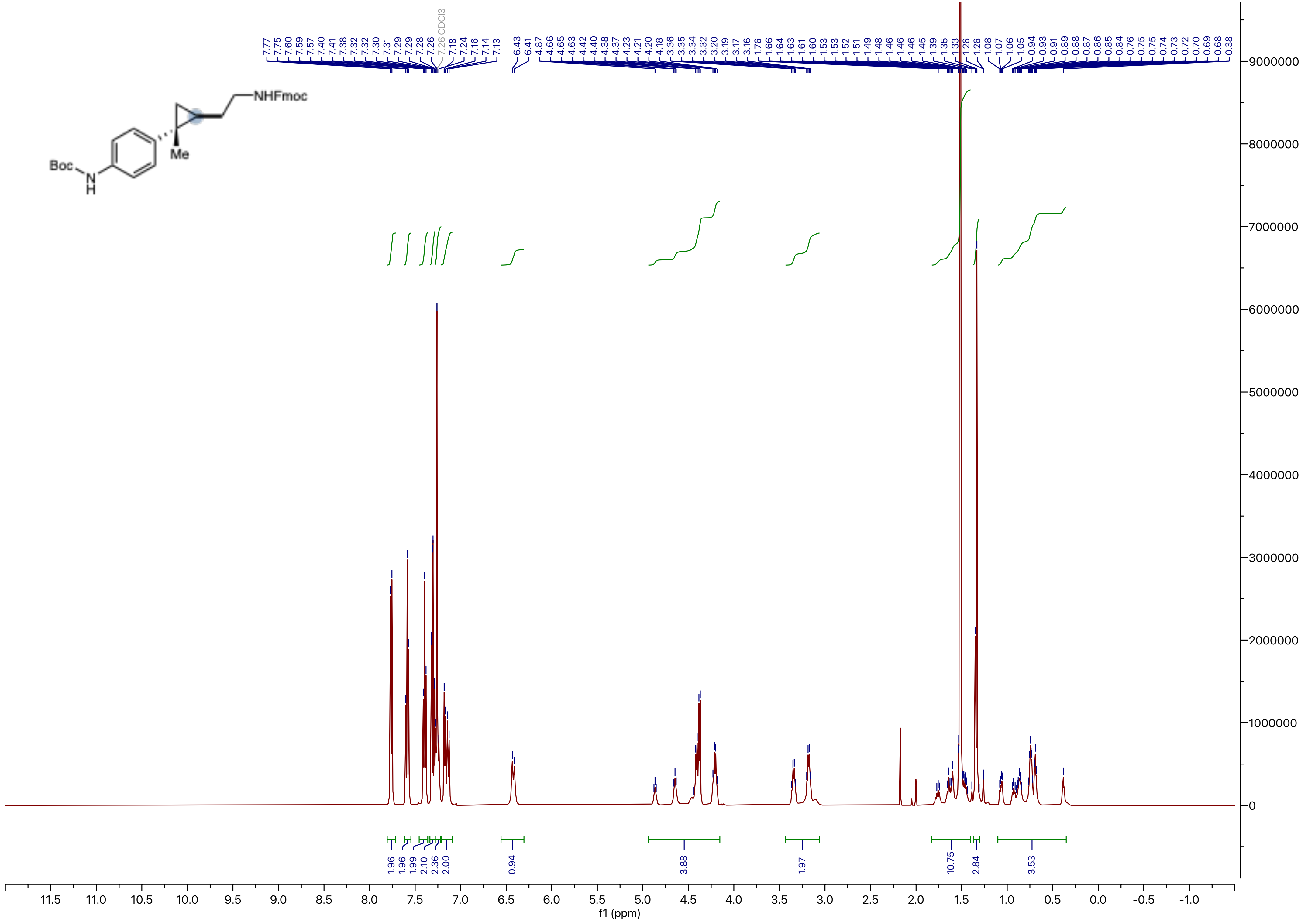

NHFmoc
Boc
N
H
Me
7.77
7.75
7.60
7.59
7.57
7.40
7.41
7.38
7.32
7.32
7.30
7.31
7.29
7.29
7.28
7.26
7.26 CDCl3
7.18
7.24
7.16
7.14
7.13
6.43
6.41
4.87
4.66
4.65
4.63
4.42
4.40
4.38
4.37
4.23
4.21
4.20
4.18
3.36
3.35
3.34
3.32
3.20
3.19
3.17
3.16
1.76
1.66
1.64
1.63
1.61
1.60
1.53
1.53
1.52
1.51
1.49
1.48
1.46
1.46
1.46
1.45
1.39
1.35
1.33
1.26
1.26
1.08
1.07
1.06
1.05
0.94
0.93
0.91
0.89
0.88
0.87
0.86
0.85
0.84
0.76
0.75
0.75
0.74
0.73
0.72
0.70
0.69
0.68
0.38
1.96
1.96
1.99
2.10
2.36
2.00
0.94
3.88
1.97
10.75
2.84
3.53
9000000
8000000
7000000
6000000
5000000
4000000
3000000
2000000
1000000
0
11.5
11.0
10.5
10.0
9.5
9.0
8.5
8.0
7.5
7.0
6.5
6.0
5.5
5.0
4.5
4.0
3.5
3.0
2.5
2.0
1.5
1.0
0.5
0.0
-0.5
-1.0
f1 (ppm)

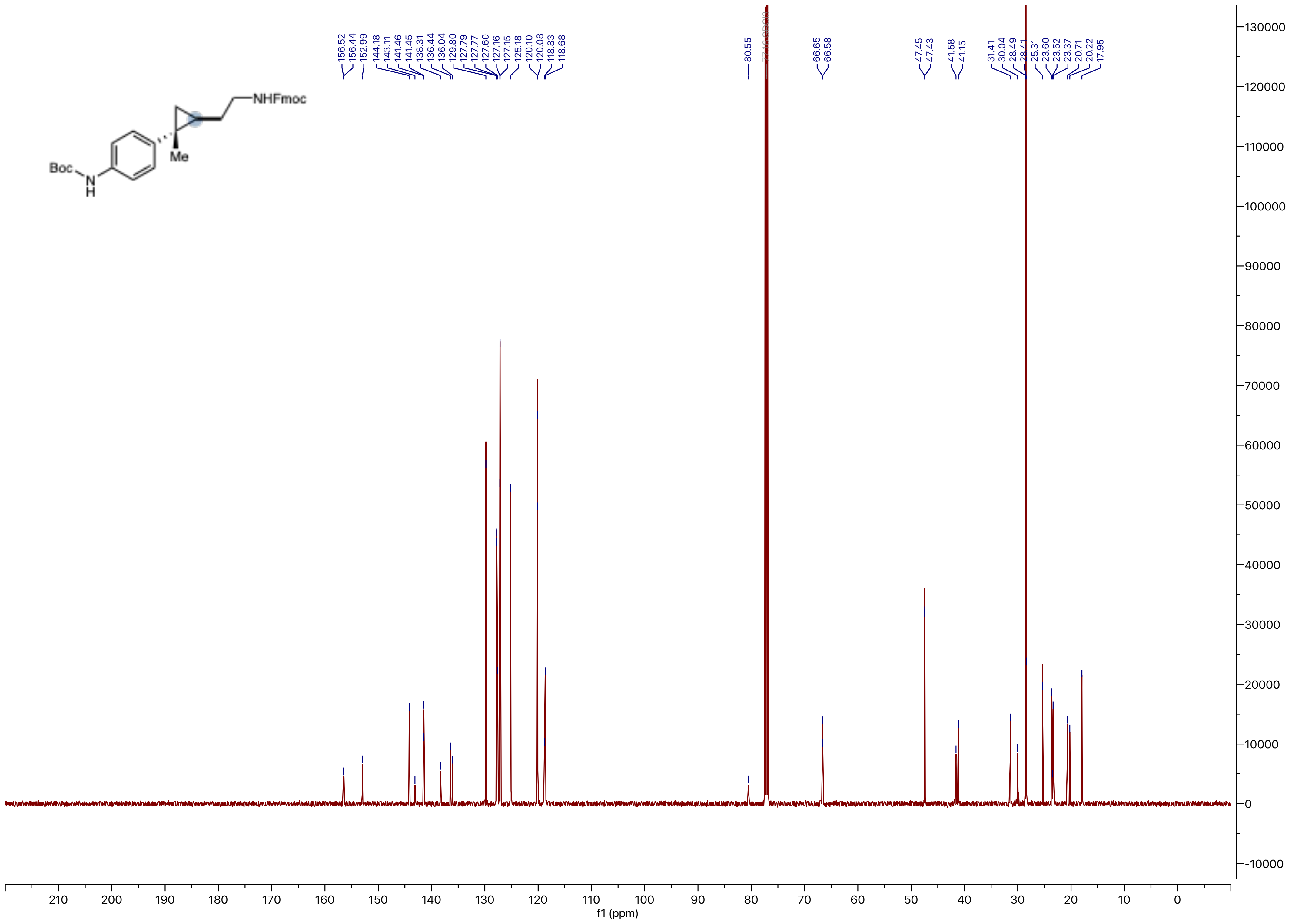

156.52
156.44
152.99
144.18
143.11
141.46
141.45
138.31
136.44
136.04
129.80
127.79
127.77
127.60
127.16
127.15
125.18
120.10
120.08
118.83
118.68
80.55
66.65
66.58
47.45
47.43
41.58
41.15
31.41
30.04
28.49
28.41
25.31
23.60
23.52
23.37
20.71
20.22
17.95
NHFmoc
Me
Boc
N
H
f1 (ppm)

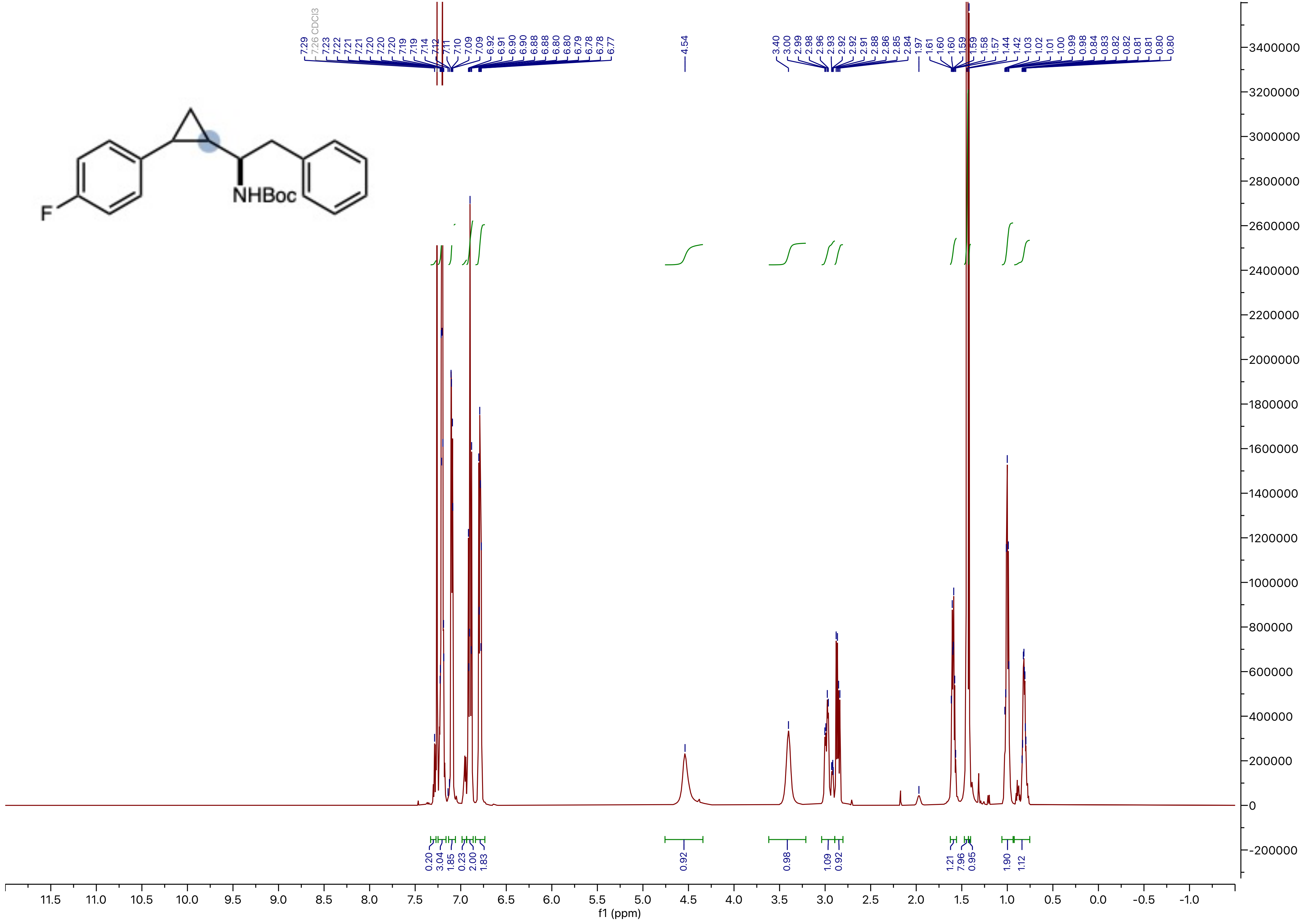

NHBoc
F
7.26 CDCl3
4.54
f1 (ppm)

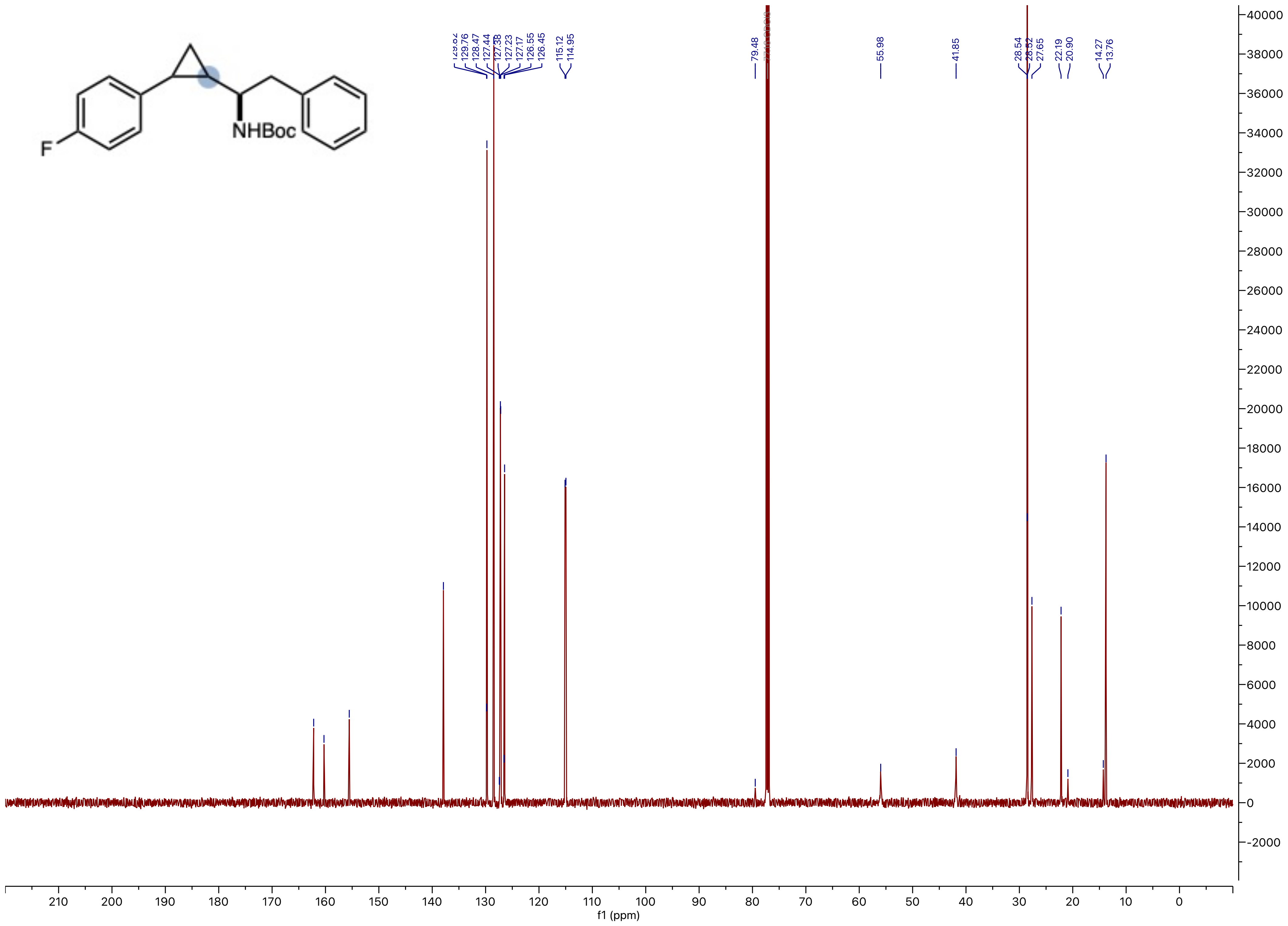

F
NHBoc
129.76
128.47
127.44
127.38
127.23
127.17
126.55
126.45
115.12
114.95
79.48
55.98
41.85
28.54
28.52
27.65
22.19
20.90
14.27
13.76
f1 (ppm)

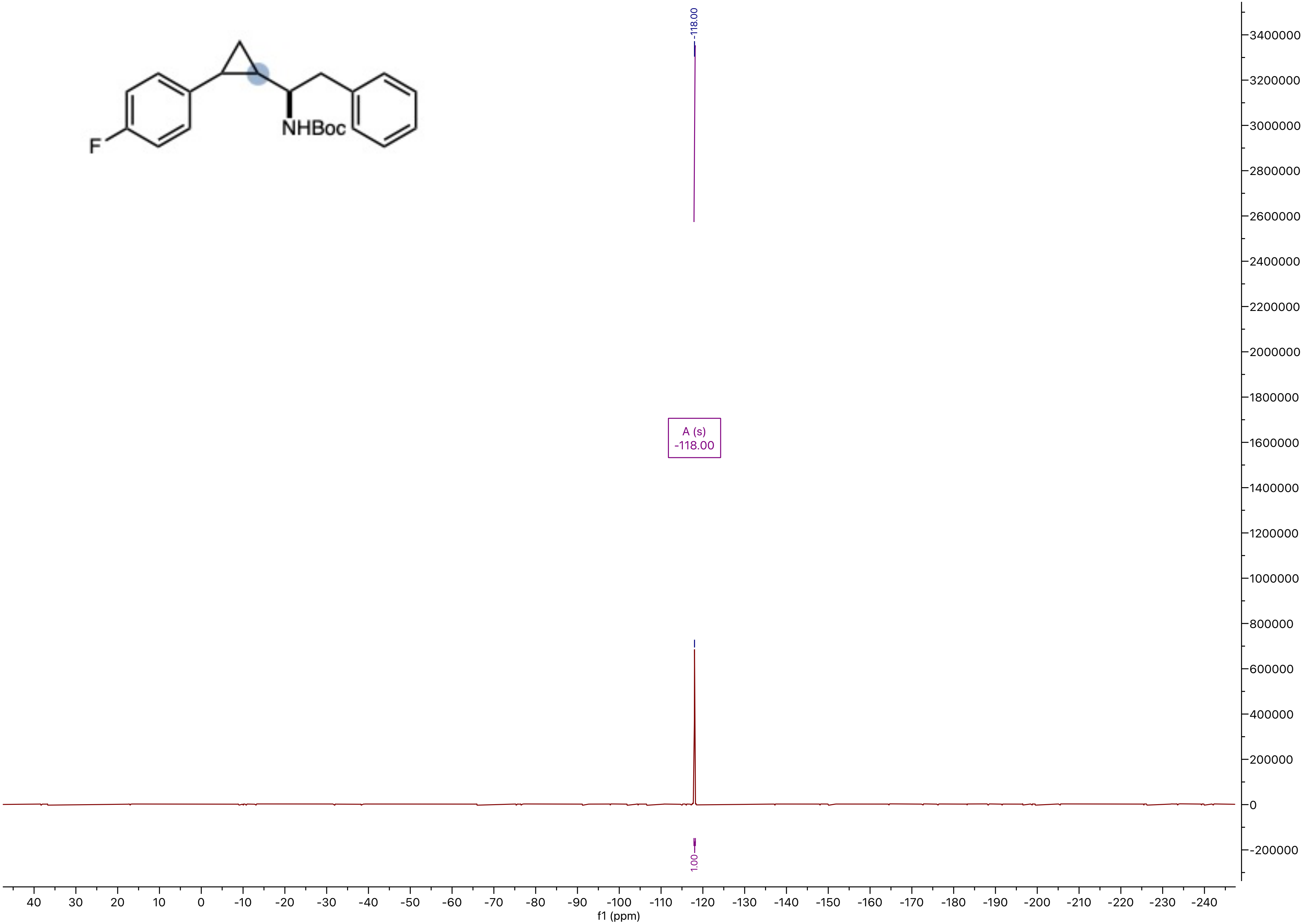
F
NHBoc
-118.00
A (s)
-118.00
1.00
f1 (ppm)

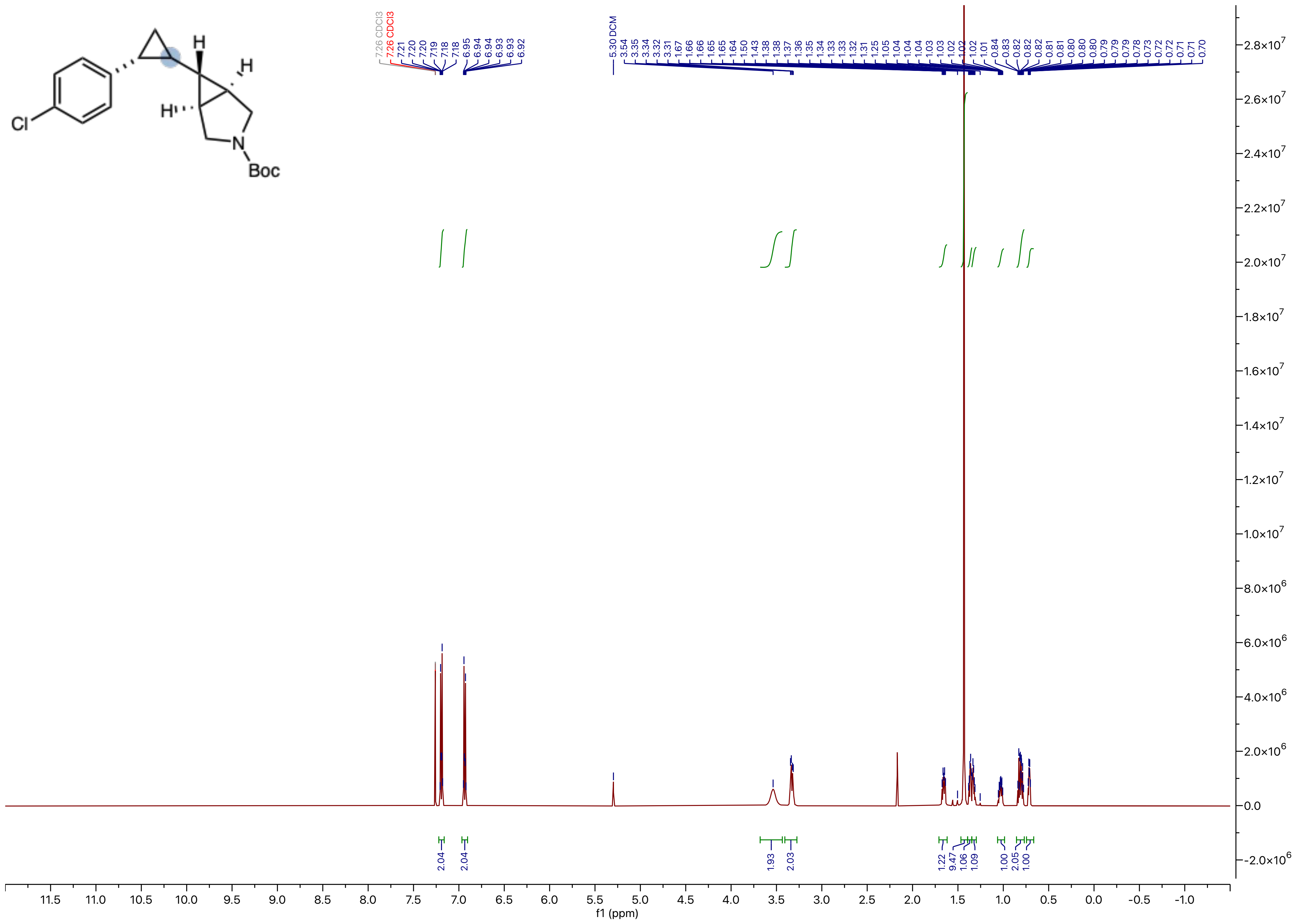
Cl
H
H
H
N
Boc
7.26 CDCl3
5.30 DCM
2.04
2.04
1.93
2.03
1.22
9.47
1.06
1.09
1.00
2.05
1.00
f1 (ppm)

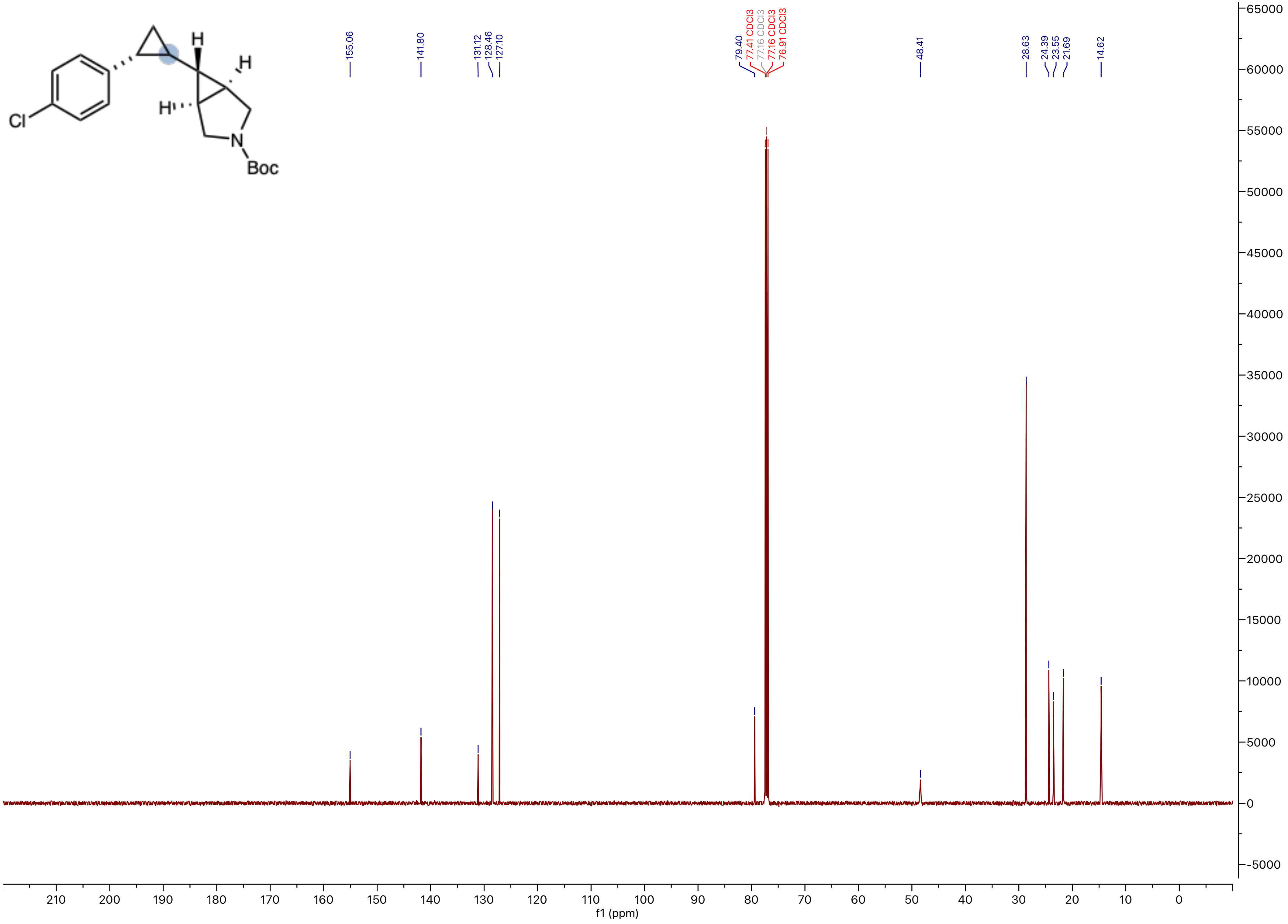

Cl
H
H
H
N
Boc
155.06
141.80
131.12
128.46
127.10
79.40
77.41 CDCl3
77.16 CDCl3
77.16 CDCl3
76.91 CDCl3
48.41
28.63
24.39
23.55
21.69
14.62
f1 (ppm)

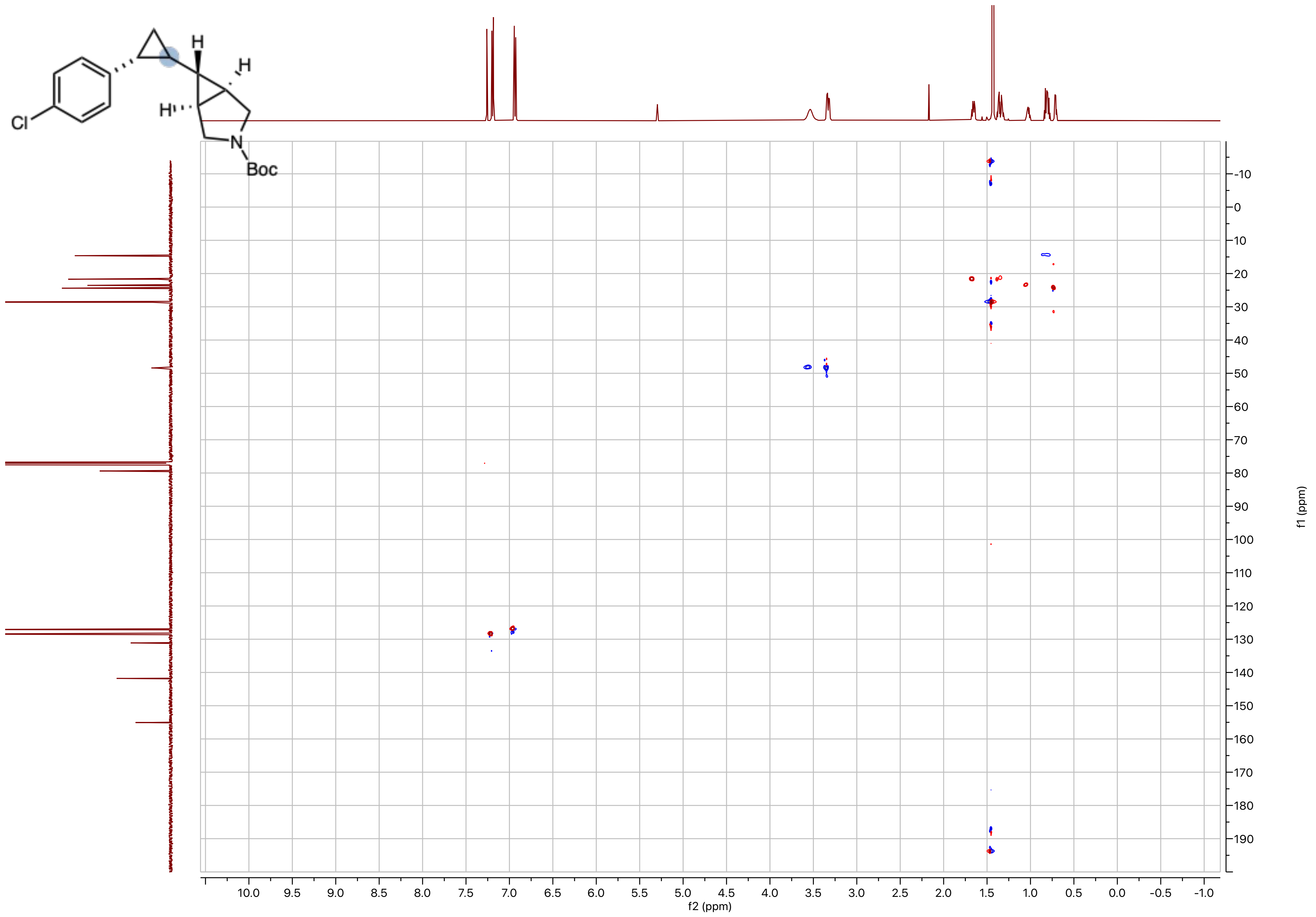

Cl
H
H
H
N
Boc
f2 (ppm)
f1 (ppm)

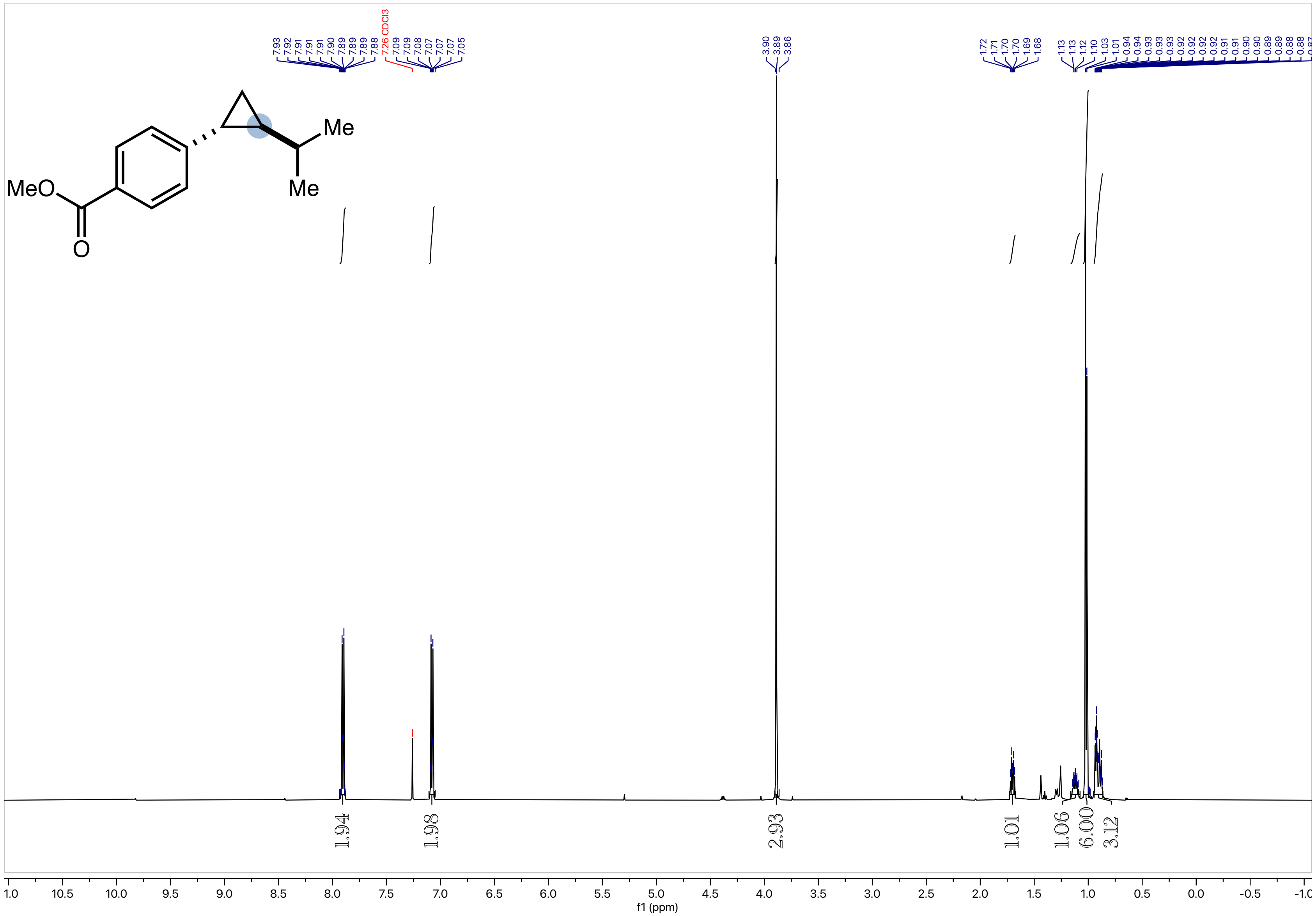
MeO
O
Me
Me
7.93
7.92
7.91
7.91
7.91
7.90
7.89
7.89
7.89
7.88
7.26 CDCl3
7.09
7.09
7.08
7.07
7.07
7.07
7.05
3.90
3.89
3.86
1.72
1.71
1.70
1.70
1.69
1.68
1.13
1.13
1.12
1.10
1.03
1.01
0.94
0.94
0.93
0.93
0.93
0.93
0.92
0.92
0.92
0.92
0.91
0.91
0.90
0.90
0.89
0.89
0.88
0.88
1.94
1.98
2.93
1.01
1.06
6.00
3.12
f1 (ppm)

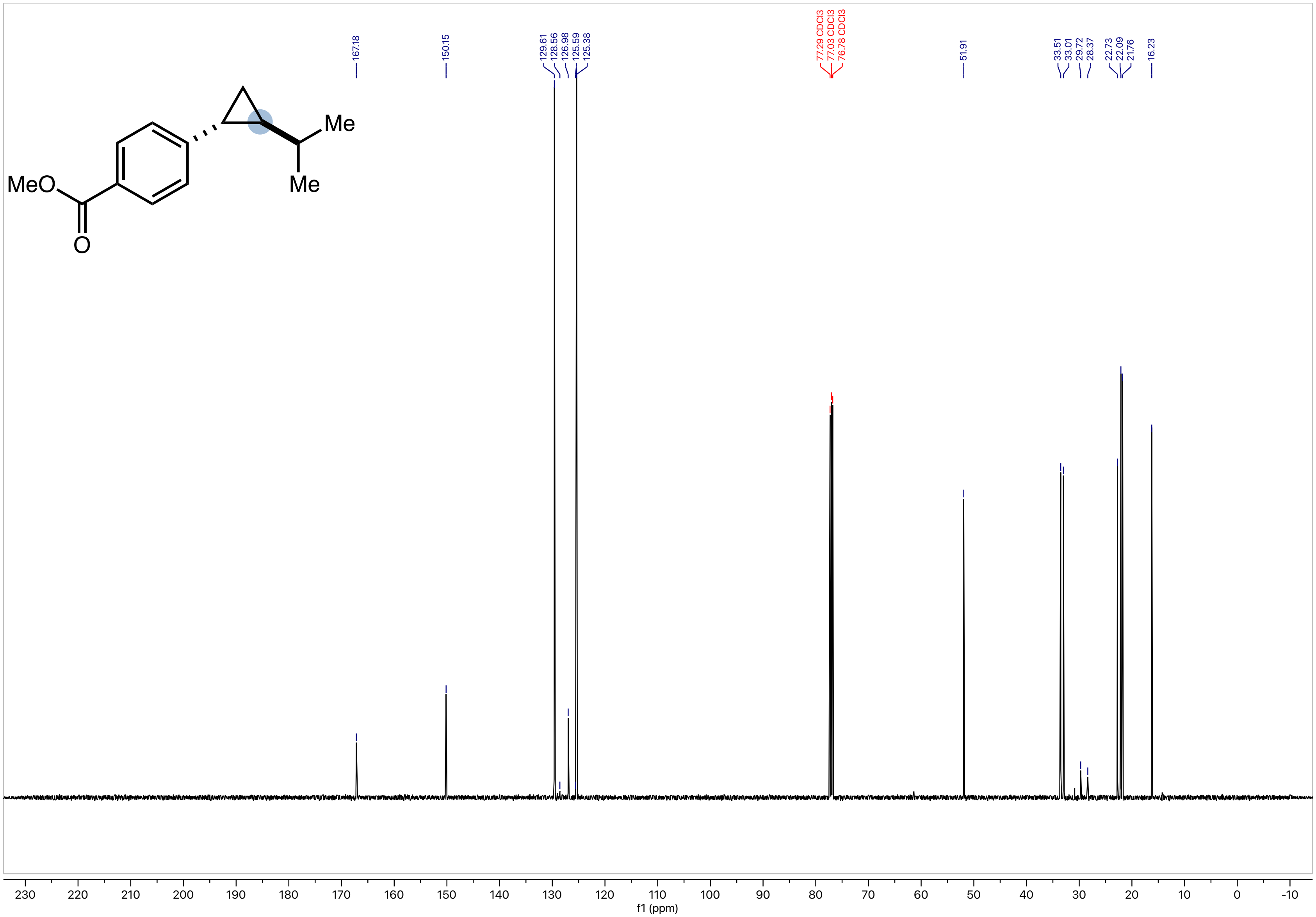

MeO
O
Me
Me
167.18
150.15
129.61
128.56
126.98
125.59
125.38
77.29 CDCl3
77.03 CDCl3
76.78 CDCl3
51.91
33.51
33.01
29.72
28.37
22.73
22.09
21.76
16.23
230 220 210 200 190 180 170 160 150 140 130 120 110 100 90 80 70 60 50 40 30 20 10 0 -10
f1 (ppm)

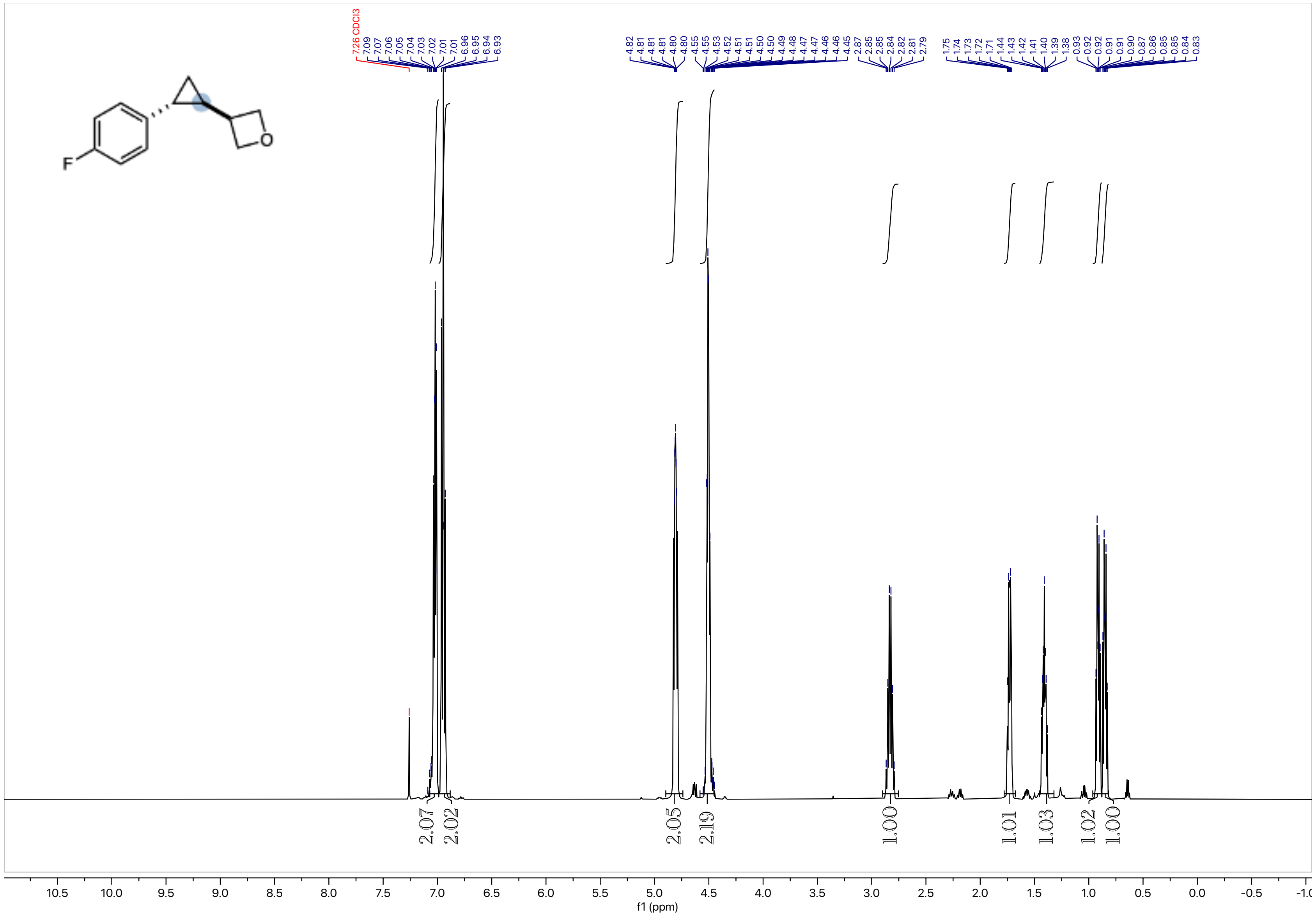

7.26 CDCl3
7.09
7.07
7.06
7.05
7.04
7.03
7.02
7.01
7.01
6.96
6.95
6.94
6.93
4.82
4.81
4.81
4.81
4.80
4.80
4.55
4.55
4.53
4.52
4.51
4.51
4.50
4.50
4.49
4.48
4.47
4.46
4.46
4.45
2.87
2.85
2.85
2.84
2.82
2.81
2.79
1.75
1.74
1.73
1.72
1.71
1.44
1.43
1.42
1.41
1.40
1.39
1.38
0.93
0.92
0.92
0.91
0.91
0.90
0.87
0.86
0.85
0.85
0.84
0.83
F
O
2.07
2.02
2.05
2.19
1.00
1.01
1.03
1.02
1.00
10.5
10.0
9.5
9.0
8.5
8.0
7.5
7.0
6.5
6.0
5.5
5.0
4.5
4.0
3.5
3.0
2.5
2.0
1.5
1.0
0.5
0.0
-0.5
f1 (ppm)

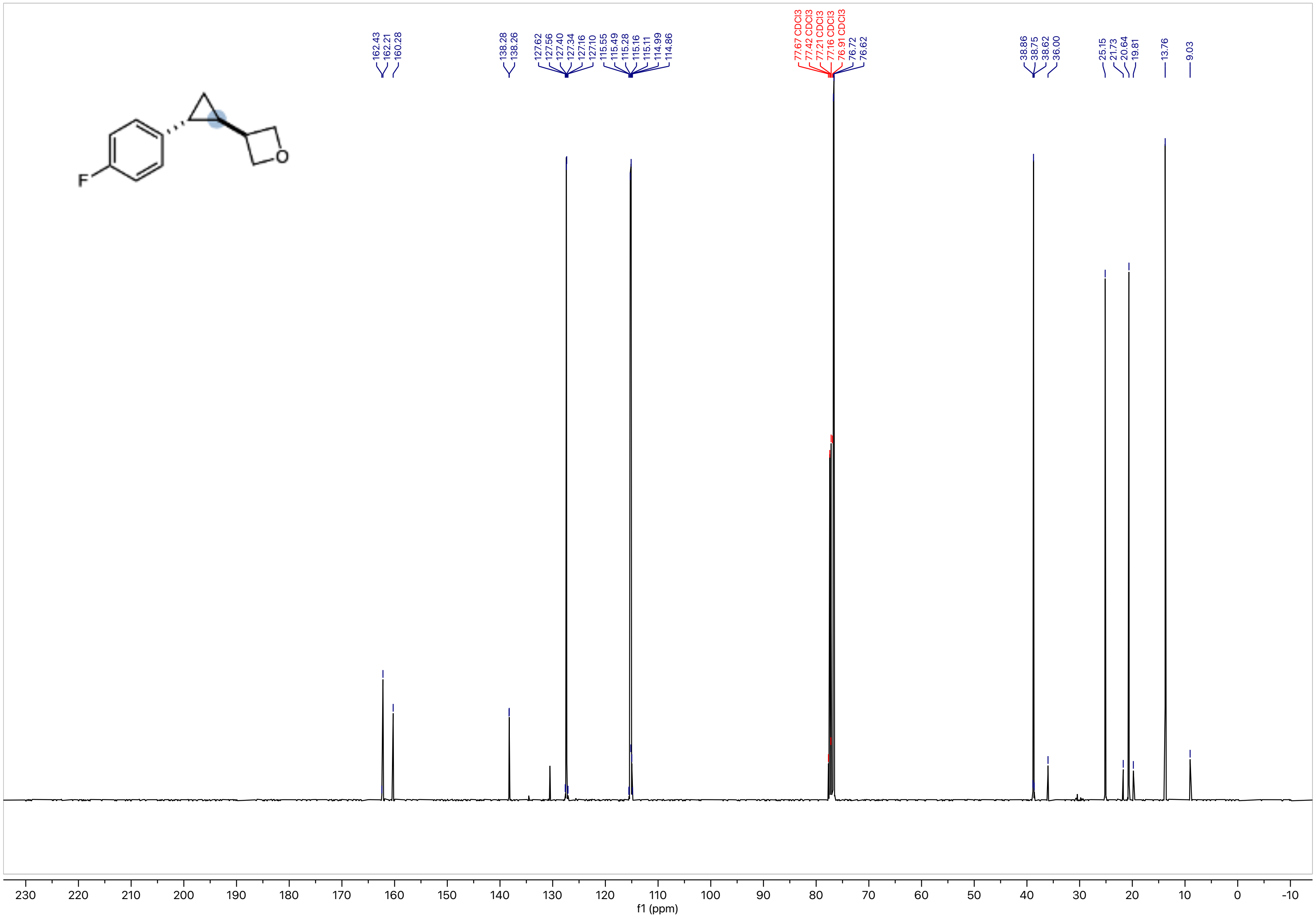
162.43
162.21
160.28
138.28
138.26
127.62
127.56
127.40
127.34
127.16
127.10
115.55
115.49
115.28
115.16
115.11
114.99
114.86
77.67 CDCl3
77.42 CDCl3
77.21 CDCl3
77.16 CDCl3
76.91 CDCl3
76.72
76.62
38.86
38.75
38.62
36.00
25.15
21.73
20.64
19.81
13.76
9.03
F
O
230 220 210 200 190 180 170 160 150 140 130 120 110 100 90 80 70 60 50 40 30 20 10 0 -10
f1 (ppm)

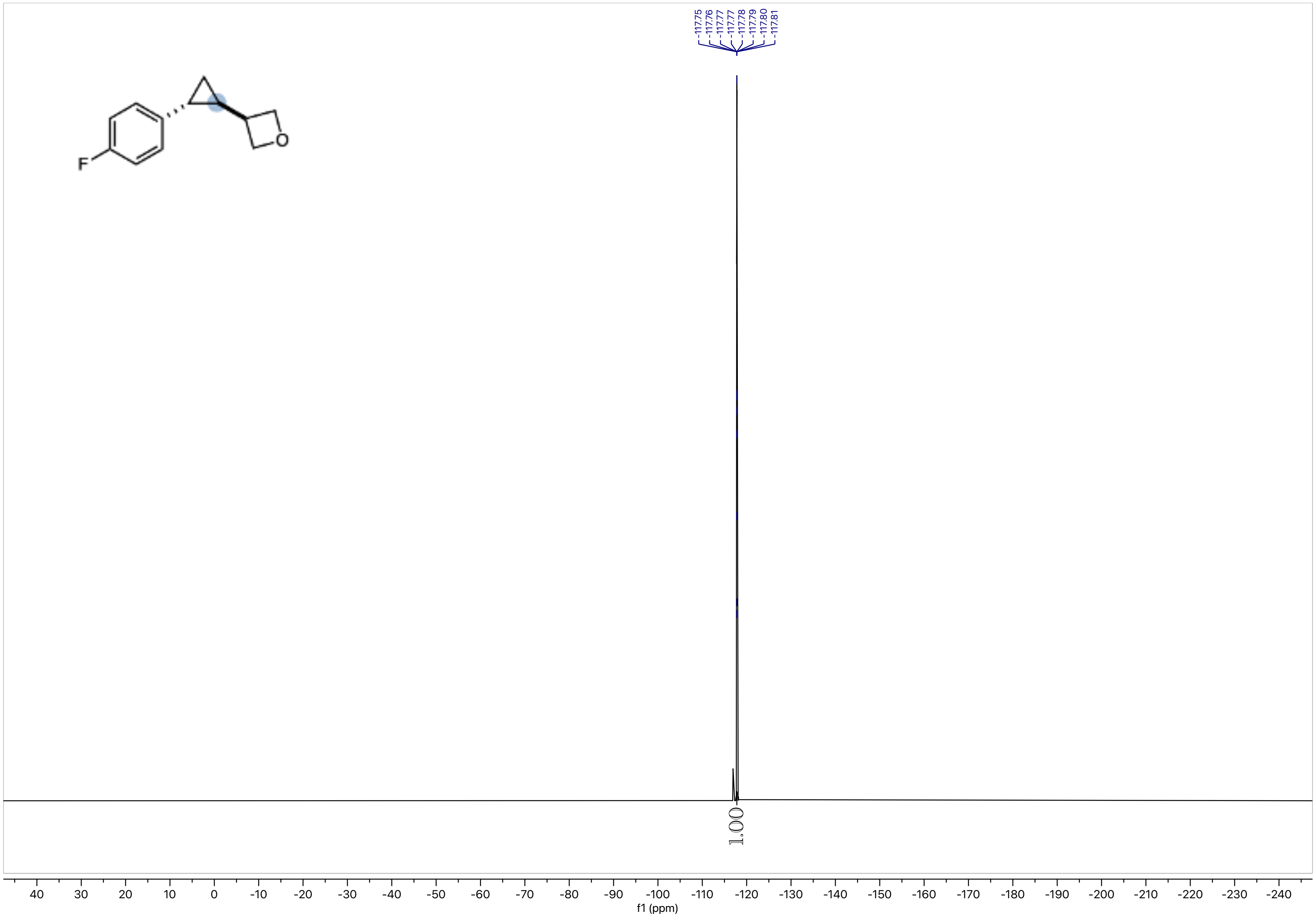

-117.75
-117.76
-117.77
-117.77
-117.78
-117.79
-117.80
-117.81
F
O
1.00
40 30 20 10 0 -10 -20 -30 -40 -50 -60 -70 -80 -90 -100 -110 -120 -130 -140 -150 -160 -170 -180 -190 -200 -210 -220 -230 -240
f1 (ppm)

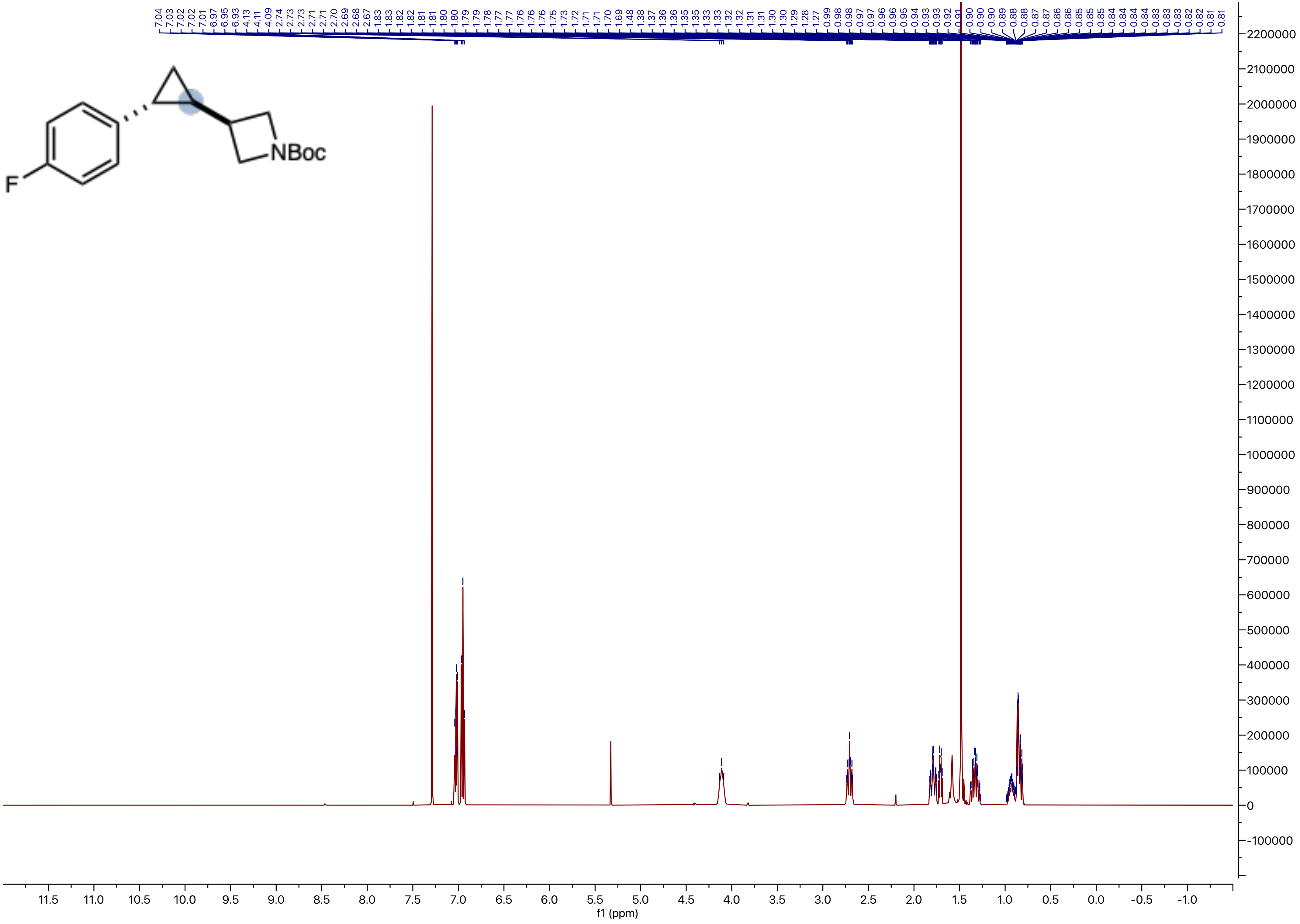
F
NBoc
f1 (ppm)

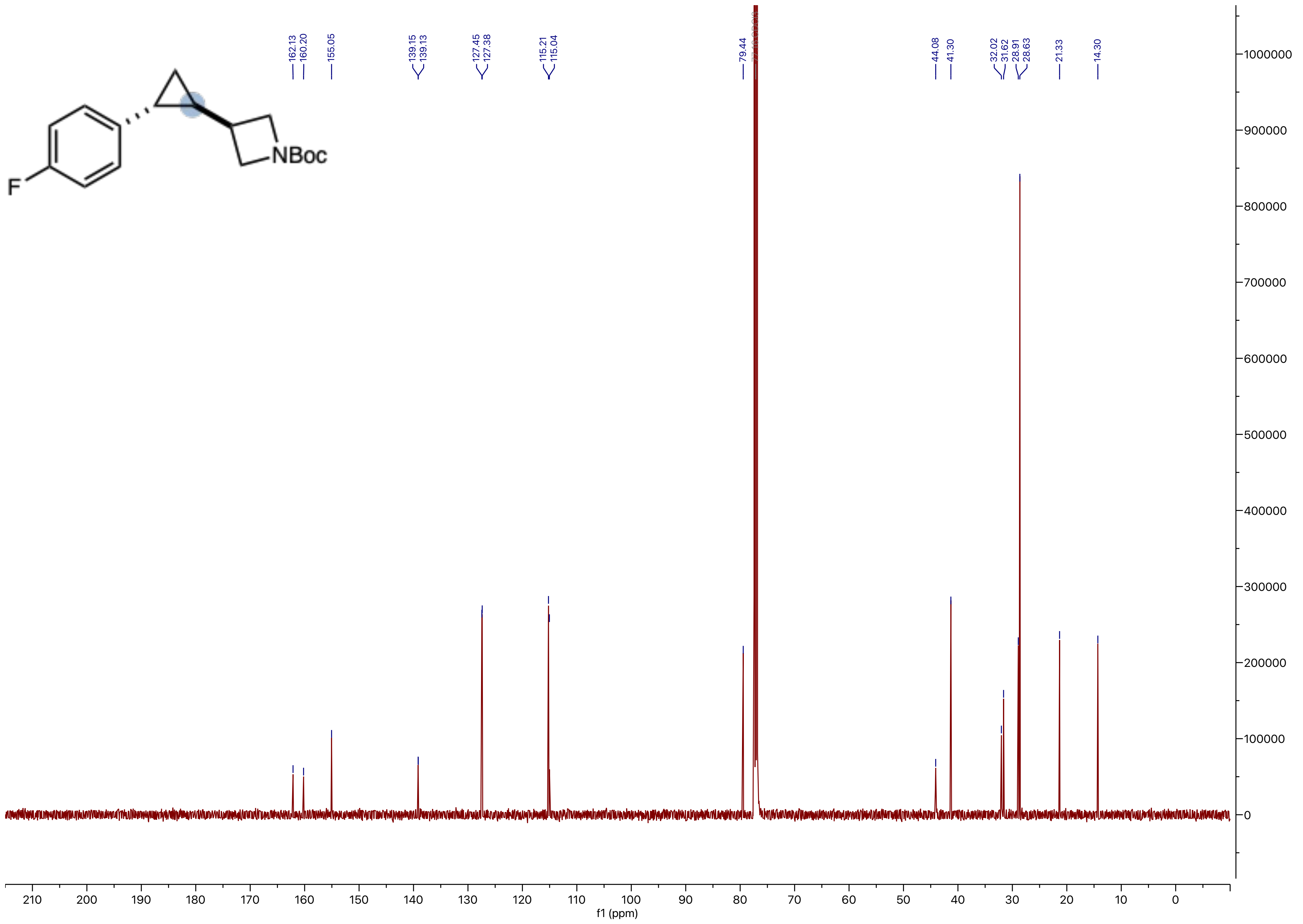

F
NBoc
162.13
160.20
155.05
139.15
139.13
127.45
127.38
115.21
115.04
79.44
44.08
41.30
32.02
31.62
28.91
28.63
21.33
14.30
1000000
900000
800000
700000
600000
500000
400000
300000
200000
100000
0
210
200
190
180
170
160
150
140
130
120
110
100
90
80
70
60
50
40
30
20
10
0
f1 (ppm)

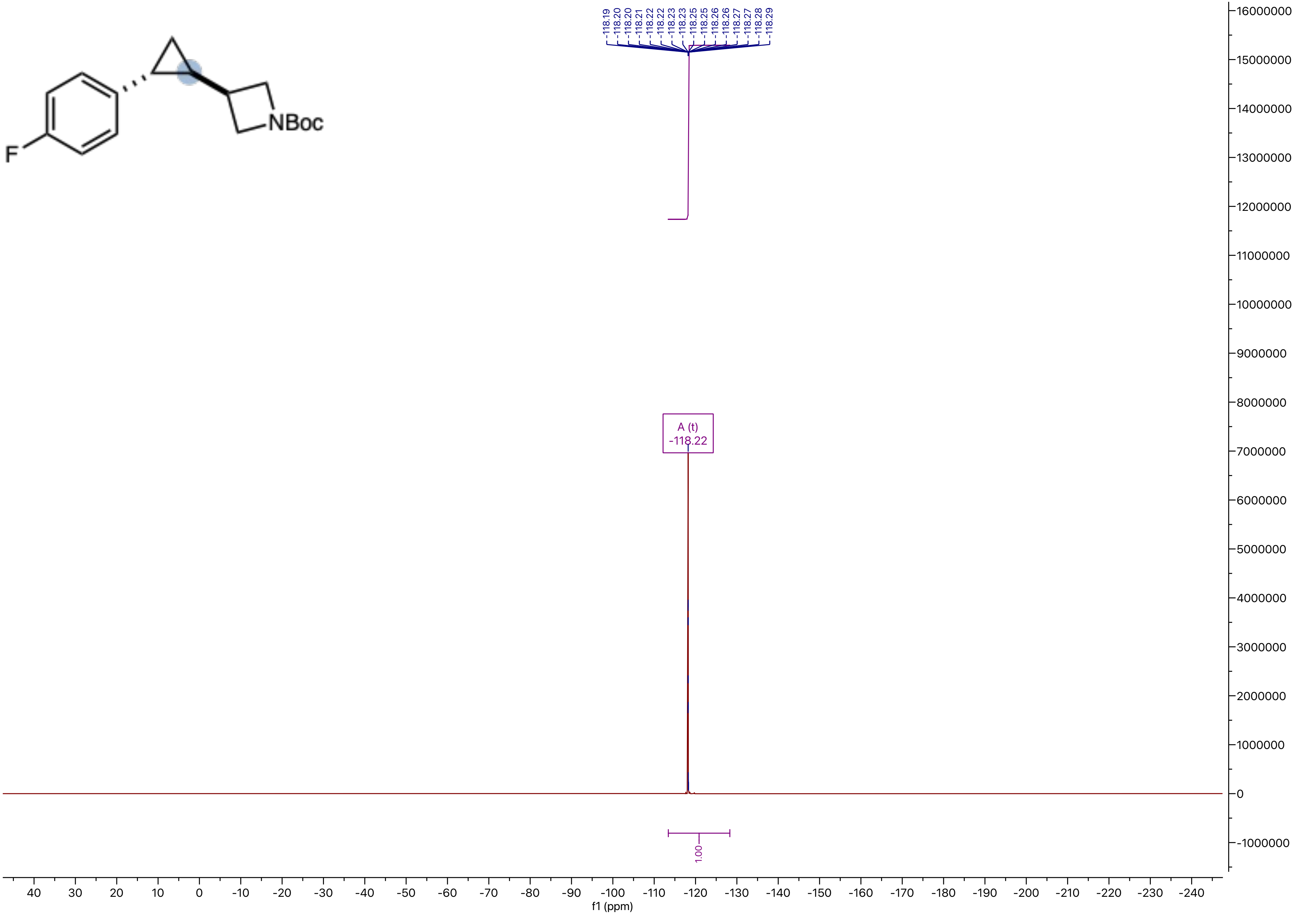

F
NBoc
-118.19
-118.20
-118.20
-118.21
-118.22
-118.22
-118.23
-118.23
-118.25
-118.25
-118.26
-118.26
-118.27
-118.27
-118.28
-118.29
A (t)
-118.22
1.00
f1 (ppm)

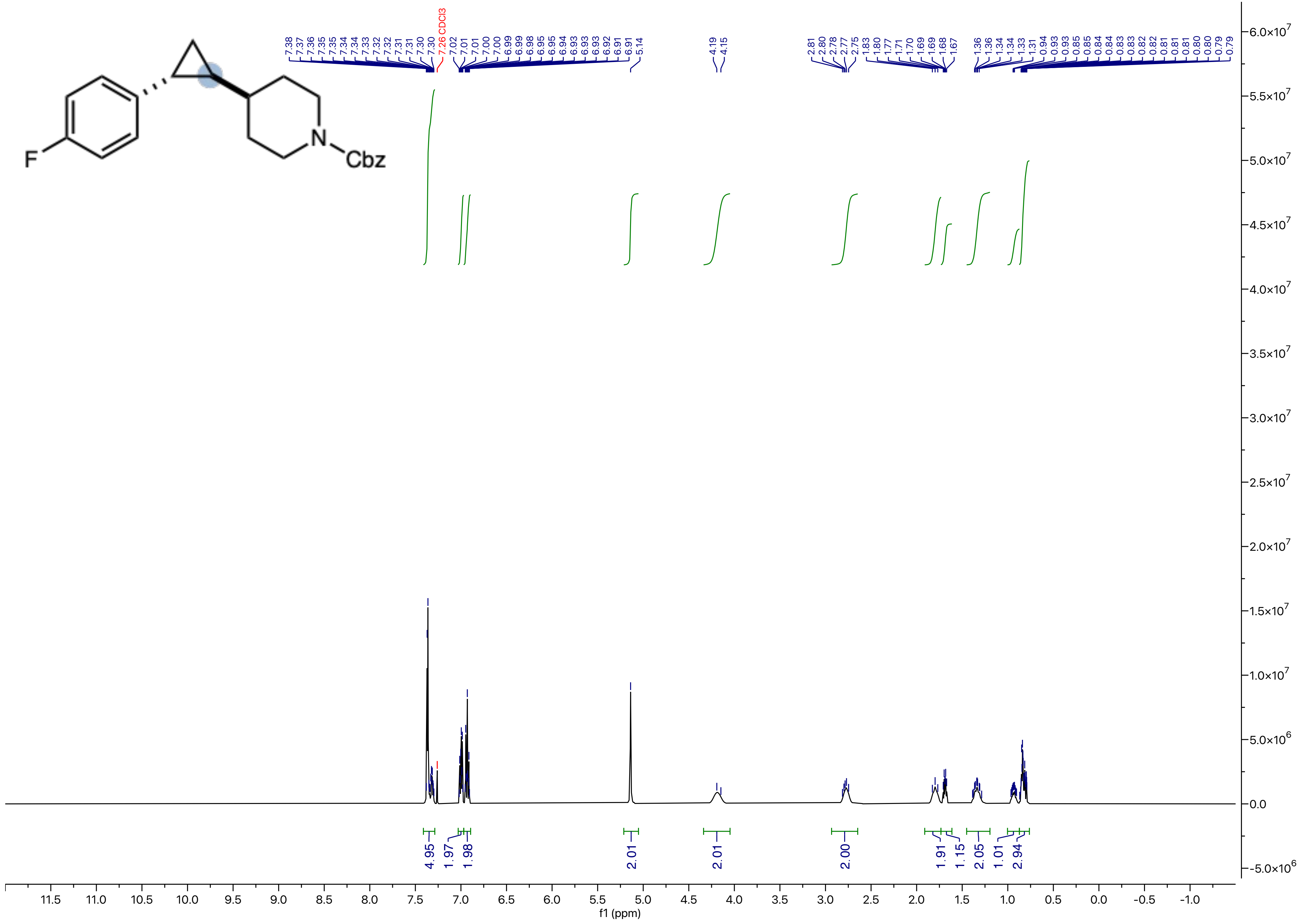
F
N
Cbz
7.38 7.37 7.36 7.35 7.35 7.34 7.34 7.33 7.32 7.32 7.31 7.31 7.30 7.30
7.26 CDCl3
7.02 7.01 7.01 7.00 7.00 6.99 6.99 6.98 6.95 6.95 6.94 6.93 6.93 6.93 6.92 6.91 6.91
5.14
4.19 4.15
2.81 2.80 2.78 2.77 2.75
1.83 1.80 1.77 1.71 1.70 1.69 1.69 1.68 1.67
1.36 1.36 1.34 1.34 1.33 1.31
0.94 0.93 0.93 0.85 0.85 0.84 0.84 0.83 0.83 0.82 0.82 0.81 0.81 0.81 0.80 0.80 0.79 0.79
4.95 1.97 1.98 2.01 2.01 2.00 1.91 1.15 2.05 1.01 2.94
11.5 11.0 10.5 10.0 9.5 9.0 8.5 8.0 7.5 7.0 6.5 6.0 5.5 5.0 4.5 4.0 3.5 3.0 2.5 2.0 1.5 1.0 0.5 0.0 -0.5 -1.0
f1 (ppm)
6.0×10^7 5.5×10^7 5.0×10^7 4.5×10^7 4.0×10^7 3.5×10^7 3.0×10^7 2.5×10^7 2.0×10^7 1.5×10^7 1.0×10^7 5.0×10^6 0.0 -5.0×10^6

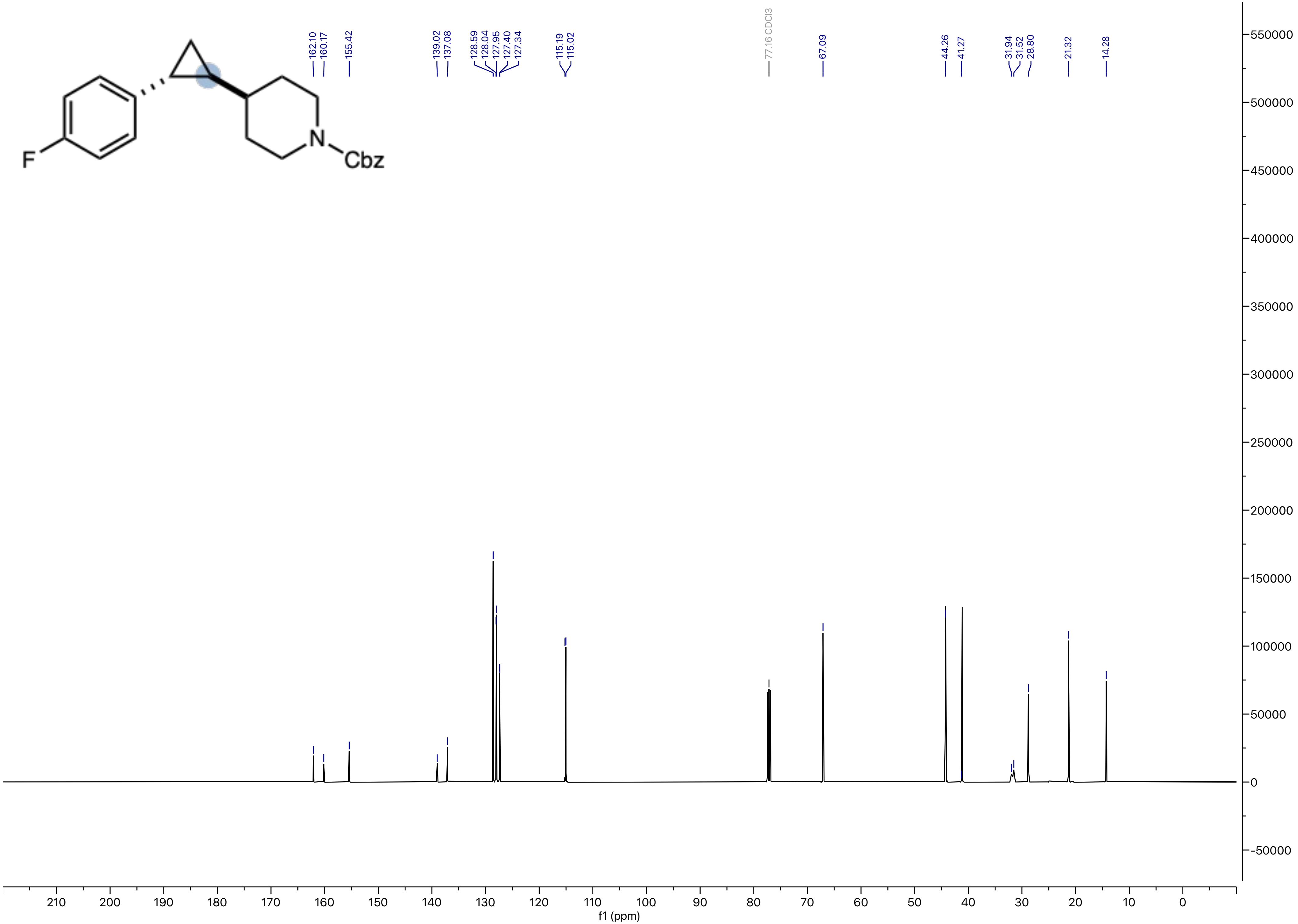
F
N
Cbz
162.10
160.17
155.42
139.02
137.08
128.59
128.04
127.95
127.40
127.34
115.19
115.02
77.16 CDCl3
67.09
44.26
41.27
31.94
31.52
28.80
21.32
14.28
550000
500000
450000
400000
350000
300000
250000
200000
150000
100000
50000
0
-50000
210
200
190
180
170
160
150
140
130
120
110
100
90
80
70
60
50
40
30
20
10
0
f1 (ppm)

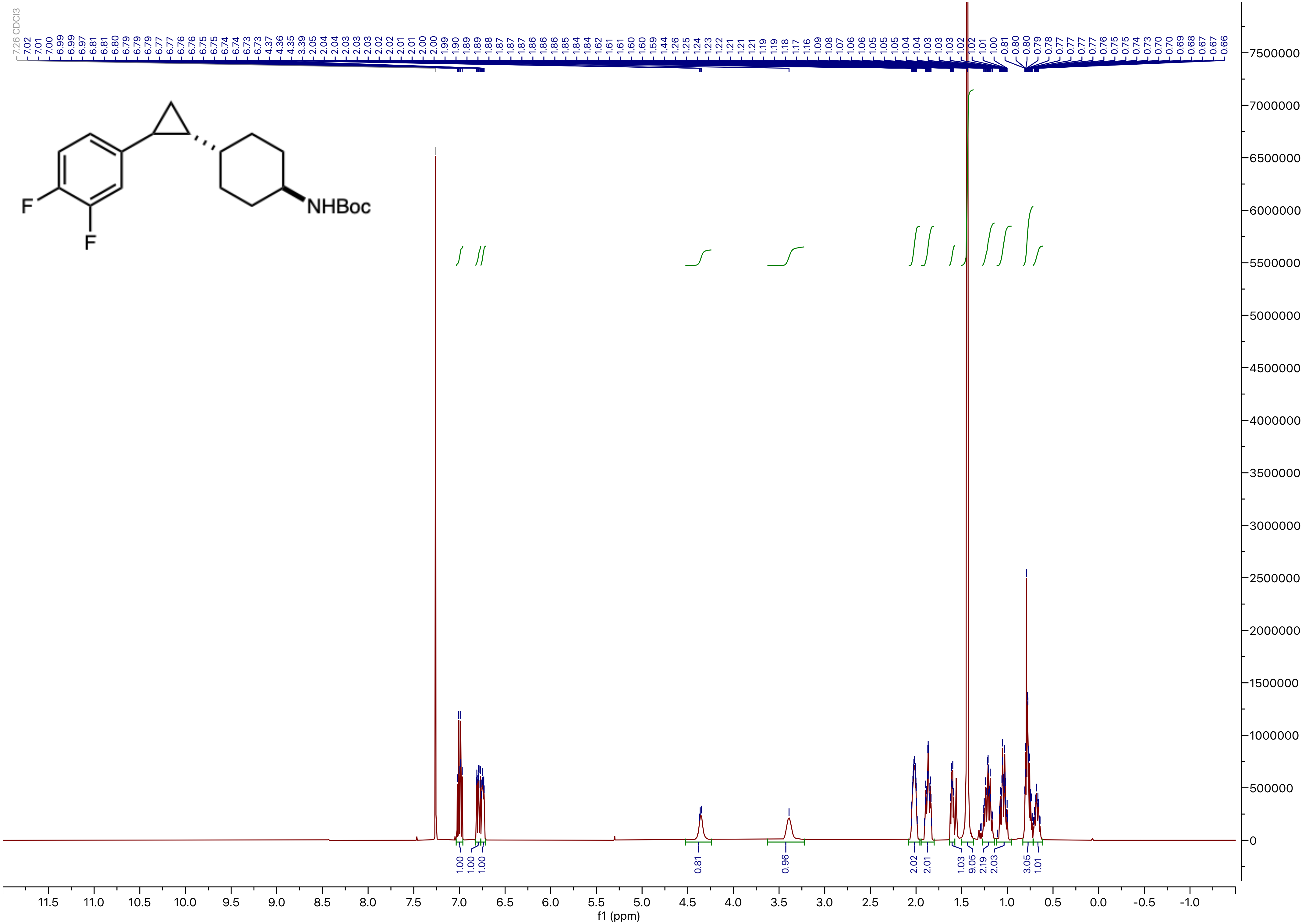
7.26 CDCl3
7.02 7.01 7.00 6.99 6.99 6.97 6.81 6.81 6.80 6.79 6.79 6.79 6.77 6.77 6.76 6.76 6.75 6.75 6.74 6.74 6.73 6.73 4.37 4.36 4.35 3.39 2.05 2.04 2.04 2.03 2.03 2.03 2.02 2.02 2.01 2.01 2.00 2.00 1.99 1.90 1.89 1.89 1.88 1.87 1.87 1.87 1.86 1.86 1.85 1.84 1.84 1.62 1.61 1.61 1.60 1.60 1.59 1.44 1.26 1.25 1.24 1.23 1.22 1.21 1.21 1.19 1.19 1.18 1.17 1.16 1.09 1.08 1.07 1.06 1.06 1.05 1.05 1.05 1.04 1.04 1.03 1.03 1.03 1.02 1.02 1.01 1.00 0.81 0.80 0.80 0.79 0.78 0.77 0.77 0.77 0.77 0.76 0.75 0.74 0.73 0.70 0.70 0.69 0.68 0.67 0.66
F
F
NHBoc
1.00 1.00 1.00 0.81 0.96 2.02 2.01 1.03 9.05 2.19 2.03 3.05 1.01
f1 (ppm)

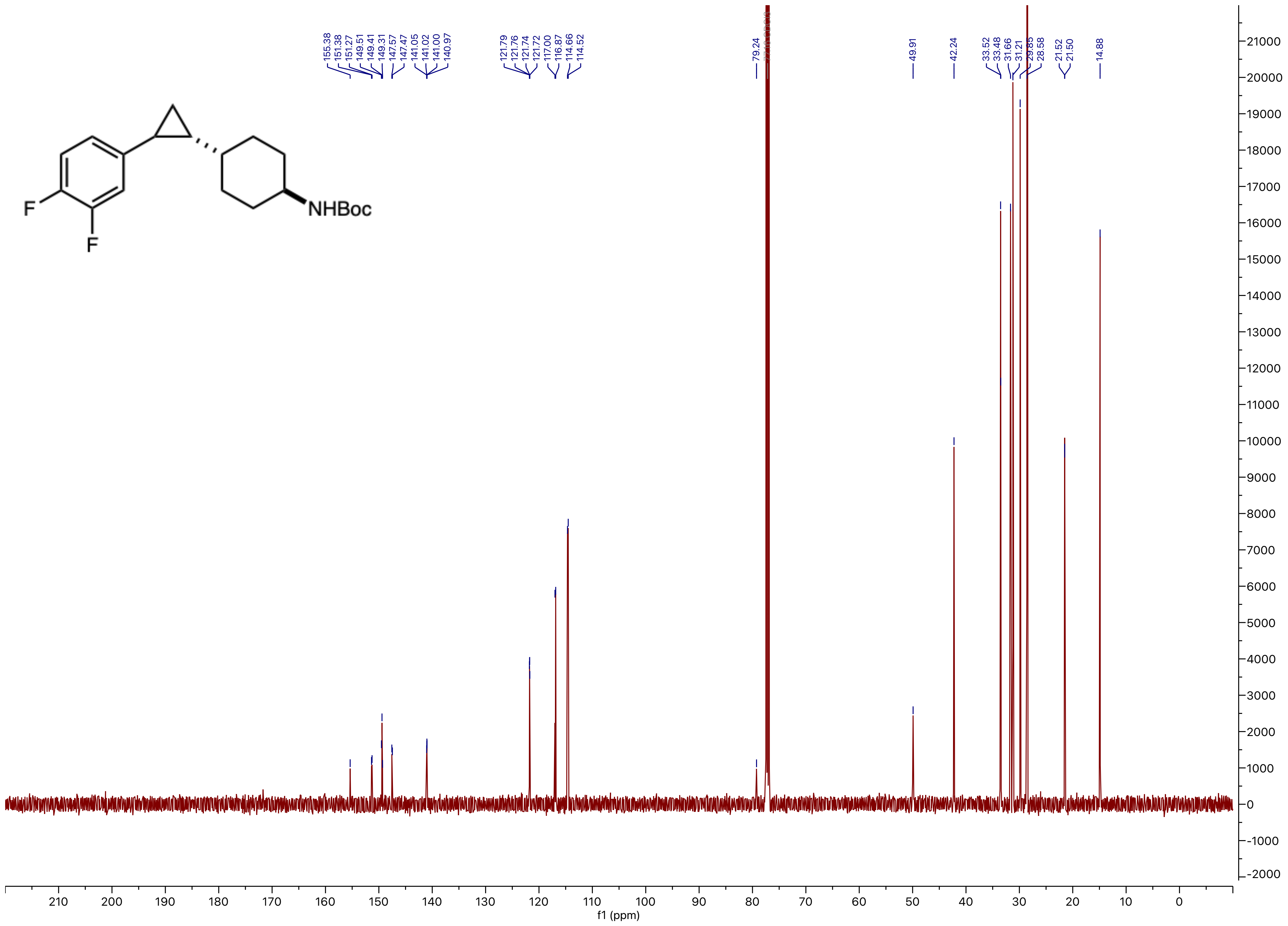

155.38
151.38
151.27
149.51
149.41
149.31
147.57
147.47
141.05
141.02
141.00
140.97
121.79
121.76
121.74
121.72
117.00
116.87
114.66
114.52
79.24
49.91
42.24
33.52
33.48
31.66
31.21
29.85
28.58
21.52
21.50
14.88
F
F
NHBoc
f1 (ppm)

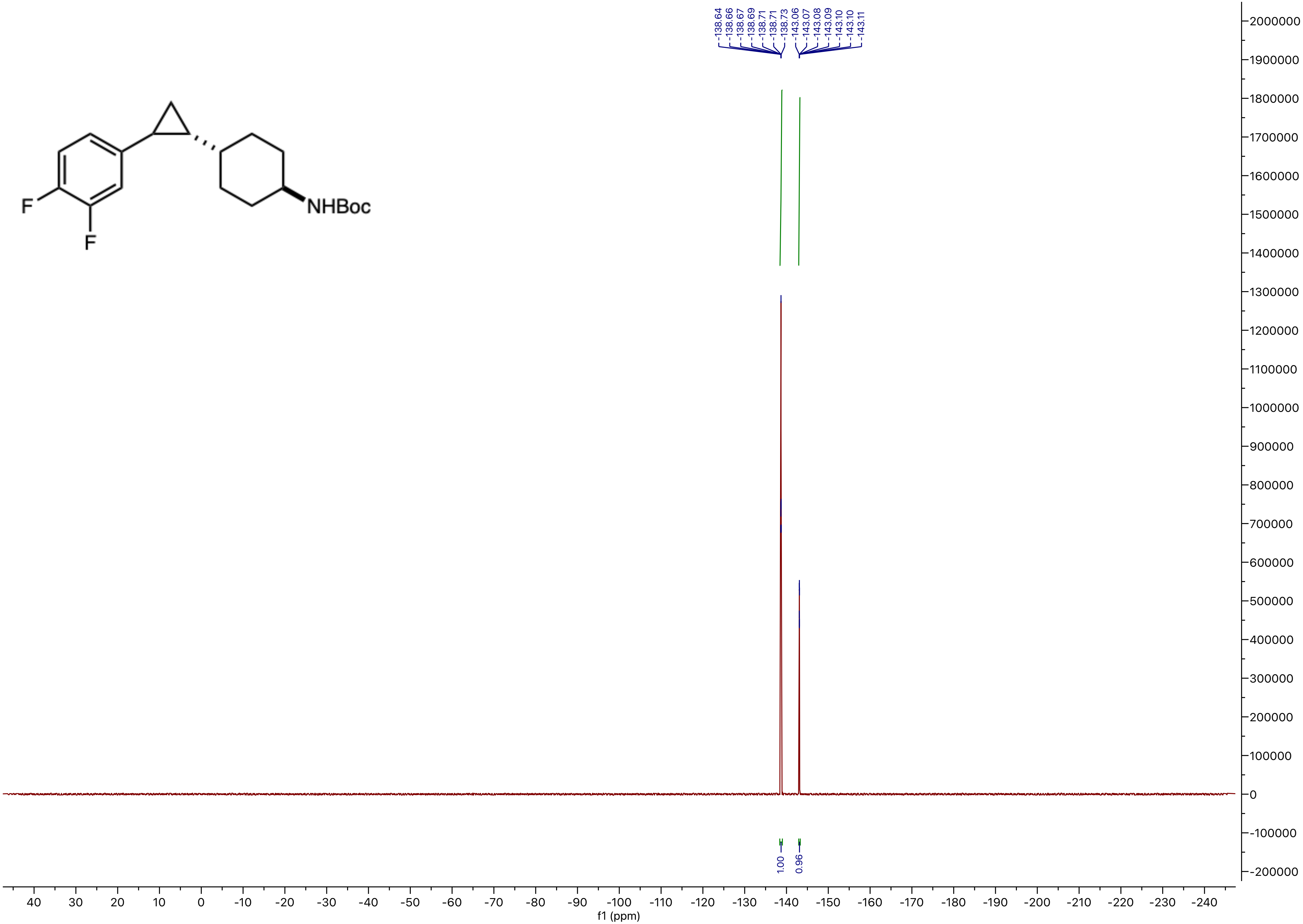
F
F
NHBoc
-138.64
-138.66
-138.67
-138.69
-138.71
-138.71
-138.73
-143.06
-143.07
-143.08
-143.09
-143.10
-143.10
-143.11
1.00
0.96
f1 (ppm)

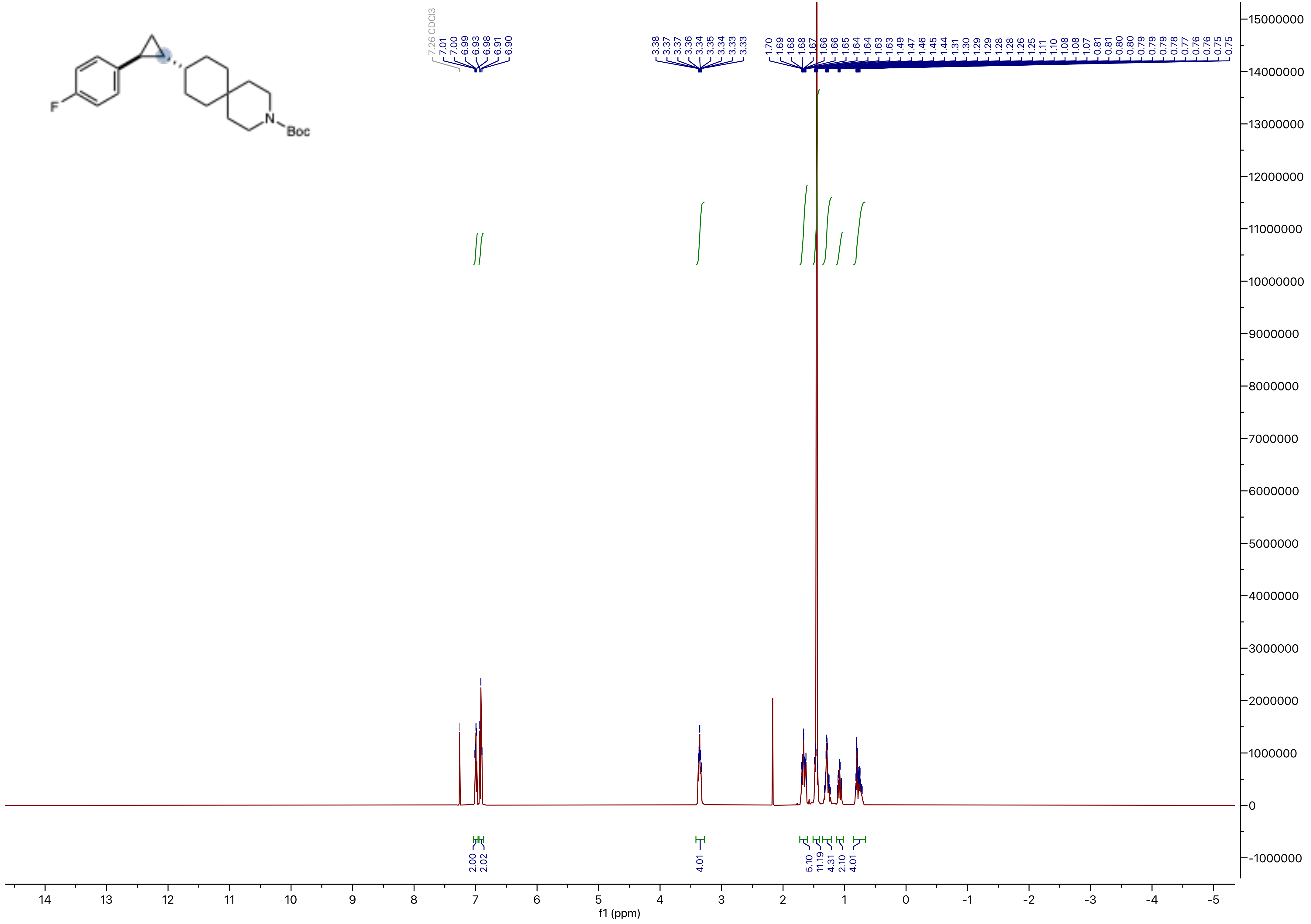
F
N
Boc
7.26 CDCl3
7.01
7.00
6.99
6.93
6.98
6.91
6.90
3.38
3.37
3.37
3.36
3.34
3.35
3.34
3.33
3.33
1.70
1.69
1.68
1.68
1.67
1.66
1.66
1.65
1.64
1.64
1.63
1.63
1.49
1.47
1.46
1.45
1.44
1.31
1.30
1.29
1.29
1.28
1.28
1.26
1.25
1.11
1.10
1.08
1.08
1.07
0.81
0.81
0.80
0.80
0.79
0.79
0.78
0.77
0.76
0.76
0.75
0.75
2.00
2.02
4.01
5.10
11.19
4.31
2.10
4.01
f1 (ppm)

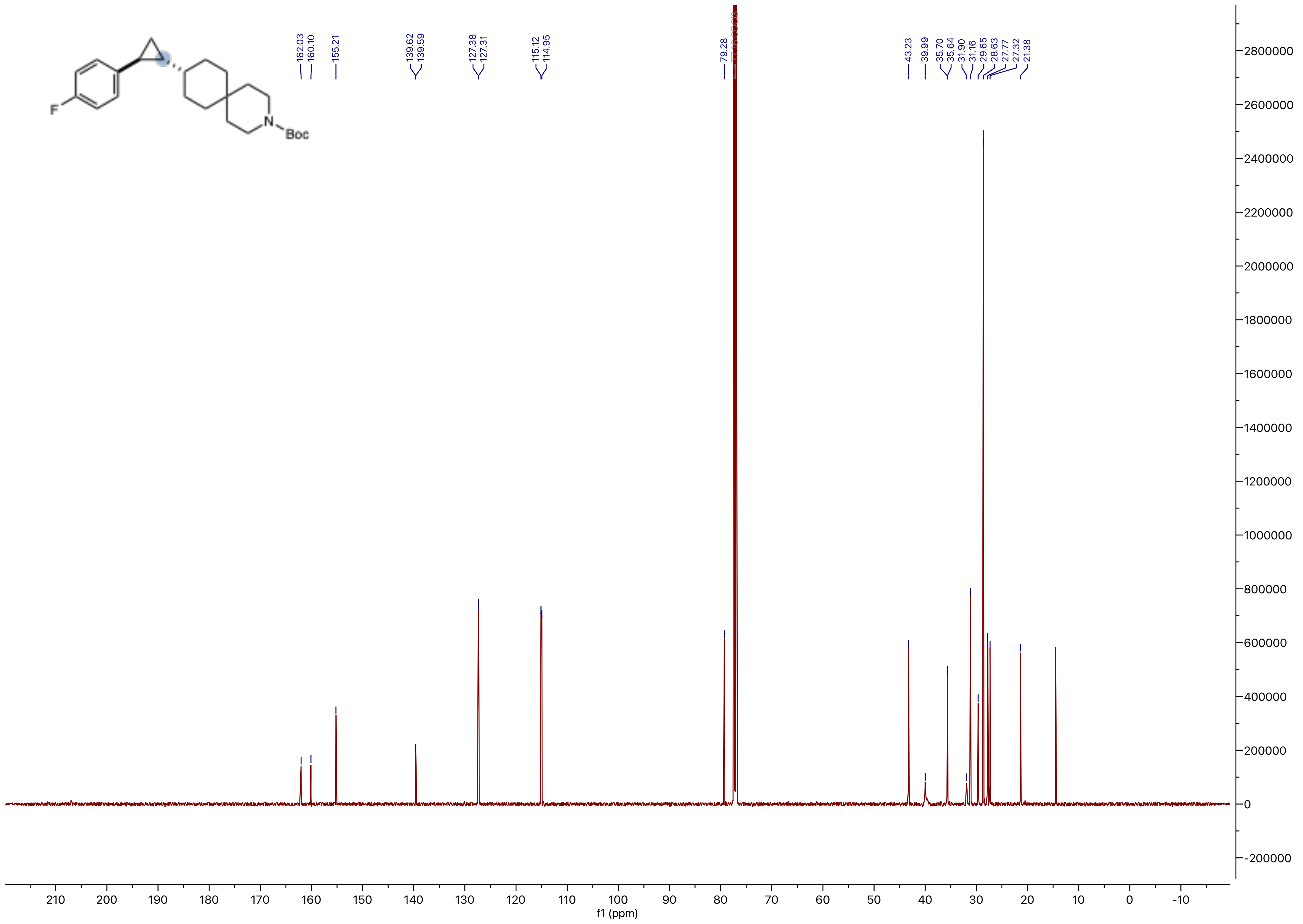
F
N
Boc
162.03
160.10
155.21
139.62
139.59
127.38
127.31
115.12
114.95
79.28
43.23
39.99
35.70
35.64
31.90
31.16
29.65
28.63
27.77
27.32
21.38
f1 (ppm)

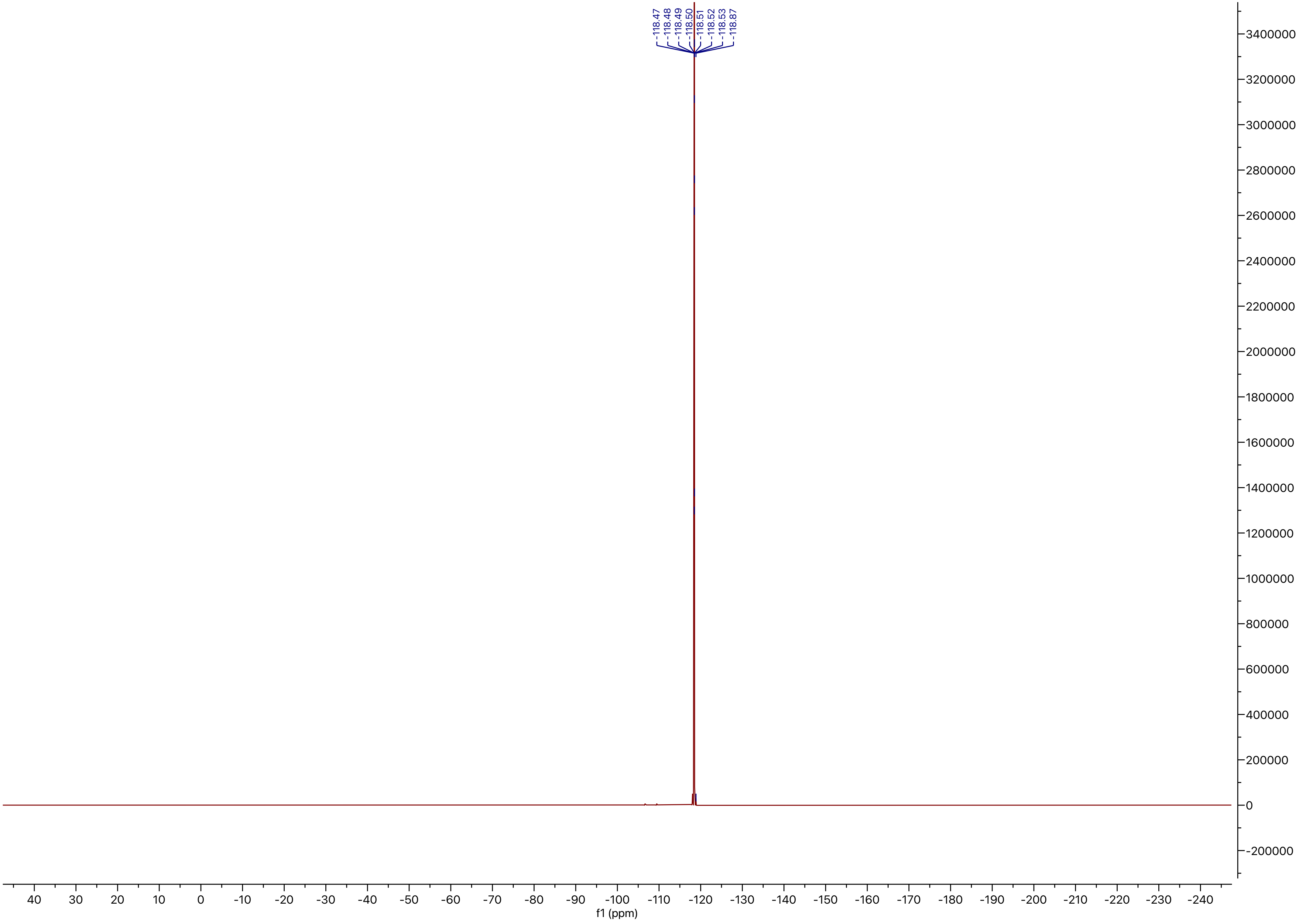

-118.47
-118.48
-118.49
-118.50
-118.51
-118.52
-118.53
-118.87
f1 (ppm)

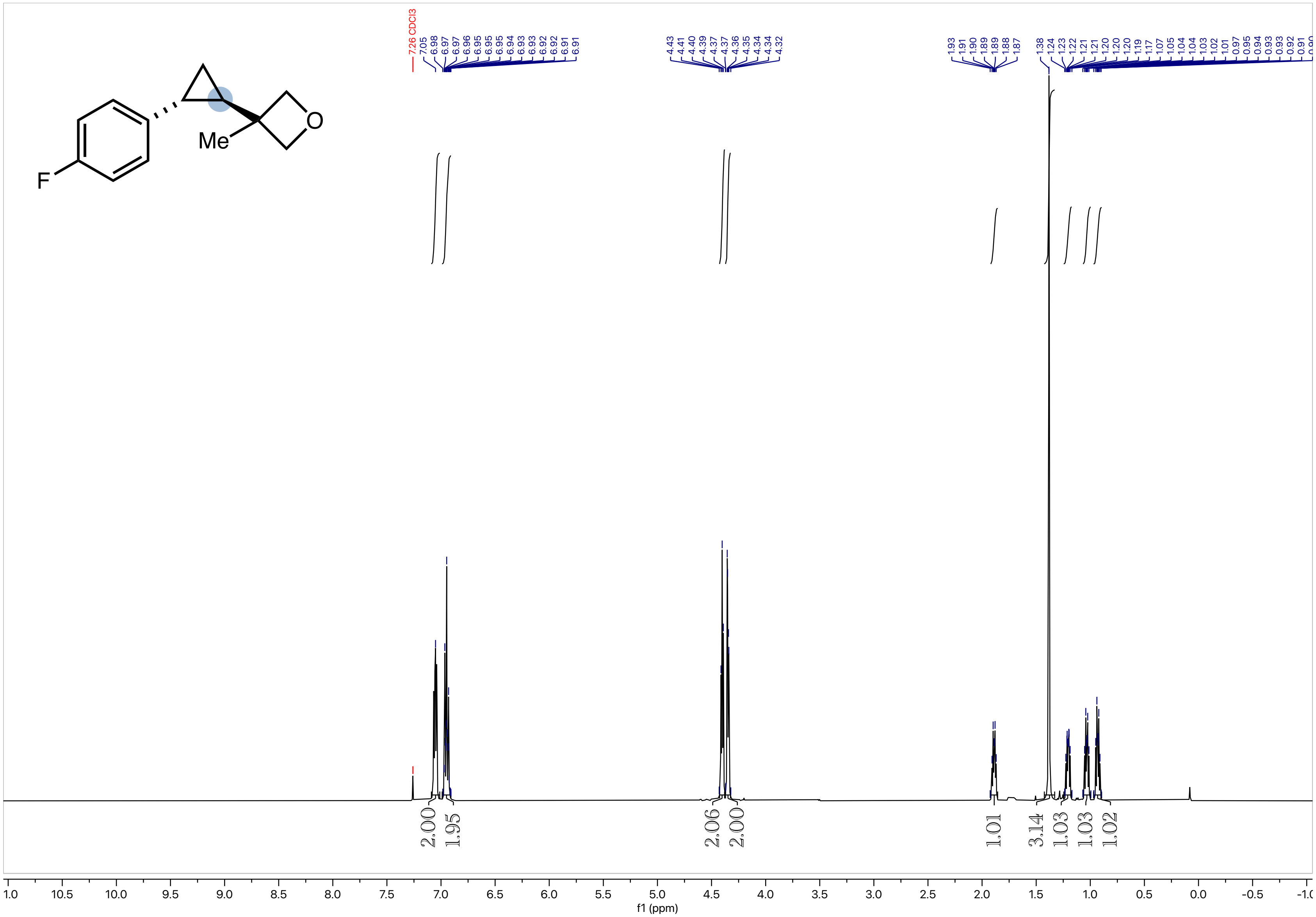

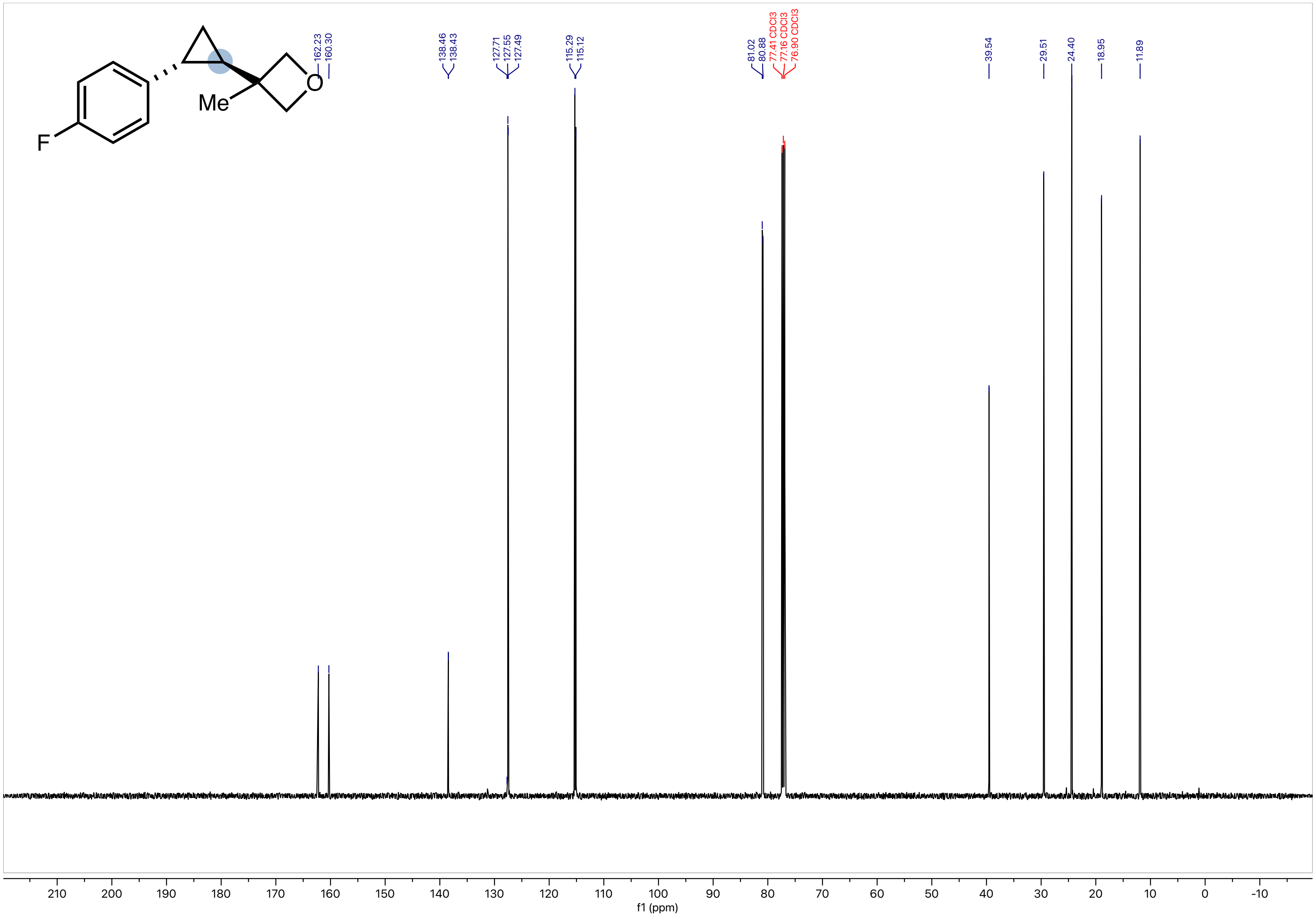

F
Me
O
162.23
160.30
138.46
138.43
127.71
127.55
127.49
115.29
115.12
81.02
80.88
77.41 CDCl3
77.16 CDCl3
76.90 CDCl3
39.54
29.51
24.40
18.95
11.89
210 200 190 180 170 160 150 140 130 120 110 100 90 80 70 60 50 40 30 20 10 0 -10
f1 (ppm)

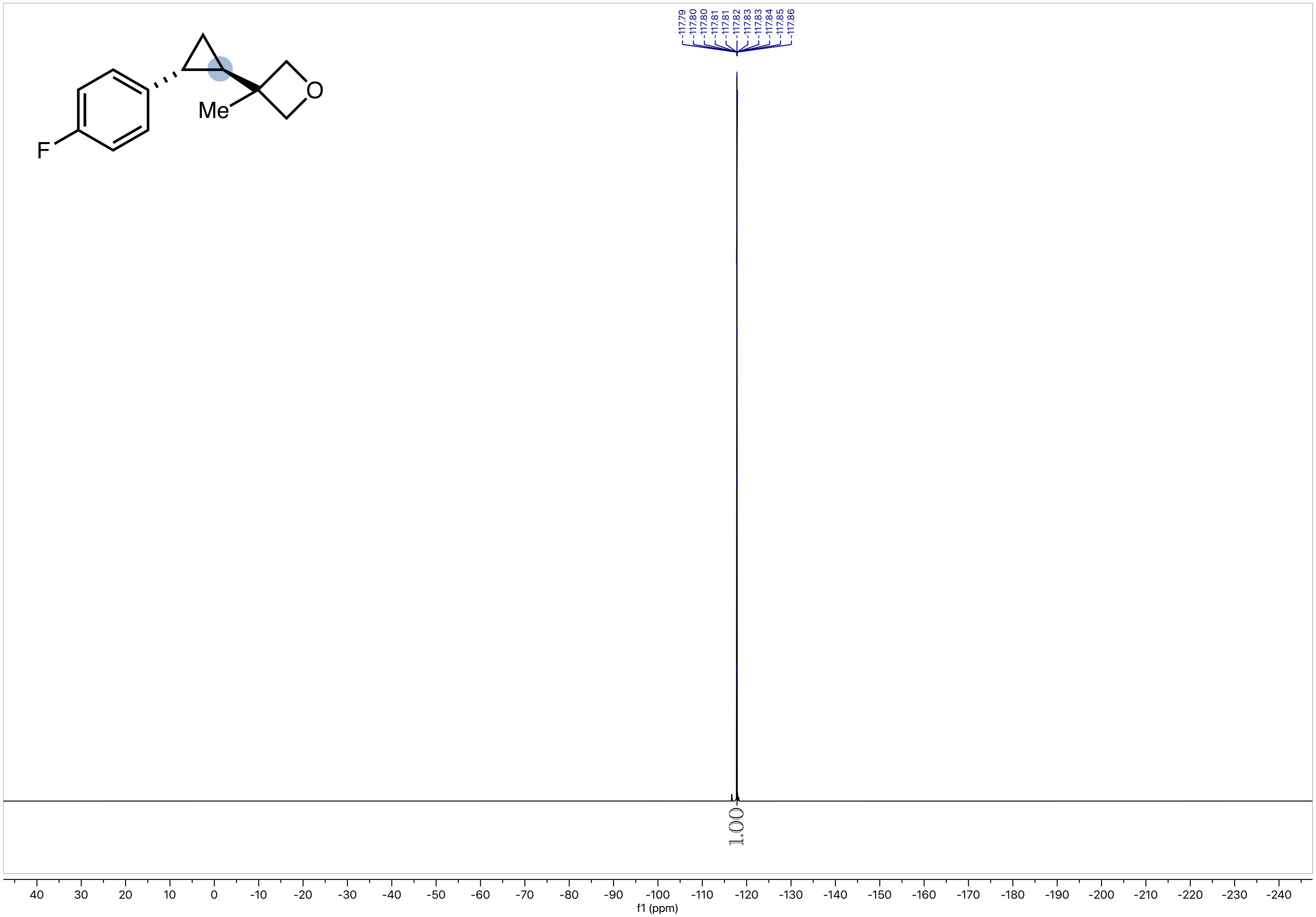

-117.79
-117.80
-117.80
-117.81
-117.81
-117.82
-117.83
-117.83
-117.84
-117.85
-117.86
F
Me
O
1.00
40 30 20 10 0 -10 -20 -30 -40 -50 -60 -70 -80 -90 -100 -110 -120 -130 -140 -150 -160 -170 -180 -190 -200 -210 -220 -230 -240
f1 (ppm)

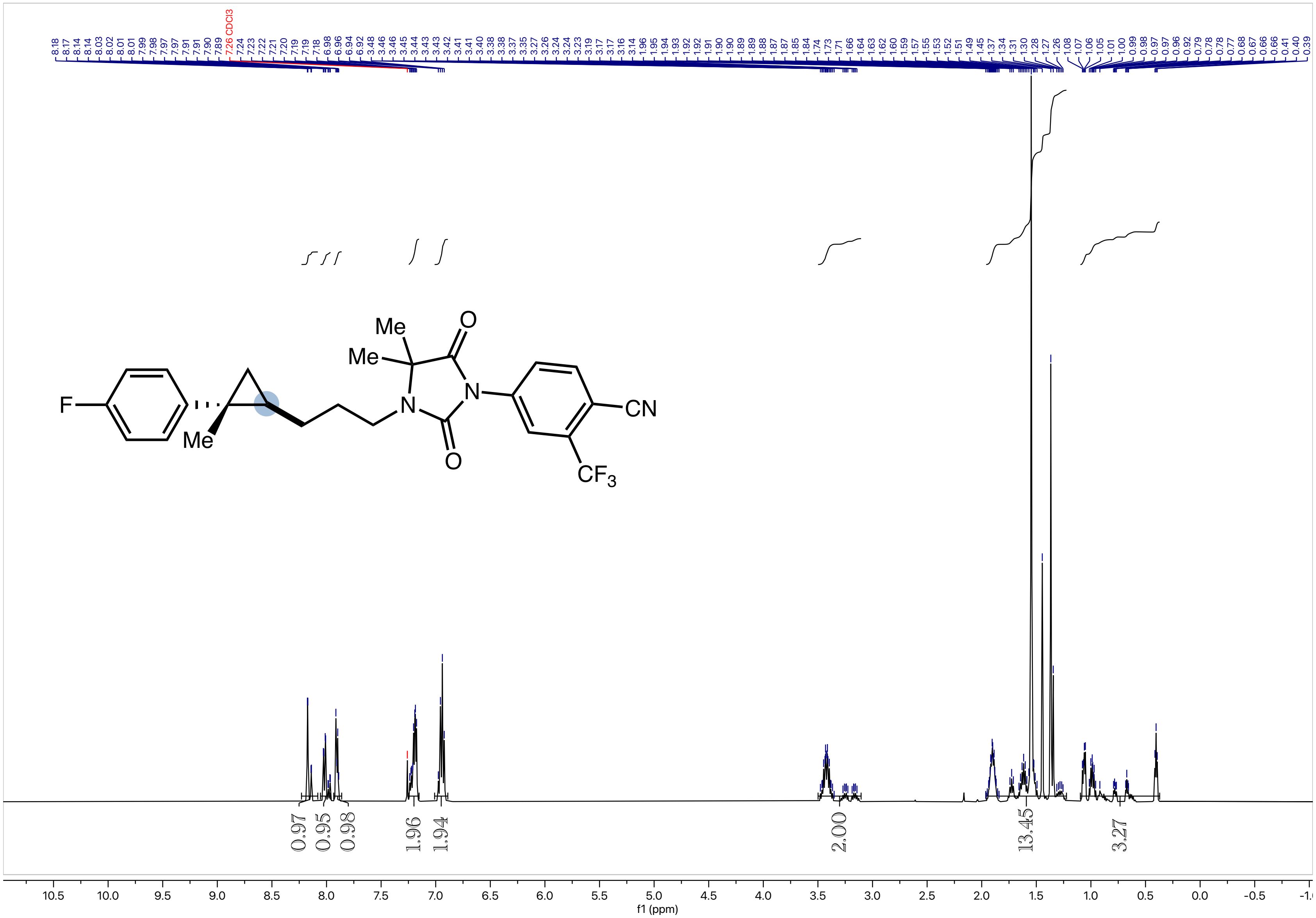
8.18 8.17 8.14 8.14 8.03 8.02 8.01 8.01 7.99 7.98 7.97 7.97 7.91 7.90 7.89 7.26 CDCl3 7.24 7.23 7.22 7.21 7.20 7.19 7.19 7.18 6.98 6.96 6.94 6.92 3.48 3.46 3.46 3.45 3.44 3.43 3.43 3.42 3.41 3.40 3.38 3.38 3.37 3.35 3.27 3.26 3.24 3.24 3.23 3.19 3.17 3.16 3.14 1.96 1.95 1.94 1.93 1.92 1.92 1.91 1.90 1.90 1.89 1.89 1.88 1.87 1.87 1.85 1.84 1.74 1.73 1.71 1.66 1.64 1.63 1.62 1.60 1.59 1.57 1.55 1.53 1.52 1.51 1.49 1.45 1.37 1.34 1.31 1.30 1.28 1.27 1.26 1.08 1.07 1.06 1.05 1.01 1.00 0.99 0.98 0.97 0.97 0.96 0.92 0.79 0.78 0.78 0.77 0.68 0.67 0.66 0.66 0.41 0.40 0.39
F
Me
Me
Me
O
O
N
N
CN
CF3
0.97
0.95
0.98
1.96
1.94
2.00
13.45
3.27
10.5 10.0 9.5 9.0 8.5 8.0 7.5 7.0 6.5 6.0 5.5 5.0 4.5 4.0 3.5 3.0 2.5 2.0 1.5 1.0 0.5 0.0 -0.5
f1 (ppm)

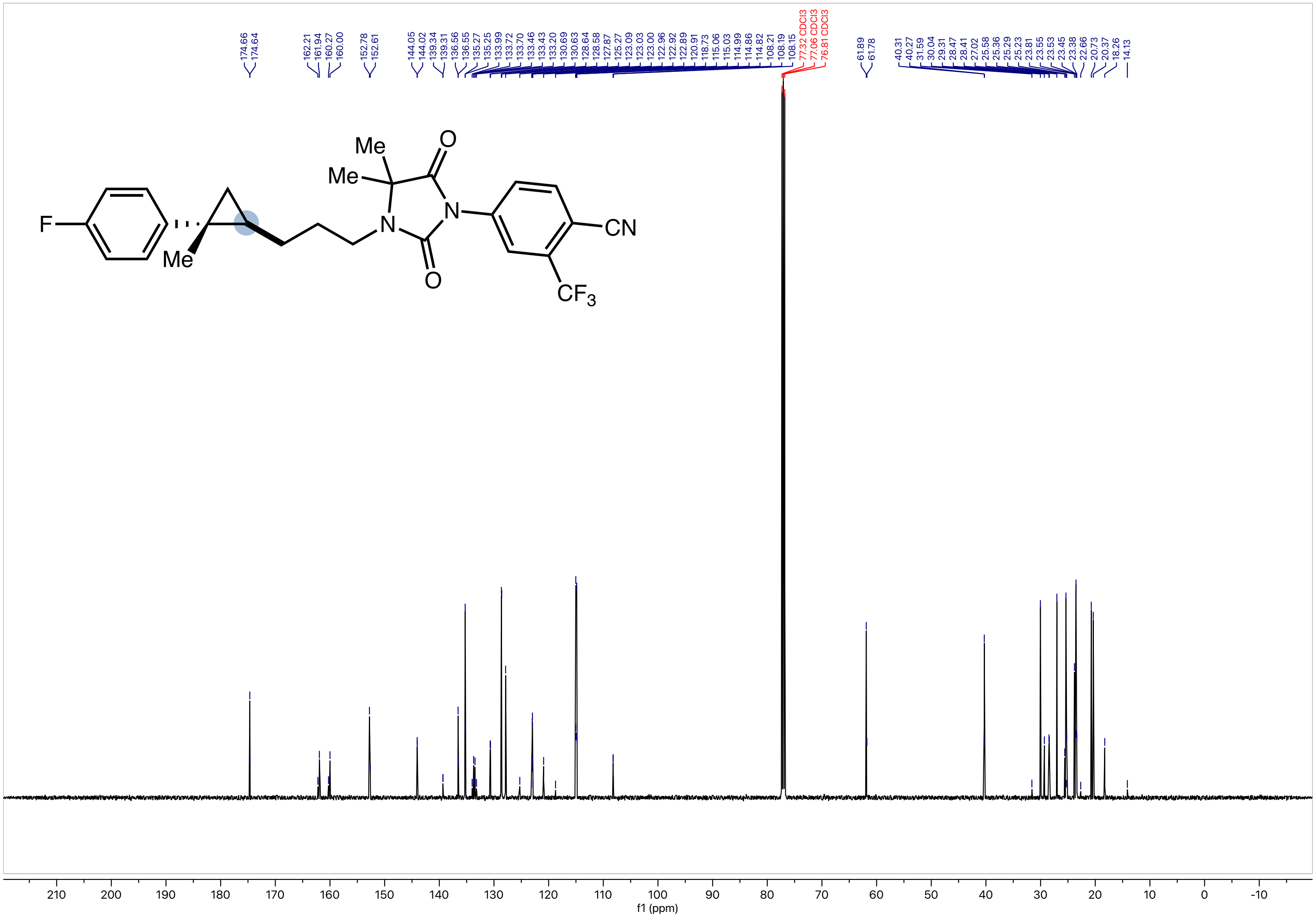

174.66
174.64
162.21
161.94
160.27
160.00
152.78
152.61
144.05
144.02
139.34
139.31
136.56
136.55
135.27
135.25
133.99
133.72
133.70
133.46
133.43
133.20
130.69
130.63
128.64
128.58
127.87
125.27
123.09
123.03
123.00
122.96
122.92
122.89
120.91
118.73
115.06
115.03
114.99
114.86
114.82
108.21
108.19
108.15
77.32 CDCl3
77.06 CDCl3
76.81 CDCl3
61.89
61.78
40.31
40.27
31.59
30.04
29.31
28.47
28.41
27.02
25.58
25.36
25.29
25.23
23.81
23.55
23.53
23.45
23.38
22.66
20.73
20.37
18.26
14.13
F
Me
Me
Me
O
N
N
O
CN
CF3
210
200
190
180
170
160
150
140
130
120
110
100
90
80
70
60
50
40
30
20
10
0
-10
f1 (ppm)

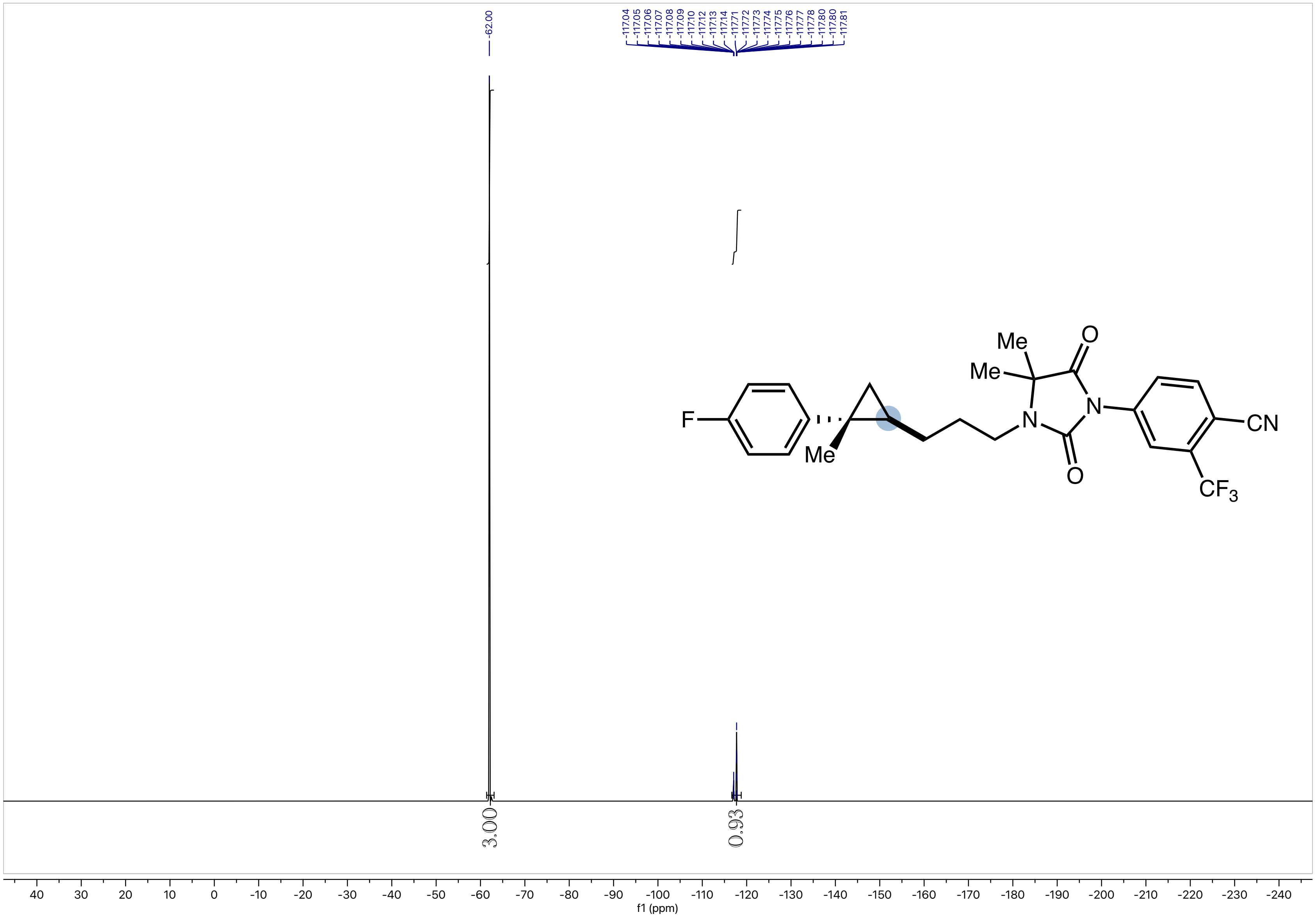

-62.00
-117.04
-117.05
-117.06
-117.07
-117.08
-117.09
-117.10
-117.12
-117.13
-117.14
-117.71
-117.72
-117.73
-117.74
-117.75
-117.76
-117.77
-117.78
-117.80
-117.81
3.00
0.93
F
Me
Me
Me
O
O
N
N
CN
CF3
40 30 20 10 0 -10 -20 -30 -40 -50 -60 -70 -80 -90 -100 -110 -120 -130 -140 -150 -160 -170 -180 -190 -200 -210 -220 -230 -240
f1 (ppm)

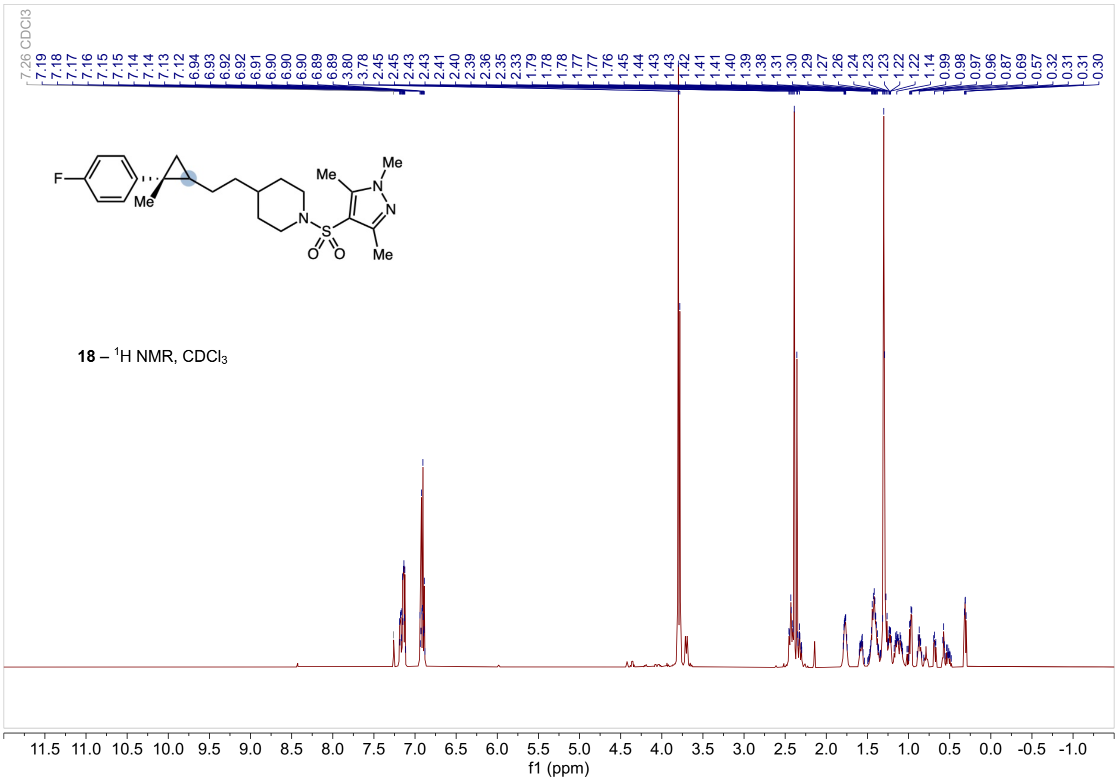
7.26 CDCl3
7.19 7.18 7.17 7.16 7.15 7.15 7.14 7.14 7.13 7.12 6.94 6.93 6.92 6.92 6.91 6.90 6.90 6.90 6.89 6.89 3.80 3.78 2.45 2.45 2.43 2.43 2.41 2.40 2.39 2.36 2.35 2.33 1.79 1.78 1.78 1.77 1.77 1.76 1.45 1.44 1.43 1.43 1.42 1.41 1.40 1.39 1.38 1.31 1.30 1.29 1.27 1.26 1.24 1.23 1.23 1.22 1.22 1.14 0.99 0.98 0.97 0.96 0.87 0.69 0.57 0.32 0.31 0.30
F
Me
Me
Me
N
N
N
S
O
O
Me
18 – 1H NMR, CDCl3
11.5 11.0 10.5 10.0 9.5 9.0 8.5 8.0 7.5 7.0 6.5 6.0 5.5 5.0 4.5 4.0 3.5 3.0 2.5 2.0 1.5 1.0 0.5 0.0 -0.5 -1.0
f1 (ppm)

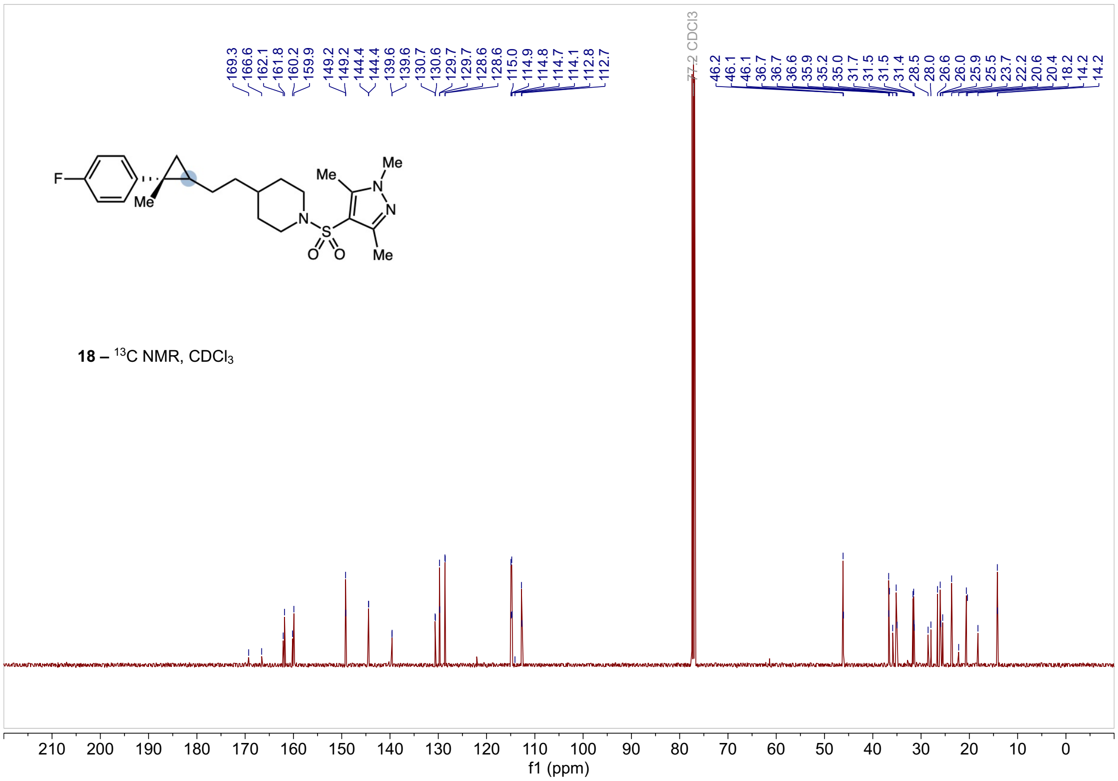
169.3 166.6 162.1 161.8 160.2 159.9 149.2 149.2 144.4 144.4 139.6 139.6 130.7 130.6 129.7 129.7 128.6 128.6 115.0 114.9 114.7 114.1 112.8 112.7
77.2 CDCl3
46.2 46.1 46.1 36.7 36.6 35.9 35.2 35.0 31.7 31.5 31.4 28.5 28.0 26.6 26.0 25.9 25.5 23.7 22.2 20.6 20.4 18.2 14.2 14.2
F
Me
Me
Me
N
N
N
S
O
O
Me
18 – 13C NMR, CDCl3
210 200 190 180 170 160 150 140 130 120 110 100 90 80 70 60 50 40 30 20 10 0
f1 (ppm)

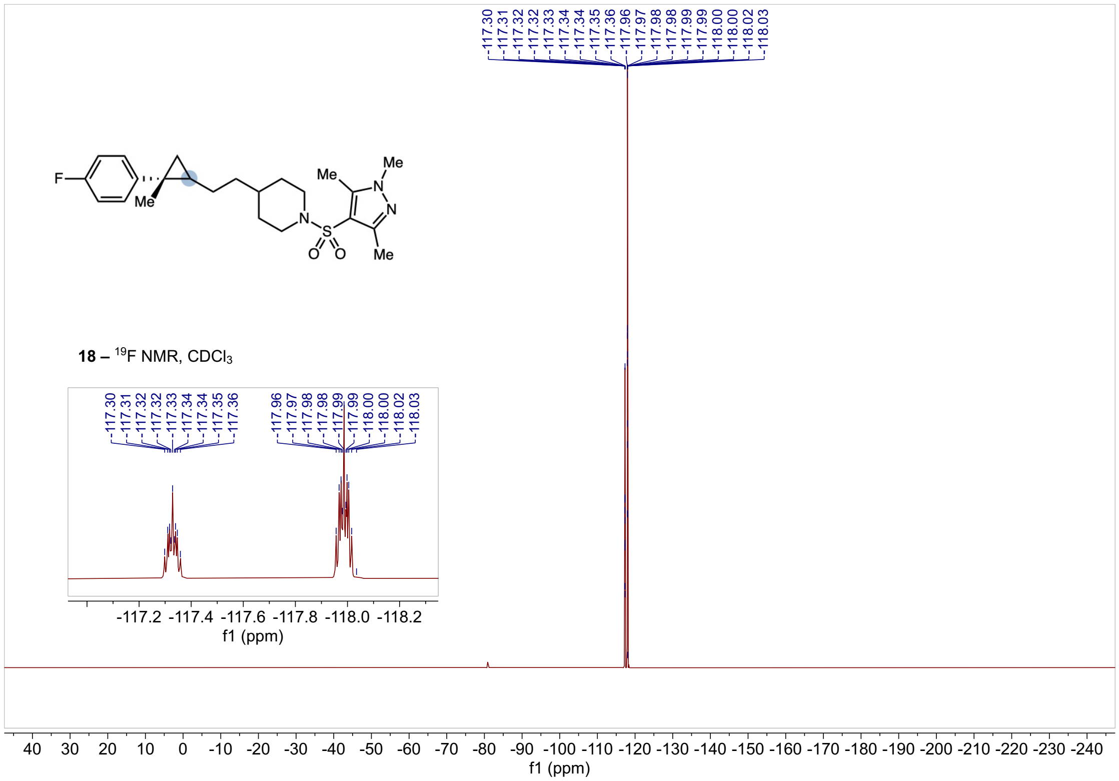

18 – 19F NMR, CDCl3
-117.30 -117.31 -117.32 -117.32 -117.33 -117.34 -117.34 -117.35 -117.36 -117.96 -117.97 -117.98 -117.98 -117.99 -117.99 -118.00 -118.00 -118.02 -118.03
-117.2 -117.4 -117.6 -117.8 -118.0 -118.2
f1 (ppm)
40 30 20 10 0 -10 -20 -30 -40 -50 -60 -70 -80 -90 -100 -110 -120 -130 -140 -150 -160 -170 -180 -190 -200 -210 -220 -230 -240
f1 (ppm)

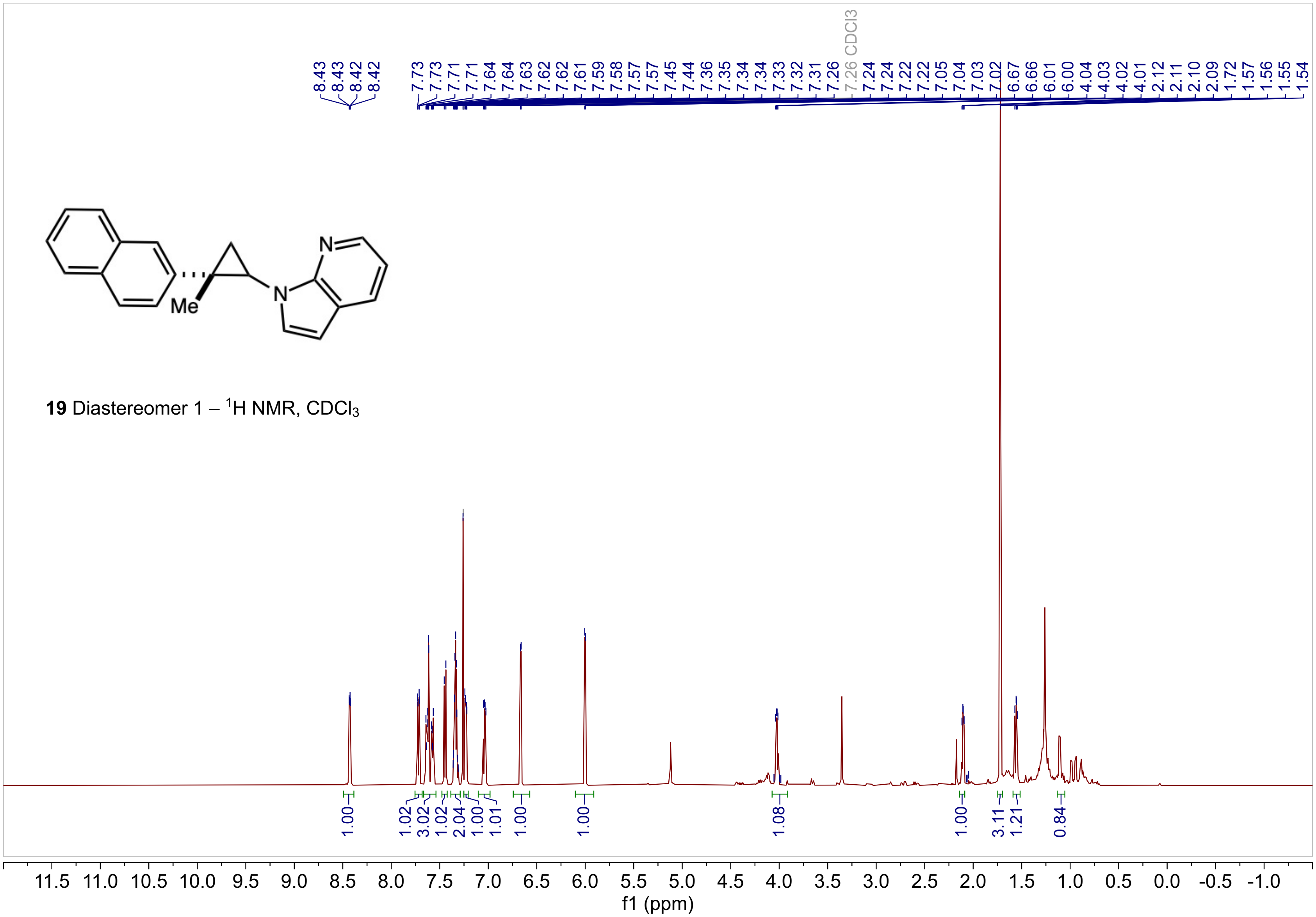

19 Diastereomer 1 – ^{1}H NMR, $CDCl_3$

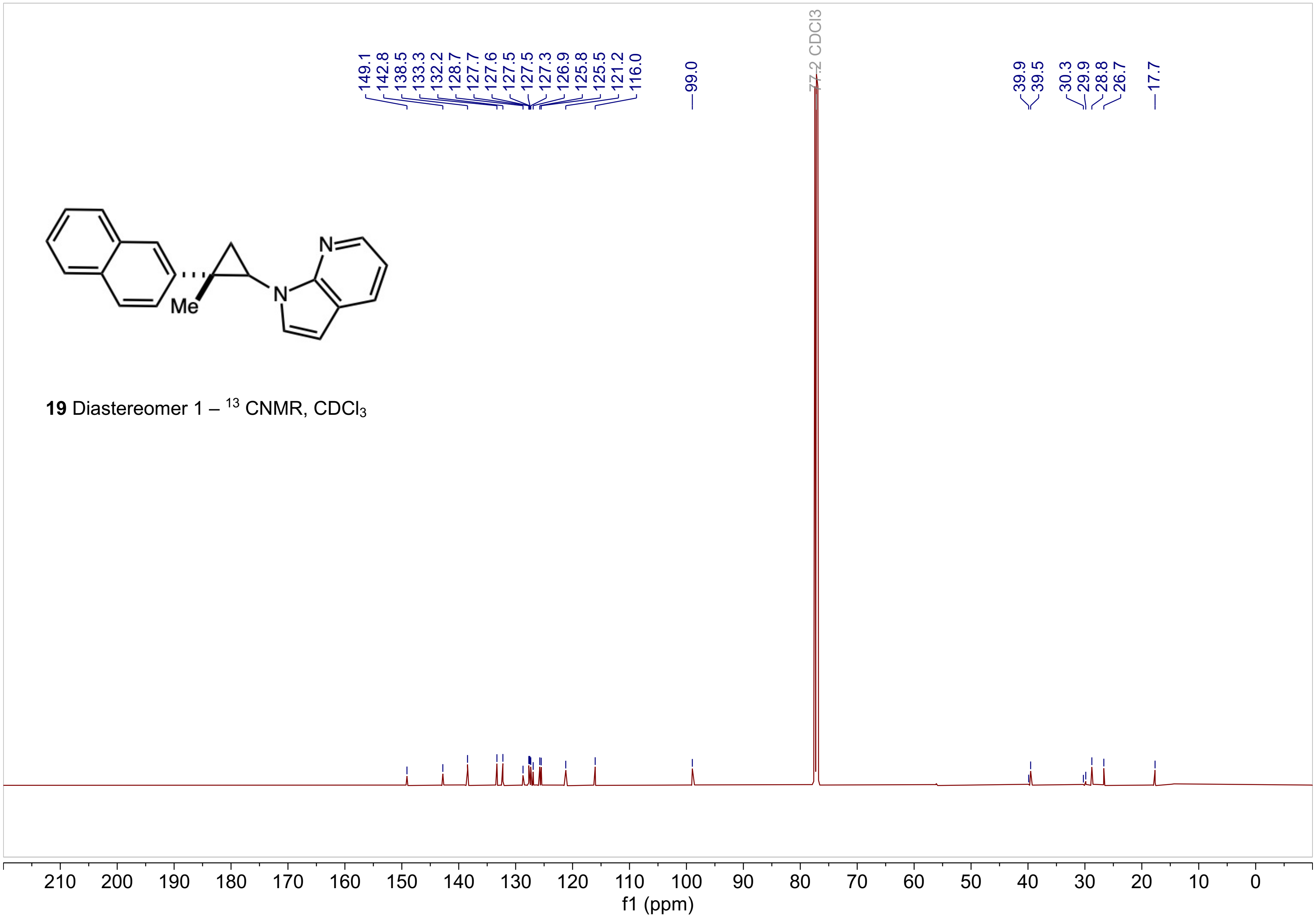
149.1
142.8
138.5
133.3
132.2
128.7
127.7
127.6
127.5
127.5
127.3
126.9
125.8
125.5
121.2
116.0
99.0
77.2 CDCl3
39.9
39.5
30.3
29.9
28.8
26.7
17.7
N
N
Me
19 Diastereomer 1 – 13 CNMR, CDCl3
210
200
190
180
170
160
150
140
130
120
110
100
90
80
70
60
50
40
30
20
10
0
f1 (ppm)

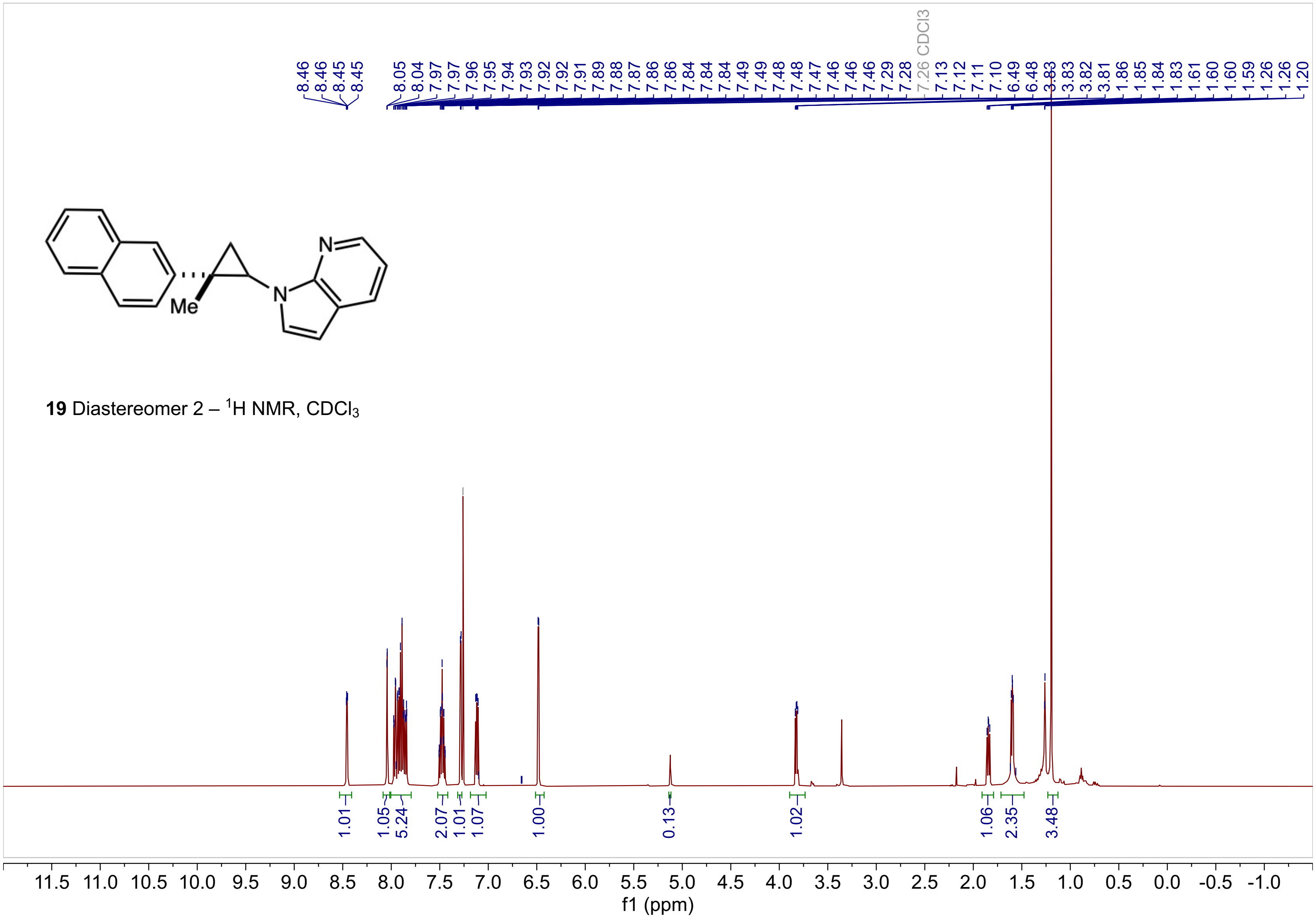

19 Diastereomer 2 – ^{1}H NMR, $CDCl_3$

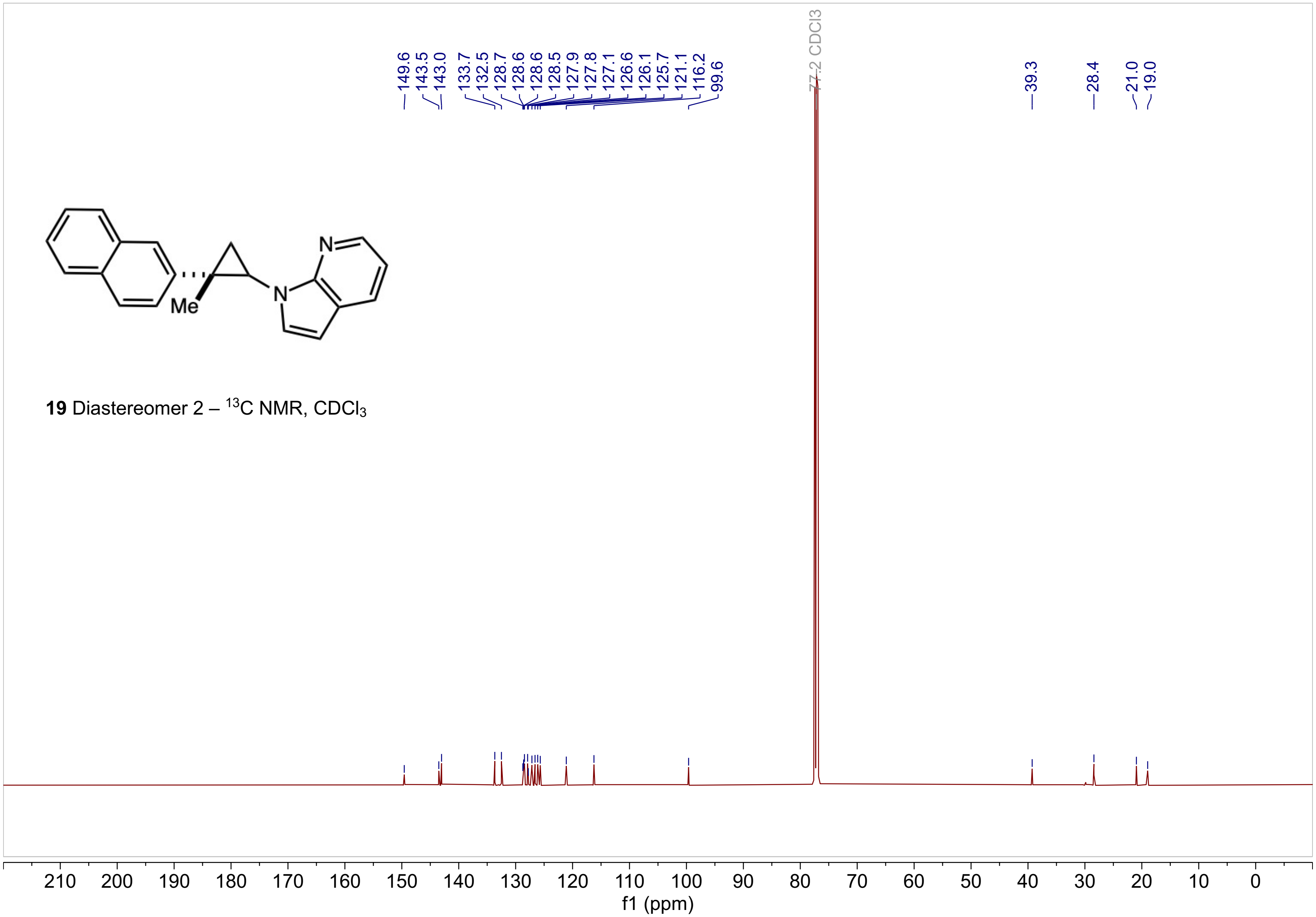

19 Diastereomer 2 – ^{13}C NMR, $CDCl_3$

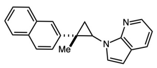

19 Diastereomer 2 – HSQC, $CDCl_3$

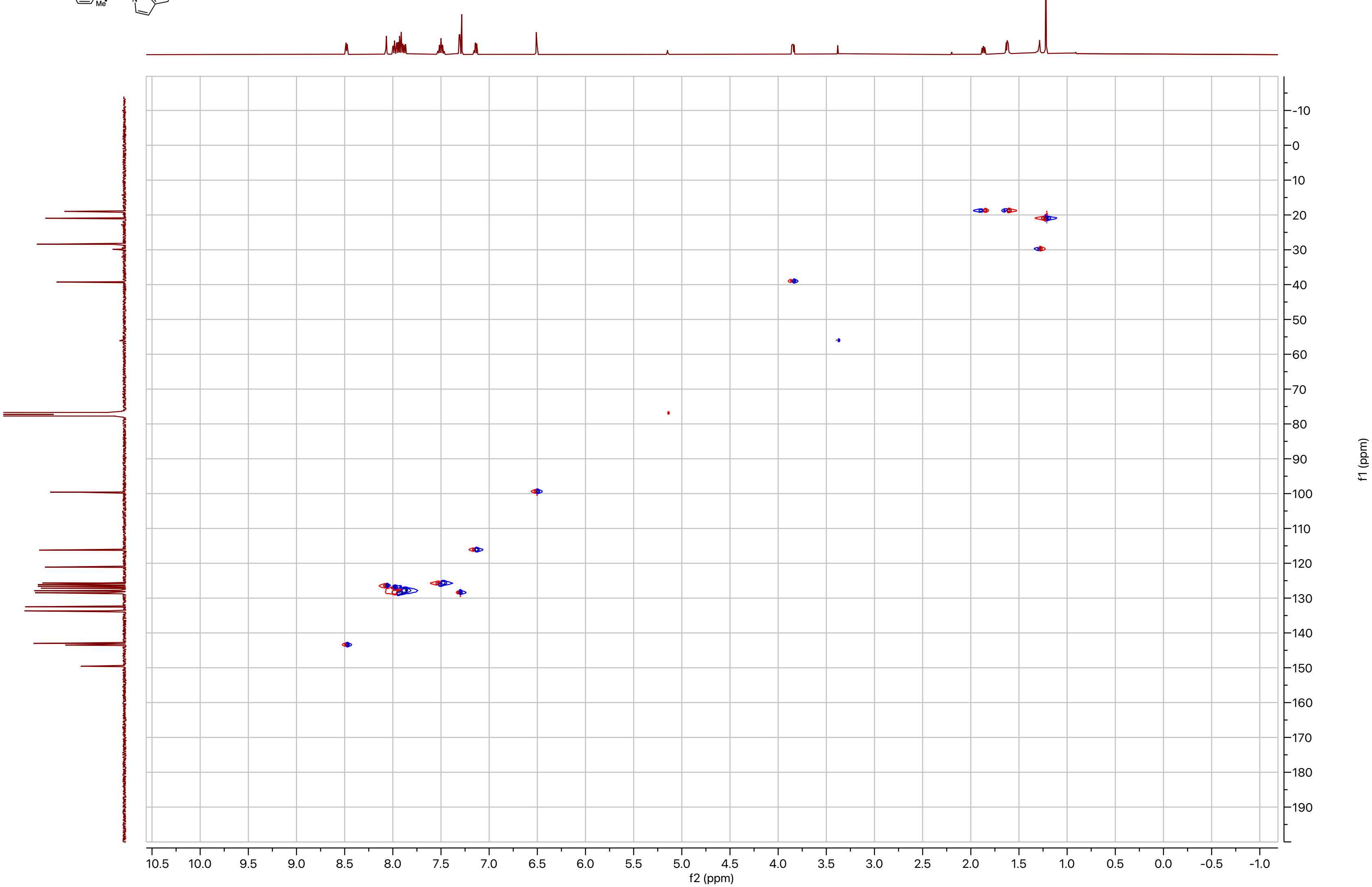

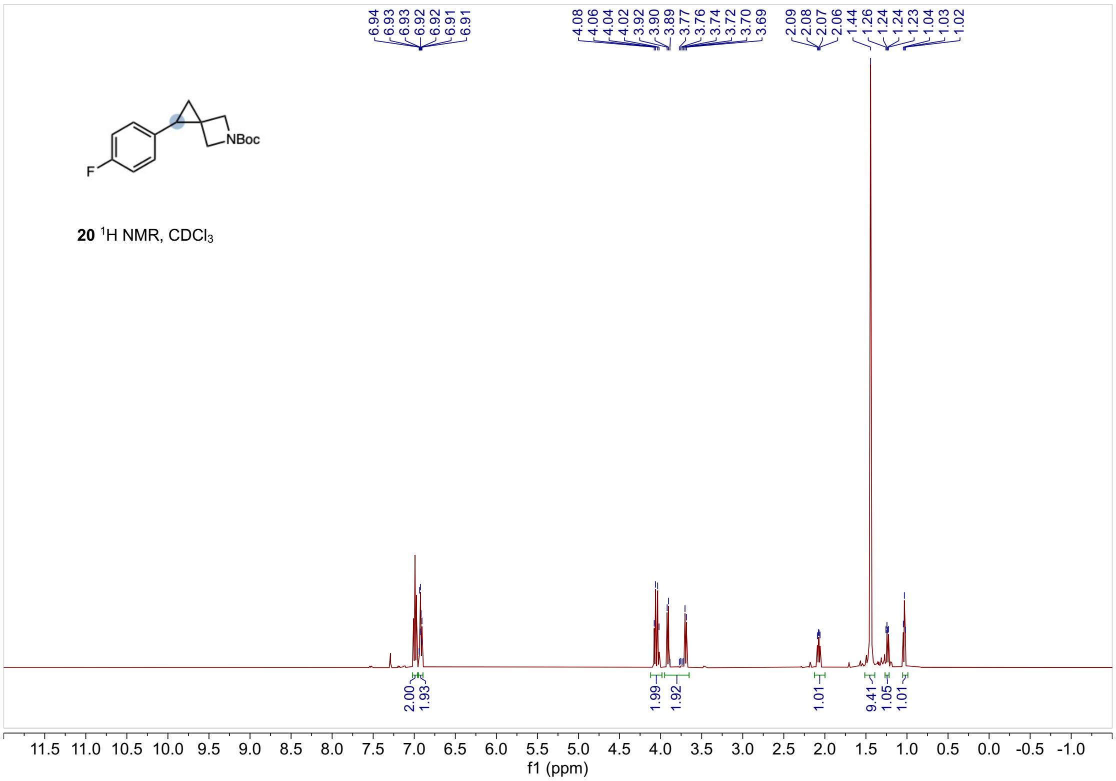
6.94
6.93
6.93
6.92
6.92
6.91
6.91
4.08
4.06
4.04
4.02
3.92
3.90
3.89
3.77
3.76
3.74
3.72
3.70
3.69
2.09
2.08
2.07
2.06
1.44
1.26
1.24
1.24
1.23
1.04
1.03
1.02
NBoc
F
20 ¹H NMR, CDCl3
2.00
1.93
1.99
1.92
1.01
9.41
1.05
1.01
11.5 11.0 10.5 10.0 9.5 9.0 8.5 8.0 7.5 7.0 6.5 6.0 5.5 5.0 4.5 4.0 3.5 3.0 2.5 2.0 1.5 1.0 0.5 0.0 -0.5 -1.0
f1 (ppm)

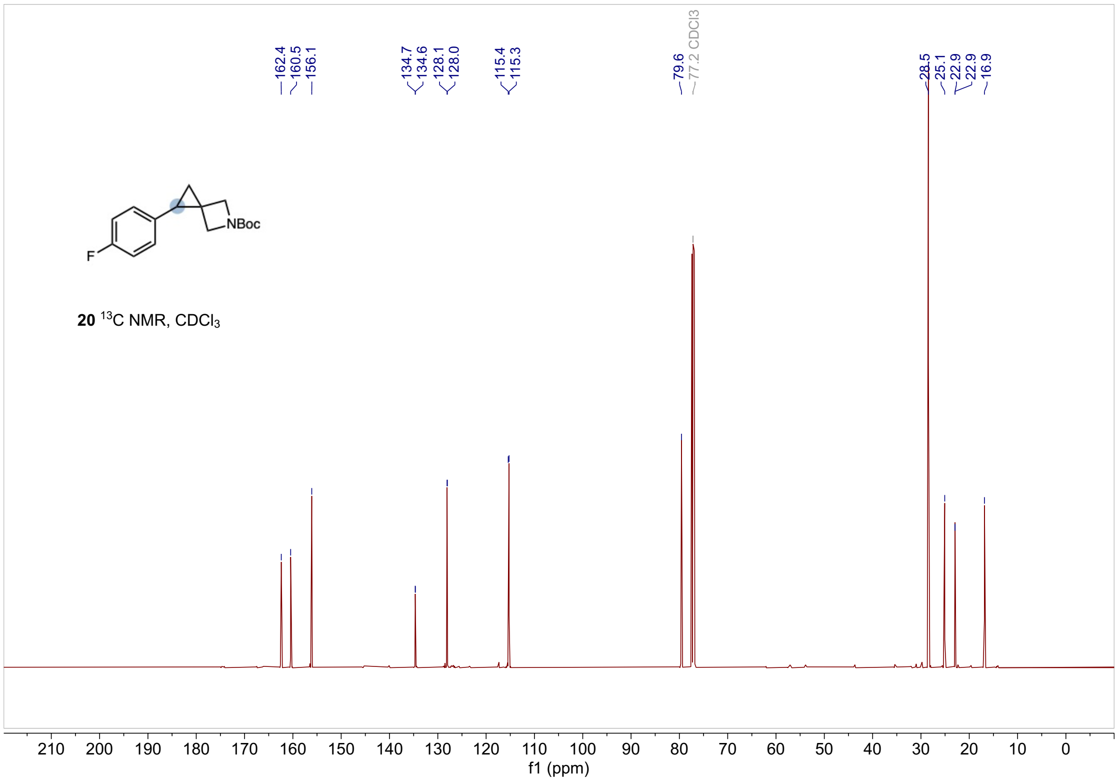
162.4
160.5
156.1
134.7
134.6
128.1
128.0
115.4
115.3
79.6
77.2 CDCl3
28.5
25.1
22.9
22.9
16.9
NBoc
F
20 ¹³C NMR, CDCl3
210 200 190 180 170 160 150 140 130 120 110 100 90 80 70 60 50 40 30 20 10 0
f1 (ppm)

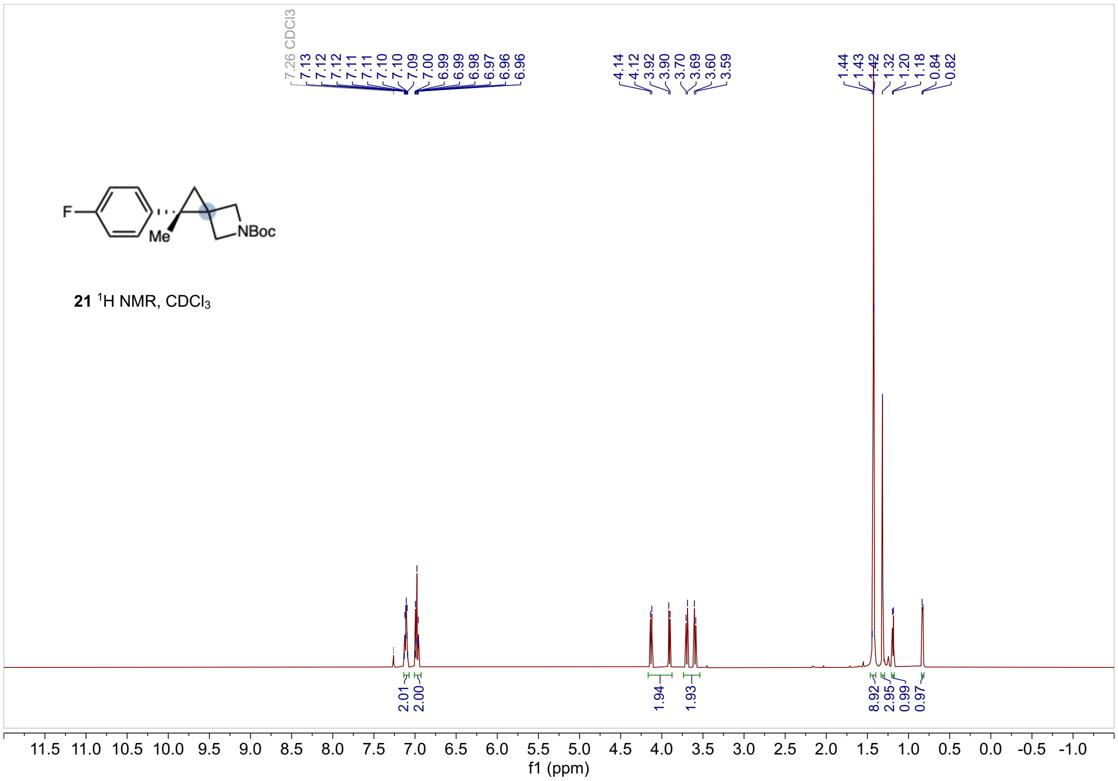
F
Me
NBoc
21 ^{1}H NMR, $CDCl_3$
7.26 CDCl3
7.13
7.12
7.12
7.11
7.11
7.10
7.10
7.09
7.00
6.99
6.99
6.98
6.97
6.96
6.96
4.14
4.12
3.92
3.90
3.70
3.69
3.60
3.59
1.44
1.43
1.42
1.32
1.20
1.18
0.84
0.82
2.01
2.00
1.94
1.93
8.92
2.95
0.99
0.97
f1 (ppm)

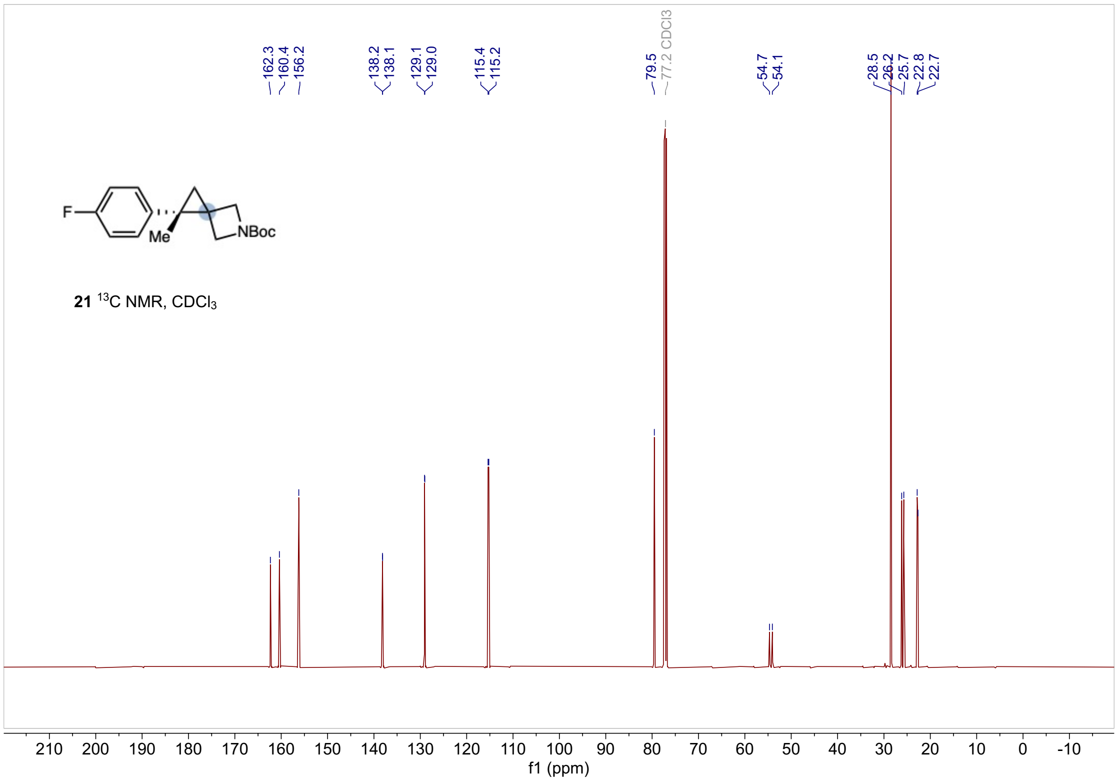
F
Me
NBoc
21 ^{13}C NMR, $CDCl_3$
162.3
160.4
156.2
138.2
138.1
129.1
129.0
115.4
115.2
79.5
77.2 CDCl3
54.7
54.1
28.5
26.2
25.7
22.8
22.7
f1 (ppm)

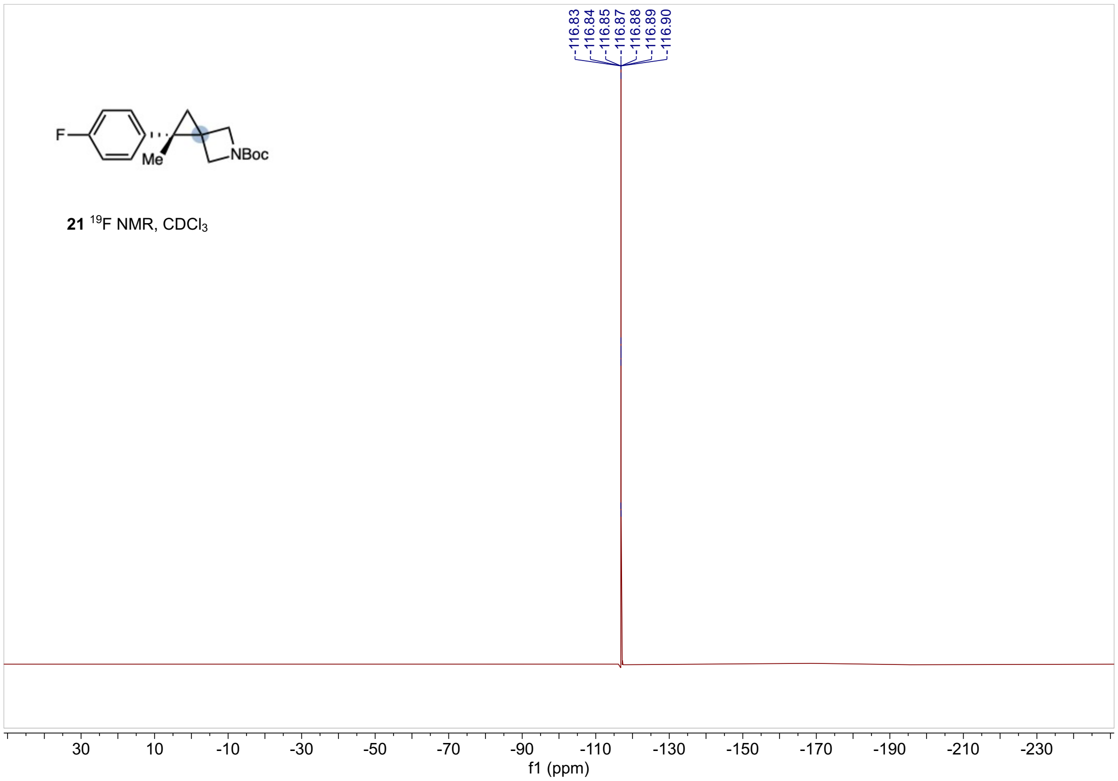

21 ^{19}F NMR, $CDCl_3$

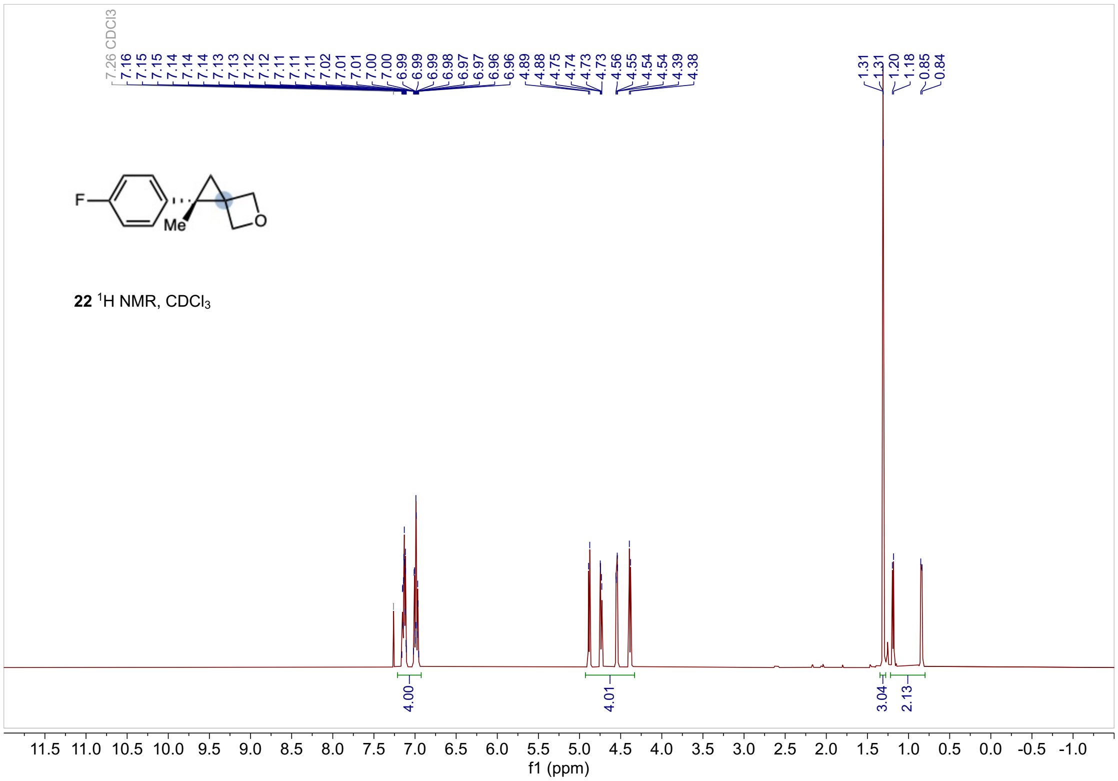

7.26 CDCl3
7.16 7.15 7.15 7.14 7.14 7.14 7.13 7.13 7.12 7.12 7.11 7.11 7.11 7.02 7.01 7.01 7.00 7.00 6.99 6.99 6.99 6.98 6.97 6.96 6.96 4.89 4.88 4.75 4.74 4.73 4.73 4.56 4.55 4.54 4.54 4.39 4.38
1.31 1.31 1.20 1.18 0.85 0.84
F
Me
O
22 ¹H NMR, CDCl₃
4.00
4.01
3.04
2.13
11.5 11.0 10.5 10.0 9.5 9.0 8.5 8.0 7.5 7.0 6.5 6.0 5.5 5.0 4.5 4.0 3.5 3.0 2.5 2.0 1.5 1.0 0.5 0.0 -0.5 -1.0
f1 (ppm)

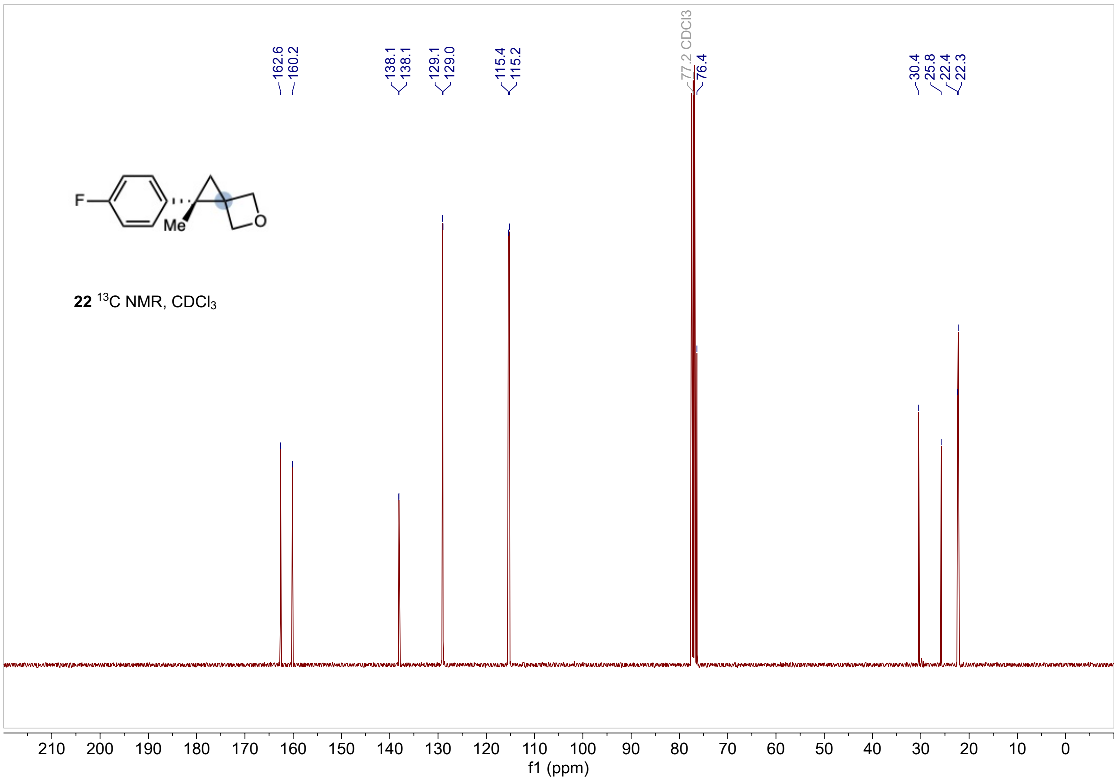

162.6 160.2
138.1 138.1
129.1 129.0
115.4 115.2
77.2 CDCl3
76.4
30.4 25.8 22.4 22.3
F
Me
O
22 ¹³C NMR, CDCl₃
210 200 190 180 170 160 150 140 130 120 110 100 90 80 70 60 50 40 30 20 10 0
f1 (ppm)

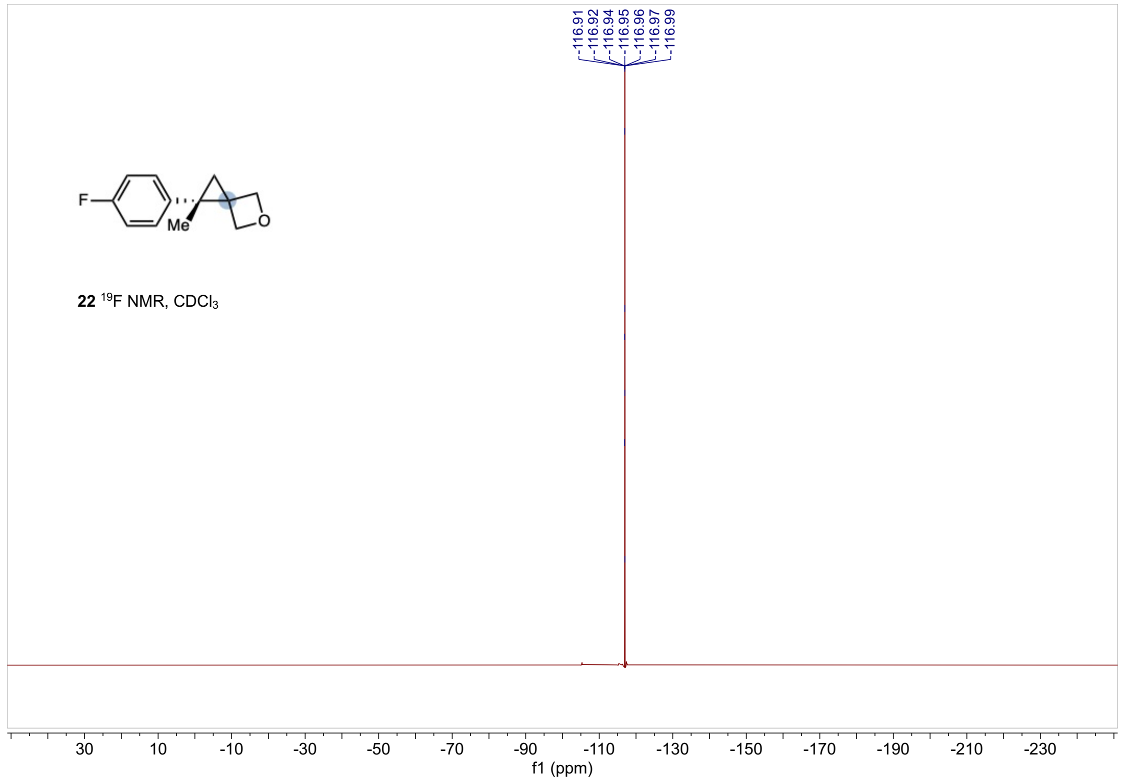

-116.91
-116.92
-116.94
-116.95
-116.96
-116.97
-116.99
F
Me
O
22 19F NMR, CDCl3
30 10 -10 -30 -50 -70 -90 -110 -130 -150 -170 -190 -210 -230
f1 (ppm)

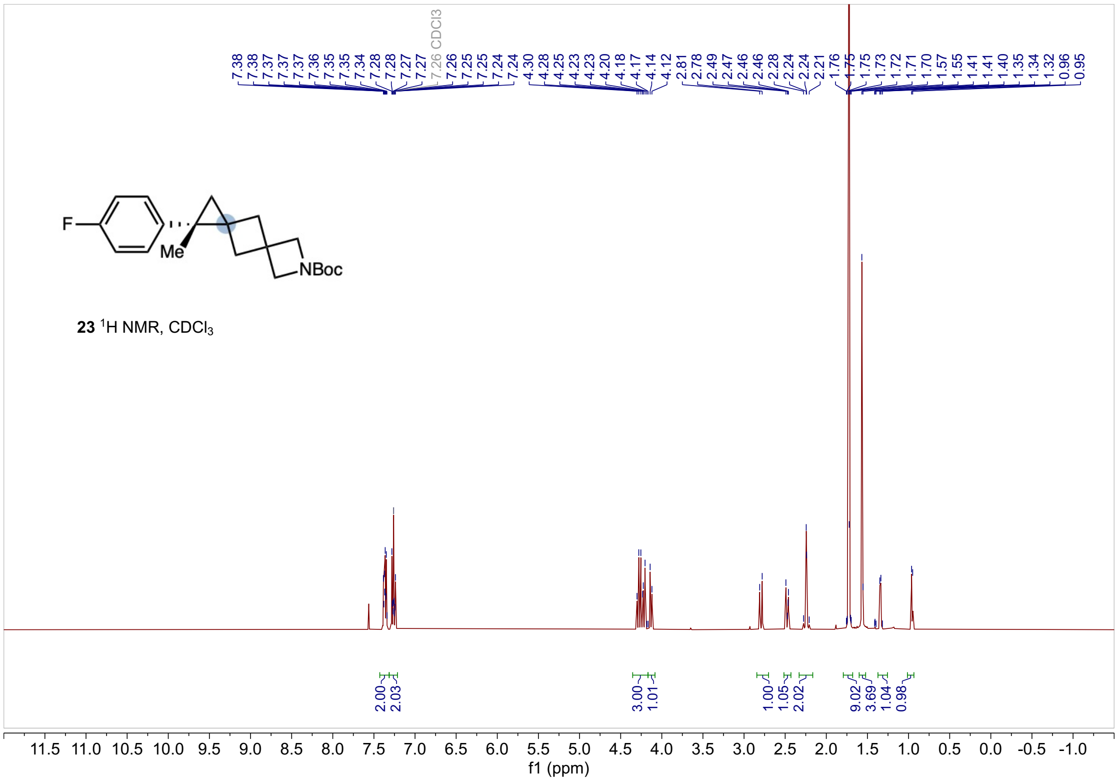

7.38
7.38
7.37
7.37
7.37
7.36
7.35
7.35
7.34
7.28
7.28
7.27
7.27
7.26 CDCl3
7.26
7.25
7.25
7.24
7.24
4.30
4.28
4.25
4.23
4.23
4.20
4.18
4.17
4.14
4.12
2.81
2.78
2.49
2.47
2.46
2.46
2.28
2.24
2.24
2.21
1.76
1.75
1.75
1.73
1.72
1.71
1.70
1.57
1.55
1.41
1.41
1.40
1.35
1.34
1.32
0.96
0.95
F
Me
NBoc
23 1H NMR, CDCl3
2.00
2.03
3.00
1.01
1.00
1.05
2.02
9.02
3.69
1.04
0.98
11.5 11.0 10.5 10.0 9.5 9.0 8.5 8.0 7.5 7.0 6.5 6.0 5.5 5.0 4.5 4.0 3.5 3.0 2.5 2.0 1.5 1.0 0.5 0.0 -0.5 -1.0
f1 (ppm)

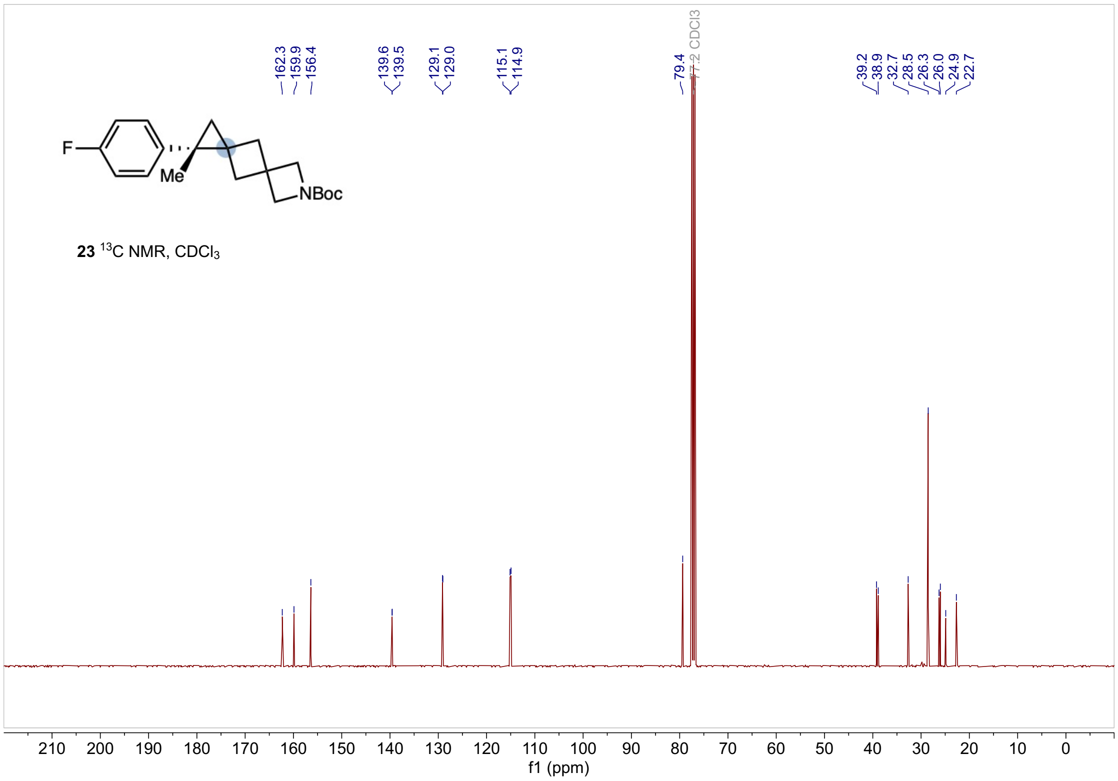

162.3
159.9
156.4
139.6
139.5
129.1
129.0
115.1
114.9
79.4
77.2 CDCl3
39.2
38.9
32.7
28.5
26.3
26.0
24.9
22.7
F
Me
NBoc
23 13C NMR, CDCl3
210 200 190 180 170 160 150 140 130 120 110 100 90 80 70 60 50 40 30 20 10 0
f1 (ppm)

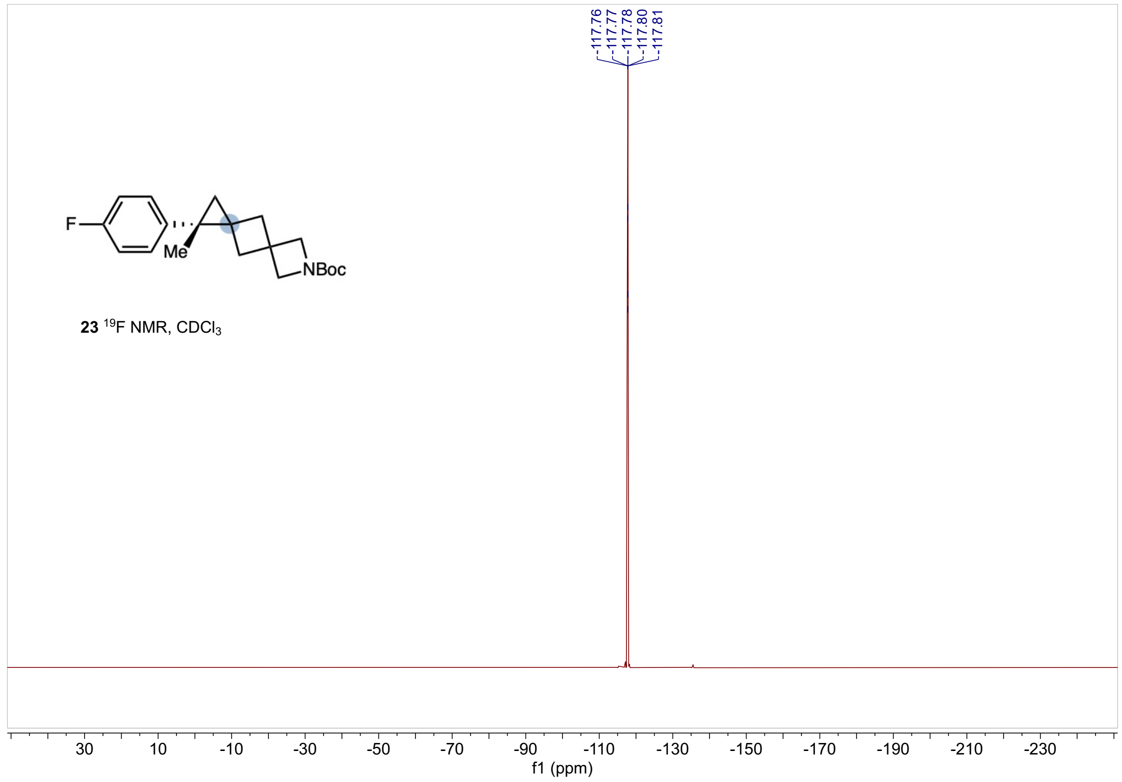

-117.76
-117.77
-117.78
-117.80
-117.81
F
Me
NBoc
23 19F NMR, CDCl3
30
10
-10
-30
-50
-70
-90
-110
-130
-150
-170
-190
-210
-230
f1 (ppm)

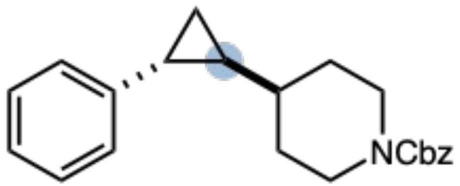
NCbz

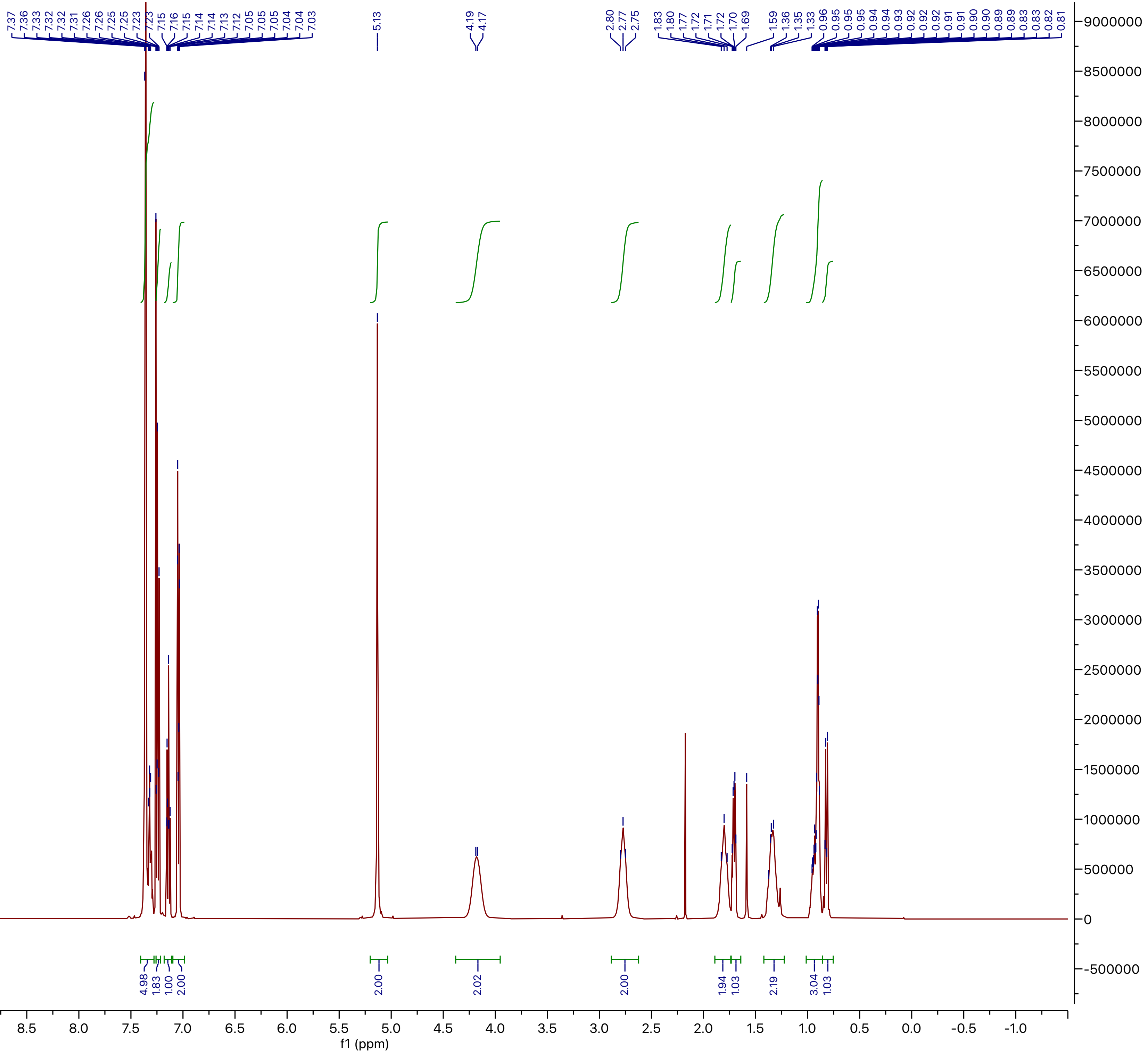
7.37
7.36
7.33
7.32
7.32
7.31
7.26
7.26
7.25
7.25
7.23
7.23
7.16
7.15
7.15
7.14
7.14
7.13
7.12
7.05
7.05
7.05
7.04
7.04
7.03
5.13
4.19
4.17
2.80
2.77
2.75
1.83
1.80
1.77
1.72
1.71
1.72
1.70
1.69
1.59
1.36
1.35
1.33
0.96
0.95
0.95
0.94
0.94
0.93
0.92
0.92
0.91
0.91
0.90
0.90
0.89
0.89
0.83
0.83
0.82
0.81
4.98
1.83
1.00
2.00
2.00
2.02
2.00
1.94
1.03
2.19
3.04
1.03
f1 (ppm)

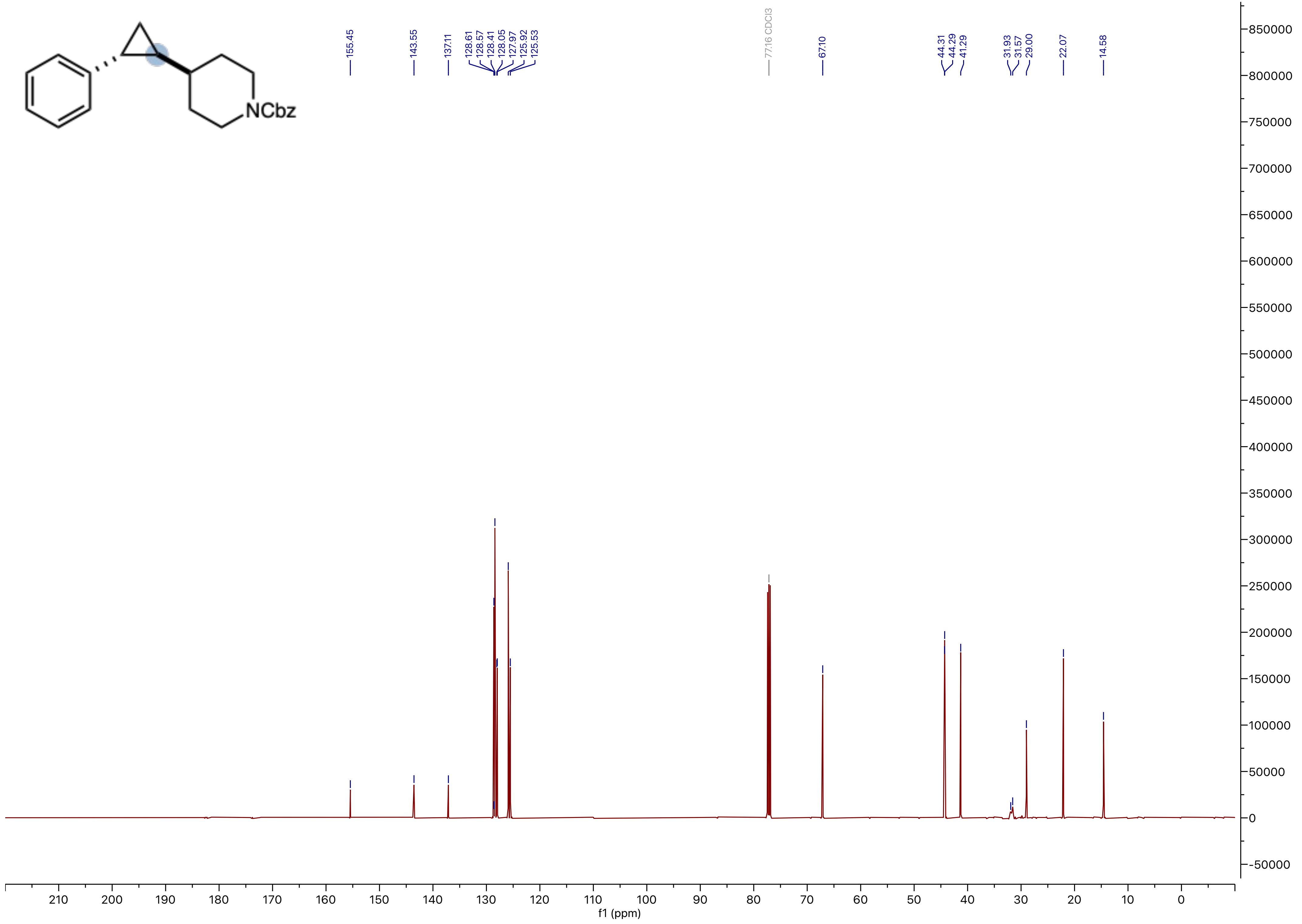
NCbz
155.45
143.55
137.11
128.61
128.57
128.41
128.05
127.97
125.92
125.53
77.16 CDCl3
67.10
44.31
44.29
41.29
31.93
31.57
29.00
22.07
14.58
f1 (ppm)

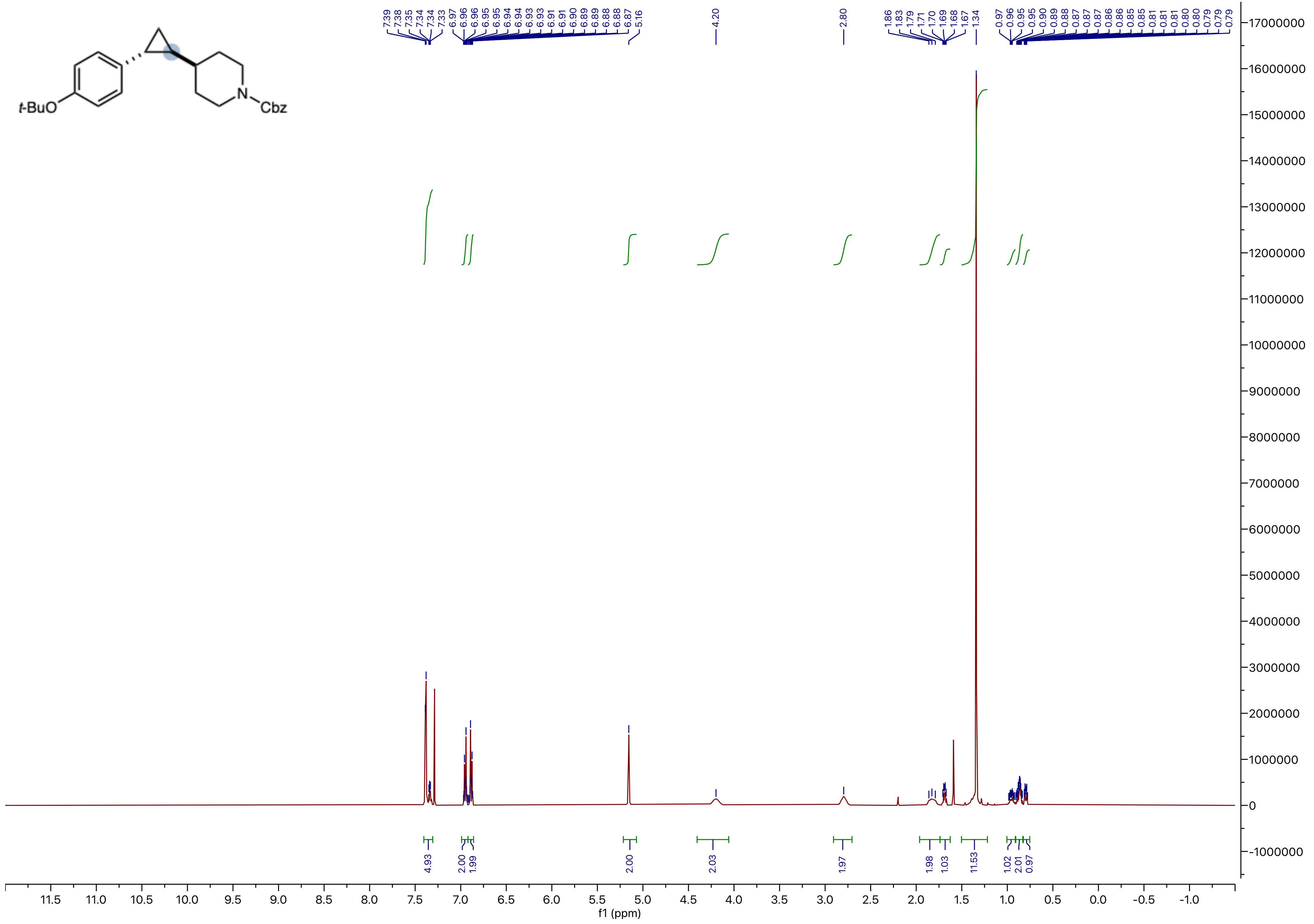

t-BuO
N
Cbz
7.39
7.38
7.35
7.34
7.34
7.33
6.97
6.96
6.96
6.95
6.95
6.94
6.93
6.93
6.91
6.91
6.90
6.89
6.89
6.88
6.88
6.87
5.16
4.20
2.80
1.86
1.83
1.79
1.71
1.70
1.69
1.68
1.67
1.34
0.97
0.96
0.95
0.95
0.90
0.89
0.88
0.87
0.87
0.86
0.86
0.85
0.81
0.81
0.80
0.80
0.79
0.79
0.79
4.93
2.00
1.99
2.00
2.03
1.97
1.98
1.03
11.53
1.02
2.01
0.97
f1 (ppm)

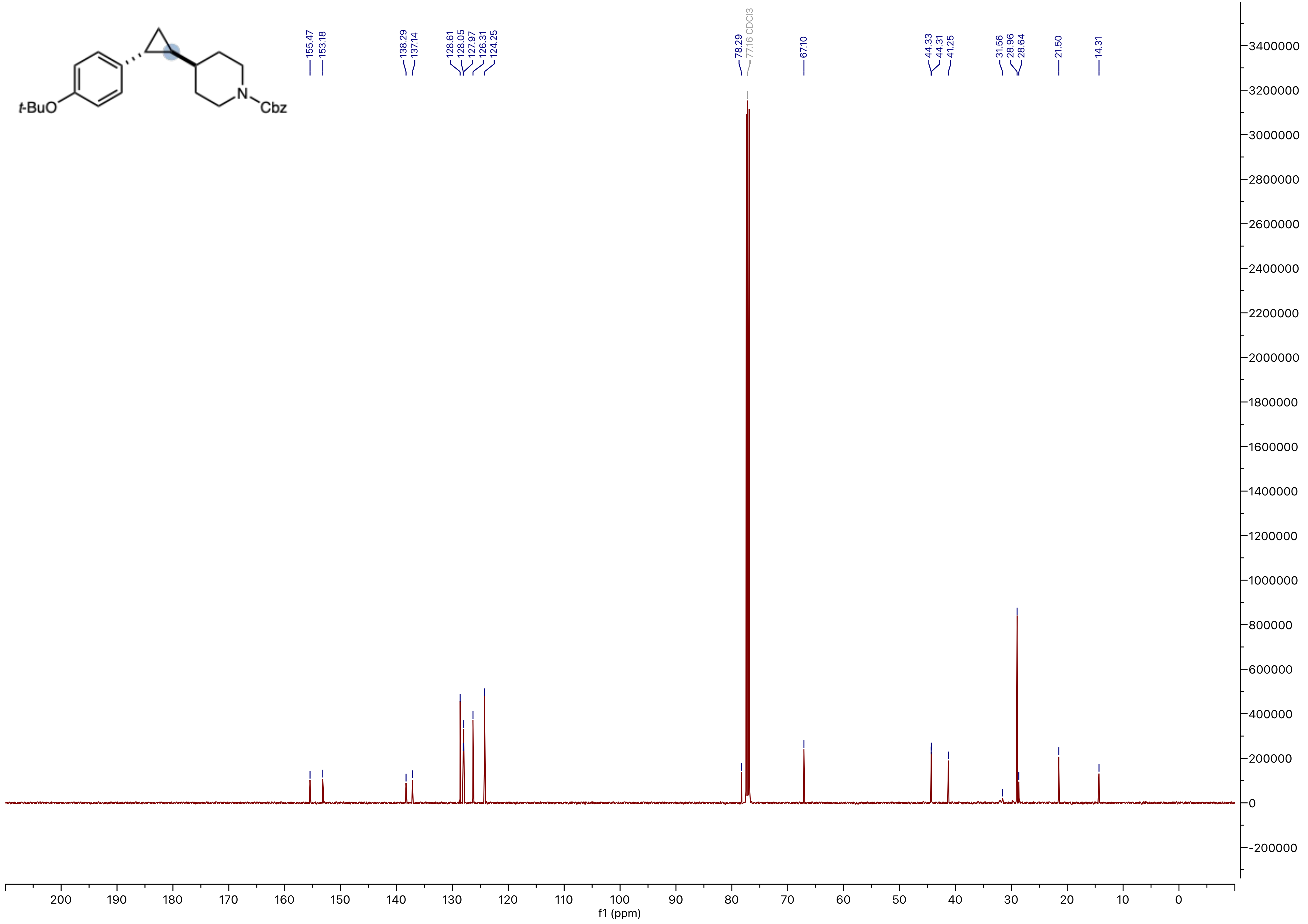
t-BuO
N
Cbz
155.47
153.18
138.29
137.14
128.61
128.05
127.97
126.31
124.25
78.29
77.16 CDCl3
67.10
44.33
44.31
41.25
31.56
28.96
28.64
21.50
14.31
f1 (ppm)

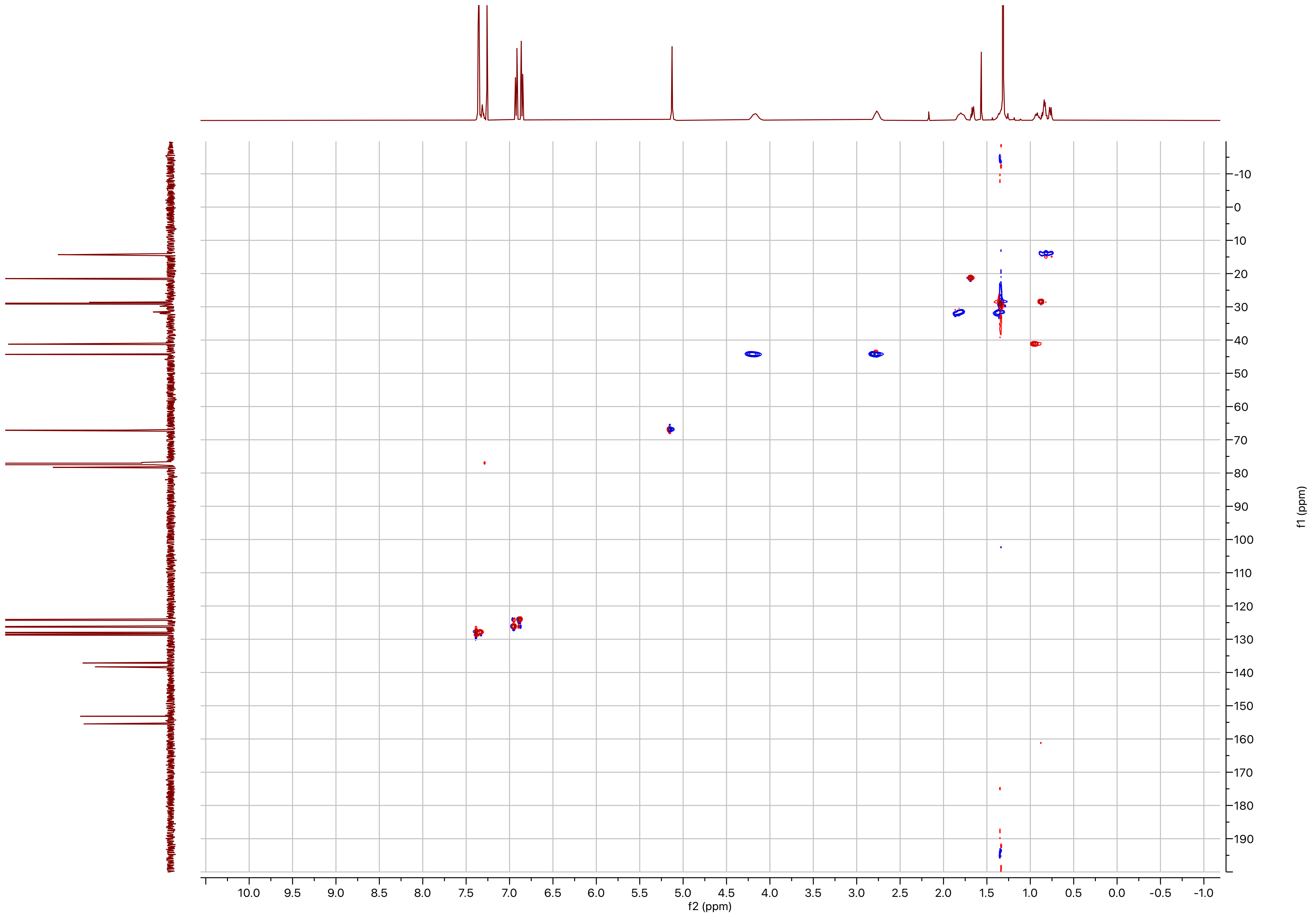
f2 (ppm)
f1 (ppm)

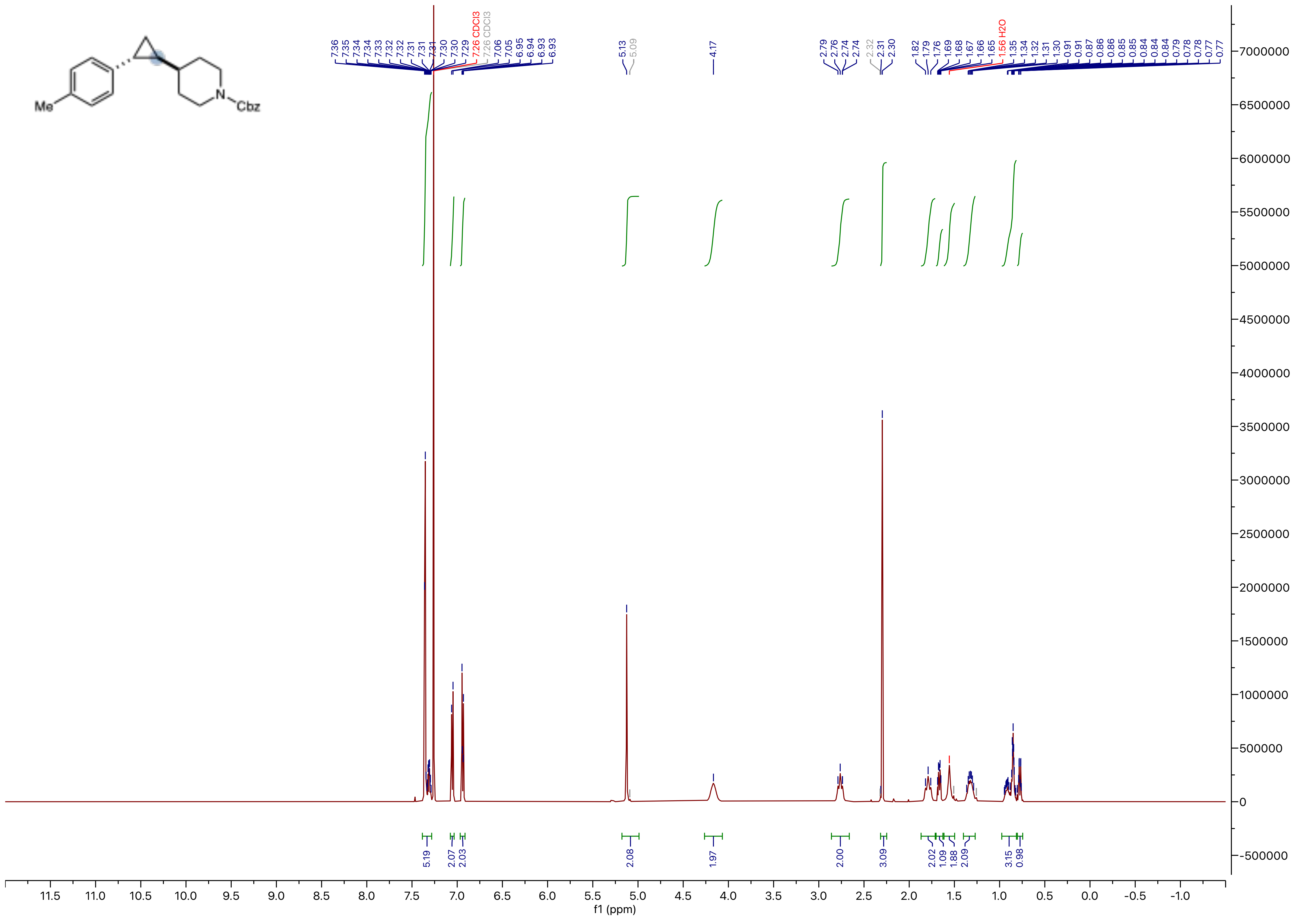

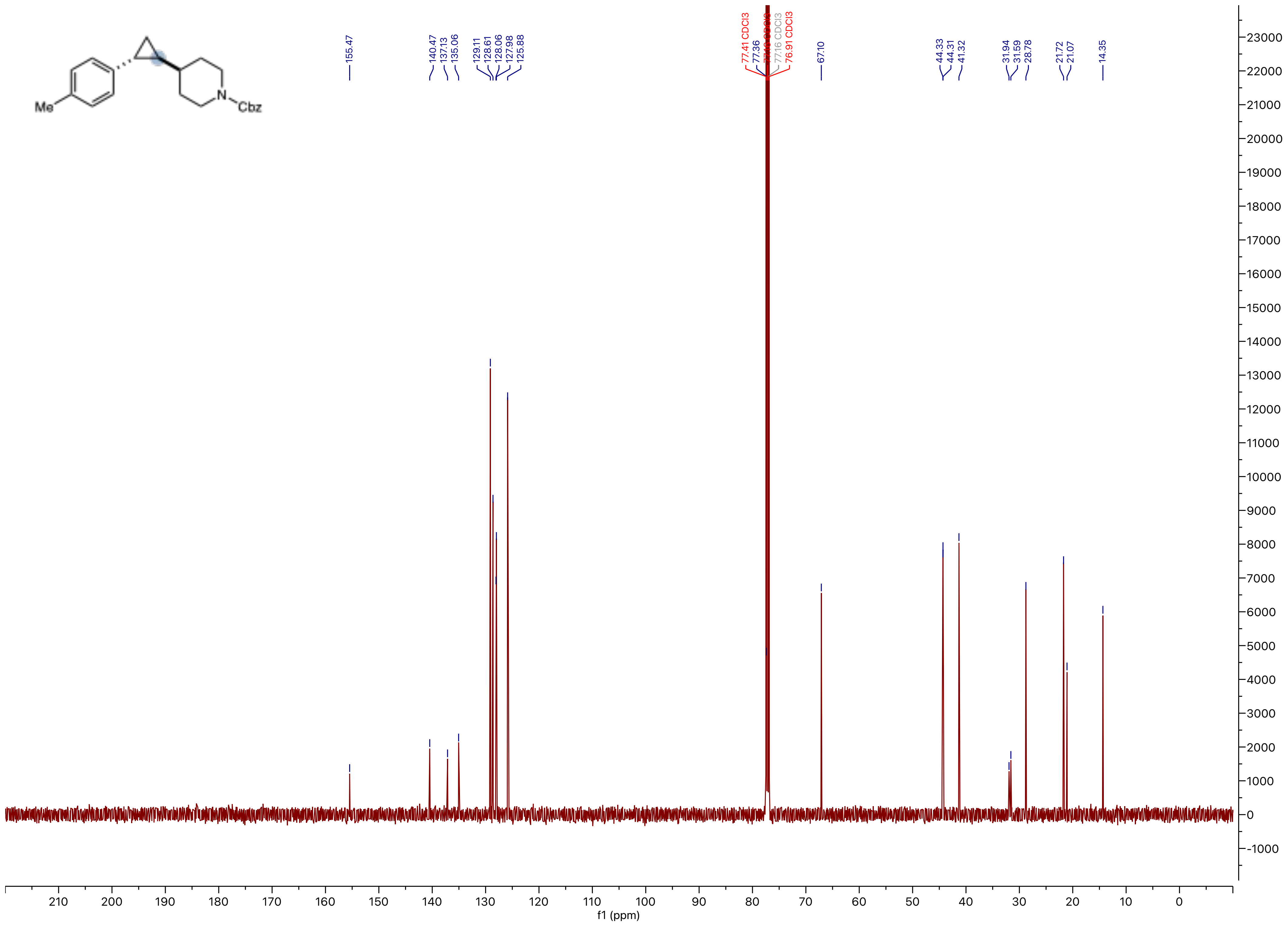

Me
N
Cbz
155.47
140.47
137.13
135.06
129.11
128.61
128.06
127.98
125.88
77.41 CDCl3
77.36
77.16 CDCl3
76.91 CDCl3
67.10
44.33
44.31
41.32
31.94
31.59
28.78
21.72
21.07
14.35
f1 (ppm)

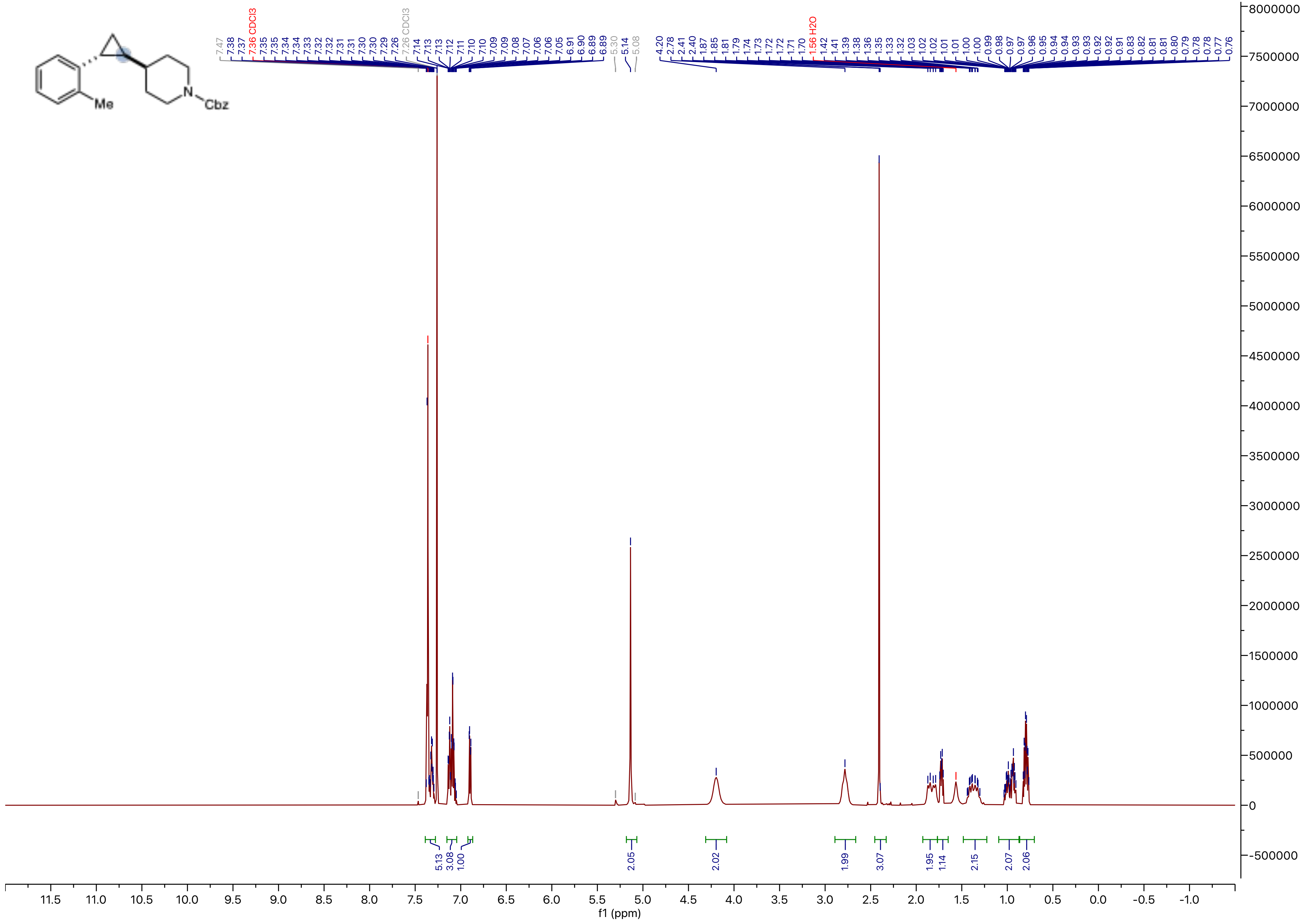

Me
N
Cbz
7.47
7.38
7.37
7.36 CDCl3
7.26 CDCl3
5.30
5.14
5.08
4.20
2.78
2.41
2.40
1.56 H2O
f1 (ppm)
5.13
3.08
1.00
2.05
2.02
1.99
3.07
1.95
1.14
2.15
2.07
2.06

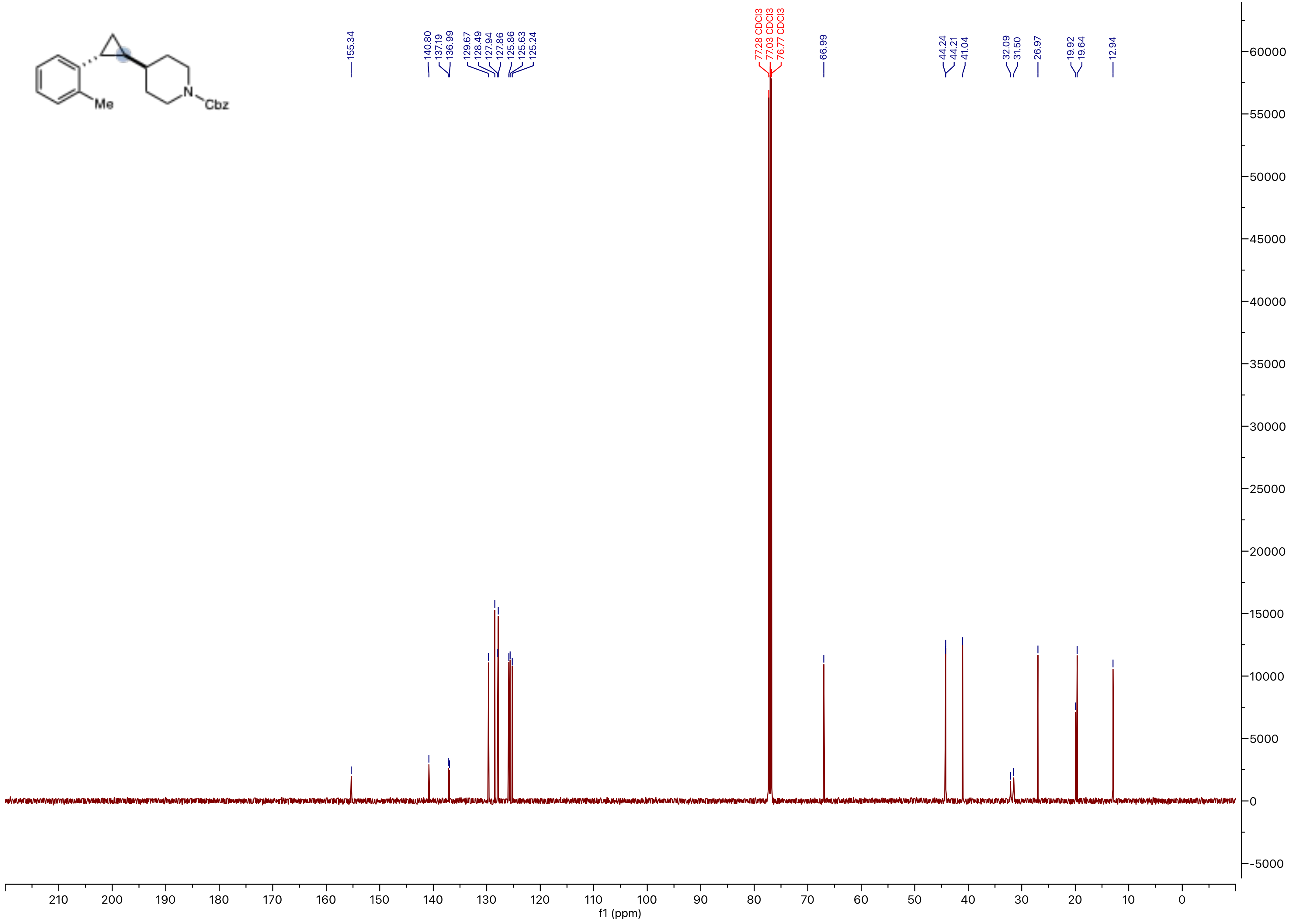

Me
N
Cbz
155.34
140.80
137.19
136.99
129.67
128.49
127.94
127.86
125.86
125.63
125.24
77.28 CDCl3
77.03 CDCl3
76.77 CDCl3
66.99
44.24
44.21
41.04
32.09
31.50
26.97
19.92
19.64
12.94
f1 (ppm)

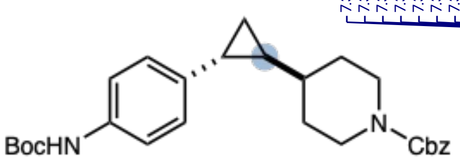
BocHN
N
Cbz

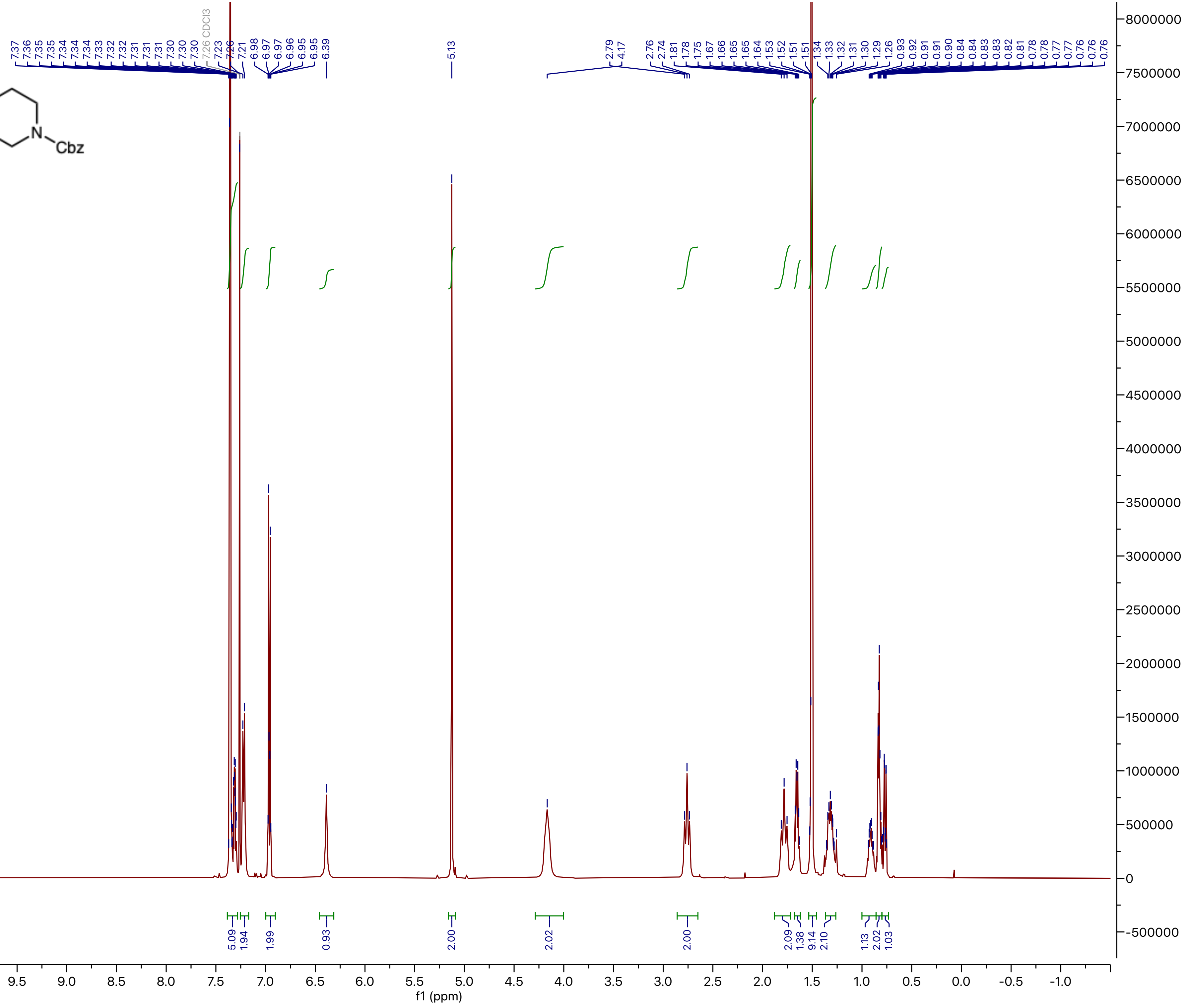
7.37
7.36
7.35
7.35
7.34
7.34
7.33
7.32
7.32
7.31
7.31
7.30
7.30
7.30
7.26 CDCl3
7.23
7.26
7.21
6.98
6.97
6.97
6.96
6.95
6.95
6.39
5.13
2.79
4.17
2.76
2.74
1.81
1.78
1.75
1.67
1.66
1.65
1.65
1.64
1.53
1.52
1.51
1.51
1.34
1.33
1.32
1.31
1.30
1.29
1.26
0.93
0.92
0.91
0.91
0.90
0.84
0.84
0.83
0.83
0.82
0.81
0.78
0.78
0.77
0.77
0.76
0.76
0.76
5.09
1.94
1.99
0.93
2.00
2.02
2.00
2.09
1.38
9.14
2.10
1.13
2.02
1.03
f1 (ppm)

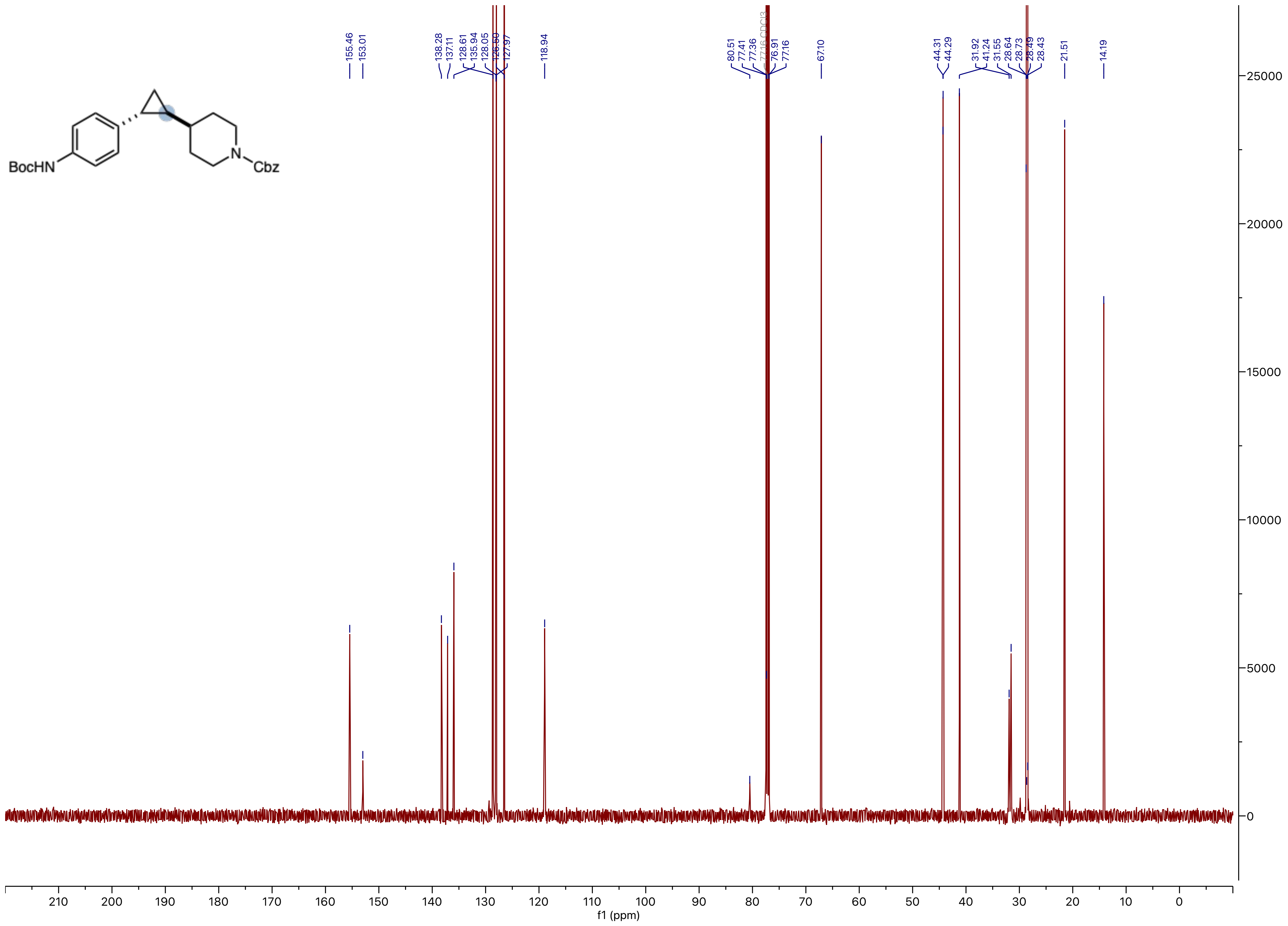

BocHN
N
Cbz
155.46
153.01
138.28
137.11
128.61
135.94
128.05
126.50
127.97
118.94
80.51
77.41
77.36
77.16 CDCl3
76.91
77.16
67.10
44.31
44.29
31.92
41.24
31.55
28.64
28.73
28.49
28.43
21.51
14.19
25000
20000
15000
10000
5000
0
210
200
190
180
170
160
150
140
130
120
110
100
90
80
70
60
50
40
30
20
10
0
f1 (ppm)

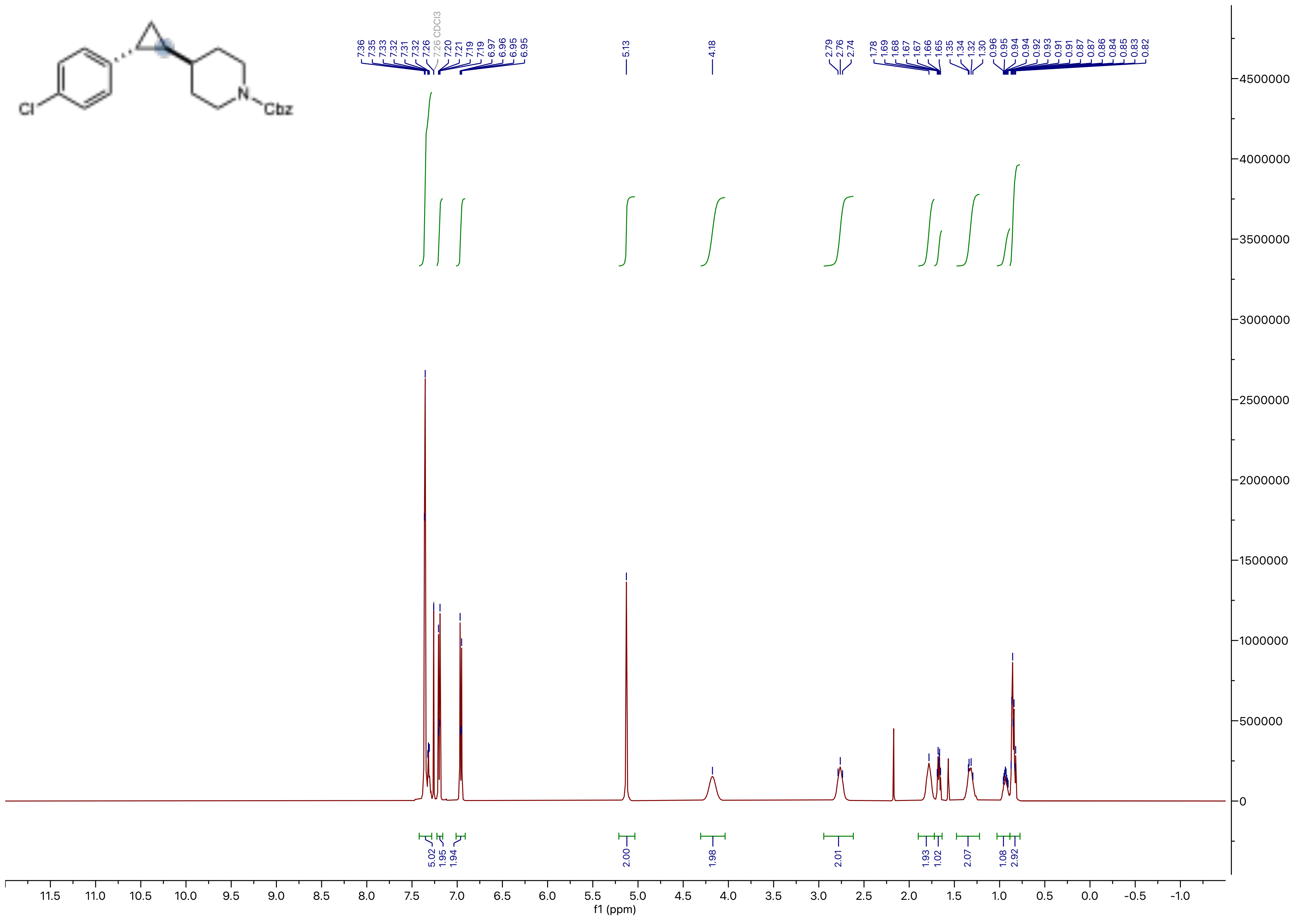
Cl
N
Cbz
7.36
7.35
7.33
7.32
7.31
7.32
7.26
7.26 CDCl3
7.20
7.21
7.19
7.19
6.97
6.96
6.95
6.95
5.13
4.18
2.79
2.76
2.74
1.78
1.69
1.68
1.67
1.67
1.66
1.65
1.35
1.34
1.32
1.30
0.96
0.95
0.94
0.94
0.92
0.93
0.91
0.91
0.87
0.86
0.84
0.85
0.83
0.82
5.02
1.95
1.94
2.00
1.98
2.01
1.93
1.02
2.07
1.08
2.92
f1 (ppm)

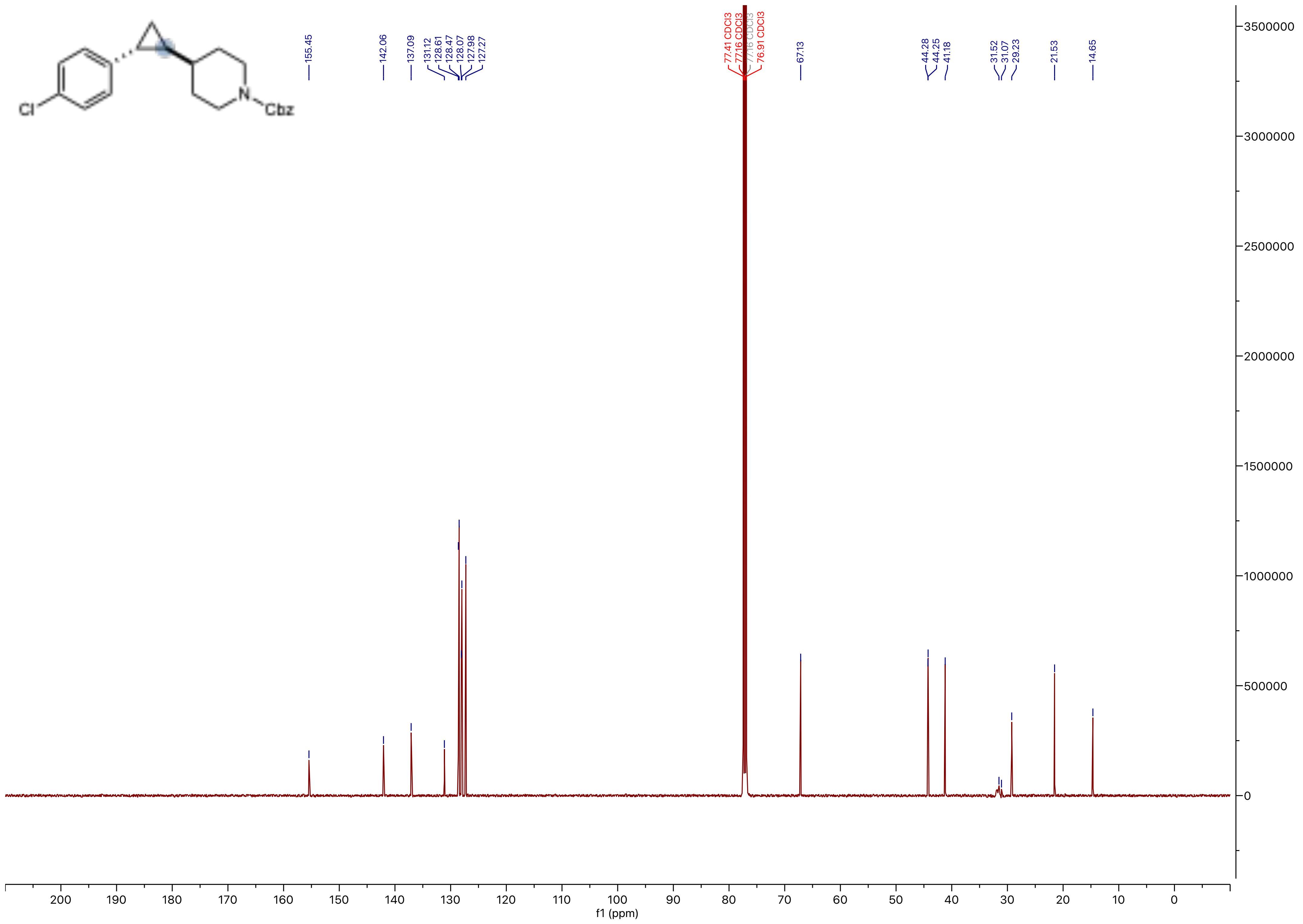

Cl
N
Cbz
155.45
142.06
137.09
131.12
128.61
128.47
128.07
127.98
127.27
77.41 CDCl3
77.16 CDCl3
77.16 CDCl3
76.91 CDCl3
67.13
44.28
44.25
41.18
31.52
31.07
29.23
21.53
14.65
3500000
3000000
2500000
2000000
1500000
1000000
500000
0
200 190 180 170 160 150 140 130 120 110 100 90 80 70 60 50 40 30 20 10 0
f1 (ppm)

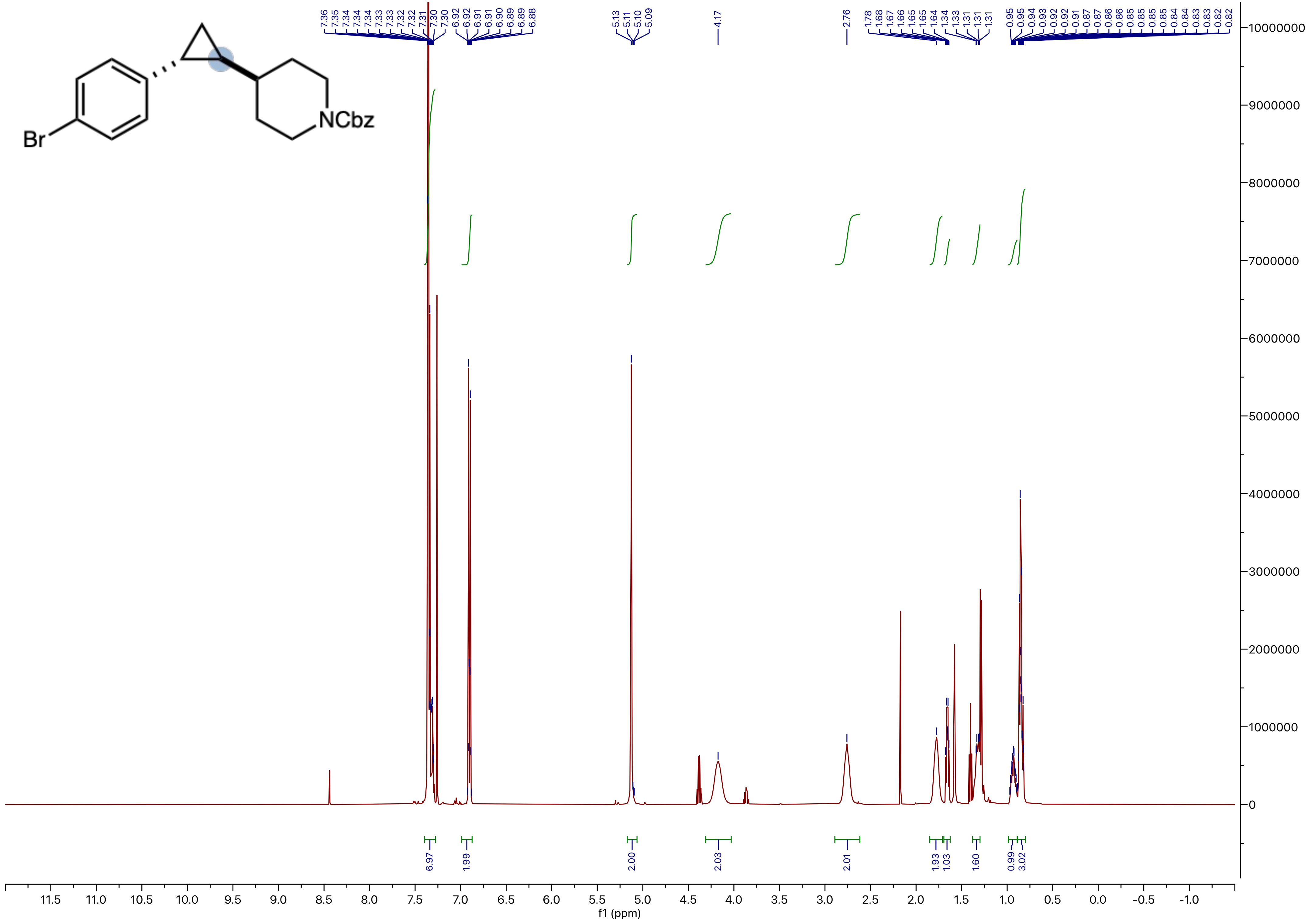

Br
NCbz
7.36 7.35 7.34 7.34 7.33 7.33 7.32 7.32 7.31 7.30 7.30 6.92 6.92 6.91 6.91 6.90 6.89 6.89 6.88
5.13 5.11 5.10 5.09
4.17
2.76
1.78 1.68 1.67 1.66 1.65 1.65 1.64 1.34 1.33 1.31 1.31 1.31
0.95 0.95 0.94 0.93 0.92 0.92 0.91 0.87 0.87 0.86 0.86 0.85 0.85 0.85 0.85 0.84 0.84 0.83 0.83 0.82 0.82
6.97 1.99 2.00 2.03 2.01 1.93 1.03 1.60 0.99 3.02
f1 (ppm)

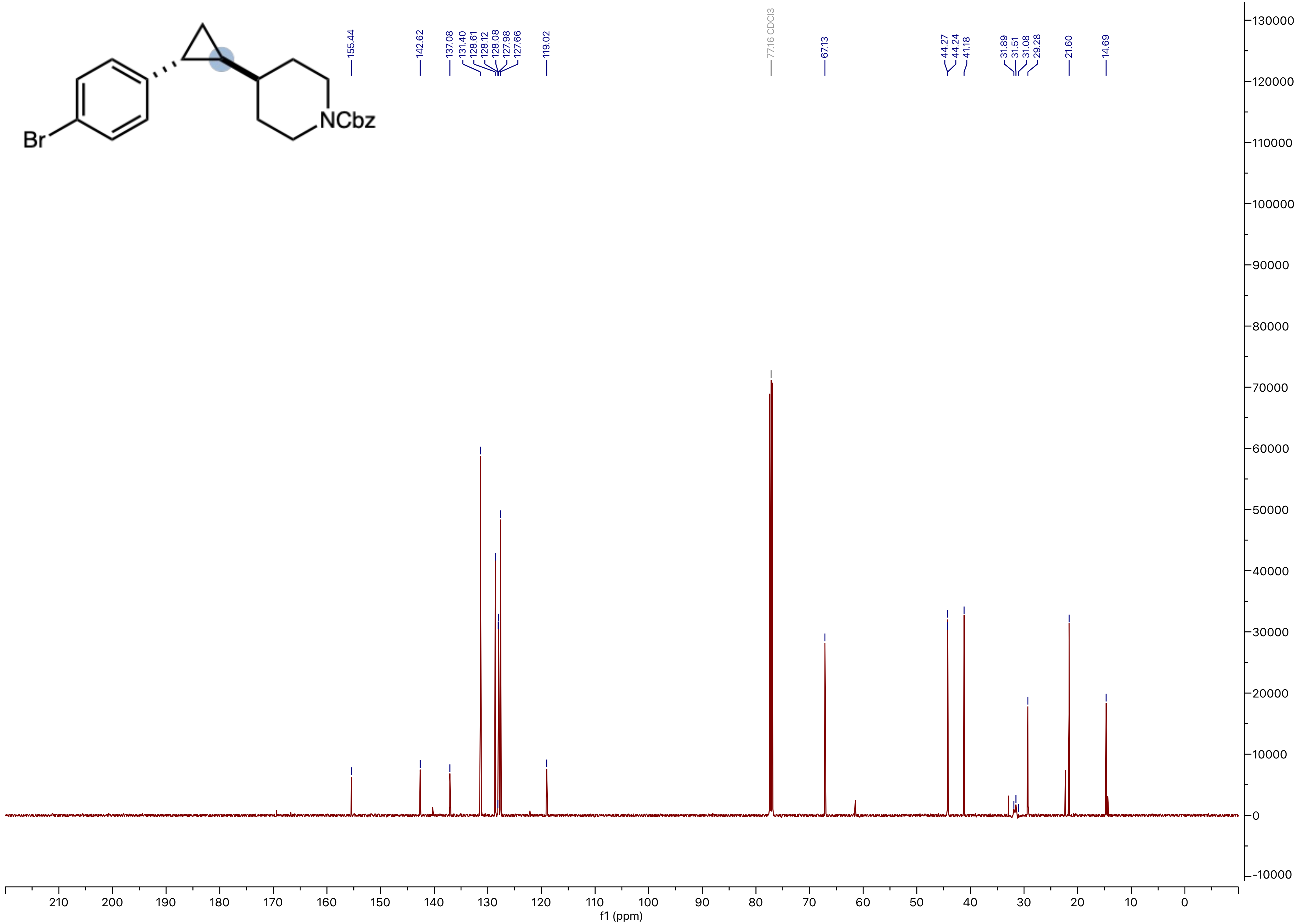

Br
NCbz
155.44
142.62
137.08
131.40
128.61
128.12
128.08
127.98
127.66
119.02
77.16 CDCl3
67.13
44.27
44.24
41.18
31.89
31.51
31.08
29.28
21.60
14.69
f1 (ppm)

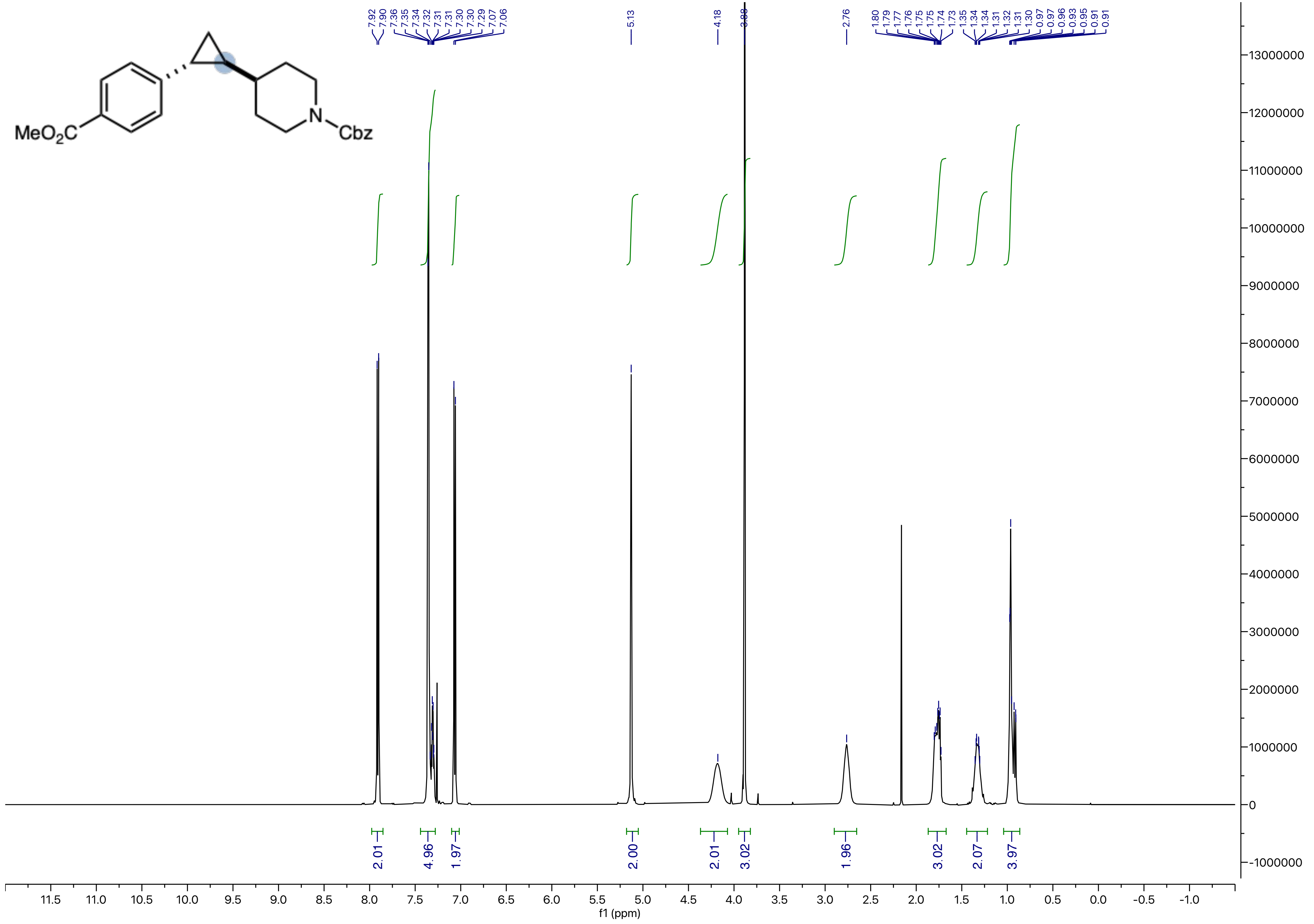

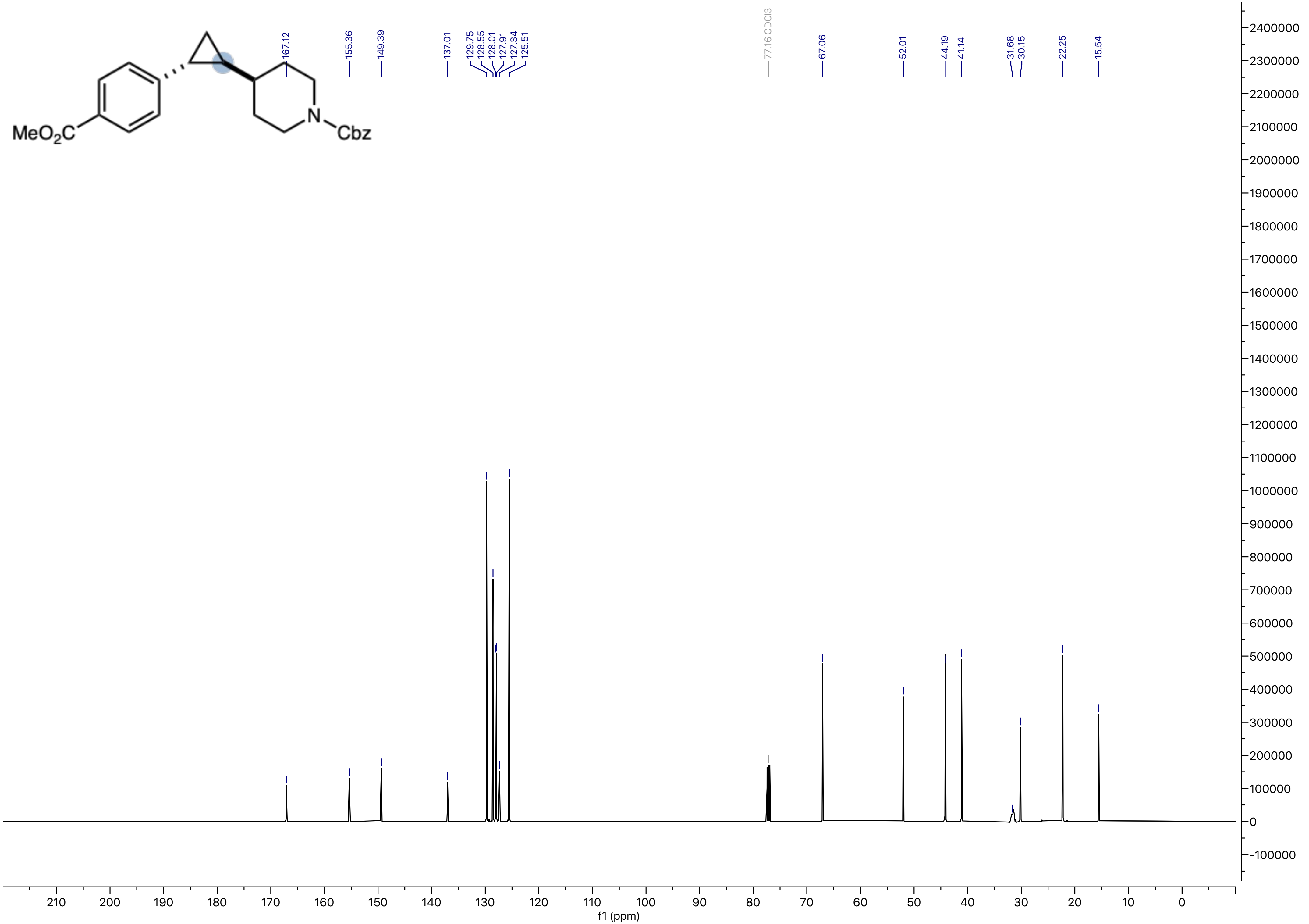

167.12
155.36
149.39
137.01
129.75
128.55
128.01
127.91
127.34
125.51
77.16 CDCl3
67.06
52.01
44.19
41.14
31.68
30.15
22.25
15.54
2400000
2300000
2200000
2100000
2000000
1900000
1800000
1700000
1600000
1500000
1400000
1300000
1200000
1100000
1000000
900000
800000
700000
600000
500000
400000
300000
200000
100000
0
-100000
210 200 190 180 170 160 150 140 130 120 110 100 90 80 70 60 50 40 30 20 10 0
f1 (ppm)

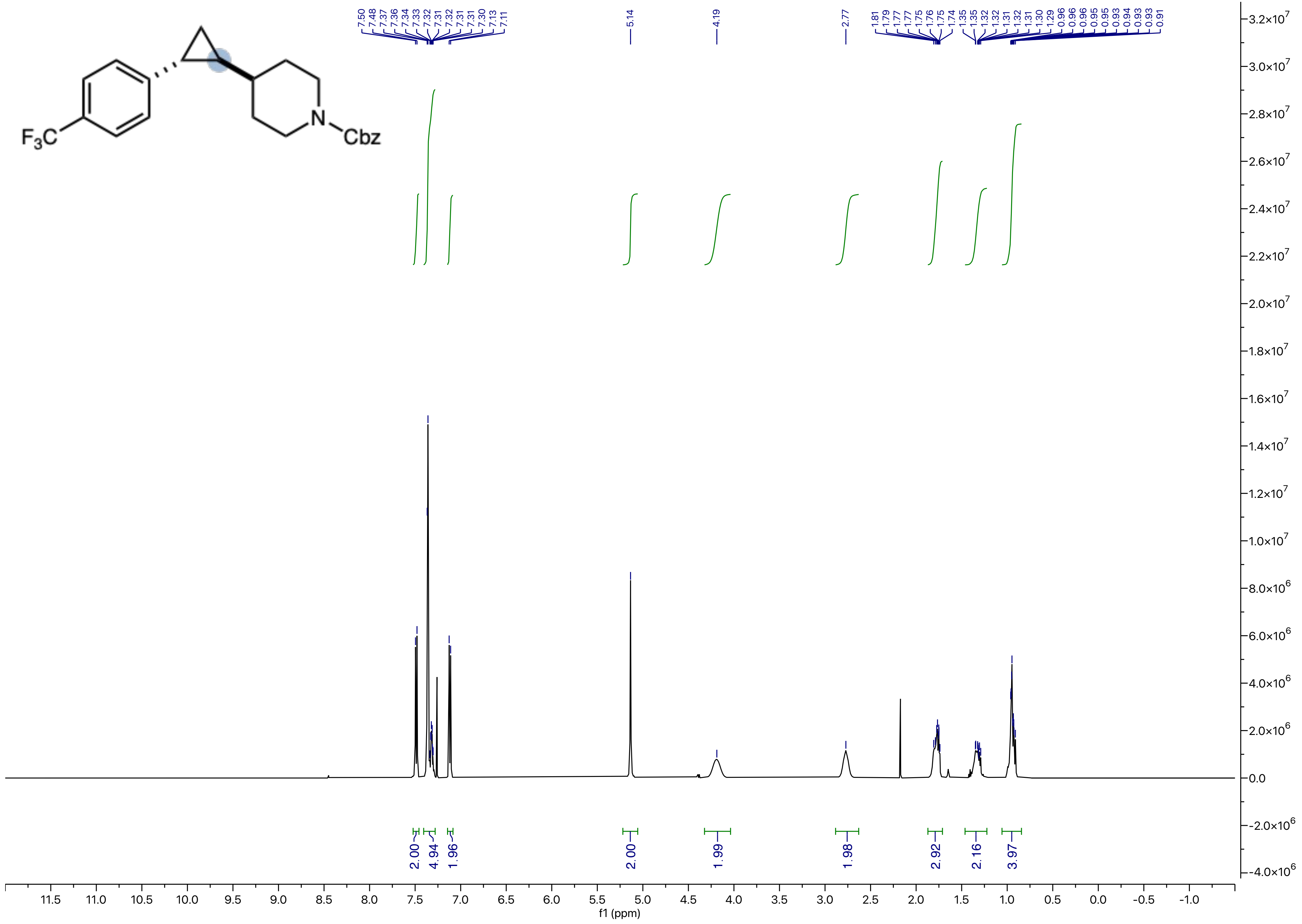

F3C
N
Cbz
7.50 7.48 7.37 7.36 7.34 7.33 7.32 7.31 7.32 7.31 7.31 7.30 7.13 7.11
5.14
4.19
2.77
1.81 1.79 1.77 1.77 1.75 1.76 1.75 1.74 1.35 1.35 1.32 1.32 1.31 1.32 1.31 1.30 1.29 0.96 0.96 0.95 0.95 0.93 0.94 0.93 0.93 0.91
2.00 4.94 1.96 2.00 1.99 1.98 2.92 2.16 3.97
f1 (ppm)

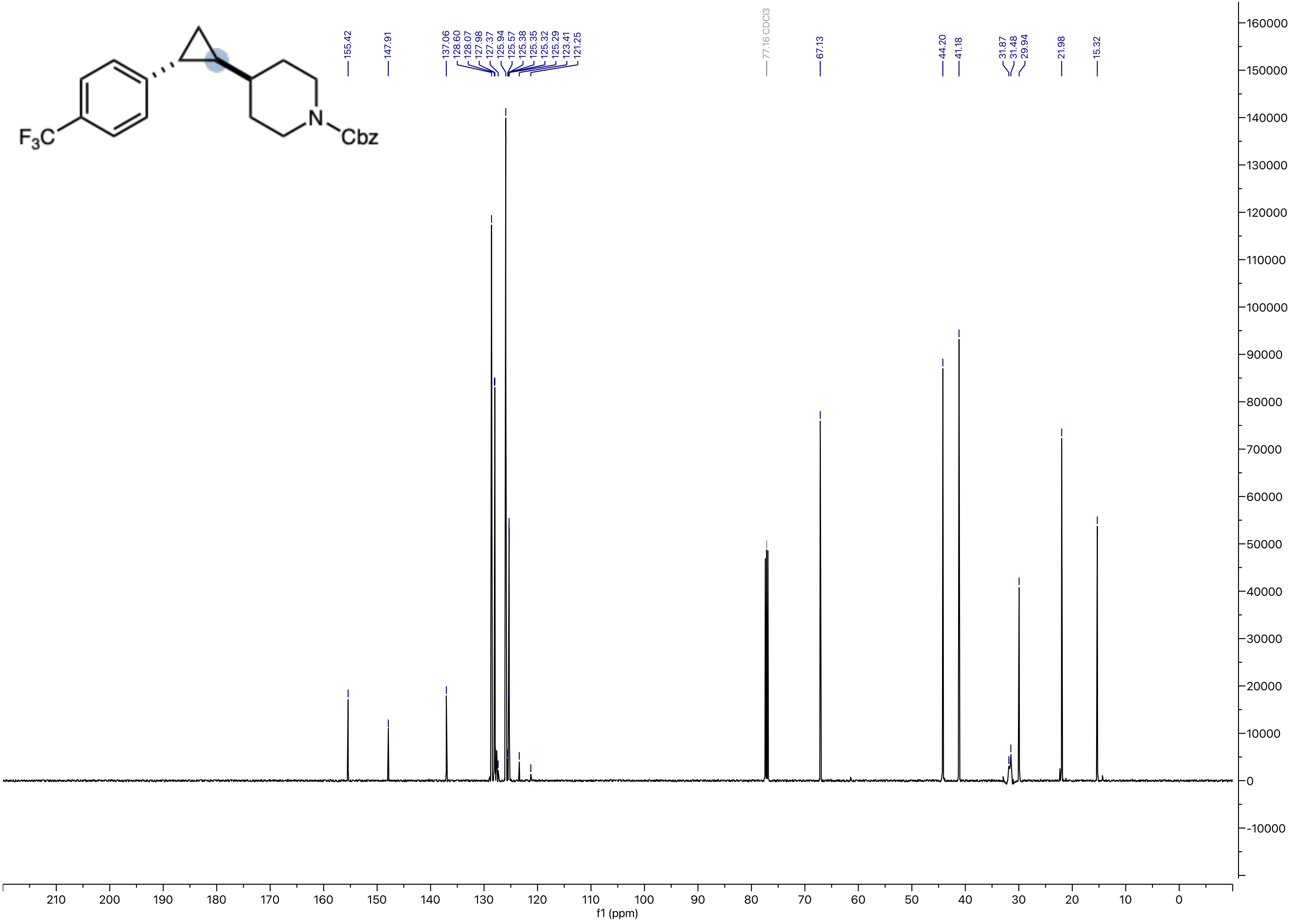

F3C
N
Cbz
155.42
147.91
137.06
128.60
128.07
127.98
127.37
125.94
125.57
125.38
125.35
125.32
125.29
123.41
121.25
77.16 CDCl3
67.13
44.20
41.18
31.87
31.48
29.94
21.98
15.32
f1 (ppm)

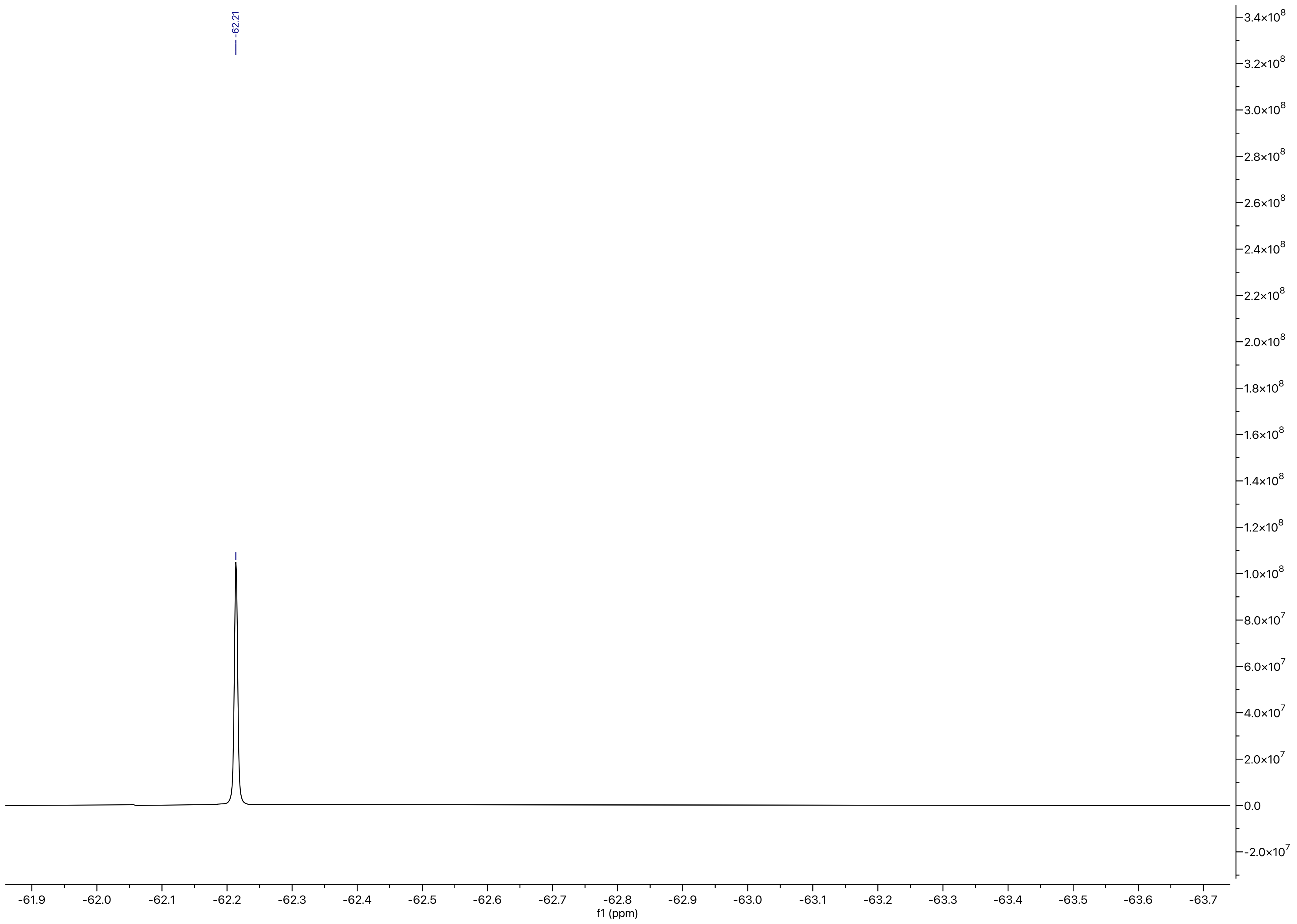

-62.21
f1 (ppm)

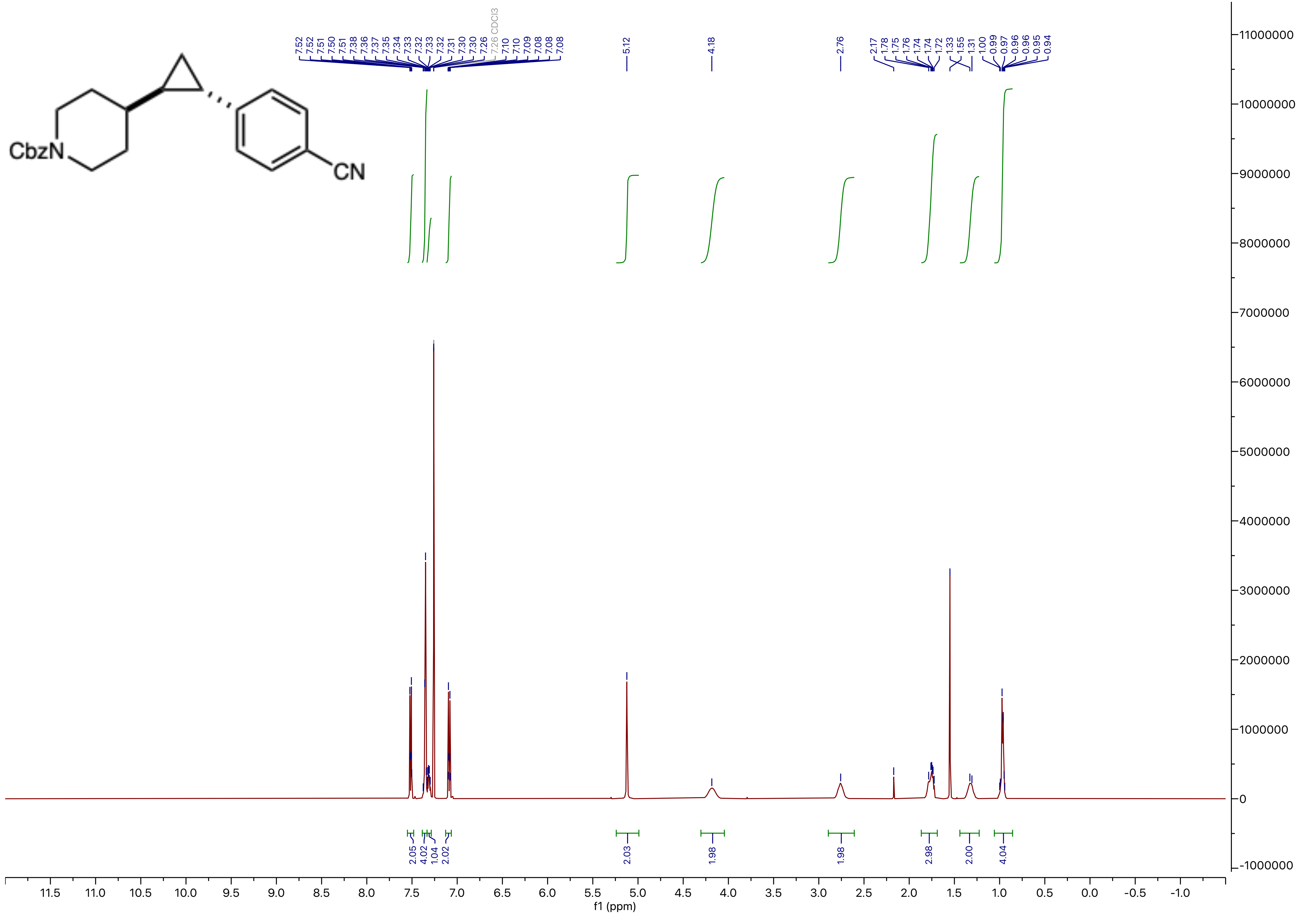

CbzN
CN
7.52
7.52
7.51
7.50
7.51
7.38
7.36
7.37
7.35
7.34
7.33
7.32
7.33
7.32
7.31
7.30
7.30
7.26 CDCl3
7.10
7.10
7.09
7.08
7.08
7.08
5.12
4.18
2.76
2.17
1.78
1.75
1.76
1.74
1.74
1.72
1.33
1.55
1.31
1.00
0.99
0.97
0.96
0.96
0.95
0.94
2.05
4.02
1.04
2.02
2.03
1.98
1.98
2.98
2.00
4.04
11.5
11.0
10.5
10.0
9.5
9.0
8.5
8.0
7.5
7.0
6.5
6.0
5.5
5.0
4.5
4.0
3.5
3.0
2.5
2.0
1.5
1.0
0.5
0.0
-0.5
-1.0
f1 (ppm)
11000000
10000000
9000000
8000000
7000000
6000000
5000000
4000000
3000000
2000000
1000000
0
-1000000

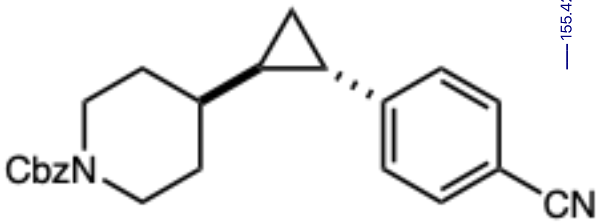
CbzN
CN

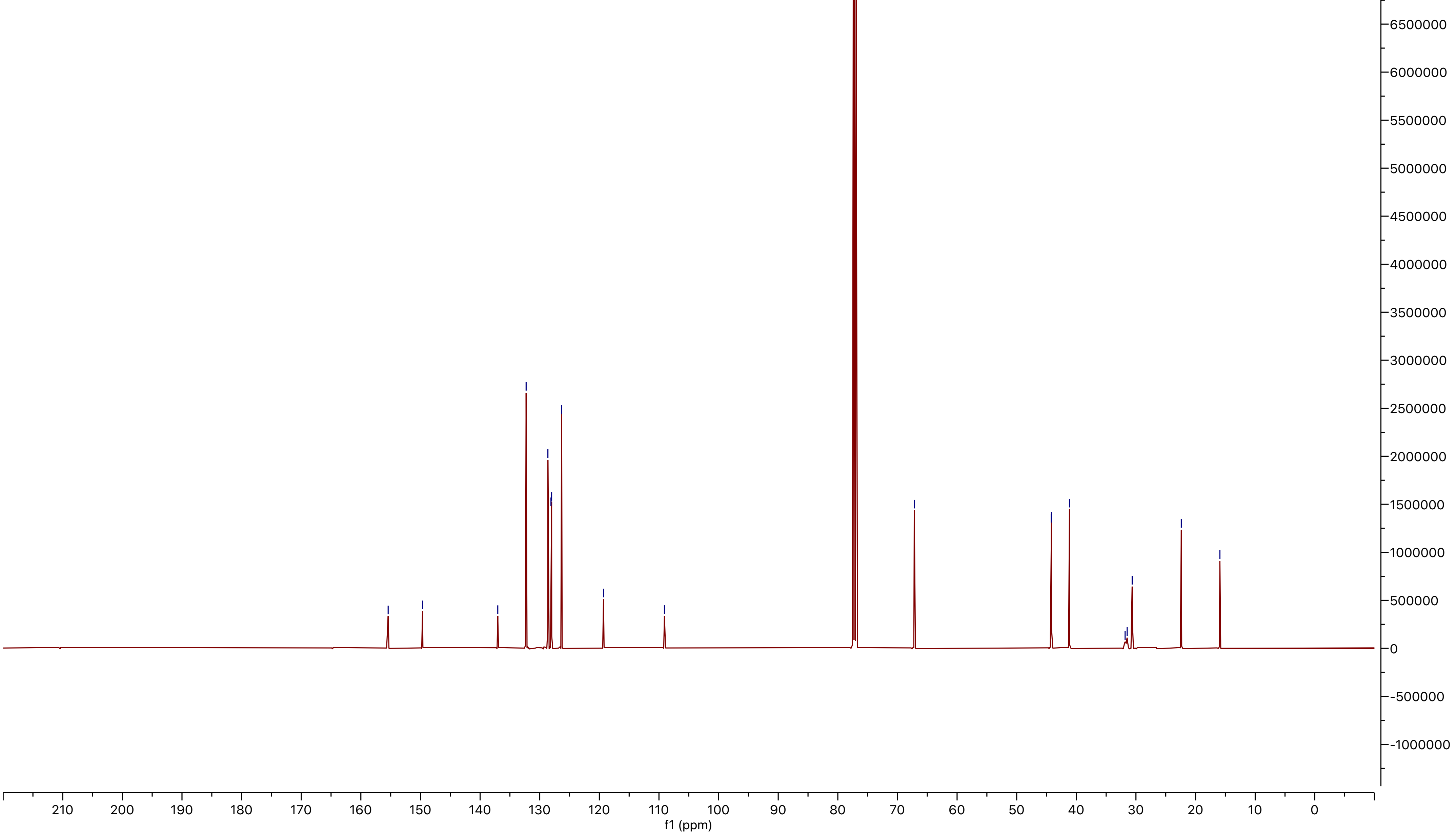
155.42
149.65
137.03
132.28
128.63
128.01
128.11
126.32
119.30
109.08
77.16 CDCl3
67.18
44.22
44.17
41.15
31.82
30.64
31.48
22.39
15.92
8500000
8000000
7500000
7000000
6500000
6000000
5500000
5000000
4500000
4000000
3500000
3000000
2500000
2000000
1500000
1000000
500000
0
-500000
-1000000
210 200 190 180 170 160 150 140 130 120 110 100 90 80 70 60 50 40 30 20 10 0
f1 (ppm)

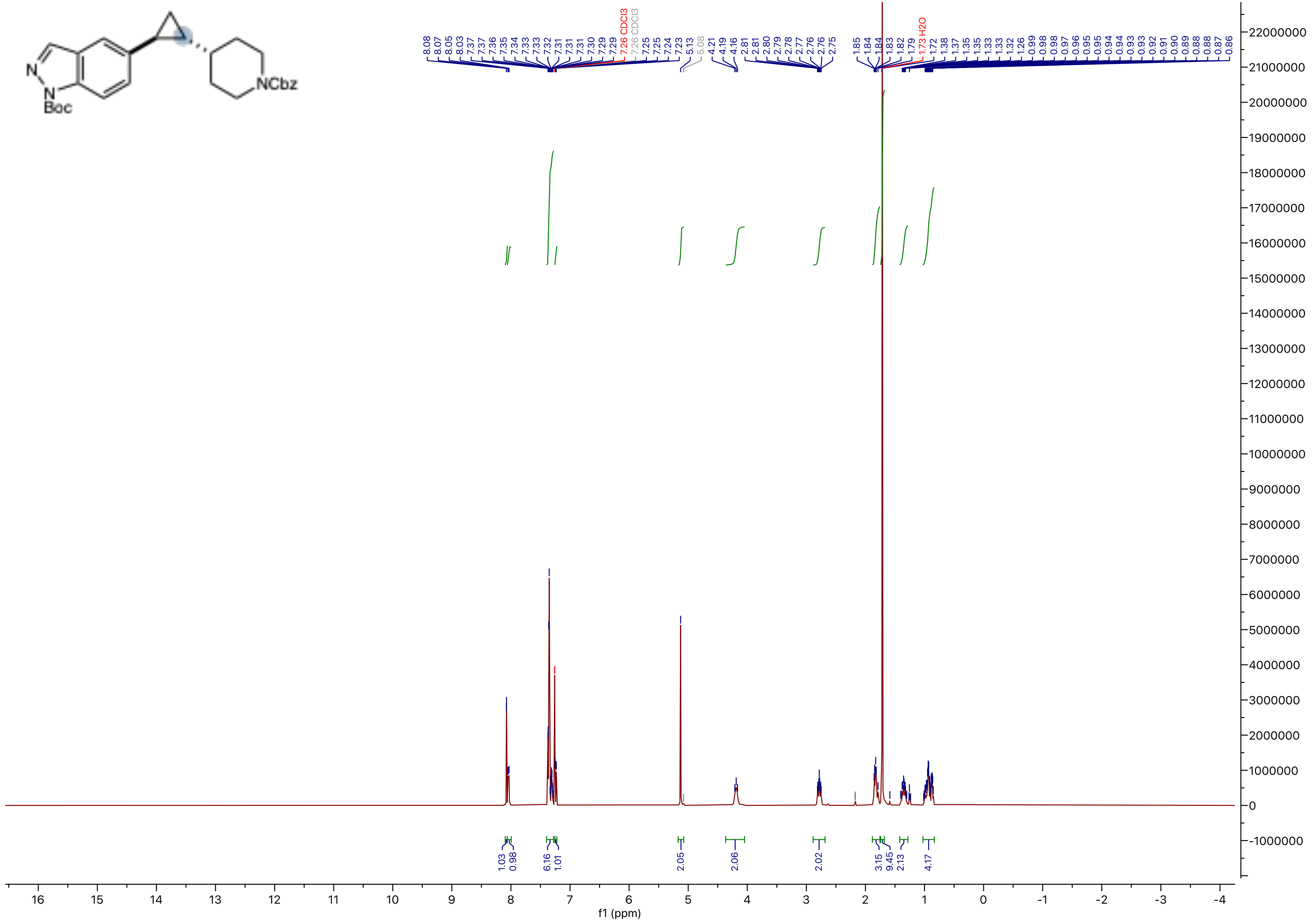

N
N
Boc
NCbz
8.08
8.07
8.05
8.03
7.37
7.37
7.36
7.35
7.34
7.33
7.33
7.32
7.31
7.31
7.31
7.30
7.29
7.29
7.26 CDCl3
7.26 CDCl3
7.25
7.25
7.24
7.23
5.13
5.08
4.21
4.19
4.16
2.81
2.80
2.79
2.78
2.77
2.76
2.76
2.75
1.85
1.84
1.84
1.83
1.82
1.79
1.73 H2O
1.72
1.38
1.37
1.35
1.35
1.33
1.33
1.32
1.26
0.99
0.98
0.98
0.97
0.96
0.95
0.95
0.94
0.94
0.93
0.93
0.92
0.91
0.90
0.89
0.88
0.88
0.87
0.86
1.03
0.98
6.16
1.01
2.05
2.06
2.02
3.15
9.45
2.13
4.17
f1 (ppm)

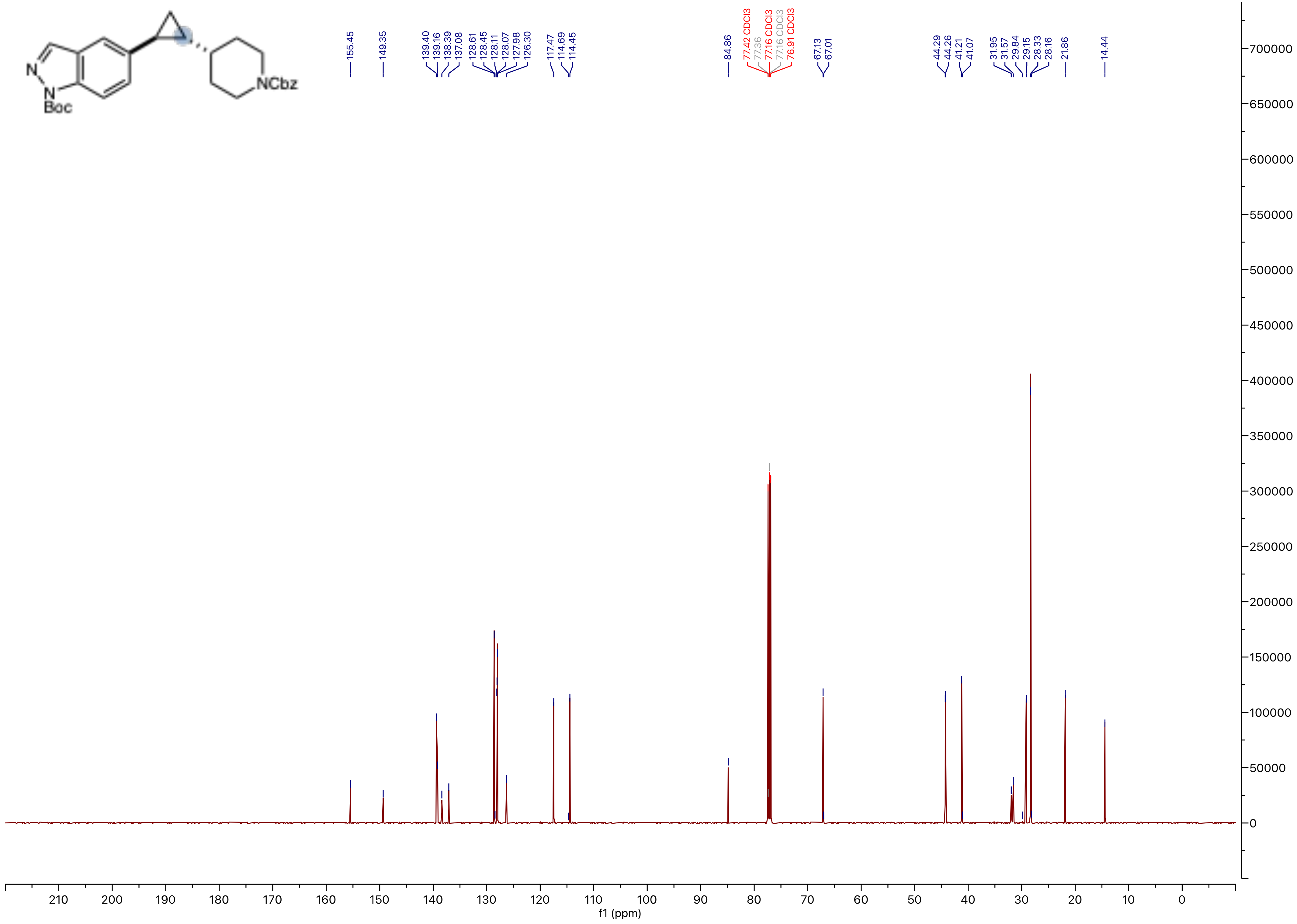

N
N
Boc
NCbz
155.45
149.35
139.40
139.16
138.39
137.08
128.61
128.45
128.11
128.07
127.98
126.30
117.47
114.69
114.45
84.86
77.42 CDCl3
77.36
77.16 CDCl3
77.16 CDCl3
76.91 CDCl3
67.13
67.01
44.29
44.26
41.21
41.07
31.95
31.57
29.84
29.15
28.33
28.16
21.86
14.44
f1 (ppm)

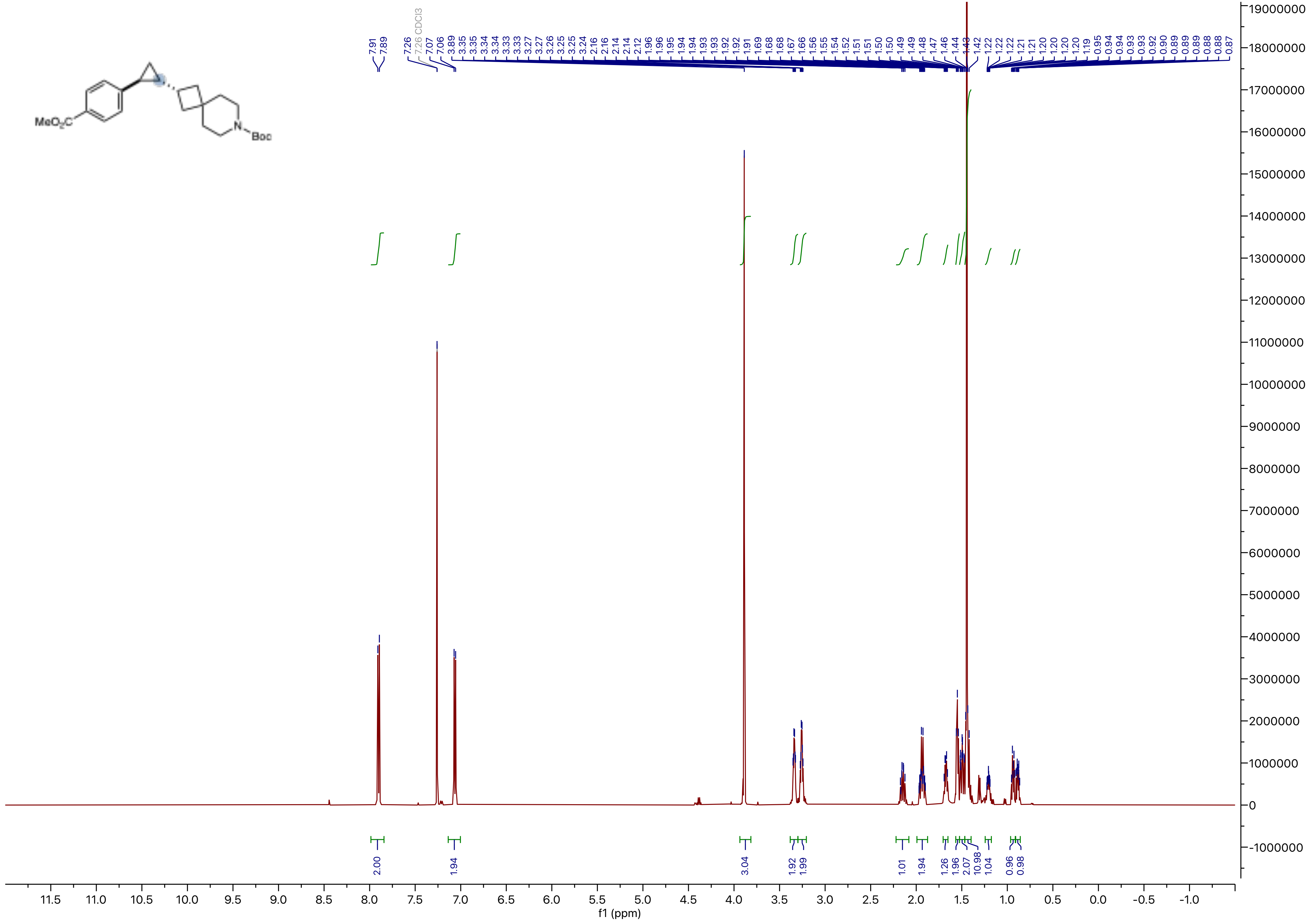
MeO2C
N
Boc
7.91
7.89
7.26
7.26 CDCl3
7.07
7.06
3.89
3.35
3.35
3.34
3.34
3.33
3.33
3.27
3.26
3.25
3.25
3.24
2.16
2.16
2.14
2.12
1.96
1.96
1.95
1.94
1.94
1.93
1.93
1.92
1.92
1.91
1.69
1.68
1.67
1.66
1.56
1.55
1.54
1.52
1.51
1.51
1.50
1.49
1.49
1.48
1.47
1.46
1.44
1.43
1.42
1.22
1.22
1.22
1.21
1.21
1.20
1.20
1.20
1.20
1.19
0.95
0.94
0.94
0.93
0.93
0.92
0.90
0.89
0.89
0.89
0.88
0.88
0.87
2.00
1.94
3.04
1.92
1.99
1.01
1.94
1.26
1.96
2.07
10.98
1.04
0.96
0.98
f1 (ppm)

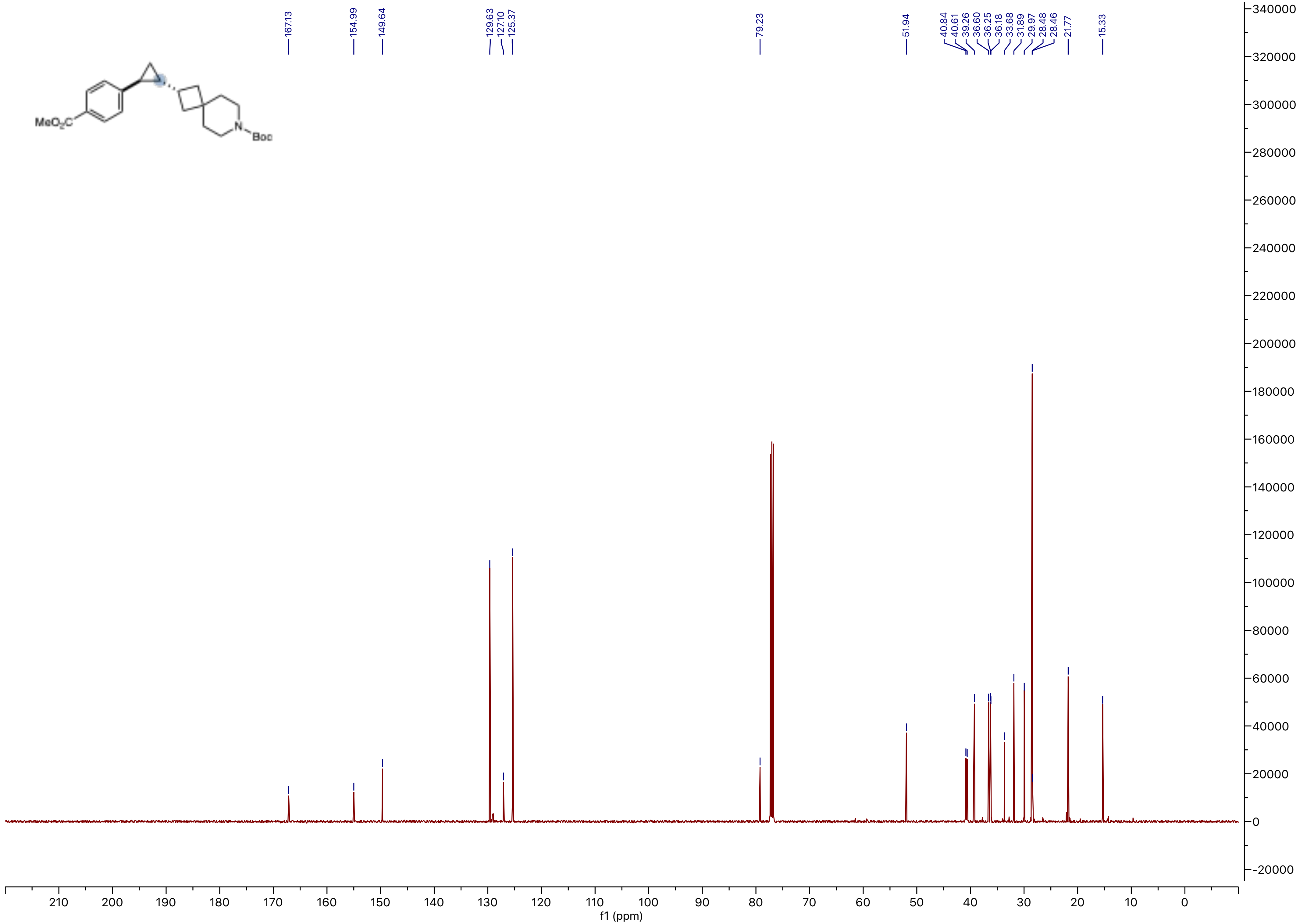

MeO2C
N
Boc
167.13
154.99
149.64
129.63
127.10
125.37
79.23
51.94
40.84
40.61
39.26
36.60
36.25
36.18
33.68
31.89
29.97
28.48
28.46
21.77
15.33
f1 (ppm)

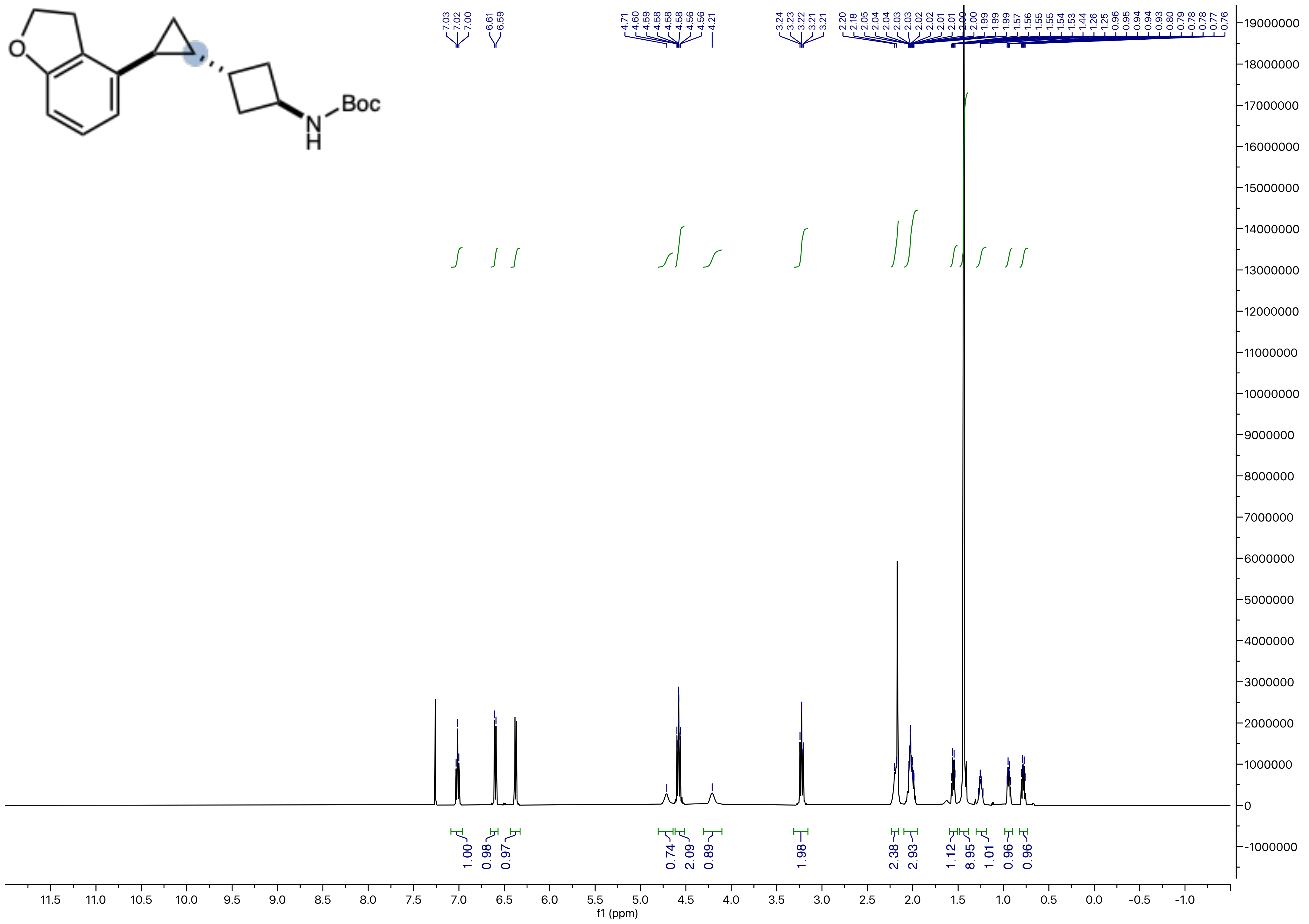

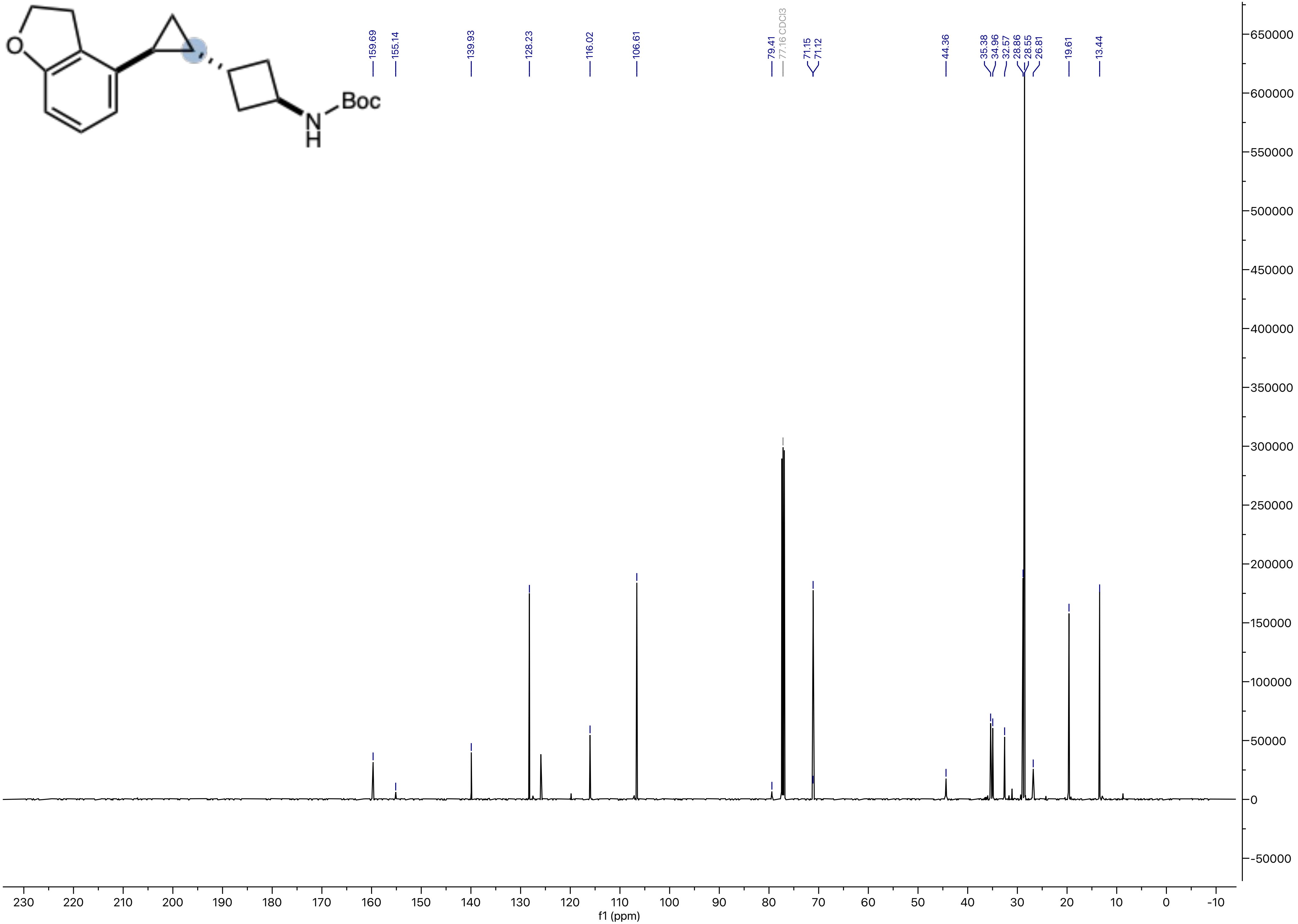

Boc
159.69
155.14
139.93
128.23
116.02
106.61
79.41
77.16 CDCl3
71.15
71.12
44.36
35.38
34.96
32.57
28.86
28.55
26.81
19.61
13.44
f1 (ppm)

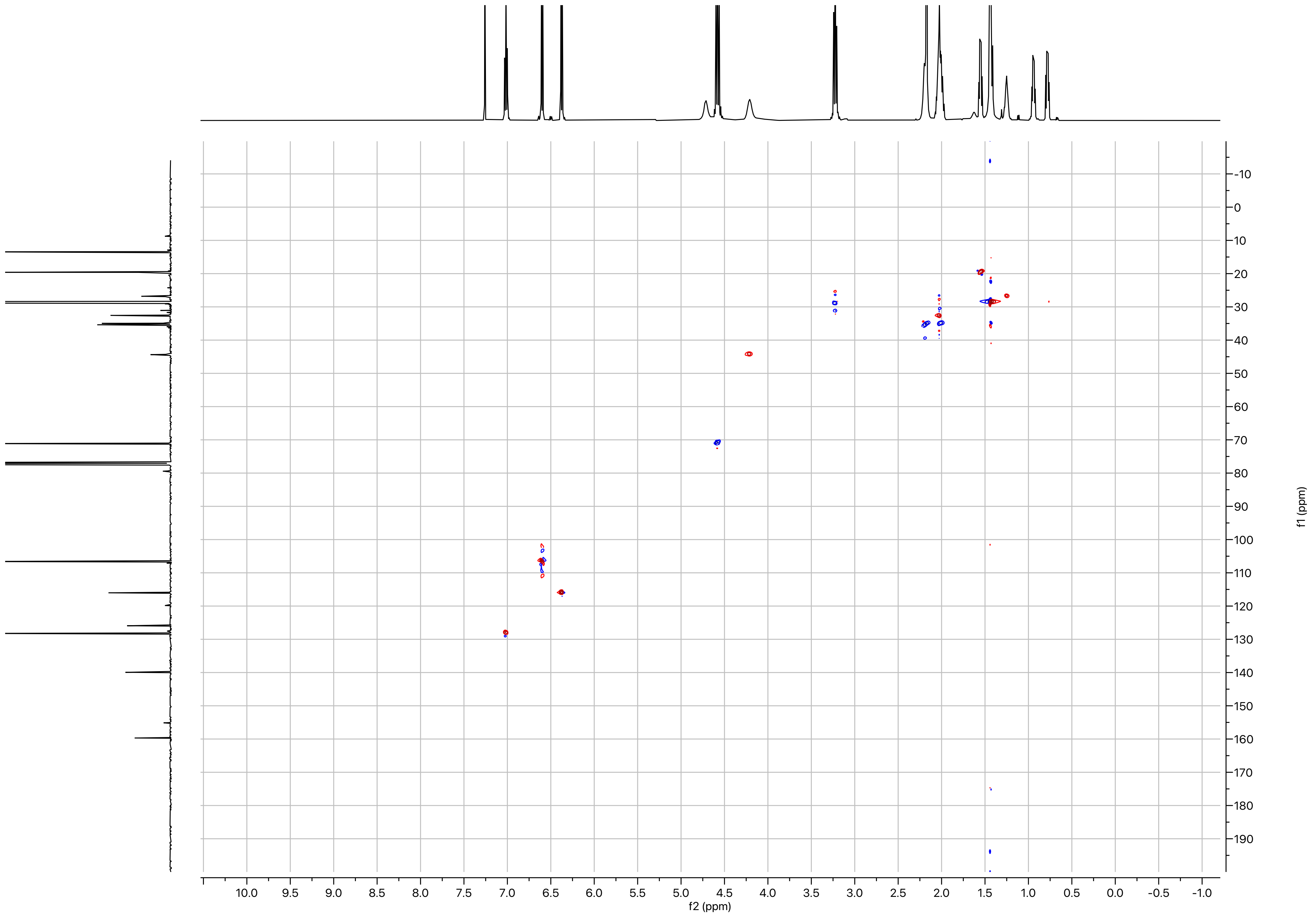
f2 (ppm)
f1 (ppm)

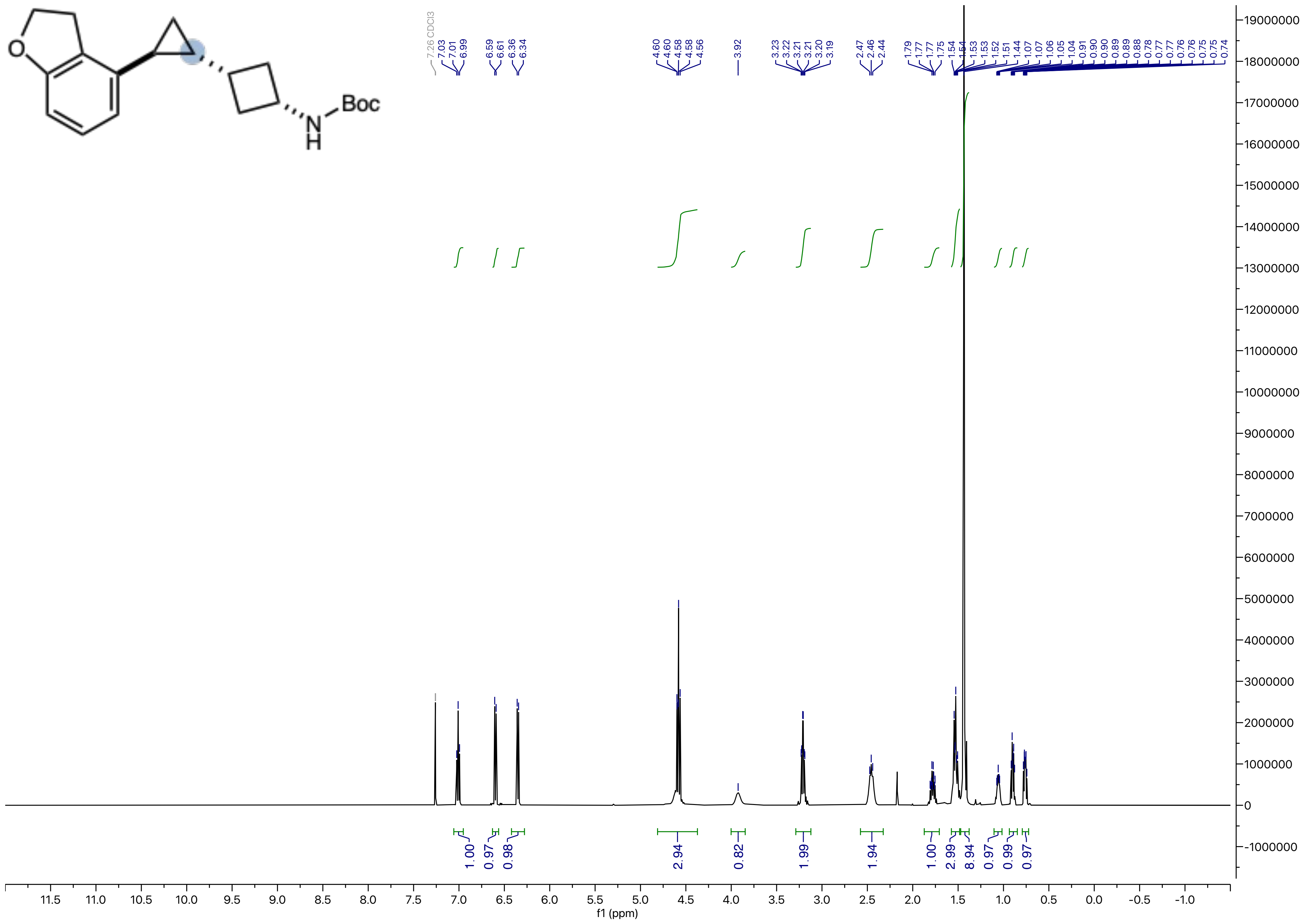

Boc
N
H
O
7.26 CDCl3
7.03
7.01
6.99
6.59
6.61
6.36
6.34
4.60
4.60
4.58
4.58
4.56
3.92
3.23
3.22
3.21
3.21
3.20
3.19
2.47
2.46
2.44
1.79
1.77
1.77
1.75
1.54
1.54
1.53
1.52
1.51
1.44
1.07
1.06
1.05
1.04
0.91
0.90
0.90
0.89
0.88
0.78
0.77
0.77
0.76
0.75
0.75
0.74
1.00
0.97
0.98
2.94
0.82
1.99
1.94
1.00
2.99
8.94
0.97
0.99
0.97
f1 (ppm)

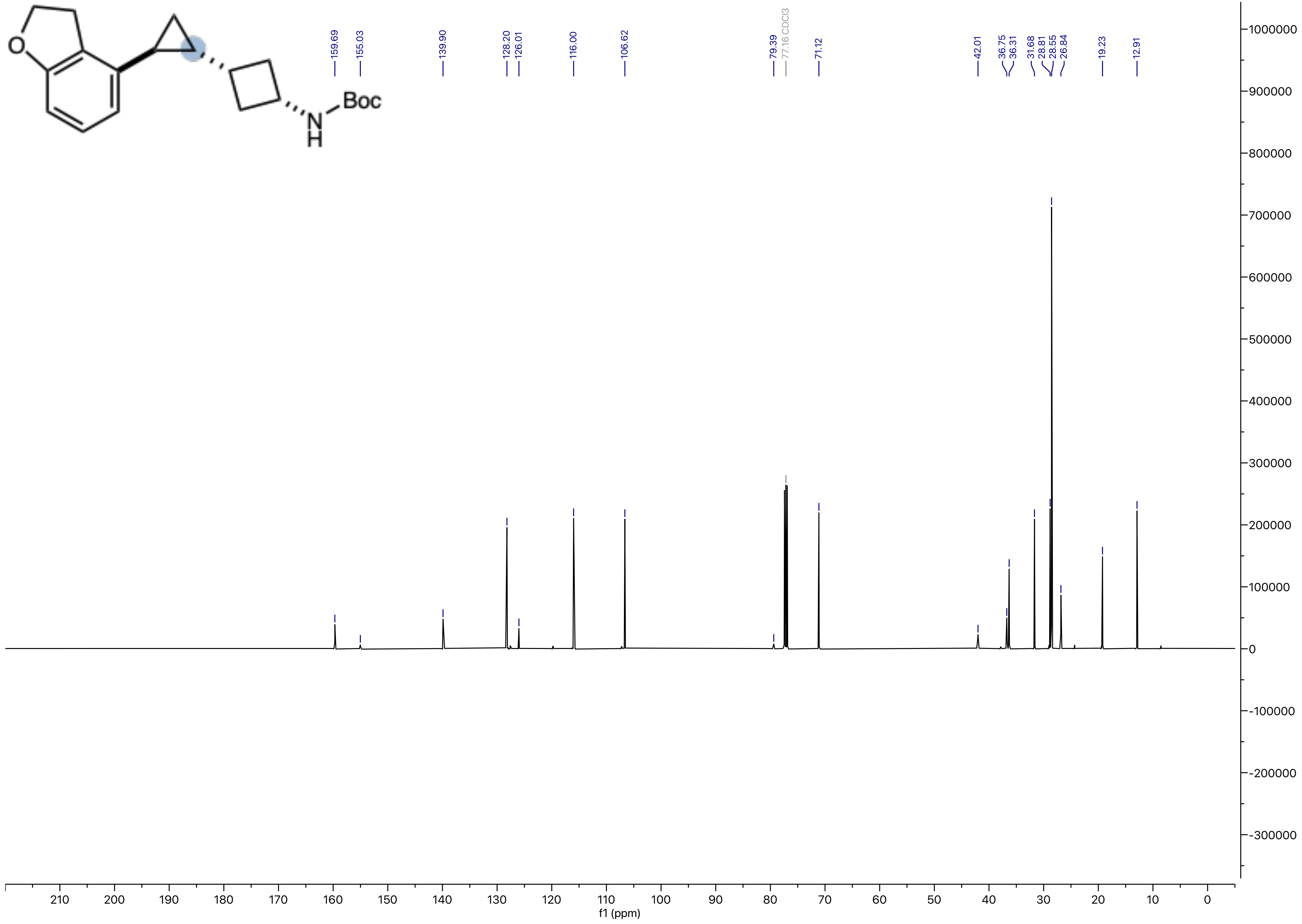

O
N
H
Boc
159.69
155.03
139.90
128.20
126.01
116.00
106.62
79.39
77.16 CDCl3
71.12
42.01
36.75
36.31
31.68
28.81
28.55
26.84
19.23
12.91
f1 (ppm)

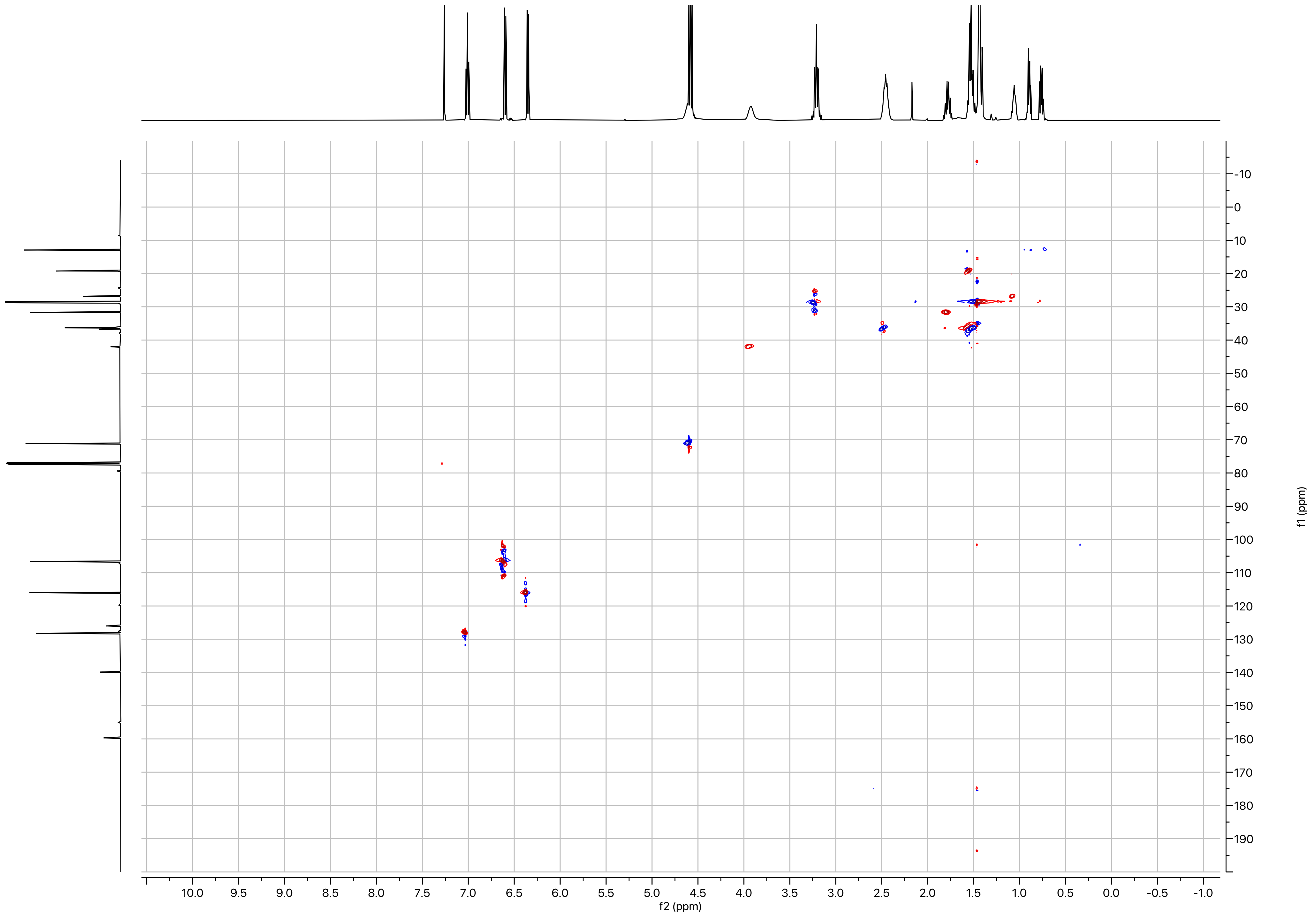

f2 (ppm)
f1 (ppm)

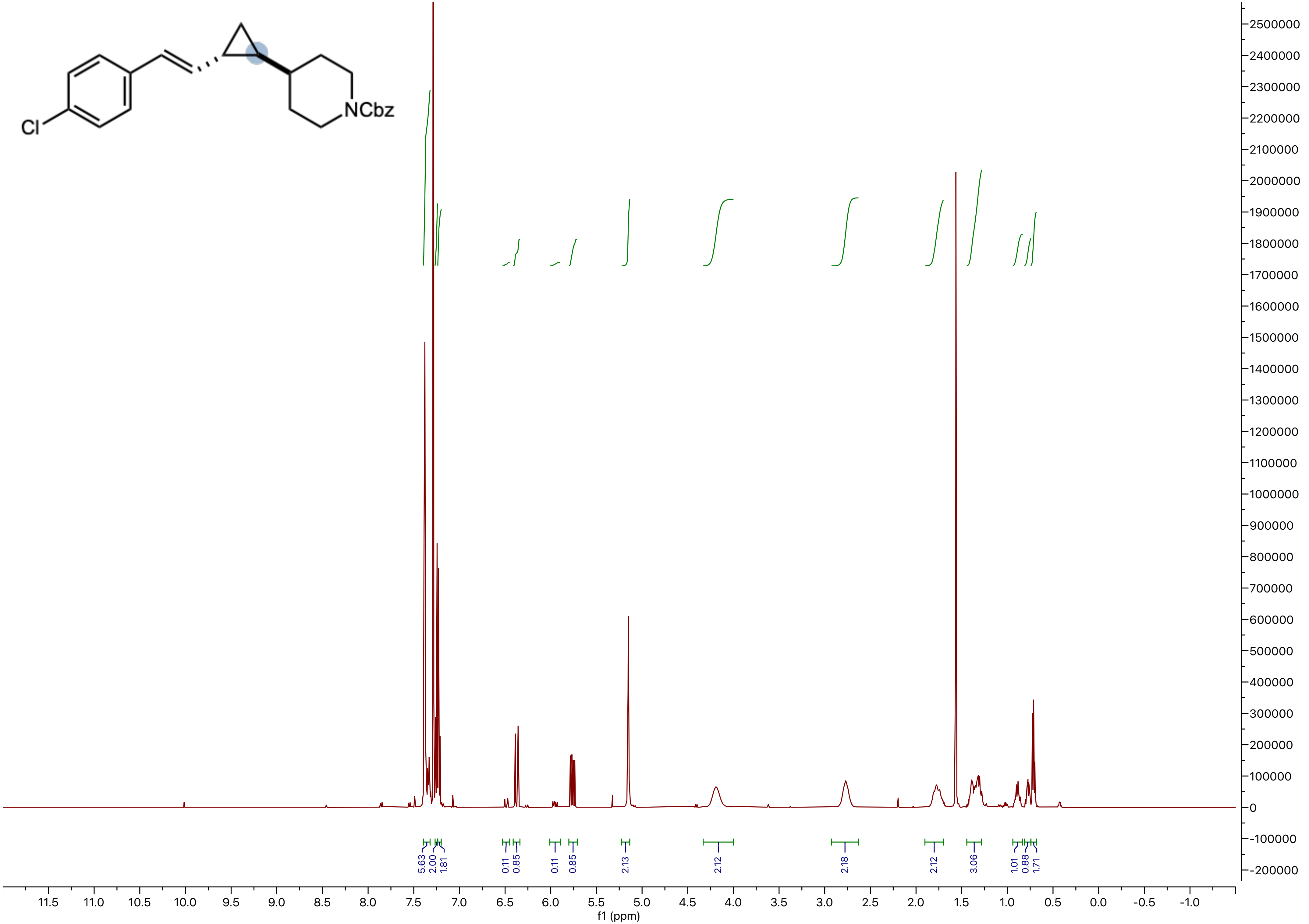

Cl
NCbz
2500000
2400000
2300000
2200000
2100000
2000000
1900000
1800000
1700000
1600000
1500000
1400000
1300000
1200000
1100000
1000000
900000
800000
700000
600000
500000
400000
300000
200000
100000
0
-100000
-200000
5.63
2.00
1.81
0.11
0.85
0.11
0.85
2.13
2.12
2.18
2.12
3.06
1.01
0.88
1.71
11.5
11.0
10.5
10.0
9.5
9.0
8.5
8.0
7.5
7.0
6.5
6.0
5.5
5.0
4.5
4.0
3.5
3.0
2.5
2.0
1.5
1.0
0.5
0.0
-0.5
-1.0
f1 (ppm)

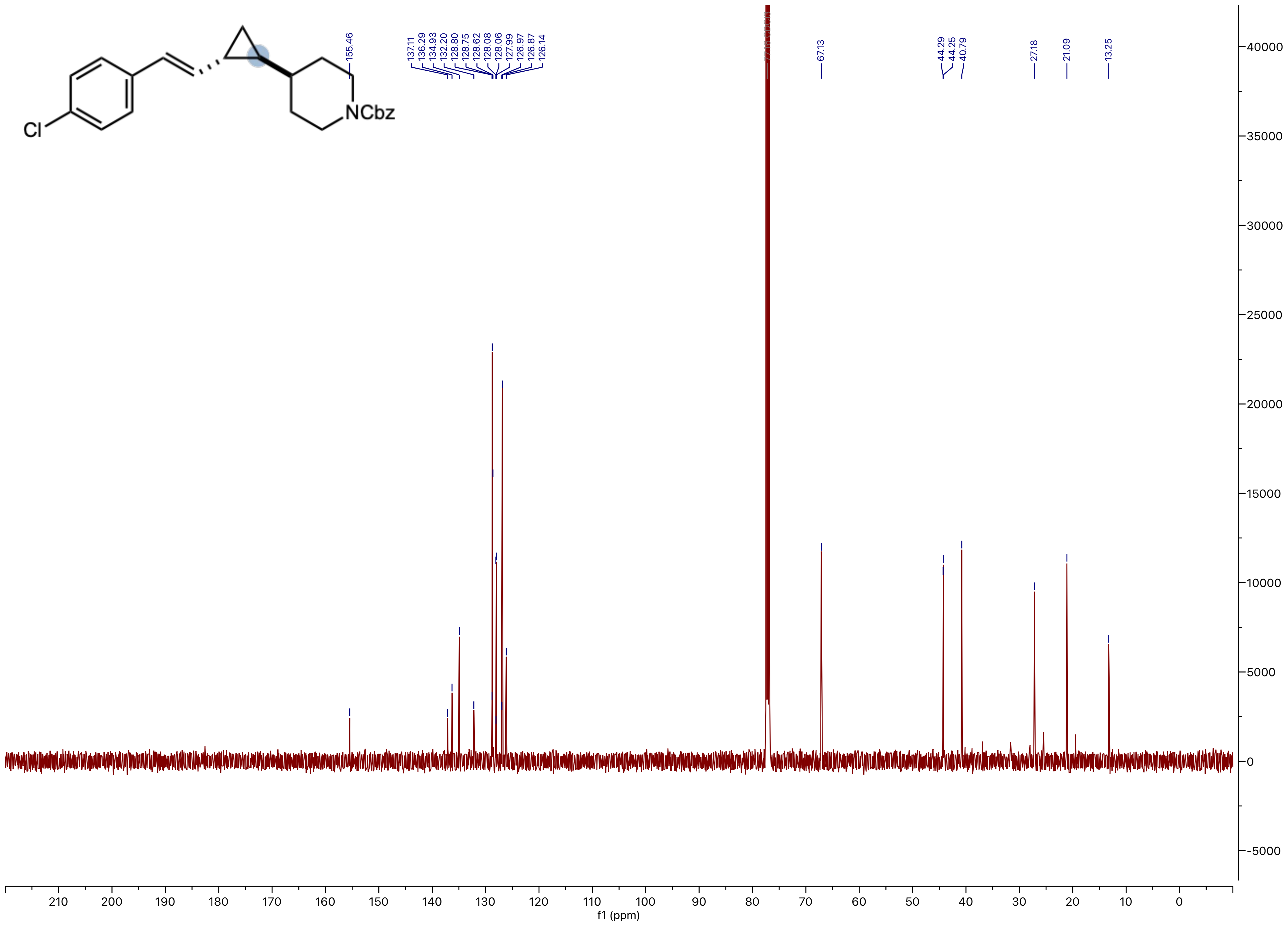

Cl
NCbz
155.46
137.11
136.29
134.93
132.20
128.80
128.75
128.62
128.08
128.06
127.99
126.97
126.87
126.14
67.13
44.29
44.25
40.79
27.18
21.09
13.25
f1 (ppm)

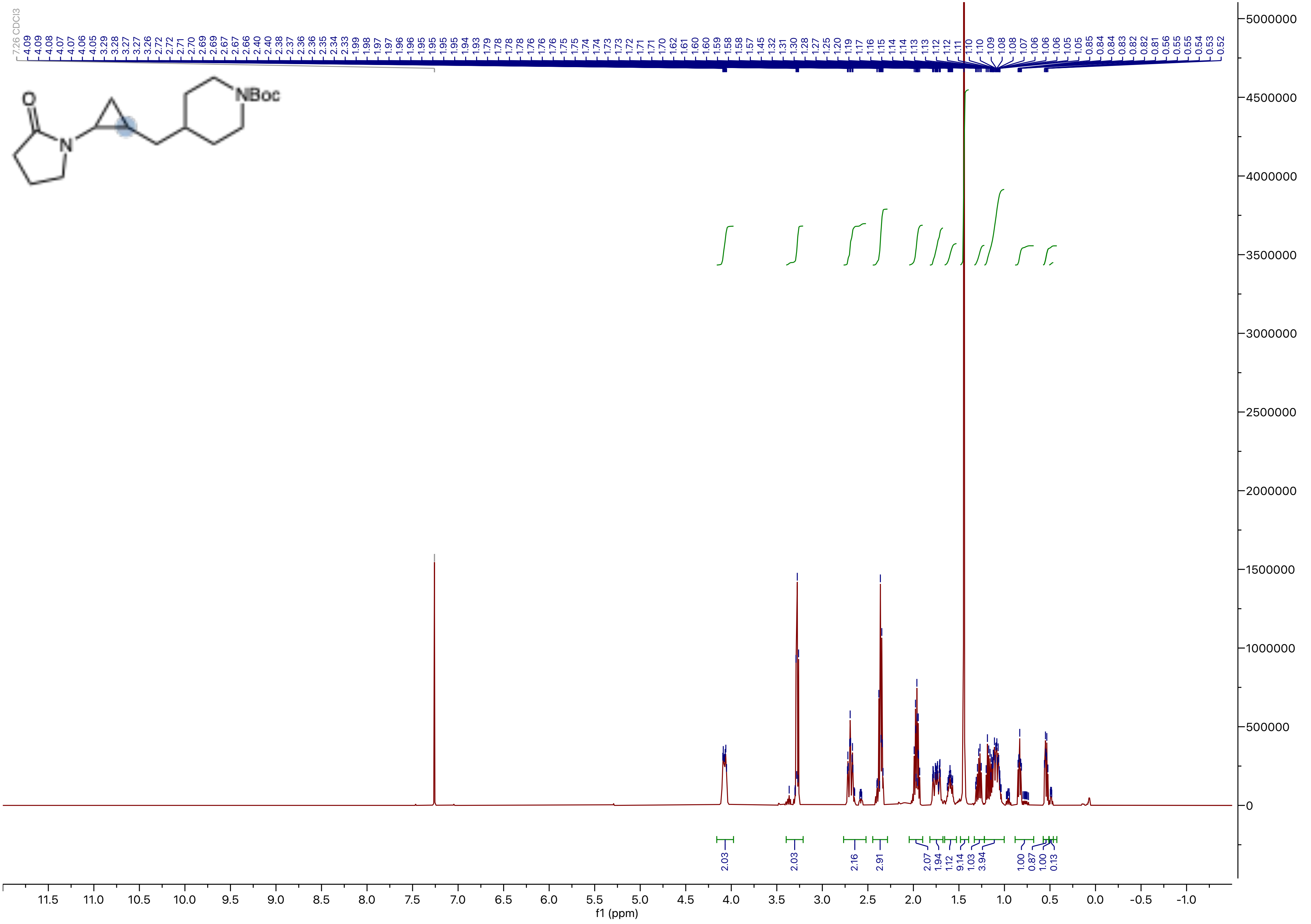

7.26 CDCl3
NBoc
O
N
f1 (ppm)
2.03
2.03
2.16
2.91
2.07
1.94
1.12
9.14
1.03
3.94
1.00
0.87
1.00
0.13

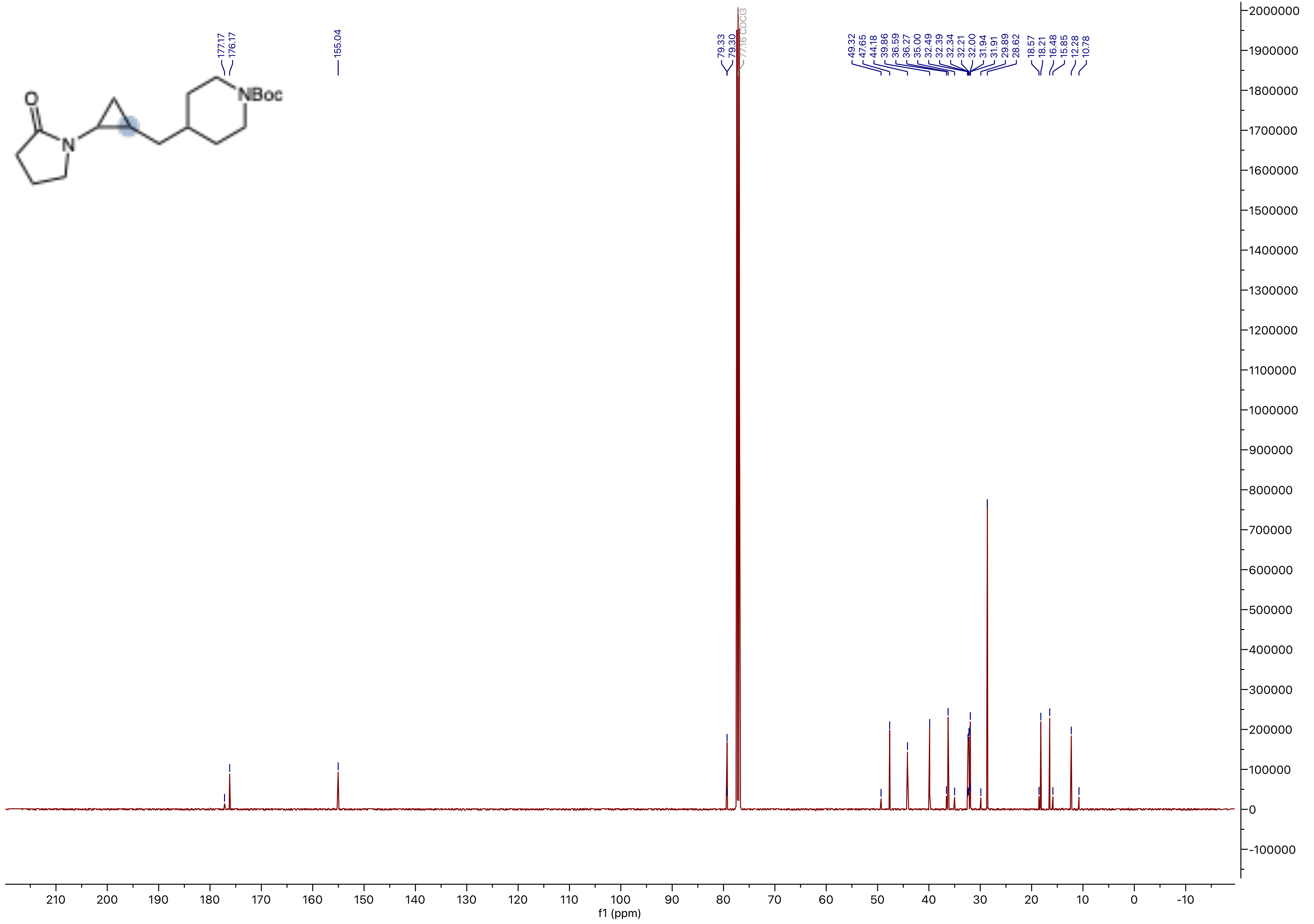

NBoc
O
N
177.17
176.17
155.04
79.33
79.30
77.16 CDCl3
49.32
47.65
44.18
39.86
36.59
36.27
35.00
32.49
32.39
32.34
32.21
32.00
31.94
31.91
29.89
28.62
18.57
18.21
16.48
15.85
12.28
10.78
f1 (ppm)

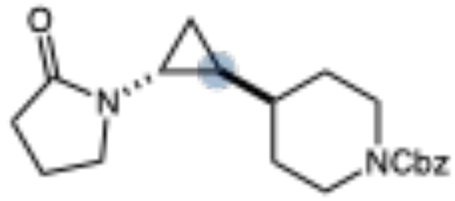
NCbz

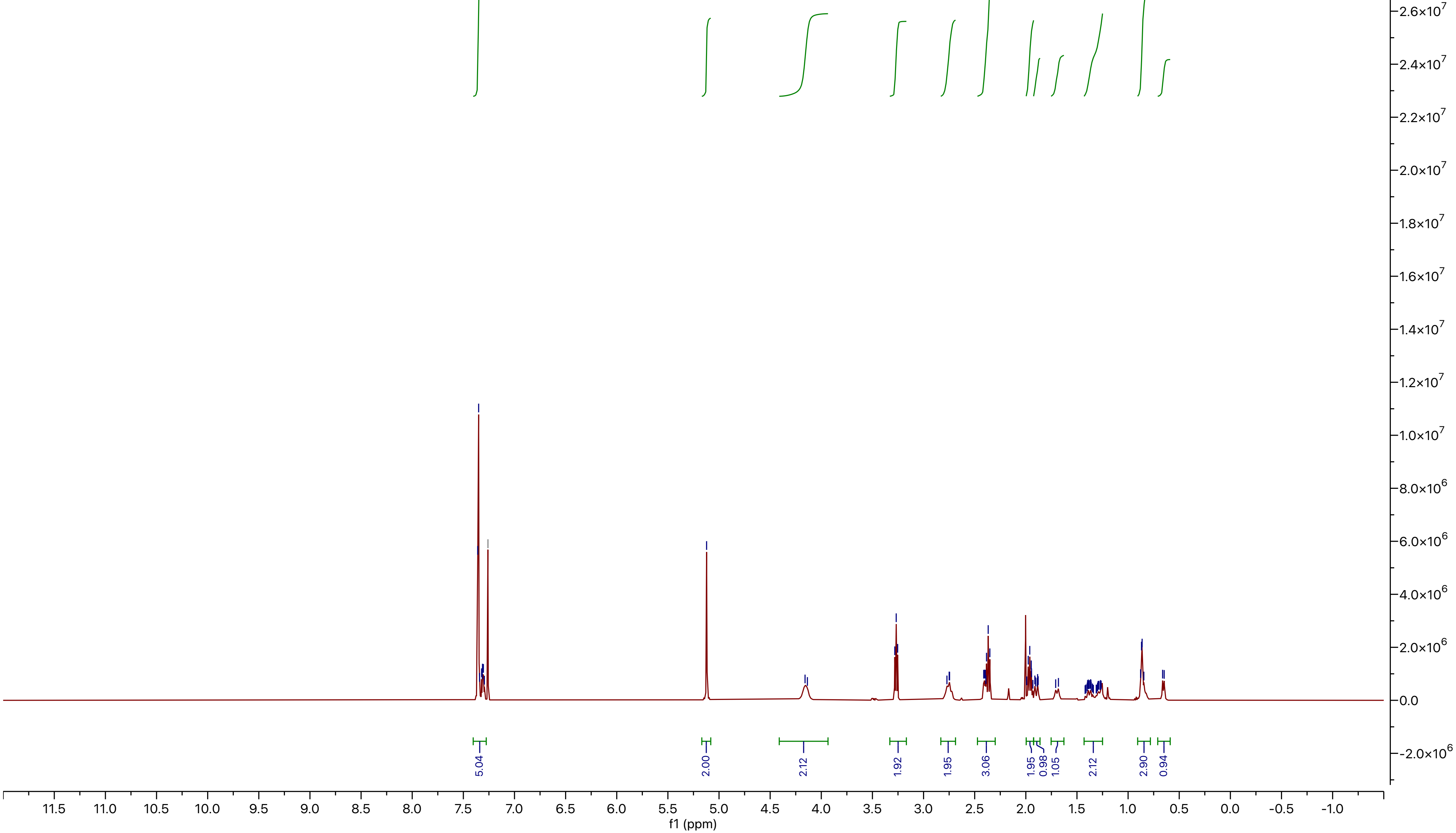
7.36
7.35
7.34
7.32
7.31
7.32
7.30
7.29
7.26 CDCl3
5.12
4.16
4.14
3.28
3.27
3.25
2.77
2.75
2.75
5.04
2.00
2.12
1.92
1.95
3.06
1.95
0.98
1.05
2.12
2.90
0.94
f1 (ppm)

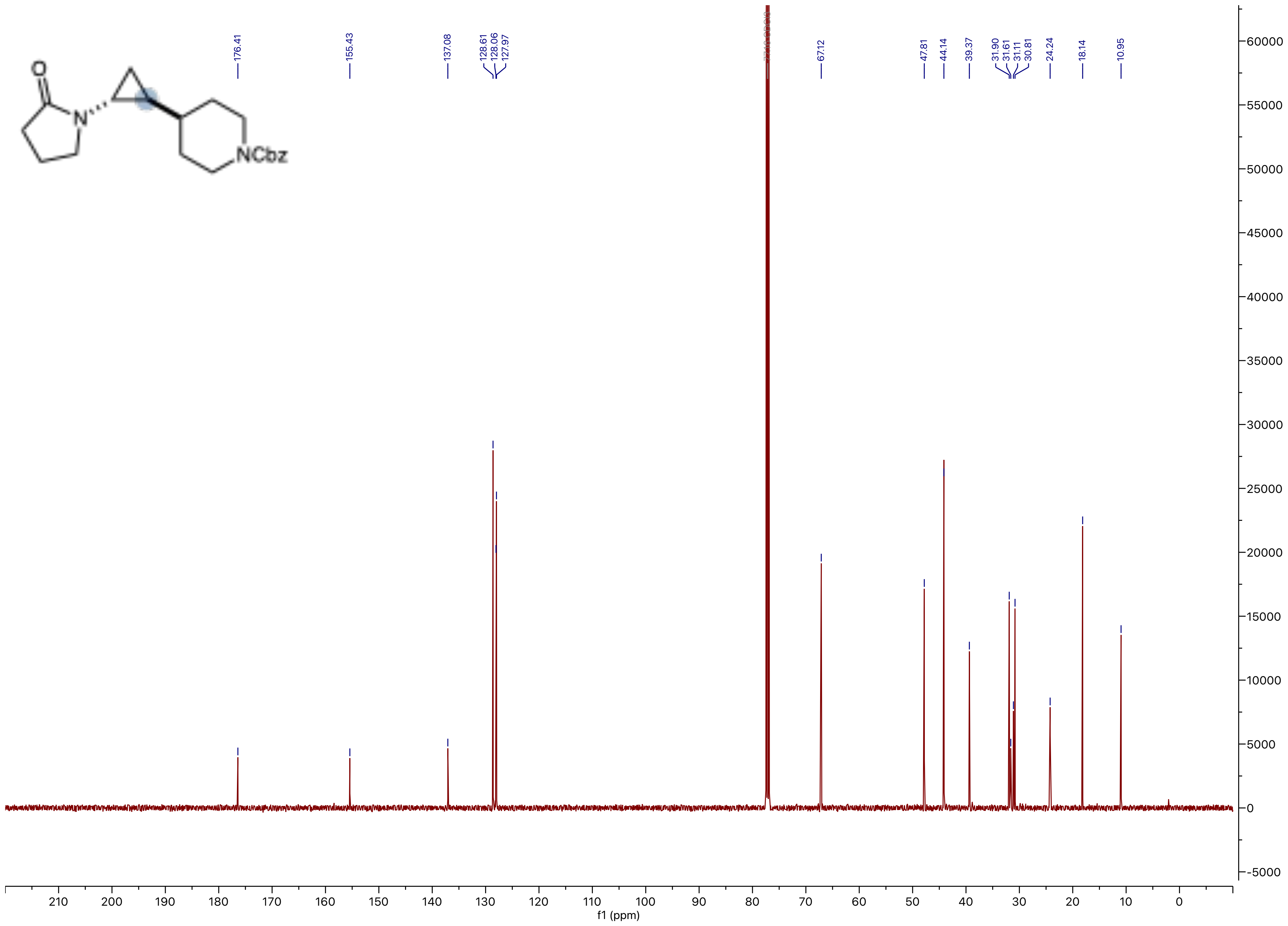

O
N
NCbz
176.41
155.43
137.08
128.61
128.06
127.97
67.12
47.81
44.14
39.37
31.90
31.61
31.11
30.81
24.24
18.14
10.95
f1 (ppm)

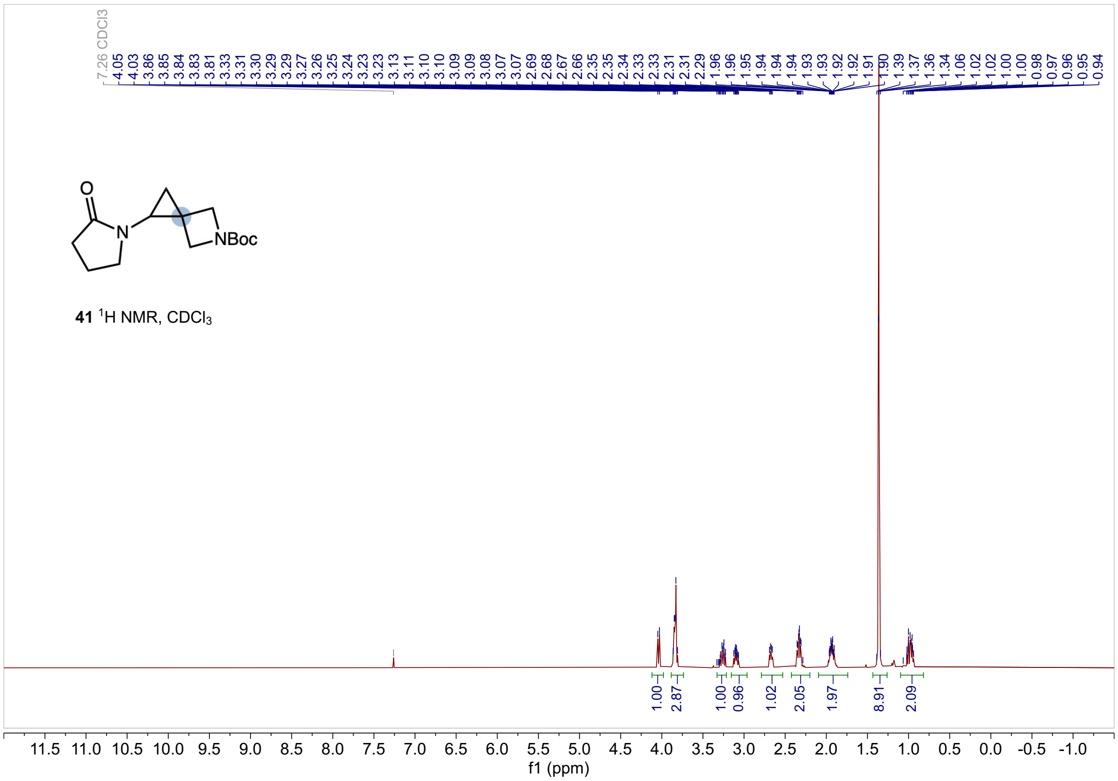

41 ^{1}H NMR, $CDCl_3$

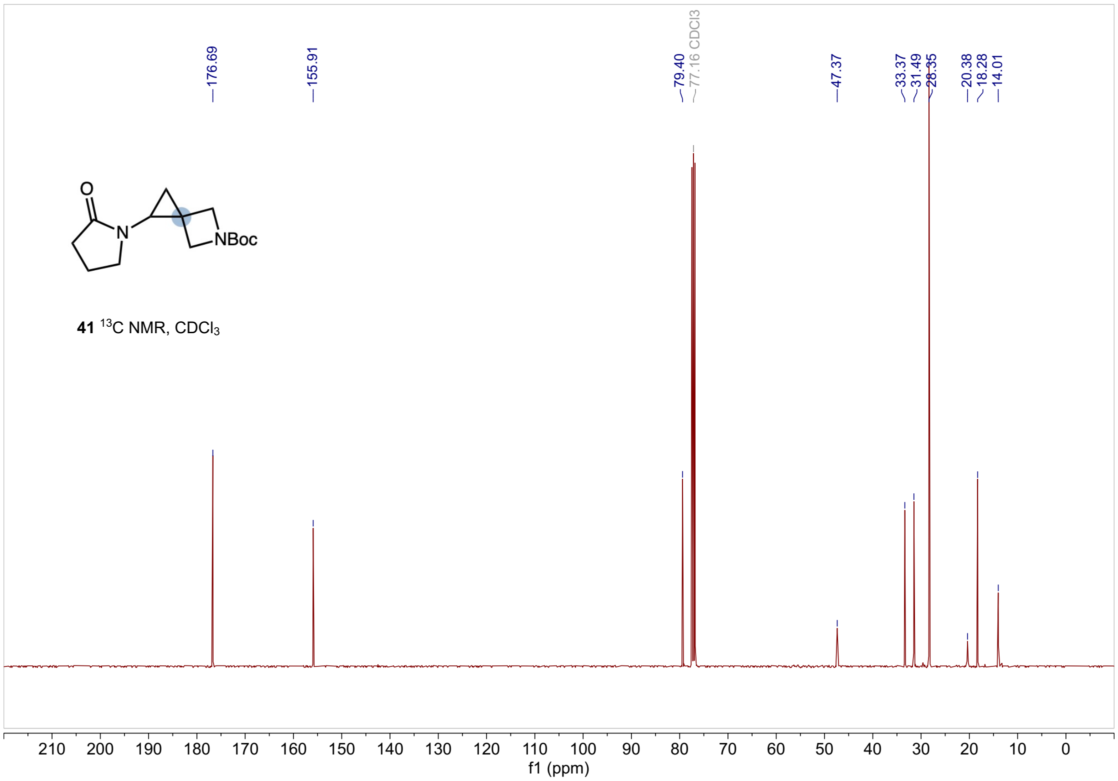

41 ^{13}C NMR, $CDCl_3$

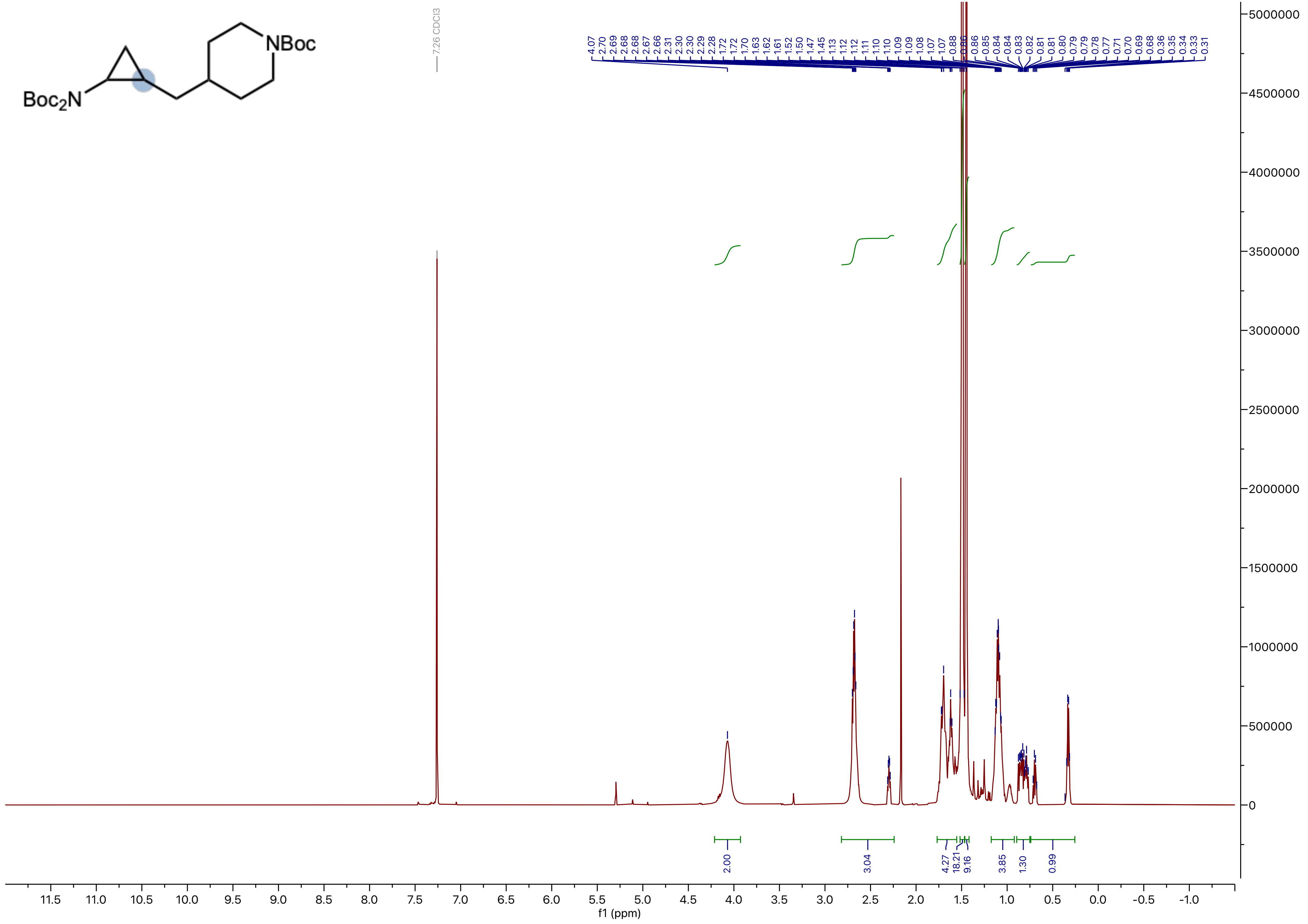

Boc2N
NBoc
7.26 CDCl3
f1 (ppm)
2.00
3.04
4.27
18.21
9.16
3.85
1.30
0.99

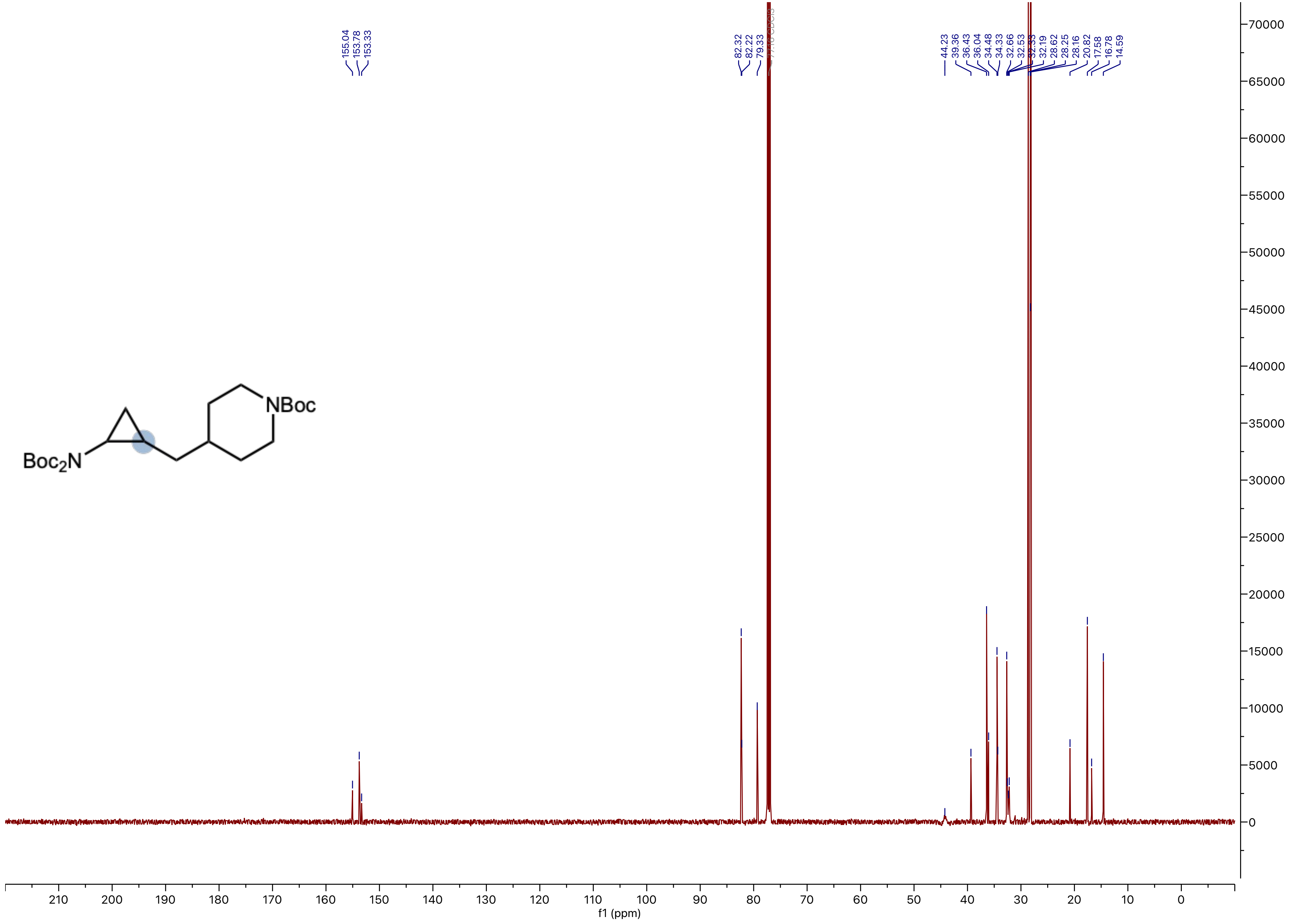

155.04
153.78
153.33
82.32
82.22
79.33
77.16 CDCl3
44.23
39.36
36.43
36.04
34.48
34.33
32.66
32.53
32.33
32.19
28.62
28.25
28.16
20.82
17.58
16.78
14.59
NBoc
Boc2N
70000
65000
60000
55000
50000
45000
40000
35000
30000
25000
20000
15000
10000
5000
0
210
200
190
180
170
160
150
140
130
120
110
100
90
80
70
60
50
40
30
20
10
0
f1 (ppm)

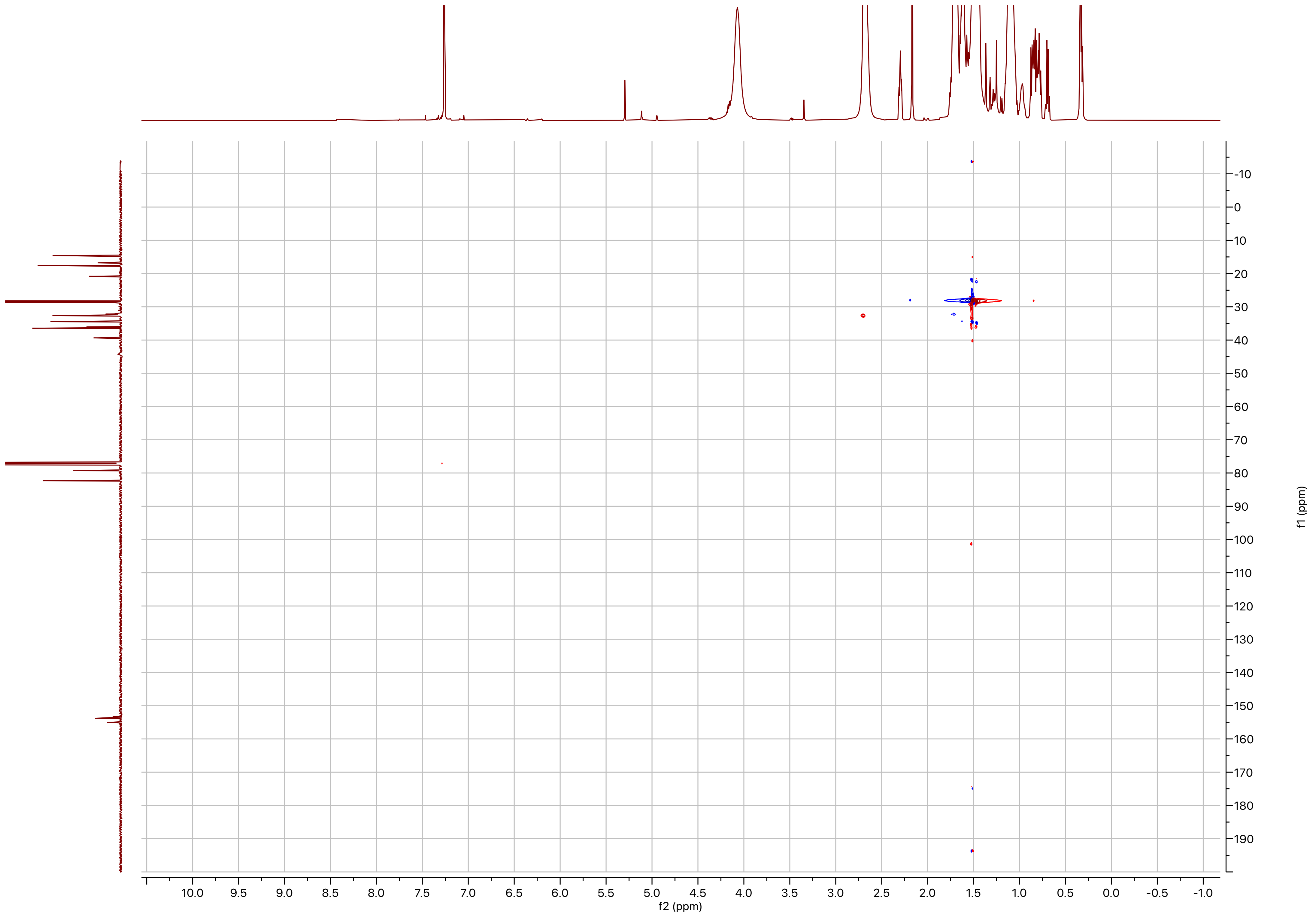

10.0 9.5 9.0 8.5 8.0 7.5 7.0 6.5 6.0 5.5 5.0 4.5 4.0 3.5 3.0 2.5 2.0 1.5 1.0 0.5 0.0 -0.5 -1.0
f2 (ppm)
-10 0 10 20 30 40 50 60 70 80 90 100 110 120 130 140 150 160 170 180 190
f1 (ppm)

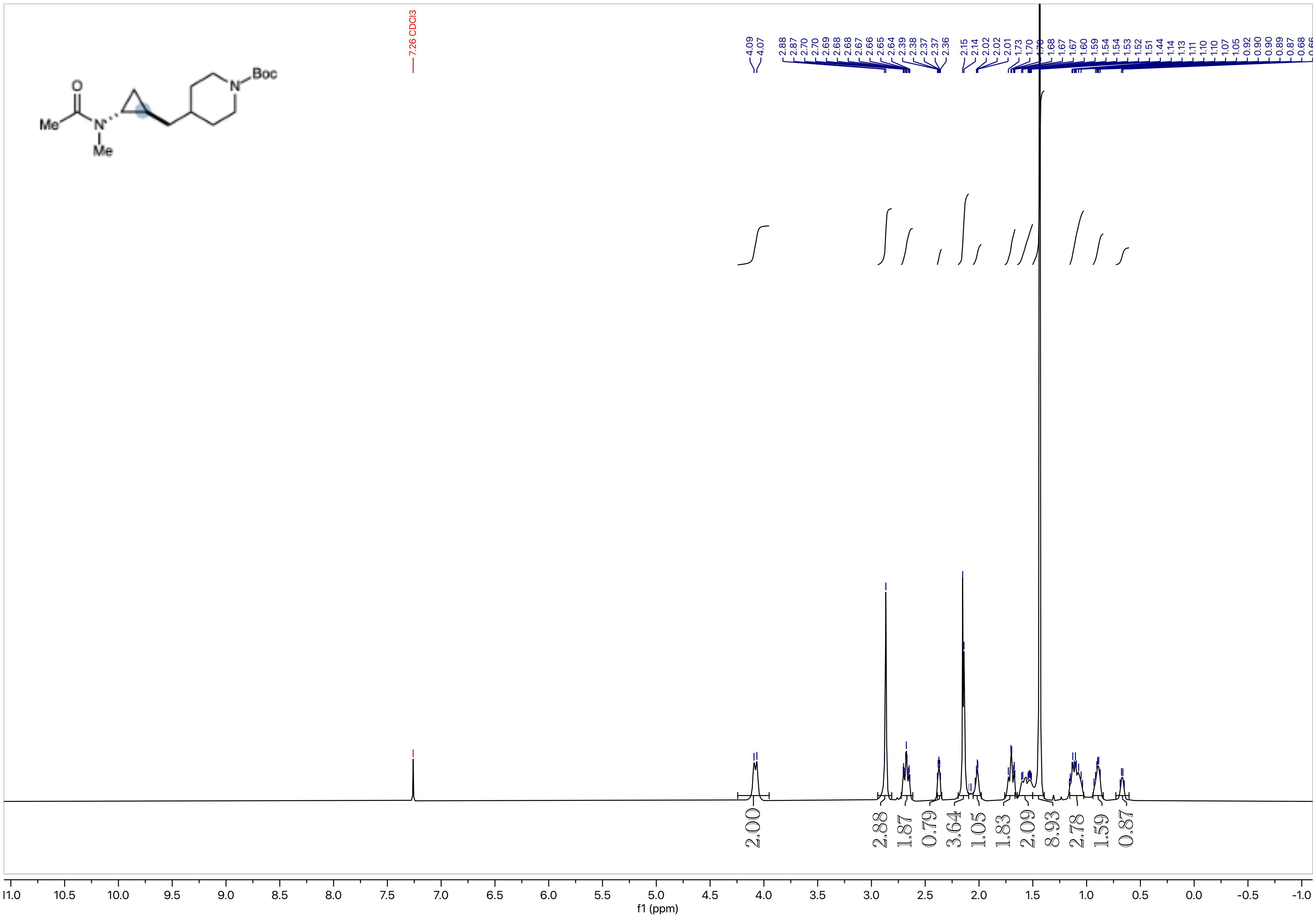

O
Me
N
Me
N
Boc
7.26 CDCl3
4.09
4.07
2.88
2.87
2.70
2.70
2.69
2.68
2.68
2.67
2.66
2.65
2.64
2.39
2.38
2.37
2.37
2.36
2.15
2.14
2.02
2.02
2.01
1.73
1.70
1.68
1.67
1.60
1.59
1.54
1.54
1.53
1.52
1.51
1.44
1.14
1.13
1.11
1.10
1.10
1.07
1.05
0.92
0.90
0.90
0.89
0.87
0.68
2.00
2.88
1.87
0.79
3.64
1.05
1.83
2.09
8.93
2.78
1.59
0.87
11.0 10.5 10.0 9.5 9.0 8.5 8.0 7.5 7.0 6.5 6.0 5.5 5.0 4.5 4.0 3.5 3.0 2.5 2.0 1.5 1.0 0.5 0.0 -0.5 -1.0
f1 (ppm)

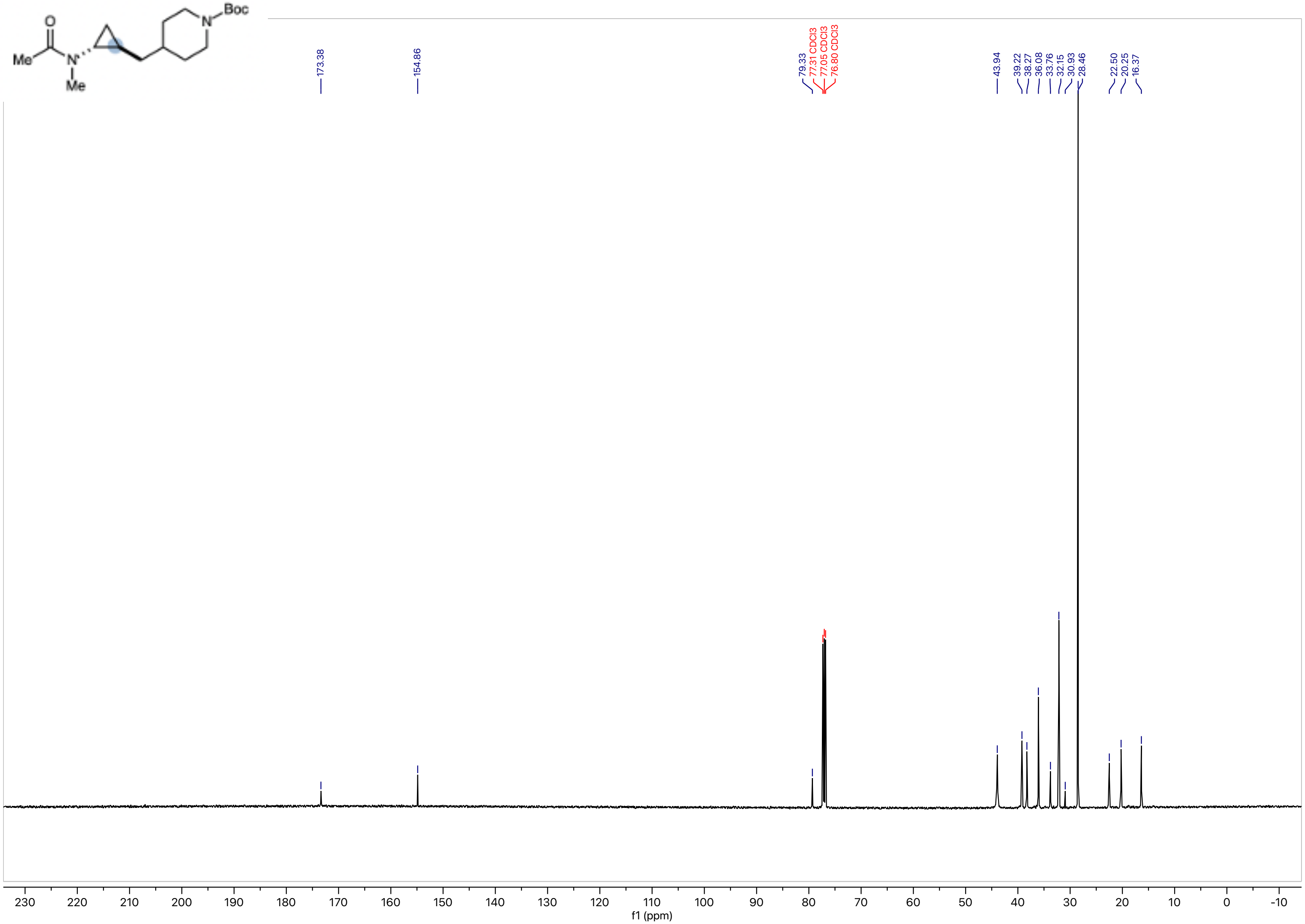
O
Me
N
Me
N
Boc
173.38
154.86
79.33
77.31 CDCl3
77.05 CDCl3
76.80 CDCl3
43.94
39.22
38.27
36.08
33.76
32.15
30.93
28.46
22.50
20.25
16.37
230 220 210 200 190 180 170 160 150 140 130 120 110 100 90 80 70 60 50 40 30 20 10 0 -10
f1 (ppm)

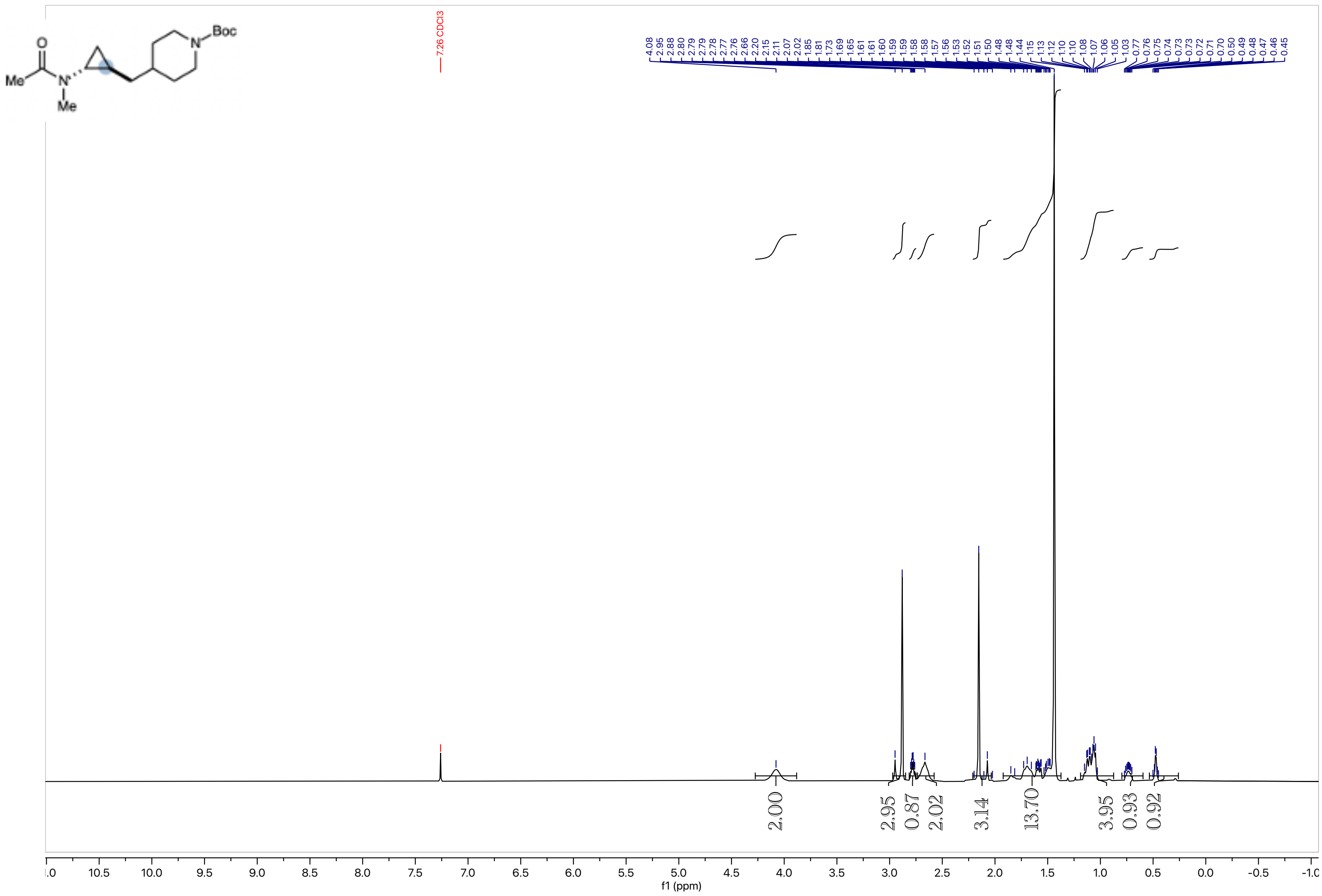

O
Me
N
Me
N
Boc
7.26 CDCl3
4.08 2.95 2.88 2.80 2.79 2.78 2.77 2.76 2.66 2.20 2.15 2.11 2.07 2.02 1.85 1.81 1.73 1.69 1.65 1.61 1.61 1.60 1.59 1.59 1.58 1.58 1.57 1.56 1.53 1.52 1.51 1.50 1.48 1.48 1.44 1.15 1.13 1.12 1.10 1.10 1.08 1.07 1.06 1.05 1.03 0.77 0.76 0.75 0.74 0.73 0.73 0.72 0.71 0.70 0.50 0.49 0.48 0.47 0.46 0.45
2.00
2.95
0.87
2.02
3.14
13.70
3.95
0.93
0.92
10.5 10.0 9.5 9.0 8.5 8.0 7.5 7.0 6.5 6.0 5.5 5.0 4.5 4.0 3.5 3.0 2.5 2.0 1.5 1.0 0.5 0.0 -0.5
f1 (ppm)

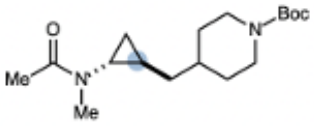
O
Me
N
Me
N
Boc

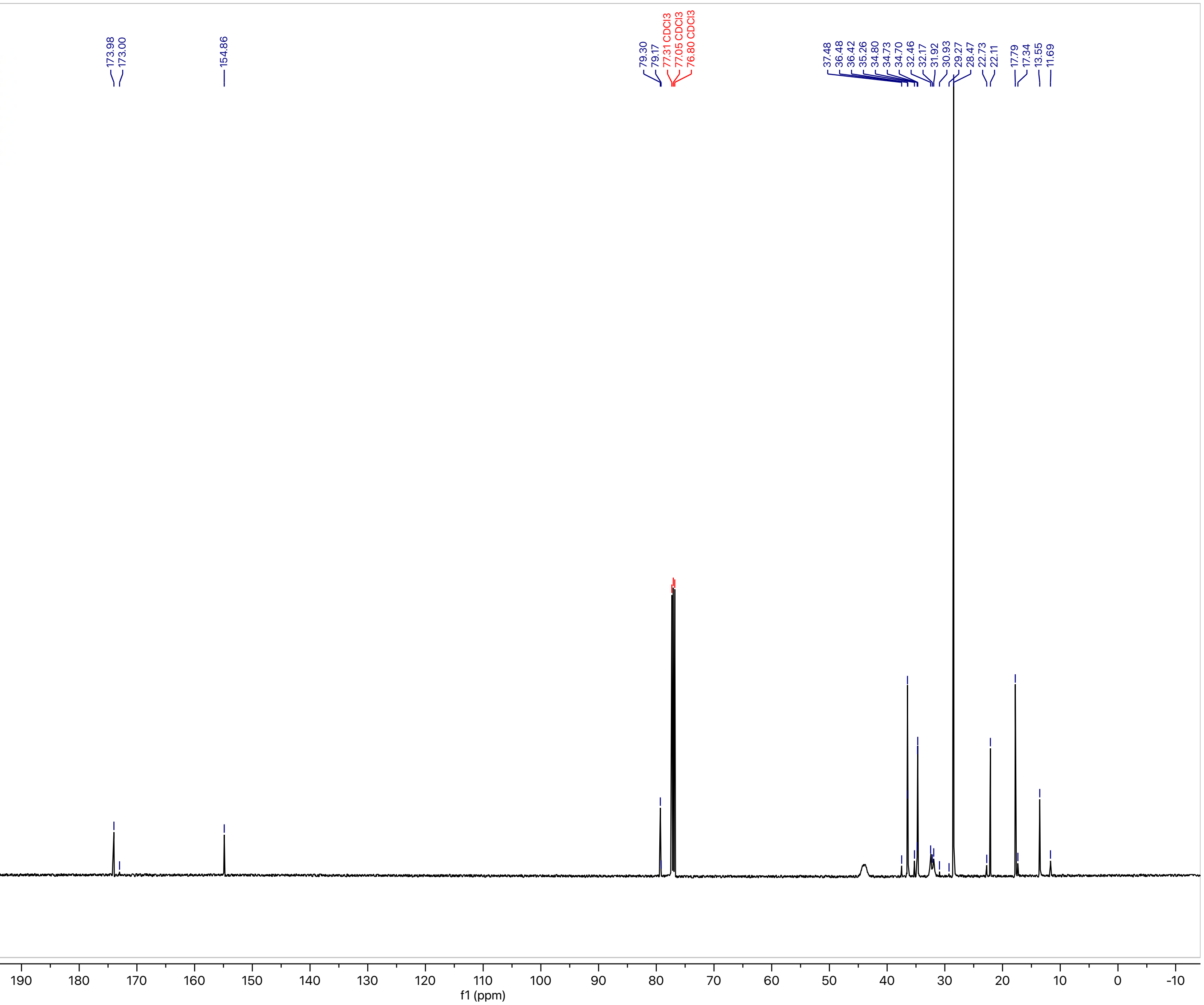
173.98
173.00
154.86
79.30
79.17
77.31 CDCl3
77.05 CDCl3
76.80 CDCl3
37.48
36.48
36.42
35.26
34.80
34.73
34.70
32.46
32.17
31.92
30.93
29.27
28.47
22.73
22.11
17.79
17.34
13.55
11.69
230
220
210
200
190
180
170
160
150
140
130
120
110
100
90
80
70
60
50
40
30
20
10
0
-10
f1 (ppm)

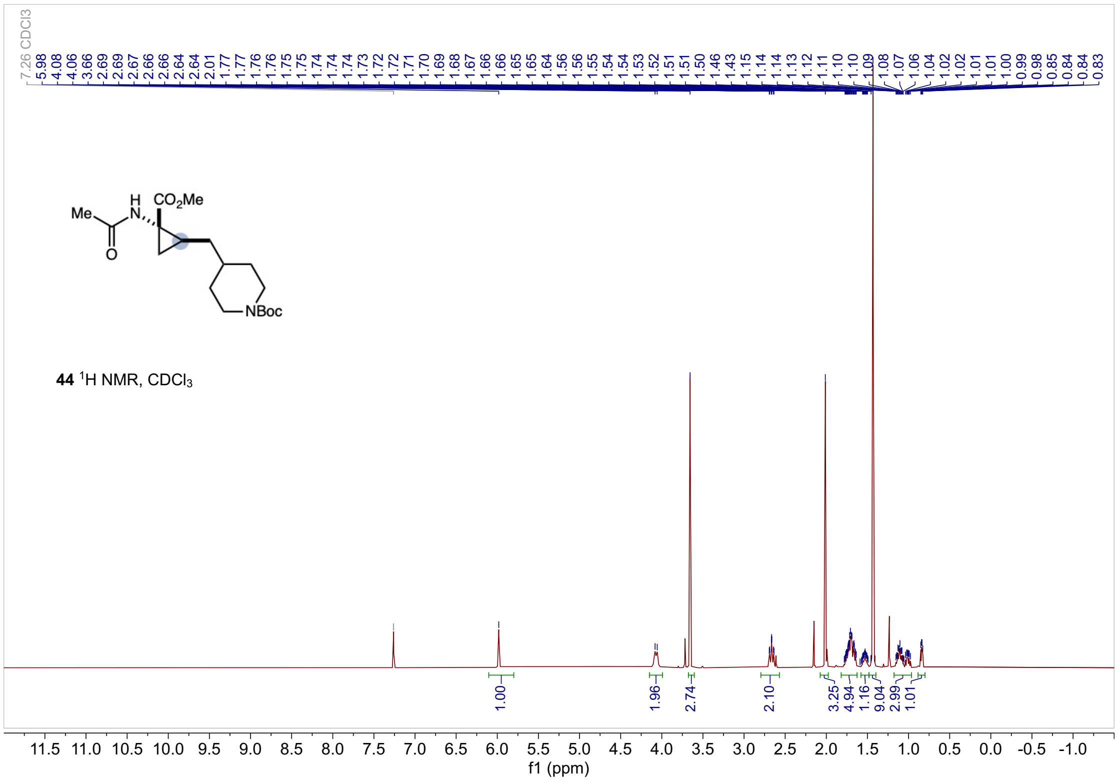
44 ¹H NMR, CDCl₃
f1 (ppm)

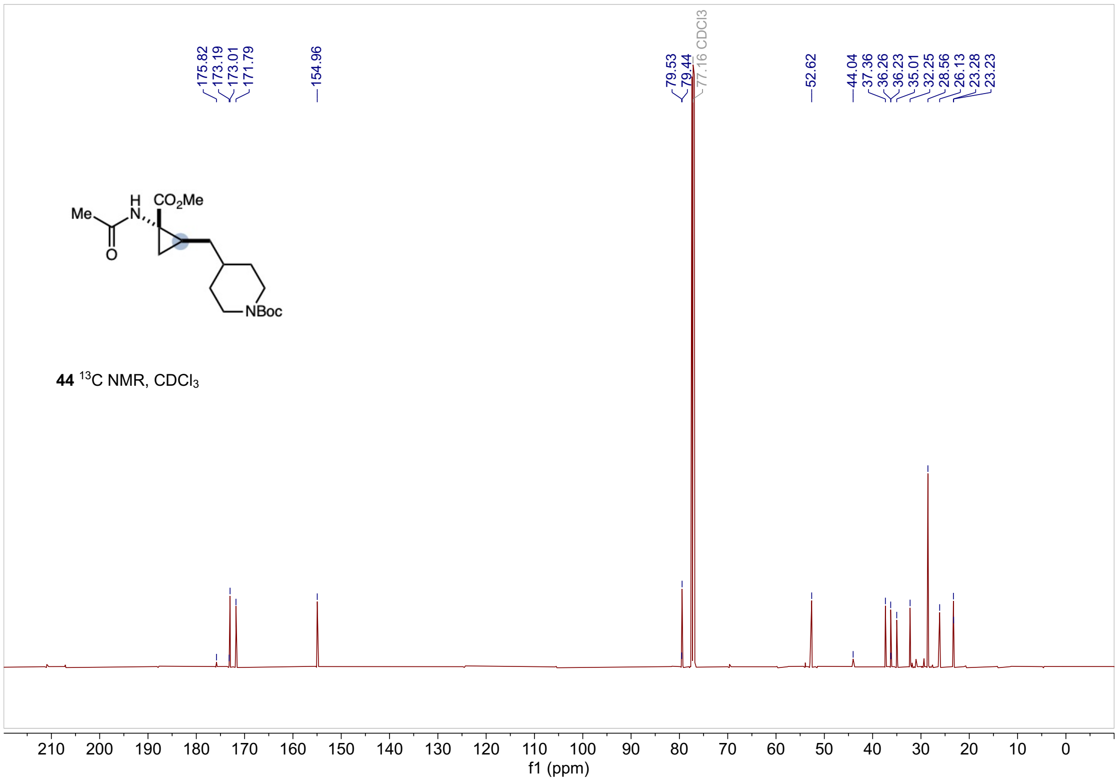
44 ¹³C NMR, CDCl₃
f1 (ppm)

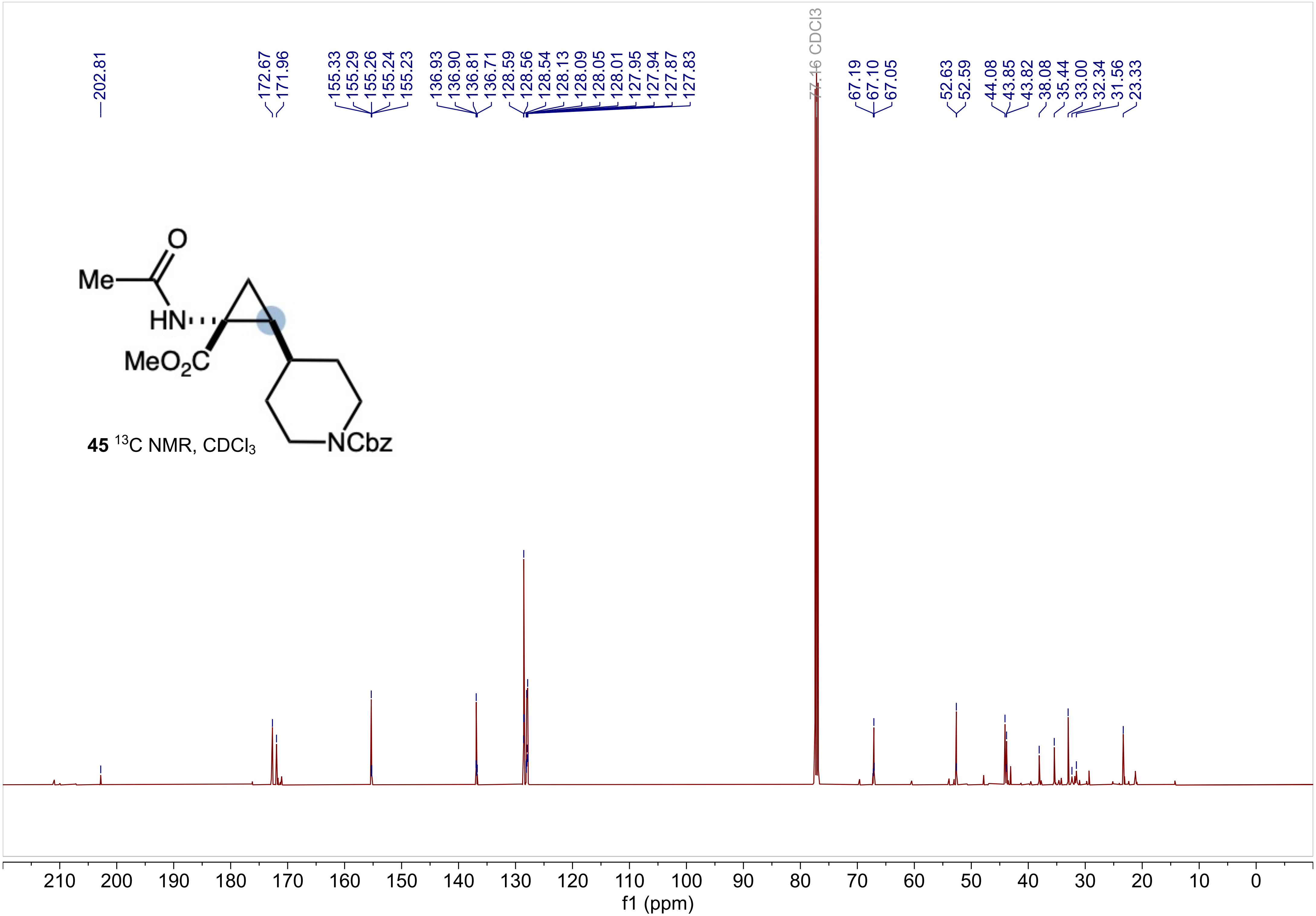

202.81
172.67
171.96
155.33
155.29
155.26
155.24
155.23
136.93
136.90
136.81
136.71
128.59
128.56
128.54
128.13
128.09
128.05
128.01
127.95
127.94
127.87
127.83
77.16 CDCl3
67.19
67.10
67.05
52.63
52.59
44.08
43.85
43.82
38.08
35.44
33.00
32.34
31.56
23.33
Me
O
HN
MeO2C
NCbz
45 13C NMR, CDCl3
210
200
190
180
170
160
150
140
130
120
110
100
90
80
70
60
50
40
30
20
10
0
f1 (ppm)

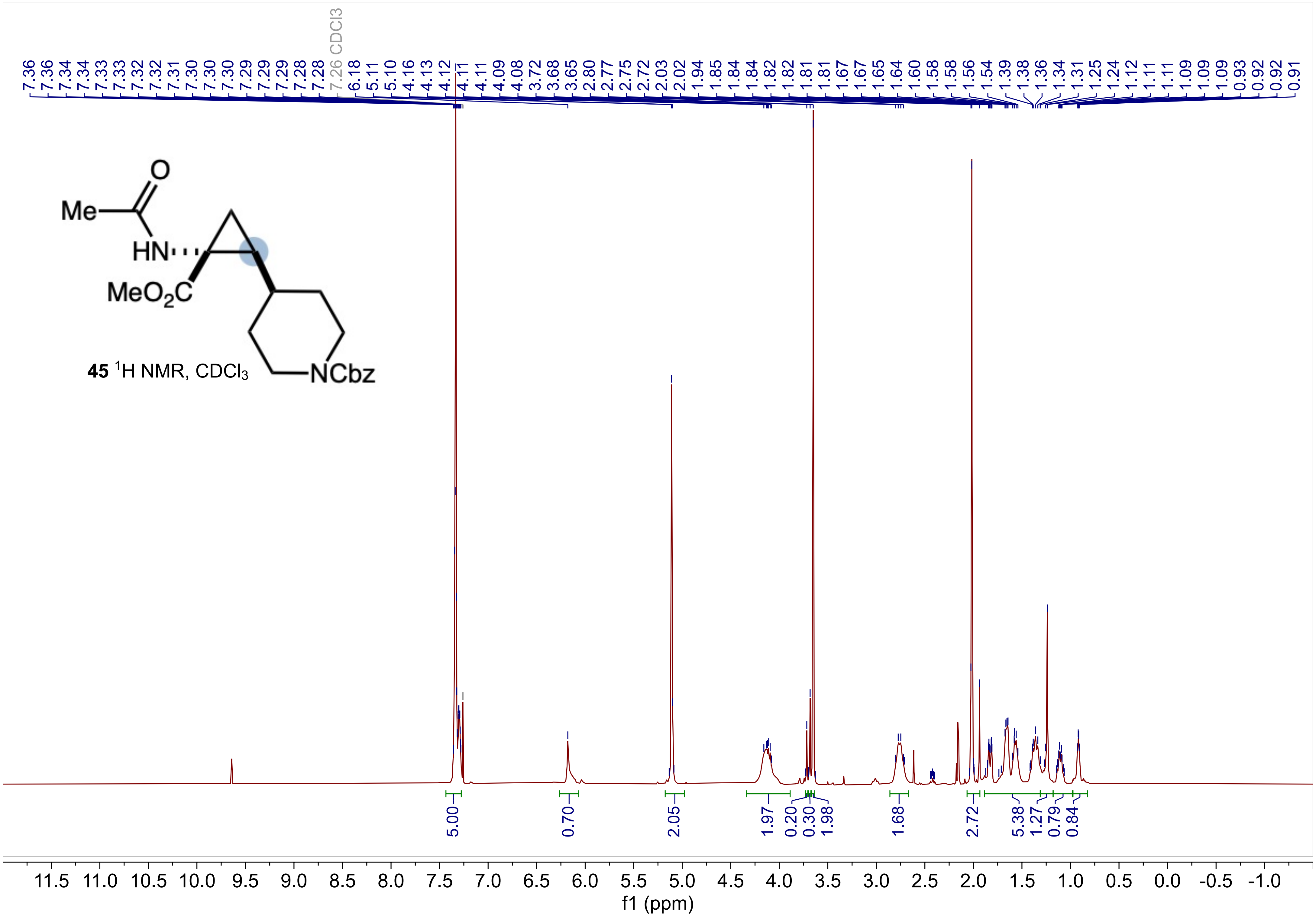
7.36 7.36 7.34 7.34 7.33 7.33 7.32 7.32 7.31 7.30 7.30 7.30 7.29 7.29 7.29 7.28 7.28 7.26 CDCl3 6.18 5.11 5.10 4.16 4.13 4.12 4.11 4.11 4.09 4.08 3.72 3.68 3.65 2.80 2.77 2.75 2.72 2.03 2.02 1.94 1.85 1.84 1.84 1.82 1.82 1.81 1.81 1.67 1.67 1.65 1.64 1.60 1.58 1.58 1.56 1.54 1.39 1.38 1.36 1.34 1.31 1.25 1.24 1.12 1.11 1.11 1.09 1.09 0.93 0.92 0.92 0.91
O
Me
HN
MeO2C
NCbz
45 1H NMR, CDCl3
5.00 0.70 2.05 1.97 0.20 0.30 1.98 1.68 2.72 5.38 1.27 0.79 0.84
11.5 11.0 10.5 10.0 9.5 9.0 8.5 8.0 7.5 7.0 6.5 6.0 5.5 5.0 4.5 4.0 3.5 3.0 2.5 2.0 1.5 1.0 0.5 0.0 -0.5 -1.0
f1 (ppm)

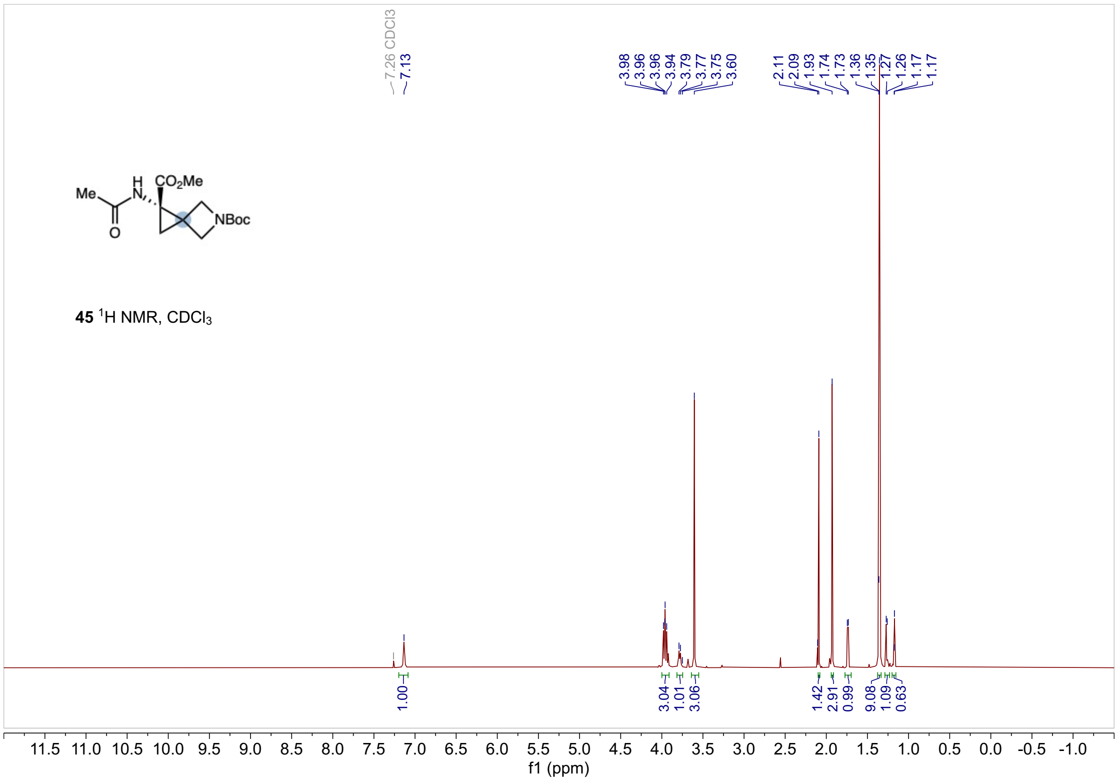
7.26 CDCl3
7.13
3.98
3.96
3.96
3.94
3.79
3.77
3.75
3.60
2.11
2.09
1.93
1.74
1.73
1.36
1.35
1.27
1.26
1.17
1.17
Me
H
N
CO2Me
NBoc
O
45 1H NMR, CDCl3
1.00
3.04
1.01
3.06
1.42
2.91
0.99
9.08
1.09
0.63
11.5 11.0 10.5 10.0 9.5 9.0 8.5 8.0 7.5 7.0 6.5 6.0 5.5 5.0 4.5 4.0 3.5 3.0 2.5 2.0 1.5 1.0 0.5 0.0 -0.5 -1.0
f1 (ppm)

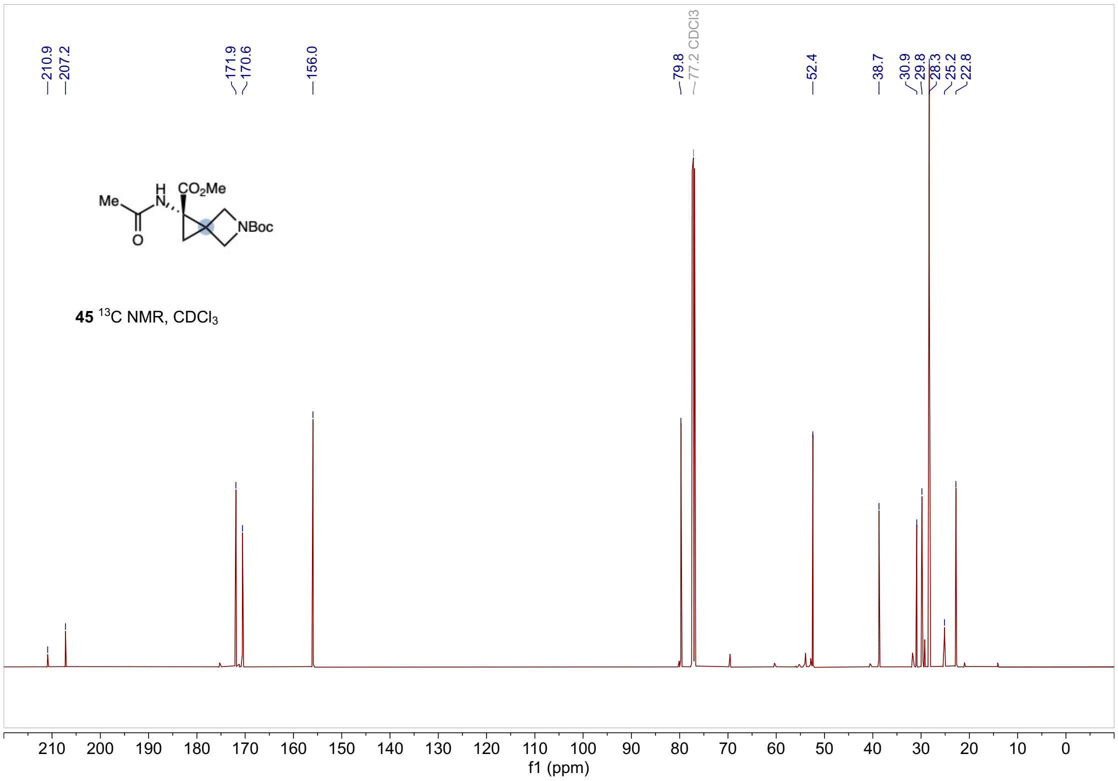
210.9
207.2
171.9
170.6
156.0
79.8
77.2 CDCl3
52.4
38.7
30.9
29.8
28.3
25.2
22.8
Me
H
N
CO2Me
NBoc
O
45 13C NMR, CDCl3
210 200 190 180 170 160 150 140 130 120 110 100 90 80 70 60 50 40 30 20 10 0
f1 (ppm)

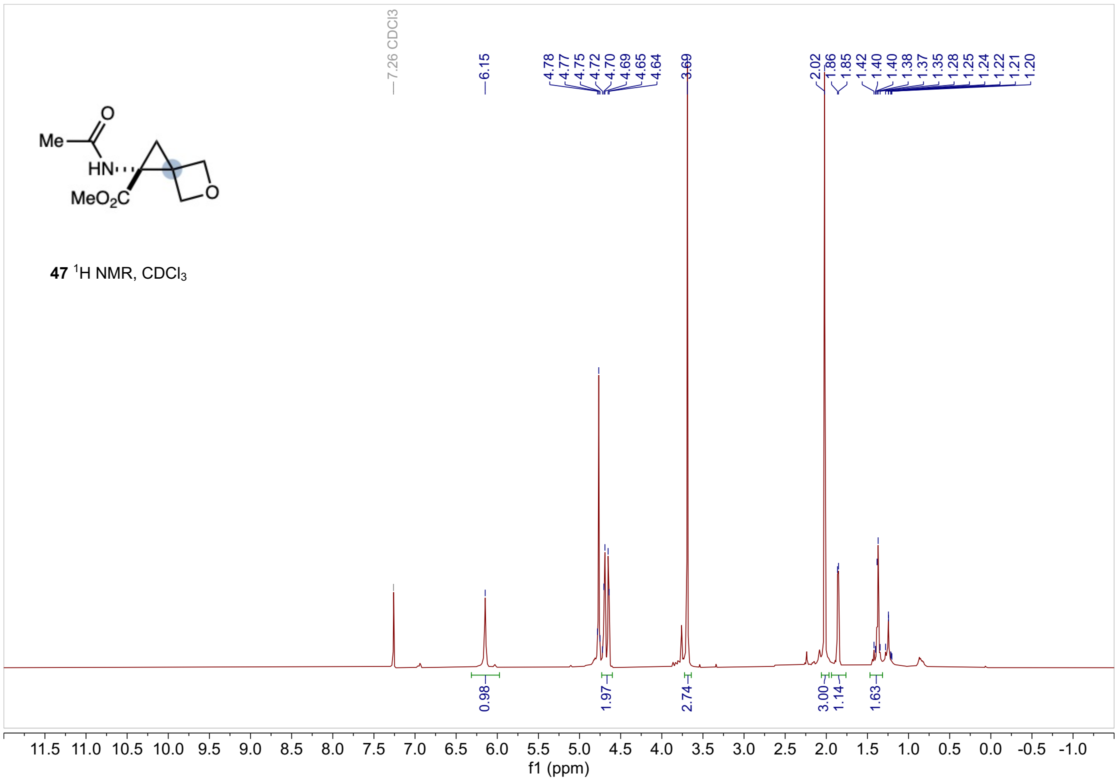

Me
O
HN
MeO2C
O
47 1H NMR, CDCl3
7.26 CDCl3
6.15
4.78
4.77
4.75
4.72
4.70
4.69
4.65
4.64
3.69
2.02
1.86
1.85
1.42
1.40
1.40
1.38
1.37
1.35
1.28
1.25
1.24
1.22
1.21
1.20
0.98
1.97
2.74
3.00
1.14
1.63
f1 (ppm)

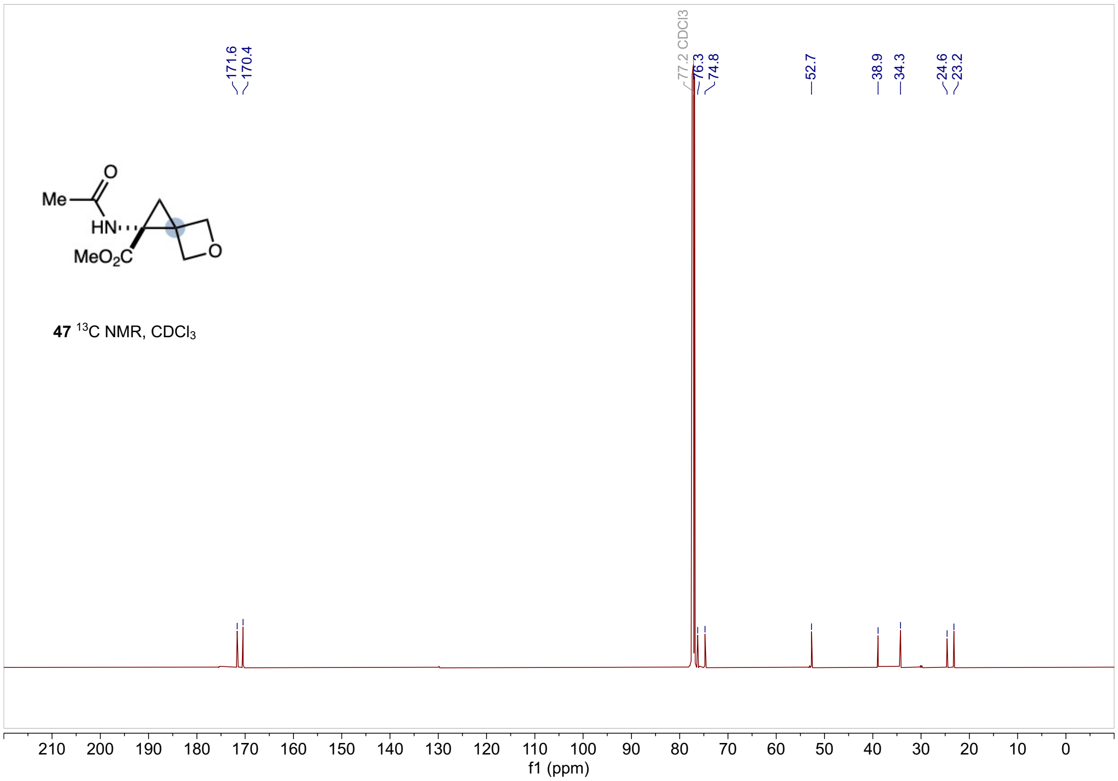

Me
O
HN
MeO2C
O
47 13C NMR, CDCl3
171.6
170.4
77.2 CDCl3
76.3
74.8
52.7
38.9
34.3
24.6
23.2
f1 (ppm)

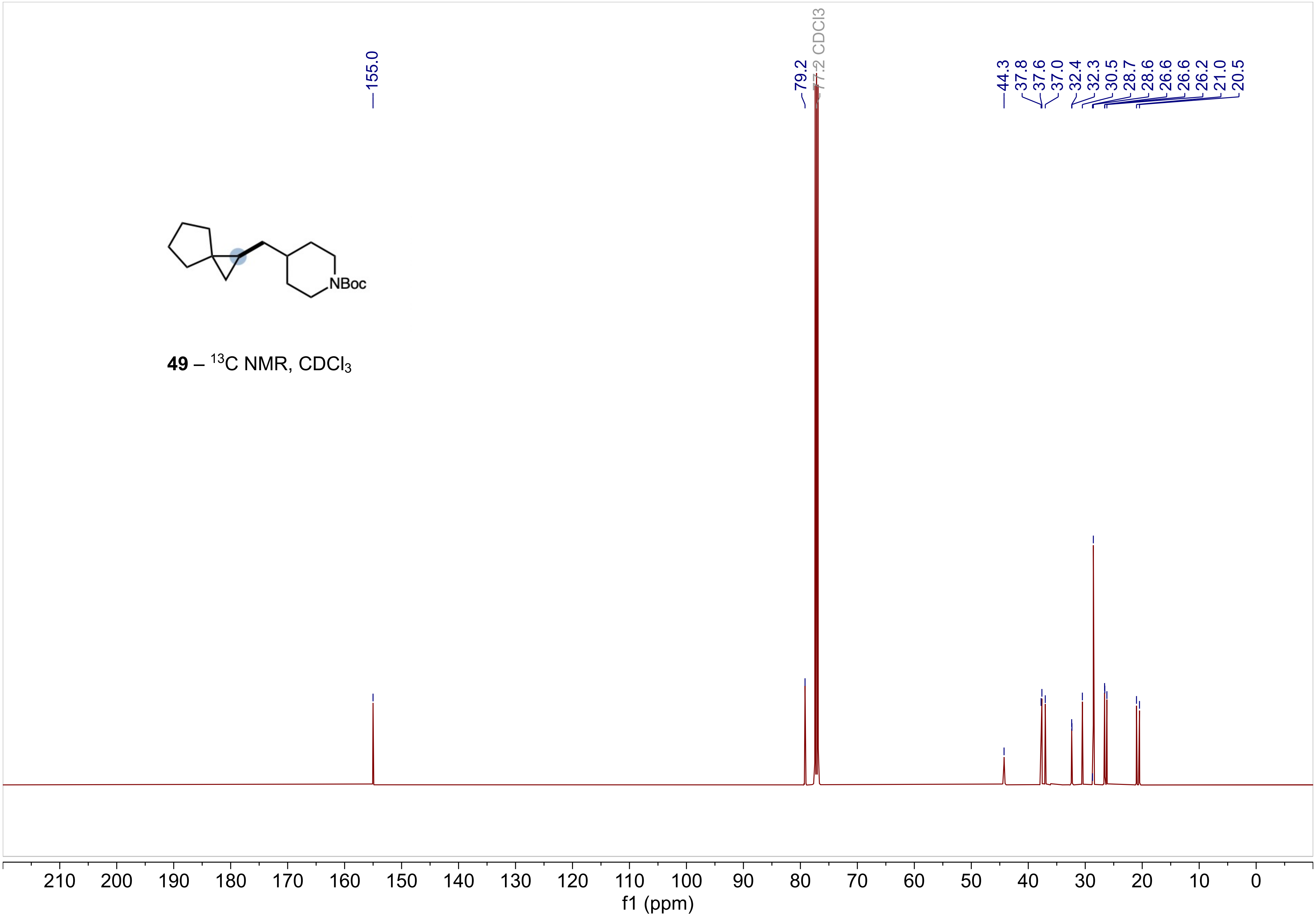
155.0
79.2
77.2 CDCl3
44.3
37.8
37.6
37.0
32.4
32.3
30.5
28.7
28.6
26.6
26.6
26.2
21.0
20.5
NBoc
49 – 13C NMR, CDCl3
210 200 190 180 170 160 150 140 130 120 110 100 90 80 70 60 50 40 30 20 10 0
f1 (ppm)

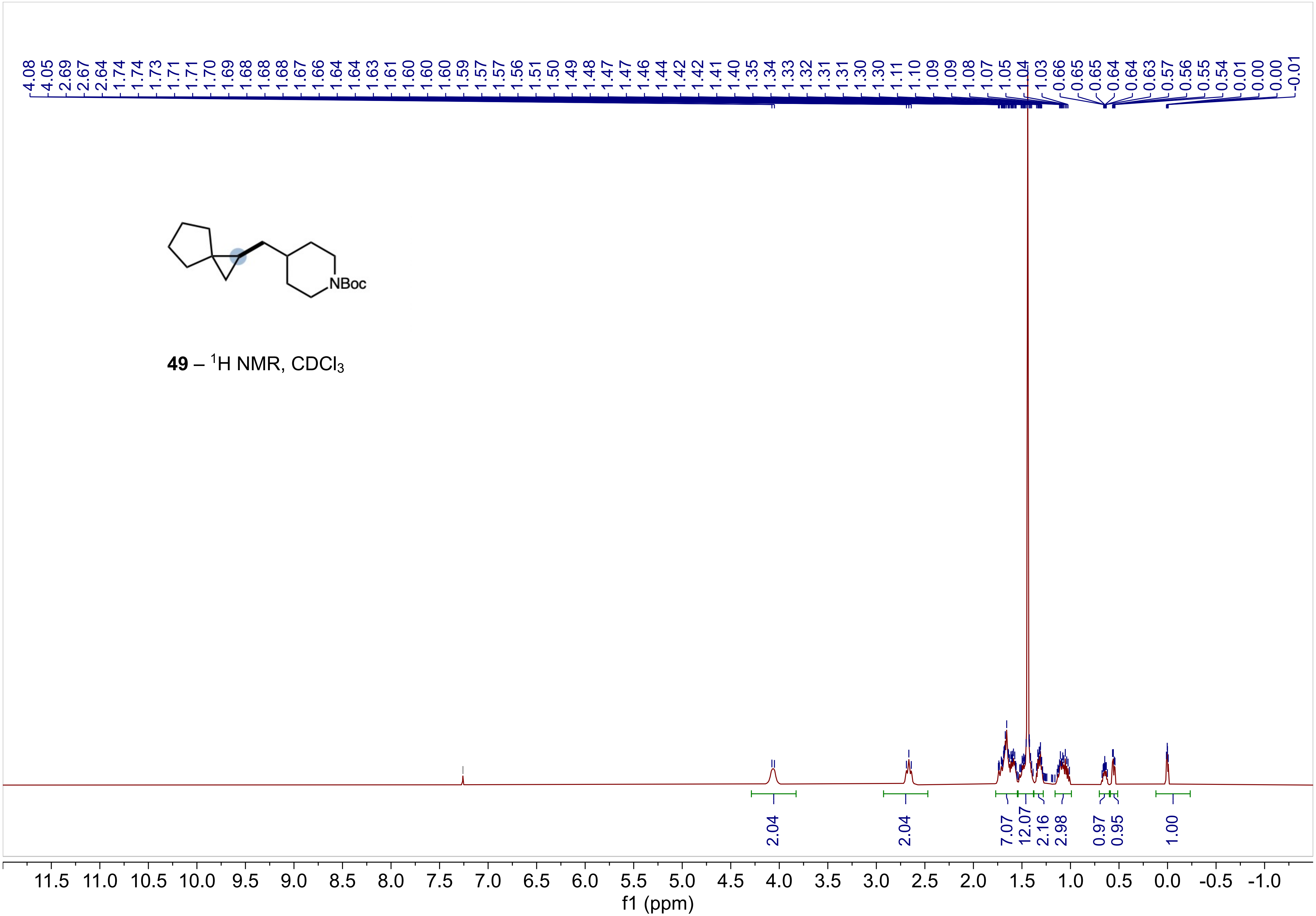

4.08 4.05 2.69 2.67 2.64 1.74 1.74 1.73 1.71 1.71 1.70 1.69 1.68 1.68 1.68 1.67 1.66 1.64 1.64 1.63 1.61 1.60 1.60 1.60 1.59 1.57 1.57 1.56 1.51 1.50 1.49 1.48 1.47 1.47 1.46 1.44 1.42 1.42 1.41 1.40 1.35 1.34 1.33 1.32 1.31 1.31 1.30 1.30 1.11 1.10 1.09 1.09 1.08 1.07 1.05 1.04 1.03 0.66 0.65 0.65 0.64 0.64 0.63 0.57 0.56 0.55 0.54 0.01 0.00 0.00 -0.01
NBoc
49 – ^{1}H NMR, CDCl$_3$
2.04
2.04
7.07
12.07
2.16
2.98
0.97
0.95
1.00
11.5 11.0 10.5 10.0 9.5 9.0 8.5 8.0 7.5 7.0 6.5 6.0 5.5 5.0 4.5 4.0 3.5 3.0 2.5 2.0 1.5 1.0 0.5 0.0 -0.5 -1.0
f1 (ppm)

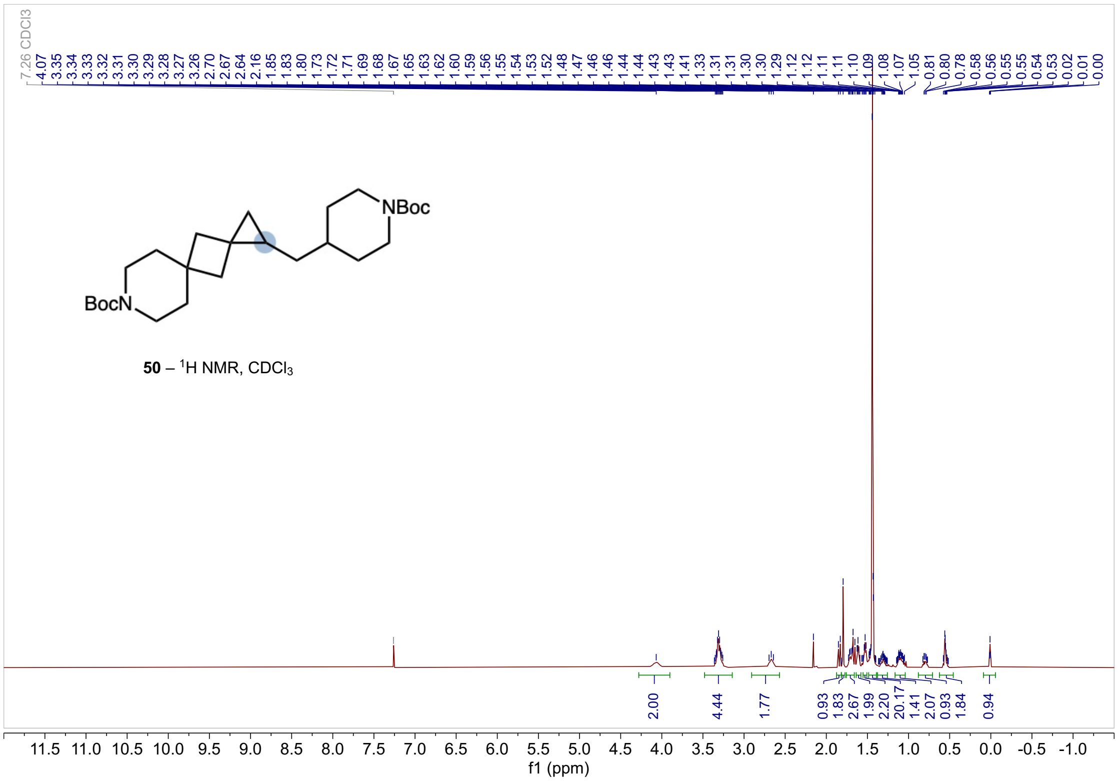
7.26 CDCl3
4.07 3.35 3.34 3.33 3.32 3.31 3.30 3.29 3.28 3.27 3.26 2.70 2.67 2.64 2.16 1.85 1.83 1.80 1.73 1.72 1.71 1.69 1.68 1.67 1.65 1.63 1.62 1.60 1.59 1.56 1.55 1.54 1.53 1.52 1.48 1.47 1.46 1.46 1.44 1.44 1.43 1.43 1.41 1.33 1.31 1.31 1.30 1.30 1.29 1.12 1.11 1.11 1.10 1.09 1.08 1.07 1.05 0.81 0.80 0.78 0.58 0.56 0.55 0.55 0.54 0.53 0.02 0.01 0.00
NBoc
BocN
50 – ¹H NMR, CDCl3
2.00 4.44 1.77 0.93 1.83 2.67 1.99 2.20 20.17 1.41 2.07 0.93 1.84 0.94
f1 (ppm)

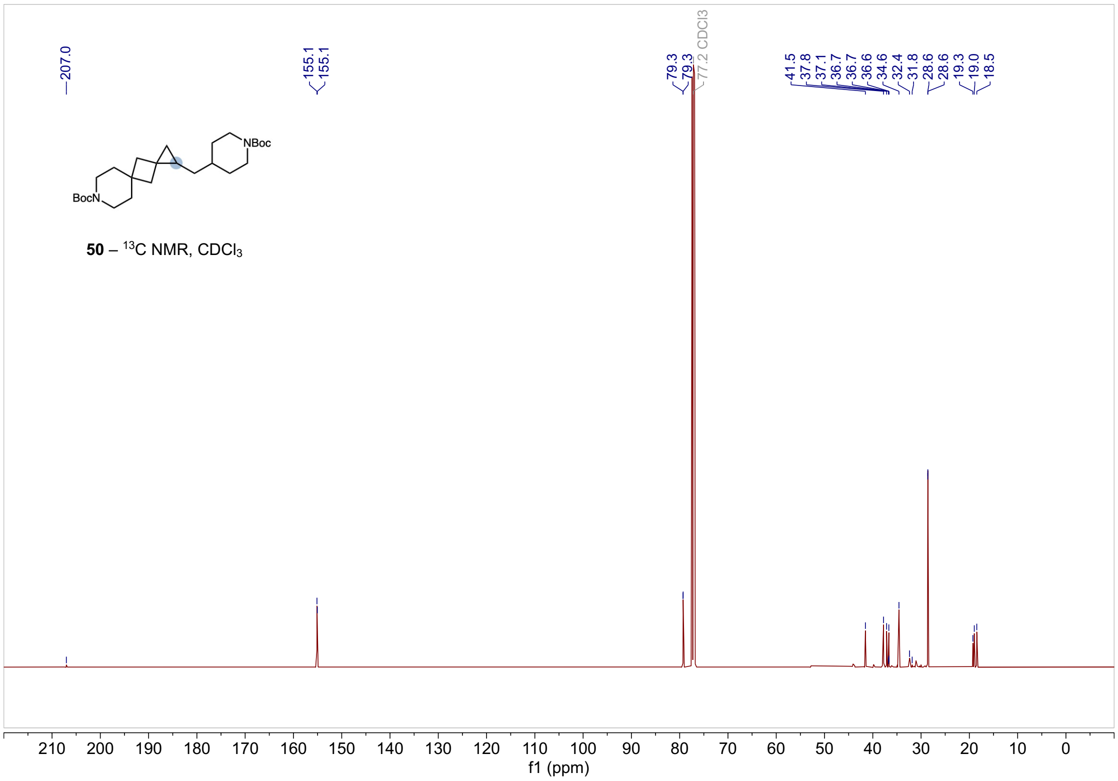
207.0
155.1 155.1
79.3 79.3 77.2 CDCl3
41.5 37.8 37.1 36.7 36.6 34.6 32.4 31.8 28.6 19.3 19.0 18.5
NBoc
BocN
50 – ¹³C NMR, CDCl3
f1 (ppm)

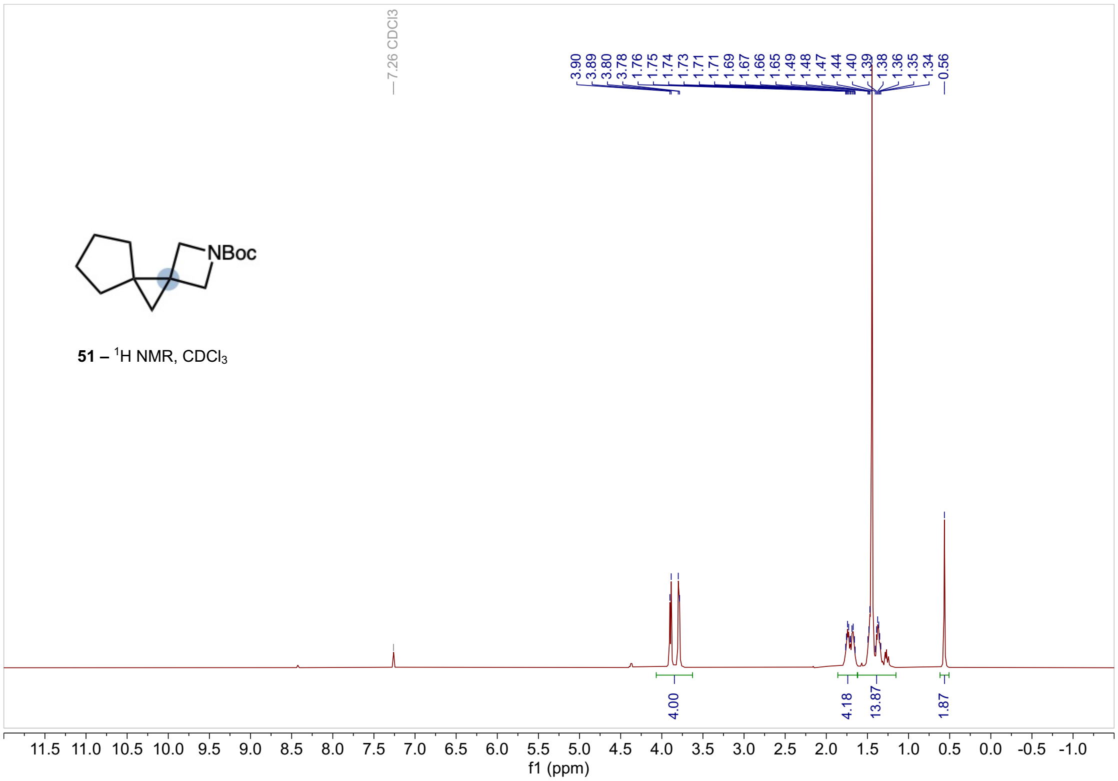
7.26 CDCl3
3.90
3.89
3.80
3.78
1.76
1.75
1.74
1.73
1.71
1.71
1.69
1.67
1.66
1.65
1.49
1.48
1.47
1.44
1.40
1.39
1.38
1.36
1.35
1.34
0.56
NBoc
51 – ^{1}H NMR, $CDCl_3$
4.00
4.18
13.87
1.87
11.5 11.0 10.5 10.0 9.5 9.0 8.5 8.0 7.5 7.0 6.5 6.0 5.5 5.0 4.5 4.0 3.5 3.0 2.5 2.0 1.5 1.0 0.5 0.0 -0.5 -1.0
f1 (ppm)

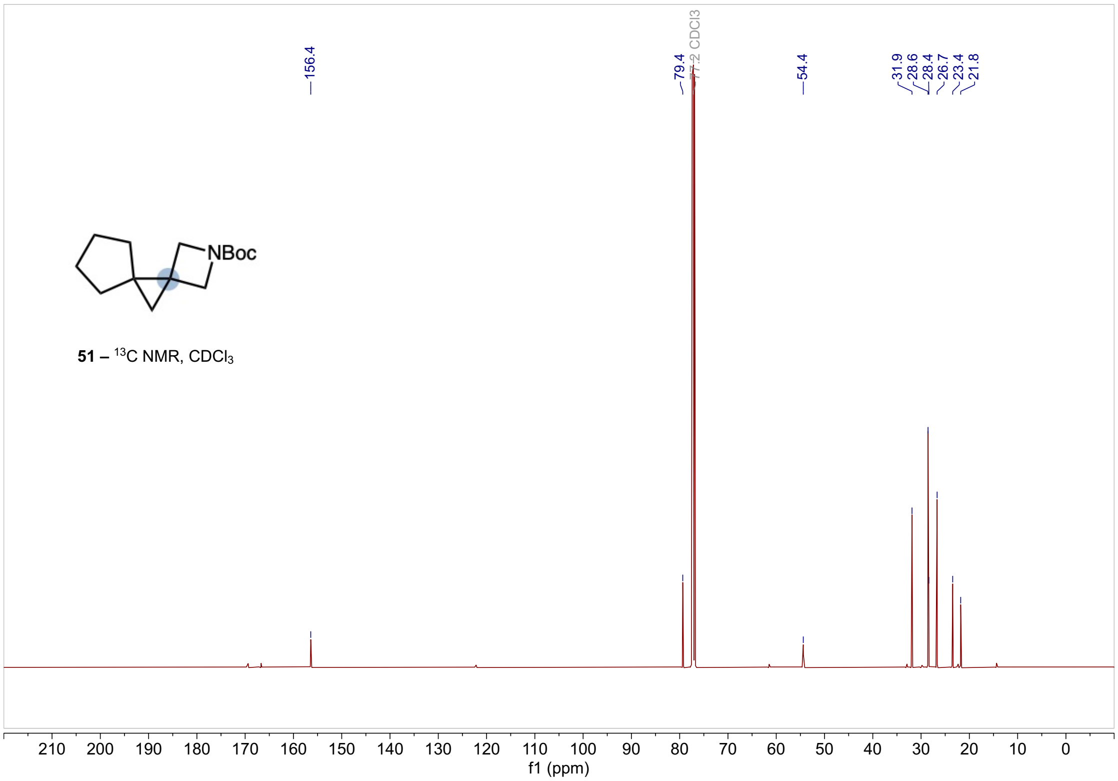
156.4
79.4
77.2 CDCl3
54.4
31.9
28.6
28.4
26.7
23.4
21.8
NBoc
51 – ^{13}C NMR, $CDCl_3$
210 200 190 180 170 160 150 140 130 120 110 100 90 80 70 60 50 40 30 20 10 0
f1 (ppm)

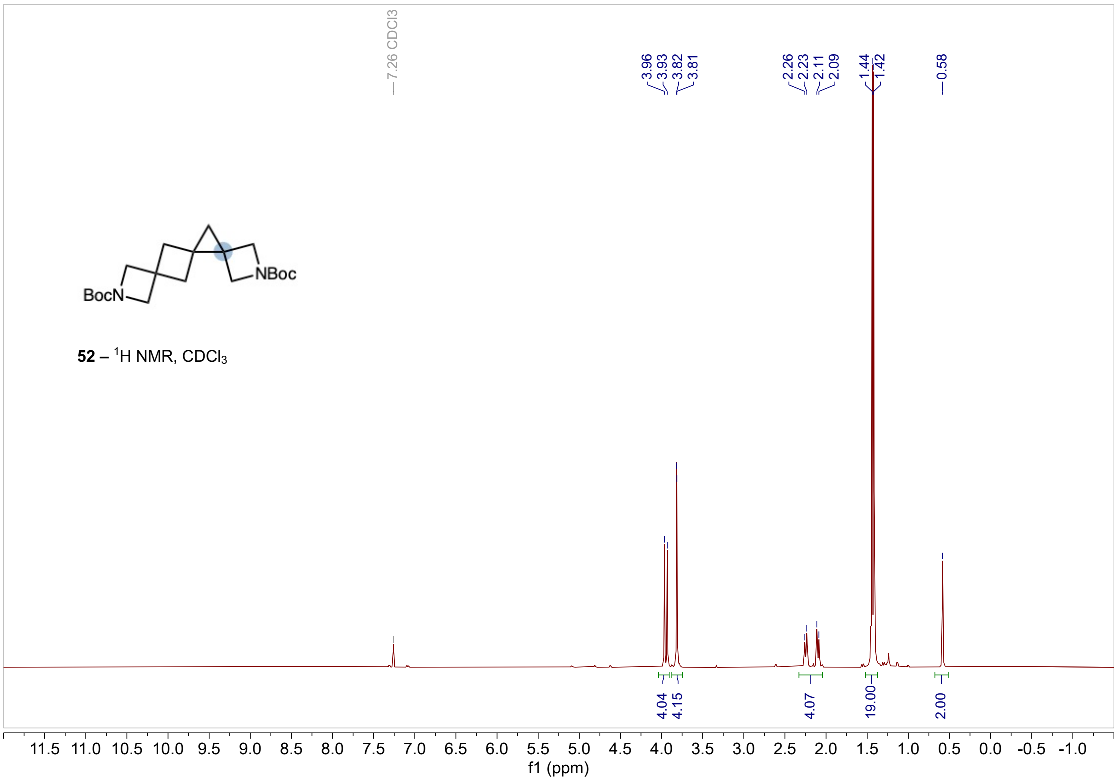
7.26 CDCl3
3.96
3.93
3.82
3.81
2.26
2.23
2.11
2.09
1.44
1.42
0.58
NBoc
BocN
52 – ^{1}H NMR, $CDCl_3$
4.04
4.15
4.07
19.00
2.00
11.5 11.0 10.5 10.0 9.5 9.0 8.5 8.0 7.5 7.0 6.5 6.0 5.5 5.0 4.5 4.0 3.5 3.0 2.5 2.0 1.5 1.0 0.5 0.0 -0.5 -1.0
f1 (ppm)

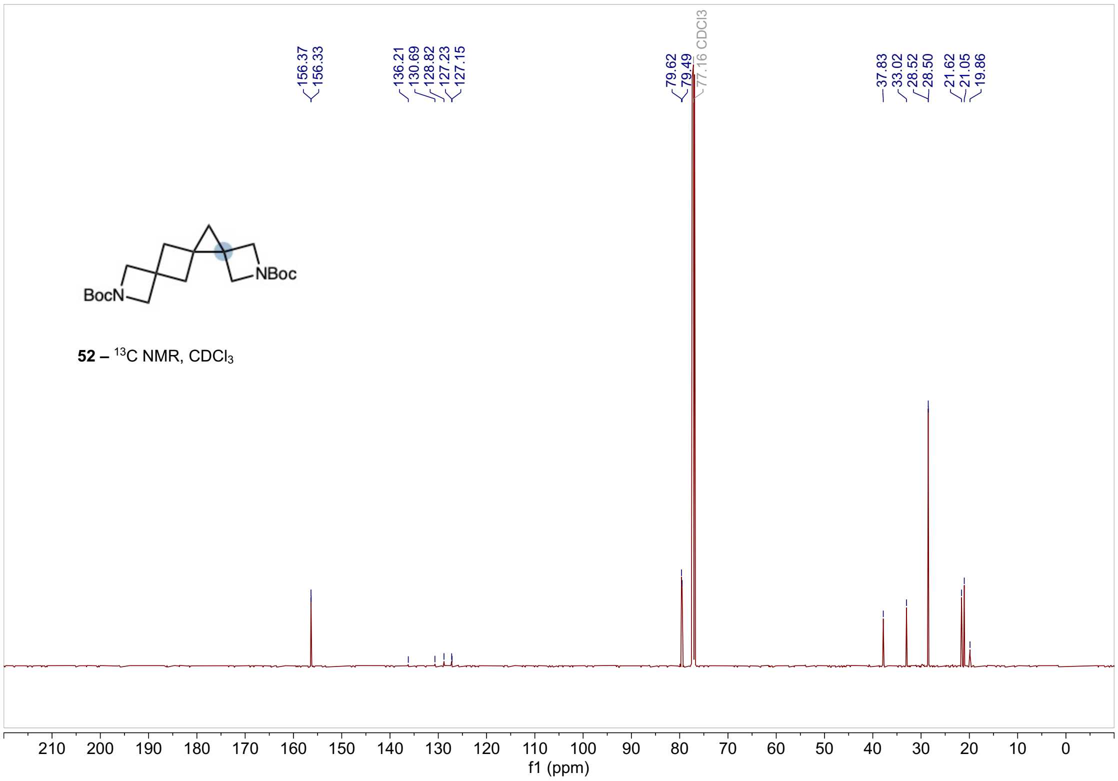
156.37
156.33
136.21
130.69
128.82
127.23
127.15
79.62
79.49
77.16 CDCl3
37.83
33.02
28.52
28.50
21.62
21.05
19.86
NBoc
BocN
52 – ^{13}C NMR, $CDCl_3$
210 200 190 180 170 160 150 140 130 120 110 100 90 80 70 60 50 40 30 20 10 0
f1 (ppm)

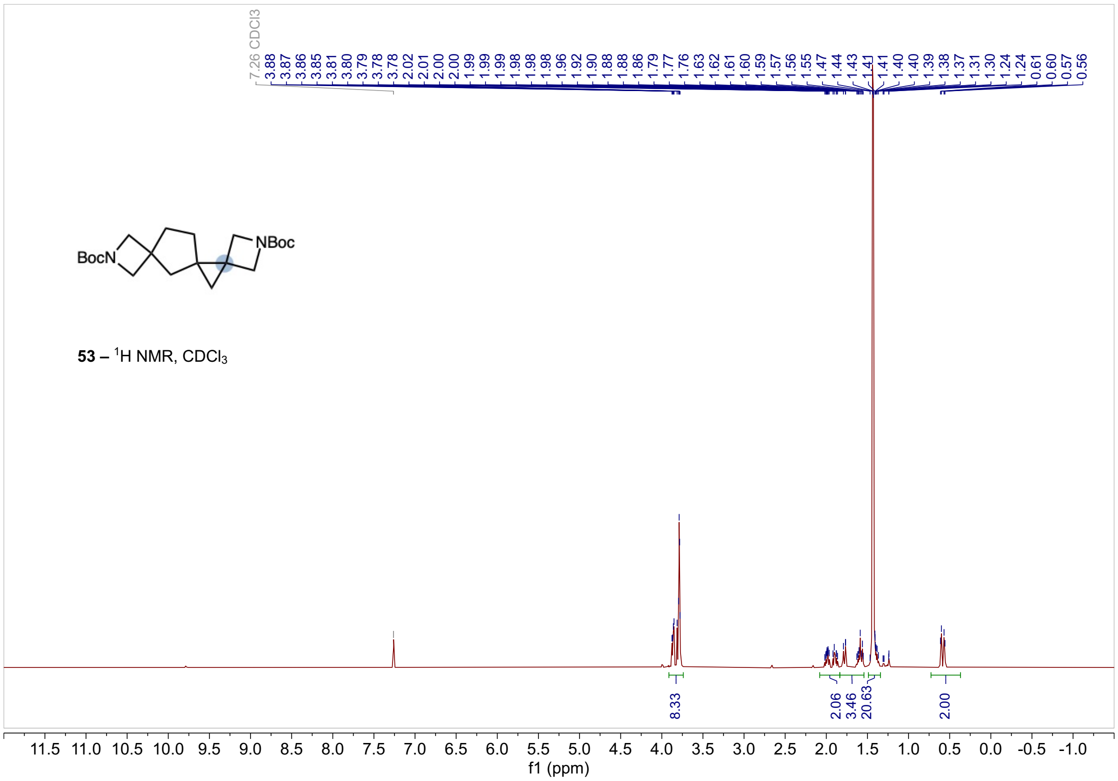

BocN
NBoc
53 – ¹H NMR, CDCl₃
7.26 CDCl3
3.88 3.87 3.86 3.85 3.81 3.80 3.79 3.78 3.78 2.02 2.01 2.00 1.99 1.99 1.98 1.98 1.98 1.96 1.92 1.90 1.88 1.88 1.86 1.79 1.77 1.76 1.63 1.62 1.61 1.60 1.59 1.57 1.56 1.55 1.47 1.44 1.43 1.41 1.41 1.40 1.39 1.38 1.37 1.31 1.30 1.24 1.24 0.61 0.60 0.57 0.56
8.33
2.06
3.46
20.63
2.00
f1 (ppm)

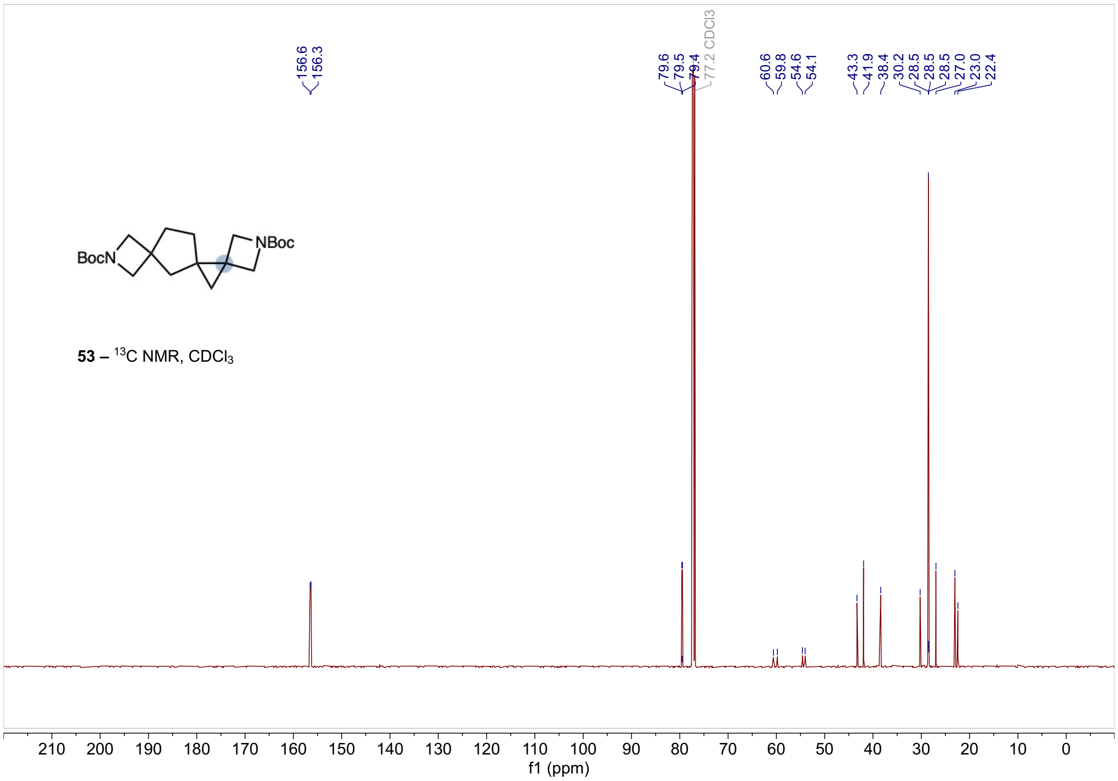

BocN
NBoc
53 – ¹³C NMR, CDCl₃
156.6
156.3
79.6
79.5
79.4
77.2 CDCl3
60.6
59.8
54.6
54.1
43.3
41.9
38.4
30.2
28.5
28.5
28.5
27.0
23.0
22.4
f1 (ppm)

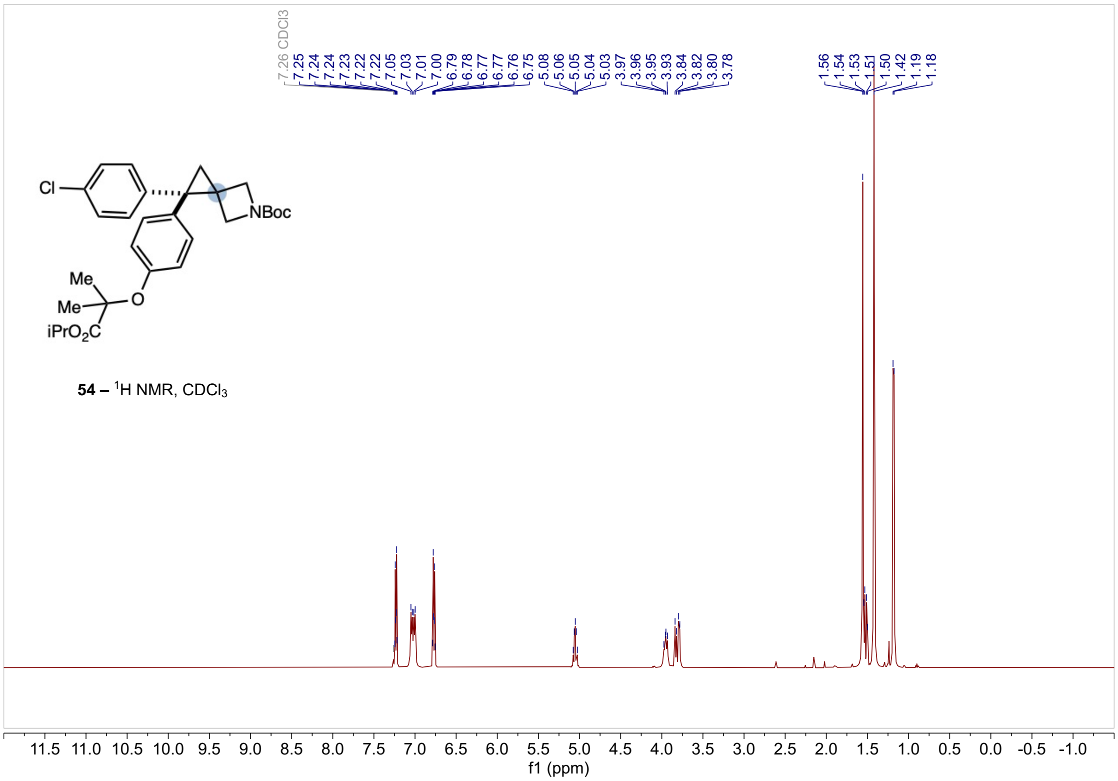

7.26 CDCl3
7.25
7.24
7.24
7.23
7.22
7.22
7.05
7.03
7.01
7.00
6.79
6.78
6.77
6.77
6.76
6.75
5.08
5.06
5.05
5.04
5.03
3.97
3.96
3.95
3.93
3.84
3.82
3.80
3.78
1.56
1.54
1.53
1.51
1.50
1.42
1.19
1.18
Cl
NBoc
Me
Me
O
iPrO2C
54 – 1H NMR, CDCl3
11.5 11.0 10.5 10.0 9.5 9.0 8.5 8.0 7.5 7.0 6.5 6.0 5.5 5.0 4.5 4.0 3.5 3.0 2.5 2.0 1.5 1.0 0.5 0.0 -0.5 -1.0
f1 (ppm)

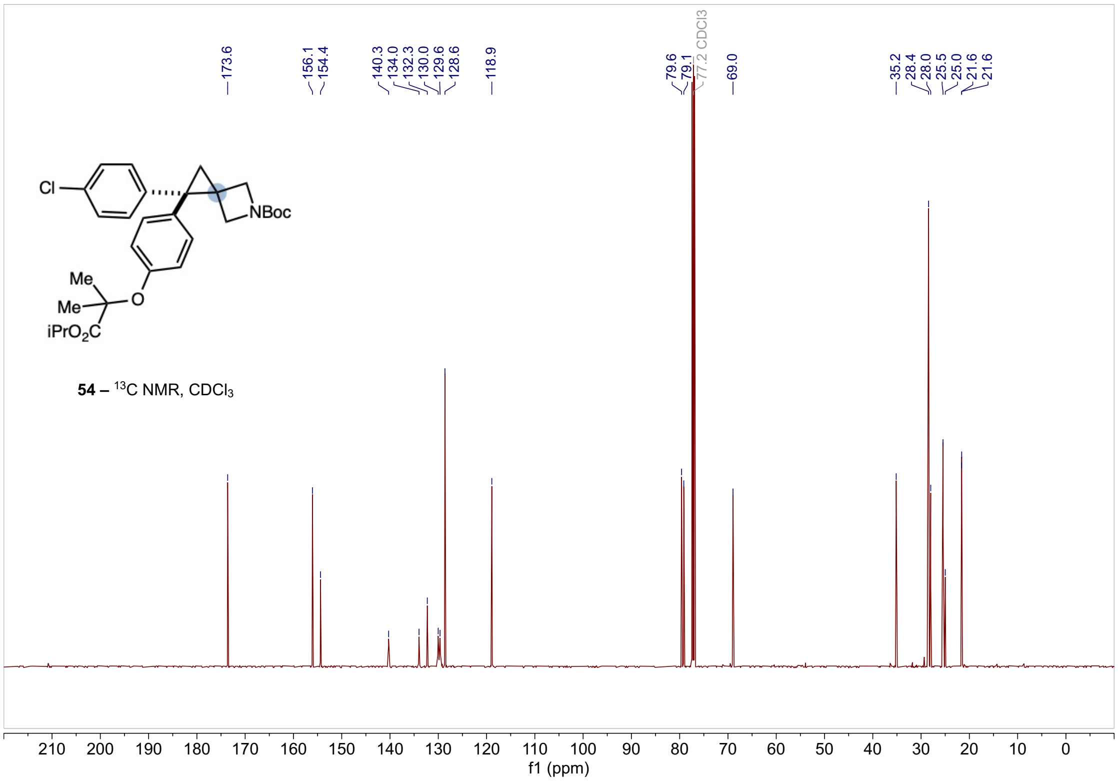

173.6
156.1
154.4
140.3
134.0
132.3
130.0
129.6
128.6
118.9
79.6
79.1
77.2 CDCl3
69.0
35.2
28.4
28.0
25.5
25.0
21.6
21.6
Cl
NBoc
Me
Me
O
iPrO2C
54 – 13C NMR, CDCl3
210 200 190 180 170 160 150 140 130 120 110 100 90 80 70 60 50 40 30 20 10 0
f1 (ppm)

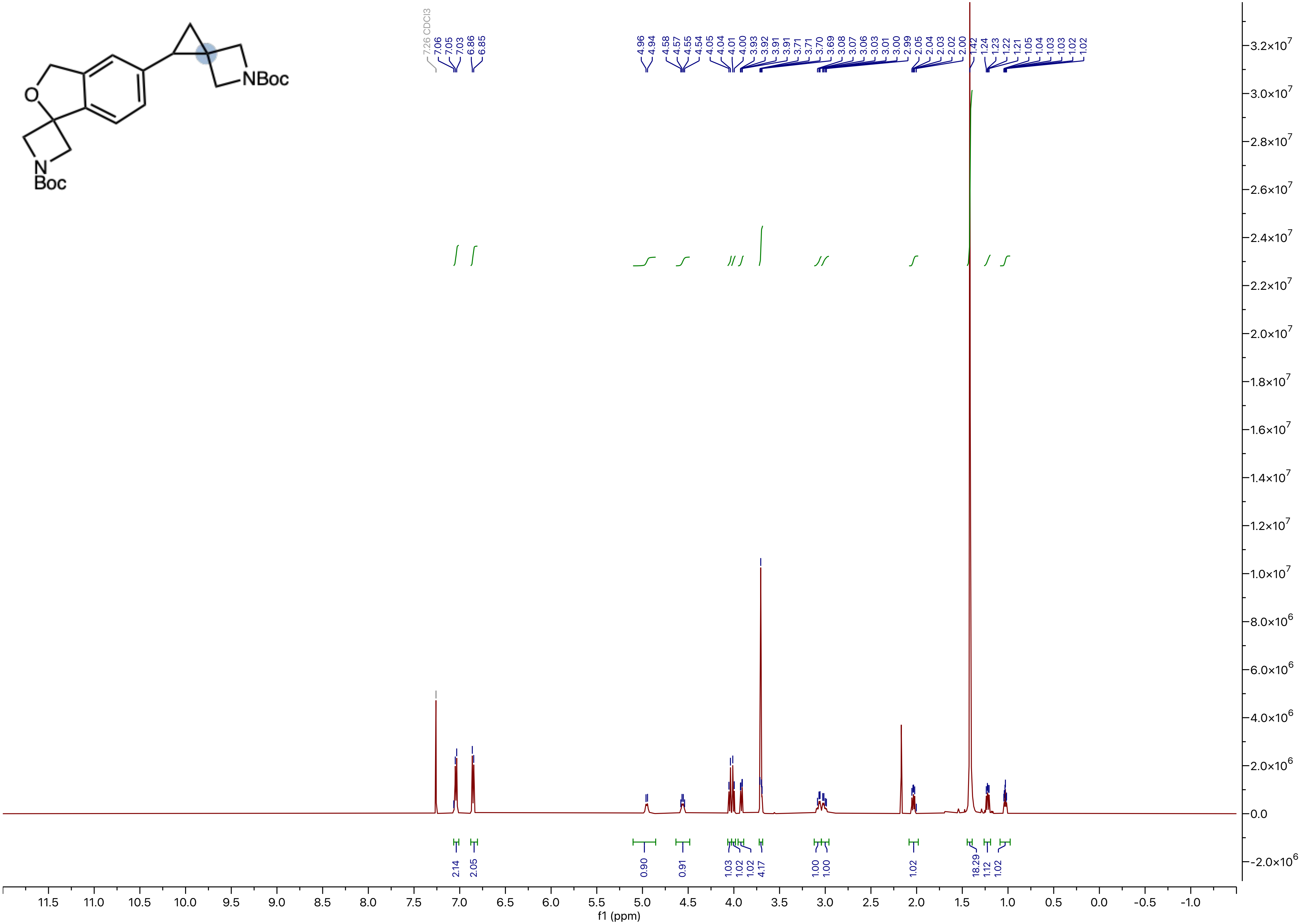
NBoc
O
N
Boc
7.26 CDCl3
7.06
7.05
7.03
6.86
6.85
4.96
4.94
4.58
4.57
4.55
4.54
4.05
4.04
4.01
4.00
3.93
3.92
3.91
3.91
3.71
3.71
3.70
3.69
3.08
3.07
3.06
3.03
3.01
3.00
2.99
2.05
2.04
2.03
2.02
2.00
1.42
1.24
1.23
1.22
1.21
1.05
1.04
1.03
1.02
1.02
2.14
2.05
0.90
0.91
1.03
1.02
1.02
4.17
1.00
1.00
1.02
18.29
1.12
1.02
f1 (ppm)

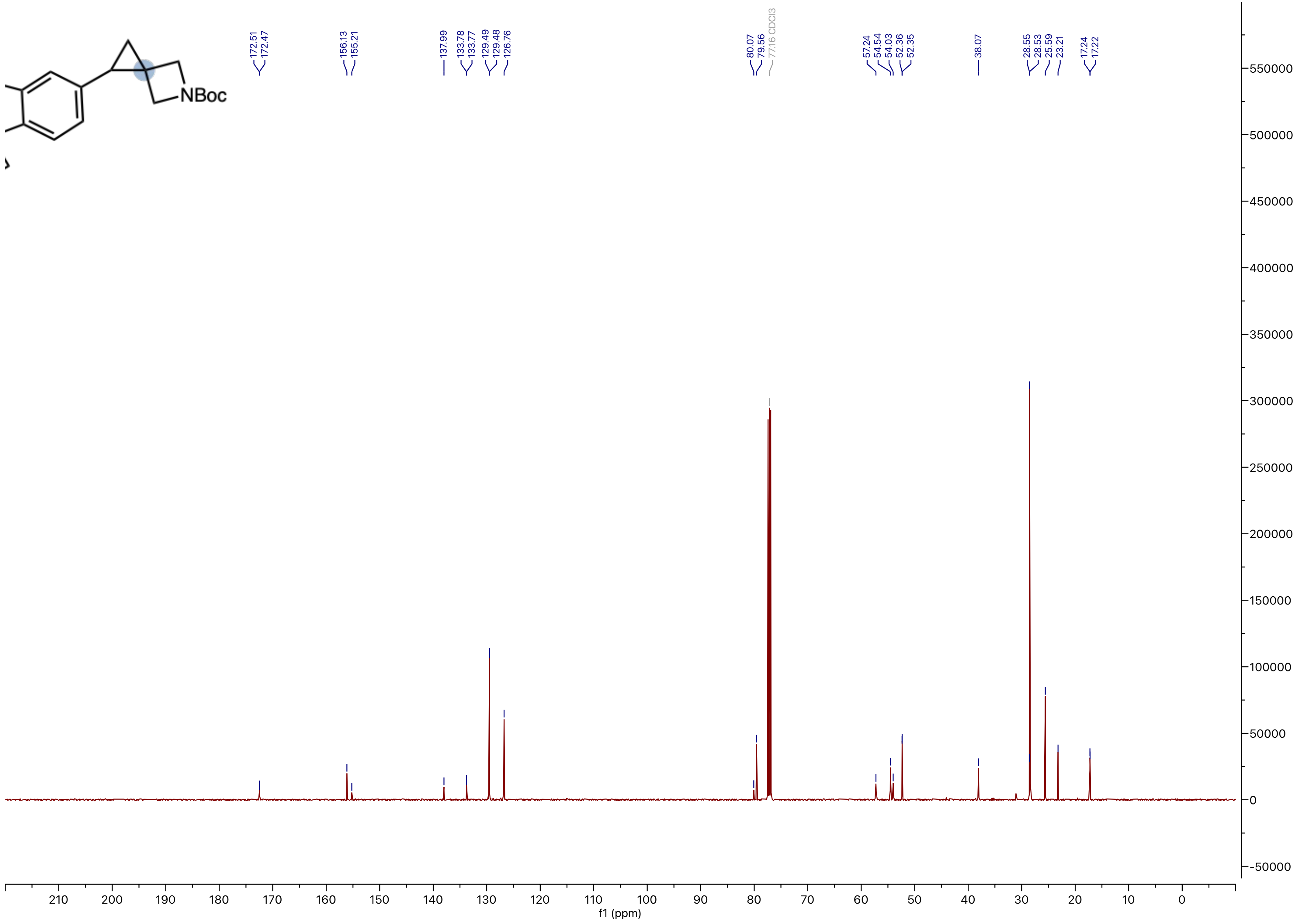

NBoc
172.51
172.47
156.13
155.21
137.99
133.78
133.77
129.49
129.48
126.76
80.07
79.56
77.16 CDCl3
57.24
54.54
54.03
52.36
52.35
38.07
28.55
28.53
25.59
23.21
17.24
17.22
550000
500000
450000
400000
350000
300000
250000
200000
150000
100000
50000
0
-50000
210
200
190
180
170
160
150
140
130
120
110
100
90
80
70
60
50
40
30
20
10
0
f1 (ppm)

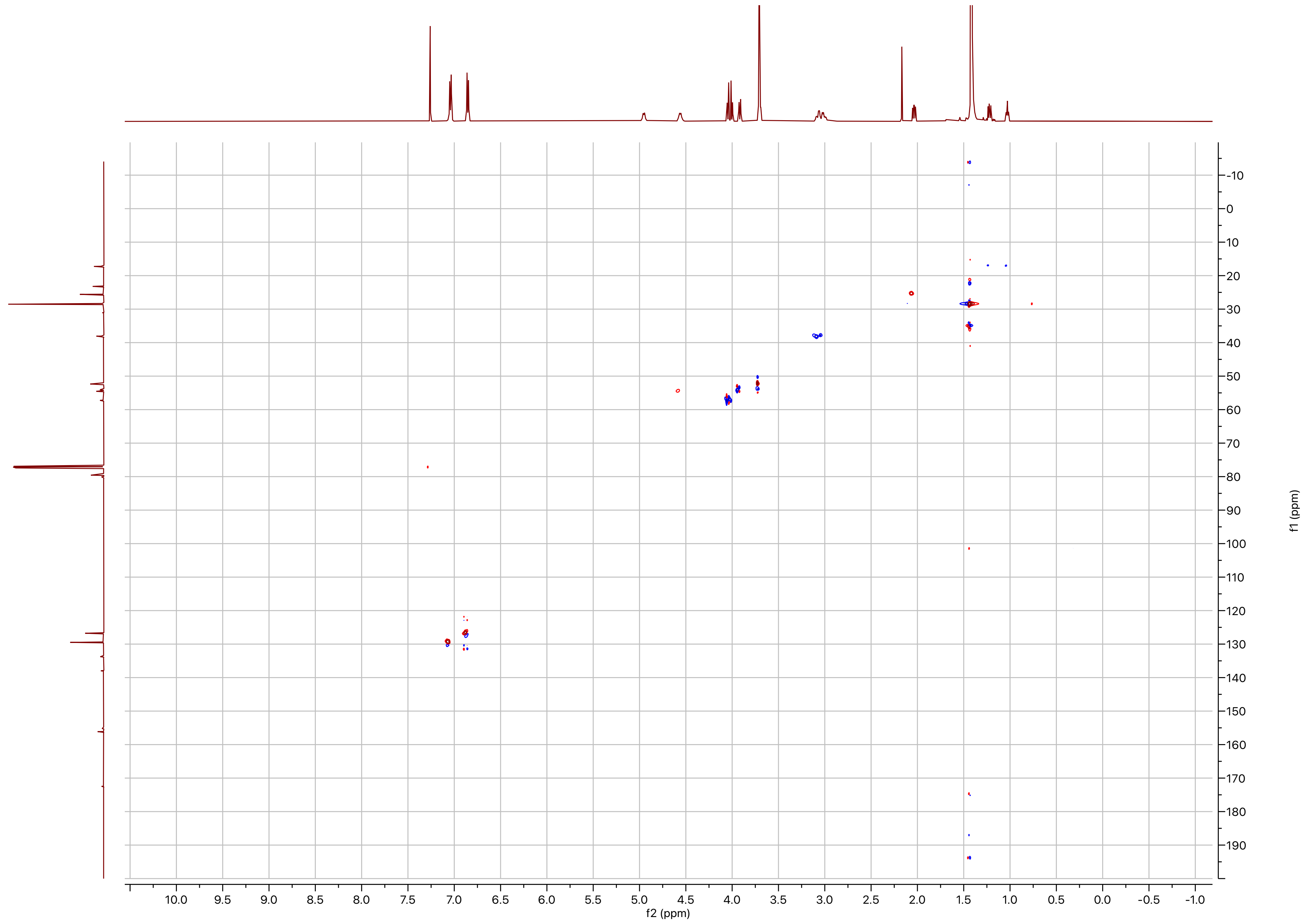

f2 (ppm)
f1 (ppm)

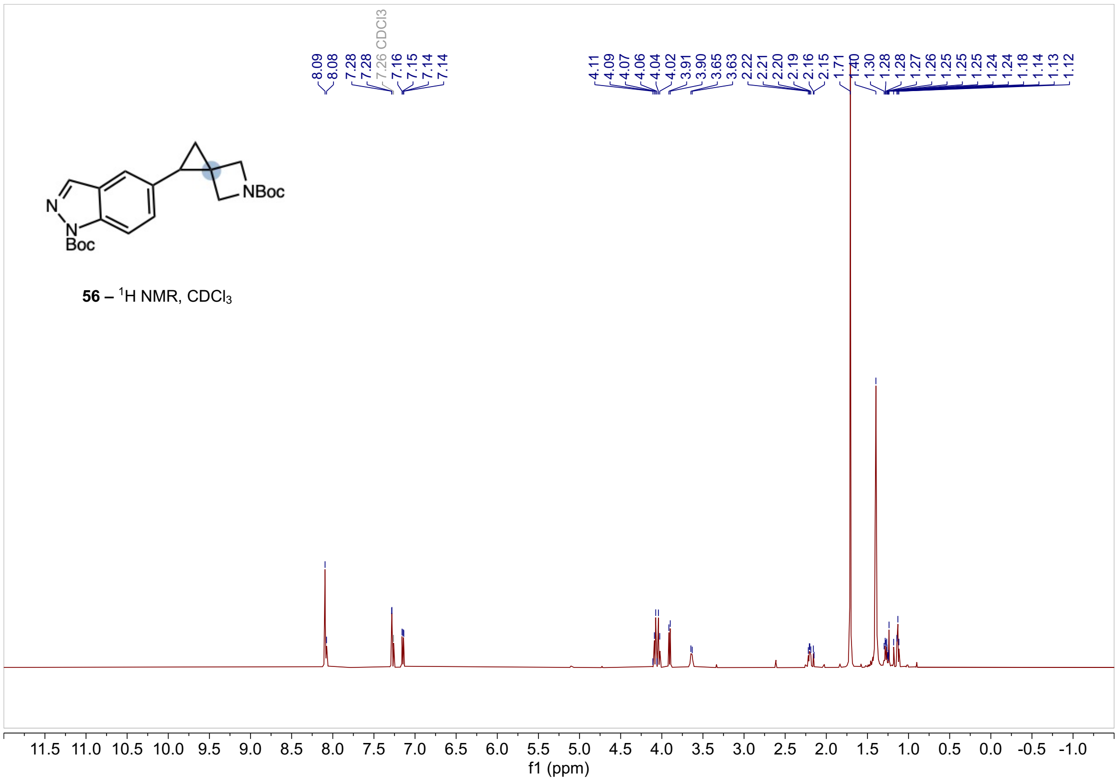

N
N
Boc
NBoc
56 – ¹H NMR, CDCl₃
8.09
8.08
7.28
7.28
7.26 CDCl3
7.16
7.15
7.14
7.14
4.11
4.09
4.07
4.06
4.04
4.02
3.91
3.90
3.65
3.63
2.22
2.21
2.20
2.19
2.16
2.15
1.71
1.40
1.30
1.28
1.28
1.27
1.26
1.25
1.25
1.24
1.24
1.18
1.14
1.13
1.12
11.5 11.0 10.5 10.0 9.5 9.0 8.5 8.0 7.5 7.0 6.5 6.0 5.5 5.0 4.5 4.0 3.5 3.0 2.5 2.0 1.5 1.0 0.5 0.0 -0.5 -1.0
f1 (ppm)

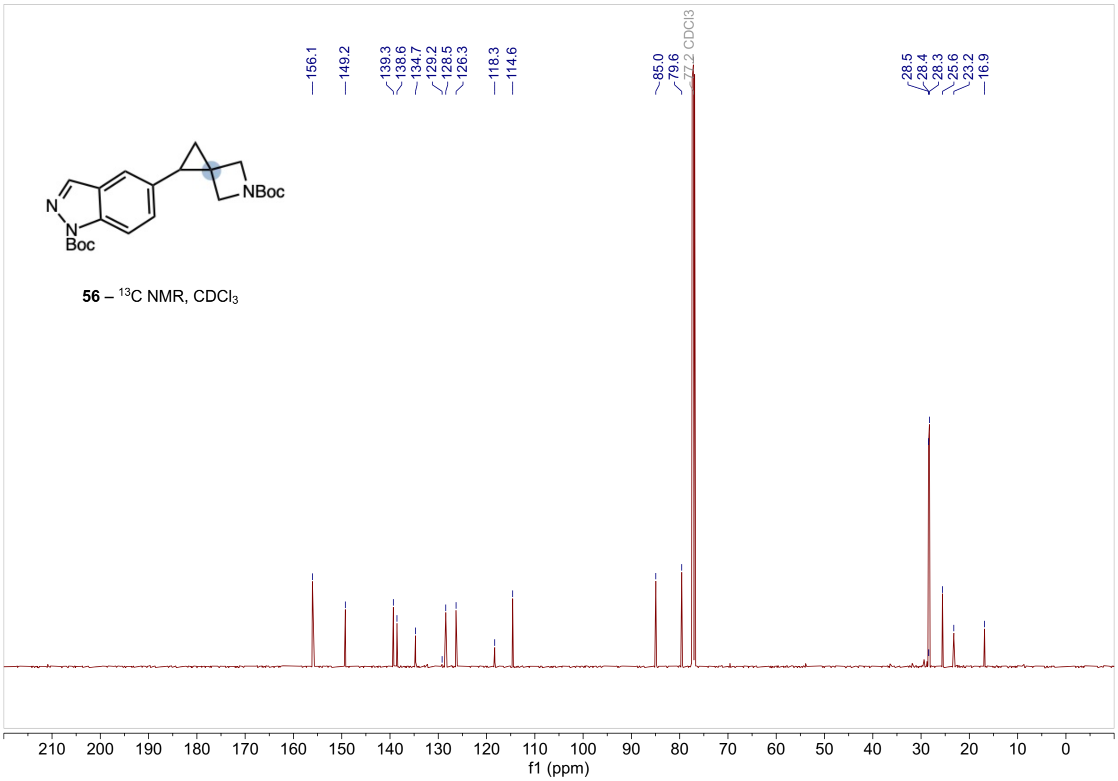

N
N
Boc
NBoc
56 – ¹³C NMR, CDCl₃
156.1
149.2
139.3
138.6
134.7
129.2
128.5
126.3
118.3
114.6
85.0
79.6
77.2 CDCl3
28.5
28.4
28.3
25.6
23.2
16.9
210 200 190 180 170 160 150 140 130 120 110 100 90 80 70 60 50 40 30 20 10 0
f1 (ppm)

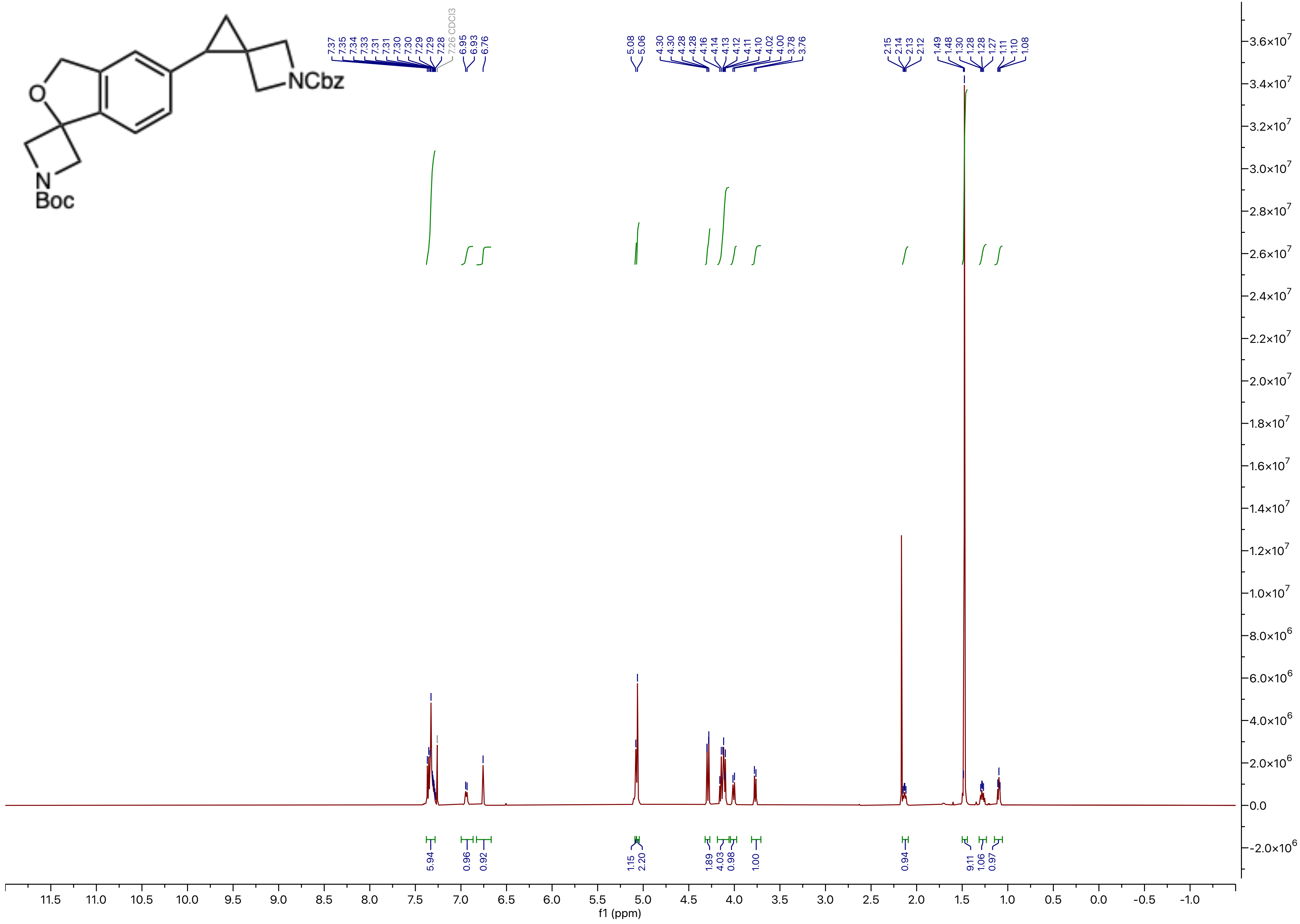

O
NCbz
N
Boc
7.37
7.35
7.34
7.33
7.31
7.31
7.30
7.29
7.29
7.28
7.26 CDCl3
6.95
6.93
6.76
5.08
5.06
4.30
4.30
4.28
4.28
4.16
4.14
4.13
4.12
4.11
4.10
4.02
4.00
3.78
3.76
2.15
2.14
2.13
2.12
1.49
1.48
1.30
1.28
1.28
1.27
1.11
1.10
1.08
5.94
0.96
0.92
1.15
2.20
1.89
4.03
0.98
1.00
0.94
9.11
1.06
0.97
f1 (ppm)

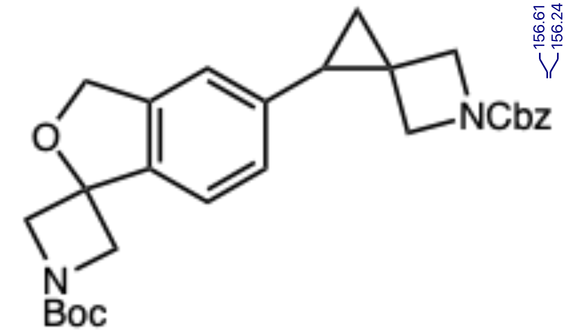
NCbz
O
N
Boc

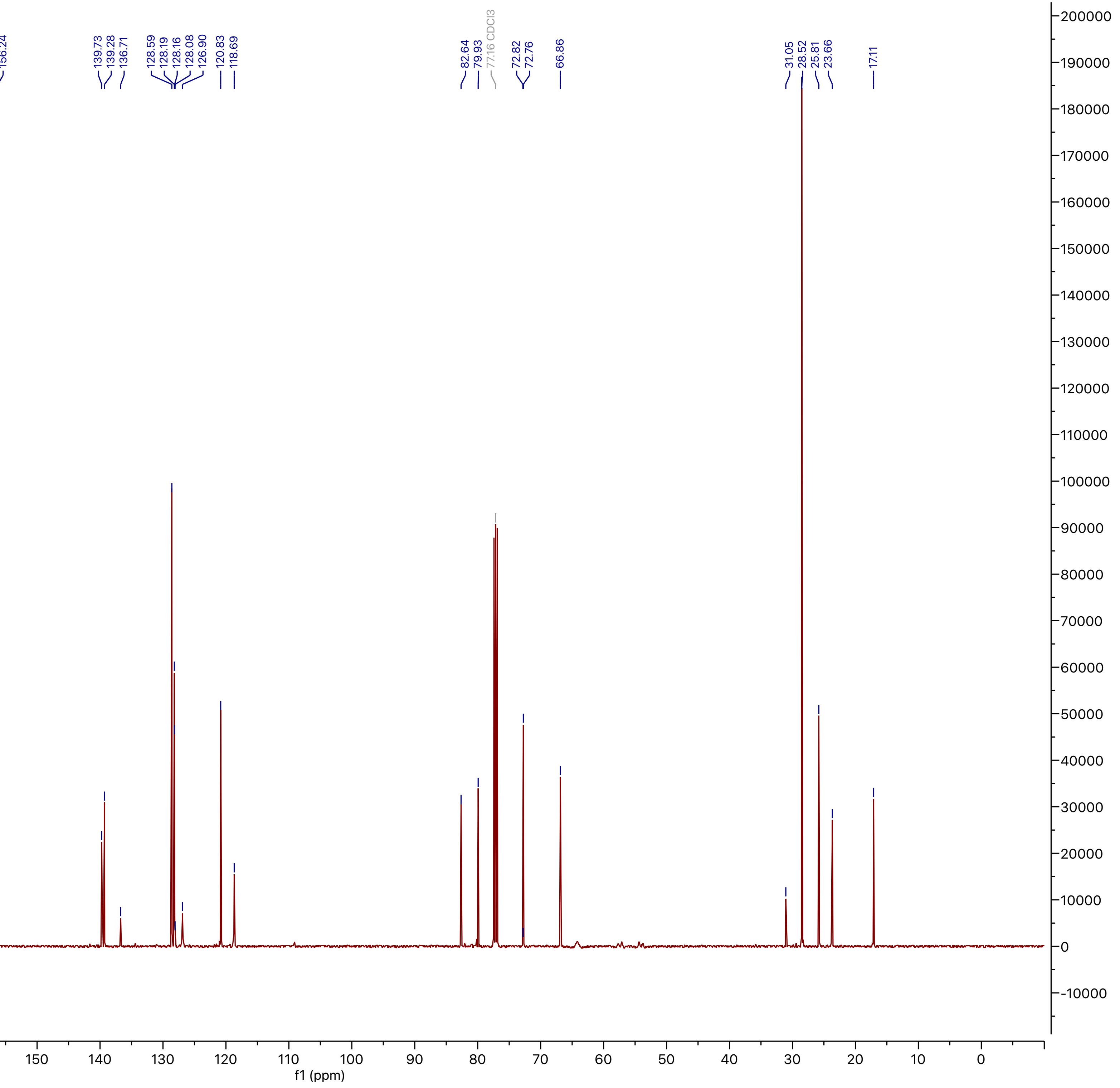
156.61
156.24
139.73
139.28
136.71
128.59
128.19
128.16
128.08
126.90
120.83
118.69
82.64
79.93
77.16 CDCl3
72.82
72.76
66.86
31.05
28.52
25.81
23.66
17.11
f1 (ppm)

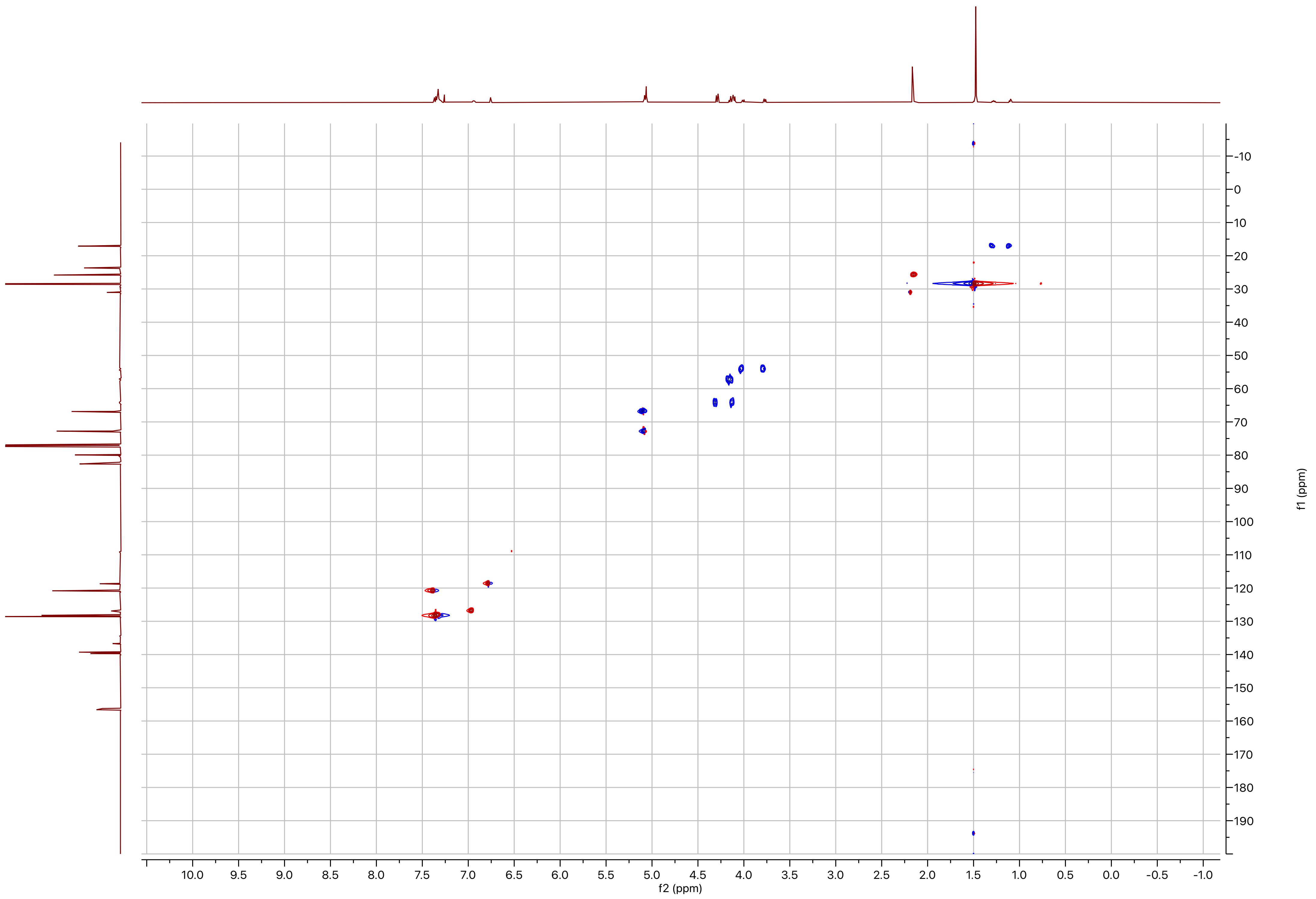
f2 (ppm)
f1 (ppm)

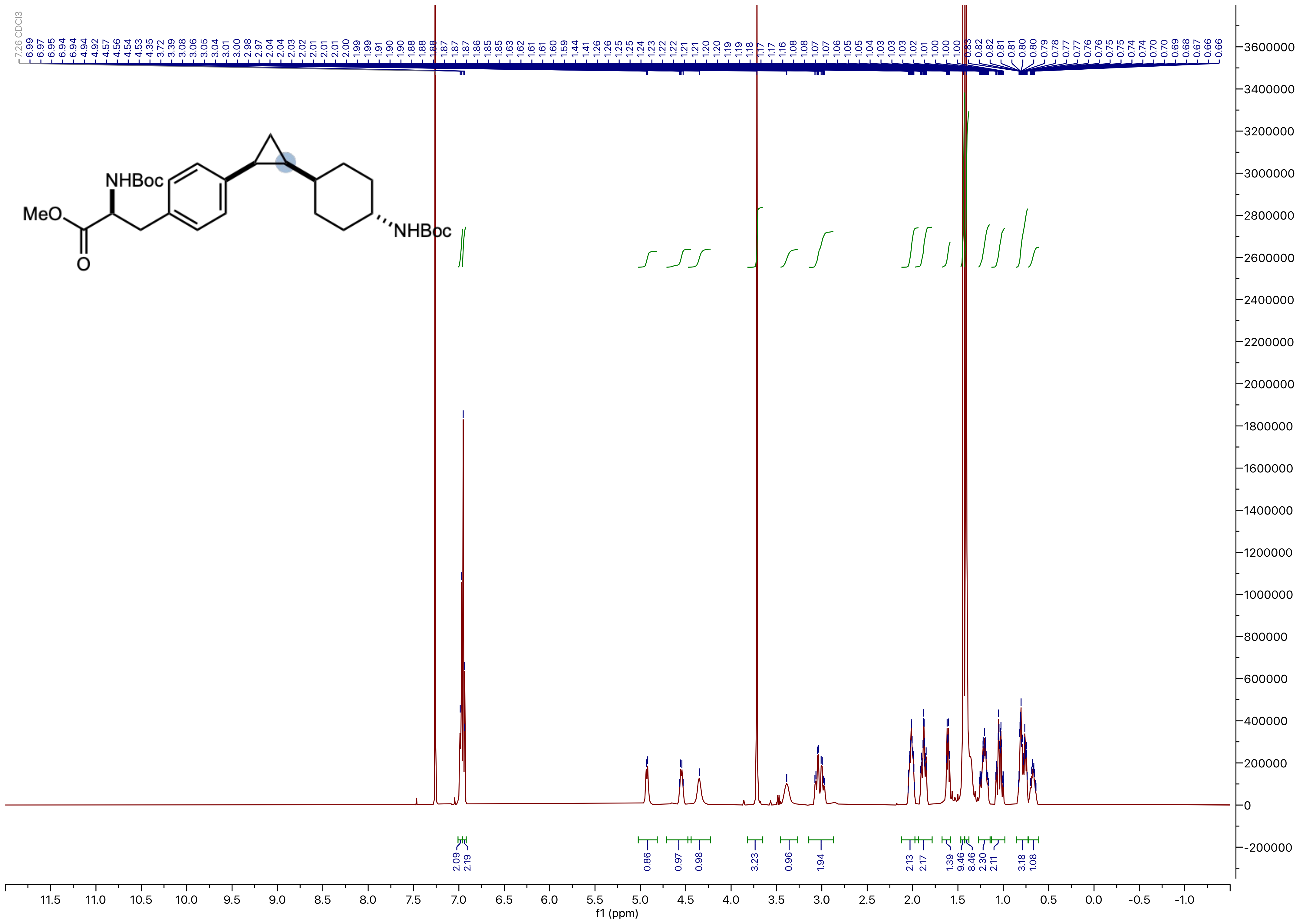
7.26 CDCl3
6.99 6.97 6.95 6.94 6.94 4.94 4.92 4.57 4.56 4.54 4.53 4.35 3.72 3.39 3.08 3.06 3.05 3.04 3.01 3.00 2.98 2.97 2.04 2.04 2.03 2.02 2.01 2.01 2.00 1.99 1.99 1.91 1.90 1.90 1.88 1.88 1.88 1.87 1.87 1.86 1.85 1.85 1.63 1.62 1.61 1.61 1.60 1.59 1.44 1.41 1.26 1.26 1.25 1.25 1.24 1.23 1.22 1.21 1.21 1.20 1.20 1.19 1.19 1.18 1.17 1.17 1.16 1.08 1.08 1.07 1.07 1.06 1.05 1.05 1.04 1.03 1.03 1.02 1.01 1.00 1.00 1.00 0.83 0.82 0.82 0.81 0.81 0.80 0.80 0.79 0.78 0.77 0.77 0.76 0.76 0.75 0.74 0.74 0.70 0.70 0.69 0.68 0.67 0.66 0.66
MeO
NHBoc
O
NHBoc
2.09 2.19 0.86 0.97 0.98 3.23 0.96 1.94 2.13 2.17 1.39 9.46 8.46 2.30 2.11 3.18 1.08
f1 (ppm)

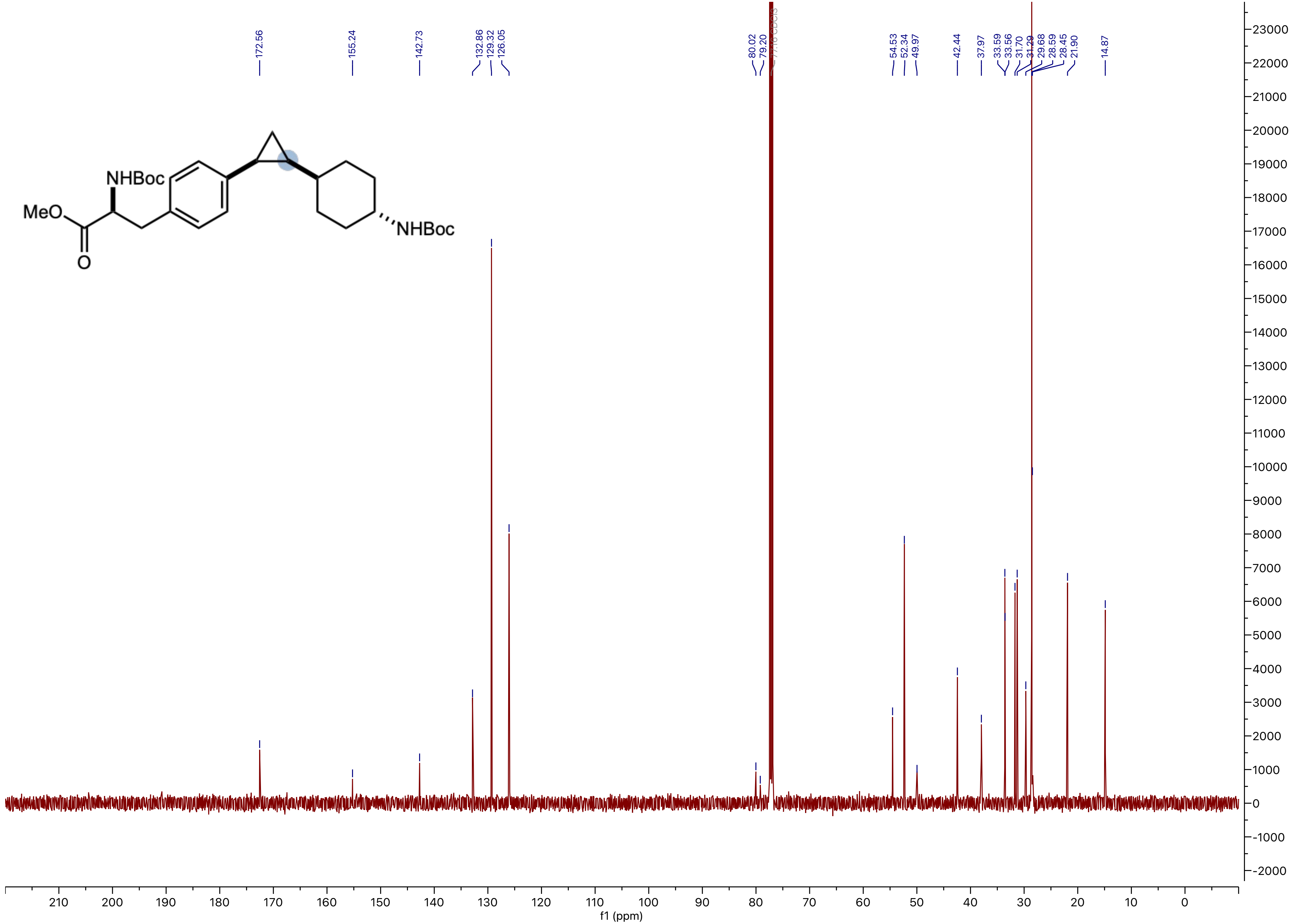

NHBoc
MeO
O
NHBoc
172.56
155.24
142.73
132.86
129.32
126.05
80.02
79.20
77.16 CDCl3
54.53
52.34
49.97
42.44
37.97
33.59
33.56
31.70
31.29
29.68
28.59
28.45
21.90
14.87
f1 (ppm)

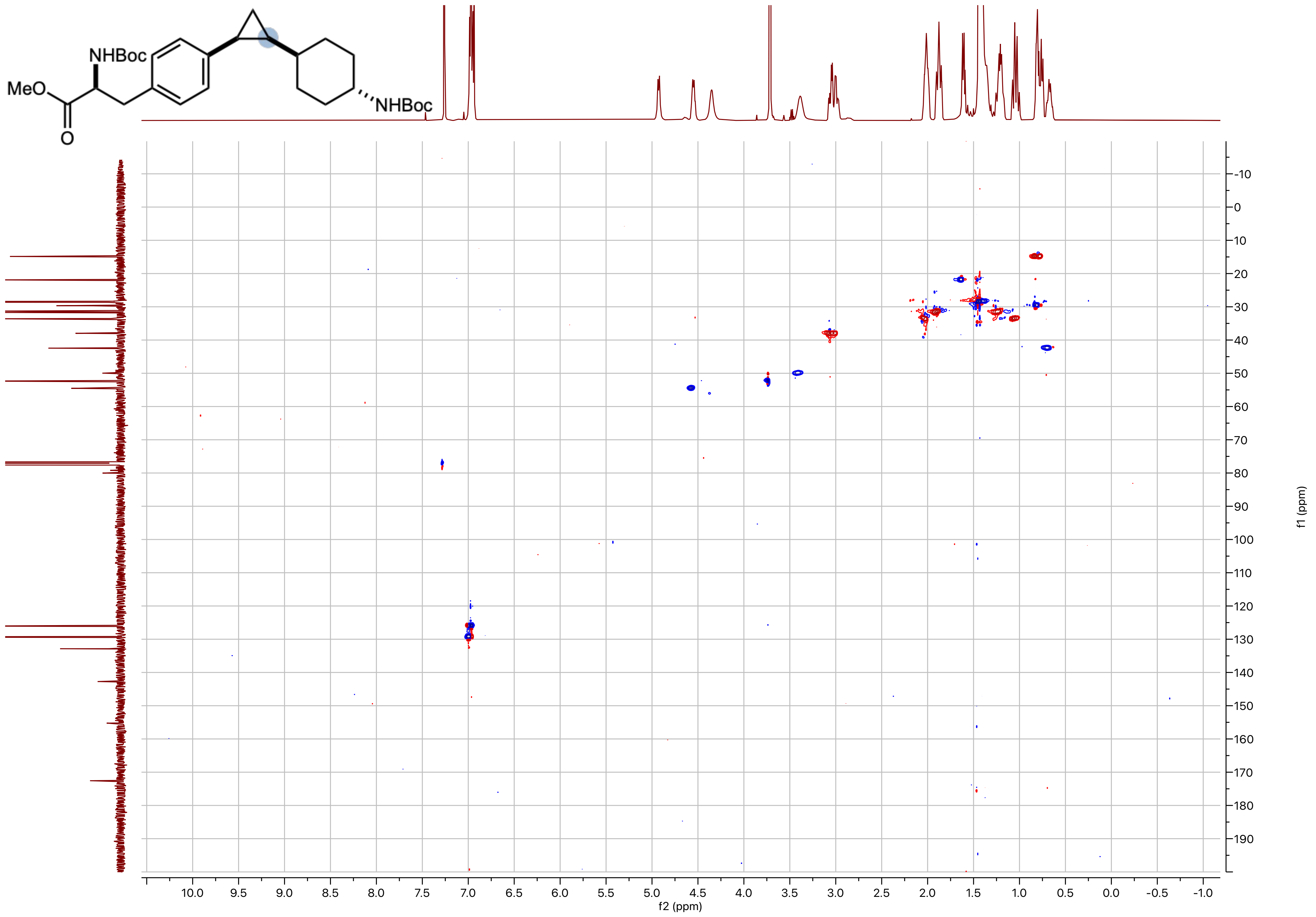
NHBoc
MeO
O
NHBoc
f2 (ppm)
f1 (ppm)

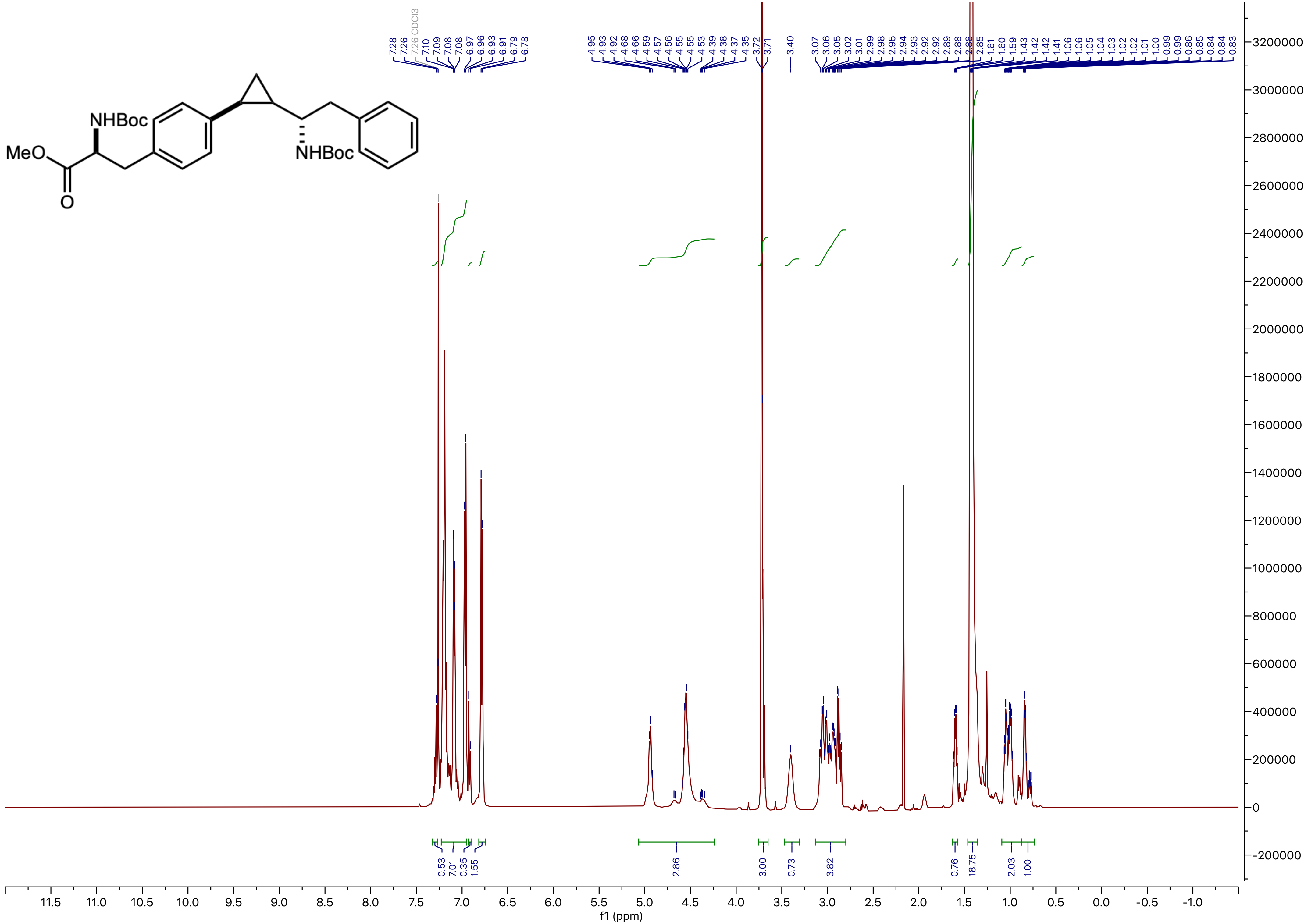

NHBoc
MeO
O
NHBoc
7.28
7.26
7.26 CDCl3
7.10
7.09
7.08
6.97
6.96
6.93
6.91
6.79
6.78
4.95
4.93
4.92
4.68
4.66
4.59
4.57
4.56
4.55
4.55
4.53
4.39
4.38
4.37
4.35
3.72
3.71
3.40
3.07
3.06
3.05
3.02
3.01
2.99
2.98
2.95
2.94
2.93
2.92
2.92
2.89
2.88
2.86
2.85
1.61
1.60
1.59
1.43
1.42
1.41
1.06
1.06
1.05
1.04
1.03
1.02
1.02
1.01
1.00
0.99
0.99
0.86
0.85
0.84
0.84
0.83
0.53
7.01
0.35
1.55
2.86
3.00
0.73
3.82
0.76
18.75
2.03
1.00
f1 (ppm)

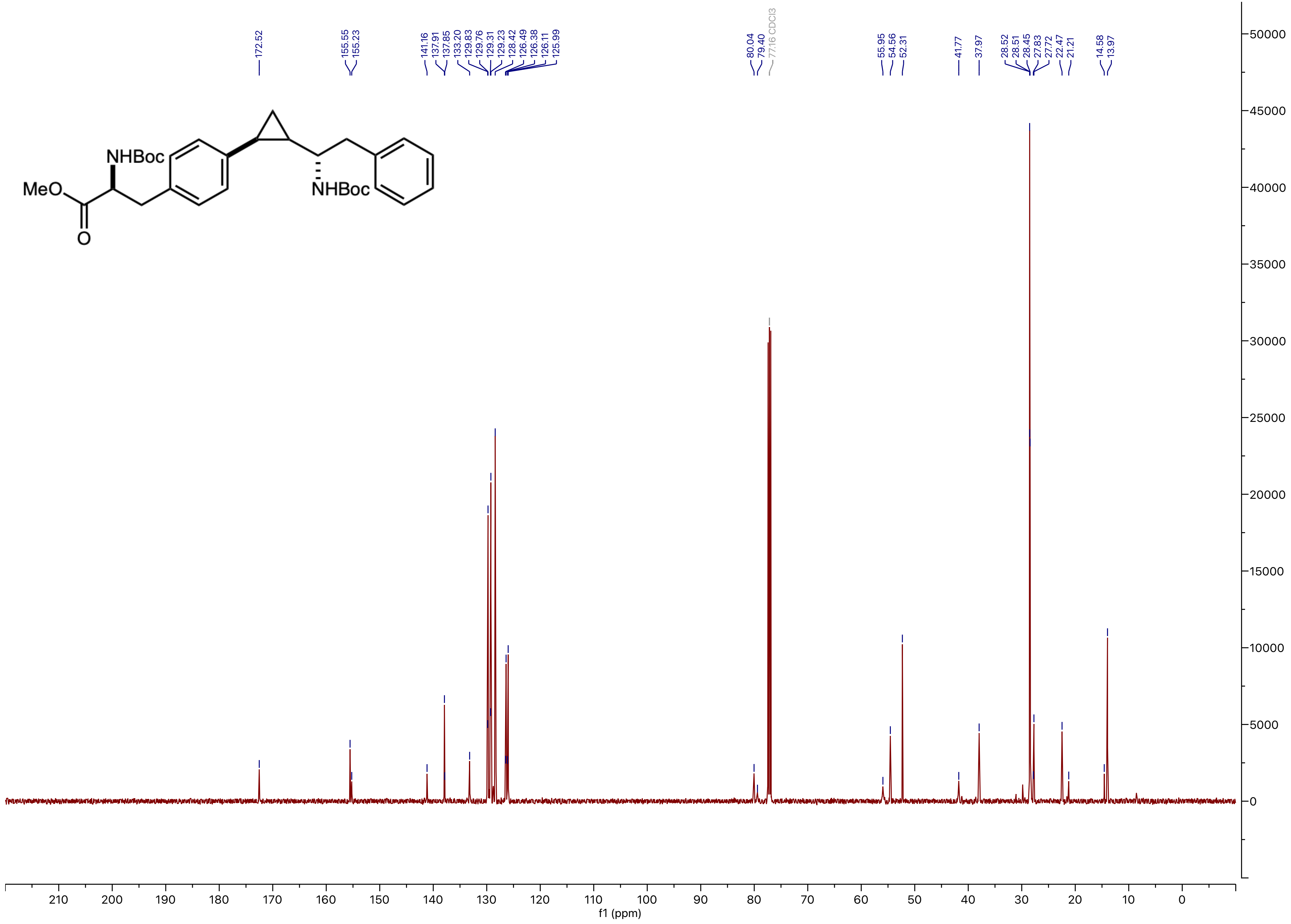

172.52
155.55
155.23
141.16
137.91
137.85
133.20
129.83
129.76
129.31
129.23
128.42
126.49
126.38
126.11
125.99
80.04
79.40
77.16 CDCl3
55.95
54.56
52.31
41.77
37.97
28.52
28.51
28.45
27.83
27.72
22.47
21.21
14.58
13.97
NHBoc
MeO
O
NHBoc
f1 (ppm)

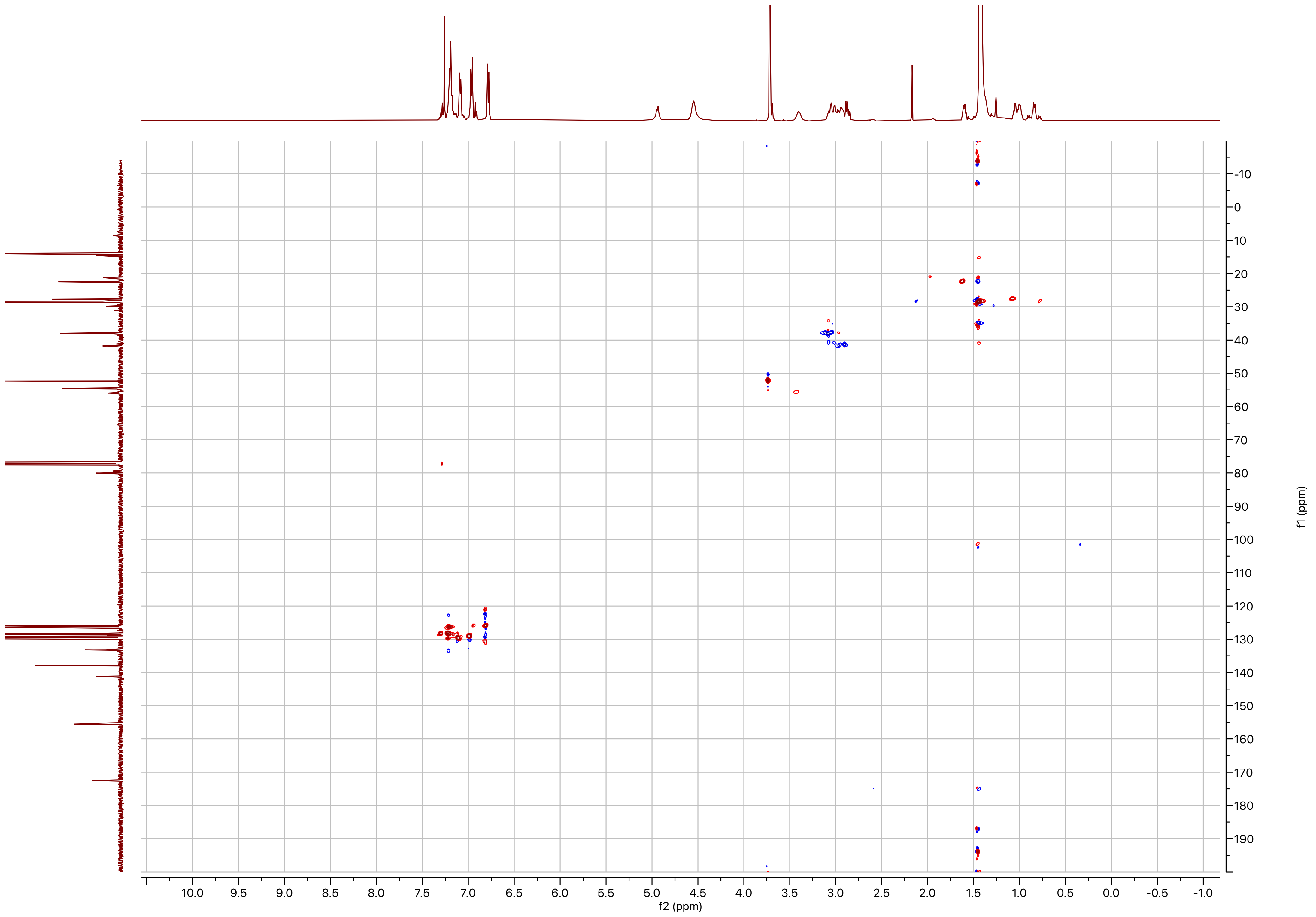

f2 (ppm)
f1 (ppm)

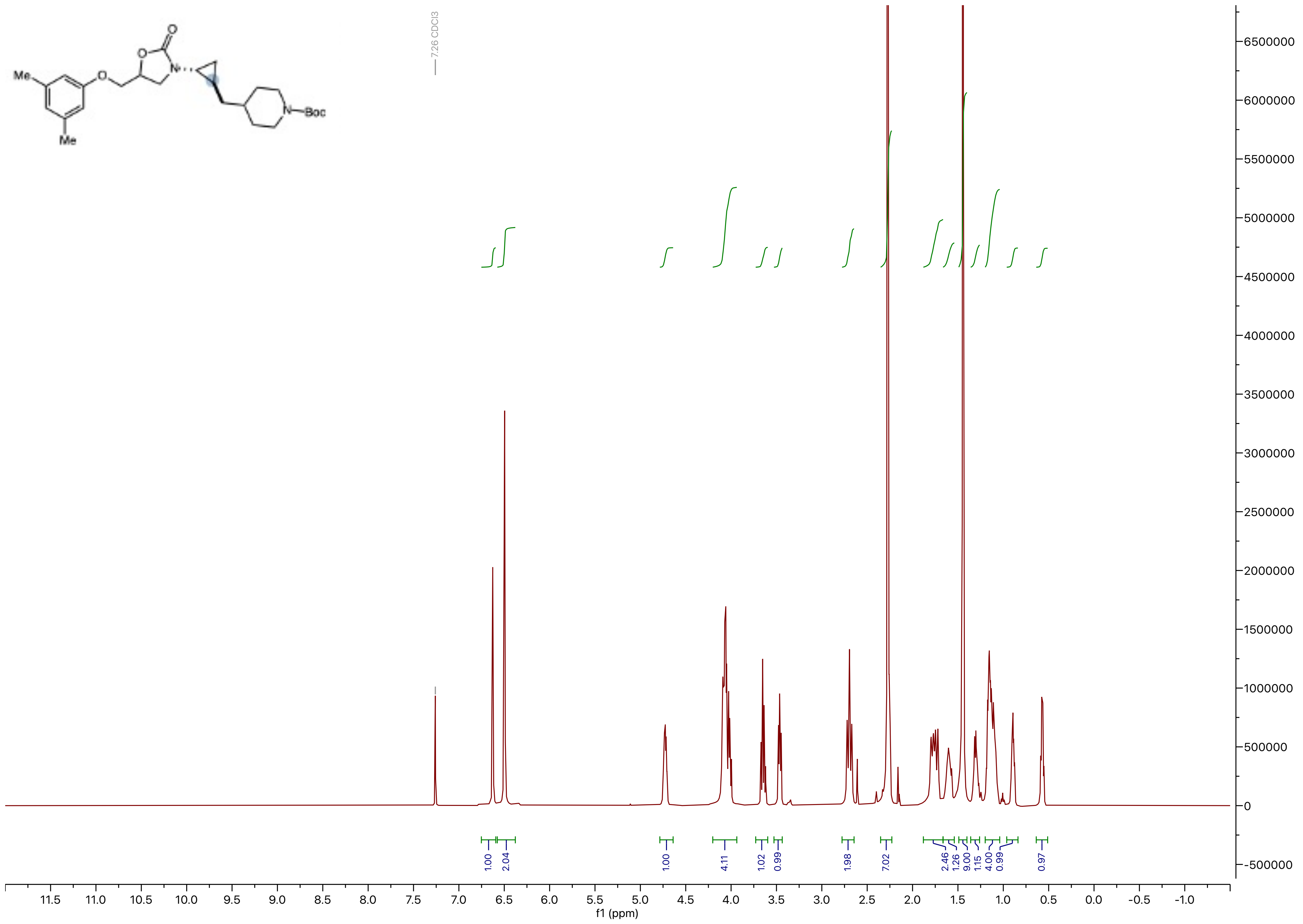

Me
O
O
N
O
N—Boc
Me
7.26 CDCl3
1.00
2.04
1.00
4.11
1.02
0.99
1.98
7.02
2.46
1.26
9.00
1.15
4.00
0.99
0.97
f1 (ppm)

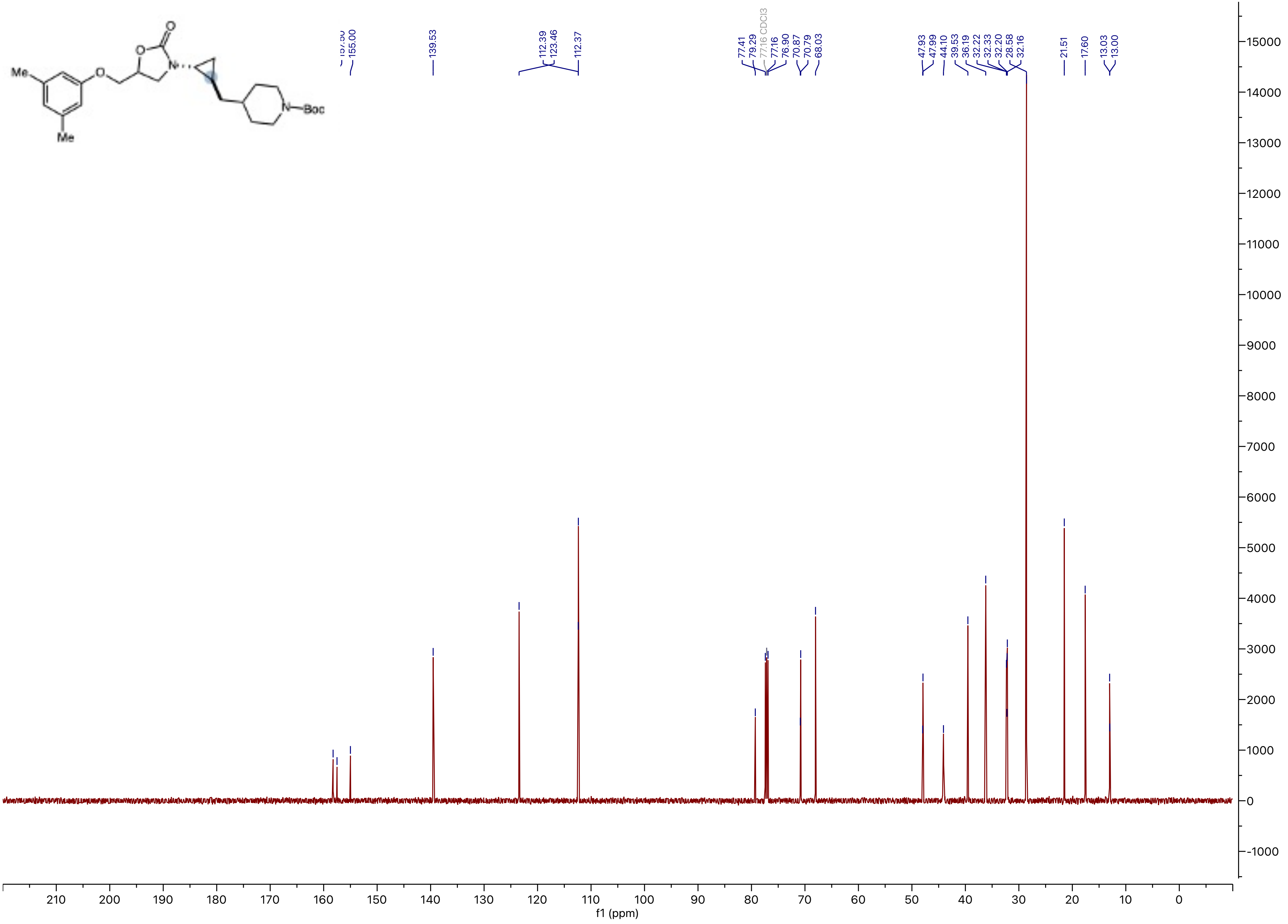

Me
Me
O
O
O
N
N
Boc
155.00
139.53
112.39
123.46
112.37
77.41
79.29
77.16 CDCl3
77.16
76.90
70.87
70.79
68.03
47.93
47.99
44.10
39.53
36.19
32.22
32.33
32.20
28.58
32.16
21.51
17.60
13.03
13.00
f1 (ppm)

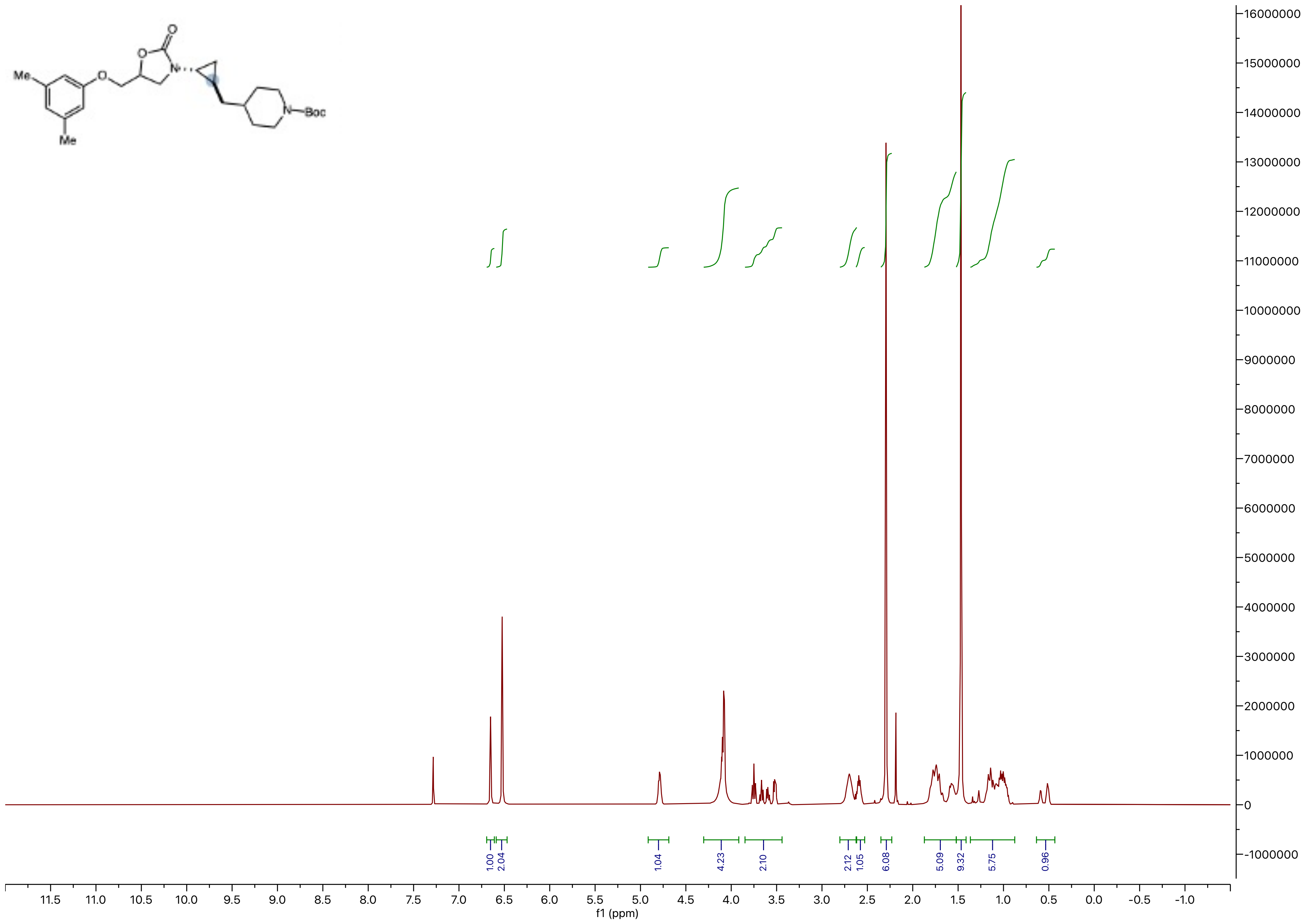

Me
O
O
O
N
N
Boc
Me
1.00
2.04
1.04
4.23
2.10
2.12
1.05
6.08
5.09
9.32
5.75
0.96
f1 (ppm)

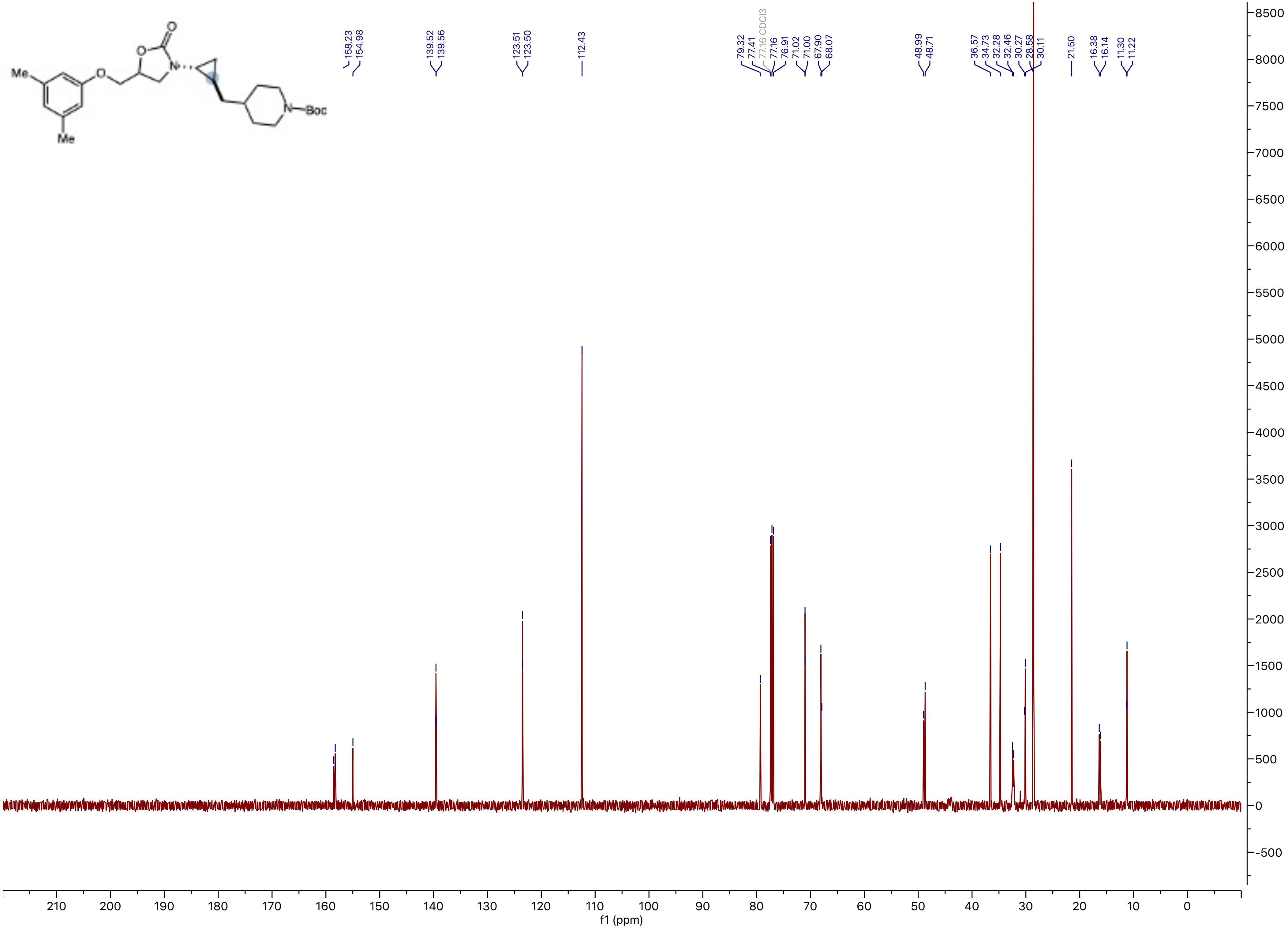
Me
O
O
O
N
N
Boc
Me
158.23
154.98
139.52
139.56
123.51
123.50
112.43
79.32
77.41
77.16 CDCl3
77.16
76.91
71.02
71.00
67.90
68.07
48.99
48.71
36.57
34.73
32.28
32.46
30.27
28.58
30.11
21.50
16.38
16.14
11.30
11.22
f1 (ppm)

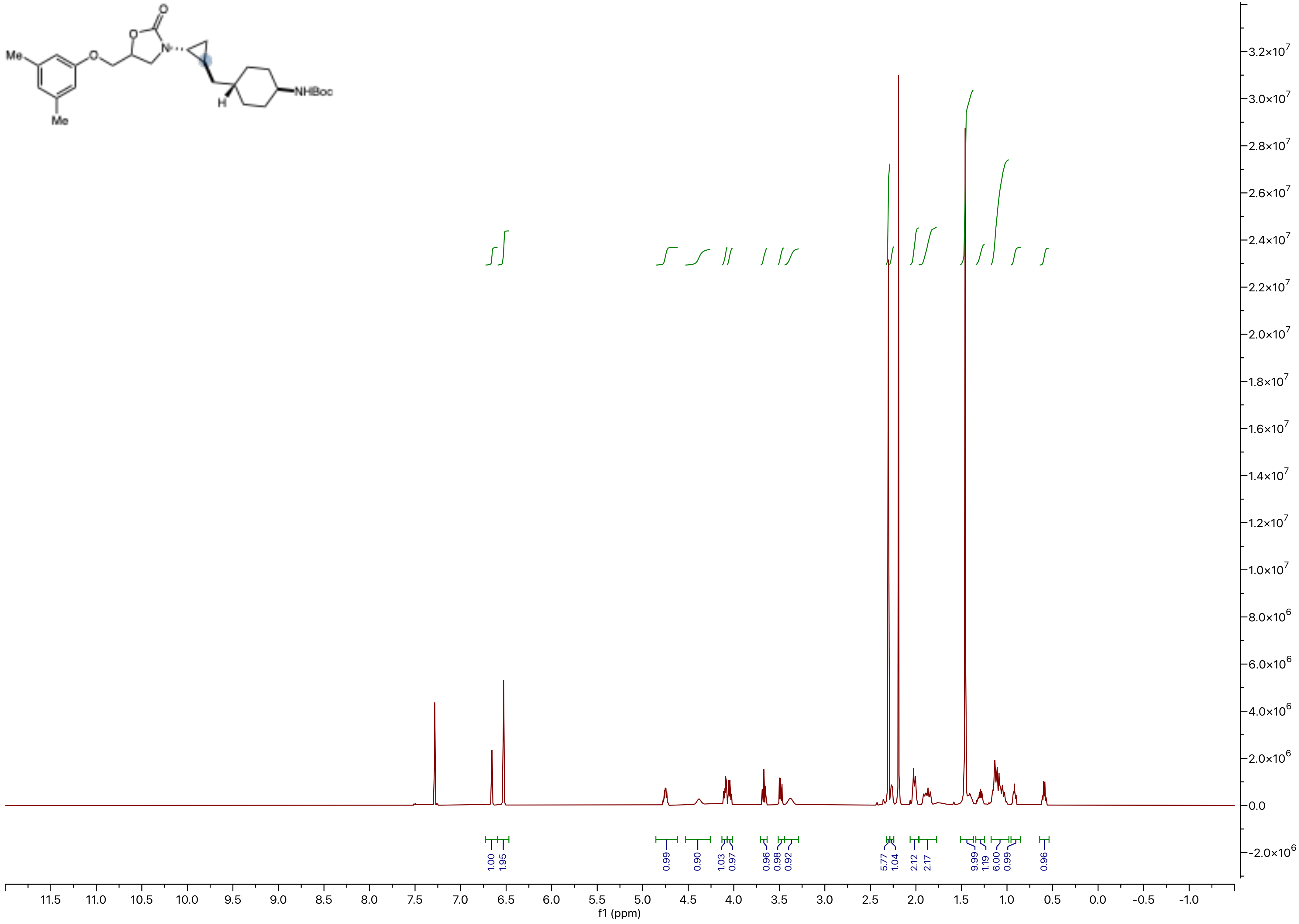

Me
O
O
N
O
Me
H
NHBoc
3.2×10^7
3.0×10^7
2.8×10^7
2.6×10^7
2.4×10^7
2.2×10^7
2.0×10^7
1.8×10^7
1.6×10^7
1.4×10^7
1.2×10^7
1.0×10^7
8.0×10^6
6.0×10^6
4.0×10^6
2.0×10^6
0.0
-2.0×10^6
1.00
1.95
0.99
0.90
1.03
0.97
0.96
0.98
0.92
5.77
1.04
2.12
2.17
9.99
1.19
6.00
0.99
0.96
11.5 11.0 10.5 10.0 9.5 9.0 8.5 8.0 7.5 7.0 6.5 6.0 5.5 5.0 4.5 4.0 3.5 3.0 2.5 2.0 1.5 1.0 0.5 0.0 -0.5 -1.0
f1 (ppm)

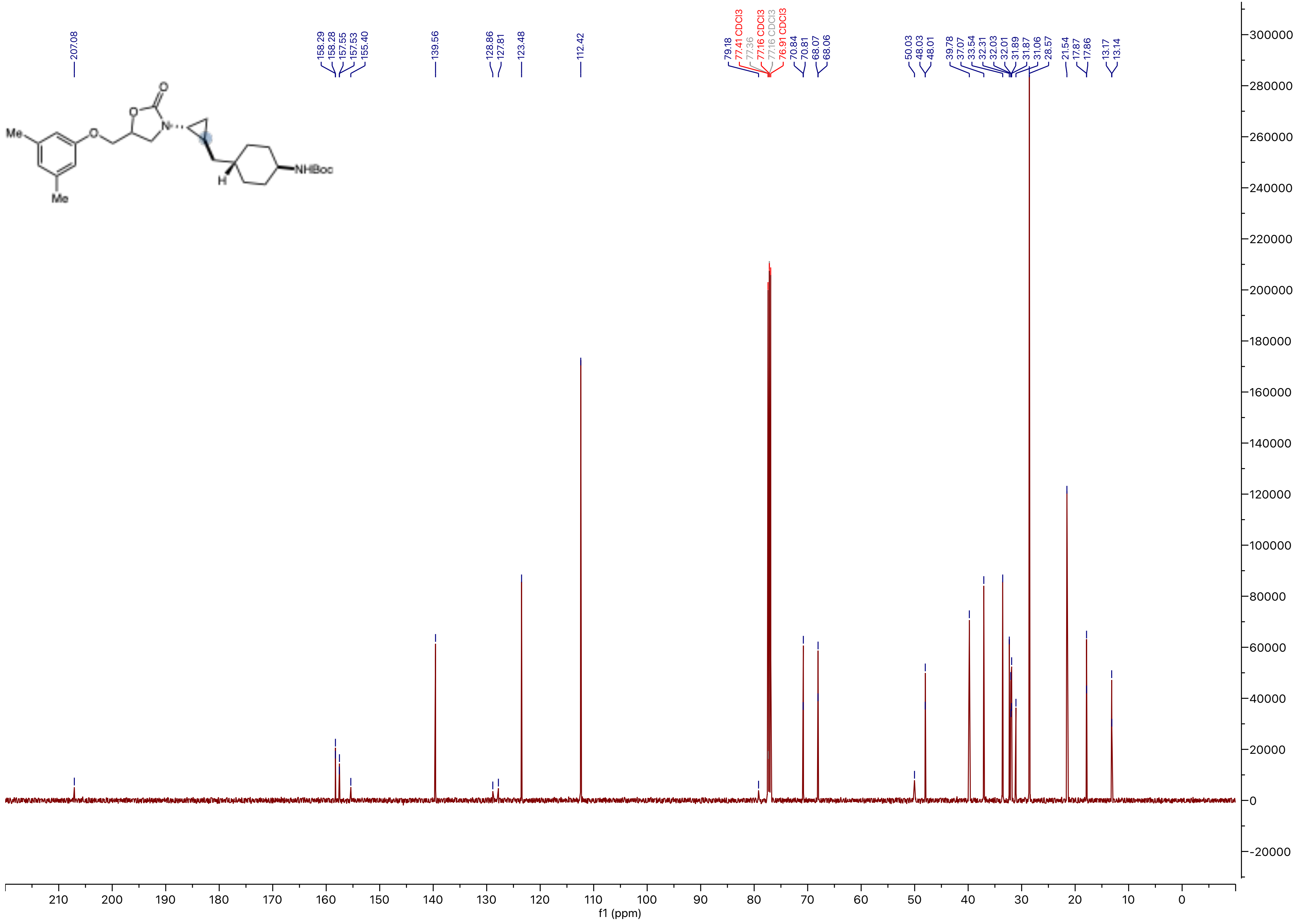
207.08
158.29
158.28
157.55
157.53
155.40
139.56
128.86
127.81
123.48
112.42
79.18
77.41 CDCl3
77.36
77.16 CDCl3
77.16 CDCl3
76.91 CDCl3
70.84
70.81
68.07
68.06
50.03
48.03
48.01
39.78
37.07
33.54
32.31
32.03
32.01
31.89
31.87
31.06
28.57
21.54
17.87
17.86
13.17
13.14
Me
O
O
N
O
H
NHBoc
Me
f1 (ppm)
210
200
190
180
170
160
150
140
130
120
110
100
90
80
70
60
50
40
30
20
10
0
300000
280000
260000
240000
220000
200000
180000
160000
140000
120000
100000
80000
60000
40000
20000
0
-20000

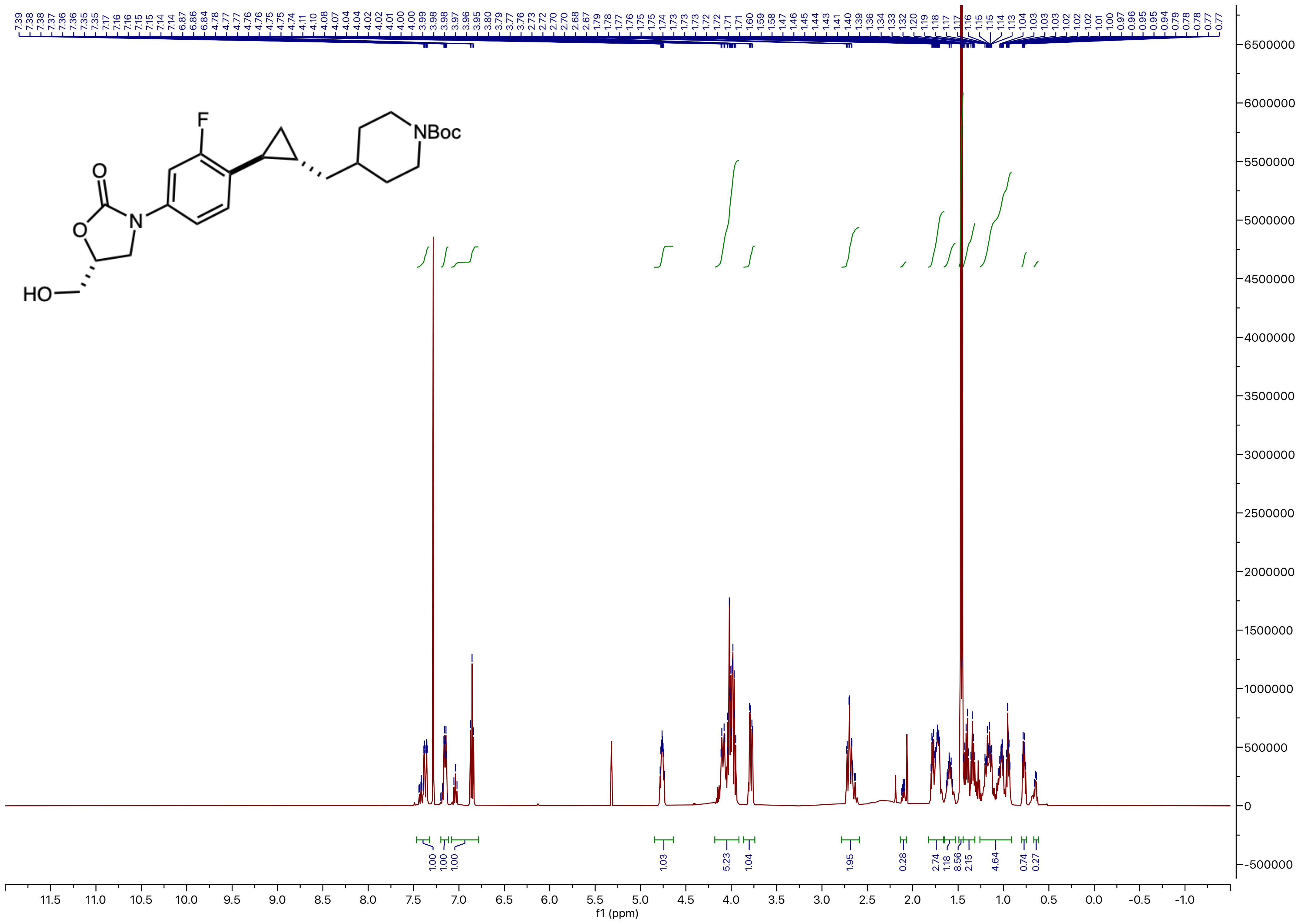

F
O
N
NBoc
O
HO
f1 (ppm)

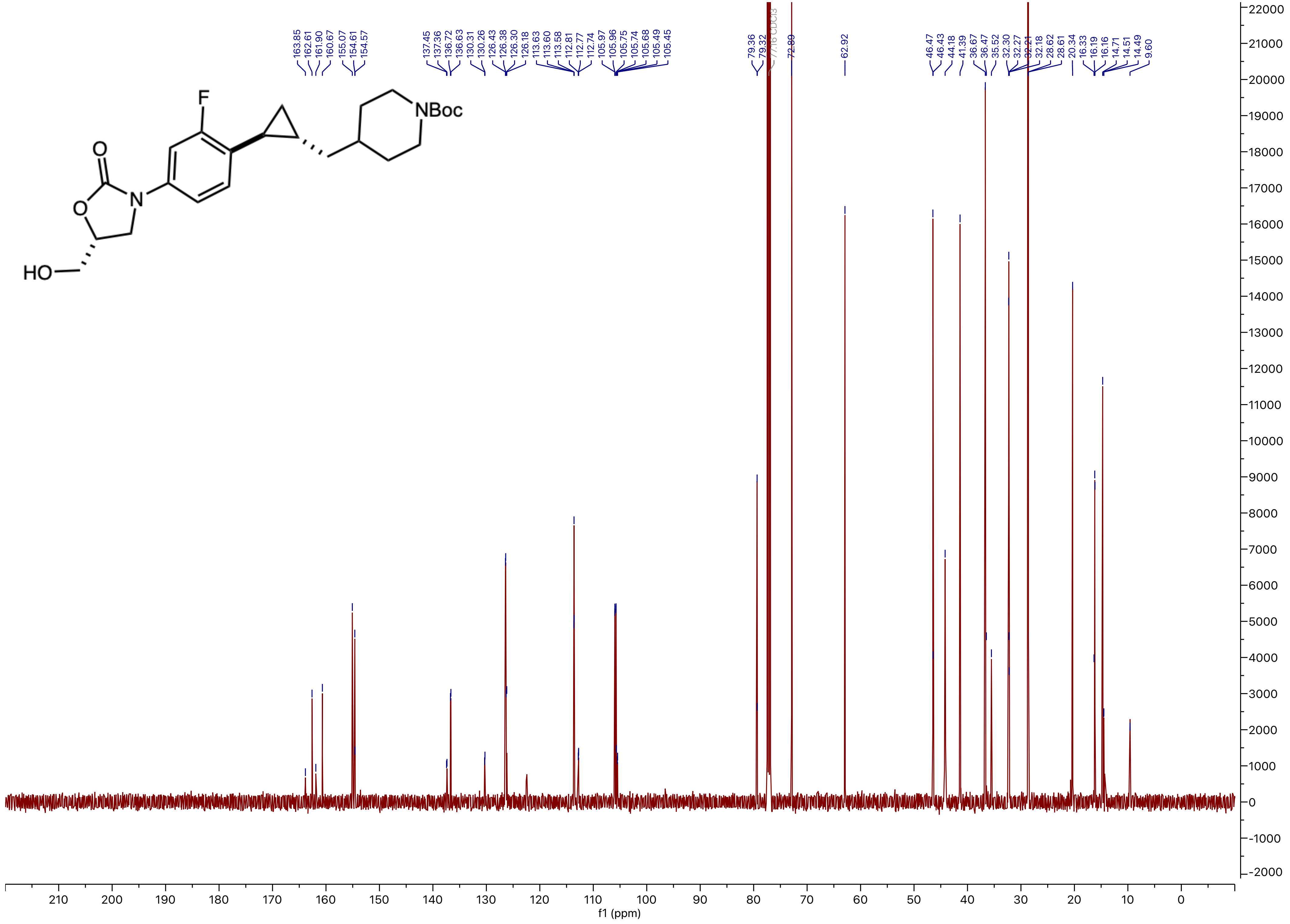

163.85
162.61
161.90
160.67
155.07
154.61
154.57
137.45
137.36
136.72
136.63
130.31
130.26
126.43
126.38
126.30
126.18
113.63
113.60
113.58
112.81
112.77
112.74
105.97
105.96
105.75
105.74
105.68
105.49
105.45
79.36
79.32
77.16 CDCl3
72.89
62.92
46.47
46.43
44.18
41.39
36.67
36.47
35.52
32.30
32.27
32.21
32.18
28.62
28.61
20.34
16.33
16.19
16.16
14.71
14.51
14.49
9.60
F
NBoc
O
N
HO
f1 (ppm)

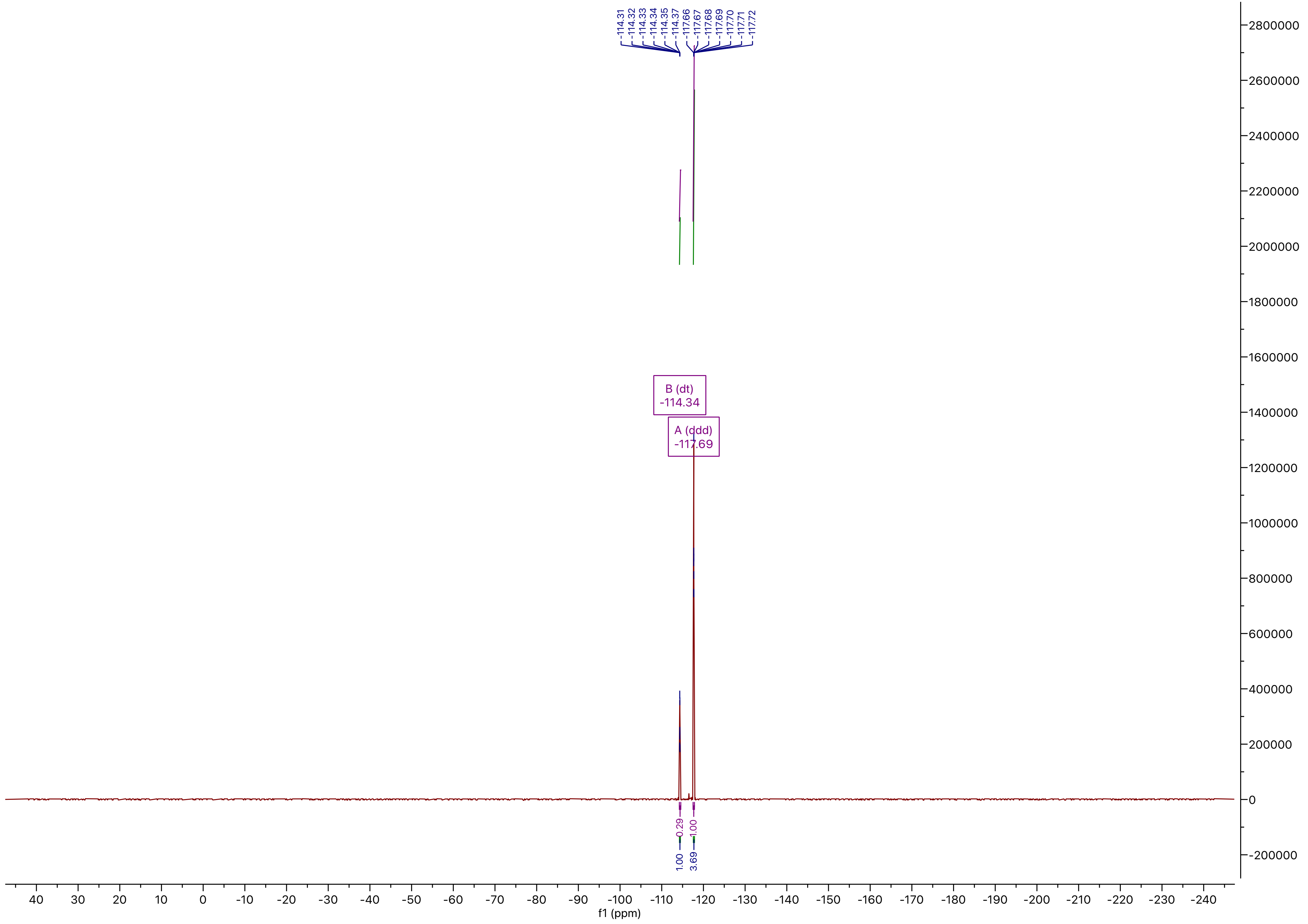

-114.31
-114.32
-114.33
-114.34
-114.35
-114.37
-117.66
-117.67
-117.68
-117.69
-117.70
-117.71
-117.72
B (dt)
-114.34
A (ddd)
-117.69
0.29
1.00
1.00
3.69
2800000
2600000
2400000
2200000
2000000
1800000
1600000
1400000
1200000
1000000
800000
600000
400000
200000
0
-200000
40 30 20 10 0 -10 -20 -30 -40 -50 -60 -70 -80 -90 -100 -110 -120 -130 -140 -150 -160 -170 -180 -190 -200 -210 -220 -230 -240
f1 (ppm)

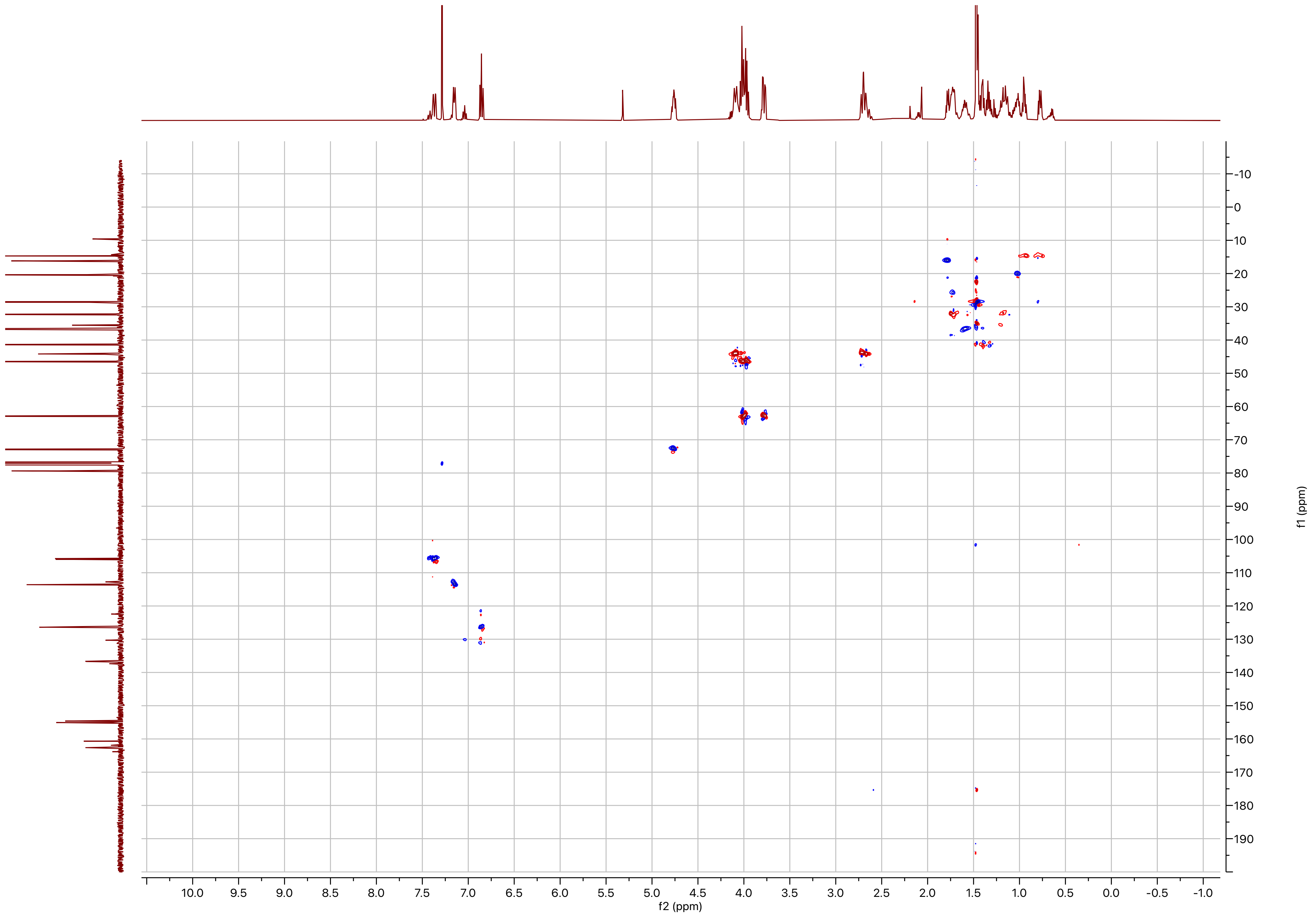
f2 (ppm)
f1 (ppm)

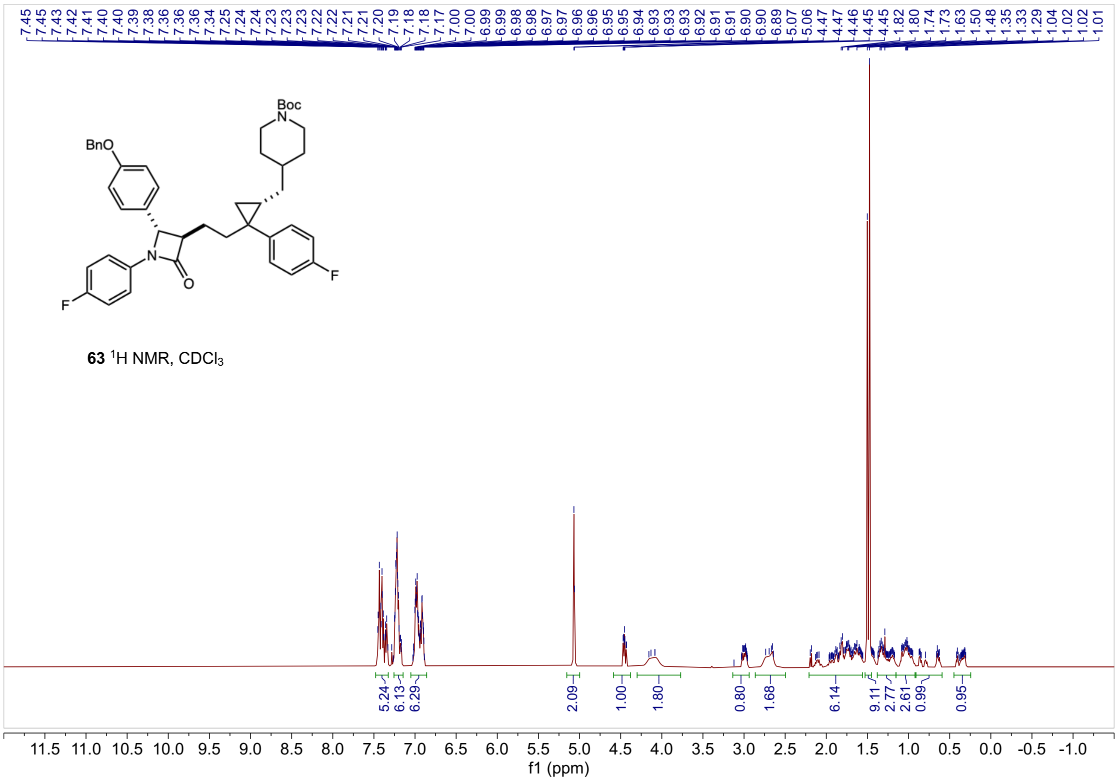
7.45 7.45 7.43 7.42 7.41 7.40 7.40 7.39 7.38 7.36 7.36 7.34 7.25 7.24 7.24 7.23 7.23 7.23 7.22 7.22 7.21 7.21 7.20 7.19 7.18 7.17 7.00 6.99 6.99 6.98 6.98 6.97 6.97 6.96 6.96 6.95 6.95 6.94 6.93 6.93 6.93 6.92 6.91 6.91 6.90 6.90 6.89 5.07 5.06 4.47 4.46 4.45 4.45 1.82 1.80 1.74 1.73 1.63 1.50 1.48 1.35 1.33 1.29 1.04 1.02 1.02 1.01
Boc
N
BnO
N
O
F
F
63 ^{1}H NMR, $CDCl_3$
5.24 6.13 6.29 2.09 1.00 1.80 0.80 1.68 6.14 9.11 2.77 2.61 0.99 0.95
11.5 11.0 10.5 10.0 9.5 9.0 8.5 8.0 7.5 7.0 6.5 6.0 5.5 5.0 4.5 4.0 3.5 3.0 2.5 2.0 1.5 1.0 0.5 0.0 -0.5 -1.0
f1 (ppm)

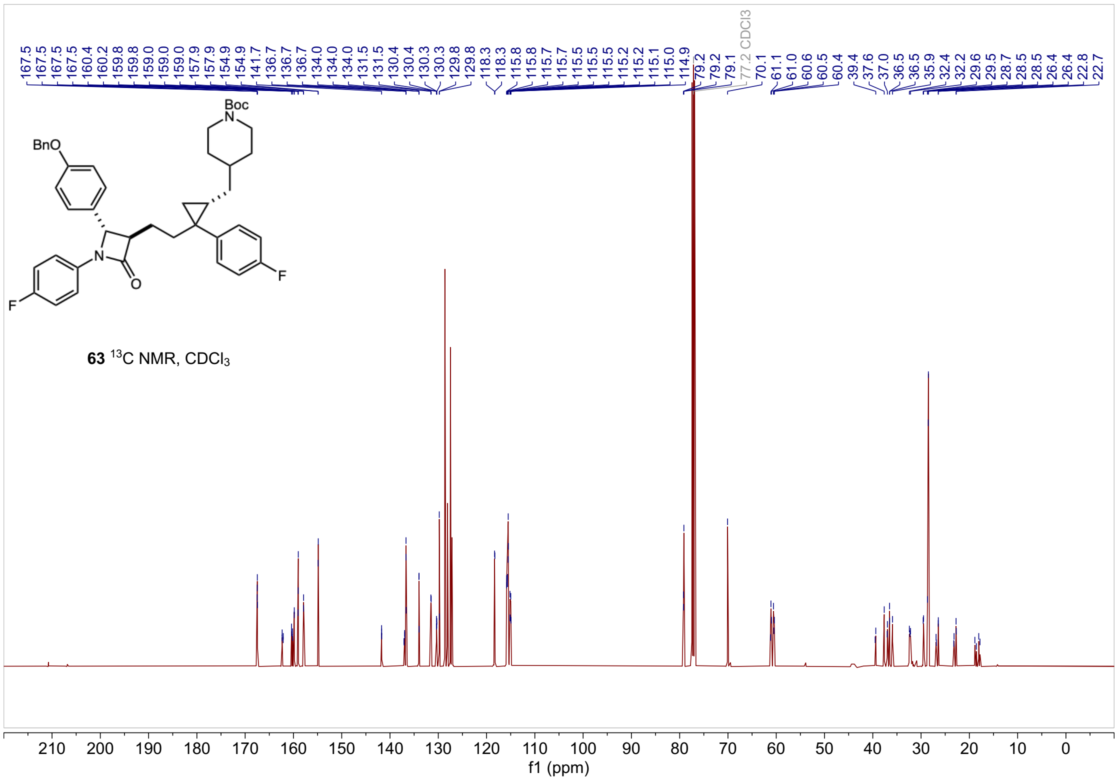
167.5 167.5 167.5 160.4 160.2 159.8 159.8 159.0 159.0 157.9 157.9 154.9 154.9 141.7 136.7 136.7 136.7 134.0 134.0 131.5 131.5 130.4 130.4 130.3 130.3 129.8 129.8 118.3 118.3 115.8 115.8 115.7 115.7 115.5 115.5 115.2 115.2 115.1 115.0 114.9 79.2 79.2 79.1 77.2 CDCl3 70.1 61.1 61.0 60.6 60.5 60.4 39.4 37.6 37.0 36.5 36.5 35.9 32.4 32.2 29.6 29.5 28.7 28.5 26.4 26.4 22.8 22.7
Boc
N
BnO
N
O
F
F
63 ^{13}C NMR, $CDCl_3$
210 200 190 180 170 160 150 140 130 120 110 100 90 80 70 60 50 40 30 20 10 0
f1 (ppm)

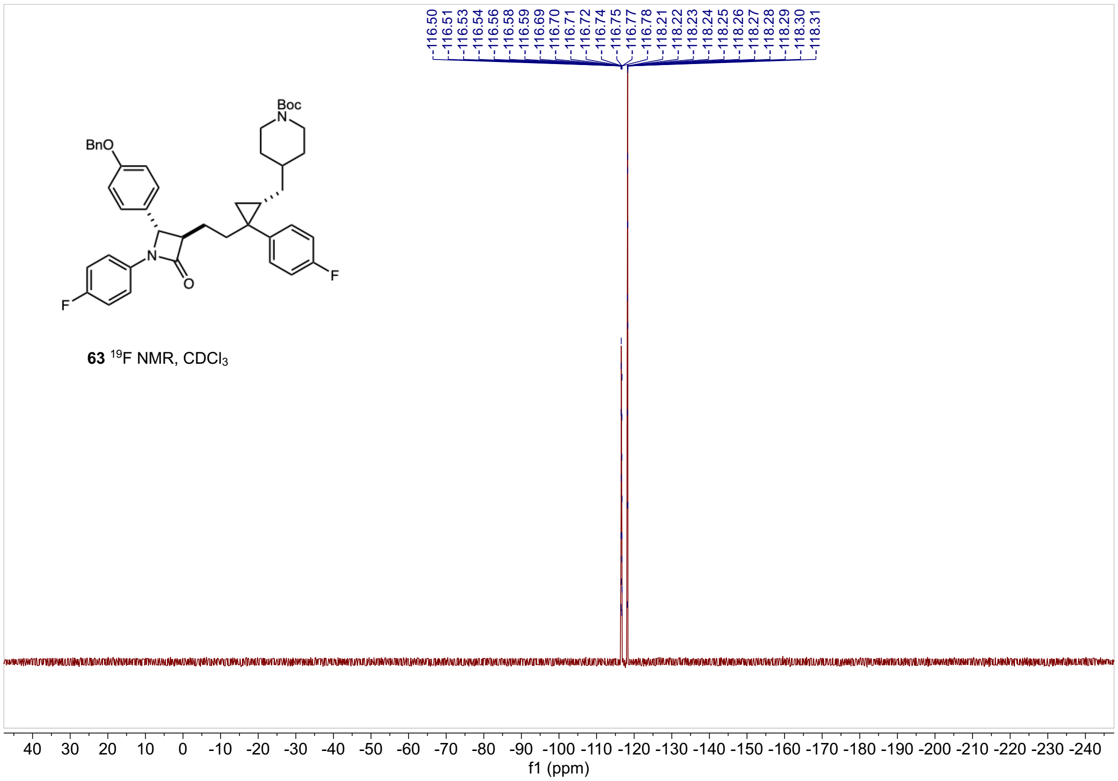

-116.50
-116.51
-116.53
-116.54
-116.56
-116.58
-116.59
-116.69
-116.70
-116.71
-116.72
-116.74
-116.75
-116.77
-116.78
-118.21
-118.22
-118.23
-118.24
-118.25
-118.26
-118.27
-118.28
-118.29
-118.30
-118.31
BnO
Boc
N
N
O
F
F
63 19F NMR, CDCl3
40 30 20 10 0 -10 -20 -30 -40 -50 -60 -70 -80 -90 -100 -110 -120 -130 -140 -150 -160 -170 -180 -190 -200 -210 -220 -230 -240
f1 (ppm)

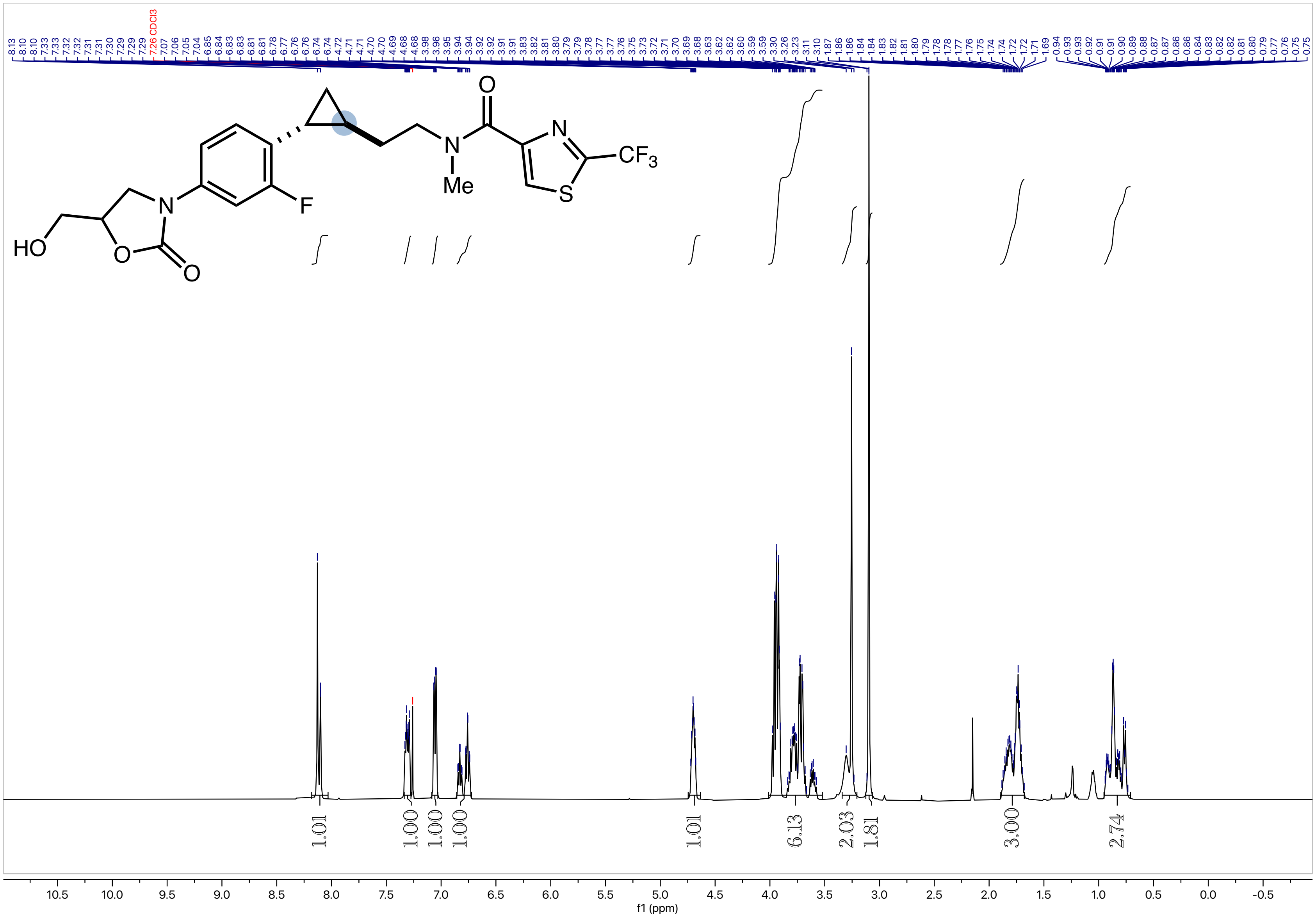
HO
O
O
N
F
N
Me
O
N
S
CF3
1.01
1.00
1.00
1.00
1.01
6.13
2.03
1.81
3.00
2.74
f1 (ppm)

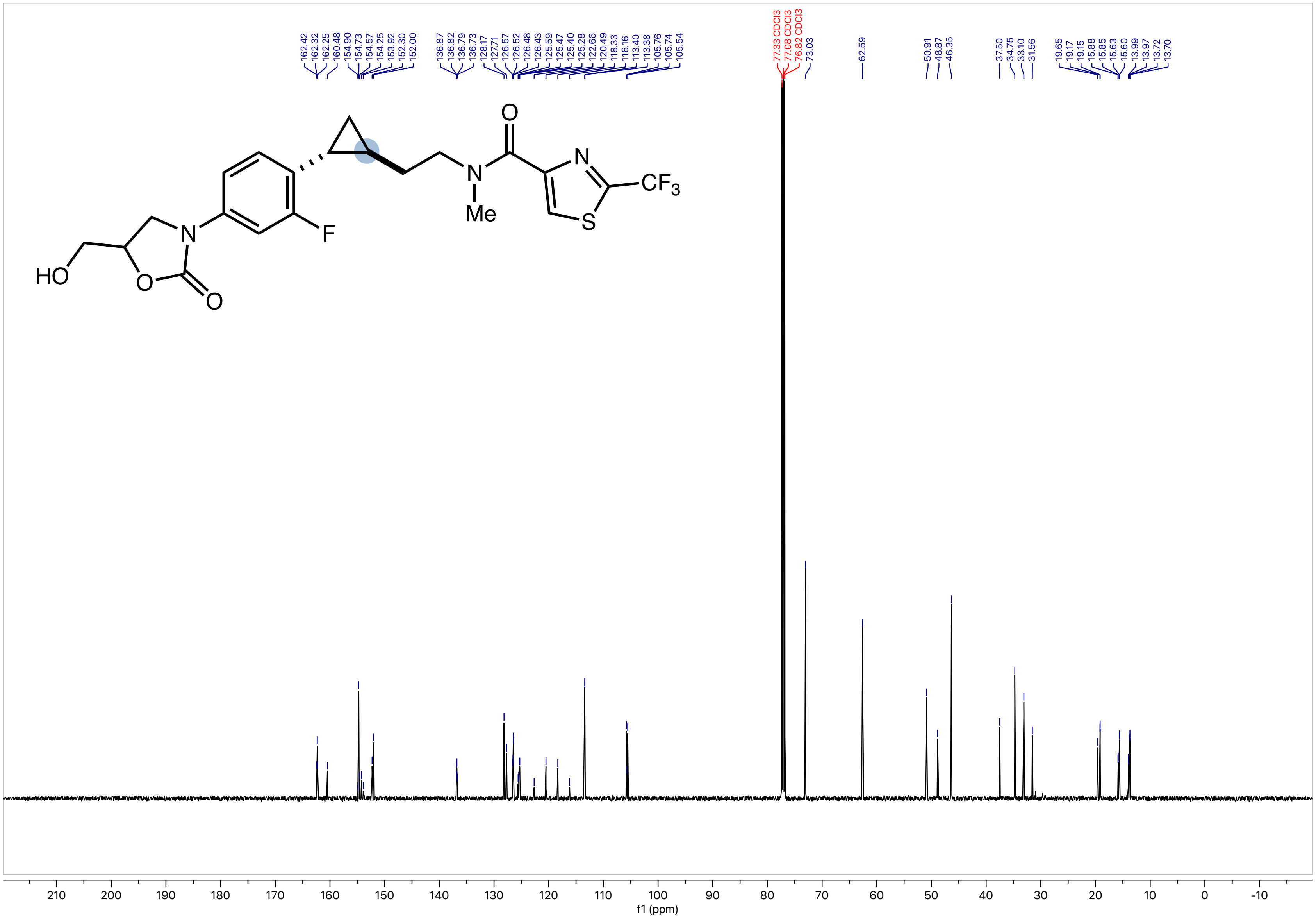

162.42
162.32
162.25
160.48
154.90
154.73
154.57
154.25
153.92
152.30
152.00
136.87
136.82
136.79
136.73
128.17
127.71
126.57
126.52
126.48
126.43
125.59
125.47
125.40
125.28
122.66
120.49
118.33
116.16
113.40
113.38
105.76
105.74
105.54
77.33 CDCl3
77.08 CDCl3
76.82 CDCl3
73.03
62.59
50.91
48.87
46.35
37.50
34.75
33.10
31.56
19.65
19.17
19.15
15.88
15.85
15.63
15.60
13.99
13.97
13.72
13.70
HO
O
O
N
F
N
Me
O
N
S
CF3
210 200 190 180 170 160 150 140 130 120 110 100 90 80 70 60 50 40 30 20 10 0 -10
f1 (ppm)

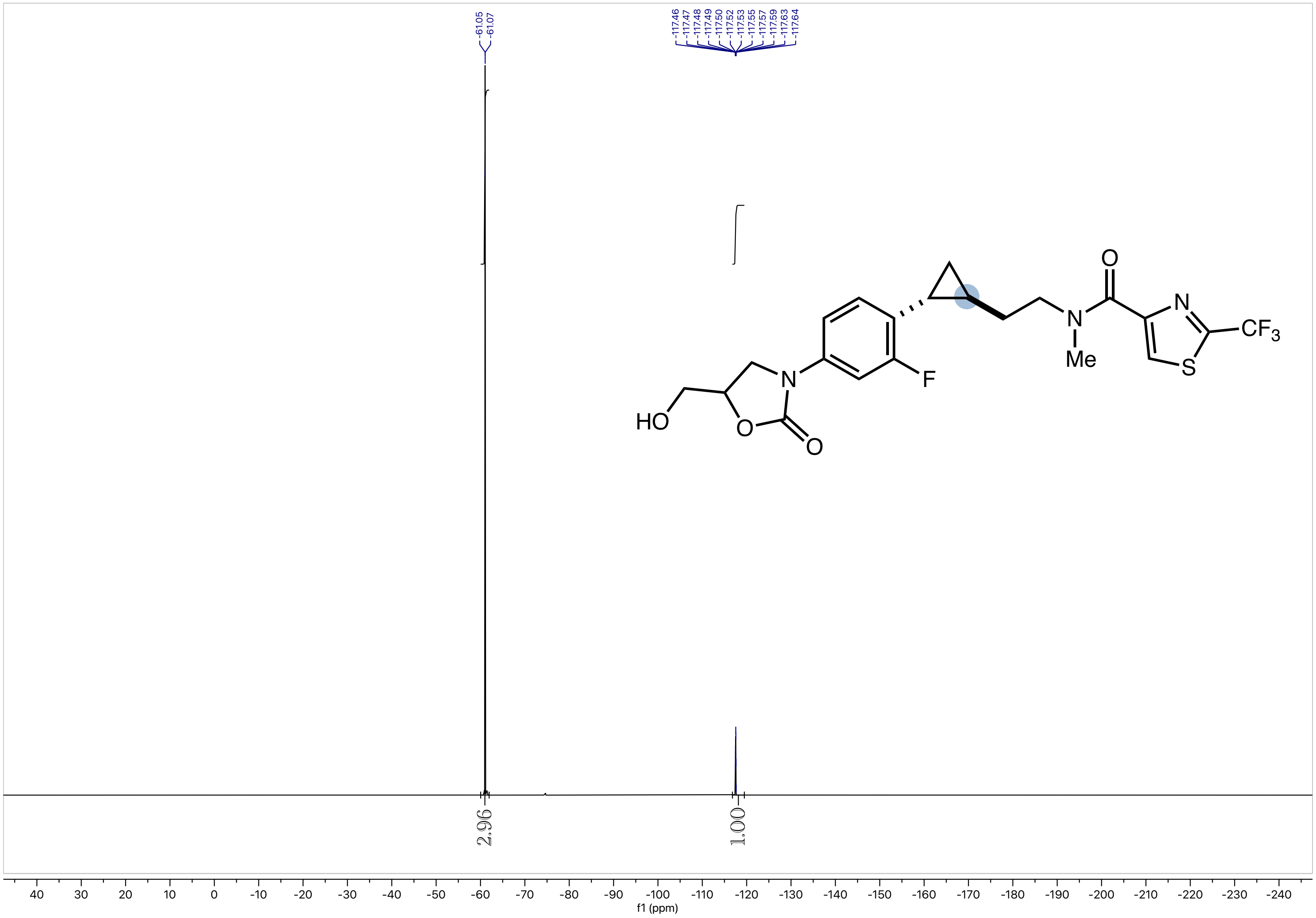

-61.05
-61.07
-117.46
-117.47
-117.48
-117.49
-117.50
-117.52
-117.53
-117.55
-117.57
-117.59
-117.63
-117.64
2.96
1.00
HO
O
N
O
F
N
Me
O
N
S
CF3
40 30 20 10 0 -10 -20 -30 -40 -50 -60 -70 -80 -90 -100 -110 -120 -130 -140 -150 -160 -170 -180 -190 -200 -210 -220 -230 -240
f1 (ppm)

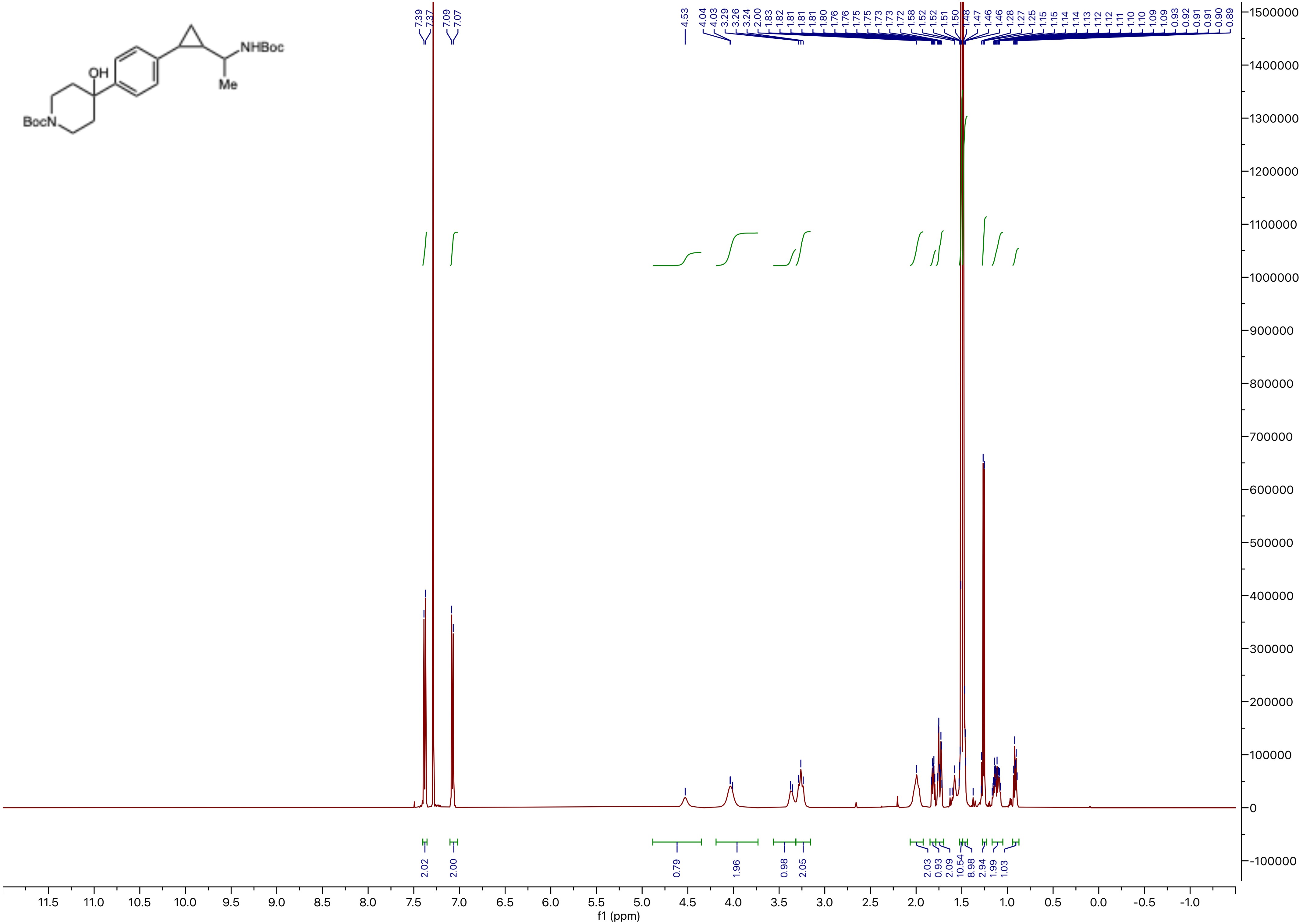
NHBoc
OH
Me
BocN
f1 (ppm)

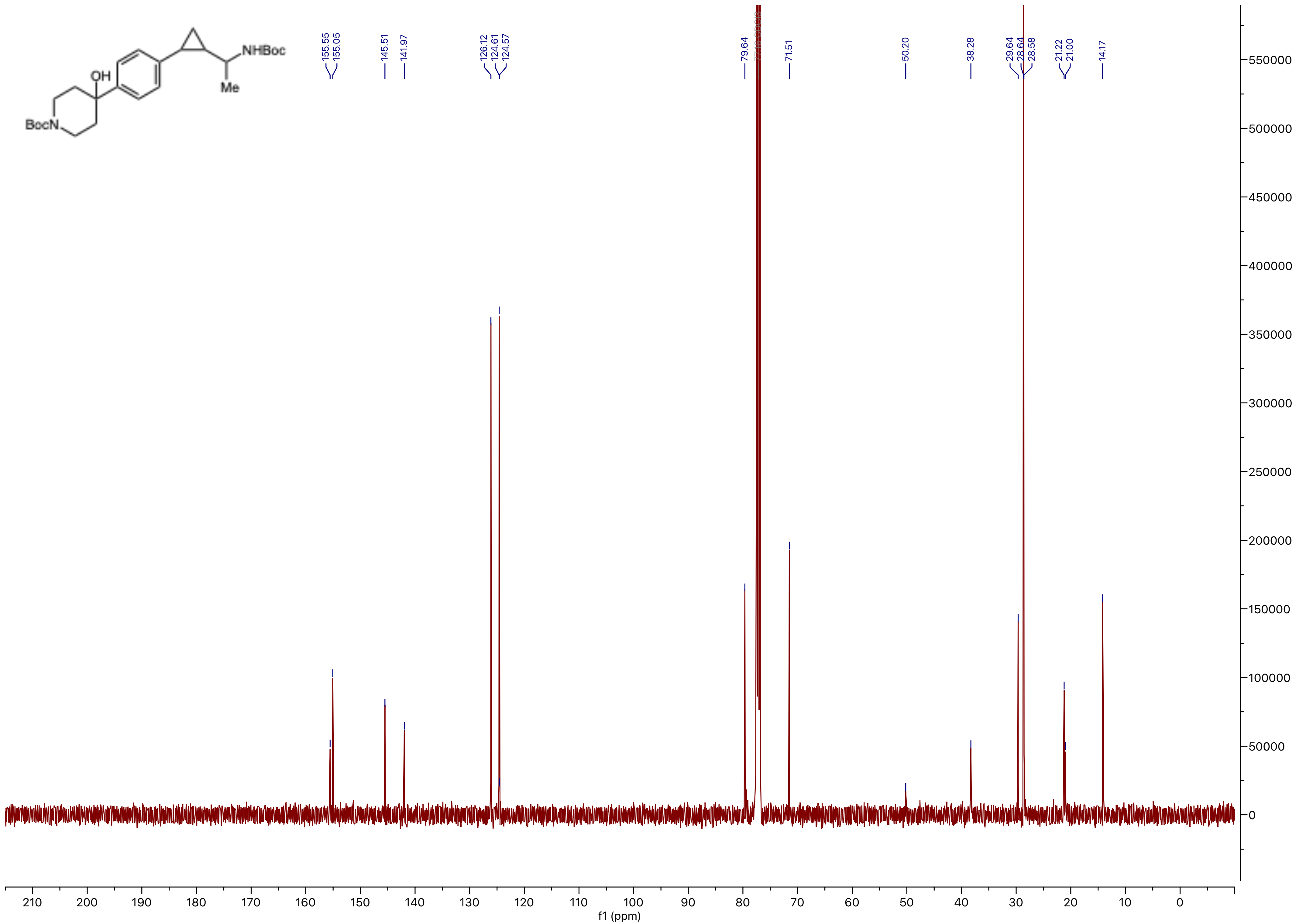

OH
BocN
NHBoc
Me
155.55
155.05
145.51
141.97
126.12
124.61
124.57
79.64
71.51
50.20
38.28
29.64
28.64
28.58
21.22
21.00
14.17
f1 (ppm)

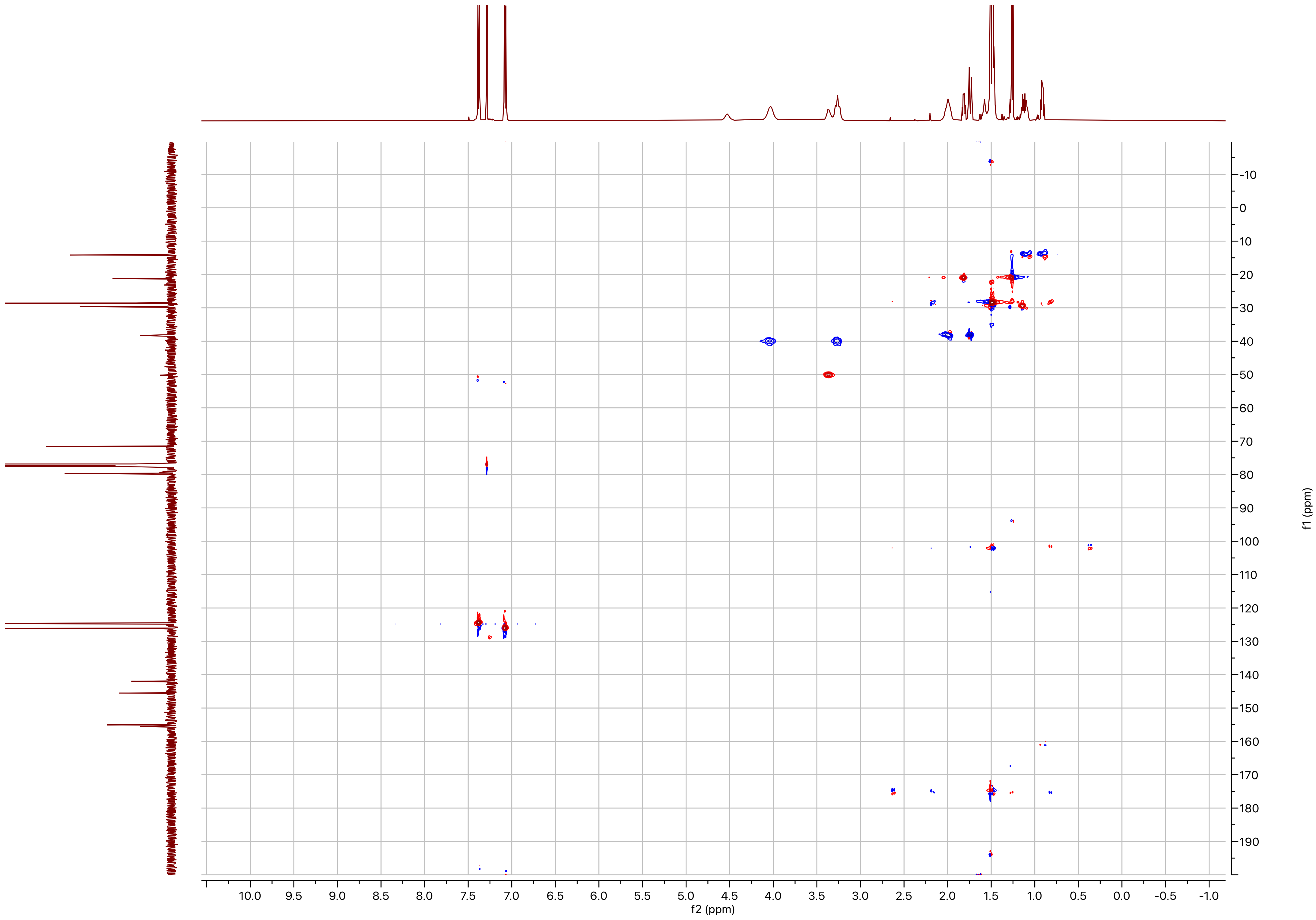
f1 (ppm)
f2 (ppm)

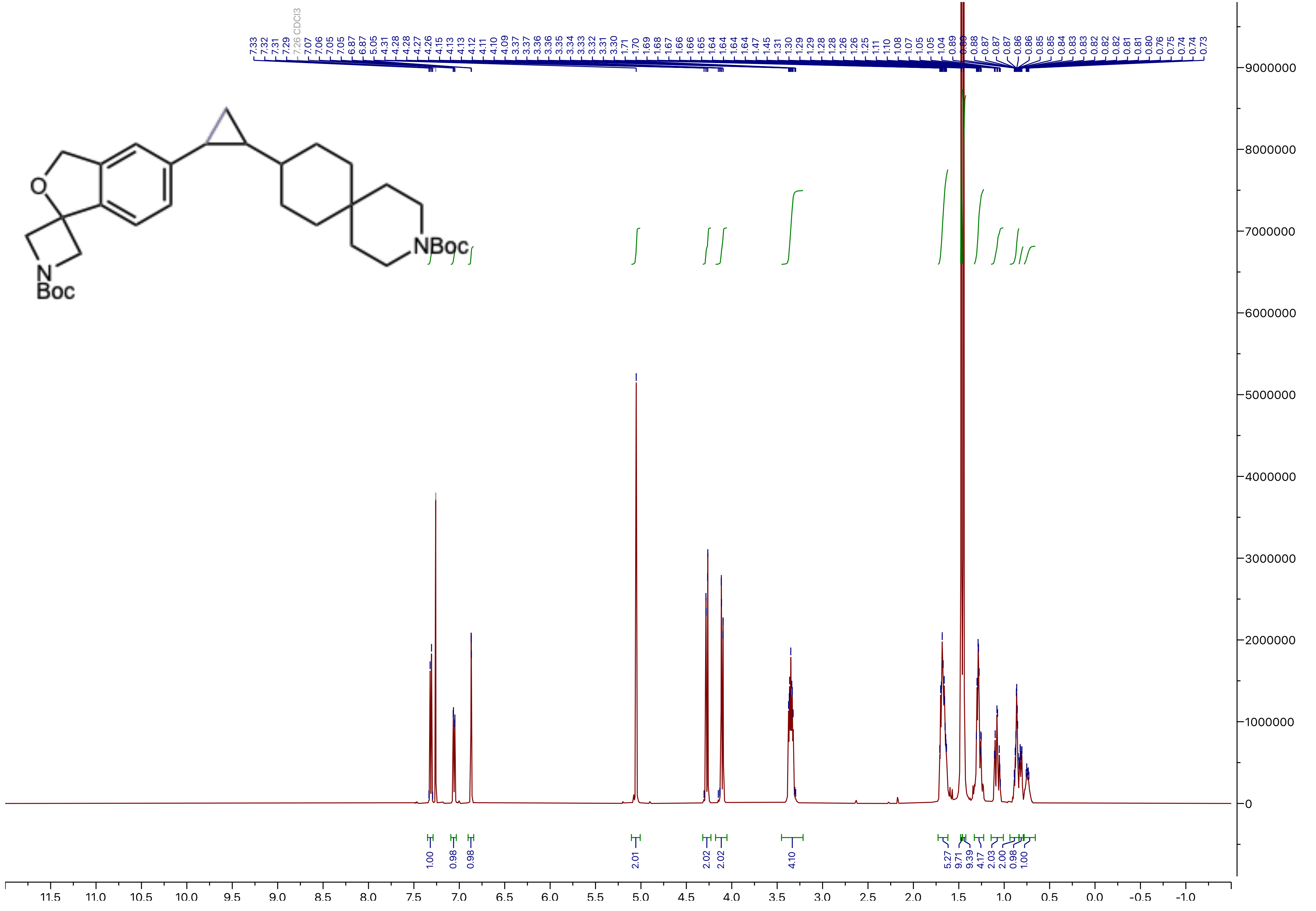
7.33 7.32 7.31 7.29 7.26 CDCl3 7.07 7.06 7.05 7.05 6.87 6.87 5.05 4.31 4.28 4.28 4.27 4.26 4.15 4.13 4.13 4.12 4.11 4.10 4.09 3.37 3.37 3.36 3.36 3.35 3.34 3.33 3.32 3.31 3.30 1.71 1.70 1.69 1.68 1.67 1.66 1.66 1.65 1.64 1.64 1.64 1.64 1.47 1.45 1.31 1.30 1.29 1.29 1.28 1.28 1.26 1.25 1.11 1.10 1.08 1.07 1.05 1.05 1.04 0.89 0.88 0.87 0.87 0.87 0.86 0.86 0.85 0.84 0.83 0.83 0.82 0.82 0.81 0.80 0.76 0.75 0.74 0.73
O
N
Boc
NBoc
1.00 0.98 0.98 2.01 2.02 2.02 4.10 5.27 9.71 9.39 4.17 2.03 2.00 0.98 1.00
11.5 11.0 10.5 10.0 9.5 9.0 8.5 8.0 7.5 7.0 6.5 6.0 5.5 5.0 4.5 4.0 3.5 3.0 2.5 2.0 1.5 1.0 0.5 0.0 -0.5 -1.0
9000000 8000000 7000000 6000000 5000000 4000000 3000000 2000000 1000000 0

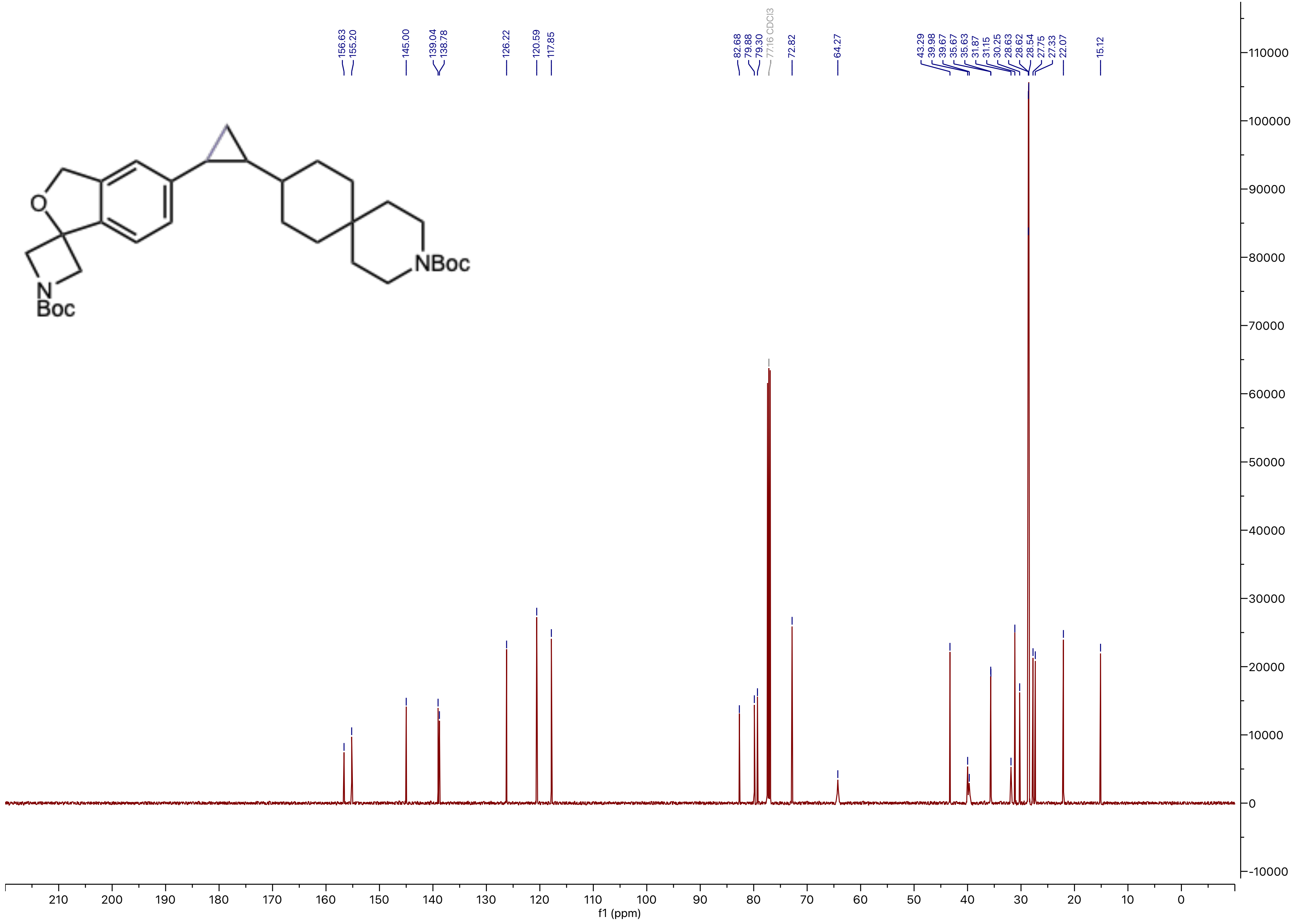

156.63
155.20
145.00
139.04
138.78
126.22
120.59
117.85
82.68
79.88
79.30
77.16 CDCl3
72.82
64.27
43.29
39.98
39.67
35.67
35.63
31.87
31.15
30.25
28.63
28.62
28.54
27.75
27.33
22.07
15.12
O
N
Boc
NBoc
f1 (ppm)

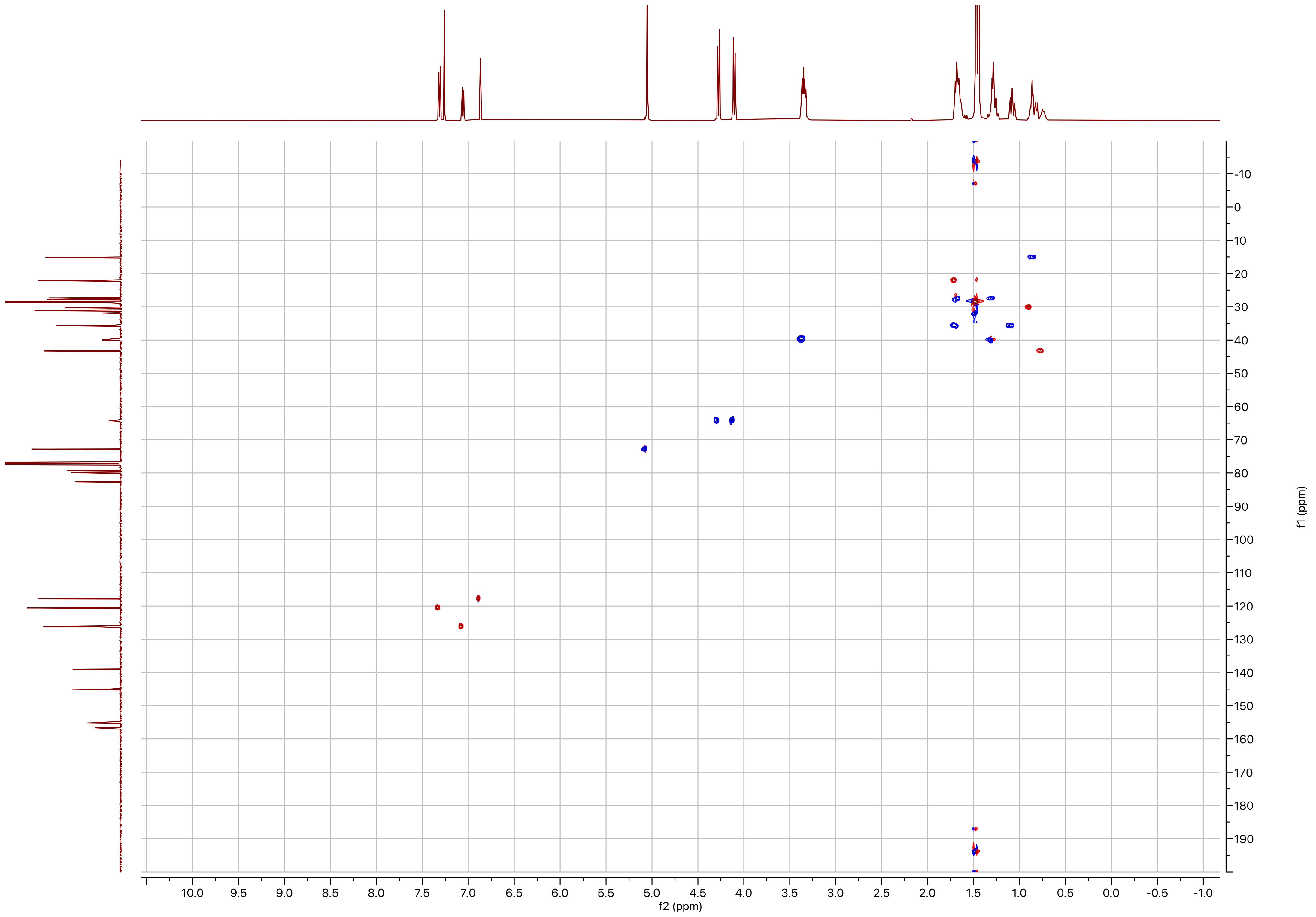

f2 (ppm)
f1 (ppm)

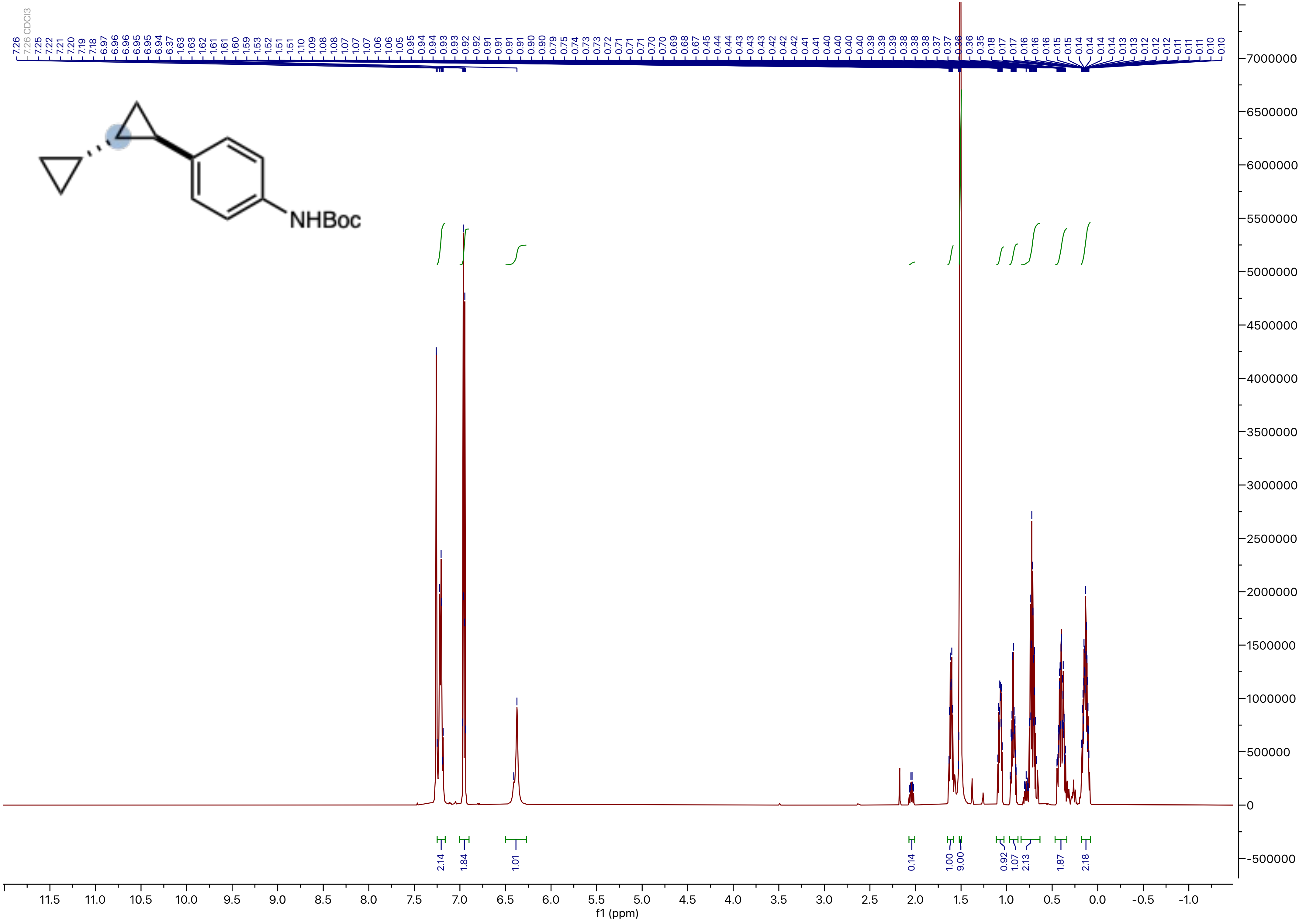
7.26, 7.26 CDCl3, 7.25, 7.22, 7.21, 7.20, 7.19, 7.18, 6.97, 6.96, 6.96, 6.95, 6.95, 6.94, 6.37, 1.63, 1.63, 1.62, 1.61, 1.61, 1.60, 1.59, 1.53, 1.52, 1.51, 1.51, 1.10, 1.09, 1.08, 1.08, 1.07, 1.07, 1.07, 1.06, 1.06, 1.05, 0.95, 0.94, 0.94, 0.93, 0.93, 0.92, 0.92, 0.91, 0.91, 0.91, 0.91, 0.90, 0.90, 0.79, 0.75, 0.74, 0.73, 0.73, 0.72, 0.71, 0.71, 0.71, 0.70, 0.70, 0.69, 0.68, 0.67, 0.45, 0.44, 0.44, 0.43, 0.43, 0.43, 0.42, 0.42, 0.41, 0.41, 0.40, 0.40, 0.40, 0.39, 0.39, 0.38, 0.38, 0.38, 0.37, 0.37, 0.36, 0.35, 0.18, 0.17, 0.17, 0.16, 0.16, 0.15, 0.15, 0.14, 0.14, 0.14, 0.14, 0.13, 0.13, 0.12, 0.12, 0.12, 0.11, 0.11, 0.10, 0.10
NHBoc
2.14
1.84
1.01
0.14
1.00
9.00
0.92
1.07
2.13
1.87
2.18
f1 (ppm)

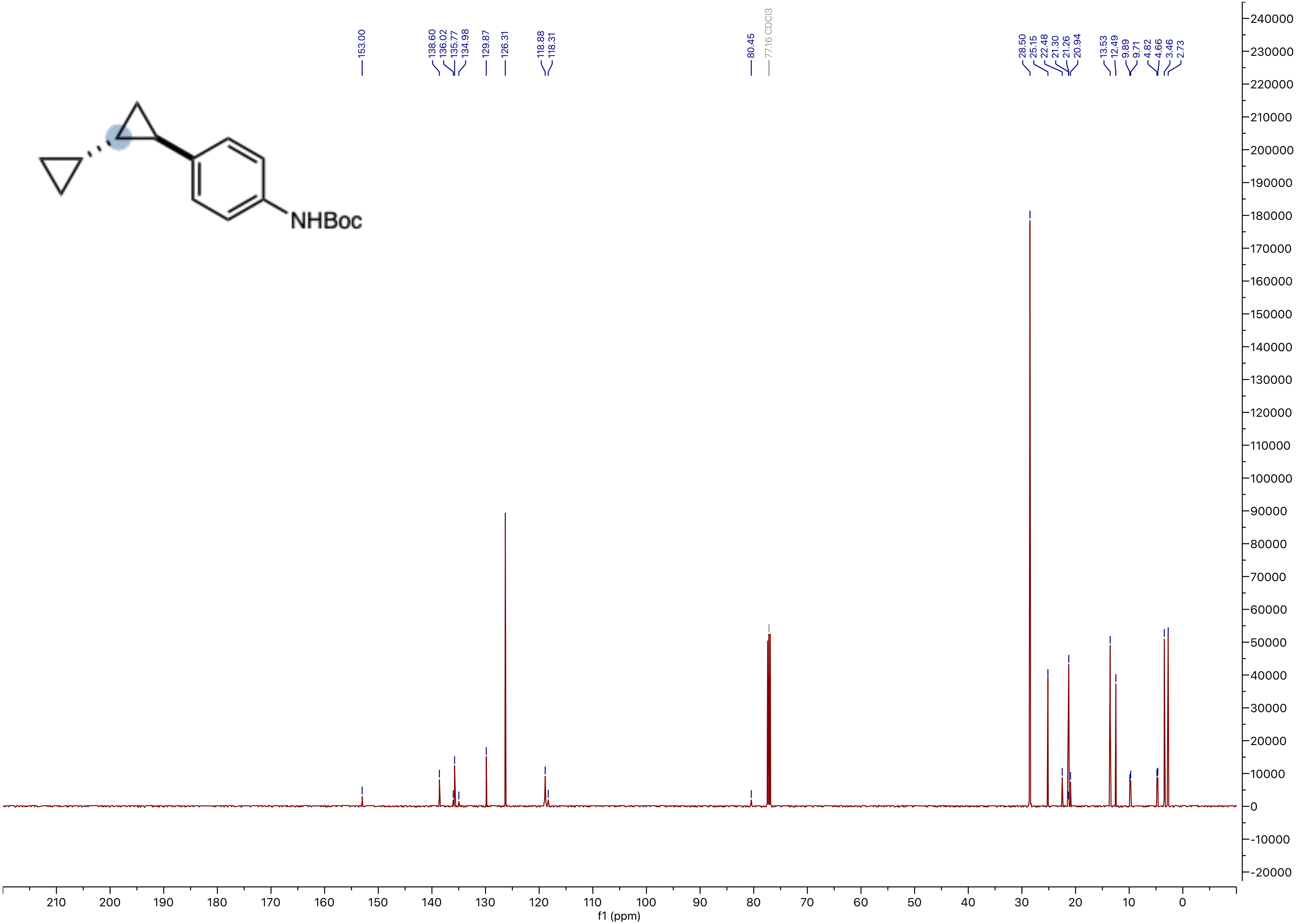
NHBoc
153.00
138.60
136.02
135.77
134.98
129.87
126.31
118.88
118.31
80.45
77.16 CDCl3
28.50
25.15
22.48
21.30
21.26
20.94
13.53
12.49
9.89
9.71
4.82
4.66
3.46
2.73
f1 (ppm)

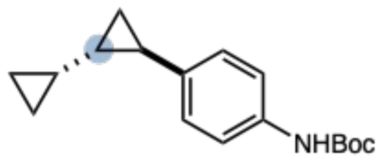
NHBoc

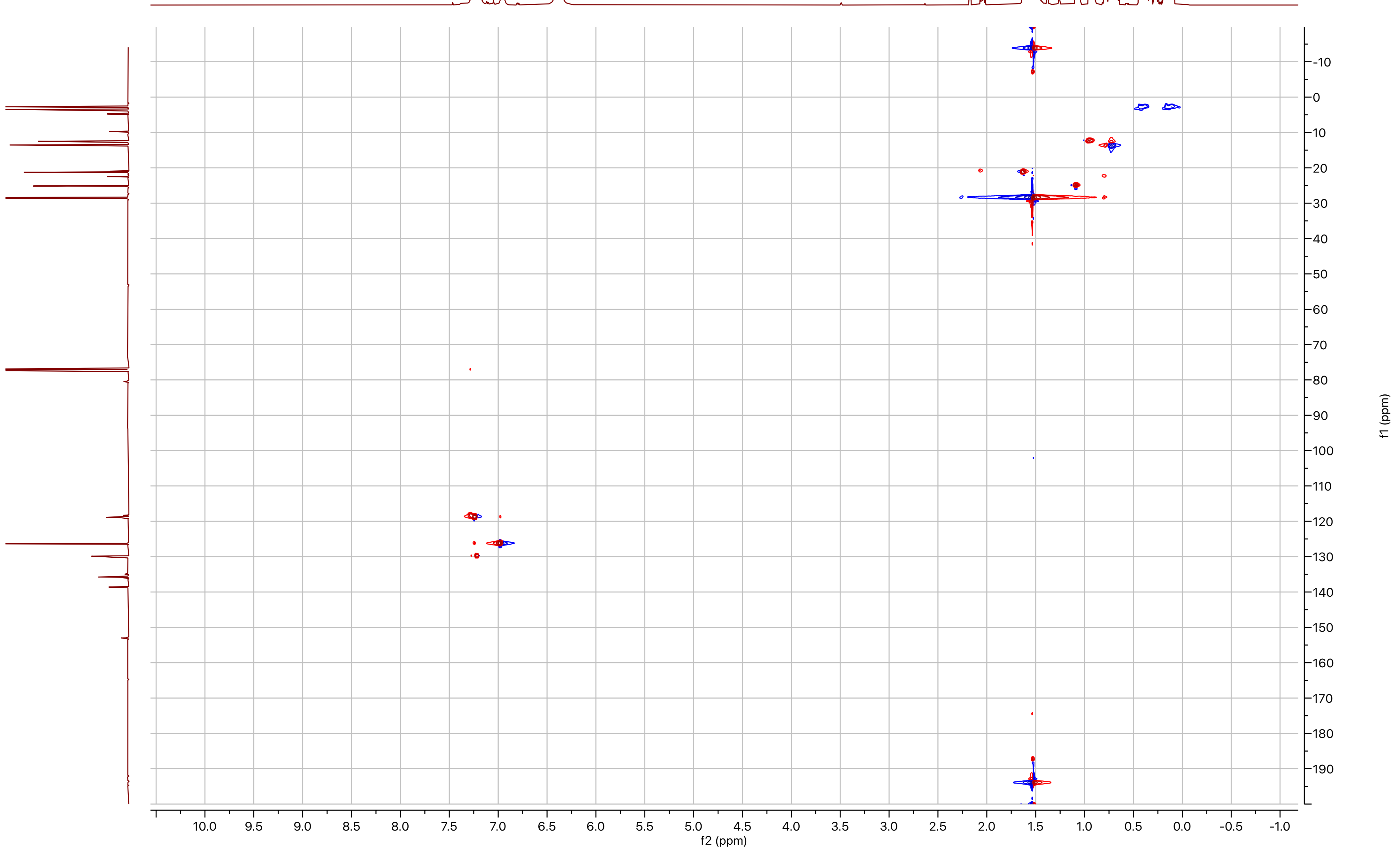
f2 (ppm)
f1 (ppm)

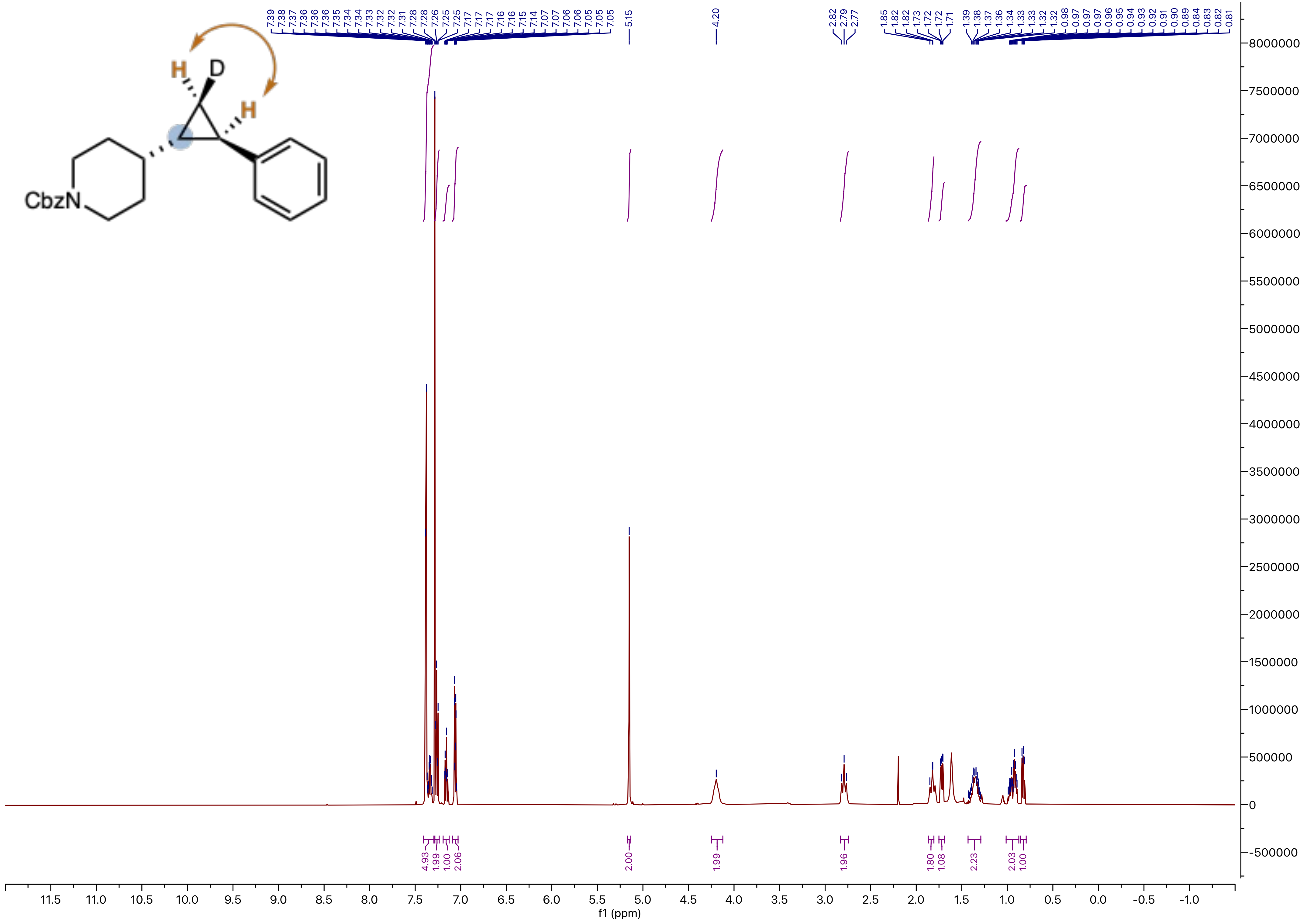

CbzN
H
D
H
f1 (ppm)

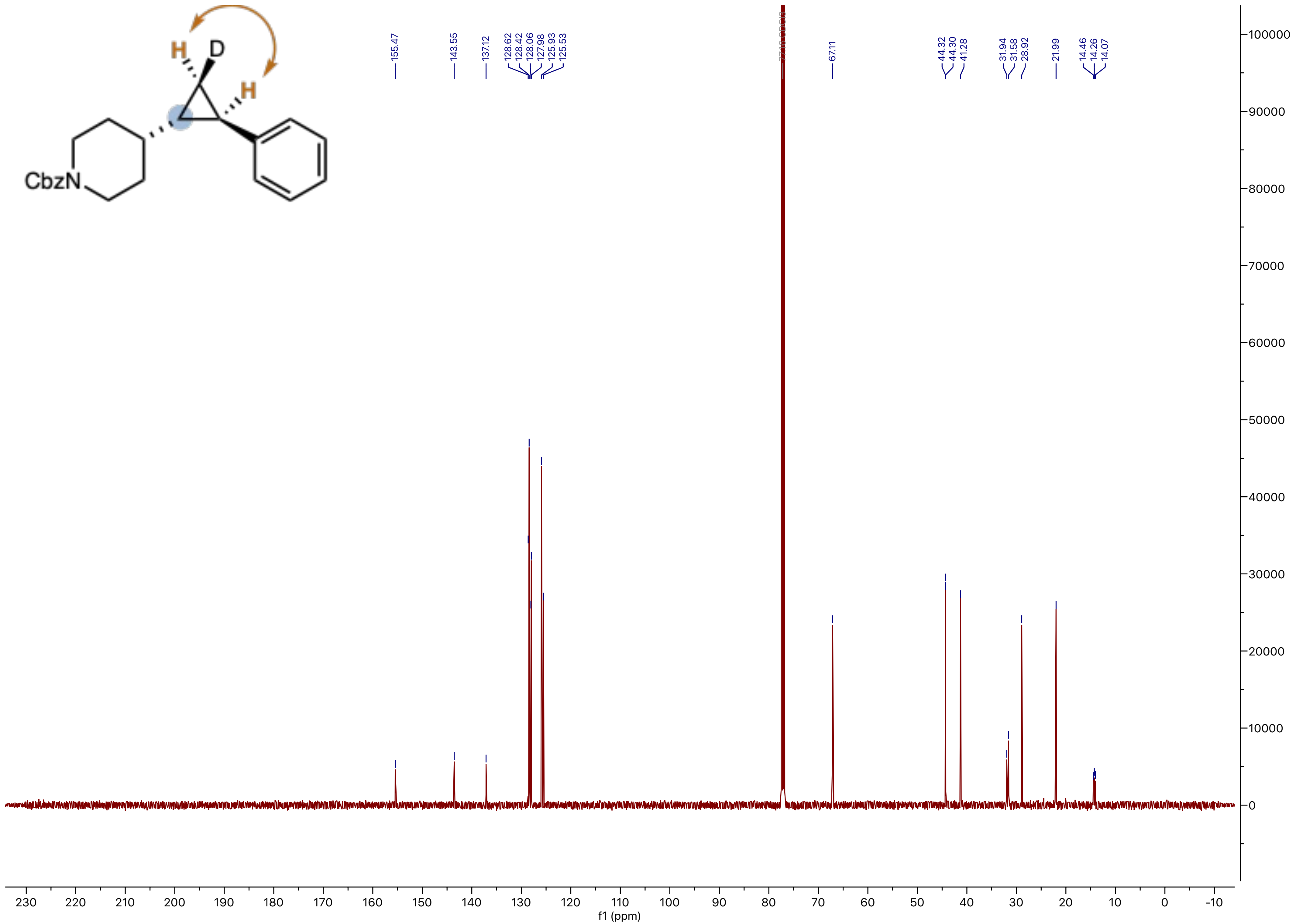

CbzN
H
D
H
155.47
143.55
137.12
128.62
128.42
128.06
127.98
125.93
125.53
67.11
44.32
44.30
41.28
31.94
31.58
28.92
21.99
14.46
14.26
14.07
100000
90000
80000
70000
60000
50000
40000
30000
20000
10000
0
230 220 210 200 190 180 170 160 150 140 130 120 110 100 90 80 70 60 50 40 30 20 10 0 -10
f1 (ppm)

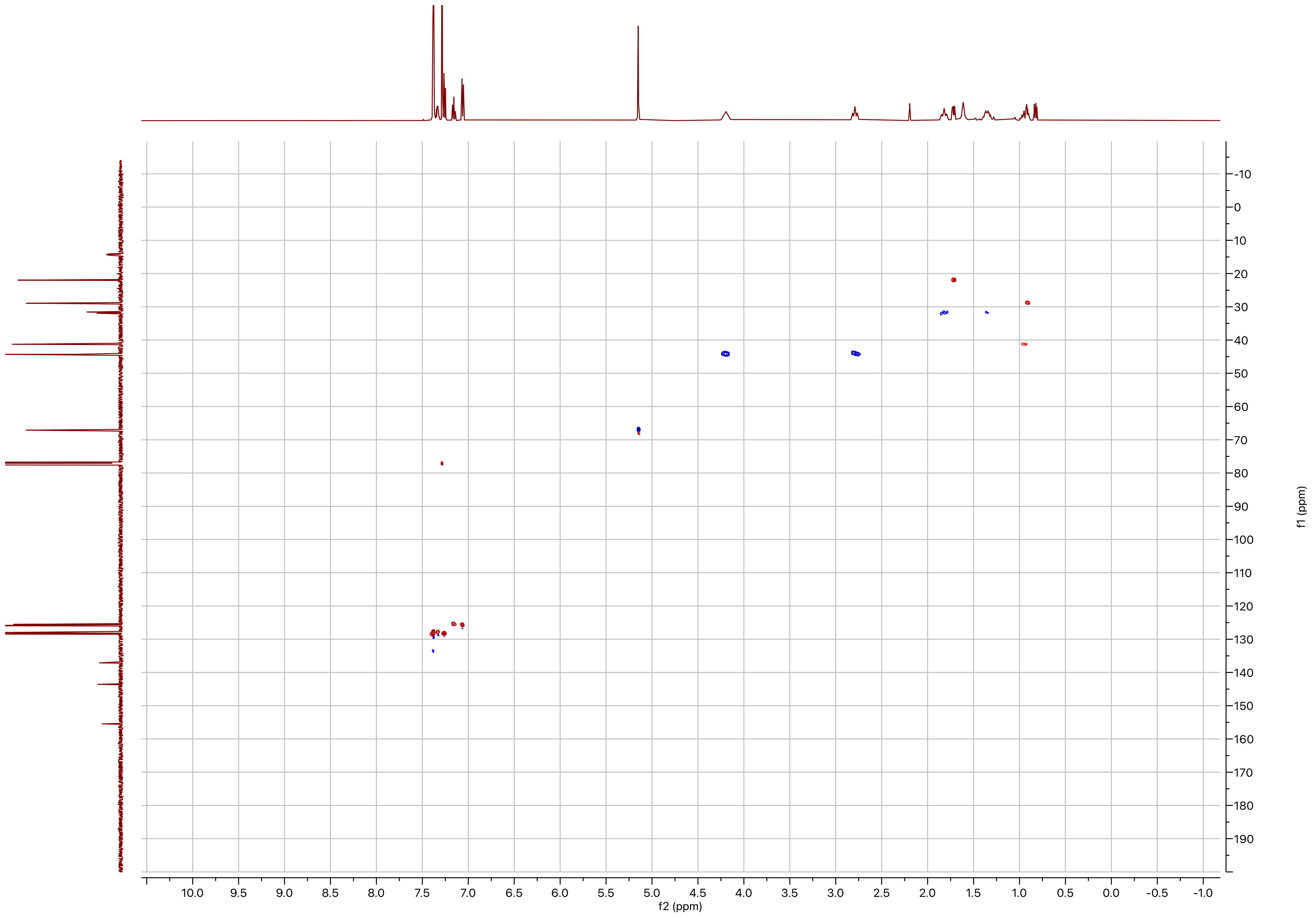

f2 (ppm)
f1 (ppm)

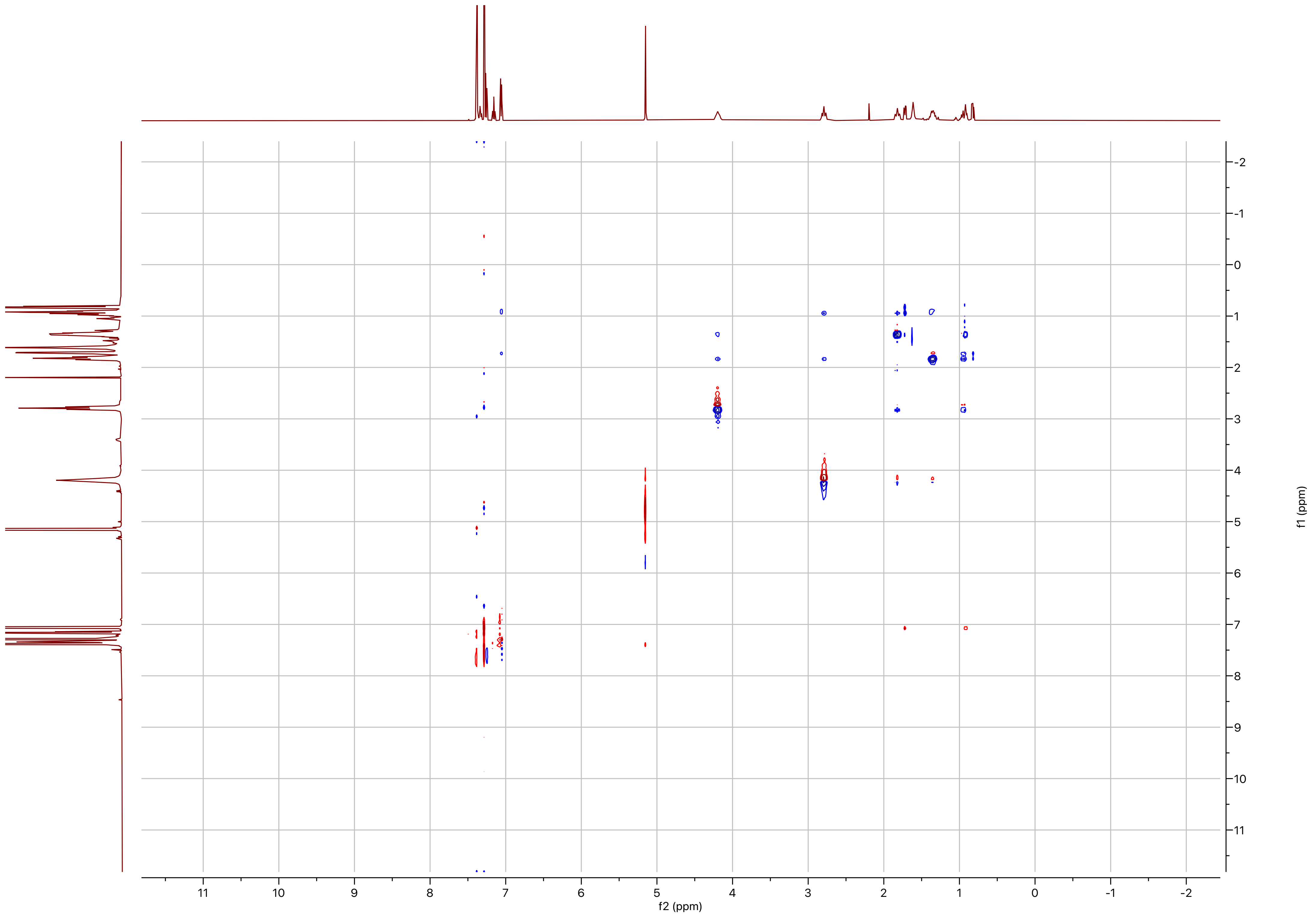

f2 (ppm)
f1 (ppm)

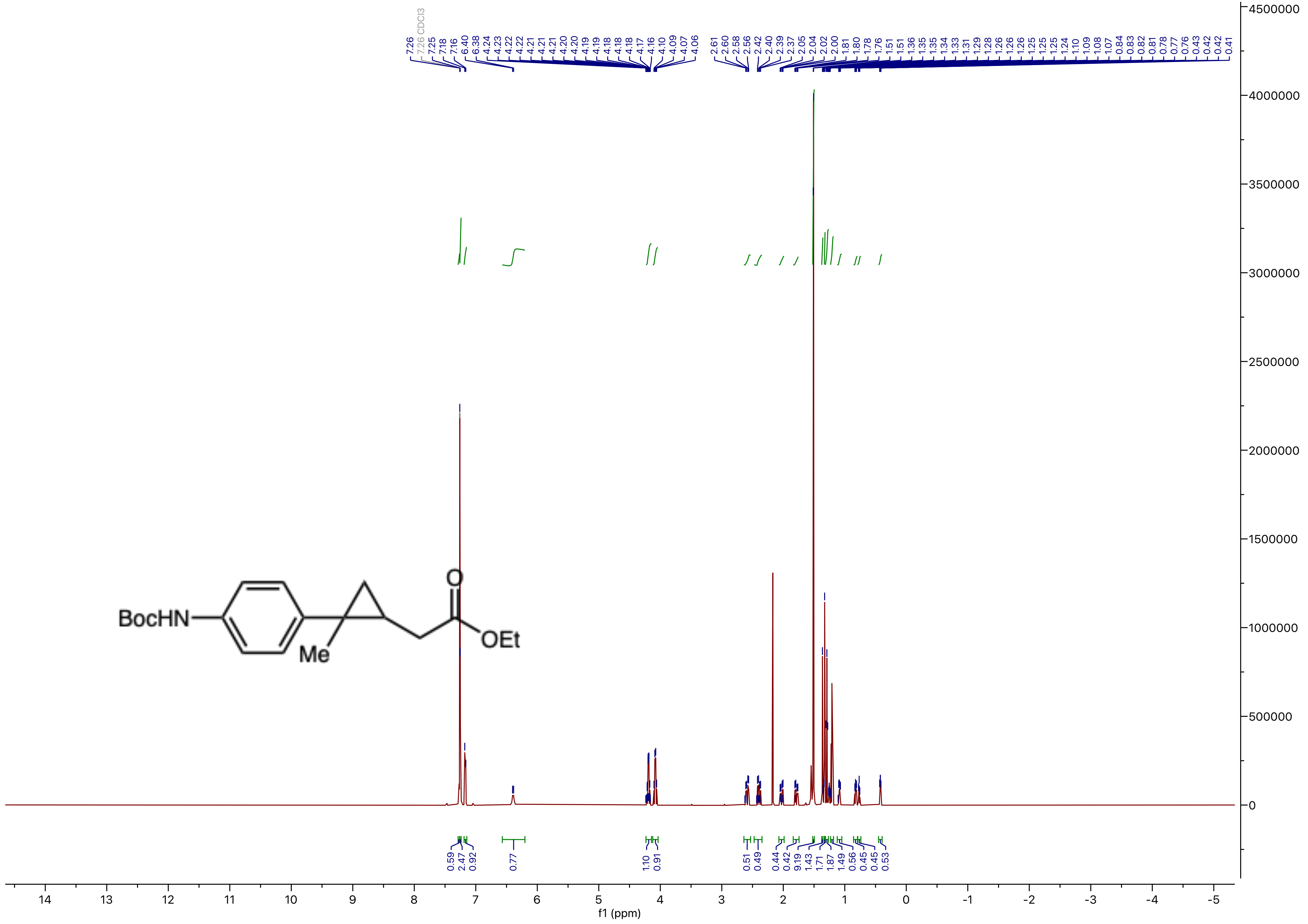

BocHN
Me
O
OEt
f1 (ppm)
CDCl3

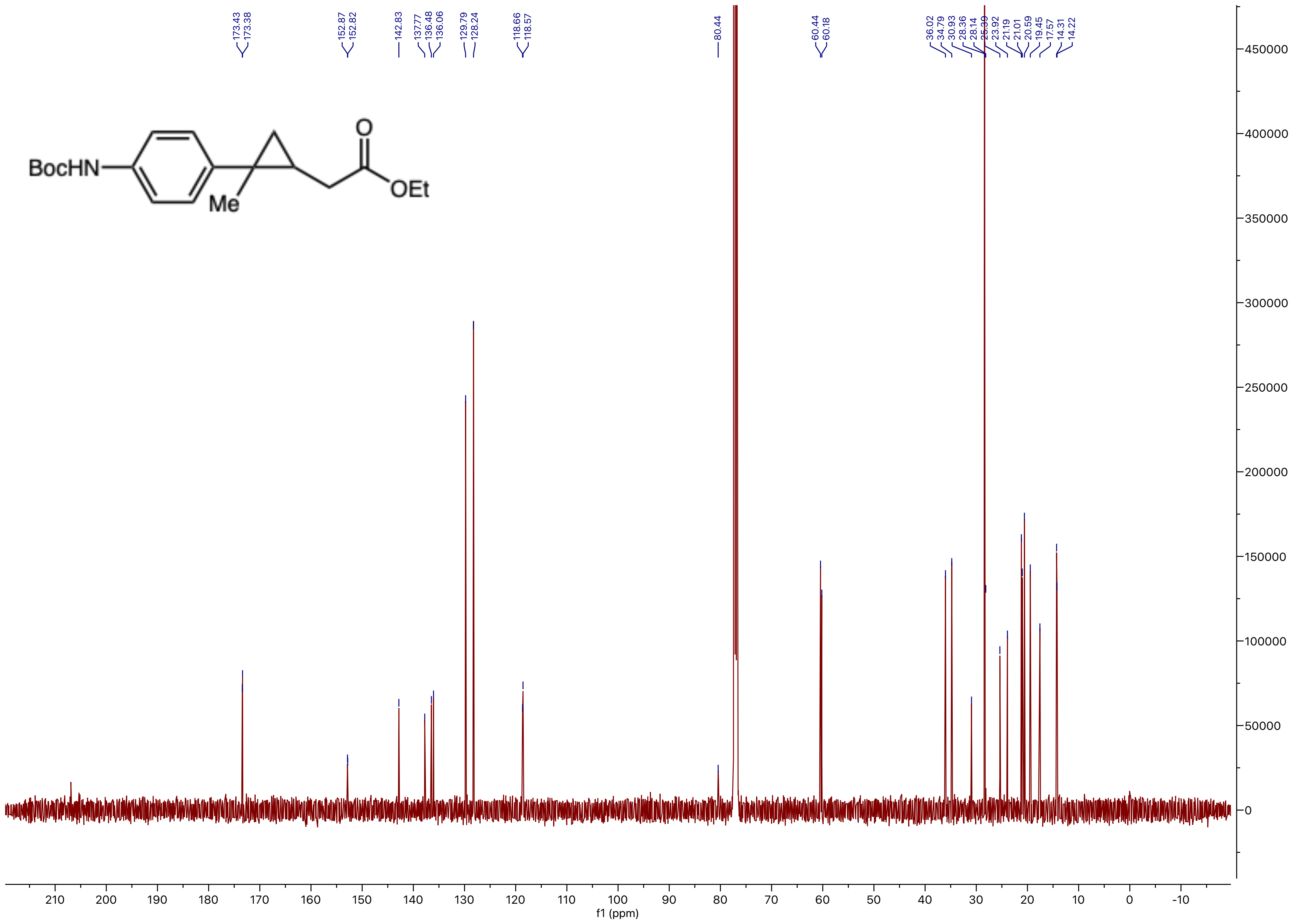

BocHN
Me
O
OEt
173.43
173.38
152.87
152.82
142.83
137.77
136.48
136.06
129.79
128.24
118.66
118.57
80.44
60.44
60.18
36.02
34.79
30.93
28.36
28.14
25.39
23.92
21.19
21.01
20.59
19.45
17.57
14.31
14.22
450000
400000
350000
300000
250000
200000
150000
100000
50000
0
210
200
190
180
170
160
150
140
130
120
110
100
90
80
70
60
50
40
30
20
10
0
-10
f1 (ppm)

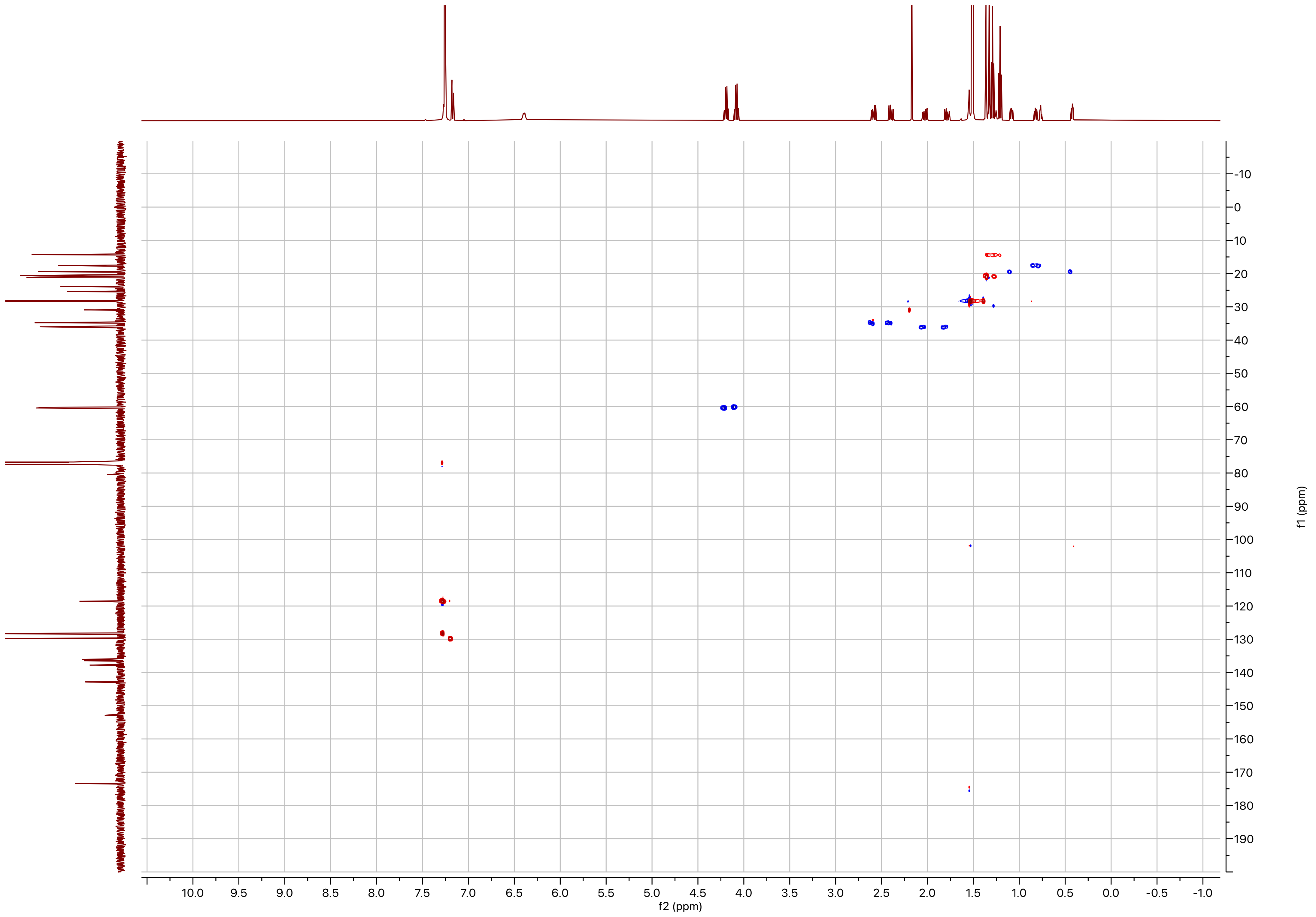

f2 (ppm)
f1 (ppm)

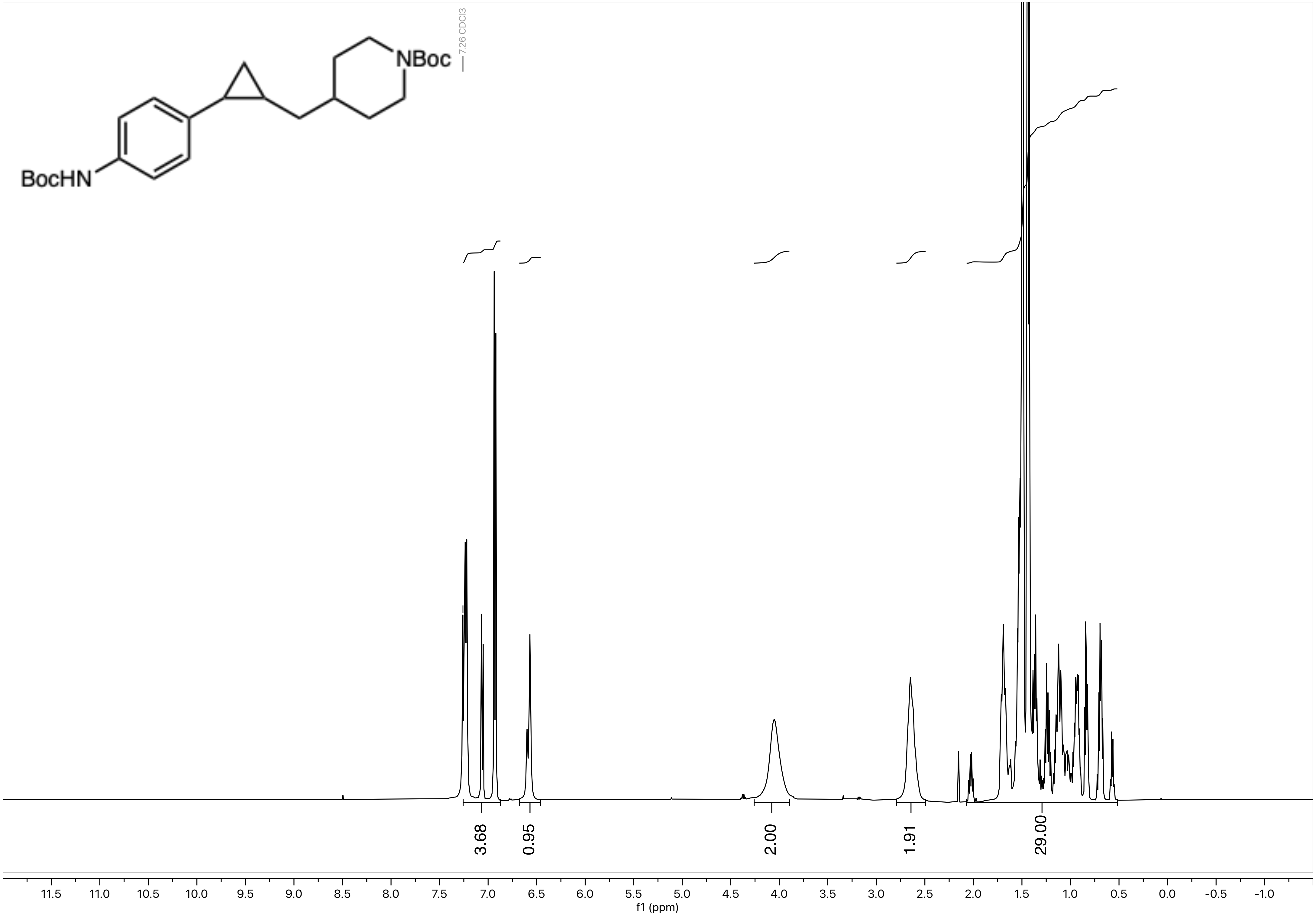
BocHN
NBoc
7.26 CDCl3
3.68
0.95
2.00
1.91
29.00
11.5 11.0 10.5 10.0 9.5 9.0 8.5 8.0 7.5 7.0 6.5 6.0 5.5 5.0 4.5 4.0 3.5 3.0 2.5 2.0 1.5 1.0 0.5 0.0 -0.5 -1.0
f1 (ppm)

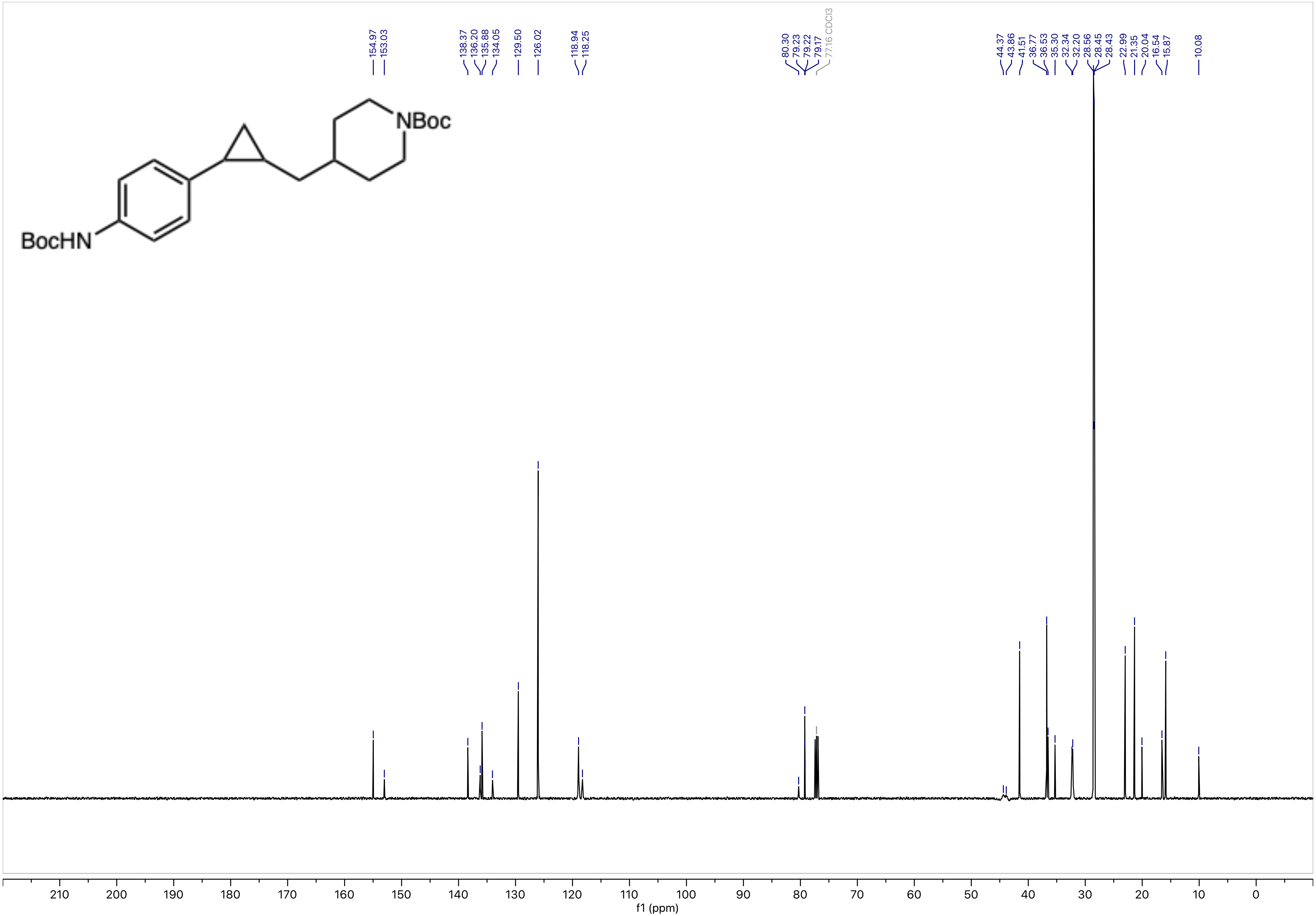
NBoc
BocHN
154.97
153.03
138.37
136.20
135.88
134.05
129.50
126.02
118.94
118.25
80.30
79.23
79.22
79.17
77.16 CDCl3
44.37
43.86
41.51
36.77
36.53
35.30
32.34
32.20
28.56
28.45
28.43
22.99
21.35
20.04
16.54
15.87
10.08
210
200
190
180
170
160
150
140
130
120
110
100
90
80
70
60
50
40
30
20
10
0
f1 (ppm)

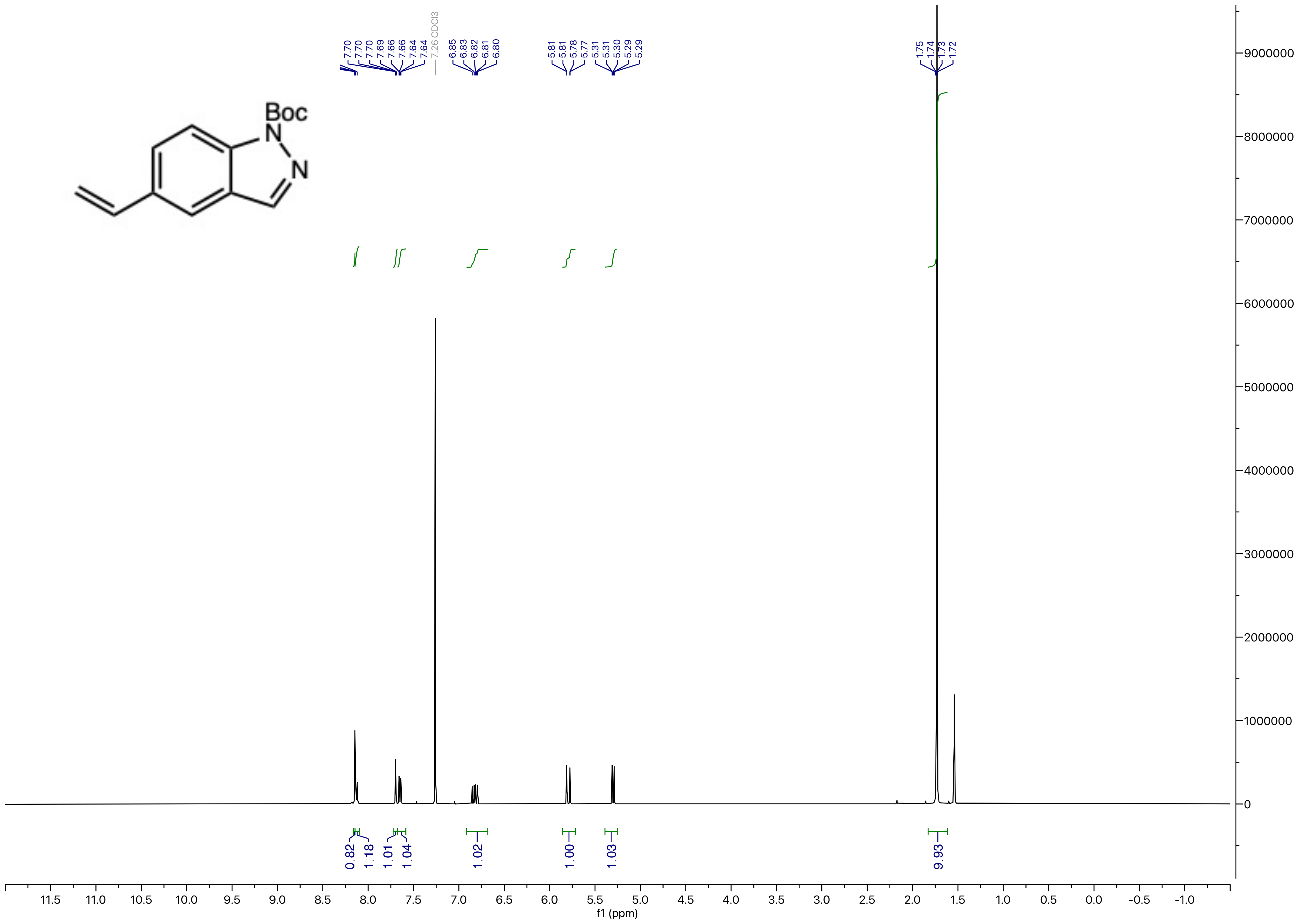

Boc
N
N
7.70
7.70
7.70
7.69
7.66
7.66
7.64
7.64
7.26 CDCl3
6.85
6.83
6.82
6.81
6.80
5.81
5.81
5.78
5.77
5.31
5.31
5.30
5.29
5.29
1.75
1.74
1.73
1.72
0.82
1.18
1.01
1.04
1.02
1.00
1.03
9.93
f1 (ppm)

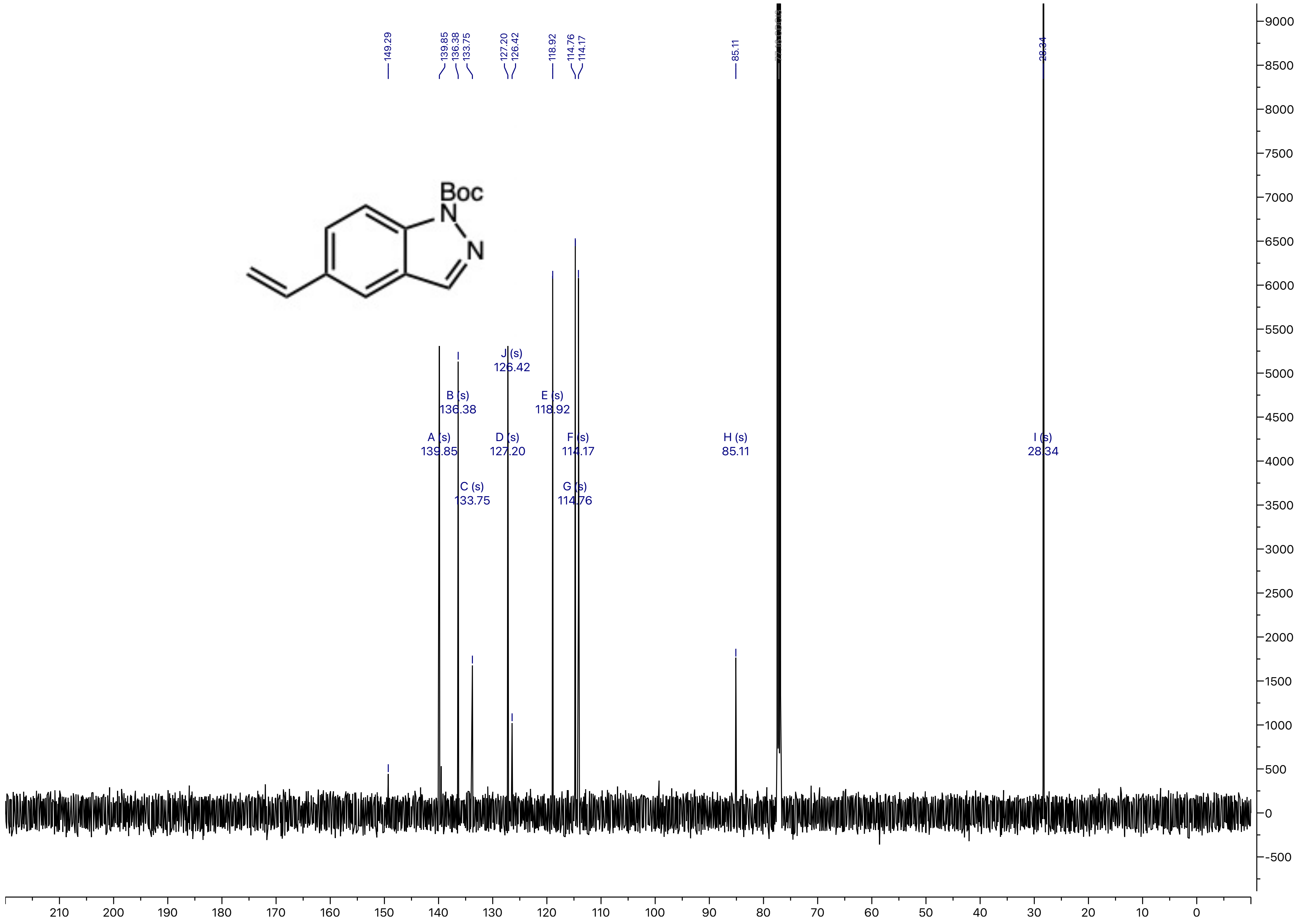

149.29
139.85
136.38
133.75
127.20
126.42
118.92
114.76
114.17
85.11
28.34
Boc
N
N
J (s)
126.42
B (s)
136.38
E (s)
118.92
A (s)
139.85
D (s)
127.20
F (s)
114.17
H (s)
85.11
I (s)
28.34
C (s)
133.75
G (s)
114.76
210 200 190 180 170 160 150 140 130 120 110 100 90 80 70 60 50 40 30 20 10 0
9000 8500 8000 7500 7000 6500 6000 5500 5000 4500 4000 3500 3000 2500 2000 1500 1000 500 0 -500

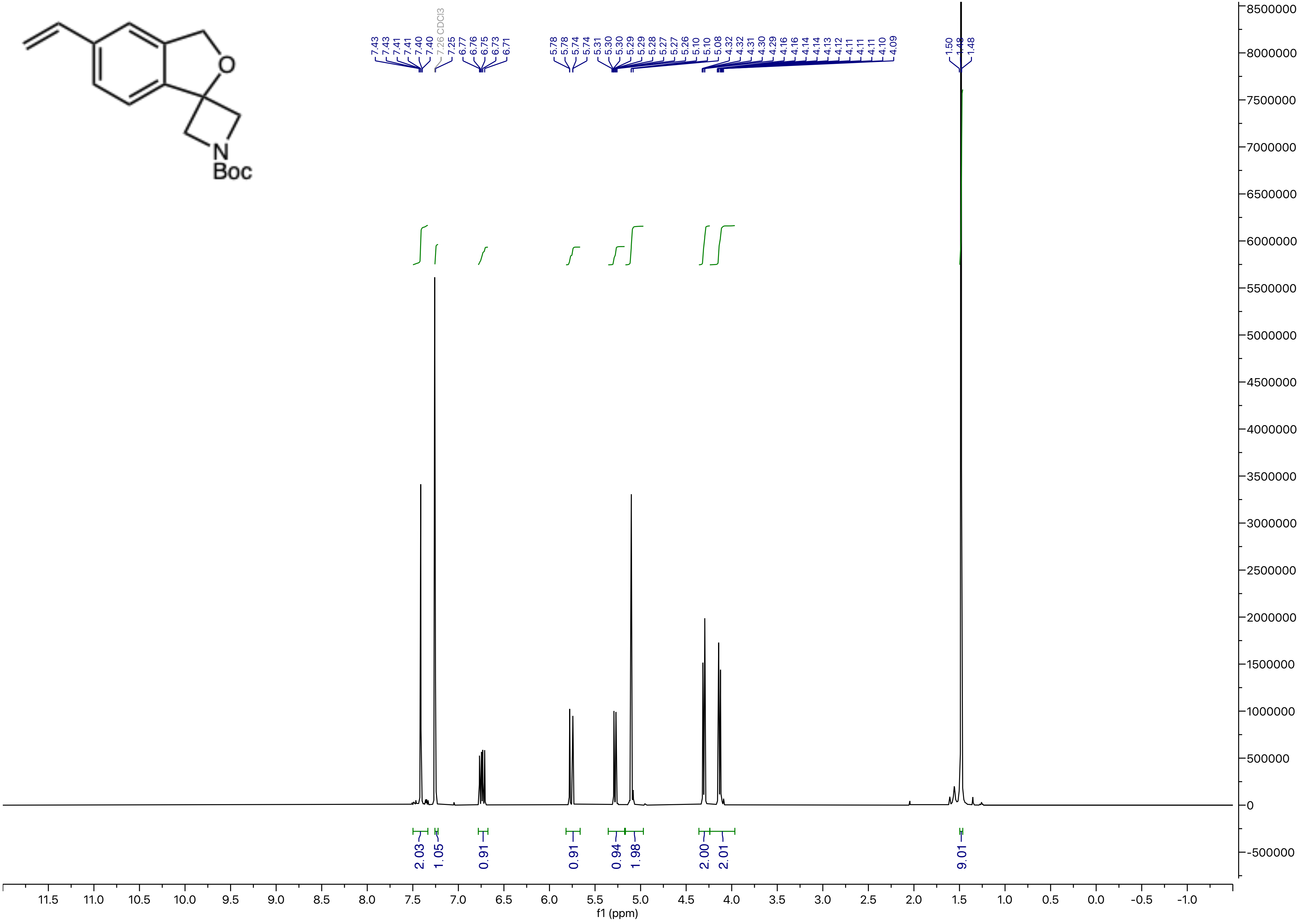

O
N
Boc
7.43
7.43
7.41
7.41
7.40
7.40
7.26 CDCl3
7.25
6.77
6.76
6.75
6.73
6.71
5.78
5.78
5.74
5.74
5.31
5.30
5.30
5.29
5.29
5.28
5.27
5.27
5.26
5.10
5.10
5.08
4.32
4.32
4.31
4.30
4.29
4.16
4.16
4.14
4.14
4.13
4.12
4.11
4.11
4.11
4.10
4.09
1.50
1.49
1.48
2.03
1.05
0.91
0.91
0.94
1.98
2.00
2.01
9.01
f1 (ppm)

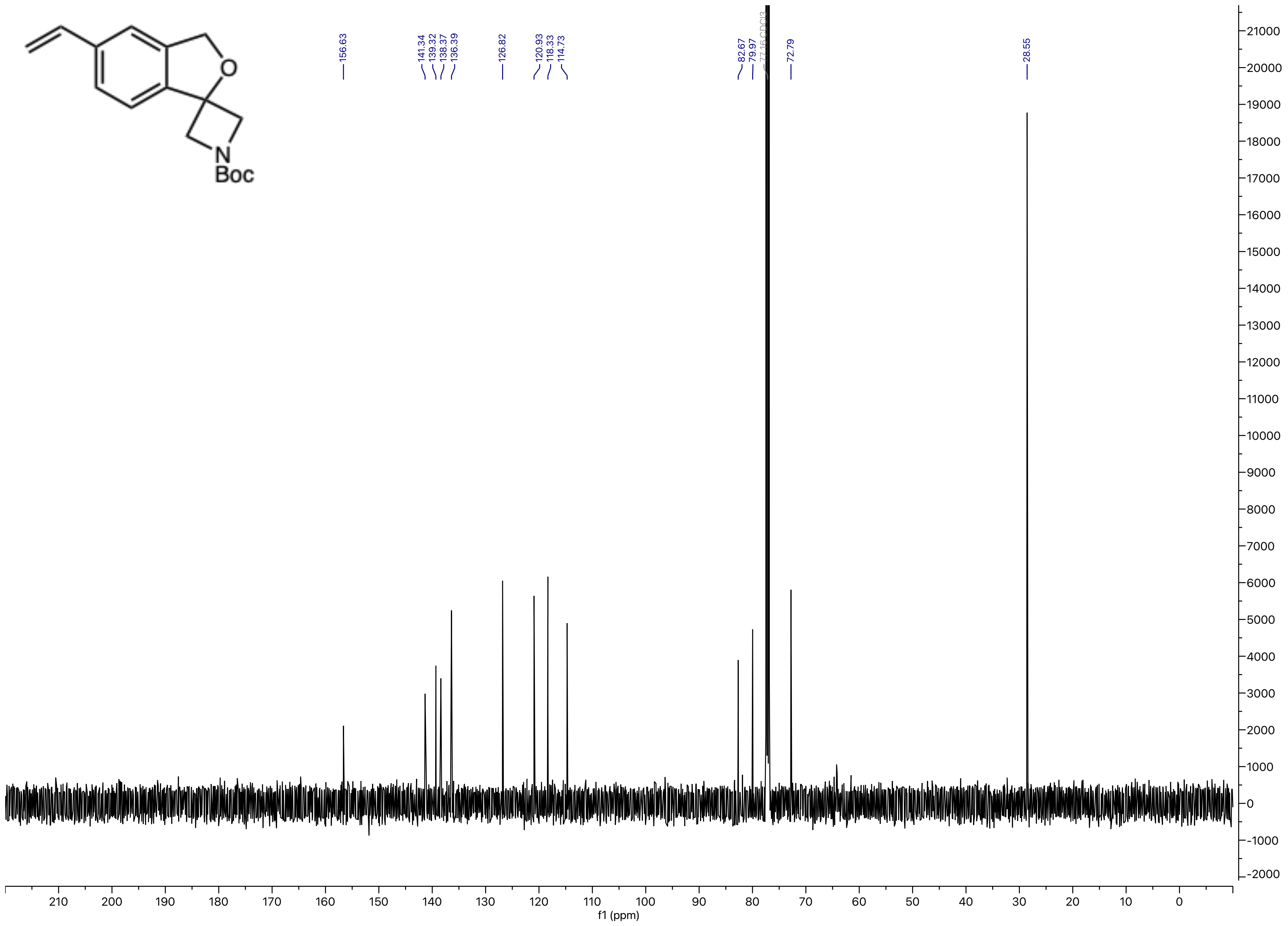

O
N
Boc
156.63
141.34
139.32
138.37
136.39
126.82
120.93
118.33
114.73
82.67
79.97
77.16 CDCl3
72.79
28.55
f1 (ppm)

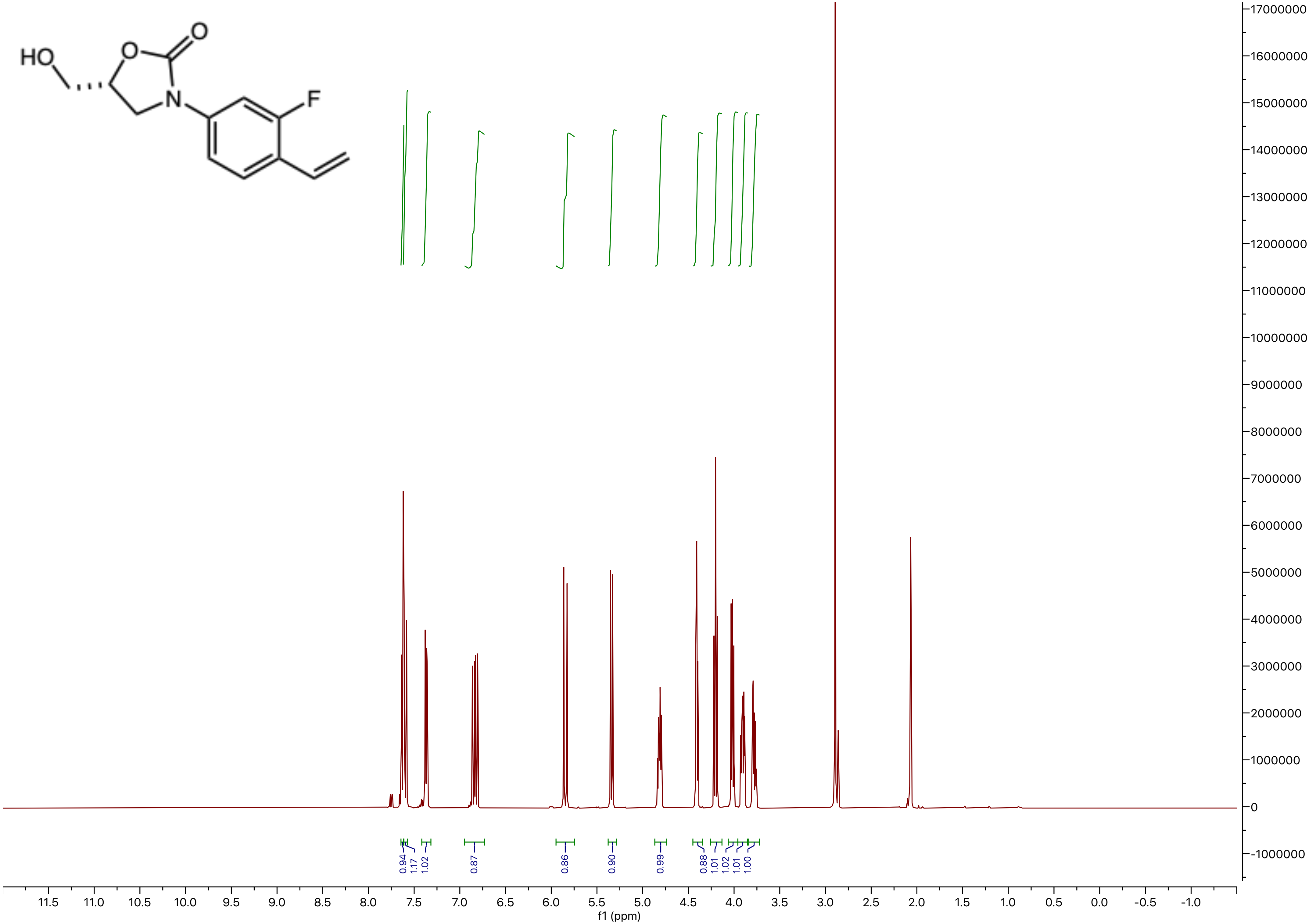
HO
O
O
N
F
0.94
1.17
1.02
0.87
0.86
0.90
0.99
0.88
1.01
1.02
1.01
1.00
f1 (ppm)

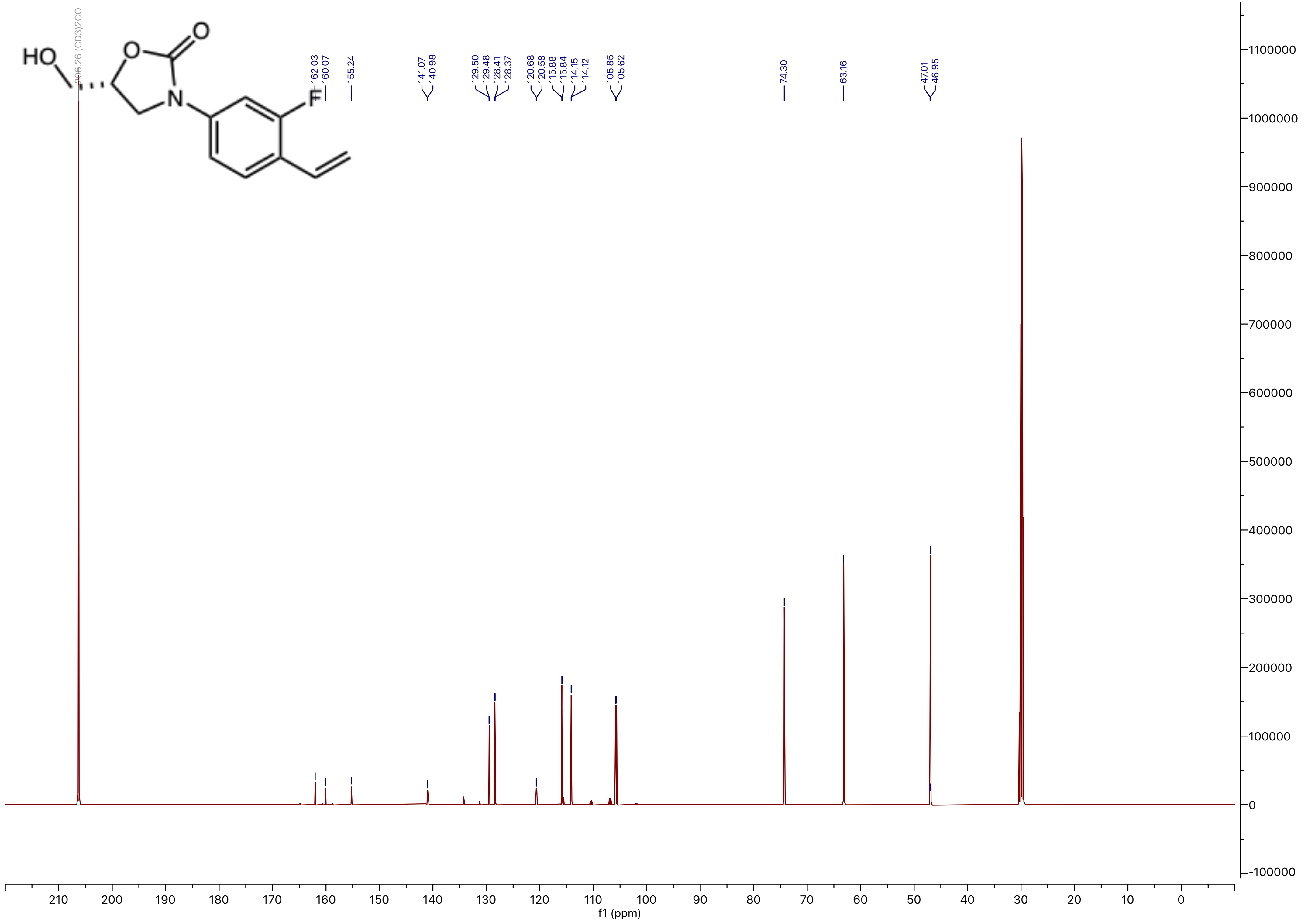

HO
O
O
N
F
206.26 (CD3)2CO
162.03
160.07
155.24
141.07
140.98
129.50
129.48
128.41
128.37
120.68
120.58
115.88
115.84
114.15
114.12
105.85
105.62
74.30
63.16
47.01
46.95
1100000
1000000
900000
800000
700000
600000
500000
400000
300000
200000
100000
0
-100000
210
200
190
180
170
160
150
140
130
120
110
100
90
80
70
60
50
40
30
20
10
0
f1 (ppm)

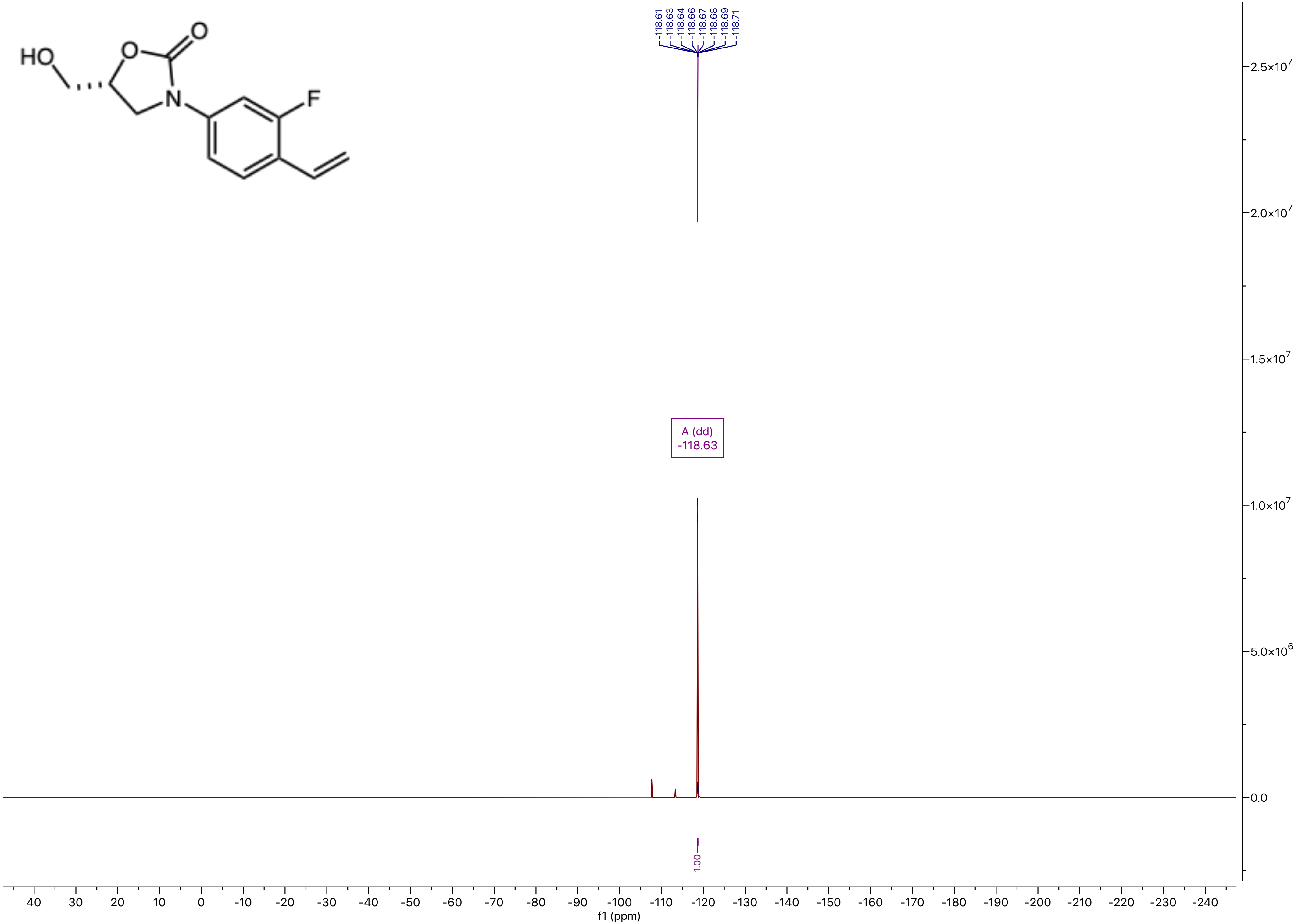

-118.61
-118.63
-118.64
-118.66
-118.67
-118.68
-118.69
-118.71
A (dd)
-118.63
1.00
2.5×10^7
2.0×10^7
1.5×10^7
1.0×10^7
5.0×10^6
0.0
40
30
20
10
0
-10
-20
-30
-40
-50
-60
-70
-80
-90
-100
-110
-120
-130
-140
-150
-160
-170
-180
-190
-200
-210
-220
-230
-240
f1 (ppm)

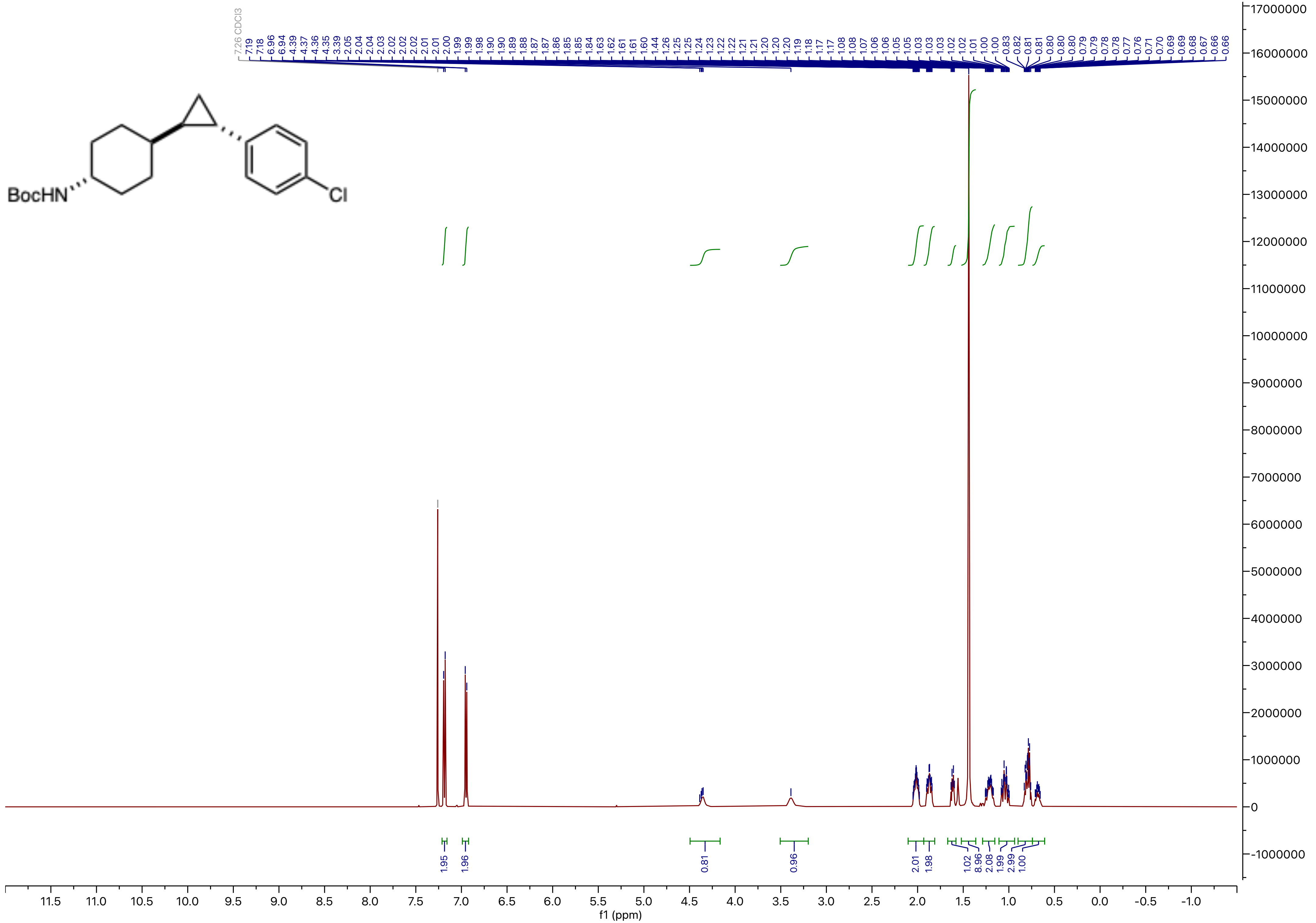

7.26 CDCl3
7.19 7.18 6.96 6.94 4.39 4.37 4.36 4.35 3.39 2.05 2.04 2.04 2.03 2.02 2.02 2.02 2.01 2.01 2.00 1.99 1.99 1.98 1.90 1.90 1.89 1.88 1.87 1.86 1.85 1.85 1.84 1.63 1.62 1.61 1.61 1.60 1.44 1.26 1.25 1.25 1.24 1.23 1.22 1.22 1.21 1.21 1.20 1.20 1.20 1.19 1.18 1.17 1.17 1.08 1.08 1.07 1.06 1.06 1.05 1.05 1.03 1.03 1.03 1.02 1.02 1.01 1.00 1.00 0.83 0.82 0.81 0.81 0.80 0.80 0.80 0.79 0.79 0.78 0.78 0.77 0.76 0.71 0.70 0.69 0.68 0.67 0.66 0.66
BocHN
Cl
1.95 1.96 0.81 0.96 2.01 1.98 1.02 8.96 2.08 1.99 2.99 1.00
f1 (ppm)

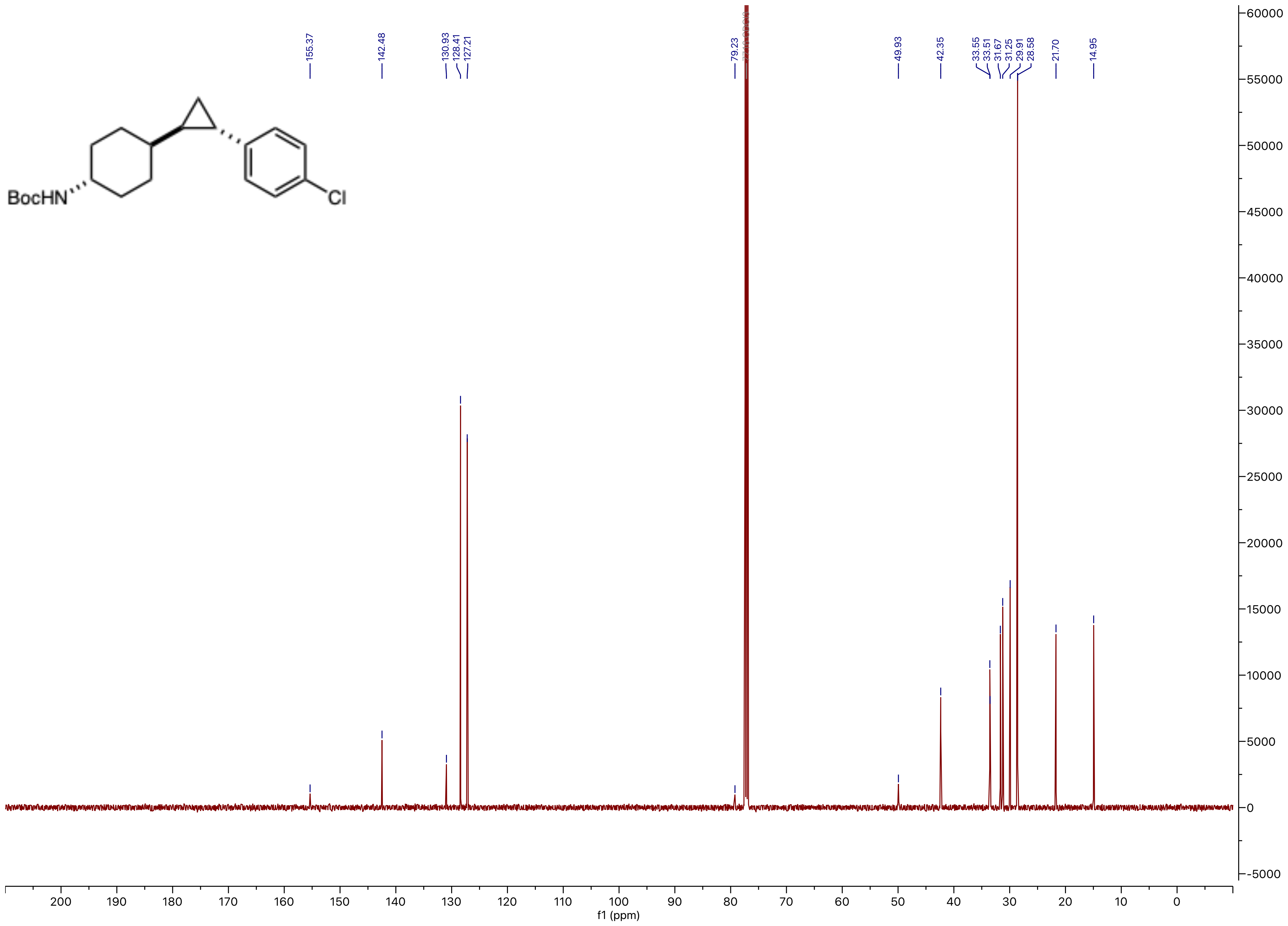

BocHN
Cl
155.37
142.48
130.93
128.41
127.21
79.23
49.93
42.35
33.55
33.51
31.67
31.25
29.91
28.58
21.70
14.95
f1 (ppm)
